# Key Role of Local Regulation in Chemosensing Revealed by a New Molecular Interaction-Based Modeling Method

**DOI:** 10.1371/journal.pcbi.0020082

**Published:** 2006-07-21

**Authors:** Martin Meier-Schellersheim, Xuehua Xu, Bastian Angermann, Eric J Kunkel, Tian Jin, Ronald N Germain

**Affiliations:** 1 Lymphocyte Biology Section and Program in Systems Immunology and Infectious Disease Modeling, Laboratory of Immunology, National Institute of Allergy and Infectious Diseases, National Institutes of Health, Bethesda, Maryland, United States of America; 2 Laboratory of Immunogenetics, National Institute of Allergy and Infectious Diseases, National Institutes of Health, Rockville, Bethesda, Maryland, United States of America; 3 BioSeek, Inc., Burlingame, California, United States of America; 4 Institute for Theoretical Physics, Faculty for Mathematics and Natural Sciences, University of Hamburg, Hamburg, Germany; Boston University, United States of America

## Abstract

The signaling network underlying eukaryotic chemosensing is a complex combination of receptor-mediated transmembrane signals, lipid modifications, protein translocations, and differential activation/deactivation of membrane-bound and cytosolic components. As such, it provides particularly interesting challenges for a combined computational and experimental analysis. We developed a novel detailed molecular signaling model that, when used to simulate the response to the attractant cyclic adenosine monophosphate (cAMP), made nontrivial predictions about *Dictyostelium* chemosensing. These predictions, including the unexpected existence of spatially asymmetrical, multiphasic, cyclic adenosine monophosphate–induced PTEN translocation and phosphatidylinositol-(3,4,5)P_3_ generation, were experimentally verified by quantitative single-cell microscopy leading us to propose significant modifications to the current standard model for chemoattractant-induced biochemical polarization in this organism. Key to this successful modeling effort was the use of “Simmune,” a new software package that supports the facile development and testing of detailed computational representations of cellular behavior. An intuitive interface allows user definition of complex signaling networks based on the definition of specific molecular binding site interactions and the subcellular localization of molecules. It automatically translates such inputs into spatially resolved simulations and dynamic graphical representations of the resulting signaling network that can be explored in a manner that closely parallels wet lab experimental procedures. These features of Simmune were critical to the model development and analysis presented here and are likely to be useful in the computational investigation of many aspects of cell biology.

## Synopsis

Cells can orient their migration in response to small local differences in the concentration of extracellular chemicals (chemoattractants). Understanding this process (chemosensing) requires analyzing the time and position-dependent behavior of the signaling molecules within the responding cell, making it an especially interesting challenge for both experimental and computational investigation. Here, the authors report the development and testing of a new detailed molecular model of the chemosensing apparatus of the amoeba Dictyostelium discoidium reacting to the chemoattractant cyclic adenosine monophosphate. Computer simulations performed using this model predicted unexpected and previously unreported patterns of changes in the concentration and location of two important intracellular signaling molecules. These predictions were experimentally verified using microscopy, suggesting the need for modifications to the current “standard” model of eukaryotic chemosensing. The high degree of detail in their model was made possible by a new software suite called “Simmune,” which allows biologists to enter information about molecular interactions using a graphical interface. Without requiring the user to write any equations, the software automatically constructs the overall reaction network, simulates the model, and provides several ways to view the biochemistry of simulated cells. This new tool should help biologists to translate qualitative representations of cell biological processes into quantitative, predictive models.

## Introduction

The ability of eukaryotic cells to process nonisotropic extracellular stimuli lies at the heart of many aspects of cellular behavior including directed cell growth, movement, and cell-cell communication [1–4]. When an external stimulus is localized to a discrete patch of membrane, for example, during contact-dependent cell-cell communication, the physical recruitment of signaling components into receptor-associated multimolecular complexes provides a straightforward mechanism for establishing the appropriate directionality of intracellular responses. Under these circumstances, it is readily apparent how the activation of excitatory components at response onset and of inhibitory components during negative regulation (sometimes called *adaptation*) is constrained to a specific region of the cell.

In contrast, during chemotaxis (directed movement along a chemical gradient) cells display a very strong intracellular biochemical polarization even though the external stimulus may surround the entire cell and differ by only a few percent in concentration at different points along the membrane. Here, the principle of physical locality invoked in the case of cell-cell contact does not seem to provide an obvious mechanism for the translation of small extracellular directional clues into an almost digital biochemical, cytoskeletal, and morphological polarization of the responding cell. The striking capacity of cells to reliably sense and respond to minute gradients has led to intense experimental analysis at the cell biological level as well as to development of quantitative mathematical models of the chemosensing phenomenon.

Many of the core components of the underlying signaling network have been elucidated in several cell types, especially neutrophils and the amoeba of Dictyostelium discoidium [5–7]. A central role is played by the phospholipid phosphatidylinositol-(3,4,5)P_3_ (PIP_3_), the product of the action of the lipid kinase phosphoinositide 3-kinase (PI3K) on the relatively abundant membrane component phosphatidylinositol-(4,5)P_2_ (PIP_2_). The local concentration of PIP_3_ is determined by the competing actions of PI3K and the phosphatase PTEN (phosphatase and tensin homolog deleted on Chromosome 10) that dephosphorylates PIP_3_ at the 3′ position to produce PIP_2_ [8]. PIP_3_ functions as a membrane anchor for signaling proteins with pleckstrin-homology (PH) domains [9]. Such proteins include several that regulate the polymerization of actin, which, in turn, is needed for the extension of pseudopods/lamellipodia that allow cells to crawl [10]. For this reason, localized PIP_3_ accumulation is frequently used as a read-out of polarization in response to chemoattractant gradients. In spite of this progress in defining the main molecular players and their interactions, however, we do not yet fully understand how eukaryotic cells are able to amplify the primary receptor signals induced by shallow extracellular gradients of chemoattractants into steep intracellular gradients of signaling molecules like PIP_3_. Nor is it known precisely how the chemosensing signaling machinery adapts to attractant stimulations or what the connection is between the mechanisms that lead to amplification and those that lead to adaptation.

The dominant hypothesis embodied in existing conceptual and computational models for how cells achieve polarization in the face of shallow chemoattractant gradients is termed “local excitation – global inhibition” [6]. Meinhardt and Gierer [11,12] in particular showed that combinations of localized positive feedback and long-range inhibition could potentially produce the biochemical inhomogeneity encountered during chemotactic responses, and variations on this theme appear in the other models in this class [13,14]. The main idea is that polarization results from a combination of local activation processes and one (or more) globally acting, signal-induced inhibitor(s) tuned to be just strong enough to suppress the local responses everywhere except in those regions of the cell where the extracellular concentration of the stimulus is highest. The globally acting inhibitor, whose level is determined by the overall occupancy of the chemoattractant receptors, indirectly plays the role of a messenger that provides cell-wide information about the average extracellular chemoattractant concentration. This average is the standard used by the cell to determine which side is experiencing “high” and which side “low” relative chemoattractant concentrations. Adaptation, in this context, is the failure of local signals to significantly rise above the average and overcome the induced level of global inhibition.

While these modeling efforts have had a major influence on the way investigators view the molecular basis for chemosensing, the field still lacks a model that reflects to the greatest extent possible the detailed biochemistry revealed by bench experiments and whose predictions have been widely validated by experimental tests at the single cell level. For example, in *Dictyostelium,* stimulation with homogeneous fields of the chemoattractant cyclic adenosine monophosphate (cAMP) produces a fast rise in PIP_3_ around the entire cell circumference, followed by a rapid return to (or below) prestimulus level [6,15]. PTEN shows the inverse pattern, leaving the membrane upon exposure to cAMP and then returning during the adaptation phase [7]. However, the concentration of membrane-bound PTEN is still considerably below its prestimulus level at the time when the PIP_3_ concentration peaks and starts to decay. Given the current belief that in these cells, PI3K is the only generator of PIP_3_ and PTEN is the most important enzyme metabolizing PIP_3_, the point at which PIP_3_'s concentration in the membrane peaks must mark the moment at which the activities of PI3K and PTEN are temporarily in equilibrium. The cAMP signal must therefore rapidly activate a component that negatively regulates PI3K so that its activity is balanced by the lower than resting level of PTEN present at the membrane at this point in time. Such a negative regulatory component is not explicitly included in existing models of *Dictyostelium* chemosensing.

In part, the absence of such specific components in previous computational treatments arises from a tendency to use abstract modules in models of complex biological signaling networks whenever the details are incompletely understood. Although such approaches are in part guided by the principle of parsimony, another, more practical reason for the introduction of abstract modules is that the literature mining involved in the creation of complex biological signaling models and their translation into computational representations can be quite difficult.

Here we report a two-pronged approach to the development of a more complete and explicit model of *Dictyostelium* chemosensing. First, at the biochemical level, we started with a basic chemosensing model containing only the principal molecular players incorporated into the current paradigm in the field. We then tried to fill in “logical” blanks (such as the negative regulator of PI3K discussed above) so as to be able to reproduce the experimentally reported dynamical behaviors of PIP_3_, PTEN, and PI3K upon exposure of a morphologically unpolarized *Dictyostelium* cell to chemoattractant exposure [7,16]. Our specific aim was to construct a model that did not contain abstract “black-box” modules but instead used specific molecular components and interactions reported in the experimental literature to provide necessary functions (positive feedback, negative regulation, amplification).

Second, to help achieve this goal of molecular detail, we utilized a new software approach called Simmune that was created to facilitate the development and simulation of realistic, and therefore frequently quite complex, cell-biological models. Originally developed because of an interest in simulating immune responses, Simmune has no attributes that are unique to immunology and it is applicable to the simulation of any cell-biological system. The software suite allows molecular reactions to be defined directly at the level of interactions between molecular binding sites, using simple graphical representations of molecules and molecular complexes. This permits expert biologists to construct and simulate complex models without direct involvement in the underlying mathematics.

The signaling network that we developed through literature mining and the application of Simmune shares some properties with the abstract model developed by Meinhardt [12], but it goes further by providing an explicit molecular definition of the feedback module and the inhibitory regulator postulated in this earlier work. Our model also includes the action of a second, slower inhibitory component that is reminiscent of Meinhard's “poisoning” element. This element was introduced to prevent cells from locking into the direction of given stimulus and becoming blind to subsequent changes in direction and strength of the external signal. However, the present scheme differs in important aspects from this previous proposal and from the “local excitation–global inhibition” models suggested by Devreotes and coworkers [14] because the locally acting feedback mechanism and the distribution of the inhibitory component (PTEN) are now coupled.

Simulations performed with our model predicted previously unrecognized biphasic spatiotemporal changes in PIP_3_ and PTEN localization and concentration in *Dictyostelium* cells responding to cAMP gradients. These predictions were confirmed across a range of gradient conditions by single-cell imaging studies, leading us to propose a substantial modification to the standard “local excitation–global inhibition” model, with locally acting negative feedback now seen as playing a key role in controlling the development of chemoattractant-induced biochemical polarity.

## Results

**Figure 1:**
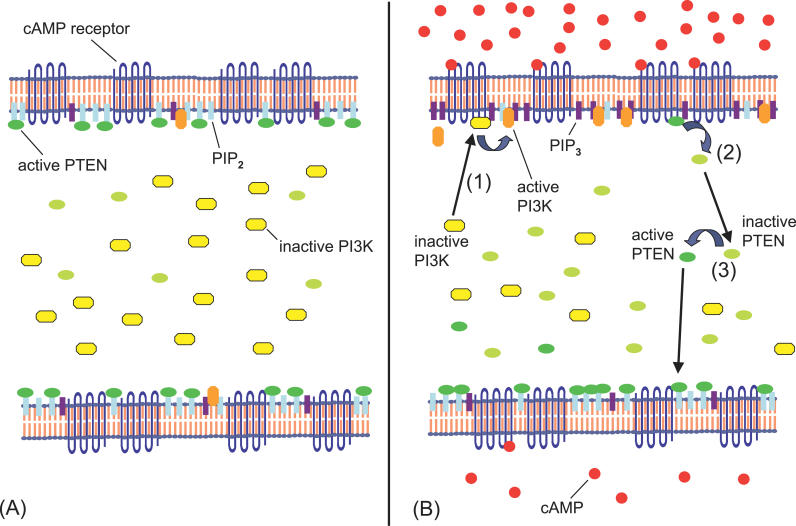
Standard Model of the Extracellular and Intracellular Distribution of Key Components of *Dictyostelium* Chemotactic Signaling (A) In an unstimulated cell PTEN is homogeneously distributed at the membrane. The cell membrane contains very little PIP_3_. (B) Stimulation of the cell leads to the membrane recruitment and activation of PI3K, as indicated by the arrows (1) leading from inactive, mainly cytosolic PI3K (yellow) to membrane-proximal, active PI3K (orange). Activated PI3K transforms PIP_2_ into PIP_3_. PTEN is deactivated following cAMP stimulation and leaves the membrane. This process is indicated by arrows (2) connecting active PTEN (dark green) and the mainly cytosolic inactive PTEN (light green). Regulatory processes lead to reactivation of PTEN (3). Differences in the speed and degree of cAMP receptor ligation between front and back of the cell lead to preferential accumulation of PTEN at the back of the cell. As a result, the front experiences a higher concentration of PI3K and a lower concentration of PTEN than the back and accumulates PIP_3_. Note: To emphasize the changes in PIP_3_ content, the amount of PIP_3_ relative to that of PIP_2_ has been overstated. Even after cAMP stimulation, the actual amount of PIP_2_ will be much higher than that of PIP_3_.

In constructing a new detailed model of *Dictyostelium* cAMP chemosensing, we needed to ensure that the major features of the cellular response to chemoattractant exposure would be present in the simulated behavior of a cell. First, when exposed to a uniform concentration of attractant, the model should show a transient membrane response that rapidly reverts to the resting state (global adaptation). Second, when exposed to a shallow gradient of attractant, the simulated cell should generate a much steeper intracellular biochemical gradient (amplification). The starting point for the construction of a model with these features was the well-established biochemical scheme described in [8] and [17] (Figure 1). Ligand binding to the cAMP receptor leads to activation of the associated Gαβγ, yielding Gα and Gβγ. These effectors, in turn, promote the activation of Ras and the membrane recruitment and activation of PI3K, along with a G protein–dependent translocation of PTEN from the membrane to the cytosol. The combination of PI3K activation and a loss of membrane-bound PTEN results in a rapid increase of PIP_3_ upon stimulation of the cells with cAMP (Figure 1).

### Development of a Refined Signaling Model of Chemosensing Based on Quantitative Live Cell Imaging and Literature Mining

Fusion molecules containing both a PIP_3_-specific PH domain and a fluorescent protein domain have been produced to monitor the dynamics of the distribution of PIP_3_ under various modes of *Dictyostelium* exposure to chemoattractant [1,18,19]. When stimulated with a homogeneous field of cAMP, previously unpolarized cells respond with a rapid, transient increase of PIP_3_ around their entire perimeter [6,20]. In contrast, upon exposure to gradients of chemoattractant, the plasma membrane concentration of PIP_3_ increases throughout the cell initially and then decays everywhere to below the pre-stimulus level, except for the side of the cell exposed to the higher chemoattractant concentration [6,15]. Using a fluorescent fusion of PTEN (GFP [green fluorescent protein]-PTEN) [7,21], similar imaging studies have been used to show that, following a homogeneous cAMP stimulus, much of the pool of PTEN translocates from the membrane to the cytosol within a few seconds and then slowly returns to the membrane [7].

Activation of membrane-recruited PI3K and translocation of PTEN to the cytosol are the two “excitatory” mechanisms that lead to the initial increase in membrane PIP_3_ revealed by PH-GFP relocalization (Figure 1) and qualitative diagrams of chemosensory signaling frequently focus on these excitatory signaling events [17]. Simulations using a model incorporating only these mechanisms would, however, show the concentration of membrane PIP_3_ to increase upon introduction of cAMP without being followed by either the experimentally reported rapid decay in the concentration of this membrane phospholipid or the relocalization of PTEN to the membrane. Therefore, the cells must contain inhibitory components that quench PI3K signaling and also reverse those changes that cause PTEN to dissociate from the membrane, allowing it to return to its pre-stimulus distribution (adaptation). As already noted, a comparison of the dynamic changes in the concentrations of PIP_3_ and membrane-bound PTEN upon exposure of *Dictyostelium* cells to a homogeneous field of cAMP allowed us to conclude that PI3K activity must be rapidly negatively regulated to account for the observed post-stimulus changes in membrane PIP_3_ levels.

**Figure 2:**
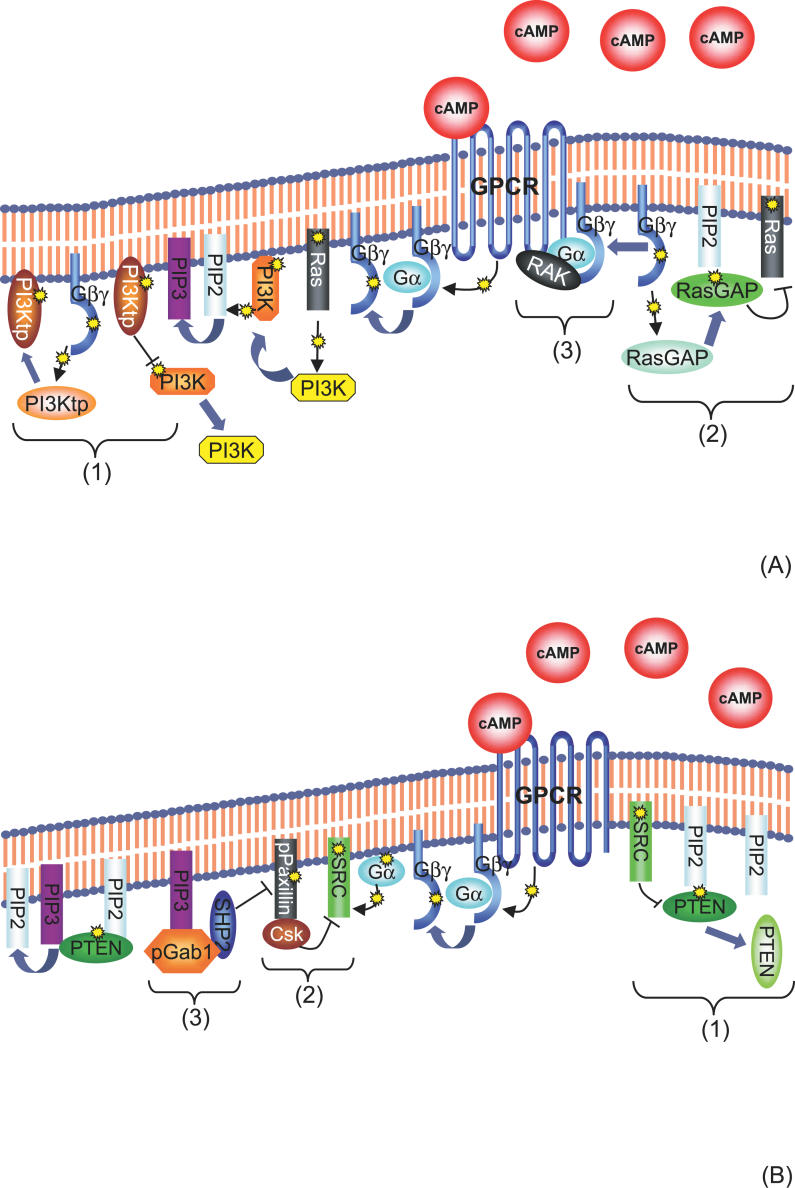
PI3K and PTEN Regulatory Modules (A) Elements controlling the activity of PI3K and upstream components. In addition to the basic, “excitatory,” signaling elements like the cAMP receptor, Gβγ, and Ras, we introduced further elements controlling the activity of PI3K and upstream components. “PI3Ktp” (module 1) stands for a tyrosine phosphatase that deactivates PI3K. This phosphatase becomes enzymatically activated and is recruited to the membrane after interaction with Gβγ. “RasGAP” (module 2) translocates to the membrane and deactivates Ras after activation by Gβγ. RAK blocks Gβγ, Gα, and the receptor, thereby reducing all signals (module 3). (B) Elements controlling the activity and localization of PTEN. In our model, PTEN is phosphorylated by a Src-like kinase, here simply called “Src” (module 1). Src is activated by Gα and deactivated by Csk, which in turn is recruited by phosphoPaxillin (“pPaxillin”) (module 2). SHP2, which is membrane recruited by pGab1 bound to PIP_3_, dephosphorylates pPaxillin (module 3), thereby leading to increased activation of PTEN.

In seeking to account for this regulation of PI3K activity, we took advantage of our previous findings that the α and the βγ subunits of the G proteins remained dissociated as long as chemoattractant was present, establishing that there was ongoing signaling through the excitatory pathways [15]. The inhibitory components thus do not simply shut off receptor signaling but actively compete with ongoing excitatory mechanisms. For the PI3K signaling branch, we therefore included stimulus-induced activation and membrane recruitment of a phosphatase that deactivates PI3K (Figure 2A, module 1) and of a Ras GAP [22] that suppresses Ras activity after an initial spike [16] (Figure 2A, module 2). Additionally, we assumed that the amount of free Gβγ contributing to activation of PI3K is controlled by a blocking element, because the dynamics of the system did not appear to permit re-association with Gα to dampen Gβγ effector function quickly enough (Figure 2A, module 3). Receptor-associated kinase (RAK) was subsequently reported to function in mammalian cells in a manner consistent with this assumption [23].

We also needed to add to the model a set of specific biochemical processes regulating PTEN localization within the cell, in particular a link from activation of the sensing receptor to the dissociation of PTEN from its resting location at the plasma membrane and a negative regulatory limb that reverses this active induction of membrane dislocation. We hypothesized that the molecular change causing PTEN to dissociate from the membrane is a phosphorylation event that in mammalian cells is mediated by a Src-like kinase [24] (Figure 2B, module 1). Reversal of the process that leads to release of PTEN from the plasma membrane thus requires control of the activity of this kinase. We adopted the pathway reported by Ren et al. [25], according to which Src activity is negatively controlled by Csk, which is recruited to the plasma membrane by phosphopaxillin (Figure 2B, module 2). The phosphorylation of paxillin in turn is controlled by the tyrosine phosphatase SHP2, which is brought into proximity of the membrane-bound paxillin through the PIP_3_-binding adaptor Gab1 (Figure 2B, module 3). Figure S1 shows the full network of enzymatic interactions. Figures S2 and S3 focus on those branches of the network that control PI3K and PTEN activity, respectively.

### Simmune Permits Construction of Detailed, Biology-Based Models That Avoid “Black-Box” Abstractions

In order to construct the model signaling network outlined above, with regulatory modules that account for the time-dependent return of the chemosensing apparatus to baseline after a stimulus (adaptation) and that could be tested for whether the model was also adequate to account for intracellular amplification, we incorporated molecules and mechanisms that are well-documented in the literature whenever possible, though in some cases we had to speculate about components and interactions (see also Text S1). Text S2 provides a detailed analysis of the resultant chemosensing signaling network in terms of modules with specific functionalities (“signal transduction module,” “adaptation module,” and “gradient amplification module”) and a discussion of the behavior of the network upon modifications of these modules.

This approach of incorporating molecular detail in regulatory pathways that have been incompletely defined in the experimental literature differs from the more frequently encountered abstract methods of filling these gaps that appear in many theoretical treatments of this topic. Often, limitations in experimental data and also in computational tools have led modelers to utilize conceptual signaling modules (“black boxes”) with the right input-output behavior, to achieve the desired overall behavior of a signaling network. While this approach reduces the effort needed to construct the differential equation equivalent of the signaling network and may provide insights about general dynamic properties of the simulated system, it cannot be used to investigate the behavior of specific molecular mechanisms and frequently fails to incorporate essential regulatory dynamics into the resultant model.

**Figure 3:**
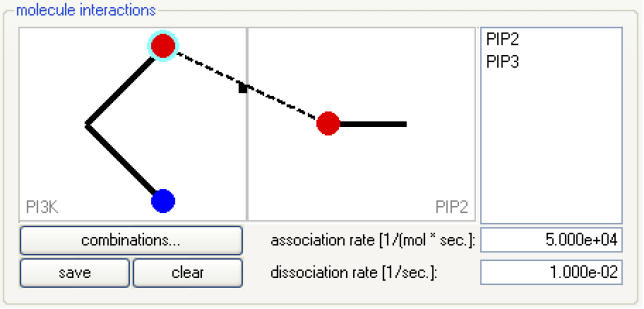
Defining Quantitative Interactions between Molecular Binding Sites Using Simmune (Screenshot) The screenshot of Simmune's modeling interface shows the graphical representations of PI3K and PIP_2_ and the binding sites through which they interact, as well as the sites of membrane attachment. The turquoise circle around the upper binding site of PI3K identifies it as an enzymatically active site. The dotted line represents the possibility of a binding interaction between these two molecules. Selecting the interaction by clicking on the handle on the dotted line allows entry of the relevant binding parameters.

The software suite Simmune was developed to overcome many of the difficulties associated with the creation of detailed, biologically realistic, quantitative models suitable for simulation. It uses the familiar steps of dialog box entry and menu selection to allow the user to readily define molecule types as well as to specify the number and properties of their binding and/or enzymatic sites (Figure 3). This same interface also allows the investigator to define whether a molecule is membrane bound, in which case its movement will be limited to intramembranous diffusion, and to note which part of a transmembrane molecule is in the extracellular environment and which inside the cell, restricting the other molecular species with which these topologically distinct domains can interact during a simulation. Symbolic graphical representations of the defined molecule types appear on the screen and interactions between these entities can be specified in Simmune by simple mouse clicking and dragging to draw connections between the potential binding sites of the reaction partners.

**Figure 4:**
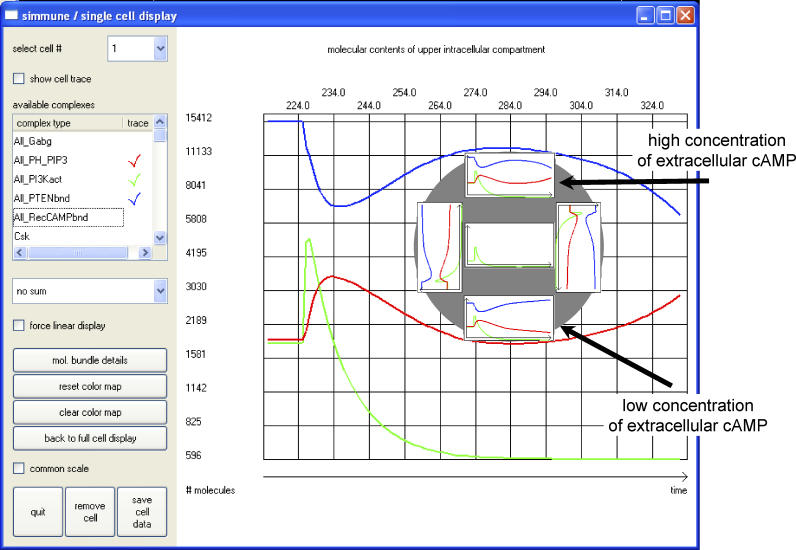
Comparison of the Simulated Activities of PI3K, Membrane-Bound PTEN, and the Resulting Behavior of PIP_3_ (Composite Screenshot) Stimulation of a cell in a 2:1 cAMP gradient (mean concentration 500 nmol) leads to a rapid 3-fold increase in the membrane proximal activity of PI3K (green) and to a loss of membrane-bound PTEN (blue; tracked as GFP-PTEN in real cells). This results in a rapid accumulation of PIP_3_ (red; reported by the location of PH-GFP in real cells). Subsequently, the PI3K activity is strongly quenched by the recruitment of regulatory components to the membrane and falls below its prestimulus level in less than 20 s. PTEN returns more slowly to the membrane. During the phase of downregulation of PI3K activity and reattachment of PTEN to the membrane, the concentration of PIP_3_ decays. In the front of the cell (which experiences a high cAMP concentration), membrane-associated PTEN only returns to a fraction of its prestimulus level and then enters a second phase of decline. After approximately 50 s, the low level of membrane-bound PTEN that is reached in the front of the cell allows PIP_3_ to increase again, even though the amount of active PI3K in this region is modest. In the back of the cell (low cAMP concentration), membrane-bound PTEN increases beyond its prestimulus level, resulting in a decrease of PIP_3_ below its resting state concentration. The circular inset shows a two-dimensional representation of the dynamics of membrane-bound PTEN and PIP_3_ in different regions of the three-dimensional simulation of a cell.

By this simple process of defining binary interactions between the binding sites of reacting species (which closely follows the way biologists traditionally think about signaling networks), the user provides Simmune with the information needed to determine which molecular complexes are possible in the system, for example, a complex consisting of the cAMP receptor and an associated Gαβγ heterotrimer. The program then automatically constructs the complete network of complexes from the component definition input, collects the reactions in which each complex participates, and calculates the contributions of these reactions to the rate of change of the concentration of each complex (which depends on the reaction rates and the—typically changing—concentrations of the reaction partners). Given a set of initial conditions, Simmune can then calculate the time course of changes in the concentrations of all molecular complexes in the model after a stimulus is applied. The software also permits the investigator to both visualize and obtain a dynamic quantitative readout of the subcellular location (Figure 4) and binding states of all the molecular complexes and of the flux through the network during the simulated signaling process (Figure S1, Video S1).

### Simulation Predictions versus Experimental Findings

Computational models are best evaluated by examining their ability to make correct predictions how a biological system will behave under conditions for which experimental data are not yet available and that were not used to establish the model's core parameters. In the present case, this meant using responses to homogeneous chemoattractant fields to establish parameters that were then used by Simmune to simulate the response of *Dictyostelium* not only to other (untested) homogeneous concentrations of cAMP but, more important, to gradients of cAMP that were not used for creating the model.

To measure quantitatively the behavior of PIP_3_ and PTEN during the chemosensing response of *Dictyostelium,* experiments were performed by single-cell microscopy of cells in a one-well chamber reacting to microinjector-delivered cAMP stimulation (see [15] for details). We simulated these experiments using Simmune by placing simulated cells with a given initial composition of signaling molecules into three-dimensional extracellular compartments (representing the culture well). The model was then run for a period of time to allow the simulated cells to achieve a biochemical steady-state in the absence of cAMP stimulation The fluctuations in shape and PIP_3_ distribution seen even in the absence of cAMP exposure using amoeboid cells with actin-based morphological prepolarization [26] were avoided in the present study by using lactrunculin to inhibit actin polymerization, thus allowing the result of this computational equilibration process to reflect the biological situation before stimulation with cAMP. After equilibration the simulated cells were exposed to in silico cAMP gradients equivalent to the actual chemoattractant conditions experienced by the real cells under experimental conditions. Simmune then calculated the spatiotemporal response of the concentrations of components like PIP_3_, activated PI3K, and membrane-bound or cytosolic PTEN. The software furthermore allowed us to save the time-dependent behavior of molecular concentrations and flows through the signaling pathways of the simulated cell to data files for visualization using automatically generated diagrams of the cell's reaction network (Figure S1, Video S1).

First, we examined in this manner the responses of simulated cells to varying concentrations of homogeneously applied cAMP. In these simulations, the time T_max_ post-cAMP exposure at which PIP_3_ reached its peak decreased with increasing concentration of the applied chemoattractant. After having adjusted the model parameters in such a way that T_max_ for one concentration of cAMP (25 nmol) in the simulation matched the experimentally observed value, simulations quantitatively predicted the correct concentration dependence of T_max_ for 10-fold higher and 10-fold lower concentrations of cAMP in latrunculin-treated (morphologically nonpolarized) *Dictyostelium* cells expressing the PIP_3_-binding chimeric molecule PH_crac_-GFP (Figure S4; [1,18,19]). This established that the model was able to accurately predict cellular behavior under conditions not used to establish the specific parameter values employed for the simulations.

We next examined the model's performance when used to simulate a cell's more complex response following exposure to extracellular cAMP gradients. The simulated cells showed the previously reported transient PIP_3_ elevation along their entire perimeter [6,20], which then completely decayed everywhere except for the “front” side of the cell that was exposed to the highest concentration of cAMP. Also, simulated PTEN translocated from the cytoplasmic membrane to the cytosol and reassociated with the membrane in the posterior of the cell as expected [7]. However, the PIP_3_ response at the “front” side of the cell showed an unanticipated behavior in these simulations: after an initial peak, it decayed transiently and then recovered to go through a second phase of increase, followed by a second, typically higher, peak. Previous experimental studies had not reported this type of biphasic behavior of PIP_3_ at the front of a cell [6], so we sought to determine whether the results indicated a flaw in the model or whether the model parameters were “incorrect” and could be adjusted to make the dip in between the two peaks shallower, yet preserve the cells' quantitatively correct behavior upon exposure to homogeneous stimuli. Although for gradients with low (less than 10% of the *K*_d_ for the binding of cAMP to the receptor) absolute cAMP concentrations we could find a parameter set yielding a monotonic response, this was not possible to achieve with any reasonable parameter set when the concentration of cAMP was increased.

We then examined in detail the time-dependent changes in PIP_3_, active PI3K, and membrane-bound as well as phosphorylated cytosolic PTEN in the different regions of the cell during a simulated gradient response, using a reasonable parameter set consistent with the accurate simulation of homogeneous field behavior. This analysis indicated that the biphasic PIP_3_ kinetics arose from a difference between the speed of signal-induced negative regulation of PI3K activity and that of PTEN membrane reattachment (Figure 4). In accordance with previously published data [27], the simulation showed PI3K activity to be downregulated very rapidly (within several seconds) after the onset of stimulation (local adaptation), while the translocation phase of PTEN took considerably longer, typically 1 min [7]. Because the level of PIP_3_ is determined by the balance between local PI3K activity and local availability of membrane-bound PTEN, the simulated cell's front experienced two phases of PIP_3_ accumulation. During the first phase PI3K is highly activated and PTEN has just begun its dissociation from the membrane. This phase ends with the rapid decrease of PI3K activity due to the operation of the regulatory pathways noted above and a partial return of PTEN to the membrane. During the second phase, the imbalance in Gα-induced Src-like kinase activity between the front and the back of the cell leads to a gradual loss of membrane-bound PTEN in the front and accumulation of PTEN in the back. With a strongly decreased level of PTEN at the front of the cell, the residual activity of PI3K now can induce a second, slower increase of PIP_3_ at the cell's anterior. Figure S5 illustrates this process that translates (even small) differences in receptor occupancy between front and back of the cell into steeper intracellular gradients of PIP_3_ and PTEN (amplification).

**Figure 5:**
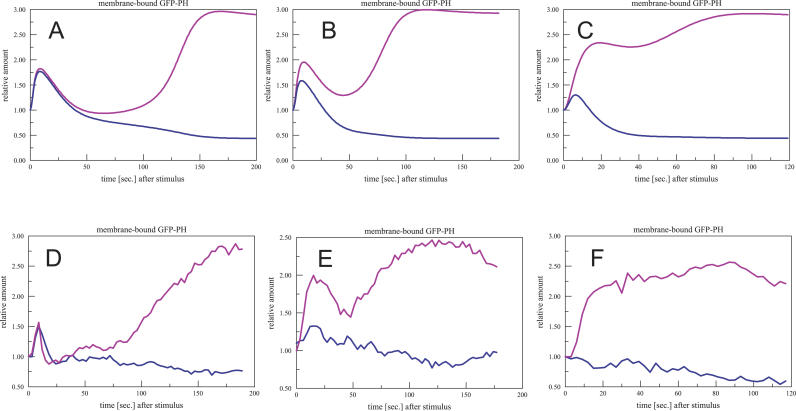
Correspondence in Time and Space between the Predicted and Measured Changes in PIP_3_ at the Front and Back of Cells Exposed cAMP Gradients Experimental data from exposure of *Dictyostelium* to a 2:1 gradient with a mean cAMP concentration of 100 nmol were used to adjust model parameters. The other two responses are predictions of the model. (A), (B), and (C) are simulated responses. (D), (E), and (F) are experimental measurements, using PH-GFP to monitor PIP_3_ levels in single cells exposed to gradients with a mean cAMP concentration of 1 μmol, 100 nmol, and 10 nmol. See Figure S6 for details on the full dataset of experimental replicates.

Following the development of new experimental techniques for rapid exposure of *Dictyostelium* cells to a defined cAMP gradient [15], we found that the predicted biphasic PIP_3_ response at the front of the cell corresponded to the actual behavior of the gradient sensing system, as did the concentration dependence of the extent of the decline in PIP_3_ levels after the initial peak (Figure 5). Furthermore, the simulation predicted that the second increase would happen later and take longer to reach its peak if the absolute concentration of the chemoattractant was increased while maintaining the relative concentration difference between front and back of the cell. We also found this prediction to be correct (Figure 5). Adjusting the model parameters so that the dip between the first and second peak of PIP_3_ corresponded quantitatively to the experimentally determined time for a single chemoattractant concentration, the simulation correctly predicted the altered slope for the second increase and for the position in time of the second PIP_3_ peak when using 10-fold higher or lower absolute concentrations of cAMP (Figure 5). The model was thus able to predict with great accuracy in both time and space the PIP_3_ response of *Dictyostelium* cells over a several-log range of chemoattractant concentration.

**Figure 6:**
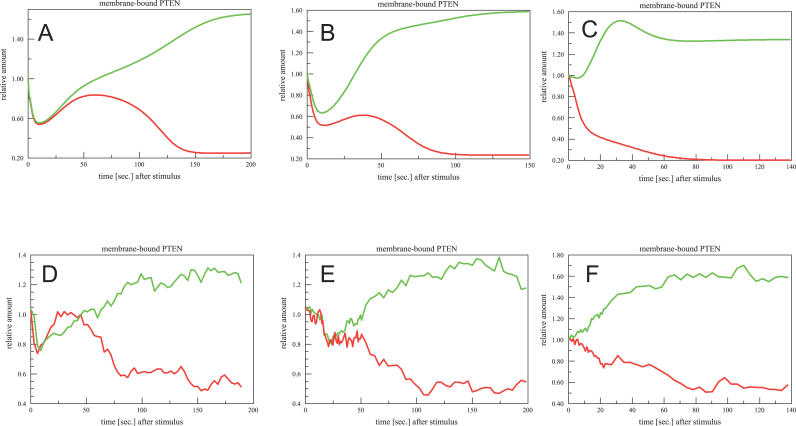
Correspondence in Time and Space between the Predicted and Measured Changes in Membrane-Bound PTEN at the Front and Back of the Cells Exposed to cAMP Gradients These dose-dependent dynamics of PTEN were produced by the simulation after the dynamics of PIP_3_ for one cAMP concentration (100 nM) had been used to adjust model parameters. (A), (B), and (C) are simulated responses. (D), (E), and (F) are experimental measurements based on GFP-PTEN analysis in single cells exposed to gradients with a mean cAMP concentration of 1 μmol, 100 nmol, and 10 nmol. See Figure S7 for details on the full dataset of experimental replicates.

To test further the predictive power of the model, we used cells expressing GFP-PTEN to examine whether the simulated spatiotemporal properties of PTEN relocation corresponded to those measured experimentally. At the cell's front, GFP-PTEN showed the triphasic behavior we expected, involving a decline, a temporary increase, and then a substantial decay. The local peak of PTEN occurred at the predicted time point and the ensuing decay correlated well with the second increase of PH_crac_-GFP at the front of an adjacent cell exposed to the same gradient. As predicted, with progressively lower concentrations of cAMP, the temporary increase of PTEN seen with a high-concentration stimulus first turned into a shallow plateau and then vanished (Figures 6, S6, and S7; see also Text S3).

## Discussion

Here we describe the construction and evaluation of a new detailed model of the chemotactic signaling response of *Dictyostelium*. We tested the model's explanatory and predictive capabilities in a close interplay between computer simulations and laboratory analysis using quantitative single-cell microscopy. The simulations correctly predicted the biochemical behavior of the cells under conditions that had never before been experimentally investigated in detail and led us to propose specific substantial changes to the existing model of chemosensory signaling in this organism, modifications that are needed to reconcile theory with experimental observations.

The model and the simulations reported here illustrate how signal-induced activation and membrane-recruitment of components controlling the availability and/or activity of Gβγ, Ras, PI3K, and PTEN can induce rapid adaptation of the level of PIP_3_ and of the localization of PTEN following homogeneous stimuli with chemoattractant. Interestingly, these same mechanisms lead to a polarized distribution of PIP_3_ in gradients of cAMP. In contrast to the predominant view in the field, our studies suggest that the intracellular amplification of the applied chemotactic gradient is the consequence of the local, rather than global, feedback-regulation of excitation and adaptation. Upon stimulation of the cell, the product (PIP_3_) of the activation of an excitatory component (PI3K) reinforces the processes leading to deactivation of an inhibitory component (PTEN), thereby allowing for a rapid, pronounced response. Following this first response, adaptation is achieved through a strong, locally controlled and signal-dependent suppression of the excitatory component which, in turn, decreases the deactivation of the inhibitory component, i.e., allows for reactivation of the inhibition. In a gradient, however, the higher concentration of PIP_3_ at the side of the cell facing the higher chemoattractant concentration (“front”) supports the activation of more PTEN-deactivating components than at the opposite side (“back”) of the cell. This leads to a net transfer of PTEN from the front to the back, which in turn decreases the concentration of PIP_3_ in the back, accelerating that side's transition into the adaptation phase. In our model, polarization of a cell in a chemoattractant gradient is thus caused by the (diffusive) communication between the sides of the cell with high or low chemoattractant concentration, respectively, interfering with the local adaptation processes.

The recruitment of Ras GAP through PIP_3_ [22] and the multiple functions of receptor-associated kinase, which in addition to phosphorylating the receptor binds to Gα and blocks Gβγ from acting on Ras and PI3K [23], are well-documented negative feedback mechanisms in G protein–coupled receptor signal transduction. Our model suggests the action of two additional regulatory components: a protein phosphatase that deactivates PI3K and a lipid phosphatase (SHIP [SH2-containing inositol 5′-phosphatase]) that is recruited to the front of the cell to control the level of PIP_3_ when the local concentration of active PTEN is low. These elements act on two different time scales. The phosphatase that deactivates PI3K is rapidly activated to control PIP_3_ levels during early adaptation of the network. SHIP is more slowly recruited to those regions of the membrane that have lost PTEN. An abstract slow second inhibitory (“poisoning”) element had been previously suggested by Meinhardt [12] to prevent the cells from losing their sensitivity to gradient changes and we can speculate that this hypothetical element corresponds to SHIP in our more specific scheme. Biochemical studies are now required to see if the various components we have incorporated into our extended biochemical model from mammalian studies have molecular analogs in *Dictyostelium* with the anticipated functions.

As noted before (and discussed in detail in Text S1), we think that it is helpful to use modeling to speculate about the role of the different components in a signaling network not in terms of abstract modules but in terms of interacting molecules. Instead of postulating, e.g., a “PTEN-translocation-control-module,” we asked which molecules have been observed to play a role in the control of the membrane attachment of PTEN and how they exert this control. Simmune in particular allows the facile construction and simulation of such biochemically specific models because components represent concrete signaling molecules. This allows simulated responses to be analyzed in molecular detail and the predicted behavior of these molecules to be directly compared to results obtained by traditional laboratory methods such as immunoprecipitation, immunoblotting, flow cytometry, and confocal microscopy. As we discuss in Text S2, previous, more abstract models with PTEN-control modules neither suggested concrete molecular equivalents of those modules nor were they able to generate the correct behavior of PTEN or PIP_3_ in response to chemoreceptor engagement.

Not all aspects of *Dictyostelium* chemotactic behavior are fully captured in even the extended model we have implemented here. While the simulations arising from the present model were quite accurate in their spatiotemporal details under diverse conditions, it is very likely that details of the regulatory mechanisms included in the present model are incorrect or that important additional components are absent. An indication that this is the case comes from observations made while experimenting with models containing alternative pathway configurations or very different parameter sets (see Text S2). In certain situations, residual PI3K inhibitors persisted at the former high-concentration side of the cell membrane once the stimulating gradient was removed. In these simulations, the persistence of these inhibitors then led to a temporarily inverted biochemical response during secondary exposure to a chemotactic gradient, a behavior that has recently been observed experimentally (XX, MMS, and TJ, manuscript in preparation). The specific model we describe here does not permit a single parameter set to give a simulation output that reproduces in detail this inversion behavior, while also being in full quantitative agreement with the primary polarization kinetics reported in this paper across a wide range of cAMP concentrations. These recent findings suggest that additional elements will need to be added to the circuits controlling local PIP_3_ levels to generate a more robust molecular model.

This issue of persistence of inhibitory components at the leading edge after cAMP removal is closely related to the key question of what mechanisms keep a cell responsive to changes in the externally applied gradient. *Dictyostelium* cells that have fully differentiated into the chemotactically competent stage behave like neutrophils and turn their “body” instead of changing the orientation of their internal biochemical polarization when the extracellular gradient changes direction. During earlier differentiation stages, however, *Dictyostelium* reacts to such changes by reorienting its internal PIP_3_-dependent polarity (C. Parent, personal communication). Cells simulated with our current model react to changes in the direction of externally applied cAMP gradient in a manner similar to these immature amoeba, with a reorientation of the internal PIP_3_ gradient. However, the simulated reorientation is only partial and occurs more slowly than that observed directly in live cells (XX, unpublished data), again pointing to the need for a reworking of the mechanisms that control the activity of PI3K at the leading edge of a cell exposed to a gradient.

Many experimental biologists see no immediate need for measuring in a highly quantitative manner either intracellular molecular concentrations, their stimulus-induced changes, or the corresponding reaction rates. One factor contributing to this viewpoint is the limited availability of user-friendly computational tools that would allow investigators to make direct use of such data for detailed simulations that could enhance their biological understanding. Simmune, the software we developed and used here to investigate the mechanisms behind *Dictyostelium*'s chemosensing capabilities, enables biologists lacking advanced mathematical or computer coding expertise to conduct the type of simulations that make the pursuit of quantitative experimental data a more useful undertaking. The software provides a simple interface for model building and parameter entry, provisions for automated construction of complex molecular interaction networks from the input of binary molecular interactions, visual output of the spatial distribution and number of specific molecules/molecular complexes in model cells during a simulation, and the capacity to view at varying levels of resolution the time-dependent changes in component concentrations or higher-order molecular complexes within a signaling pathway. It also permits creating models across varying scales of biological resolution, from intracellular molecular networks to individual cell behavior to the activity of groups of simulated cells.

Quantitative modeling is just beginning its foray into the cell biology of intracellular signaling, and measurements of the temporal behavior of the concentrations of most of the relevant molecules are still missing. We hope that Simmune will help encourage investigators in diverse fields of biological research to fill these gaps by providing them with an easily mastered yet powerful tool for translating biological knowledge and data into models and simulations that can enhance understanding of a complex biological system, as we show here for chemosensing in *Dictyostelium*.

## Materials and Methods

### Cell lines, single-cell microscopy.

Single-cell imaging of *Dictyostelium* for analysis of the activation of trimeric G proteins, relocation of PH-GFP in response to changing PIP_3_ levels, and chemoattractant concentrations at the membrane was performed as described in [15]. For the experiments with GFP-PTEN, cells from the D. discoideum cell line expressing GFP-PTEN [21] were developed into the chemotactic stage, then exposed to cAMP and imaged as described in [15]. The cells were treated with latrunculin prior to the experiments to suppress actin polymerization. This treatment immobilizes the cells, thereby facilitating the quantitative analysis of changes in the local concentration of PH-GFP. Furthermore, it eliminates any morphological prepolarization of the cells that otherwise might cause different sensitivities toward chemotactic stimulation at their different sides, and, finally, it decouples actin dynamics from the purely biochemical network we wished to study.

### Simulation software.

We used the “Simmune” modeling and simulation software, as described in the text and supplementary material. Simmune runs on Linux, MacOS 10, and Windows XP. Copies of Simmune (executables) can be requested by sending an e-mail to software@simmune.org. Updated documentation and information about new releases and bug fixes are available through http://www.simmune.org.

## Supporting Information

Figure S1 Automatically Constructed Visualization of the Enzymatic Reaction Network of the Chemotactic Signaling Model These screenshots of the network browser show the enzymatic reaction network before (A) and after (B) stimulation with cAMP. (B) Shows the response at the “front” (high cAMP concentration) of the cell. Simmune uses the binary interaction possibilities defined by the user to automatically construct the permitted network topology and to generate a graphical view of the network with several user defined outputs available for the display (binding interactions, enzymatic reactions, and so on). Concentrations of enzymes and their (single-) molecular substrates are represented in the color saturation of the circles and ellipsoids representing the network components. The concentration of each component is displayed relative to its maximum concentration during a simulated experiment (darker = greater concentration). Similarly, the (relative) reaction flow for enzymatic transformations is encoded in the saturation level of the blue lines representing the biochemical transformations. A line with a circle represents the action of an enzyme on its substrate. A line with an arrow indicates the transformation of the substrate into the product of the reaction. Video S1 shows how concentrations and flows in the front of a cell (high cAMP concentration) change during the course of a stimulation of a model cell exposed to a gradient of cAMP. Because concentrations and reaction flows are encoded in the color saturation of the component symbols and their connecting lines, some lines are almost invisible in the snapshots of the network dynamics presented here.
(101 KB PDF)

Figure S2 The Branch of the Signaling Network That Controls the Activity of PI3K A line with a circle represents the action of an enzyme on its substrate. A line with an arrow indicates the transformation of the substrate into the product of the reaction. The green lines represent those passive binding possibilities that play important roles in regulation of PI3K activity.
Gβγ is assumed to recruit PI3K to the membrane where it then can interact with Ras and become fully activated [7]. Ras itself becomes activated through a GEF that in turn also depends on Gβγ for its activation [37]. In our model, we adopt this activation pathway for PI3K but allow for a direct activation of Ras through Gβγ. In addition, Gβγ activates RasGAP and the tyrosine phosphatase “PI3Ktp”. Active Ras (“Ras_act”) activates PI3K. Activated RasGAP (“RasGAPopen”) can attach itself to PIP_2_ and PIP_3_, which places it in close proximity of its membrane-bound substrate, Ras_act. The activated phosphatase PI3Ktp_act deactivates PI3K. It can attach itself to the phosphorylated membrane-bound adaptor pPI3Ktp_anch (not shown) to localize near membrane-associated, active PI3Kact. The receptor-mediated stimulus leads to a local higher concentration of inhibitors of PI3K activity. Note: Unactivated enzymes have a (low) basal affinity for/rate of action on their substrates. Accordingly, even the unactivated enzyme states are linked to their substrates by lines with dots.
(31 KB PDF)

Figure S3 The Branch of the Signaling Network That Controls the Activity of PTEN A line with a circle represents the action of an enzyme on its substrate. A line with an arrow indicates the transformation of the substrate into the product of the reaction. The green lines represent those passive binding possibilities that play important roles for the regulation of PTEN activity.
PTEN is an interfacial enzyme, having a significantly higher phosphatase activity when positioned in close proximity to its membrane-embedded substrates like PIP_3_ [38]. Mammalian PTEN contains two domains that contribute to recruitment of the phosphatase to the membrane [39]. Both domains can lose their membrane-binding ability upon phosphorylation of the phosphatase at two specific sites in the C-terminus [40]. A recent report on the role and mechanism of membrane recruitment of *Dictyostelium* PTEN has shown that in this organism, the N-terminal region possesses a PIP_2_ binding motif that is crucial for the membrane recruitment of PTEN and its physiological activity [41]. Because PTEN leaves the membrane of *Dictyostelium* after stimulation of the cells with chemoattractant and because the relatively high membrane abundance of PIP_2_ is not likely to be substantially decreased upon chemotactic stimulation, we assume in our model that the chemotactic signal leads to phosphorylation of PTEN that interferes with its membrane binding capabilities and its phosphatase activity. In mammalian cells, PTEN phosphorylation has been shown to be mediated by Src [24], so we assume in our signaling network a similar role for a Src-like kinase that in turn is stimulated via active Gβγ. Activation of Gα leads to activation of “SrcAct,” the component that activates Src. Active Src (“Src_act”) phosphorylates PTEN. Phosphorylated PTEN (“pPTEN,” or membrane-bound: “pPTEN_bnd”) has lost its phospholipid phosphatase activity and rapidly dissociates from the membrane. Csk deactivates Src_act. Csk is brought into close proximity with membrane-bound Src through its capability to bind to phosphorylated paxillin (“pPaxillin”). SHP2 dephosphorylates pPaxillin, thereby reducing the amount of membrane-proximal Csk. SHP2 is recruited to the membrane by binding to Src-phosphorylated Gab1 (“pGab1”) [42], which in turn binds to PIP_3_. The PIP_3_-dependent recruitment of SHP2 and SrcAct thus represents a positive feedback for the processes leading to phosphorylation (and deactivation) of PTEN; the more PTEN becomes deactivated, the more PIP_3_ that can be produced by PI3K. Increasing concentrations of PIP_3_ lead to recruitment of more SHP2 and SrcAct and result in faster deactivation of PTEN. This explains (in our model) the efficiency of the stimulus-induced translocation of PTEN from the cell's front to its back.
(49 KB PDF)

Figure S4 Predicted and Measured Dose Dependence of the Time T_max_ until the PIP_3_ Accumulation Reaches Its Maximum after Homogeneous Stimulation with cAMP With increasing concentrations of homogeneously applied cAMP, the cells adapt more rapidly to the stimulus.
(10 KB PDF)

Figure S5 Local Adaptation Processes, When Combined with Diffusive Communication between the Different Sides of the Cell, Can Explain the Intracellular Amplification of the External cAMP Gradient The primary signal (ligation of the receptor, activation of the G proteins) is shown in light blue. Excitatory processes are shown in orange, inhibitory processes in gray. The processes that are part of the feedback mechanism establishing the intracellular polarization are shown in yellow.
(81 KB PDF)

Figure S6 Global Dataset of Changes in Membrane-Bound PH-GFP in Cells Exposed to 2:1 Gradients of cAMP Mean cAMP concentrations at the cell surface: (A) 1 μmol (15 datasets); (B) 100 nmol (18 datasets); (C) 10 nmol (25 datasets).
Due to the considerable variation in the responses of single cells, the average behavior of membrane-bound PH-GFP shows less pronounced “dips” in the cell front after the first peak than single-cell responses. For the comparison with simulation results, we chose single-cell responses that have their characteristic features (minima and maxima) at approximately the same time points as the multi-cell averages.
(25 KB PDF)

Figure S7 Global Dataset of Changes in Membrane-Bound GFP-PTEN in Cells Exposed to 2:1 Gradients of cAMP Mean cAMP concentrations at cell surface: (A) 1 μmol (four datasets); (B) 100 nmol (12 datasets); (C) 10 nmol (six datasets).
Due to the considerable variation in the responses of single cells, the average behavior of membrane-bound GFP-PTEN shows less pronounced minima in the front of the cell than single-cell responses. For the comparison with simulation results, we chose single-cell responses that have their characteristic features (minima and maxima) at approximately the same time points as the multicell averages.
(25 KB PDF)

Figure S8 The Influence of Parameter Variation on the Signaling Dynamics This screenshot of Simmune shows the results of simulated exposure to a 2:1 cAMP gradient (100 nmol mean concentration) with an automated variation of the parameters determining the number of Csk molecules per cell and the association rate for the activation by Gβγ of the phosphatase (PTENph) that dephosphorylates pPTEN. Csk varies (vertical axis) from 80,000 (upper row) to 160,000 (lower row) molecules per cell. The PTENph-Gβγ association rate varies (horizontal axis) from 50,000 1/(mol*s) (left column) to 500,000 1/(mol*s) (right column). While for all parameter sets the overall behaviors of membrane-bound GFP-PH and PTEN show the characteristic minima and maxima, their positions in terms of time points and molecule amounts change significantly with varying parameters. While the qualitative features are thus “robust” to the examined range of parameter values, the quantitative details are sensitive to these numerical changes. With parameter set (1), for example, the second peak of the accumulation of GFP-PH at the high-concentration side of the applied cAMP gradient (red) does not reach the experimentally observed value. On the other hand, the increase in PTEN at the low-concentration side of the cell (blue) for this set of parameters is too strong. Set (3), with a high PTENph-Gβγ association rate and a high concentration of Csk, reaches the second peak in the accumulation of GFP-PH at the side with high cAMP concentration too late. The dynamics obtained with set (2) (which is the parameter set chosen for all simulations reported here) correspond well to the experimentally observed behavior of membrane-bound GFP-PH and PTEN.
A comparison of the simulated behavior of membrane-bound PTEN after homogeneous stimulation with 100 nmol cAMP: the curves (1), (2), and (3) were obtained with the same parameter sets as the correspondingly labeled simulation results in (A). Set (2), which shows the correct behavior under homogeneous stimulation, also produces quantitatively and qualitatively correct results for stimulation with a cAMP gradient [see (A)]. See also Video 7 or Text S13.
(138 KB PDF)

Figure S9 Modifications of PI3K Membrane Attachment Result in Changed Behavior of PTEN Stimulation with a homogeneous concentration of 100 nmol of cAMP leads to a transient loss of membrane-bound PTEN. For this dose, there is an 80% recovery of membrane-bound PTEN within 40 s. The dashed curve shows the simulated behavior of membrane-bound PTEN for a cell with membrane attachment of not only activated but also nonactivated PI3K with basal enzymatic activity. In agreement with previously reported data [7], PTEN shows a slower, only partial return to the membrane.
(11 KB PDF)

Figure S10 Behavior of PTEN with an Alternative Mechanism Regulating PTEN Membrane Attachment See Text S2, Section 3 for details.
(A) Loss and recovery of membrane-bound PTEN after stimulation with a homogeneous concentration of 400 nmol (solid line) and 40 nmol (dashed line). For both concentrations, PTEN shows a qualitatively correct behavior.
(B) Translocation of PTEN after stimulation with a linear gradient of 40 nmol in the “front” and 10 nmol in the “back” of the cell. The applied gradient leads to a pronounced accumulation of PTEN in the back and loss of PTEN in the front.
(C) Translocation of PTEN after stimulation with a linear gradient of 400 nmol in the “front” and 100 nmol in the “back” of the cell. The mean concentration of the applied gradient is closer to the saturation dose of the cAMP receptor. The receptor signal strengths in front and back are not strongly different. Due to the lack of an amplifying element, the applied gradient fails to induce a strong polarization of membrane-bound PTEN across the cell diameter.
(22 KB PDF)

Figure S11 Mechanisms of Cellular Behavior in Simmune (Composite Screenshot) Cells created in Simmune contain specified numbers of molecules and molecular complexes in their cytosol and on their membrane, based on the definition of their biochemistry by the modeler. The molecules will then diffuse and react according to their diffusion coefficients and reaction rates. Such intracellular reaction-diffusion networks determine the low-level behavior of the cells. Simmune offers a variety of possibilities for inspection of the intracellular biochemistry of simulated cells (see Figures 4 and S1). The higher-level behavior of cells in Simmune is defined by the ***stimulus-response mechanisms*** they possess (see Text S4). In the example illustrated here, the stimulus consists of the ligation of the receptor for the green molecules. A second condition used in the simulation is the absence of a suprathreshold amount of ligated receptors for the red molecules. If both conditions are fulfilled, the cell secretes the blue molecules. In the simulation screenshot which shows a two-dimensional cut through a three-dimensional extracellular compartment, it can be seen that only cells within regions of high concentration of the green molecules and relatively low concentrations of the red molecules secrete the blue molecules. The cells in the region indicated by the white circle sense a sufficiently high concentration of green molecules but also a high concentration of red molecules. They do not respond.
(240 KB PDF)

Figure S12 The “Complex Definition” Window of Simmune (Composite Screenshot) Molecule complexes consist of molecules that are connected through interactions between their binding sites. During a simulation, the program creates representations for all possible molecule complexes (to an upper limit in the number of molecular components that can be set by the user), based on the binding interactions defined for the molecules in the model. The modeler does not have to define each complex by hand.
Wherever the local biochemistry allows the formation of a complex in some part of the simulated system, this complex will be taken into account for the simulation of the molecular reactions. Importantly, the set of (partial) differential equations that are processed during a simulation only includes equations for such complexes that are actually part of the local biochemistry. For example, the differential equations describing the reactions and the diffusional exchange of molecules for a cytosolic region of the cell never include components that describe the reaction dynamics of plasma membrane receptors. This minimizes computational cost.
Specific molecule complexes can be created for use in the definition of cellular mechanisms (see Text S4) or for the definition of enzymatic transformations (see Simmune Tutorial 3 in Video S4, or Text S11). If the local concentrations of particular molecule complexes are to be tracked during a simulation, these complexes have to be defined first.
The structures in the left list of defined molecules within the “complexes” window can be used as building blocks to create molecular complexes. Molecule names from the list can be dragged and dropped into the white area of the “complex definition” part of the window. The light gray squares in the area indicate different possible positions of the molecules within a complex. Depending on the properties of the molecule (whether it is a receptor or cytosolic molecule) and the presence of other molecules that have already been put into the complex definition field, some positions within the field are possible/allowed while others are not. Simmune indicates the possible positions by turning the allowed squares green while a molecule is being dragged into the complex definition area. Once a molecule has been dropped into an allowed square, the program will indicate all possible binding interactions with the other molecules within the “complex definition” field. Double-clicking the small gray square attached to the potential connection between two molecular binding sites establishes the connection. The binding sites involved in the connection will snap together (see inset). Once all the connections for the complex the modeler wishes to build have been defined, a name can be specified and the complex saved. If the molecules are not properly connected or if the complex is isomorphic to an already existing complex, Simmune will issue an error message.
(55 KB PDF)

Figure S13 Comparison of Simmune's Diffusion with Analytical Solution See Text S7 for details.
(55 KB PDF)

Table S1 Model Parameters (48 KB XLS)

Text S1 Rationale for Model Building (21 KB PDF)

Text S2 Modular Analysis of the Chemosensing Signaling Network (32 KB PDF)

Text S3 Note on the Determination of Parameter Values for the Chemosensing Model (16 KB PDF)

Text S4 From Molecular Interactions to Cellular Behavior—How Simmune Works (32 KB PDF)

Text S5 Simmune's Internal Representation of Molecular Complexes (13 KB PDF)

Text S6 Automatic Creation of the Reaction Network (23 KB PDF)

Text S7 Discretization of Extracellular and Intracellular Space in Simmune (35 KB PDF)

Text S8 Simmune Tutorial Part 1 Defining molecules, binding and debinding events, and allosteric molecular modifications.
(310 KB PDF)

Text S9 Simmune Tutorial Part 2 Defining transmembrane receptors and transmembrane signaling events.
(217 KB PDF)

Text S10 Simmune Tutorial Part 3 Defining specific molecular complexes and enzymatic transformations.
(733 KB PDF)

Text S11 Simmune Tutorial Part 4 Defining cells and extracellular space; running simulations.
(1.7 MB PDF)

Text S12 Simmune Tutorial Part 5 Simulating the Dictyostelium chemosensing model.
(513 KB PDF)

Text S13 Simmune Tutorial Part 6 Automated parameter variation for the chemosensing model.
(1.0 MB PDF)

Text S14 Simmune Tutorial Part 7 Building and simulating a simple model of cellular gradient sensing.
(2.3 MB PDF)

Video S1 Simmune's Display of Signaling Dynamics within the Reaction Network (3.8 MB MOV)

Video S2 Simmune Tutorial Part 1 Defining molecules, binding and debinding events, and allosteric molecular modifications.
(2.8 MB MOV)

Video S3 Simmune Tutorial Part 2 Defining transmembrane receptors and transmembrane signaling events.
(2.3 MB MOV)

Video S4 Simmune Tutorial Part 3 Defining specific molecular complexes and enzymatic transformations.
(5.8 MB MOV)

Video S5 Simmune Tutorial Part 4 Defining cells and extracellular space; running simulations.
(6.8 MB MOV)

Video S6 Simulating the Dictyostelium Chemosensing Model (Tutorial Part 5). (6.9 MB MOV)

Video S7 Automated Parameter Variation for the Chemosensing Model (Tutorial Part 6). (6.9 MB MOV)

## Abbreviations

cAMP: cyclic adenosine monophosphate
GFP: green fluorescent protein
PH: pleckstrin-homology
PI3K: phosphoinositide 3-kinase
PIP_3_: phosphatidylinositol-(3,4,5)P_3_
PIP_2_: phosphatidylinositol-(4,5)P_2_
PTEN: phosphatase and tensin homolog deleted on Chromosome 10
RAK: receptor-associated kinase
SHIP: SH2-containing inositol 5'-phosphatase

## Notes

Obstacles and Solutions to Accessible, Biology-Faithful, Quantitative Computer Modeling and Simulation Detailed modeling and simulation of complex biological systems remains difficult because of both their inherent complexity and the limitations of current tools available for assisting in such research. Even in a single cell, signaling processes typically involve events on several hierarchical spatial and organizational scales. Following a membrane receptor stimulus, modifications of molecular interaction domains change association and dissociation rates for receptor-proximal bimolecular interactions. These changes, in turn, increase or decrease the availability of membrane-proximal docking sites and/or enzymatic activities. The consequences on the next higher spatial scale may be formation of macromolecular signalosomes or redistribution of molecules among membrane subdomains or between intracellular compartments [28]. Additional complexity is introduced when considering the behavior of an entire cell and of groups of cells that interact with each other, or the physiology of complex tissues with both cellular and stromal elements that constrain molecular diffusion or limit cell migration. In many modeling efforts, modelers attempt to reduce this complexity by simplifying the system using assumptions that biological experts would not accept as yielding a faithful representation of known biology. Furthermore, most modeling tools for cellular signaling currently focus on the “biochemistry scale,” translating signaling maps into sets of ordinary differential equations that describe the dynamics of changing concentrations of molecular complexes based on mass-action relationships [29–31]. However, reducing a biological model into a mass-action network “by hand” runs the risk that multicomponent complexes critical to the operation of the pathway in question may be ignored for the sake of computational simplicity or inadvertently omitted. Moreover, such translation removes molecular details usually considered important by most biologists, in particular, information about the specific binding sites responsible for the formation of molecular complexes or for membrane recruitment of signaling components. New methods that are more automated and that adhere more closely to the underlying biology/biochemistry would be a major boon in this area of investigation. If possible, such tools should also free the user from having to learn specialized coding rules to construct a model and conduct simulations, so that the largest number of investigators can employ these quantitative methods. Over the past several years, there has been significant progress toward reaching these goals. Modeling tools like BioNetGen or Moleculizer [32,33] now better reflect biology by utilizing interactions between discrete binding sites as the elementary units of a reaction scheme. However, those methods still require the user to use a special syntax for writing definition files of molecular properties, imposing a steep learning curve requirement that limits widespread application. In addition, although bimolecular interactions are the building blocks for biochemical cellular signaling networks, many cellular phenomena involve the processing of spatially inhomogeneous extracellular stimuli and the formation of intracellular concentration gradients. Such processes cannot be described properly by models that assume well-stirred biochemistry [34]. For simulations of reaction-diffusion systems that take the location of molecular species into account, specialized mathematical descriptions of intracellular space have been developed (see, e.g., [34,35]), but these methods do not provide a general means of conducting such model building and simulation. An important advance that addresses some of these latter limitations is the “Virtual Cell” framework [30]. It offers a graphical interface for the construction of spatially highly resolved simulations of single cells, including a capacity for direct importation of microscopic images into the modeling environment. Despite its sophistication, however, the software still requires the user to define explicitly every reaction in a signaling network and is limited to single-cell simulations. Other tools that address the issue of spatial information in modeling (for example, MCell [36]), while providing excellent capacity for high-resolution model building, require the user to learn a new, complex scripting language for their operation and/or can only operate with models involving very few molecular components. The modeling and simulation software *Simmune* that we used for the development and quantitative exploration of our chemosensing model was created as an effort to overcome many of these constraints. In accord with the manner in which most biologists think about signaling pathways, Simmune allows molecular interactions to be defined directly at the level of binding events between molecular domains using simple graphical representations of molecules and molecular complexes. Like other approaches [29–33], Simmune can create text files with sets of differential equations describing the modeled molecular signaling networks that can be imported into differential equation solvers. However, Simmune's own, built-in, time integration methods and its simulation interface have important advantages over general differential equation solvers. For example, they allow the modeler to select specific molecular complexes or families of complexes that share common properties for a detailed inspection of their spatiotemporal behavior during ongoing simulations. Detailed information on the schemes used by Simmune to solve the problem of translating graphical input into mathematical representations of interactions between molecular species and of the resulting complexes, as well as to allow these interactions and complexes to be tracked in space and time during a simulated process of reaction and diffusion, are provided in Texts S3, S4, S5, and S6. Simmune can handle models involving tens or even hundreds of individual molecular components, eliminating the need to neglect a known component of a pathway due to computational complexity. The major limitation becomes simulation speed as the model complexity grows. Bridging the scale gap between intracellular biochemistry and the behavior of multicellular systems, Simmune can also create populations of simulated “cells” with spatially resolved reaction-diffusion processes taking place within the cytoplasm and on the plasma membrane. Each cell is individually identifiable and situated in three-dimensional representations of tissue culture vessels or tissues. Simulated cells can be added by mouse click at user-defined positions and then exposed at any desired point in the simulation to new ligands by the localized introduction of molecules or other cells and by the definition of static or dynamic molecular concentration fields. Each of these actions is readily specified using simple screen commands in the graphical interface. Individual cells can be selected by mouse click for detailed investigation of the distribution of individual molecular species or molecular complexes at any moment during the simulation. Graphs reporting the relevant biochemical state of cytoplasmic or membrane subregions of the cell over time then appear (Figure 4).
