# Supplementary material for: Key Role of Local Regulation in Chemosensing Revealed by a New Molecular Interaction-Based Modeling Method
Source: PLoS Comput Biol. 2006 Jul 21;2(7):e82. doi: 10.1371/journal.pcbi.0020082 (PMC1513273; doi:10.1371/journal.pcbi.0020082)
Supplement: Supplementary file 1 [file pcbi.0020082.sd001.pdf]

Text S1:

Rationale for model building

The experimental support for many of the particular additional biochemical elements we have added to the core *Dictyostelium* chemosensing pathway comes from studies in mammalian cells and not *Dictyostelium*. We believe it is nevertheless justified to construct a model based on such data, because the goal of the exercise was to test a hypothetical signaling network for its ability to predict biological behavior. If this can be done with such a hybrid model that combines experimental data from different species, then a strong impetus would be created to look for the *Dictyostelium* functional equivalents of the molecular components drawn from the mammalian literature and to develop a detailed, experimentally validated, second-generation biochemical scheme. This process is quite comparable to the more familiar use of information from studies in one species to formulate hypotheses guiding wet lab experimentation in another (for example, the use of *C. elegans* data on apoptosis to identify Bcl-2 as an anti-apoptotic protein in mammalian cells [1]).

In addition, most of the molecular concentrations and interaction rates required for simulations based on this model have not been directly measured and only limited tools exist for such analyses at present [2,3]. We therefore estimated parameter ranges based on information available about homologous or related molecules reported in the literature, when available, then refined the initial values of these parameters and simultaneously estimated those for which such information is not available by searching the parameter space iteratively until the simulation output reproduced the previously known spatiotemporal dynamics of PIP_3 and PTEN [4,5] and our own measurements of G protein activation in these cells after stimulation with homogenous concentrations of cAMP [6]. It has to be noted, however, that until efforts in high-throughput quantitative proteomics provide extensive data sets detailing intracellular molecular concentrations and reaction rates, models of signaling networks as complex as the eukaryotic chemosensory machinery will frequently contain too many unknown parameters to perform best-fit estimates of particular parameter values.

One approach for limiting the number of parameters that need to be determined experimentally involves analyzing the sensitivity of such a model’s behavior towards

changes in each of its parameters and measuring experimentally only those that most strongly influence simulation outcomes. In Fig. S8 we demonstrate how Simmune's parameter scanning capabilities can be used to investigate the change of behavior of the simulated cellular responses using automatic variation of multiple biological parameters. However, spatially resolved simulations of very complex reaction networks come at high computational cost and a thorough sensitivity analysis at this scale is a project unto itself. The purpose of the present study was to explore plausible candidates for the signaling components of the eukaryotic chemosensory response by simulations involving reasonable parameter sets and to assess whether the implemented model had predictive power. If so, then the next steps will be to verify the identity of the proposed molecular interactions through experimental biochemical analyses and to conduct a broader sensitivity analysis to identify concentration and rate parameters whose accurate measurement is central to the behavior of the model.

1. Hengartner MO, Horvitz HR (1994) Activation of C. elegans cell death protein CED-9 by an amino-acid substitution in a domain conserved in Bcl-2. Nature 369: 318-320.
2. Schoeberl B, Eichler-Jonsson C, Gilles ED, Muller G (2002) Computational modeling of the dynamics of the MAP kinase cascade activated by surface and internalized EGF receptors. Nat Biotechnol 20: 370-375.
3. von Dassow G, Meir E, Munro EM, Odell GM (2000) The segment polarity network is a robust developmental module. Nature 406: 188-192.
4. Funamoto S, Meili R, Lee S, Parry L, Firtel RA (2002) Spatial and temporal regulation of 3-phosphoinositides by PI 3-kinase and PTEN mediates chemotaxis. Cell 109: 611-623.
5. Iijima M, Devreotes P (2002) Tumor suppressor PTEN mediates sensing of chemoattractant gradients. Cell 109: 599-610.
6. Xu X, Meier-Schellersheim M, Jiao X, Nelson LE, Jin T (2005) Quantitative imaging of single live cells reveals spatiotemporal dynamics of multistep signaling events of chemoattractant gradient sensing in dictyostelium. Mol Biol Cell 16: 676-688.
