# Supplementary material for: Key Role of Local Regulation in Chemosensing Revealed by a New Molecular Interaction-Based Modeling Method
Source: PLoS Comput Biol. 2006 Jul 21;2(7):e82. doi: 10.1371/journal.pcbi.0020082 (PMC1513273; doi:10.1371/journal.pcbi.0020082)
Supplement: Supplementary file 2 [file pcbi.0020082.sd002.pdf]

Text S2:

Modular analysis of the chemosensing signaling network

The signaling network described here does not contain any abstract elements responsible for behaviors such as ‘adaptation’ or ‘gradient amplification’. Nevertheless, it is possible to analyze its components in terms of ‘modules’ with such functionalities:

1) The ‘positive signal transduction module’: The cAMP receptor in its activated state (RecCAMP_act) activates the receptor-bound Gα. Gα, once activated, dissociates from Gβγ (Gbetagamma). Free Gβγ activates Ras. Active Ras transforms PI3K into PI3Kact, which can attach itself to the membrane and phosphorylate PIP_2 to produce PIP_3. Active Gα, on the other hand, activates the SrcActivator, which transforms Src into Src_act. Src_act phosphorylates PTEN, producing pPTEN which cannot bind to the plasma membrane.

2) The ‘adaptation module’: Gβγ activates a tyrosine phosphatase (PI3Ktp) that deactivates PI3Kact (interaction (1) in Fig. 2A) and RasGAP, which negatively controls Ras activity (interaction (2) in Fig. 2A). It also activates a phosphatase that dephosphorylates pPTEN to allow PTEN to return to the plasma membrane. Csk, recruited to the membrane via pPaxillin deactivates Src_act (interaction (2) in Fig. 2B). SHP2, which is recruited to the membrane by Gab1 that, in turn, binds to PIP_3, negatively controls the amount of pPaxillin (interaction (3) in Fig. 2B). With decreasing levels of PIP_3 (due to the deactivation of PI3K), more Csk is recruited to the membrane to deactivate Src, thereby reducing the rate of phosphorylation of PTEN and supporting the reassociation of PTEN with the membrane. SHIP, which is recruited to the membrane via pSHIPanch, negatively controls PIP_3 levels. The amount of pSHIPanch is locally controlled by PTEN (through dephosphorylation). In regions with high concentrations of PTEN, the concentration of pSHIPanch is low. Loss of PTEN allows pSHIPanch to accumulate and to recruit SHIP, which then controls the level of PIP_3 in the absence of PTEN.

The coupling between the deactivation of PI3K and the reassociation of PTEN with the membrane noted above means that a weaker adaptation of PI3K would result in a slower adaptation of PTEN. This has indeed been observed experimentally [1]. In Fig. S9 we show the change in the behavior of PTEN resulting from allowing even non-

activated PI3K to attach itself to the plasma membrane where it can interact more directly with activating Ras. The hyperactivation of PI3K results in an incomplete return of PTEN to the membrane – in accordance with the previously reported experimental data.

3) The ‘gradient amplification module’: Local levels of pPaxillin (which recruits the Src inhibitor Csk – see above) are negatively controlled by SHP2, which becomes recruited to the membrane by pGAB1, which, in turn, binds to PIP_3 via its PH domain. Local increase of PIP_3 therefore leads to an increase in the concentration of SHP2, which reduces the local concentration of pPaxillin and, consequently, of Csk, allowing Src_act to accumulate and phosphorylate PTEN (which then leaves the membrane) (interaction (1) in Fig. 2B). This closes a feedback loop because a loss of membrane-bound PTEN leads to an increase of PIP_3. Even a relatively small difference in the receptor signal between two sides of the cell, resulting in initially small differences in the levels of PIP_3 and membrane-bound PTEN, can therefore be amplified by the cell to produce pronounced gradients of PIP_3 and PTEN (with opposite orientations).

Different mechanisms regulating the gradient-induced translocation of PTEN have been proposed, for example, a mechanism according to which the receptor signal is translated into phosphorylation of PTEN without feedback elements [2]. Here, we implement such an alternative mechanism by allowing active Gα to induce phosphorylation of PTEN while activating the phosphatase that dephosphorylates PTEN through Gβγ, as before. After appropriate adjustment of the model parameters, this modified model was able to qualitatively reproduce the behavior of membrane-bound PTEN after homogenous stimulation with cAMP (Fig. S10A). When we simulated the responses of cells with this model to stimulation with gradients of cAMP, however, the lack of a positive feedback regulating the translocation of PTEN manifested itself by an inability of the signaling network to reproduce the proper polarization behavior of PTEN. With such a model, PTEN is accumulated in the back and lost in the front (Fig. S10B), at low absolute concentrations of cAMP, but with dynamics that do not match the experimentally observed data (see Fig. 6), and at higher concentrations of cAMP there is a failure to induce a pronounced intracellular polarization of PTEN (Fig. S10C). The reason is that for higher concentrations of cAMP, the differences between the levels of receptor activation in the front and the back of the cell become smaller due to the onset of

saturation, therefore requiring intracellular amplification to be able to induce steep intracellular gradients of PIP_3 and PTEN.

1. Funamoto S, Meili R, Lee S, Parry L, Firtel RA (2002) Spatial and temporal regulation of 3-phosphoinositides by PI 3-kinase and PTEN mediates chemotaxis. Cell 109: 611-623.
2. Ma L, Janetopoulos CJ, Yang L, Devreotes PN, Iglesias PA (2004) Two local excitation, global inhibition mechanisms acting complementarily in parallel can explain the chemoattractant-induced PI(3,4,5)P3 response in Dictyostelium. Biophys J.
