# Supplementary material for: Key Role of Local Regulation in Chemosensing Revealed by a New Molecular Interaction-Based Modeling Method
Source: PLoS Comput Biol. 2006 Jul 21;2(7):e82. doi: 10.1371/journal.pcbi.0020082 (PMC1513273; doi:10.1371/journal.pcbi.0020082)
Supplement: Supplementary file 3 [file pcbi.0020082.sd003.pdf]

Text S3:

Note on the determination of parameter values for the chemosensing model

It is important to point out that construction of the model and the initial parameter fitting that led to the characteristic biphasic dynamics of PIP_3 and membrane-bound PTEN in the front of the cell was done using experimental data sets generated under conditions (homogeneous cAMP exposure) distinct from those (transcellular gradients) relevant to the main predictions that were eventually examined by experiment. Based on the experimental data confirming the predicted biphasic response of PIP_3 in the cell's anterior, we then finely adjusted the model parameters further such that the position of the 'dip' of PIP_3 in the simulation coincided with the experimentally determined time point. The simulations then correctly predicted the behavior of PIP_3 for different concentrations of applied cAMP and the spatiotemporal dynamics of PTEN. We have thus described two rounds of prediction. With the assumptions about molecular species involved in the sensing machinery, and given a limited input / training data set, the model was able to produce major testable predictions about experimental conditions not used in this set-up procedure. Data generated in the course of testing these first generation predictions not only validated the model but also provided information used to further adjust the model's parameters and to make additional predictions, which in turn were validated as well. It becomes a matter of some interest to know if the specific parameters chosen are indeed close to reality, or if a form of biological 'robustness' as discussed by [1] is at play, with many possible parameter set solutions fitting the data, of which only some are biologically meaningful.

1. von Dassow G, Meir E, Munro EM, Odell GM (2000) The segment polarity network is a robust developmental module. Nature 406: 188-192.
