# Supplementary material for: Key Role of Local Regulation in Chemosensing Revealed by a New Molecular Interaction-Based Modeling Method
Source: PLoS Comput Biol. 2006 Jul 21;2(7):e82. doi: 10.1371/journal.pcbi.0020082 (PMC1513273; doi:10.1371/journal.pcbi.0020082)
Supplement: Supplementary file 4 [file pcbi.0020082.sd004.pdf]

Text S4:

From molecular interactions to cellular behavior – how Simmune works

Scales of modeling:

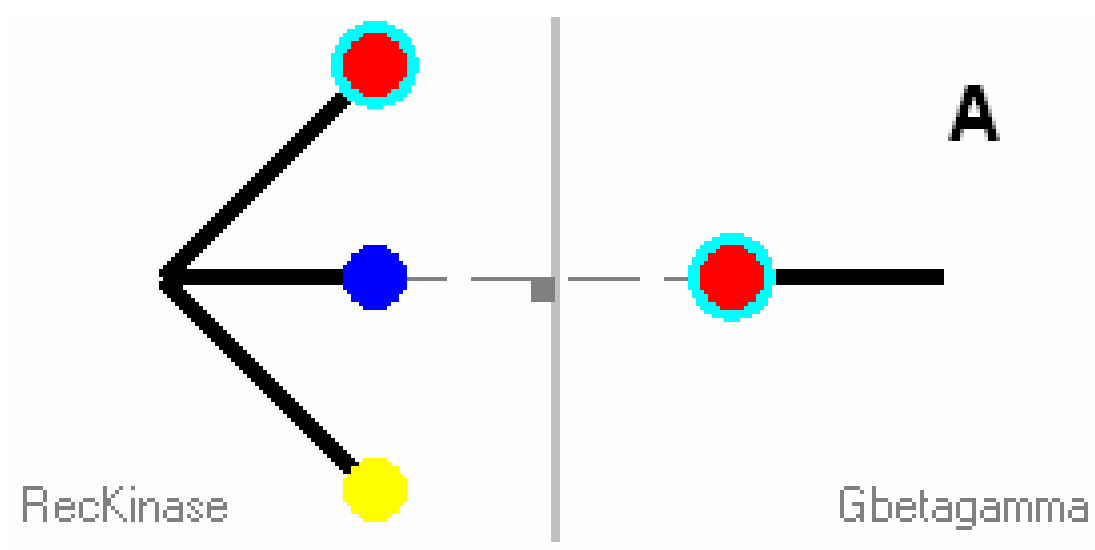

The models that are simulated with Simmune describe biological components and their interrelationships on several scales. The fundamental level involves interactions between molecular binding sites (A). The next scale deals with molecular complexes. The components from these first two scales are used for the definition of cellular stimulus-response mechanisms (Fig. S11). Finally, the compartments representing extracellular space form the highest level in the hierarchy of model components.

Binding events and their consequences:

By defining the interactions between molecular binding sites (see Fig. A, Movie 2, Text S9), the modeler provides the information that Simmune needs to create the biochemical network of molecular complexes and their reactions (Fig. S1). Molecules and molecular complexes can participate in three fundamental processes: they can bind to other molecules or complexes, they can decay into their components, and they can undergo transformations that can be either spontaneous or enzymatically induced. In addition to specifying which particular molecular interactions can occur, the modeler must therefore also enter association rates for interactions between molecular binding sites and dissociation rates that describe how rapidly an assembled complex undergoes separation back to its component elements. In addition to this primary rate information, the modeler also defines whether association or dissociation events induce changes in the reaction properties of the participating molecules. In such cases, the modeler enters information about the rates at which molecular complexes undergo spontaneous transformations and the rates at which enzymatically active complexes transform their substrates.

Complexes and spatially-constrained reactions:

Based on these inputs, Simmune determines which molecule complexes can form during a simulation of the model, the biochemical reactions in which they can participate, and rates of these reactions (see Texts S4, S5 for details). In order to trace the spatio-temporal behavior of specific complexes or to define their role in enzymatic transformations the modeler can build representations of these complexes by simple graphical operations (Fig. S12).

Simmune employs the user inputs defining reaction rates, the diffusion coefficients for the molecules in the model, and the number of each type of molecule present in the simulated cells and the extracellular space at the start of the simulation to calculate how the distribution of molecular complexes changes over time. Most typical differential equation solvers used for modeling describe the interactions of all possible complexes at all locations of the system. In contrast, Simmune always minimizes the computational cost by creating reaction networks for the different regions of the simulation that reflect only the local molecular contents. Such a dynamic treatment of the reaction-diffusion system cannot be readily accomplished with standard differential equation solvers (see Text S6 for a description of how the formal representation of the reaction network is created and Text S7 for a description of the spatial resolution used in the chemosensing simulations reported here).

Stimulus-response mechanisms:

While some phenomena are understood in great detail at the molecular level, other processes have been studied only on the coarser scale of whole cell responses, or even as populations of interacting cells. Even when the underlying molecular events are reasonably well known, it can be useful to model cell behavior at a higher, less fully defined, scale. One example might be analysis of the migration of a population of cells in gradients of chemoattractants, without explicitly considering all of the details of intracellular signaling and actin regulation. Directional cell migration in response to such gradients can be simulated based only on asymmetrical chemokine receptor occupancy and a resulting rule for cell movement [1].

Although not employed in the present detailed analysis of the intracellular events involved in chemosensing, Simmune permits the definition of cellular stimulus-response

mechanisms to accommodate this higher scale of description. These mechanisms link the appearance of a threshold concentration of specific molecular complexes in some part of the cell to macroscopic responses such as secretion, expression of new membrane receptors, movement, or cell-type transitions such as differentiation, division or death. Here, Simmune's concept has some overlap with cellular automata simulators like ImmSim [2], but it differs from these methods in important ways. In particular, whereas cellular automata define discrete cell states, Simmune works with a quasi-continuum of cell states defined by the individual biochemistry of each cell. Using this feature of Simmune, it is possible to define a reduced complexity model of cellular biochemistry that can simulate the macroscopic responses of the cell to discrete molecular stimuli. This is done with the help of *stimulus-response mechanisms*. A stimulus-response mechanism links actions a cell can perform to conditions that have to be fulfilled before the cell actually undertakes the actions (Fig. S11). Typically, such conditions consist of the existence (or absence) of a certain threshold amount of particular molecular complexes at certain locations within a cell or on its surface. Simmune also offers two pseudo conditions: “INITIALLY” and “DEFAULT”. INITIALLY is used for actions a cell should perform only once immediately when it is created (i.e., when it enters the simulation). DEFAULT is used for actions that should be performed repeatedly. For actions that are to be performed more than once, a timescale has to be specified: this determines the time a cell must wait after it has performed an action before it can perform the same action again (if the mechanism's conditions are fulfilled). Cells and molecules in Simmune ‘live’ in three-dimensional compartments simulating *in vivo* or *in vitro* space. The volume of these compartments is subdivided into small elements that can be occupied by either cells or molecules (for details see Texts S5, S6). The locations (or directions) specified in the conditions and actions are always relative to locations defined by the stimuli a cell receives. For example, a cell might secrete a particular molecule at the side through which the cell received the stimulus for the secretion. The condition in this case would be the ligation of receptors on a particular side of the cell. The action would be the secretion of the signaling molecule to the side defined by the ligation event (Fig. S11).

Texts S8-S14 and movies S1-S7 give introductions/tutorials for the different steps of model building and analysis with Simmune.

1. Meier-Schellersheim M (2001) The Immune System as a Complex System: Description and Simulation of the Interactions of its Constituents. Hamburg: University of Hamburg.
2. Celada F, Seiden PE (1992) A computer model of cellular interactions in the immune system. Immunol Today 13: 56-62.
