# Supplementary material for: Key Role of Local Regulation in Chemosensing Revealed by a New Molecular Interaction-Based Modeling Method
Source: PLoS Comput Biol. 2006 Jul 21;2(7):e82. doi: 10.1371/journal.pcbi.0020082 (PMC1513273; doi:10.1371/journal.pcbi.0020082)
Supplement: Supplementary file 5 [file pcbi.0020082.sd005.pdf]

Text S5:

Simmune’s internal representation of molecular complexes

One of the technical challenges associated with the automated construction of the network of interacting complexes is to find a formalism for unique and numerically efficient representation of molecular complexes. Molecular complexes are characterized by their component molecules and the binding contacts between them. These two sets of data (components and binding contacts) can be translated into unique identifiers for molecular complexes. In Simmune, each molecule possesses a unique numerical ID. Each molecular complex in turn contains a list of the molecule types forming the complex. The molecule types within these lists are ordered according to their numerical IDs. The binding contacts are encoded by assigning each element (molecule) of the complex a list of pairs of numbers indicating the binding contacts mediated by each of its binding sites: the first number encodes the binding partner, the second number the partner's binding site involved in the binding. This additional information, attached to each molecular component of a complex, can be used determine (up to isomorphisms) the order of the molecular components within the component list of the complex even in those cases in which the complex contains several instances of the same molecule type. The unique representation of complexes is important, for example, during the construction of the signaling network (to avoid duplicate complexes).
