# Supplementary material for: Key Role of Local Regulation in Chemosensing Revealed by a New Molecular Interaction-Based Modeling Method
Source: PLoS Comput Biol. 2006 Jul 21;2(7):e82. doi: 10.1371/journal.pcbi.0020082 (PMC1513273; doi:10.1371/journal.pcbi.0020082)
Supplement: Supplementary file 6 [file pcbi.0020082.sd006.pdf]

Text S6:

Automatic creation of the reaction network

When the modeler starts a simulation, Simmune first creates the network of all possible molecular complexes (up to the user-defined upper limit of the number molecular components (molecule types) within single complexes). The molecular complexes are characterized by their component molecule types, their binding code lists (see Text S5), and the set of reactions in which they participate. The reactions are categorized as follows: 1) Association events: these events are characterized by the binding partner, the resulting complex, and the association rate. The resulting complex, in turn, is uniquely specified by the reaction partners and the binding sites involved in the binding event. Some reactions may involve changes in the characteristics of one (or both) of the molecules involved in the interaction. A typical example is allosteric modification that renders a binding site more or less accessible to its binding partners. In Simmune, such changes are internally handled by replacing the molecules originally involved in the formation of the complex with other user-defined molecules that possess the new characteristics imparted by the binding reaction but that have the same list of binding partners as the original molecules. For the example of an allosteric modification that alters the accessibility of binding sites, the replacement molecules would have higher or lower association rates for the reactions that use those sites than the original molecules. (Such molecules are called 'alternatives'. See also part 1 of the Simmune tutorial, here Movie 2, Text S9). 2) Dissociation events: these events are characterized by the molecules or partial complexes that remain after the dissociation event and the applicable dissociation rate. Like association events, dissociation events may involve changes in the characteristics of molecular components involved in the event. 3) Transformations: Spontaneous transformations of assembled complexes take place according to user-defined transformation rates and give rise to new complexes with altered molecular characteristics. An enzymatically-induced transformation additionally carries information about the inducing enzyme and the molecular component within the complex that is the enzyme's direct substrate (for the particular transformation). Simmune does not use the Michaelis-Menten approximation for enzymatically-induced transformations because this approximation (in its simple form) is not valid in cases in which the enzyme can act on

different competing substrates or where its enzymatically active site can become bound by an inhibitor that competes with the enzyme's substrate(s).

The complexes within this full network are represented in computer memory by large data structures. For the calculation of the local biochemical reactions for different cell types or different regions within cells, Simmune constructs local networks of molecule complex representations that consist of smaller data sets describing only reactions that can occur based on the particular local biochemistry and just containing references to the concentrations (or molecule numbers) affected by the reactions and the reaction rates. The construction of these reduced representations can become complicated if reactions establish links between separate spatial regions, for example, between membrane-associated molecules and molecules in the extracellular milieu (This is the case for membrane receptor ligation events). Then, the program needs to identify not only the types of molecules and complexes involved in the reactions, but additionally needs to keep track of the spatial origin of the reacting components.

To integrate the reaction-diffusion system, Simmune first collects all reactions in which each complex participates, and then calculates the contributions of these reactions to the rate of change of the concentration of each complex (which depends on the reaction rates and the – typically changing – concentrations of the reaction partners). It then adds to these rates the rates of concentration change due to molecular diffusion. Because of the large number of different complexes that can be formed and for which the concentration has to be calculated (note that the total number of concentrations that have to be calculated is given by the sum of the complex types in the different extracellular and intracellular regions in the simulated model), Simmune does not use matrix representations of the reaction-diffusion system but employs a 'Jacobian-free' method to integrate the system over time, which is capable of a very efficient and precise treatment of stiff ODEs [1]. Also, because of the very large number of interacting components, we are currently unable to perform stochastic simulations of the chemosensing network (a version of Simmune that uses a modified Gillespie algorithm [2] for stochastic simulation will become available soon). However, in the network investigated here and with the currently used relatively coarse spatial discretization, the amounts of locally interacting

molecule complexes are always high enough to justify the usage of the deterministic mass-action formulation of the biochemical reactions.

1. Weiner R, Schmitt B, Podhaisky H (1996) ROWMAP - a ROW-code with Krylov techniques for large stiff ODEs. Report 39, FB Mathematik und Informatik, Universitaet Halle.
2. Gillespie D (1976) A general method for numerically simulating the stochastic time evolution of coupled chemical reactions. Journal of Computational Physics 22: 403-434.
