# Supplementary material for: Key Role of Local Regulation in Chemosensing Revealed by a New Molecular Interaction-Based Modeling Method
Source: PLoS Comput Biol. 2006 Jul 21;2(7):e82. doi: 10.1371/journal.pcbi.0020082 (PMC1513273; doi:10.1371/journal.pcbi.0020082)
Supplement: Supplementary file 7 [file pcbi.0020082.sd007.pdf]

Text S7:

Discretization of extra-and intracellular space in Simmune

In the version of Simmune we used for the simulations reported here, extracellular space was discretized into cubic volume elements with a diameter of 10 μm. Within volume elements that are not occupied by cells, the concentration of each molecule species is given by a single number: the average concentration of the molecule within the element. When a cell is placed into an element (either by the modeler directly or by cellular processes like movement or division), the molecular contents that previously filled the volume element are distributed over neighboring elements. The receptors on the cell's surface "see" the molecules in the neighboring elements. Those molecules that bind to the cell's receptors are taken out of the bulk concentration of freely diffusible molecules. The membrane area of cells is represented by 6 area elements, akin to the 6 sides of a cube. In Fig. S13A, a 2-dimensional cut through this structure is shown (note that the structures used in the simulations are 3-dimensional). These area elements are interconnected by membrane-bound diffusion (red arrows in Fig. S13A). The cytoplasmic volume of cells is represented by one central and 6 peripheral volume elements, corresponding to the simplest (on a cubic lattice) discretization of the 3-dimensional Laplace operator (blue squares and blue arrows in Fig. S13A). The sum of the areas of the surface elements is equal to the surface of a sphere of 10 μm diameter. The sum of the cytosolic volume elements is equal to the sphere's volume. A finite difference scheme was used to transform the PDEs of the diffusion part of the reaction-diffusion equations into ODEs.

In order to illustrate that this coarse discretization leads to reasonable results within the limits of its resolution, we compared the simulated diffusion of a membrane bound receptor with a diffusion coefficient of $10^{-14}\, m^2/s$ to the behavior of the analytically solved diffusion on a sphere. We simulated the diffusion starting with a concentration distribution that depends on the polar angle θ as $f(\theta,t) = \frac{1}{4\pi R^2}(1+\cos(\theta))$, where R is the radius of the sphere (in our case 5 μm). The highest concentration is at the 'north pole'. At the south pole the concentration is 0 (Fig. S13B). The integral of this distribution over the sphere's surface is 1. It can be shown that

the time development of this concentration distribution is $f(\theta,t)=\frac{1}{4\pi R^2}(1+\cos(\theta)e^{-2tD/R^2})$ (see e.g. [1]).

At time *t=0*, the fraction of molecules situated on the 'north pole cap' is: $\int_0^{2\pi}\int_0^{\bar{\theta}} f\left(\theta,0\right)R^2\sin(\theta)d\theta d\phi=\frac{1}{2}\left[-\cos(\theta)+\frac{1}{2}\sin^2(\theta)\right]_0^{\bar{\theta}}\approx 0.3$. Here, $\bar{\theta}$ is the polar angle for which $\frac{1}{4\pi R^2}\int_0^{2\pi}\int_0^{\bar{\theta}} R^2\sin(\theta)d\theta d\phi=\frac{1}{6}$ (i.e. the pole cap – indicated in red in Fig. S13B – is defined here to occupy 1/6 of the total surface of the sphere; $\bar{\theta}=\arccos(2/3)\approx 48^\circ$.) The fraction of molecules on the 'south pole' cap at *t=0* is approx. 0.03. To perform the simulation of the molecular diffusion with Simmune, we put 100,000 molecules on the cell surface; approx. $0.3\times 100,000=30,000$ on the upper area element, 3,000 on the lower element and distributed the remaining 67,000 equally over the four 'equatorial' sides of the cell (Fig. S13A). Fig. S13C below shows the comparison of the Simmune's simulated diffusion with the analytical solution. After 500 seconds the discrepancy between the analytical solution and Simmune's simulation is approximately 10% of the total number of molecules that diffused away from the upper membrane region. Note that part of this discrepancy may be due to the special axial symmetry around the z-axis ($\theta=0$) that was introduced by the way the poles were used to populate the upper and lower membrane regions.

1. Buelow T (2002) Spherical Diffusion for 3D Surface Smoothing. 1st International Symp on 3D Data Proc Vis and Transm.
