# Supplementary material for: Key Role of Local Regulation in Chemosensing Revealed by a New Molecular Interaction-Based Modeling Method
Source: PLoS Comput Biol. 2006 Jul 21;2(7):e82. doi: 10.1371/journal.pcbi.0020082 (PMC1513273; doi:10.1371/journal.pcbi.0020082)

Simmune tutorial 1

Creating molecules
Defining interactions between molecular binding sites

(1) Start the Simmune modeler, Simmod.
(2) Create a new model. In our tutorial we call it 'CT_tutorial'.

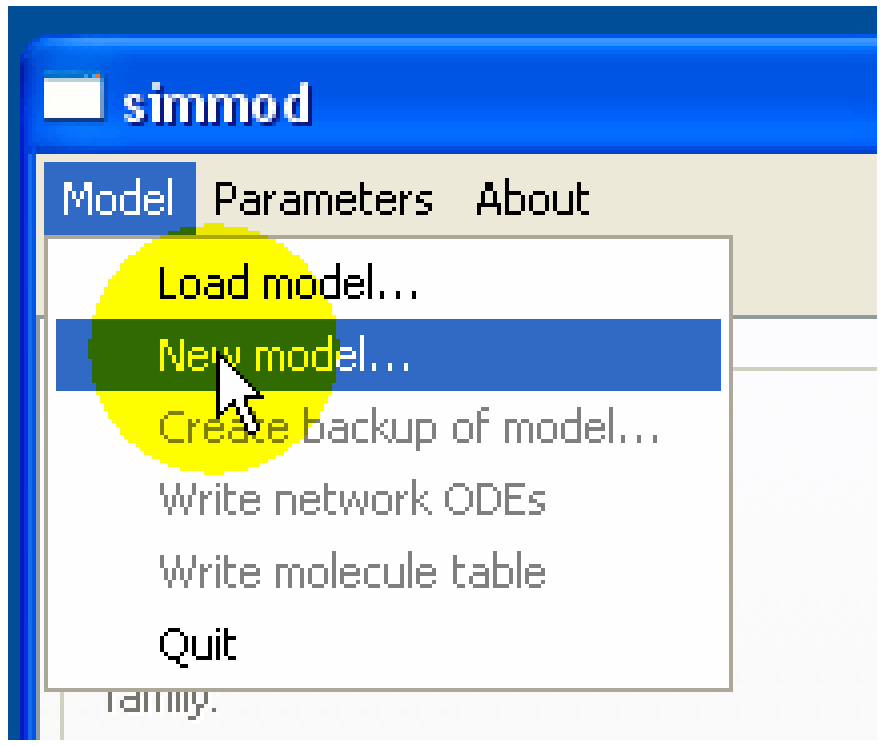

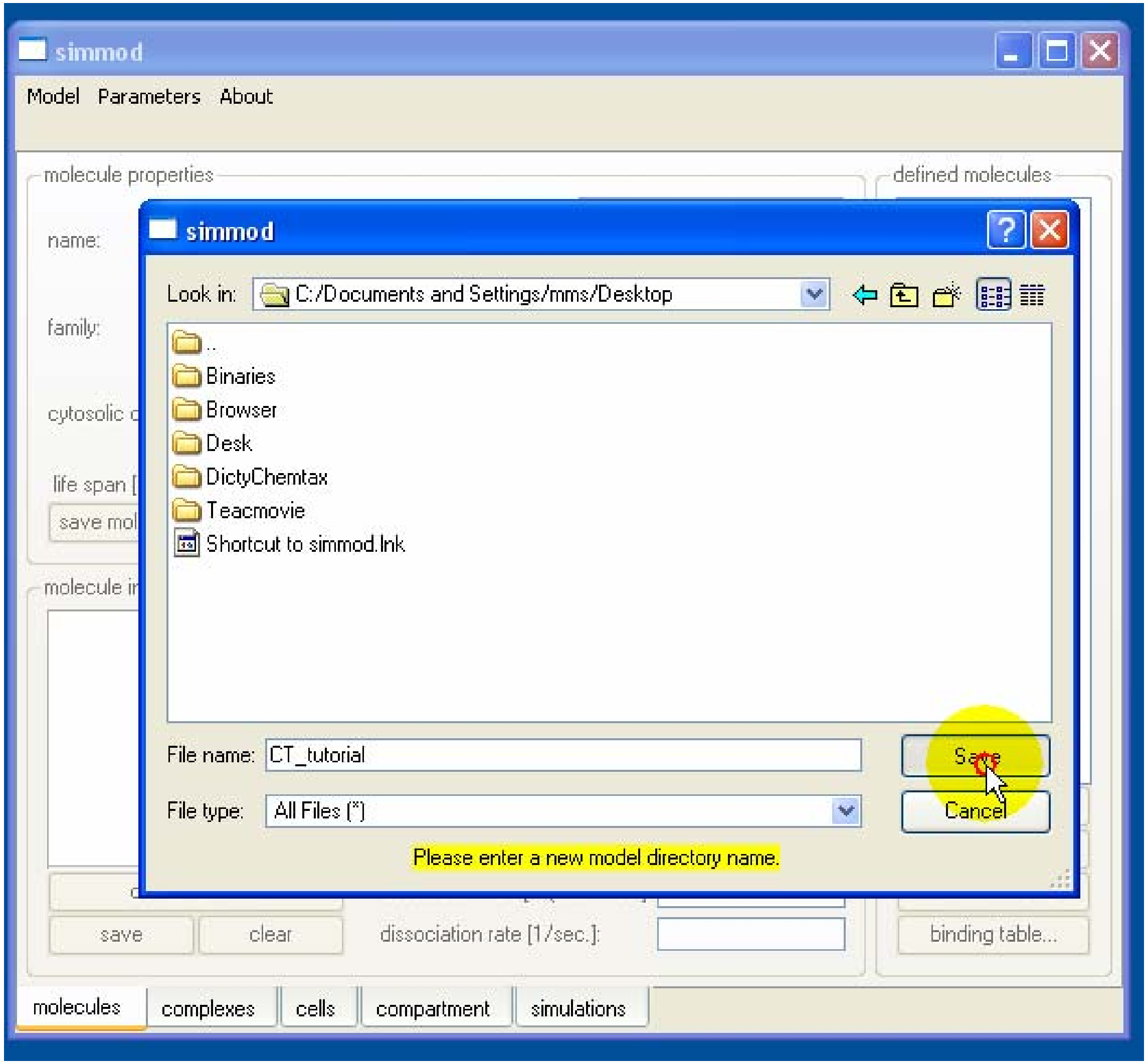

(3) Click the ‘new molecule’ button to create a new molecule.

(4) Define the molecules properties:

- (i) number of binding sites
- (ii) which one of them will be functioning as an enzymatically active site
- (iii) the molecule’s category
- (iv) whether the molecule is freely diffusing or membrane-bound

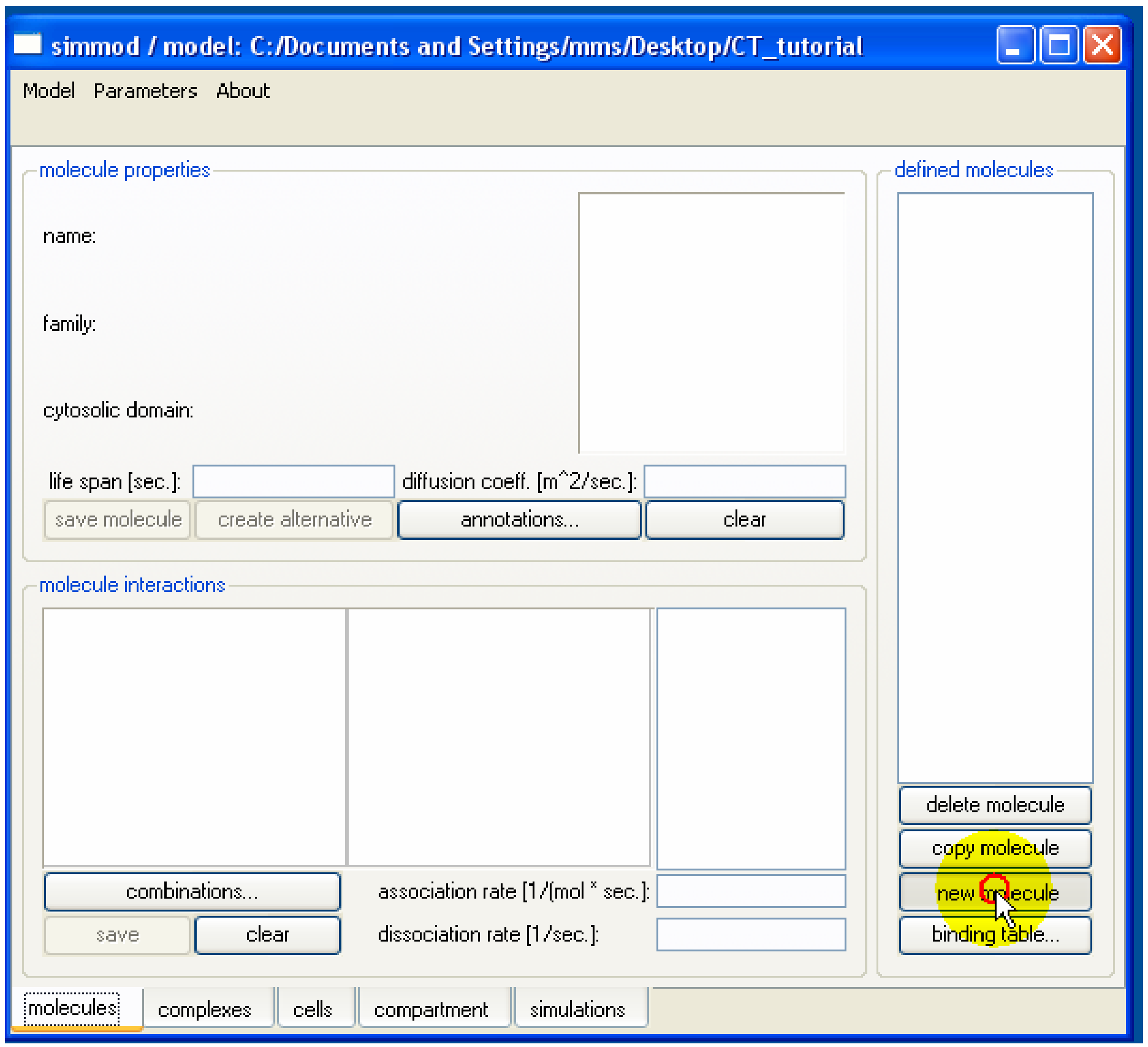

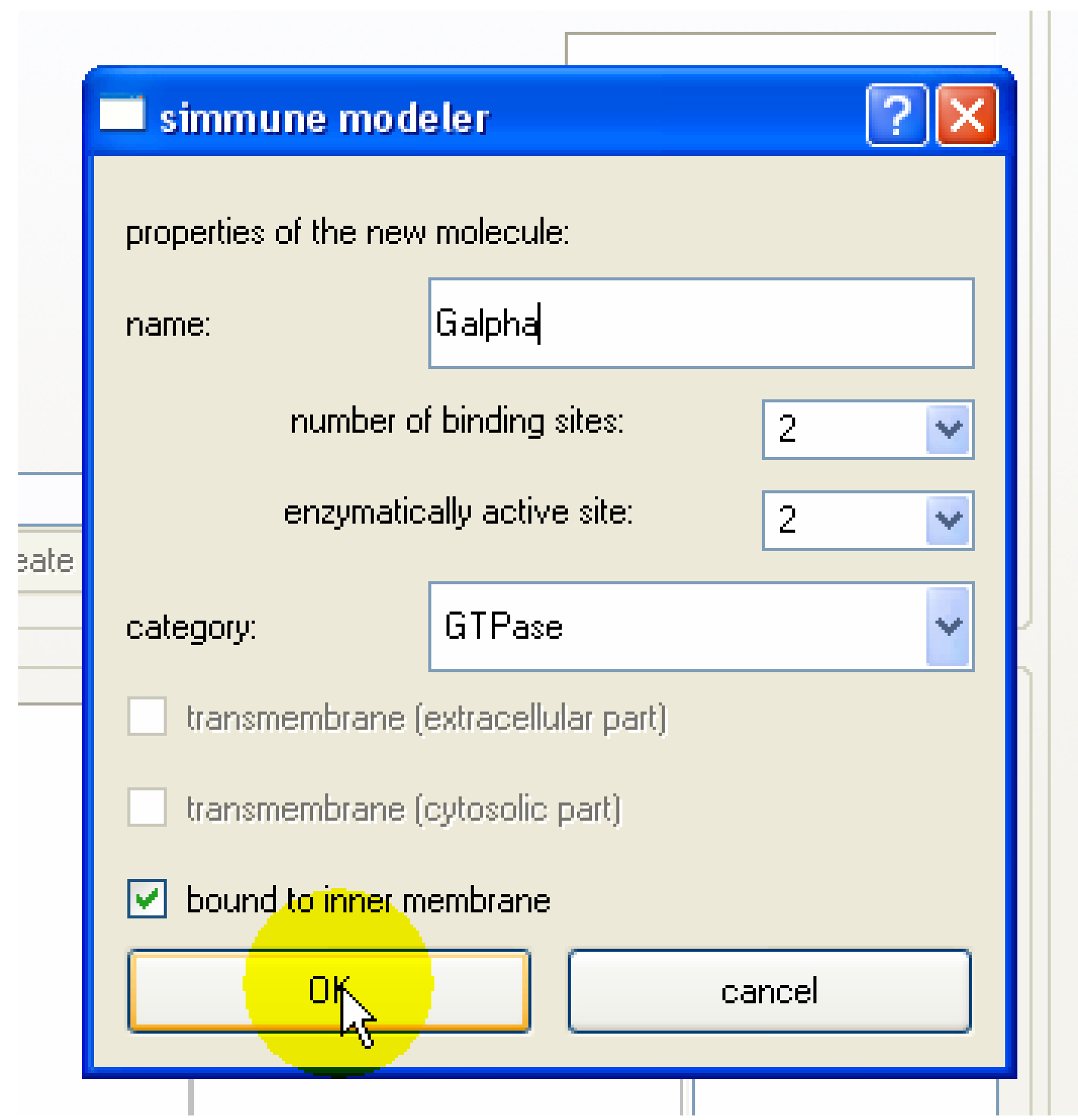

(5) Similarly, create Gbg as a membrane-bound molecule of category 'adaptor' with one binding site and define this binding site to be enzymatically active.
(6) Drag and drop Galpha and Gbetagamma into the 'molecule interactions' field.
(7) Draw a connection between the binding sites of Galpha and Gbetagamma.

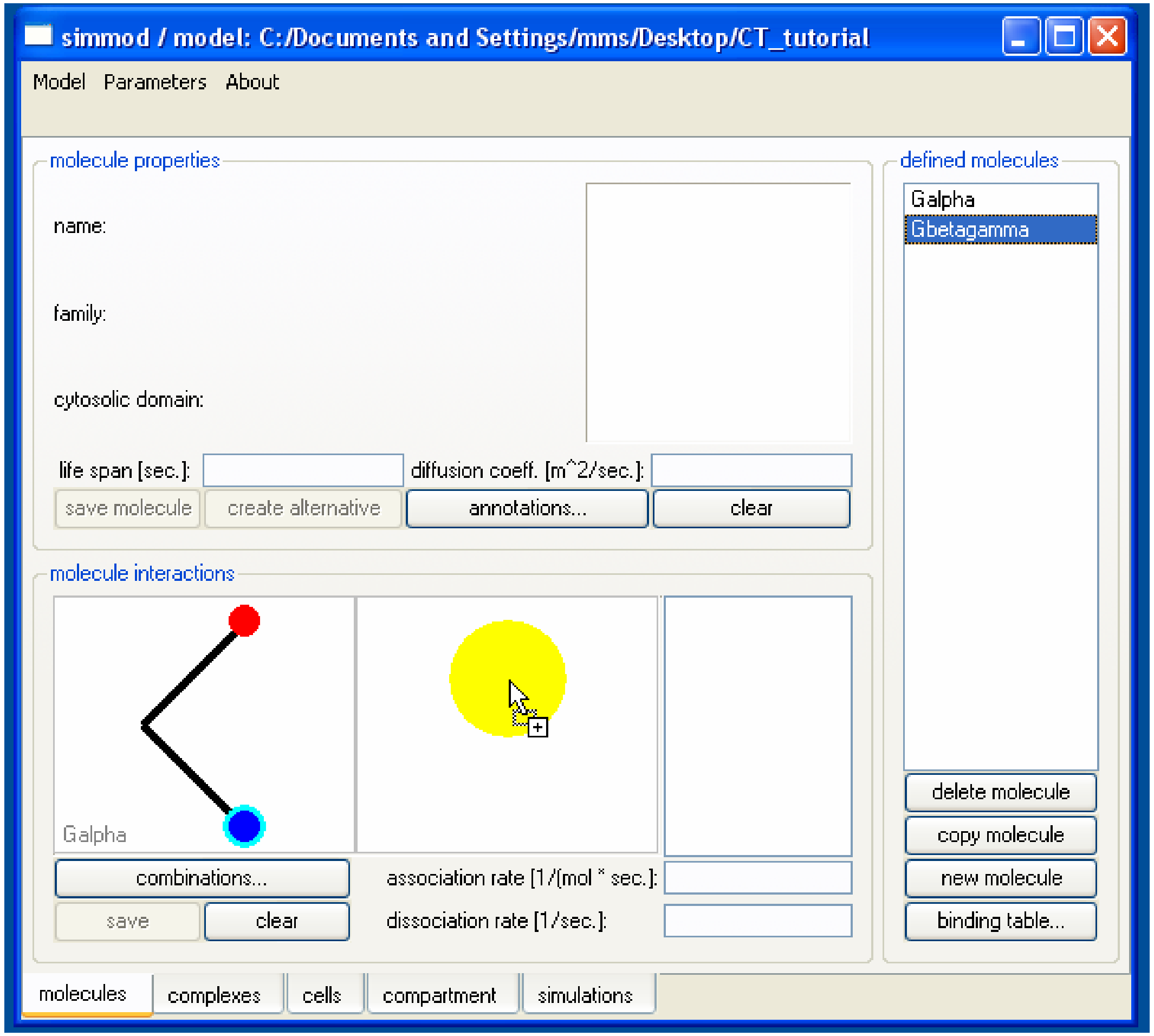

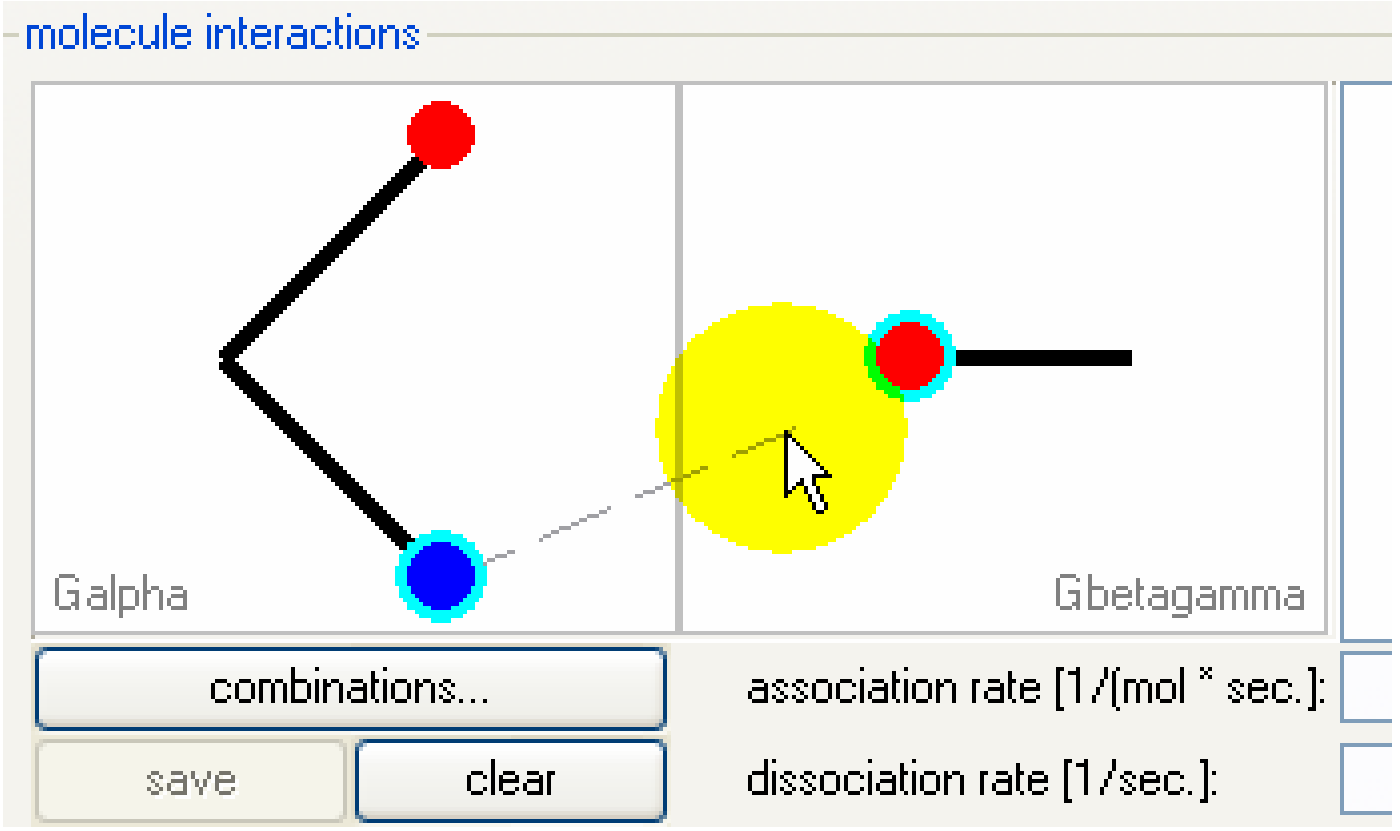

(8) Click the small rectangle at the interaction line between Galpha and Gbetagamma to select it for entering the reaction rates.
(9) Enter the association and dissociation rates.
(10) Don’t forget to save the changes to the molecules’ binding properties.

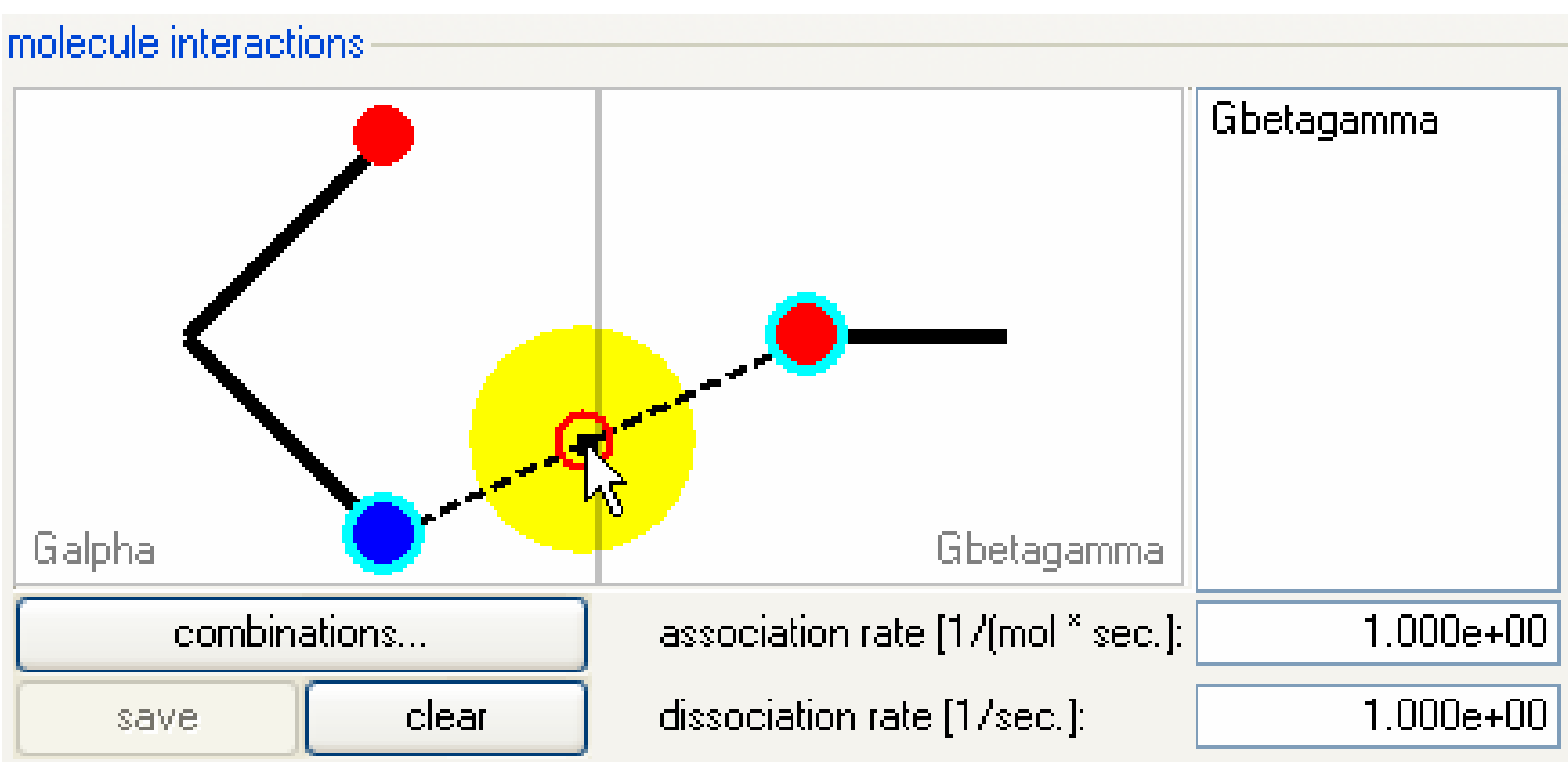

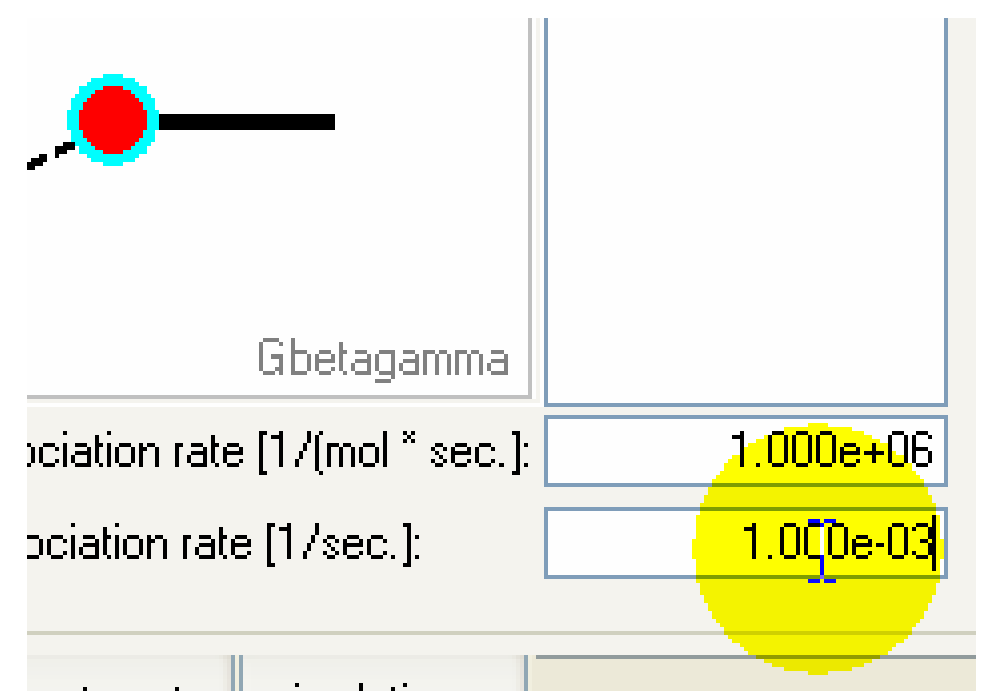

(11) Double-click Galpha and Gbetagamma in the ‘defined molecules’ list to select them for defining properties like diffusion coefficient.
(12) After having selected Galpha click ‘create alternative’ to define an alternative state for Galpha.

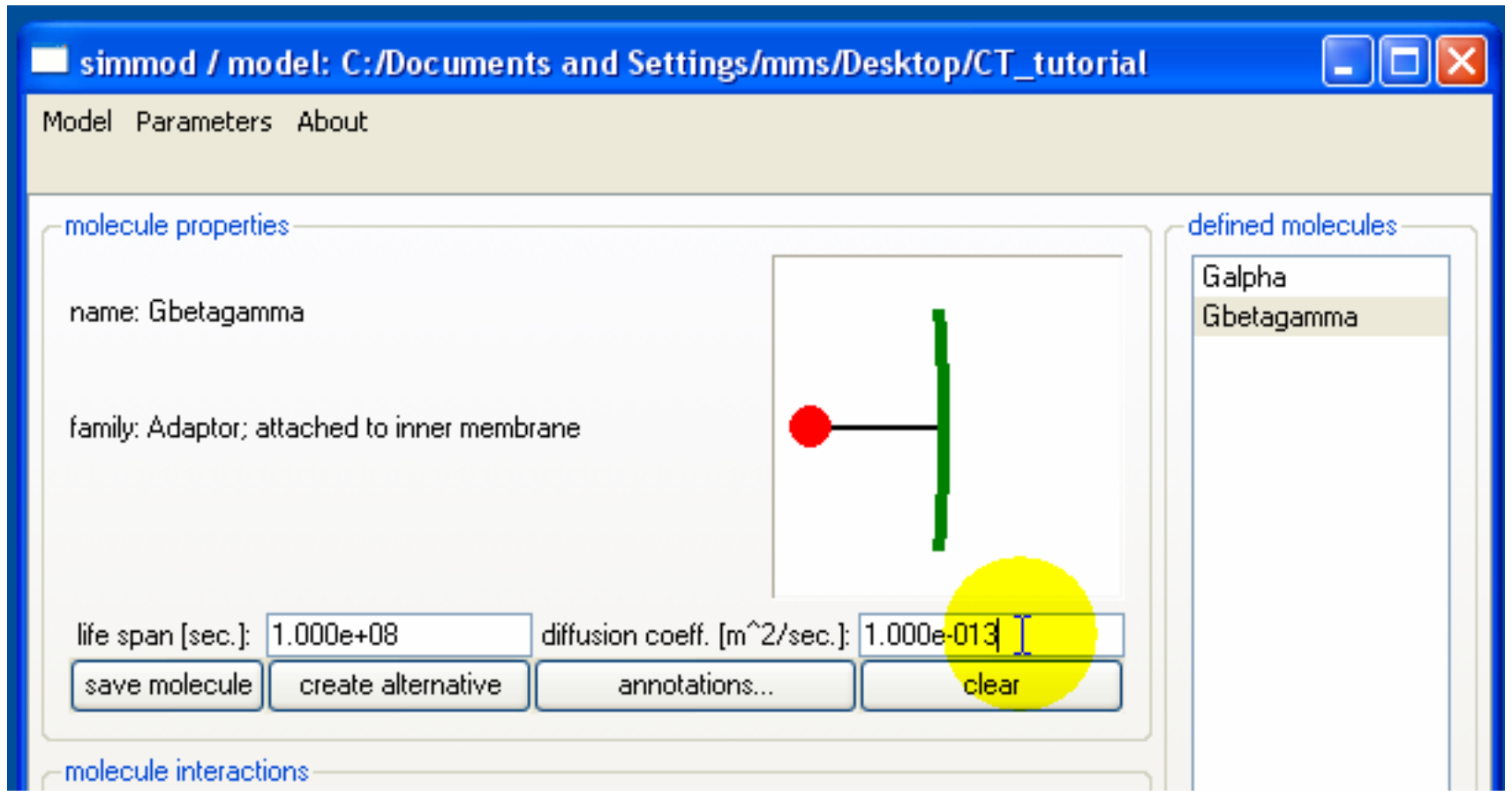

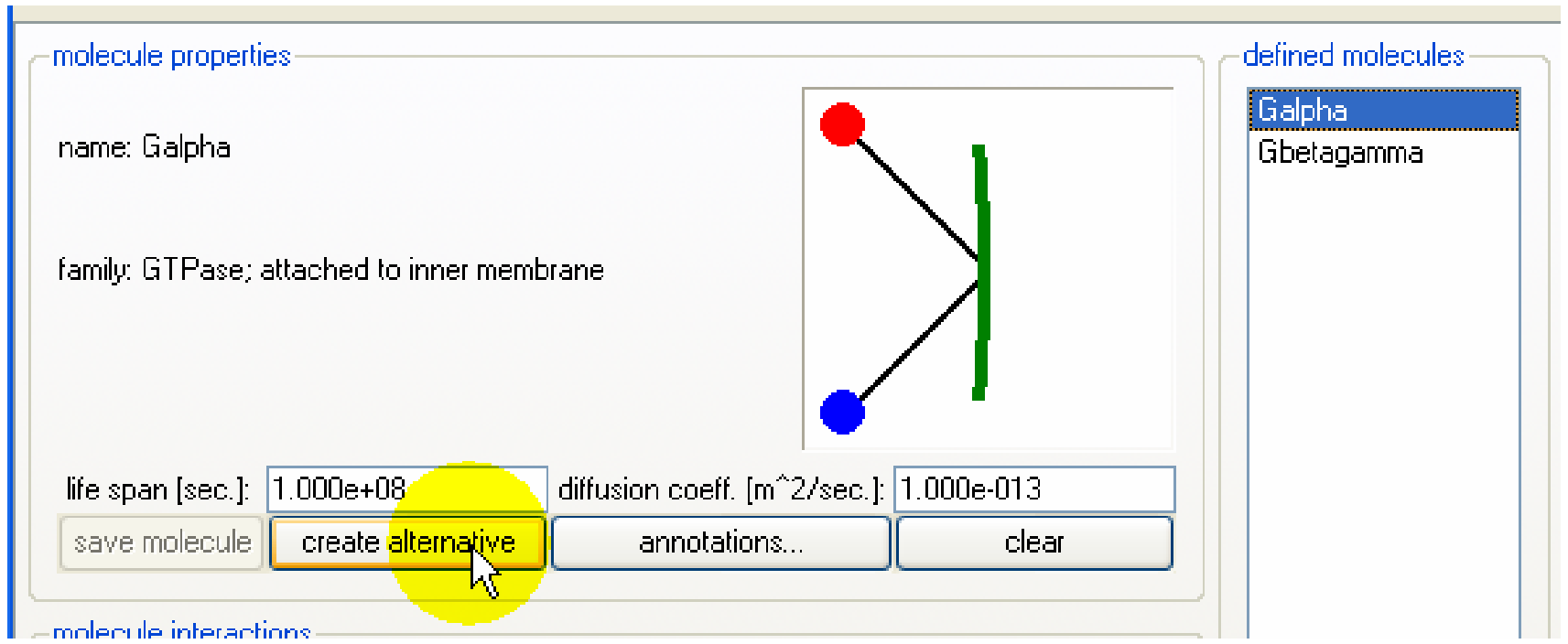

Alternative molecules inherit all properties (including binding possibilities) from their template molecule.

(13) Call the alternative state 'Galpha_bnd'.

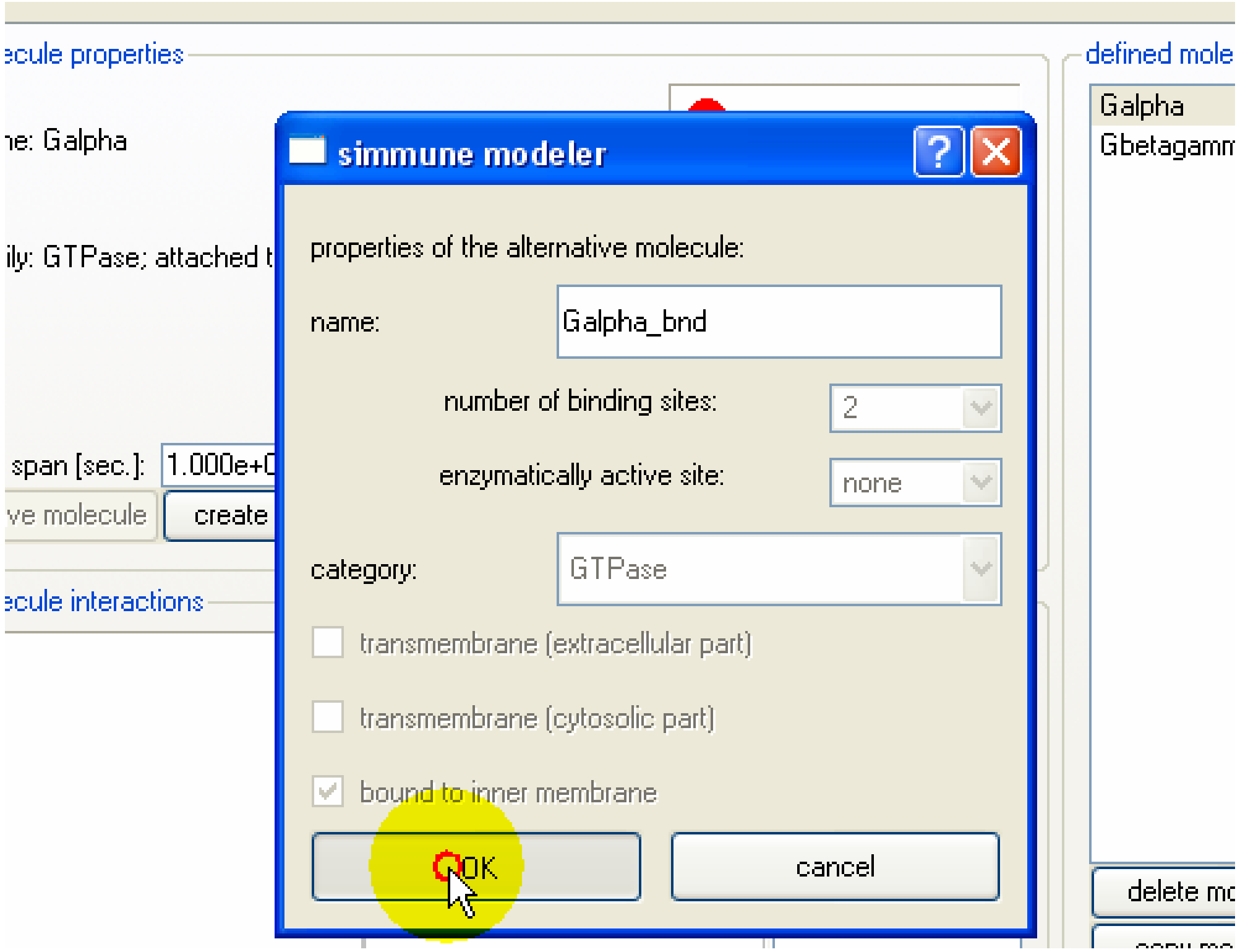

(14) In the ‘molecule interactions’ double-click the link between Galpha and Gbetagamma and then ‘binding properties’ in the small window that appears.
(15) Select ‘define new transformation’
(16) Select ‘transformation caused by binding’.
(17) Select Galpha_bnd to be the product (in the ‘to’ list) of the transformation.

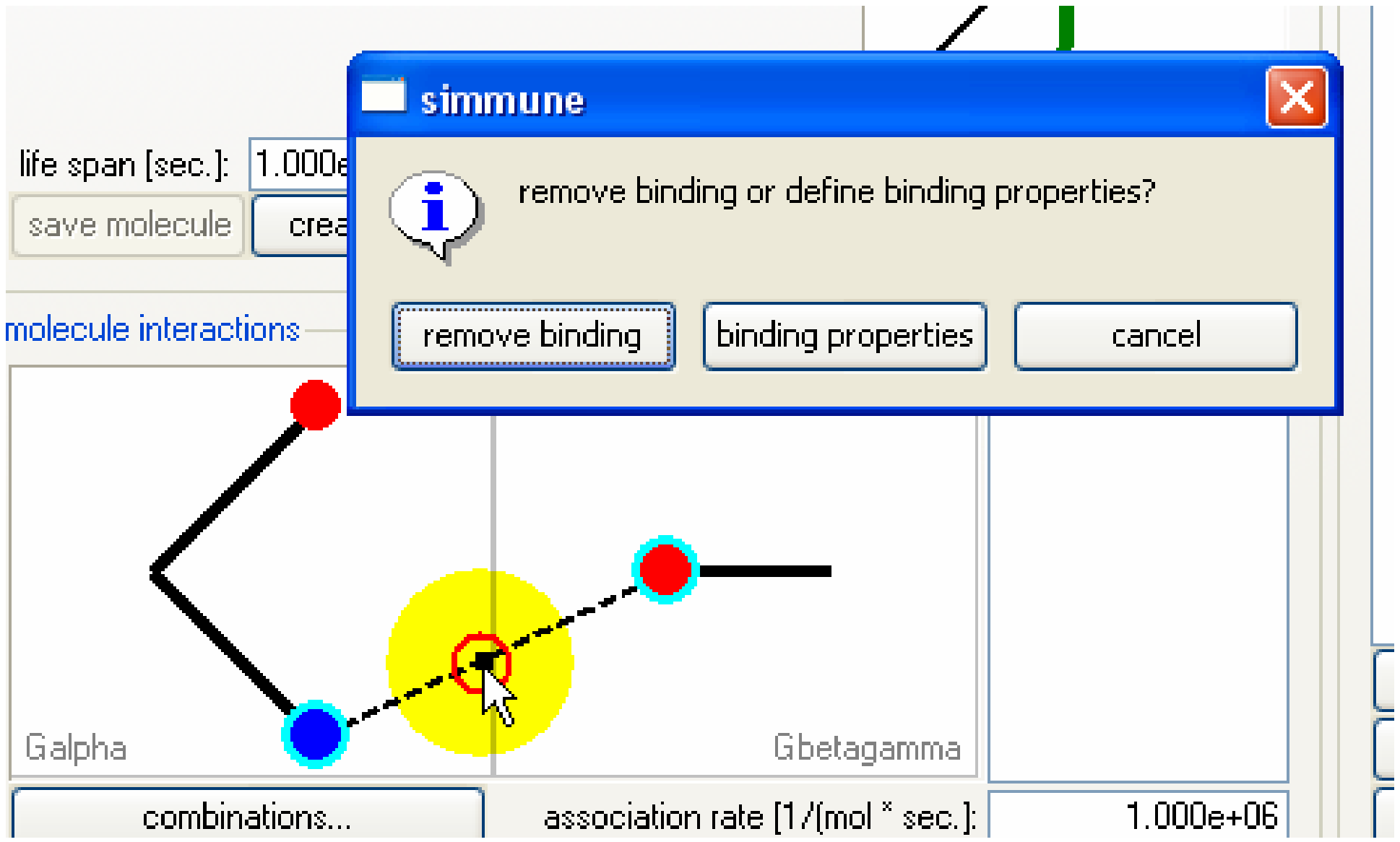

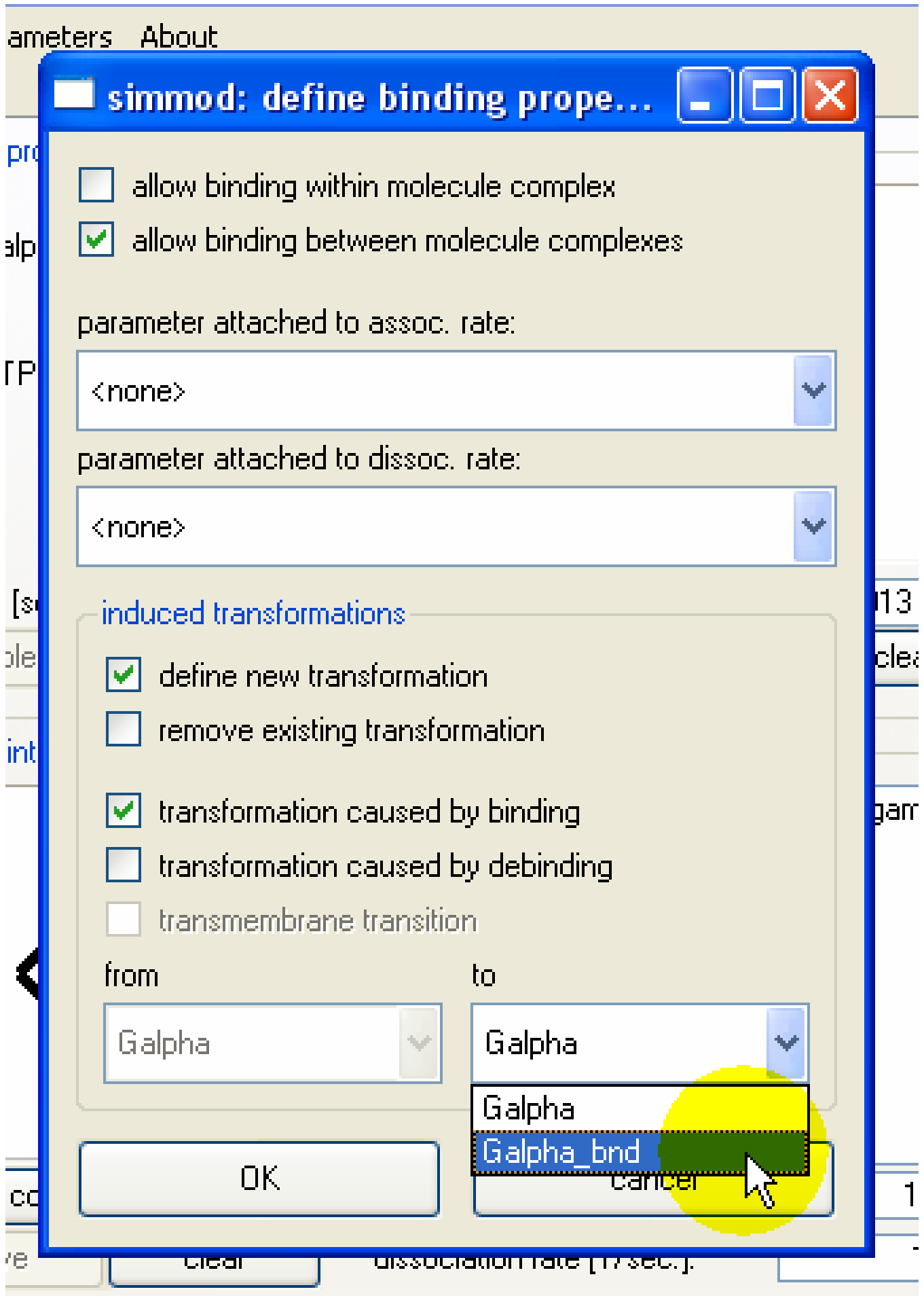

(18) Clear the molecule interactions field.

(19) Drag Galpha_bnd into left area of the molecule interactions field and Gbetagamma into the right area.

(20) Double-click the link between Galpha_bnd and Gbetagamma and then ‘binding properties’ in the small window that appears.

(21) Select ‘define new transformation’

(22) Select ‘transformation caused by **debinding**’.

(23) Select Galpha to be the product (in the ‘to’ list) of the transformation.

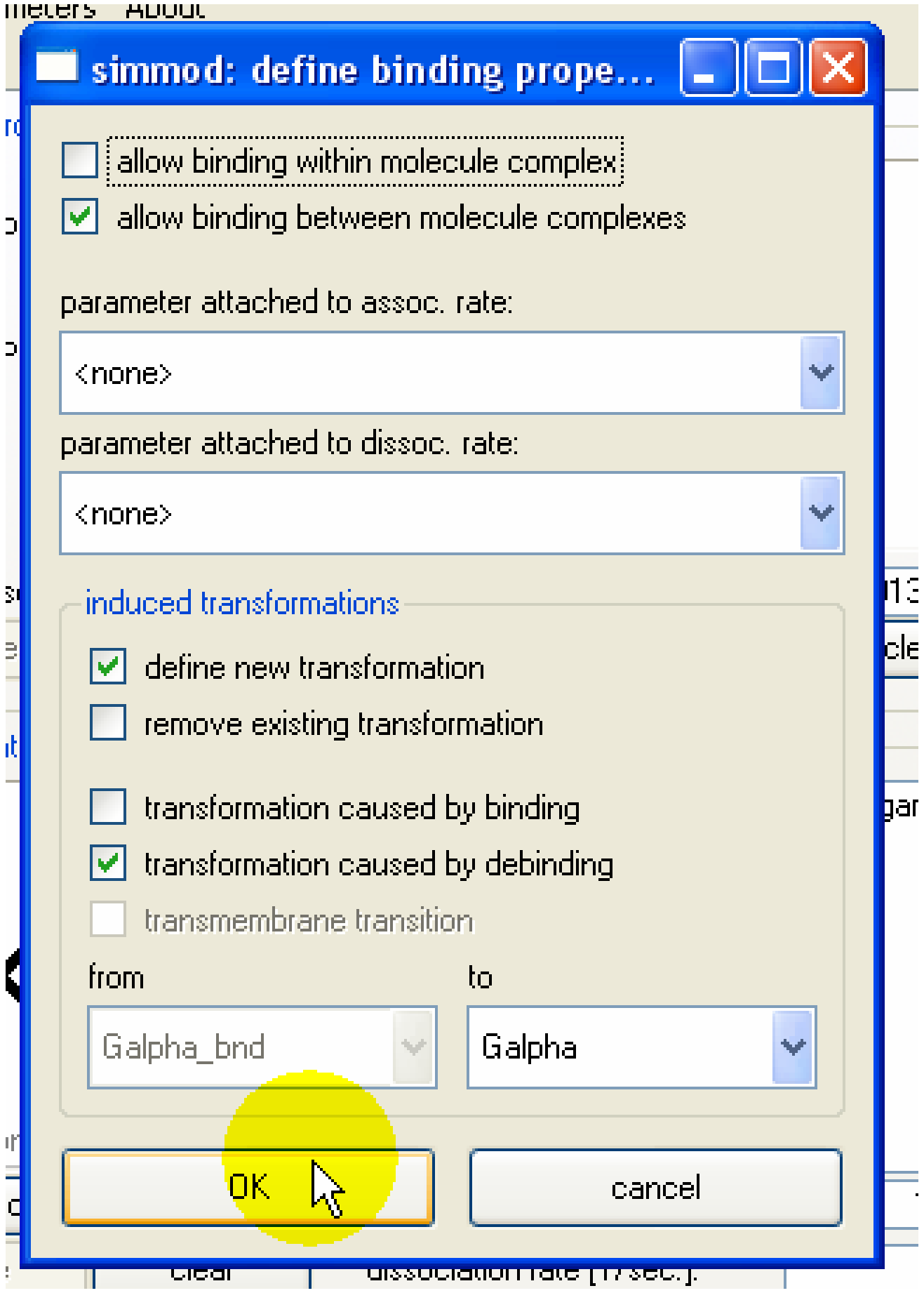

(24) Finally, click the ‘binding table’ button to inspect the molecular interactions and binding- or debinding induced transformations we have defined so far.

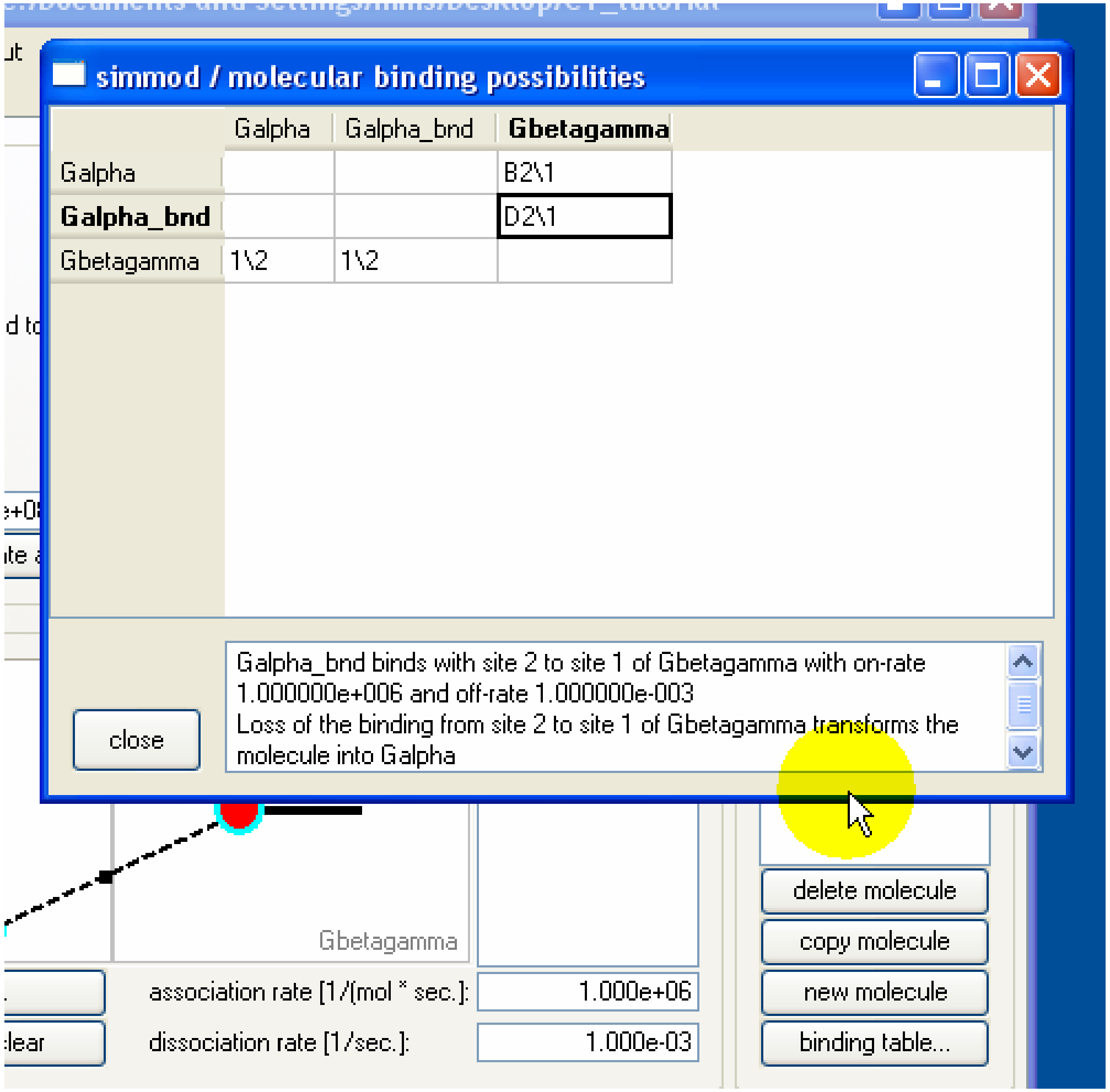

Supplement: Supplementary file 8 [file pcbi.0020082.sd008.pdf]
