# Supplementary material for: Key Role of Local Regulation in Chemosensing Revealed by a New Molecular Interaction-Based Modeling Method
Source: PLoS Comput Biol. 2006 Jul 21;2(7):e82. doi: 10.1371/journal.pcbi.0020082 (PMC1513273; doi:10.1371/journal.pcbi.0020082)

Simmune tutorial 2

Creating transmembrane receptors
Defining transmembrane signaling events

(1) Load the CT_tutorial model from Tutorial 1.

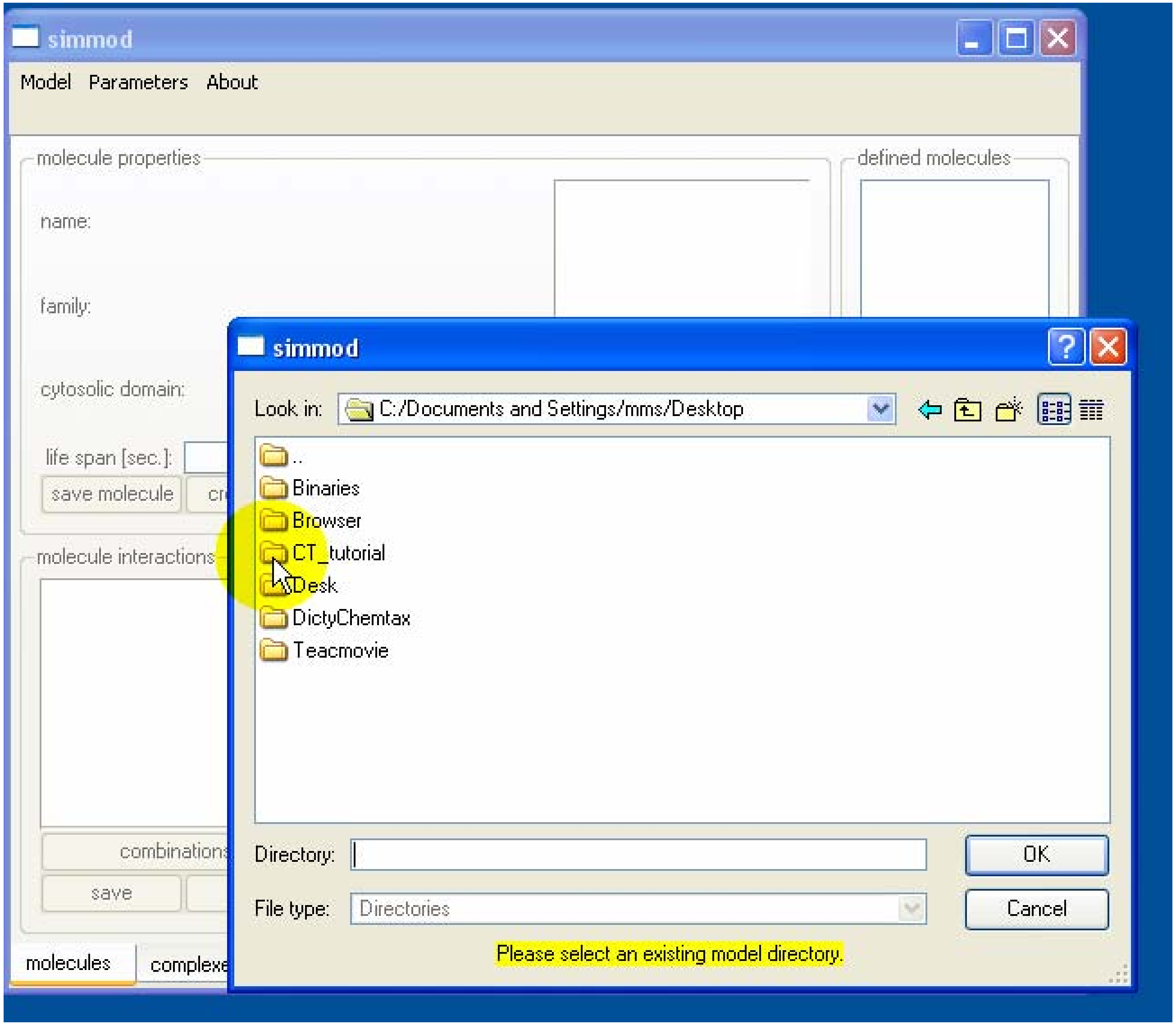

(2) Create the cAMP molecule and the **extracellular** part of the cAMP receptor.

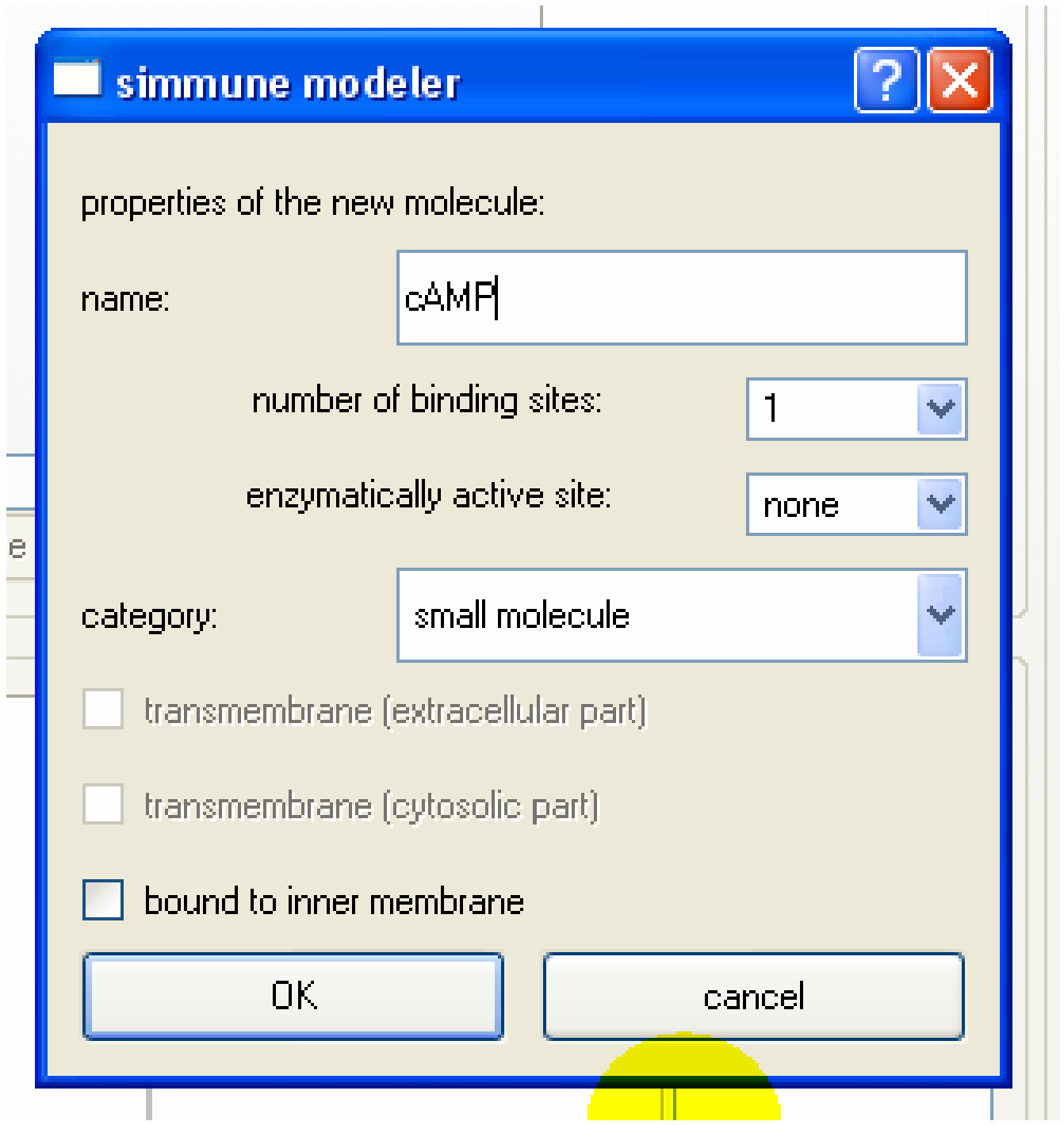

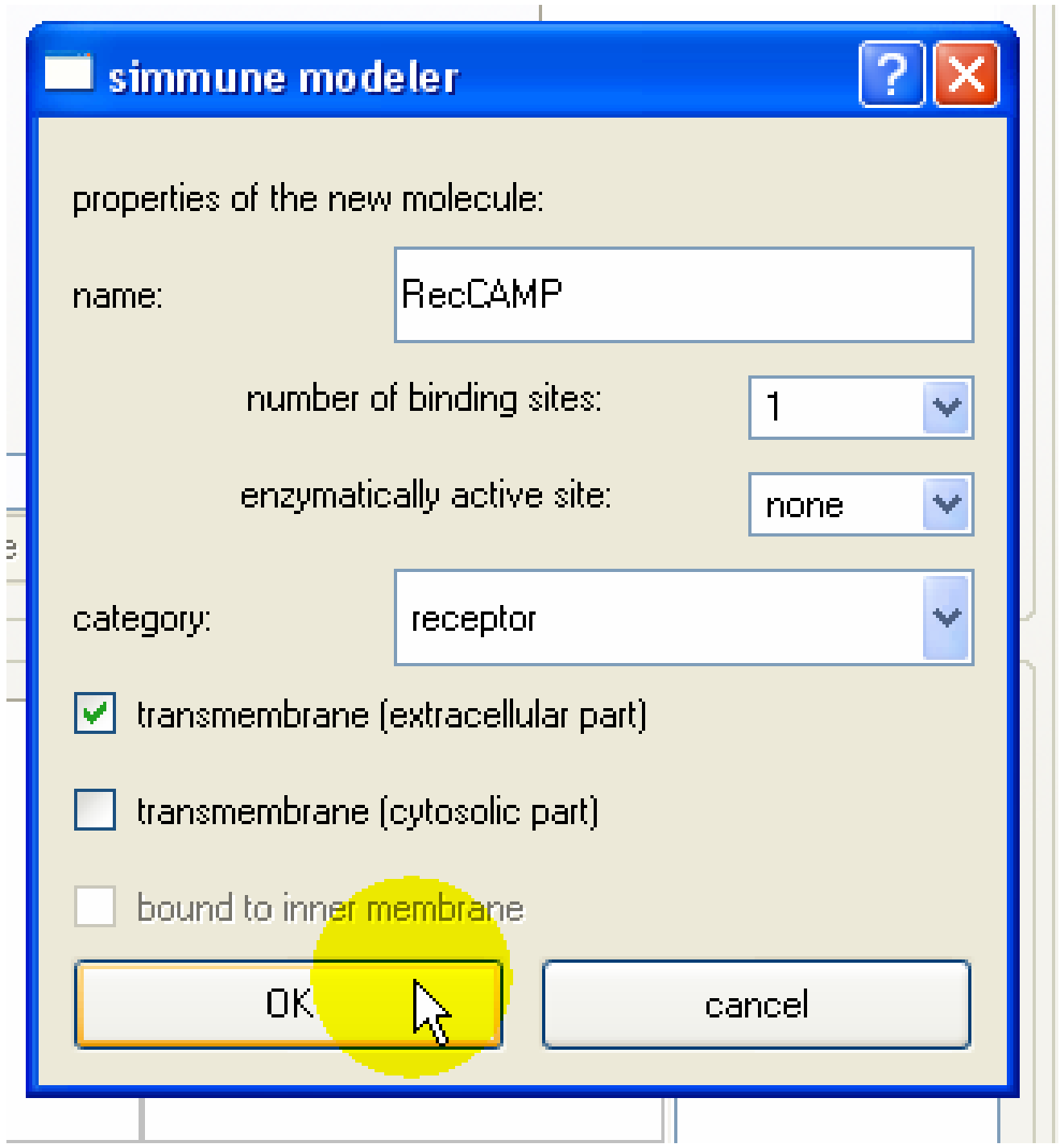

(3) Define the binding property between the cAMP receptor (RecCAMP) and cAMP.

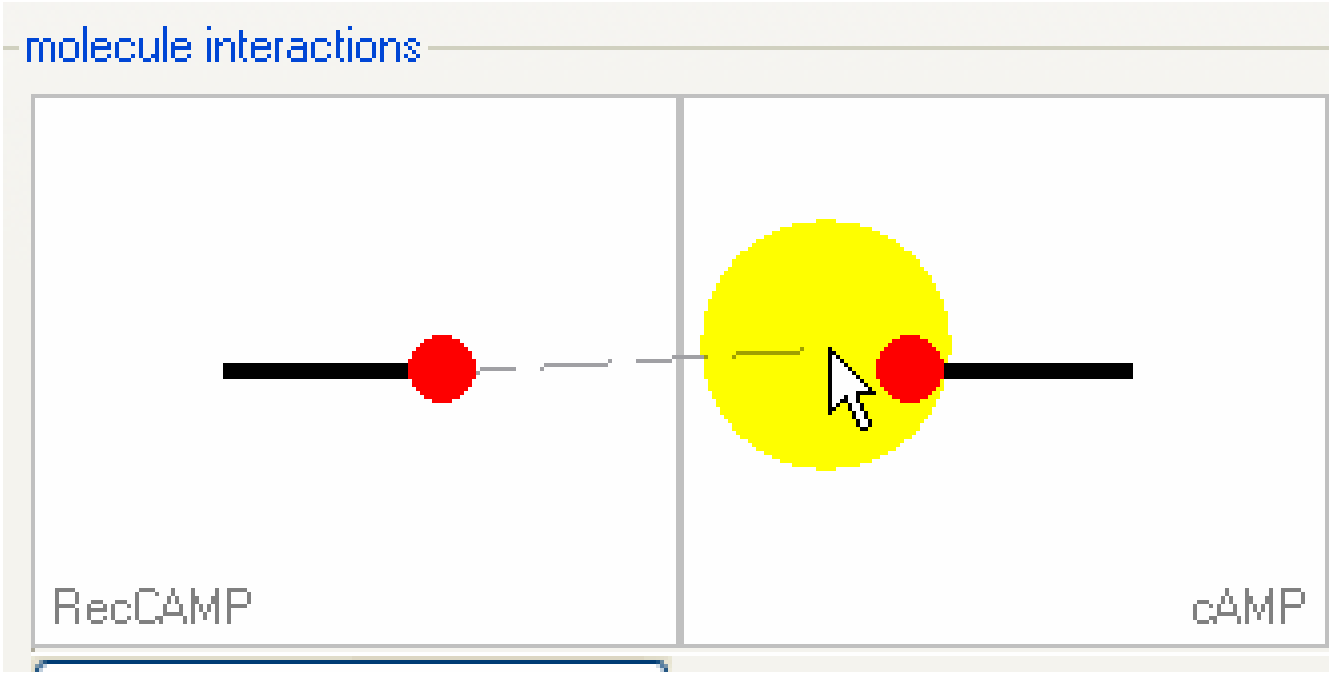

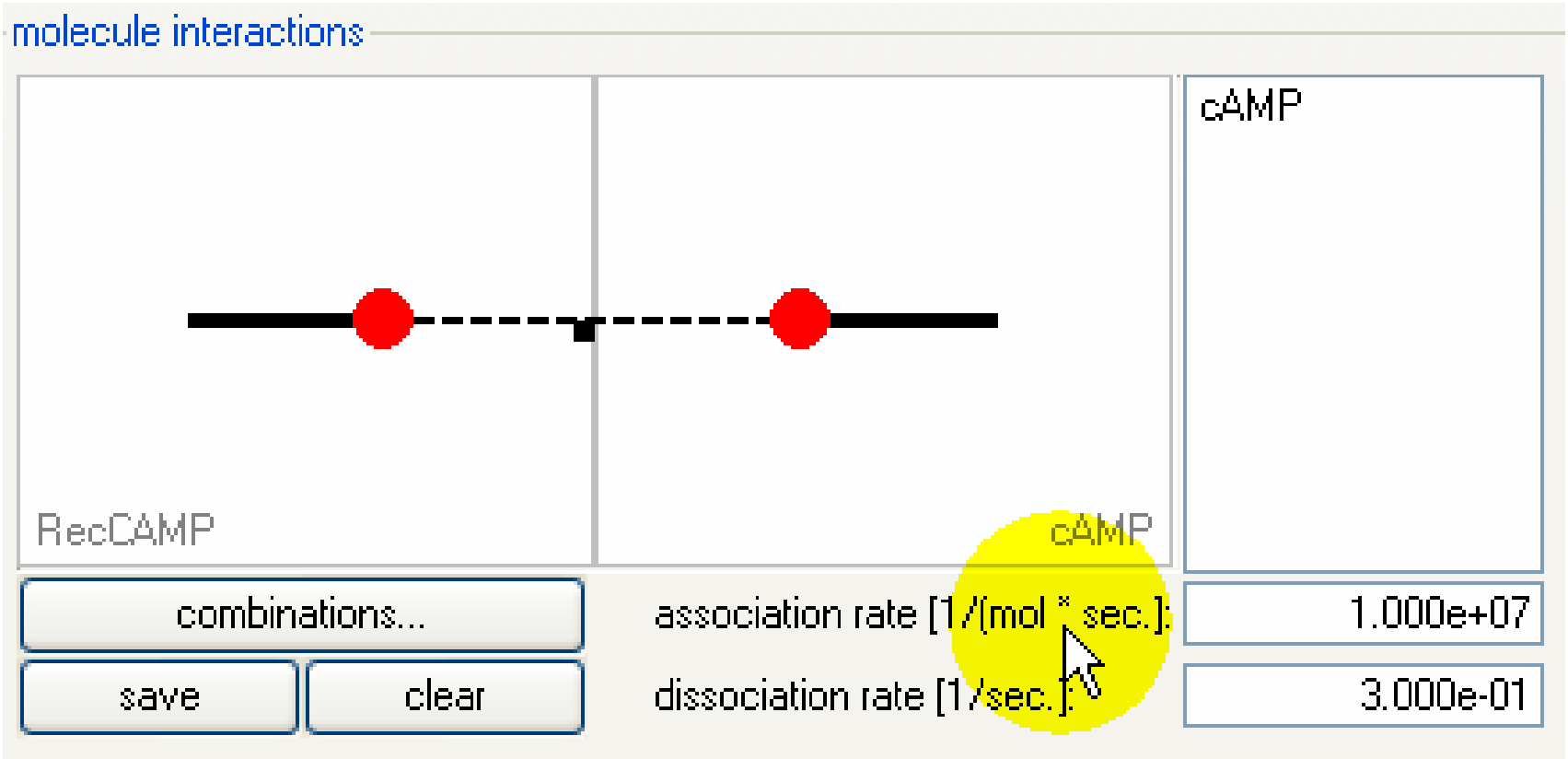

(4) Define the diffusion coefficient of the cAMP receptor.

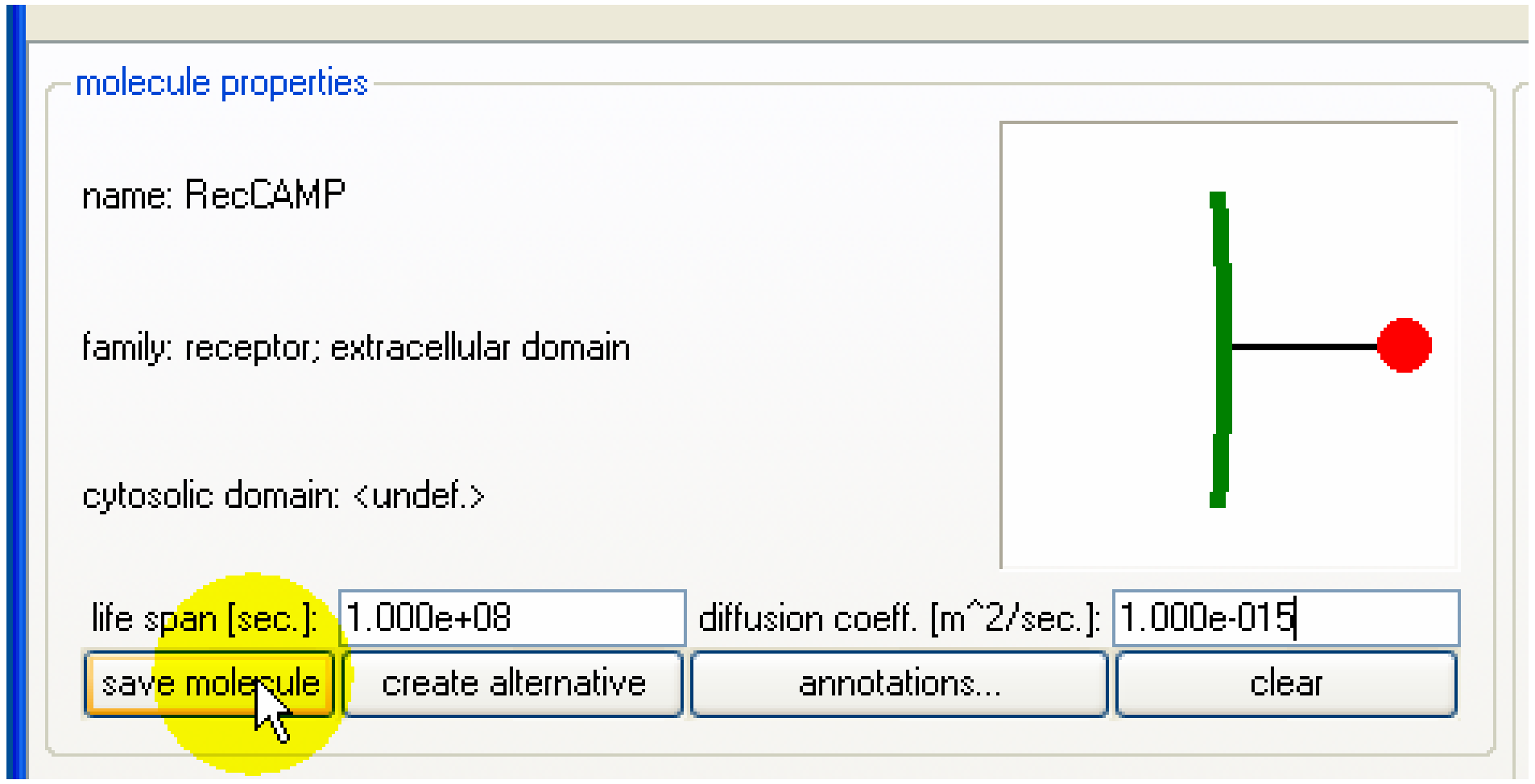

(5) Create the cytosolic part of the cAMP recptor.
(6) Drag it onto the RecCAMP symbol in the ‘molecule properties’ field to define that RecCAMPcyto should be the cytosolic domain of RecCAMP.

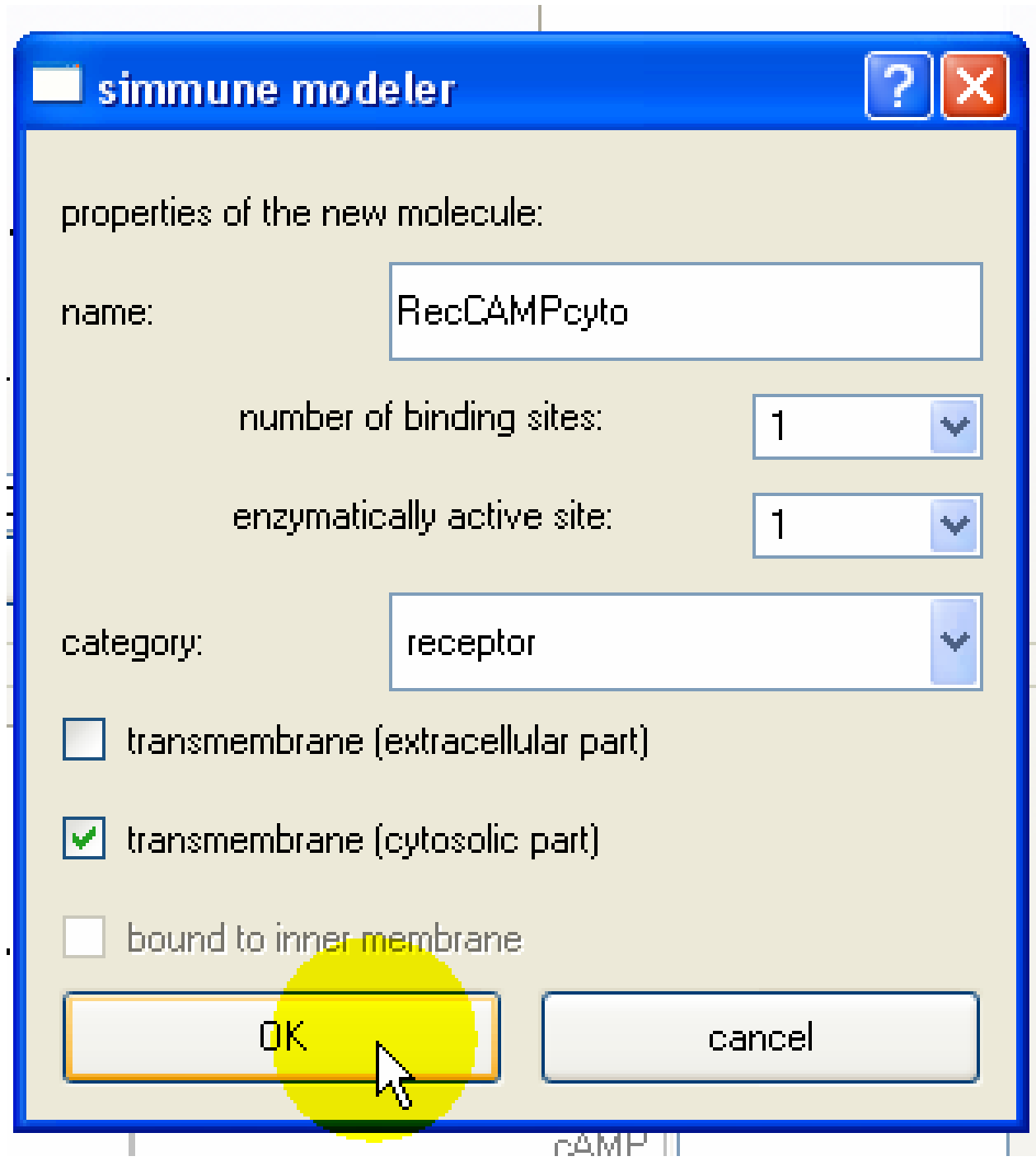

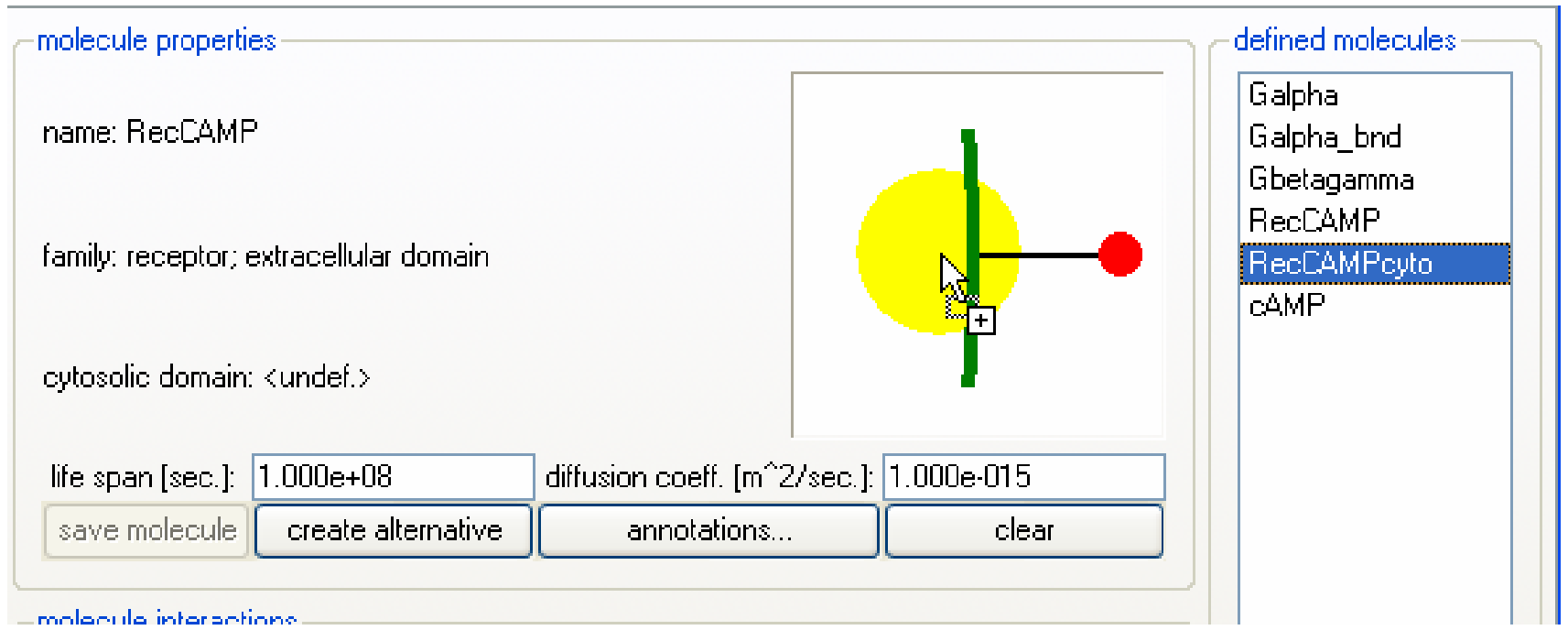

(7) Select RecCAMPcyto (by double-clicking it in the 'defined molecules' list.
(8) Create an alternative to it, called 'RecCAMPcyto_act'.

RecCAMPcyto_act inherits everything from RecCAMPcyto, including the property of being a cytosolic part for the cAMP receptor, RecCAMP.

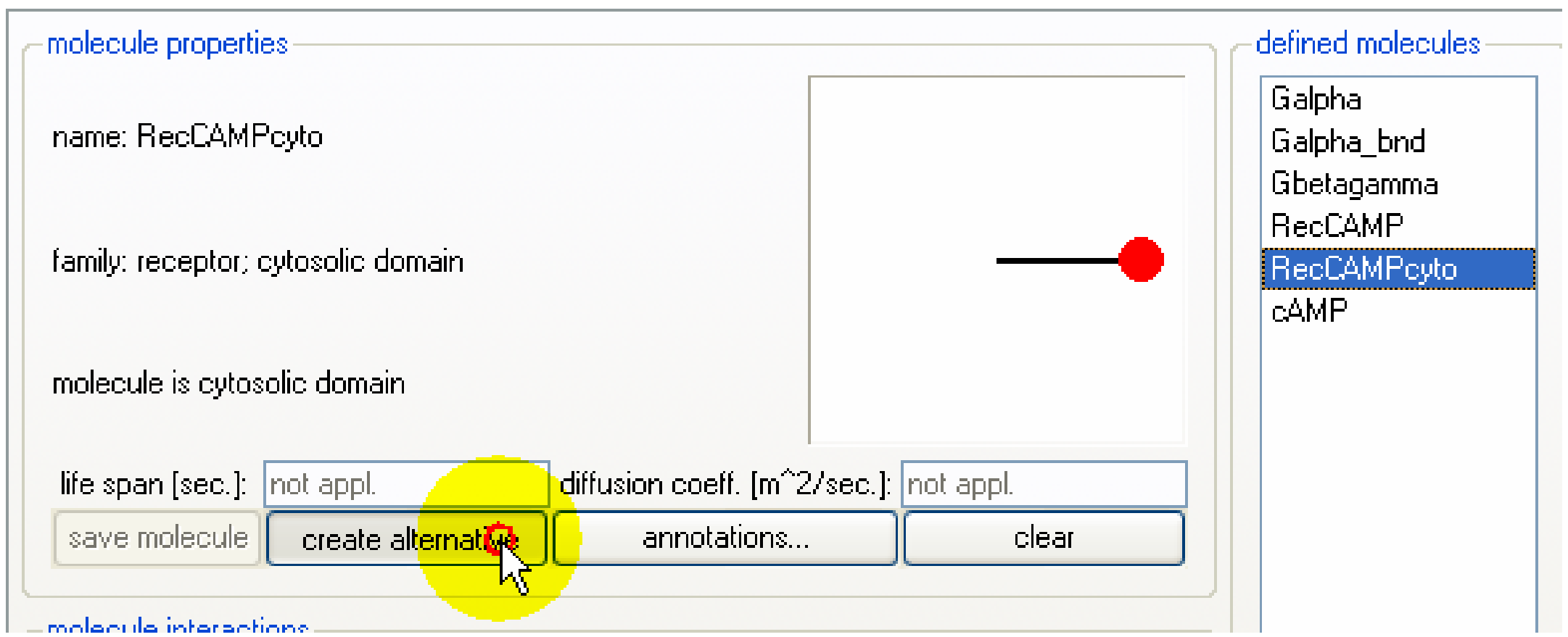

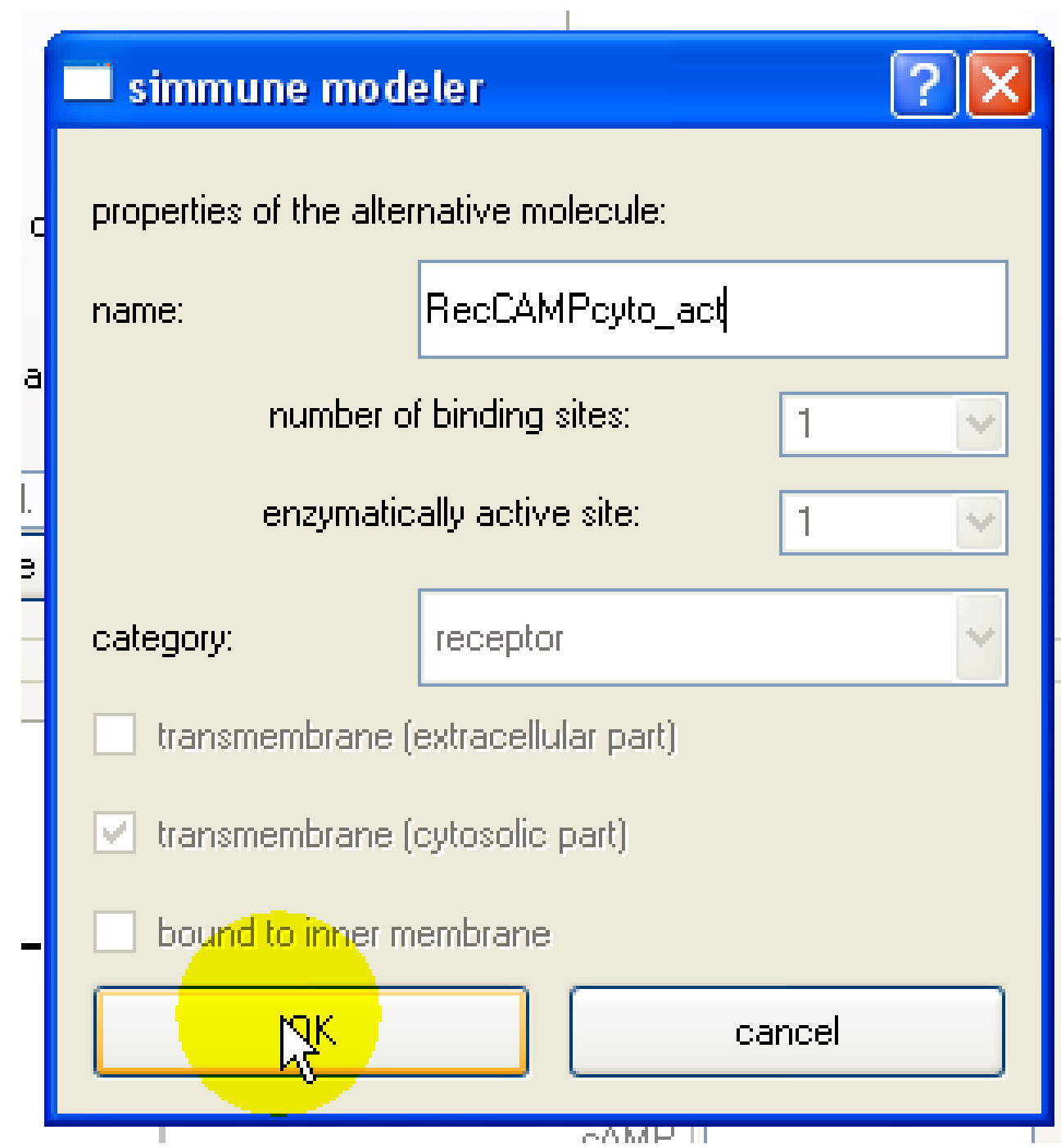

(9) Double-click the link between RecCAMP and cAMP to associate a Binding-induced transformation to the binding between RecCAMP and cAMP.
(10) Important: this time we define a **‘transmembrane transformation’**, leading from RecCAMPcyto to RecCAMPcyto_act.

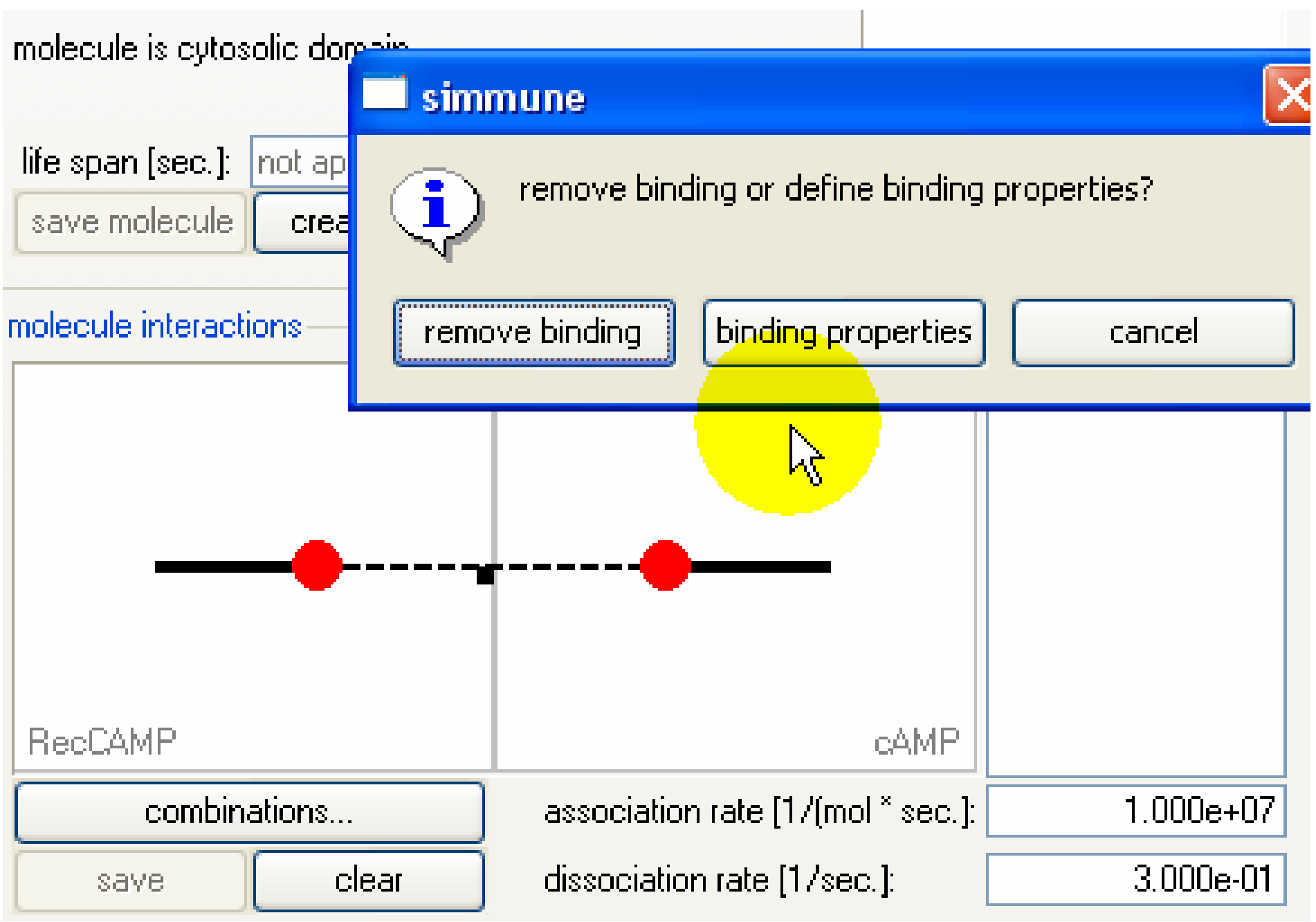

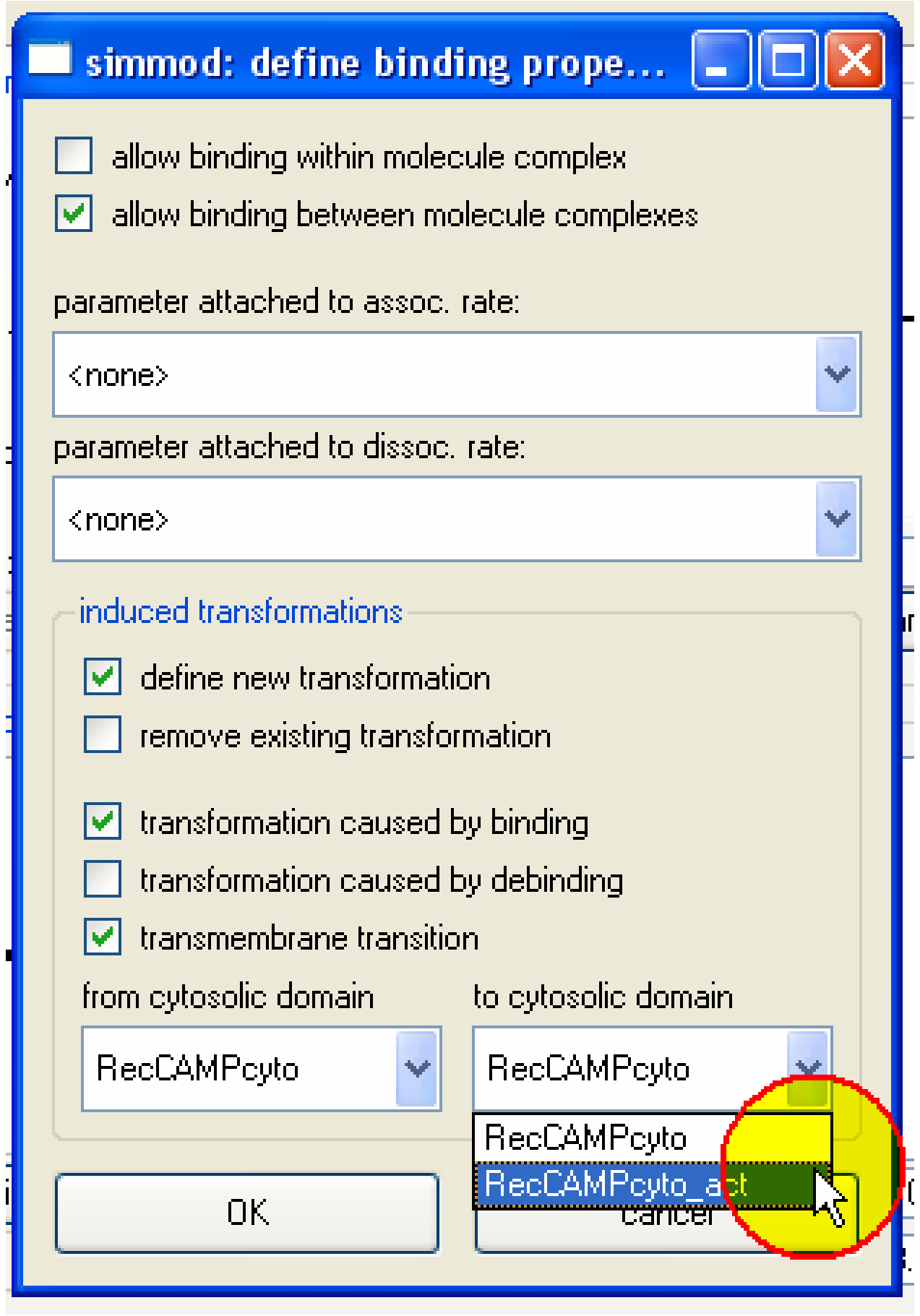

(11) Similarly, we link a **de**binding-induced transformation to the debinding event between RecCAMP and cAMP.
(12) We define a **‘transmembrane transformation’**, leading from RecCAMPcyto_act to RecCAMPcyto.

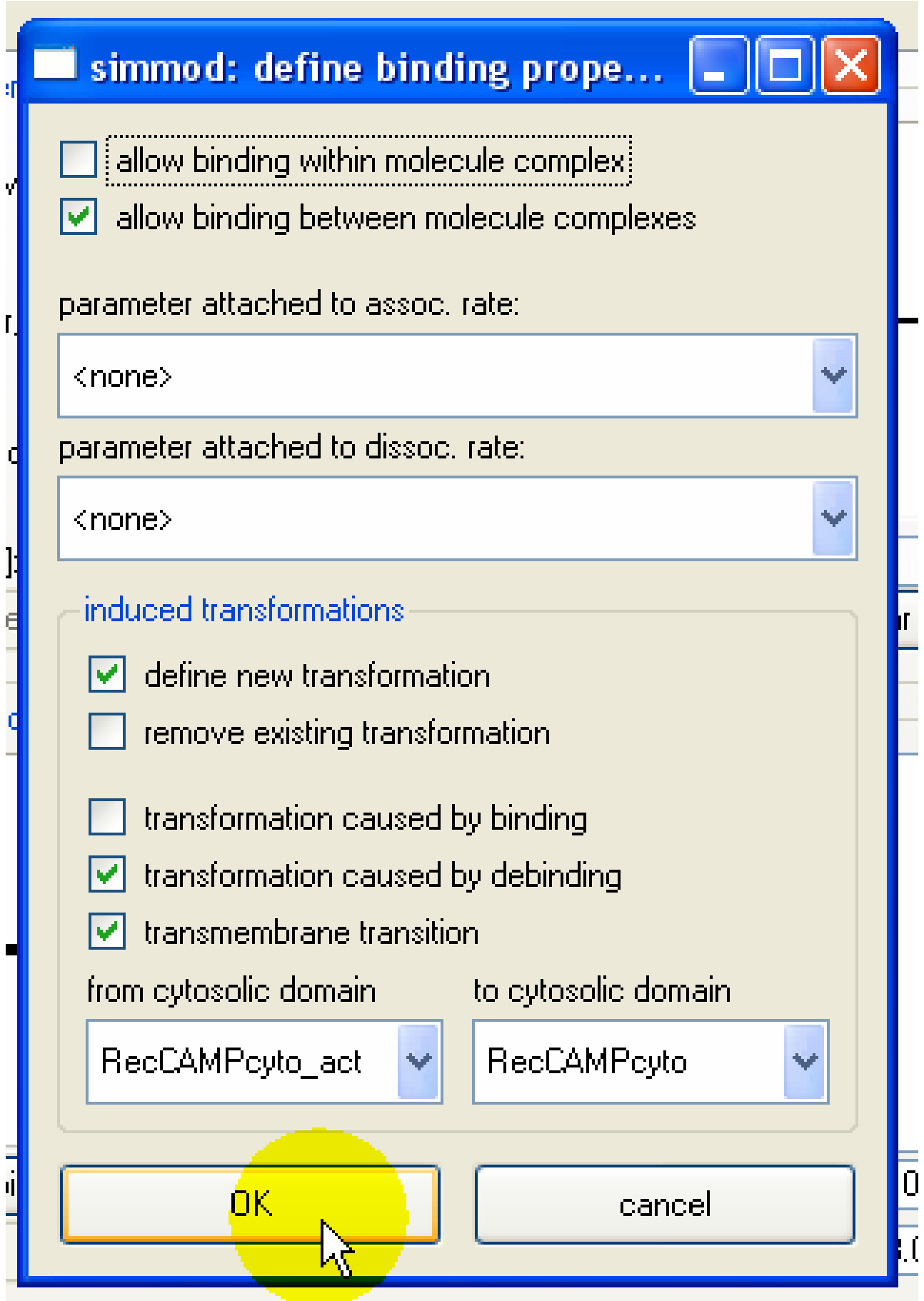

Supplement: Supplementary file 9 [file pcbi.0020082.sd009.pdf]
