# Supplementary material for: Key Role of Local Regulation in Chemosensing Revealed by a New Molecular Interaction-Based Modeling Method
Source: PLoS Comput Biol. 2006 Jul 21;2(7):e82. doi: 10.1371/journal.pcbi.0020082 (PMC1513273; doi:10.1371/journal.pcbi.0020082)

Simmune tutorial 3

Creating molecular complexes
Defining enzymatic transformations

(1) Load the CT_tutorial model from Tutorial 2.

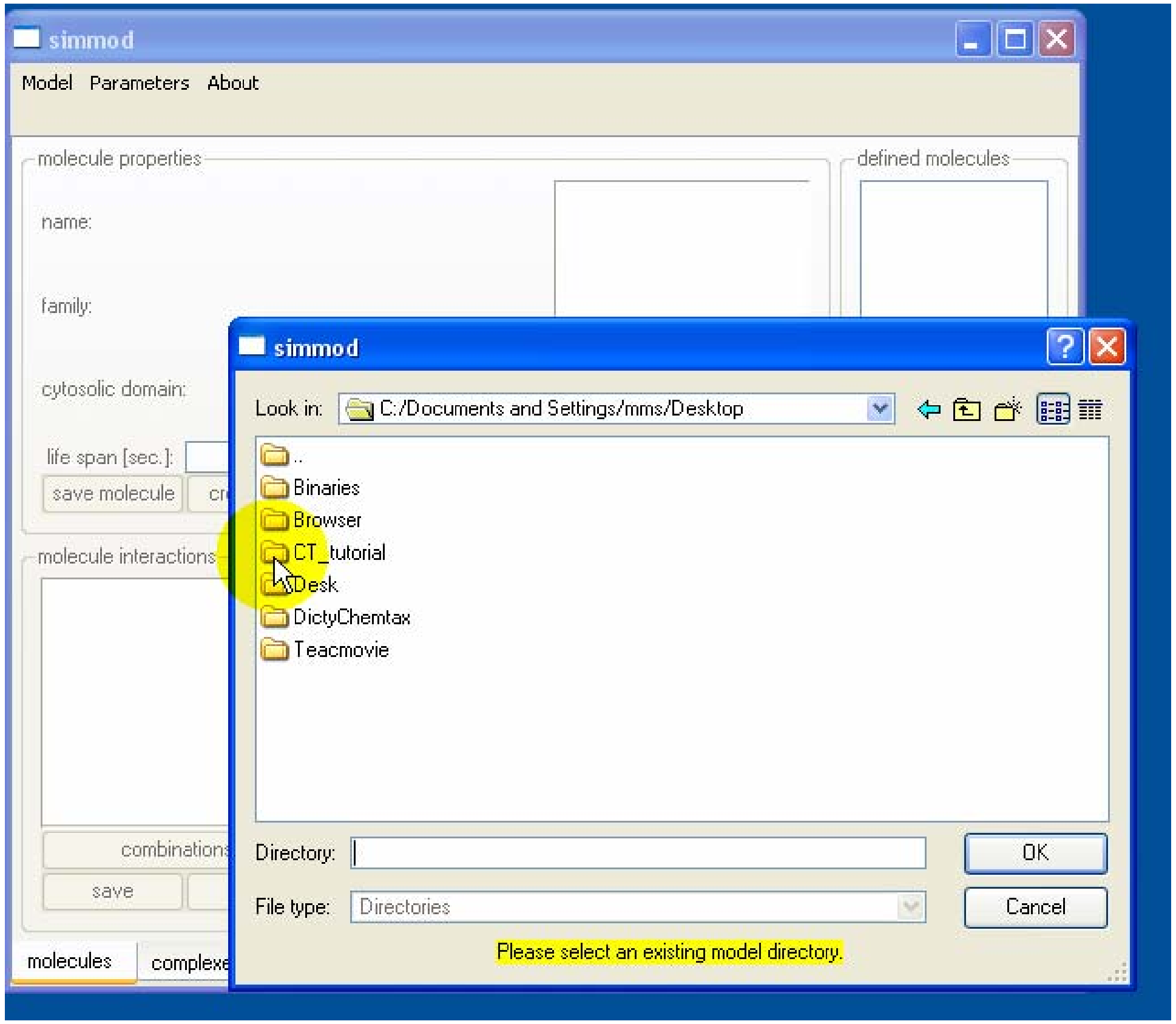

(2) Go to the 'complexes' tab.

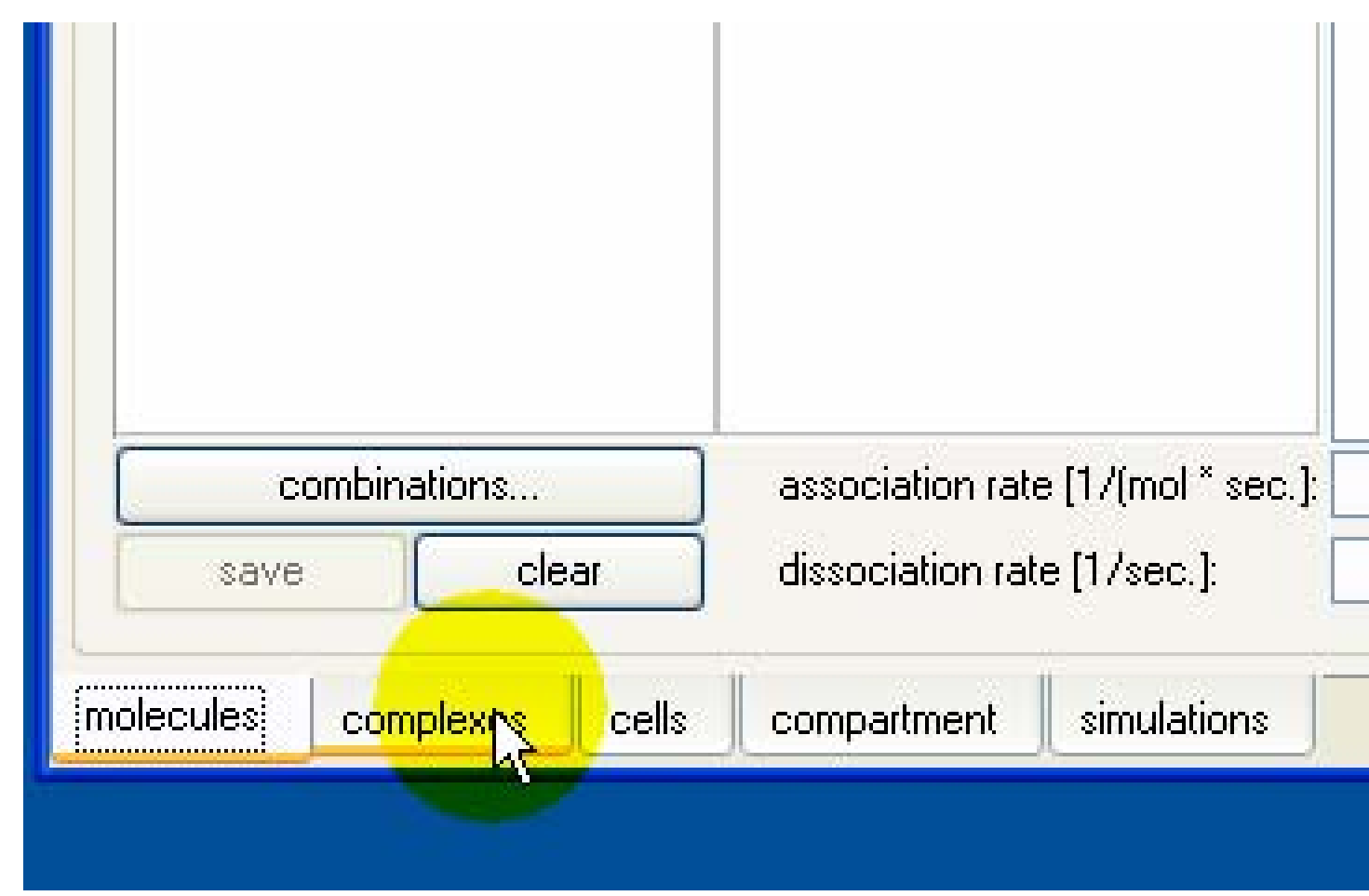

(3) To start building the transmembrane receptor drag the extracellular Part of the cAMP receptor into the ‘complex definition’ field.

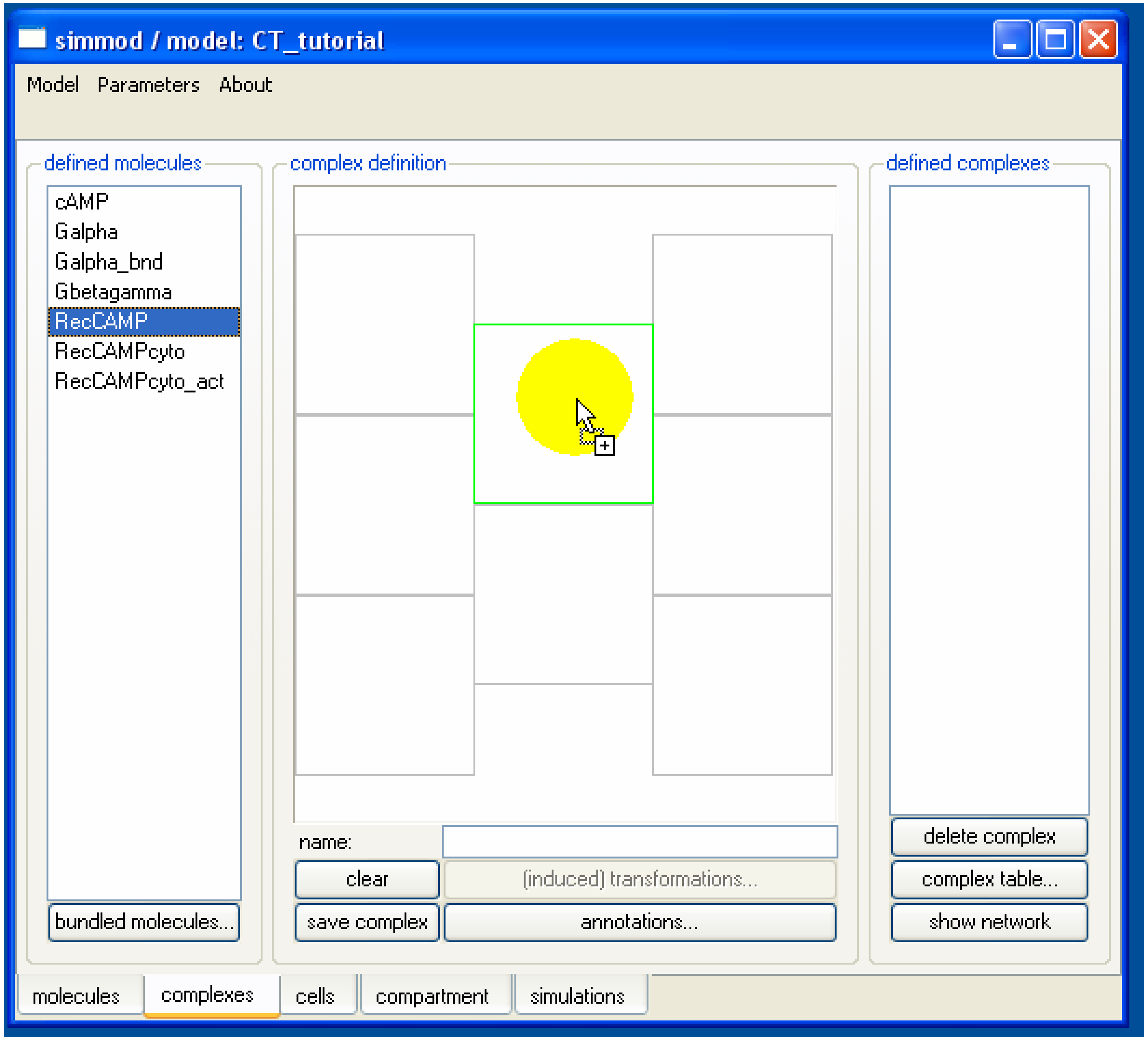

(4) Drag and drop the intracellular part of the receptor onto the extracellular part of the cAMP receptor in the ‘complex definition’ field.

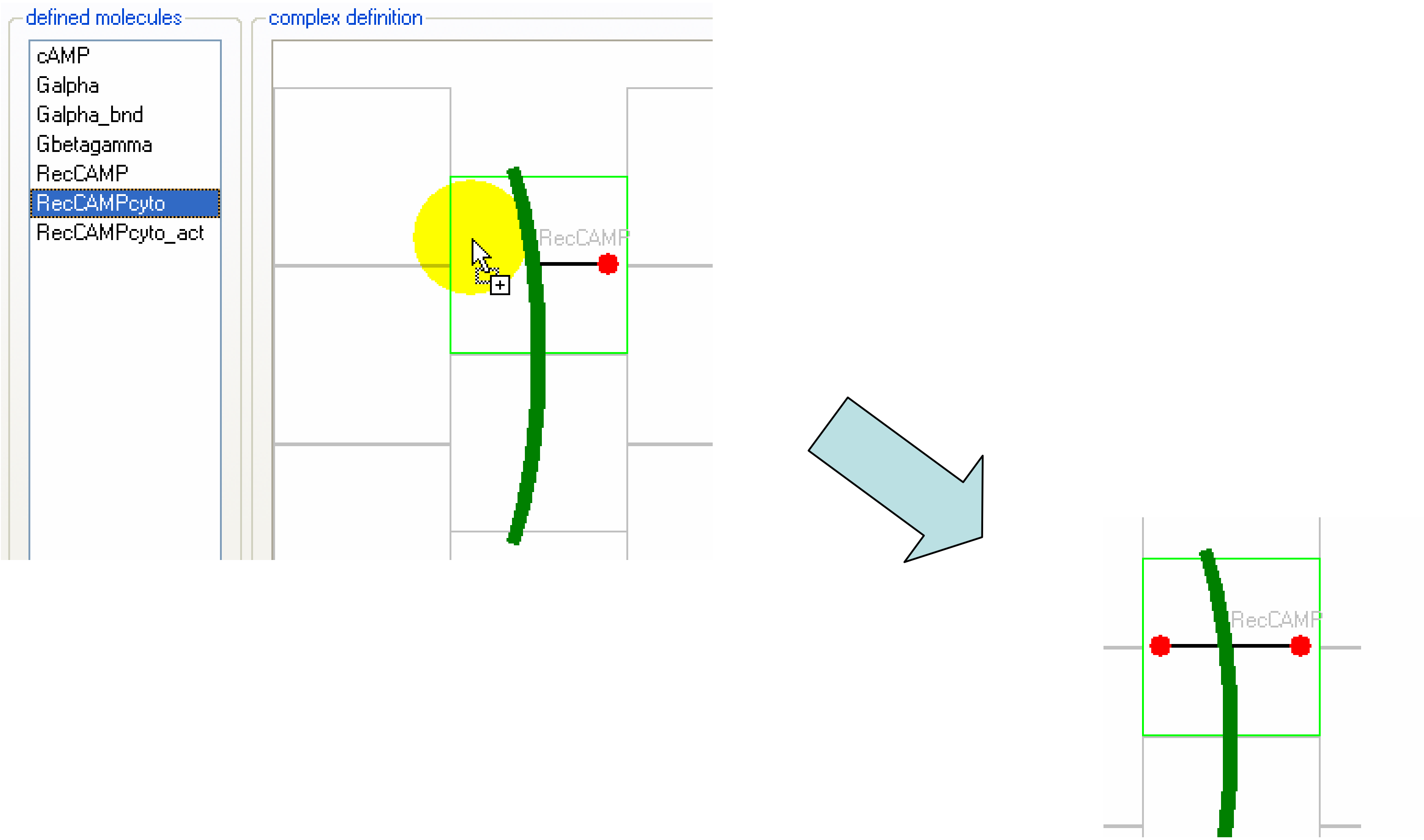

Simmod will show the complete transmembrane receptor.

(5) Save the transmembrane receptor as 'RecCAMP'. It is OK for the complete receptor complex to have the same name as the extracellular domain.

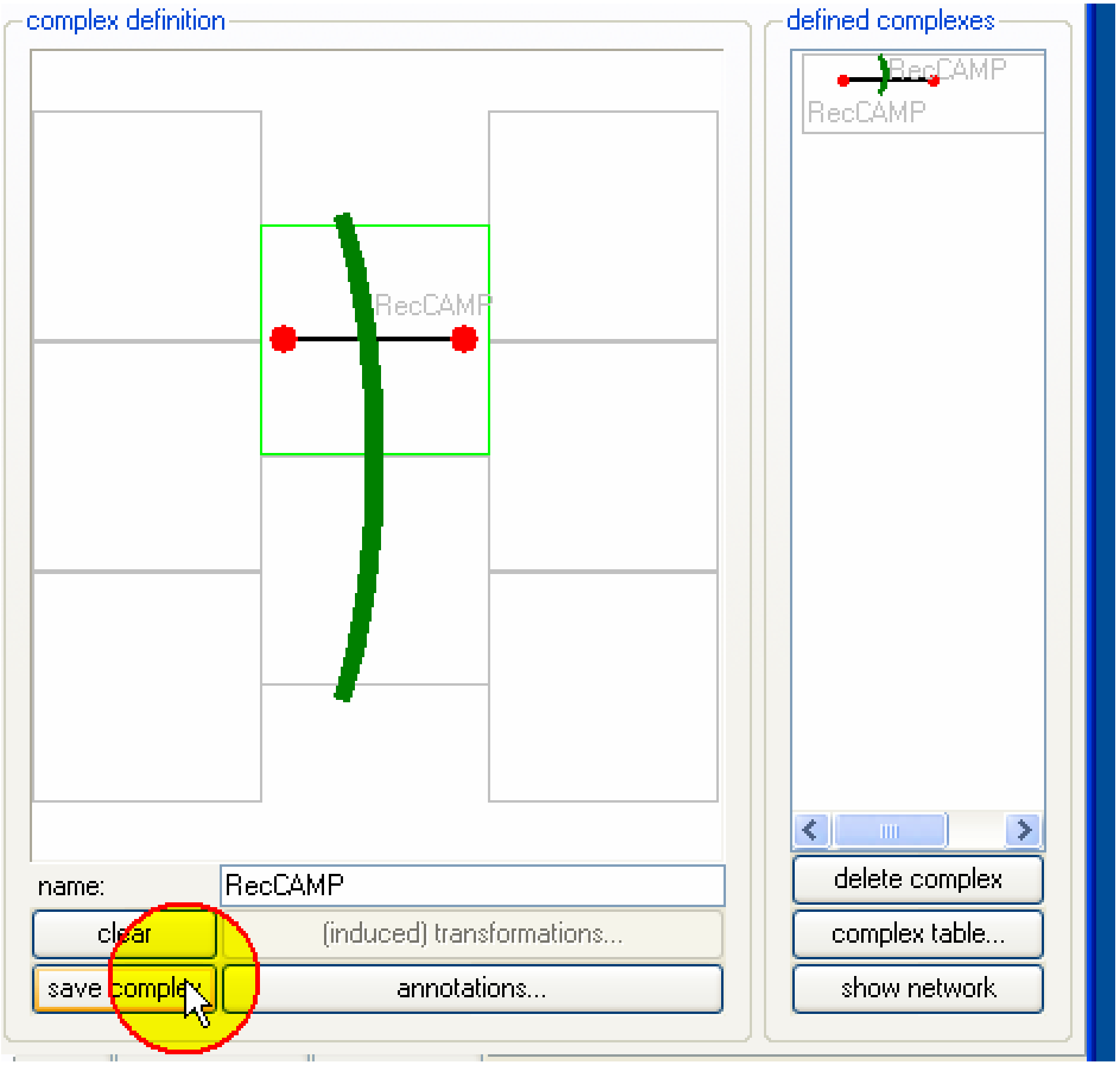

(6) Perform the same actions to create the activated receptor with the RecCAMPcyto_act intracellular domain.

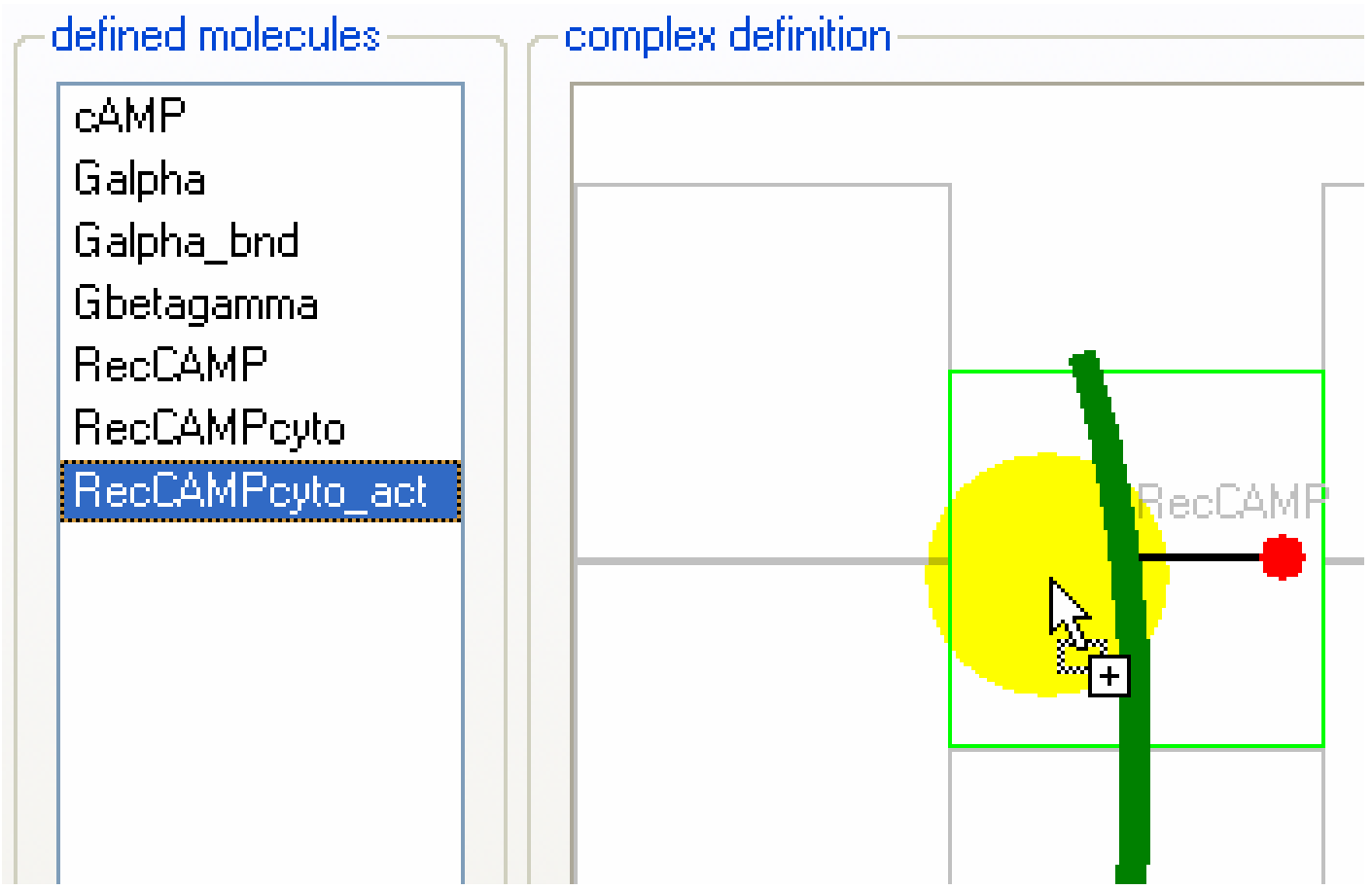

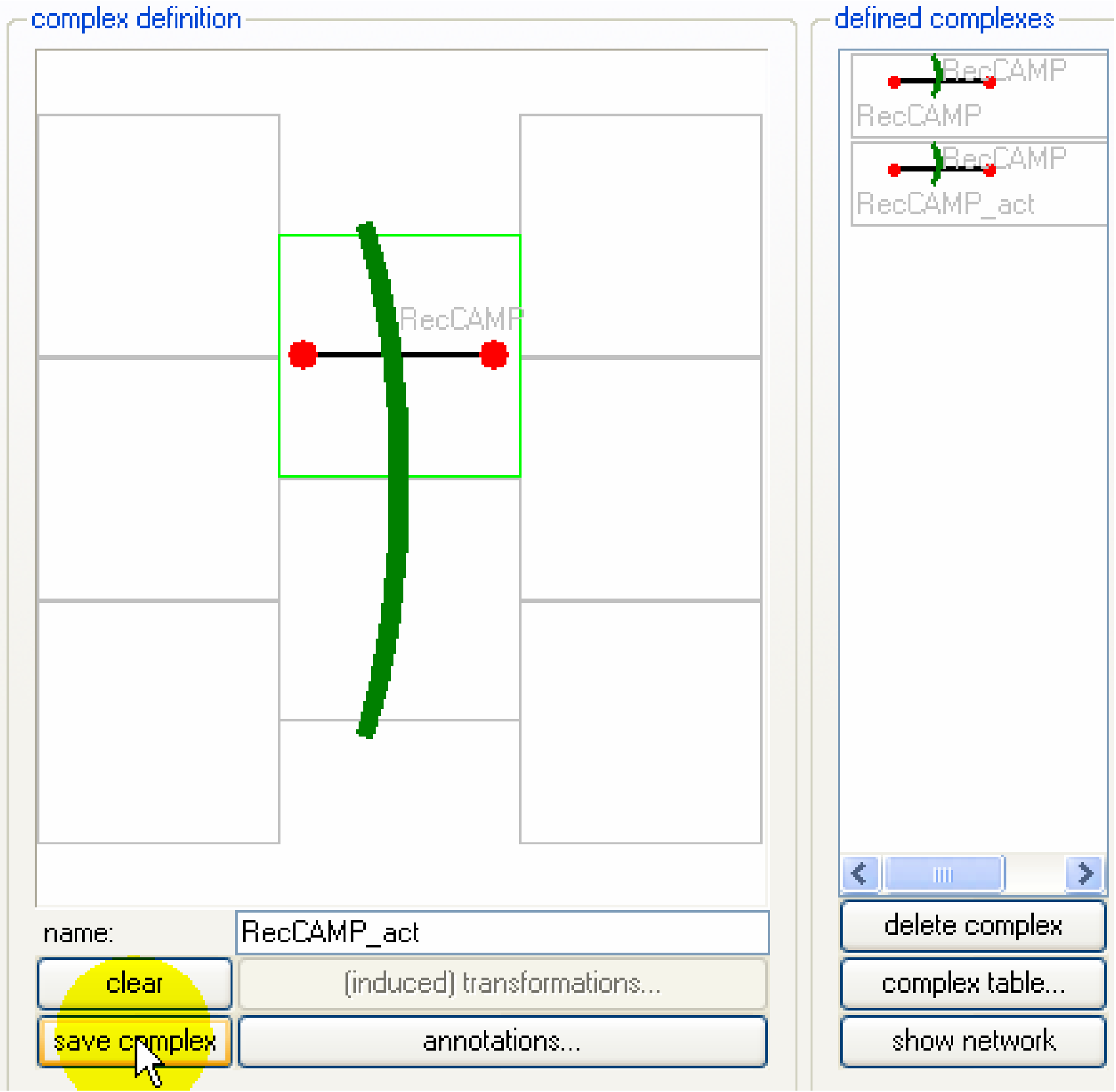

Save the receptor complex as ‘RecCAMP_act’.

(7) Drag and drop Galpha_bnd and Gbetagamma molecules into the ‘complex definition’ field as shown below.

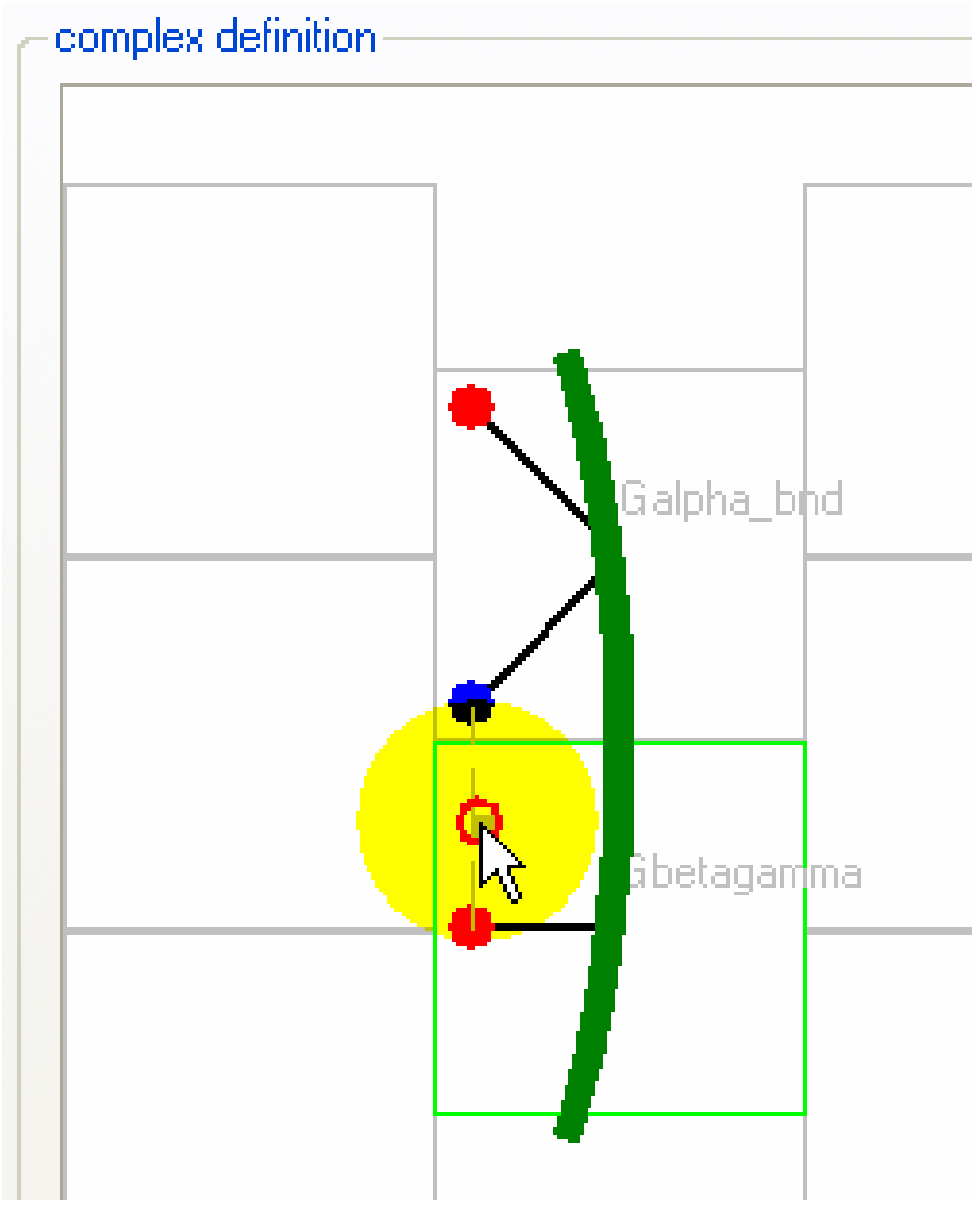

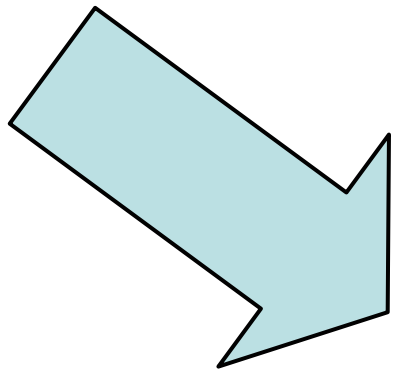

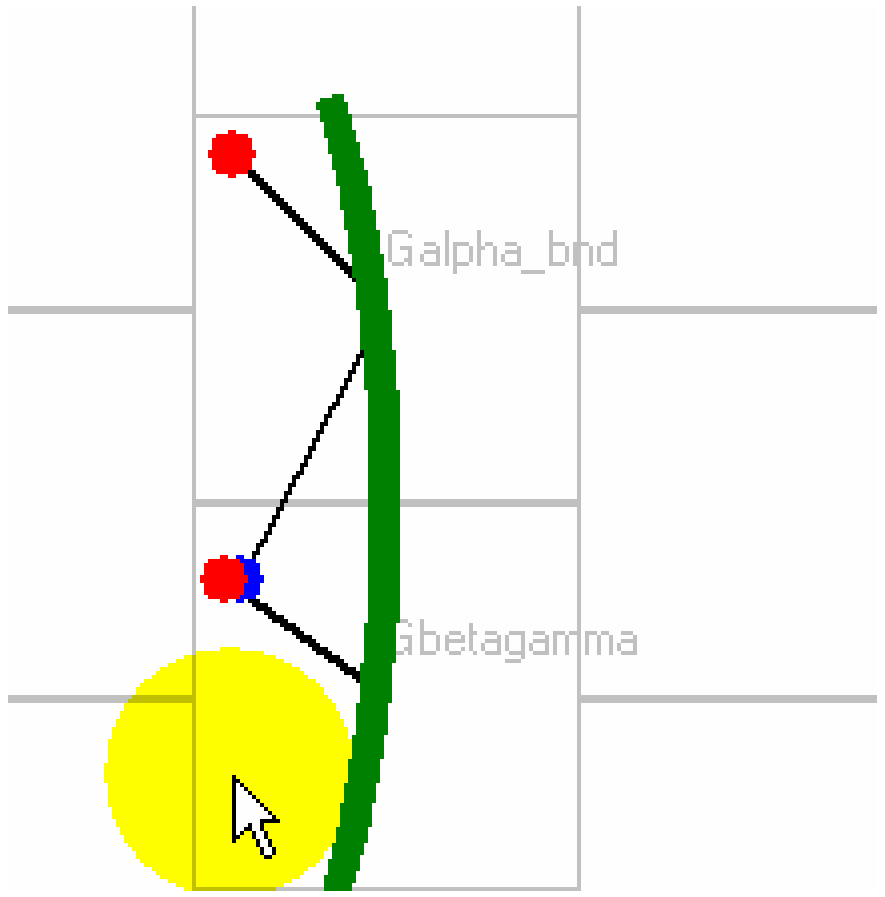

(8) Double-click the small rectangle indicating a binding possibility to create the complex.
Save it as ‘Gabg’ (short for Galphabetagamma).

(9) Go back to the ‘molecules’ tab, drag the Galpha molecule into the ‘molecule properties’ field and click ‘create alternative’ to create an alternative state for Galpha.

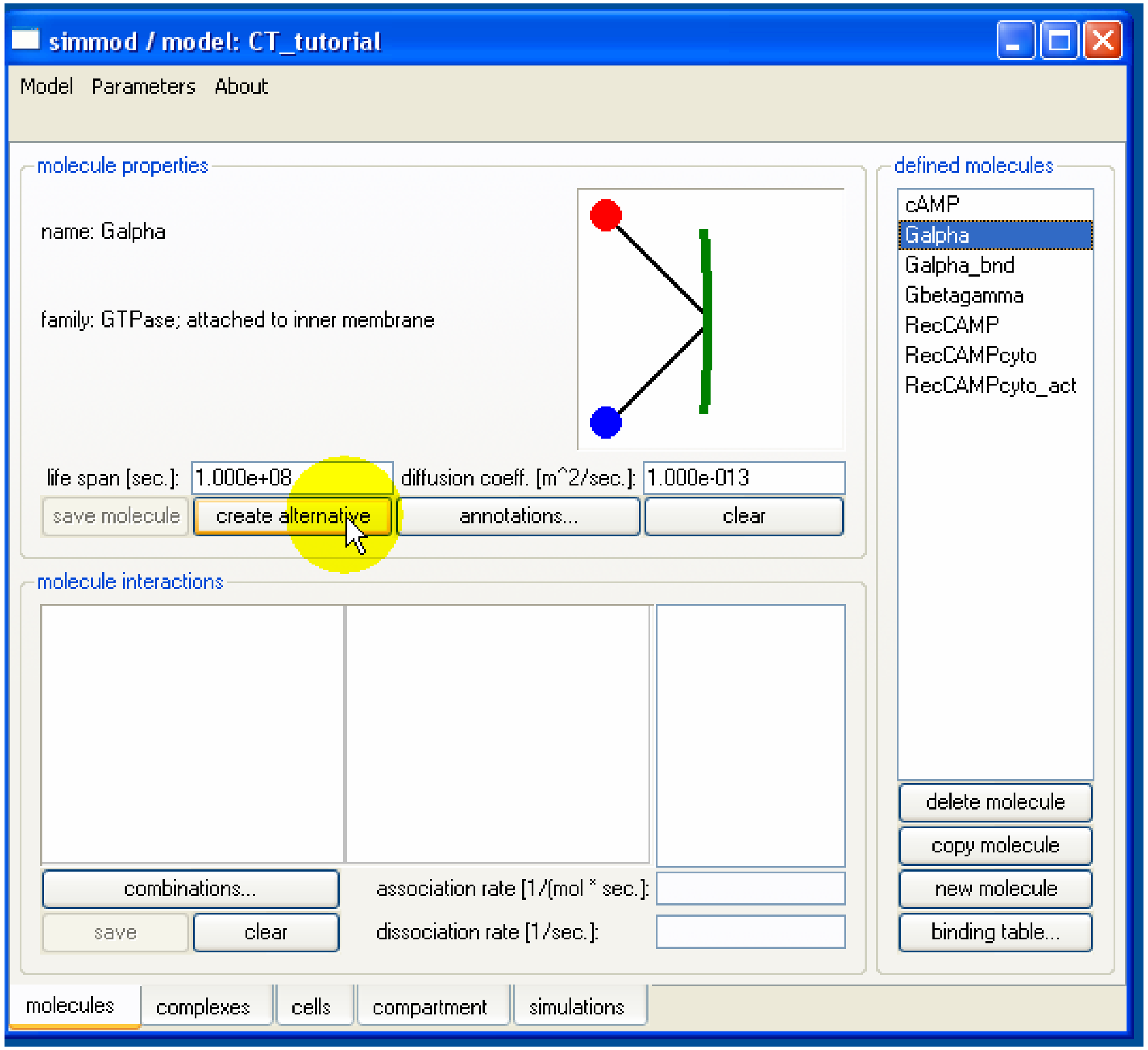

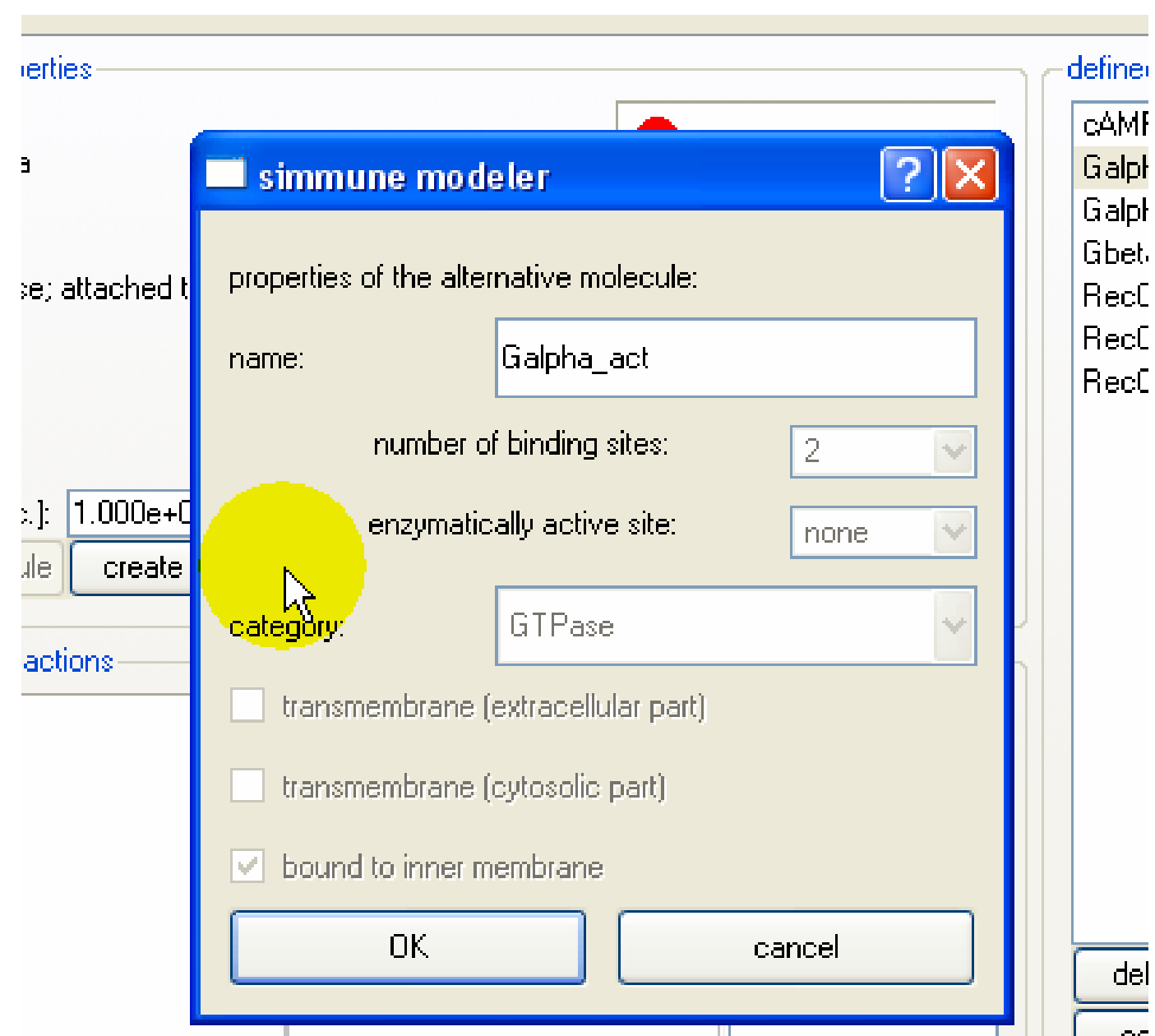

(10) Assign the name ‘Galpha_act’ to the new state.

(11) Similarly, create the ‘Galpha_act_bnd’ alternative state for Galpha_bnd.

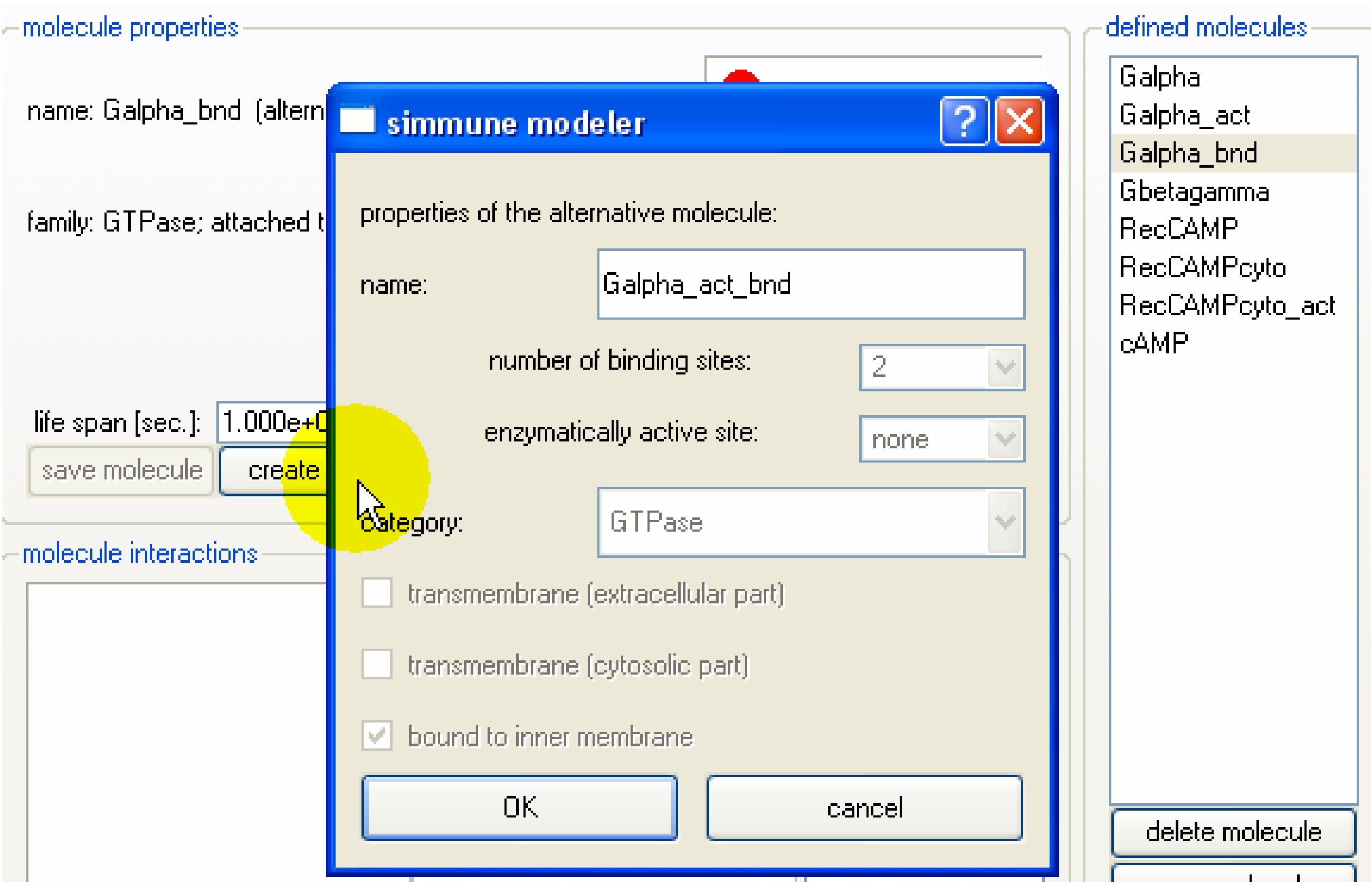

Simmod will remind you that Galpha_bnd is itself an alternative of Galpha and that Galpha_act_bnd will be an alternative of Galpha as well.

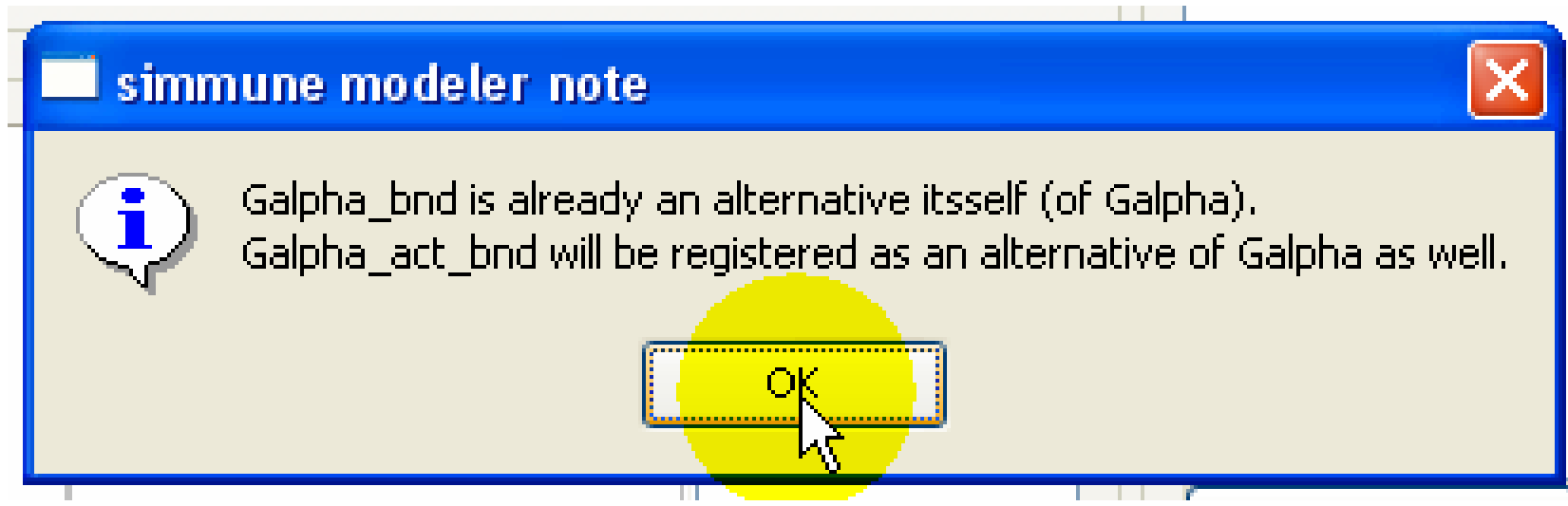

(12) Now, drag and drop RecCAMPcyto_act and Galpha_bnd in the 'molecule interactions' field and draw a connection between the enzymatic (turquoise circled) binding site of RecCAMPcyto_act and the first (red) binding site of Galpha_bnd.

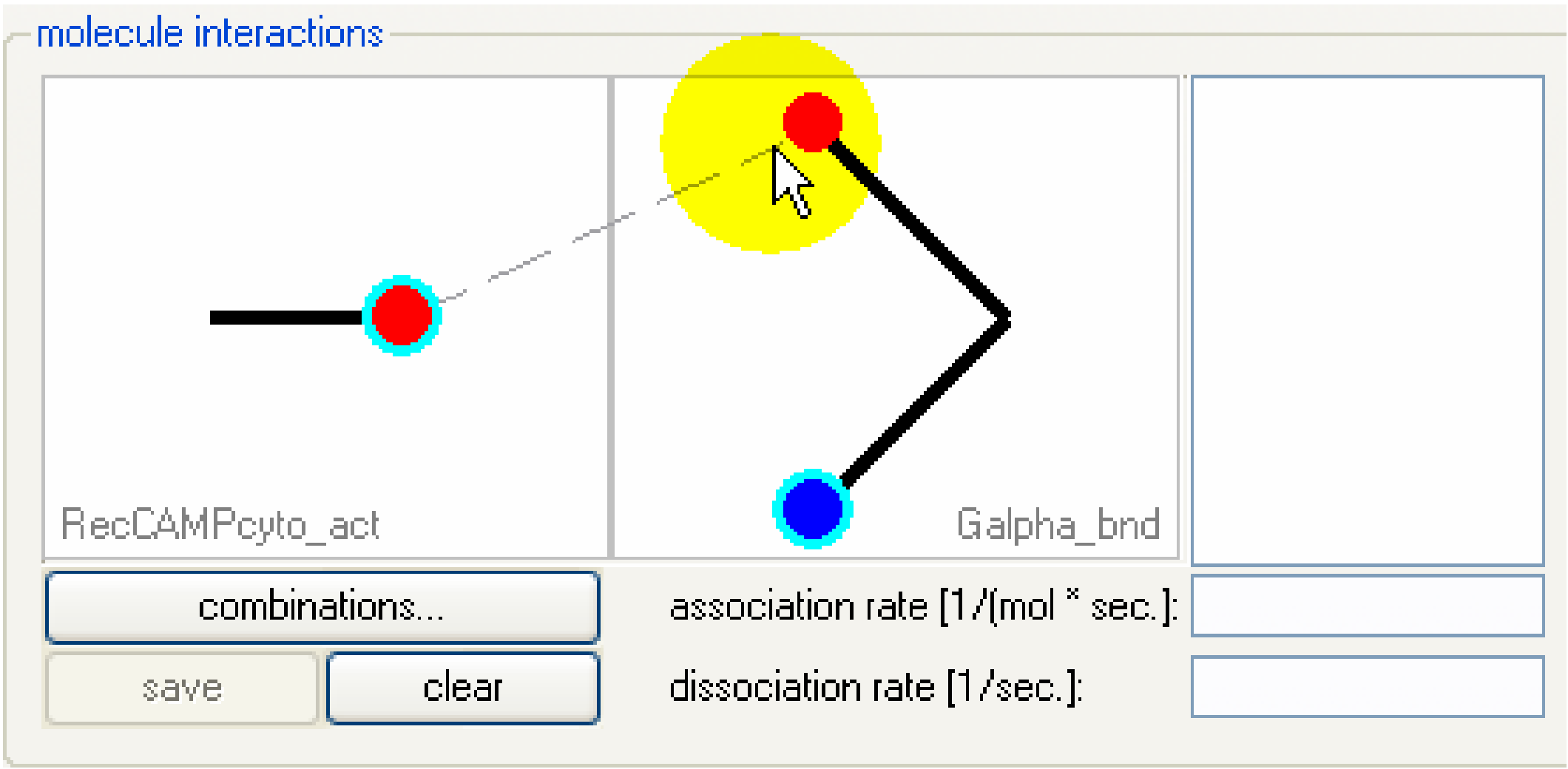

Simmod will remind you that RecCAMPcyto_act and Galpha_bnd are alternatives of other states and that Simmod will create default binding possibilities between all alternatives.

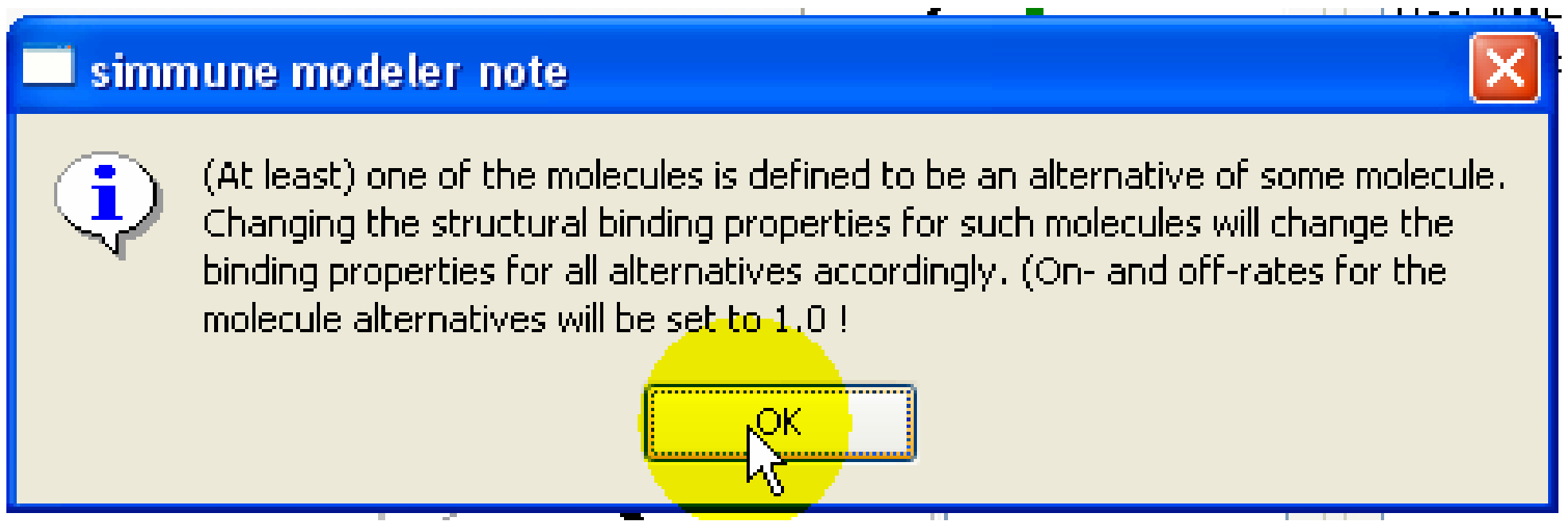

(12) Click the small rectangle at the link appearing between the binding sites.

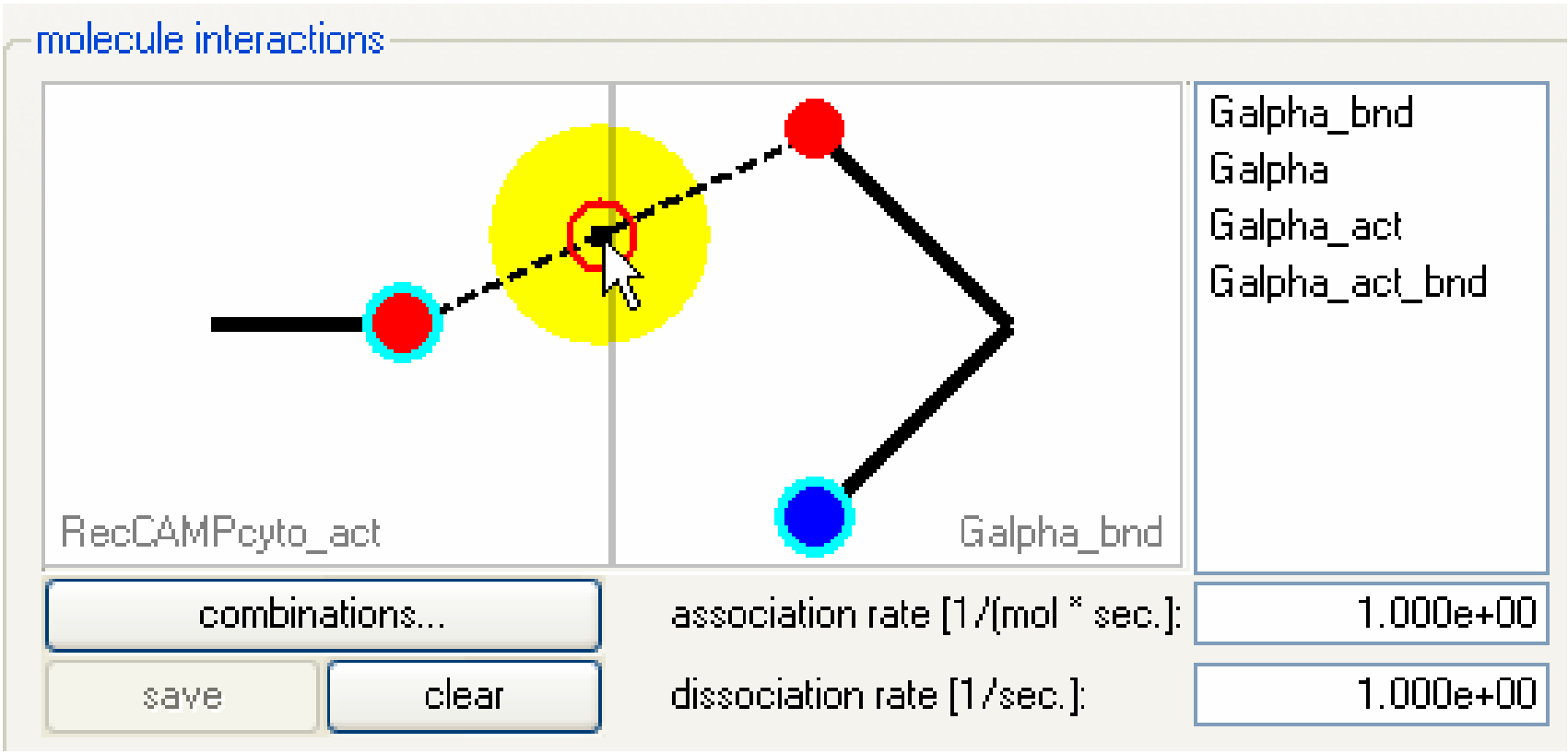

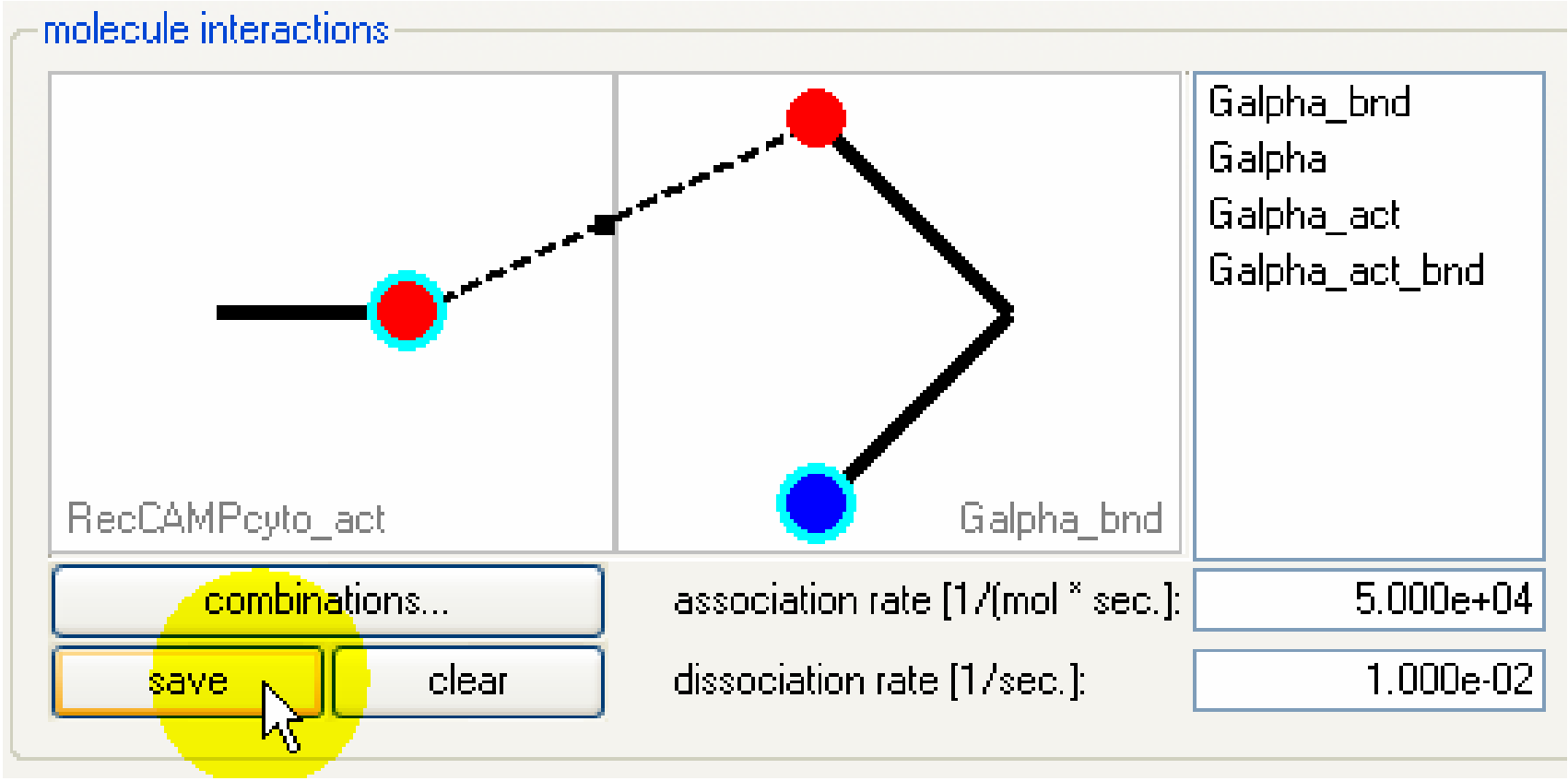

(13) Set the on- and off rates for the interaction.

(14) In the list of binding partners of RecCAMPcyto_act now double-click **Galpha_act_bnd** and activate the interaction by clicking the small rectangle at the link between the binding sites.

Galpha_act_bnd will be the result (product) of the action of the enzyme RecCAMPcyto_act on Galpha_bnd. The off-rate we enter here will be the rate of the enzymatic transformation of Galpha_bnd into Galpha_act_bnd.

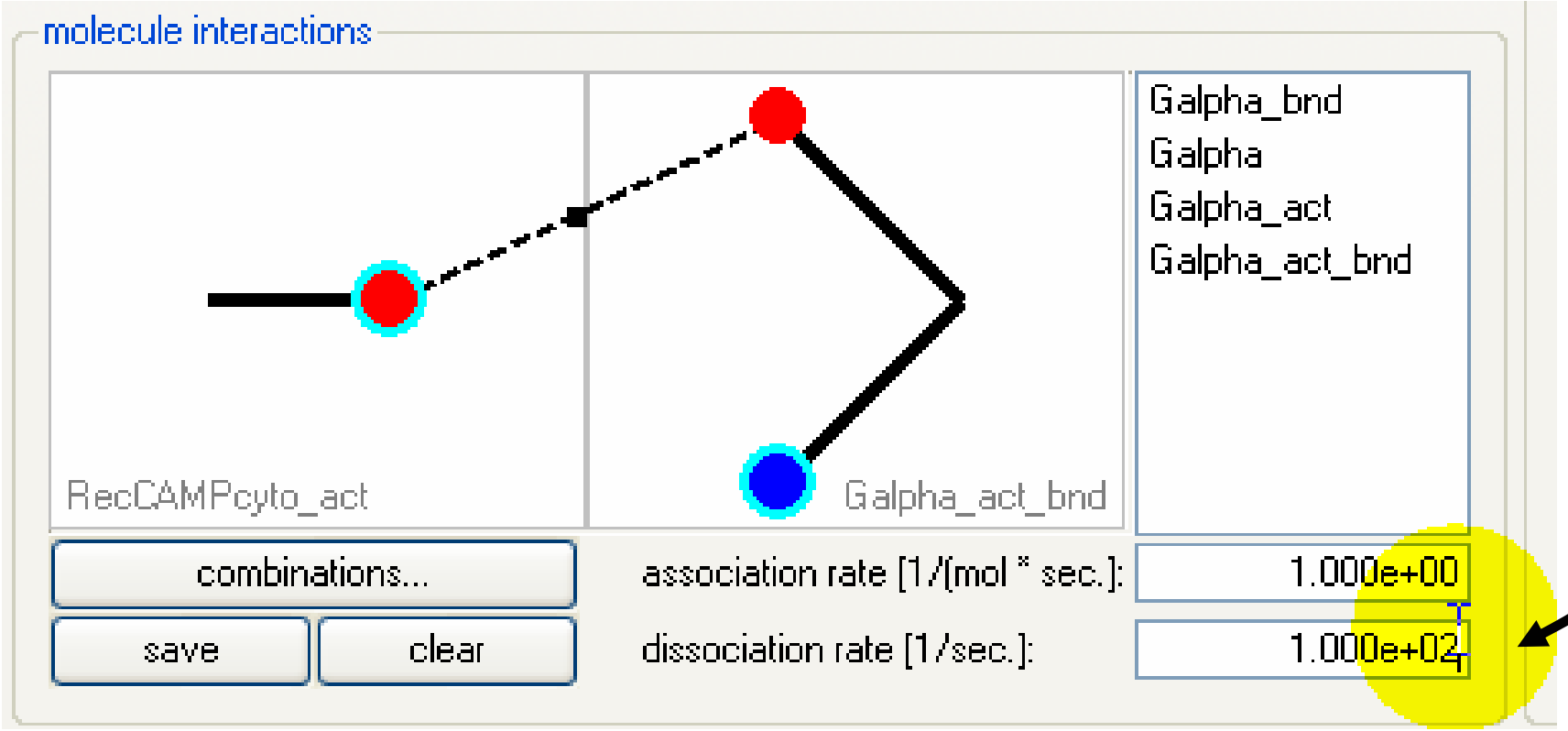

(15) Similar as before in steps (7) and (8) the Gabg complex now define the complex consisting of Galpha_act_bnd bound to Gbetagamma.

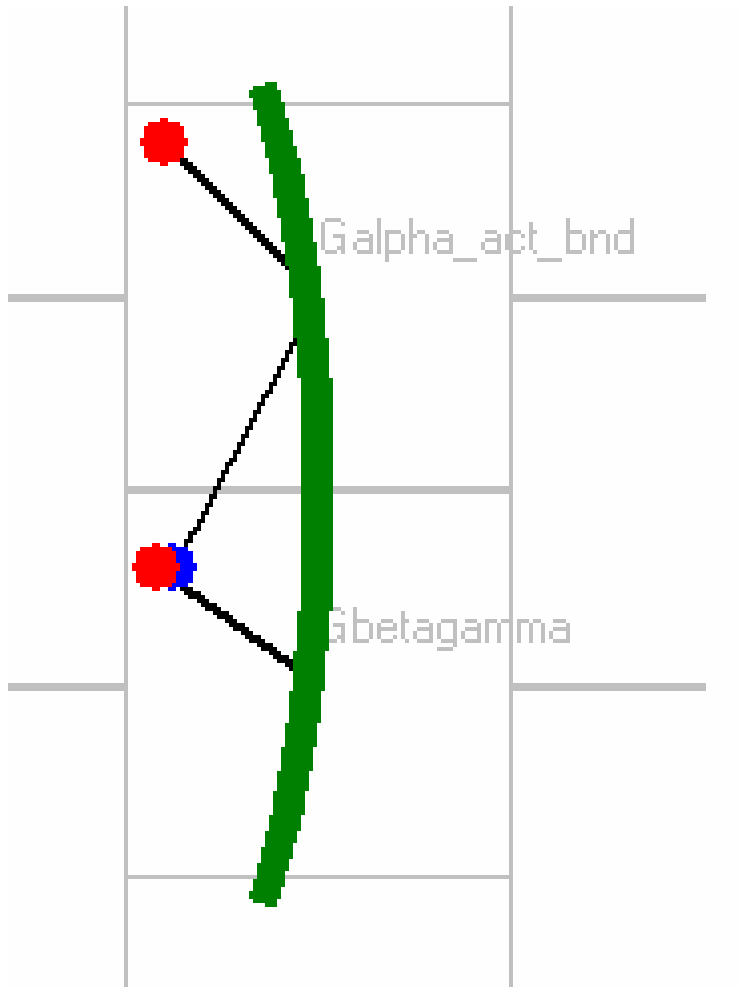

(16) Now, double-click the complex Gabg in the list of defined complexes

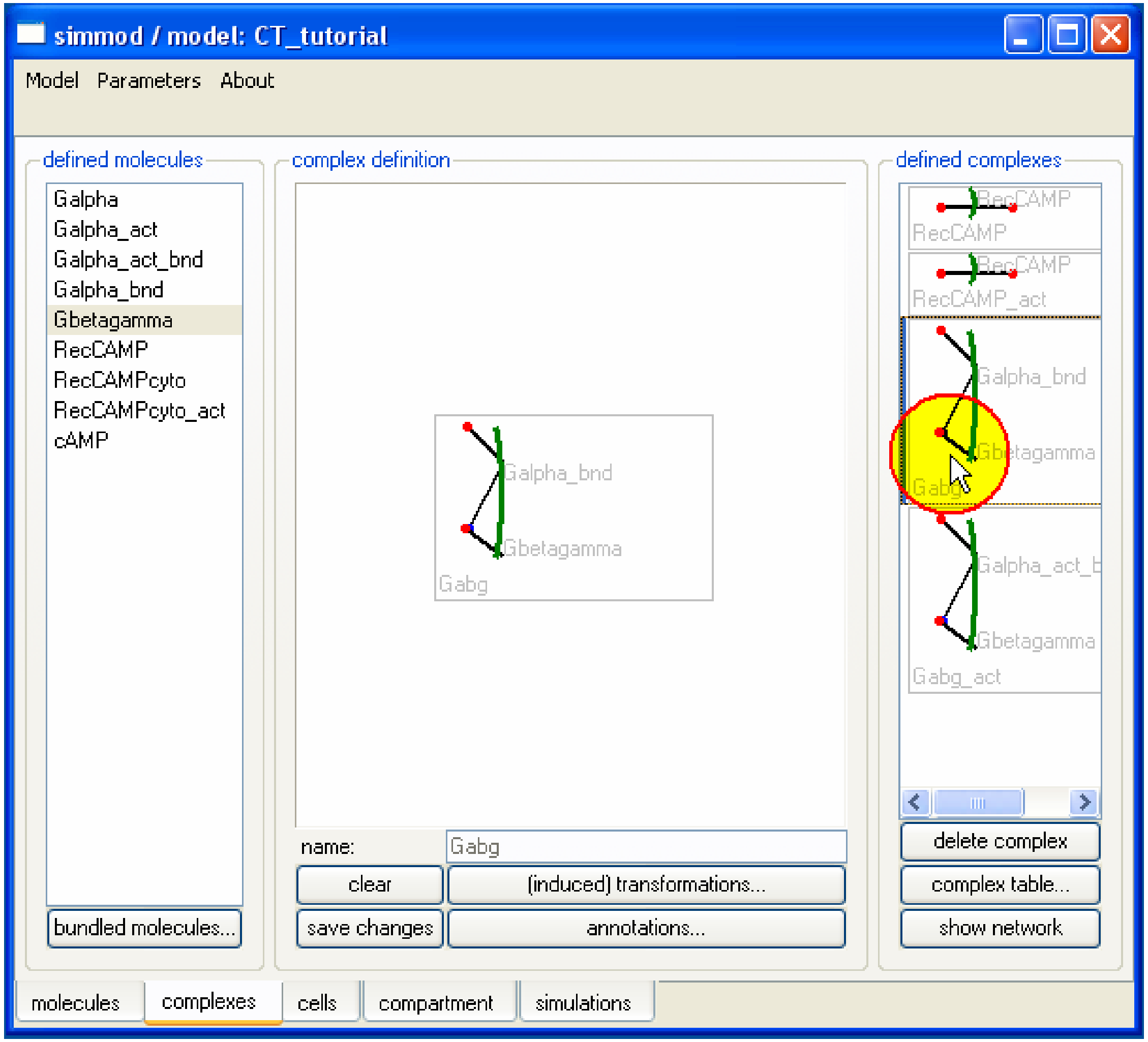

(17) Click the '(induced) transformations' button.

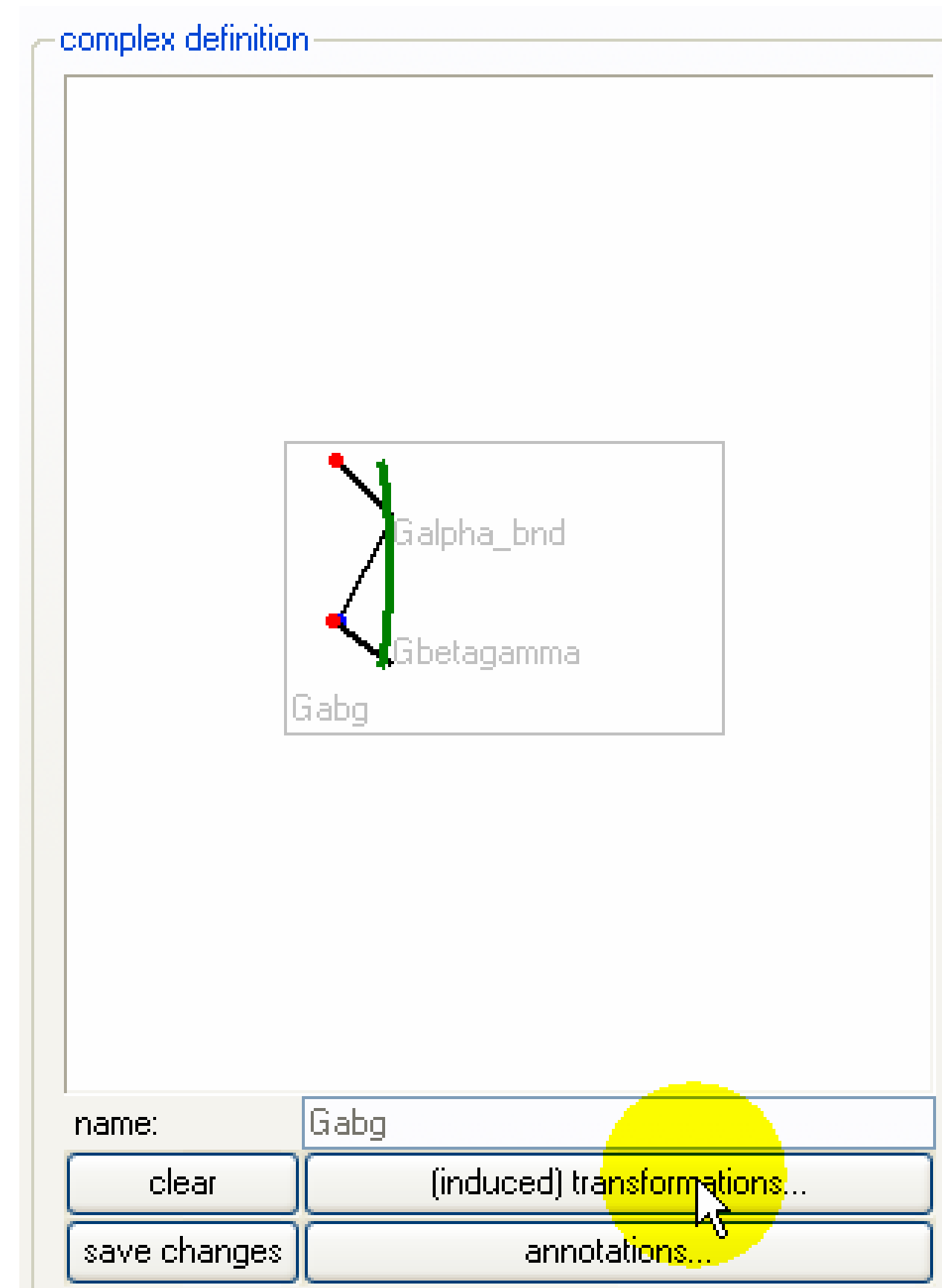

(18) A window for the definition of spontaneous and enzymatically induced transformations will appear.

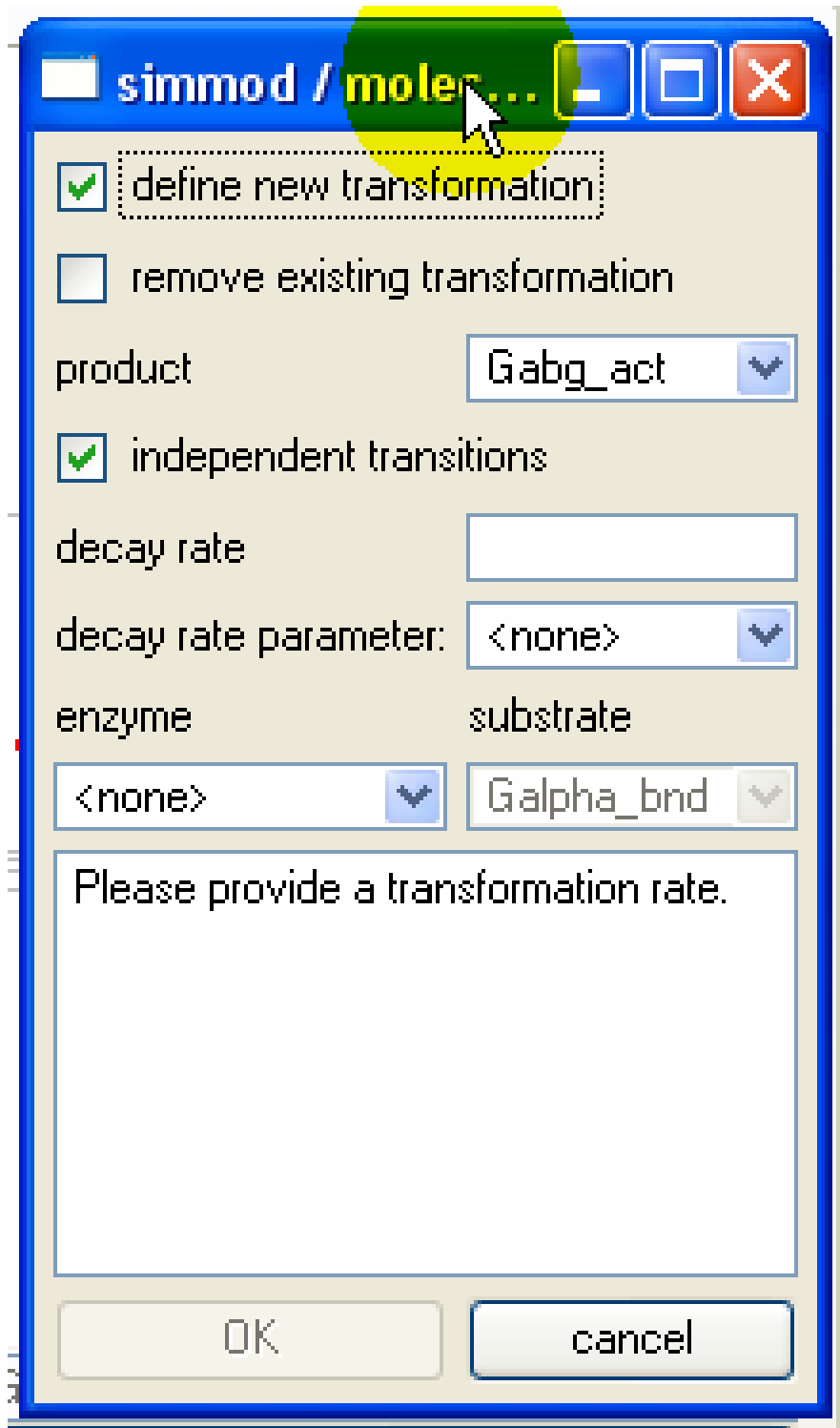

(19) Simmod will offer Gabg_act as product (it is the only complex we have defined so far that is structurally equivalent to Gabg). Select RecCAMPcyto_act as enzyme, Galpha_bnd as substrate and click OK.

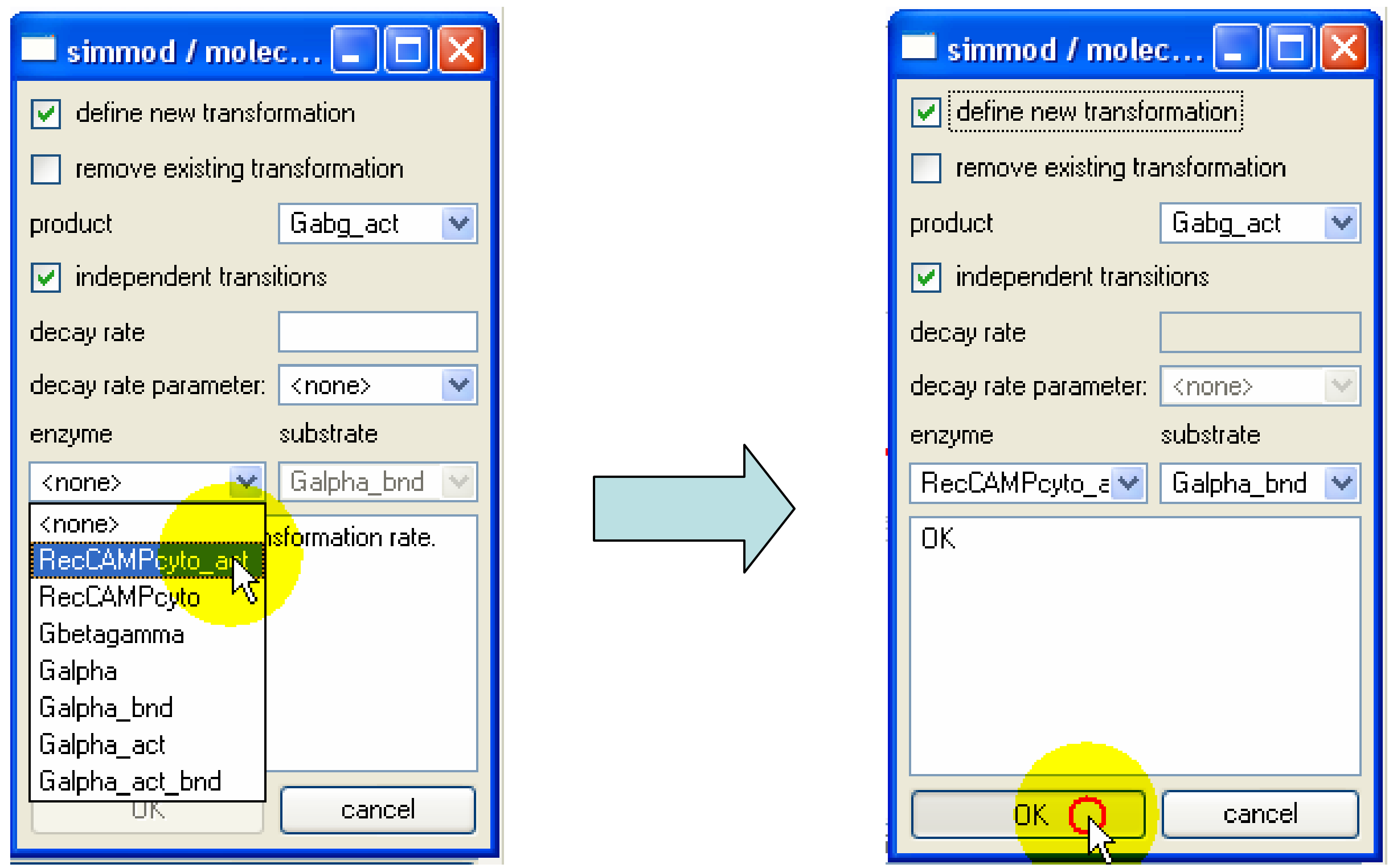

We have now defined the enzymatic transformation of Gabg into Gabg_act. Save the Gabg complex to make the changes active.

Click the ‘complex table’ button to inspect the molecule complexes we have defined so far.

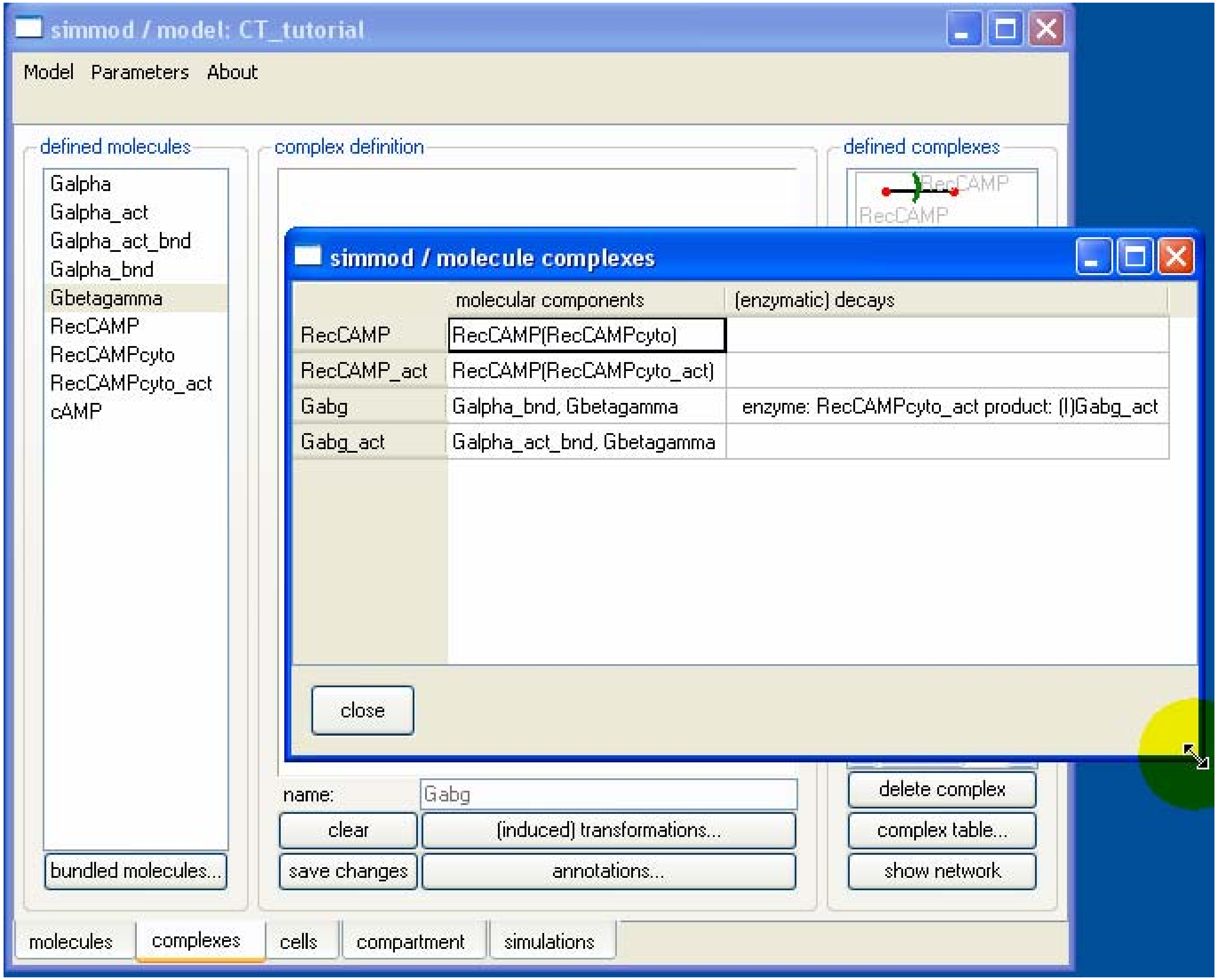

(20) Define a high off-rate for the dissociation of Galpha_act_bnd and Gbetagamma. Now, whenever Galpha_bnd becomes activated it rapidly dissociates from Gbetagamma.

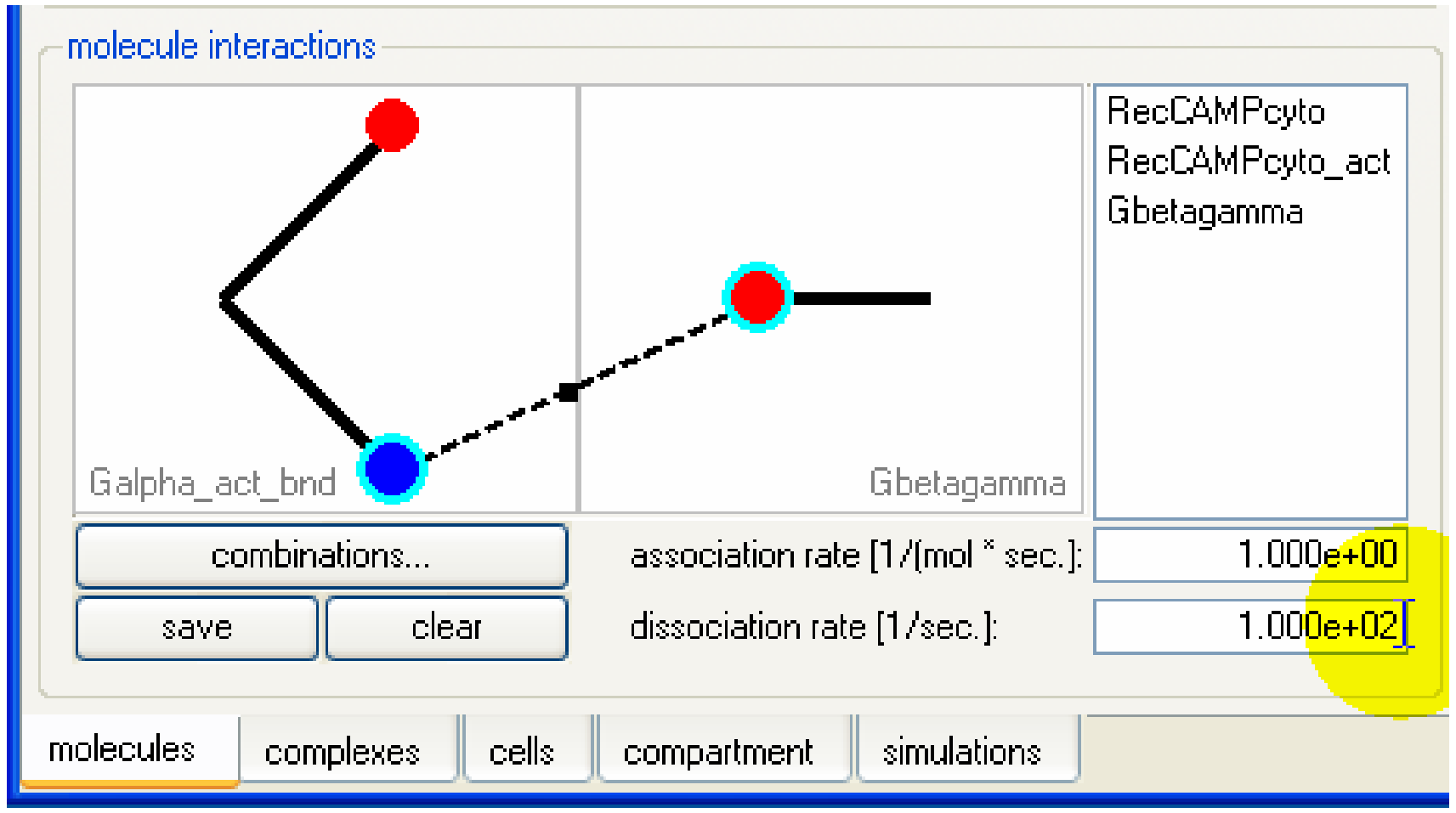

(21) **Important:** When Galpha_act_bnd dissociates from Gbetagamma it should assume the state Galpha_act, indicating that it is no longer bound to Gbetagamma. Define the corresponding transformation as a debinding-induced event analog to the steps 14-17 in tutorial 1.

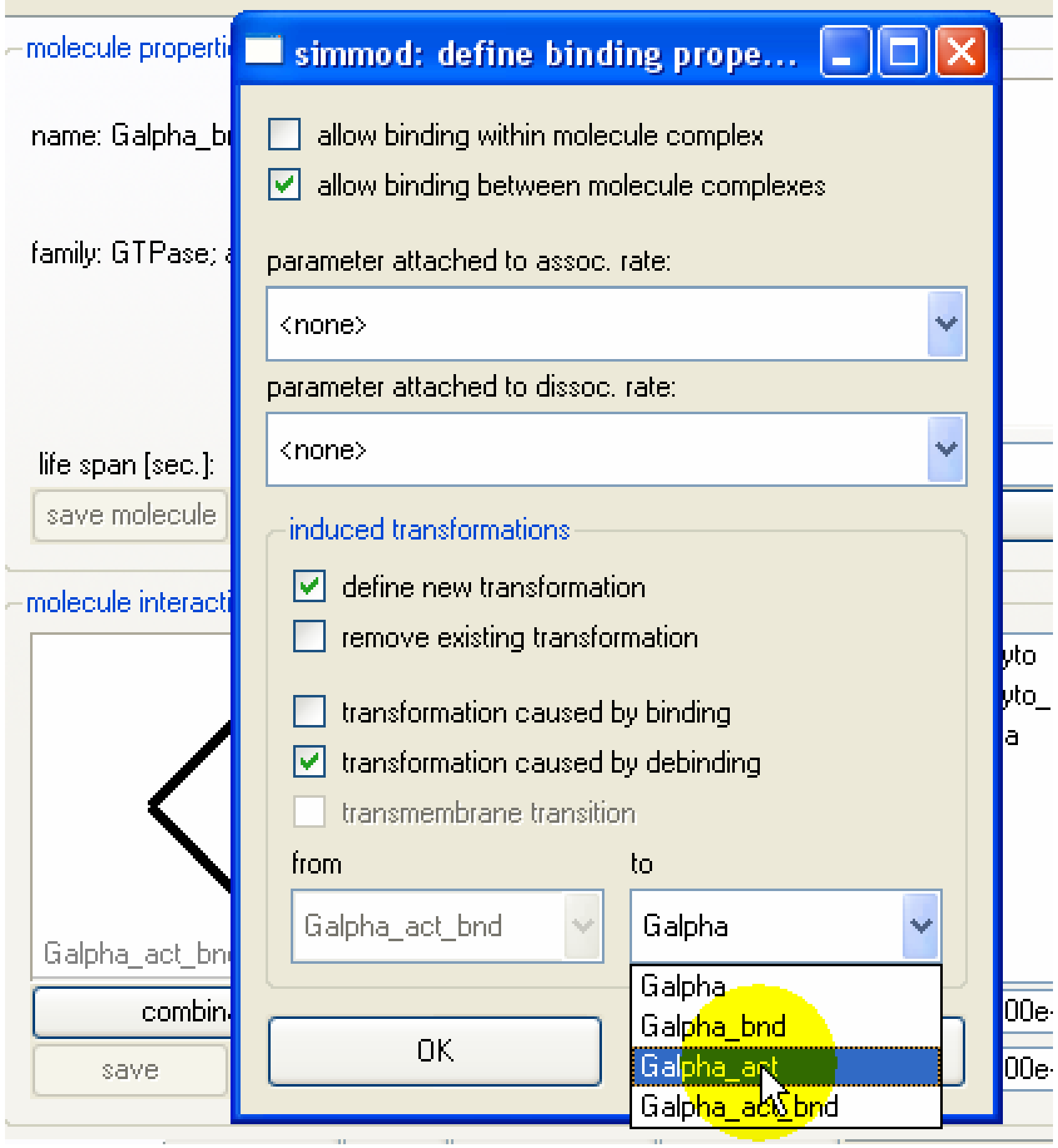

(22) Now, we want to define a spontaneous transformation of Galpha_act into Galpha. First, drag Galpha_act into the ‘complex definition’ field and save it as a complex with the same name (in principle, the name does not have to be the same but it helps to remember what the complex consists of). Analogously for Galpha.

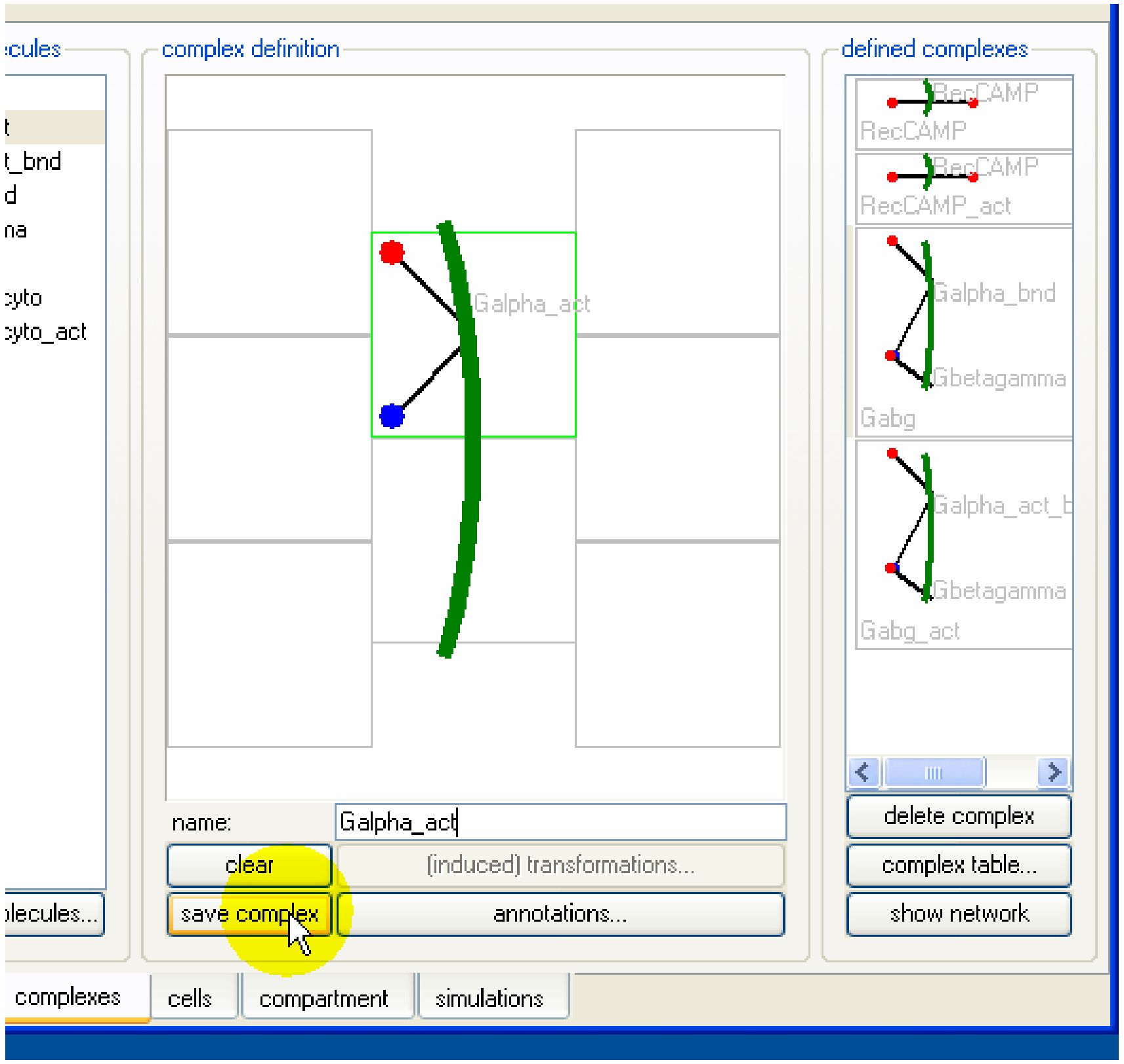

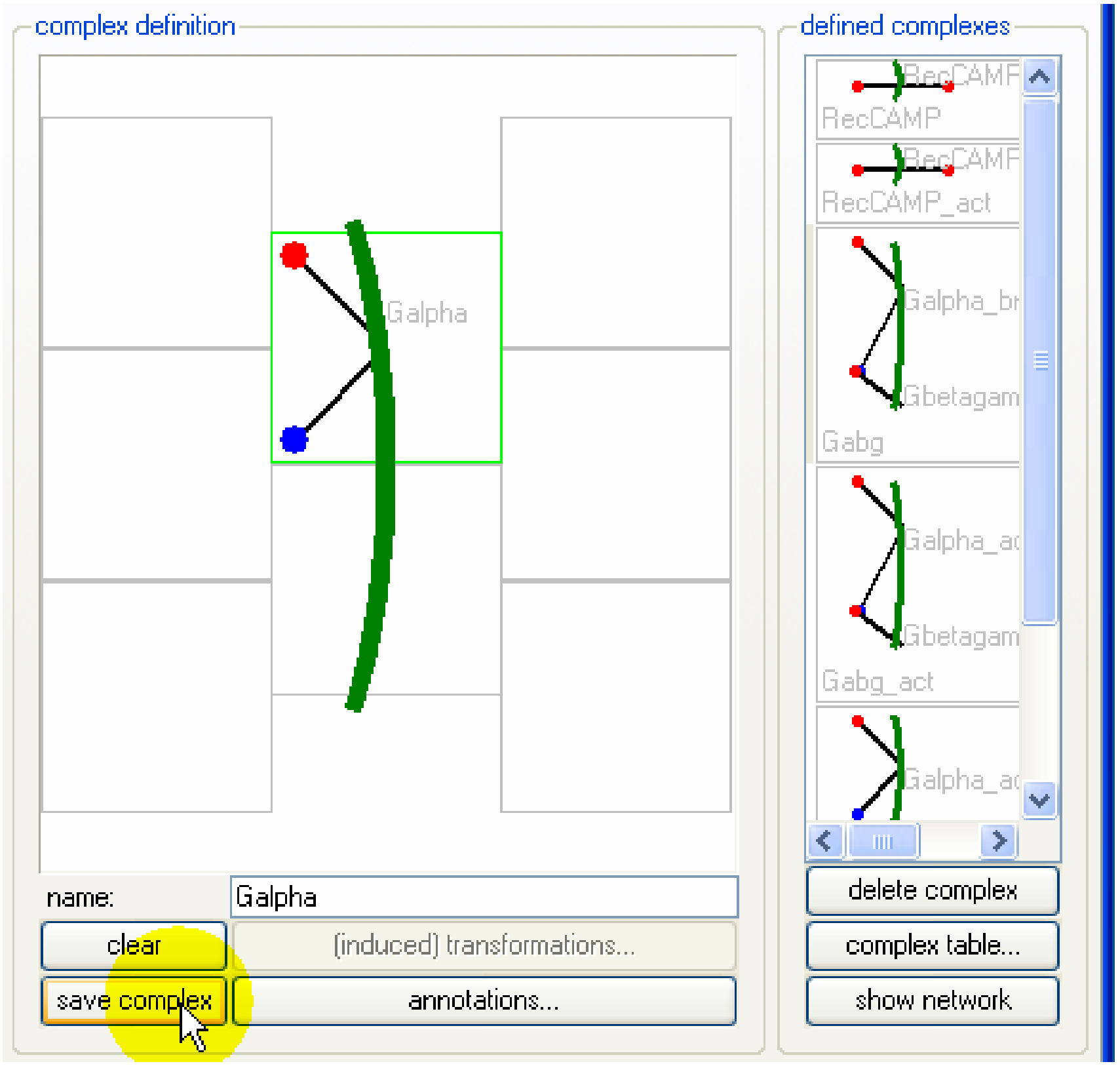

(23) Double-click the Galpha_act complex in the list of defined complexes to select it for editing.
(24) Click the ‘(induced) transformations’ button and then define a spontaneous transformation (without enzyme) from Galpha_act to Galpha.
Don’t forget to save these new properties of Galpha_act.

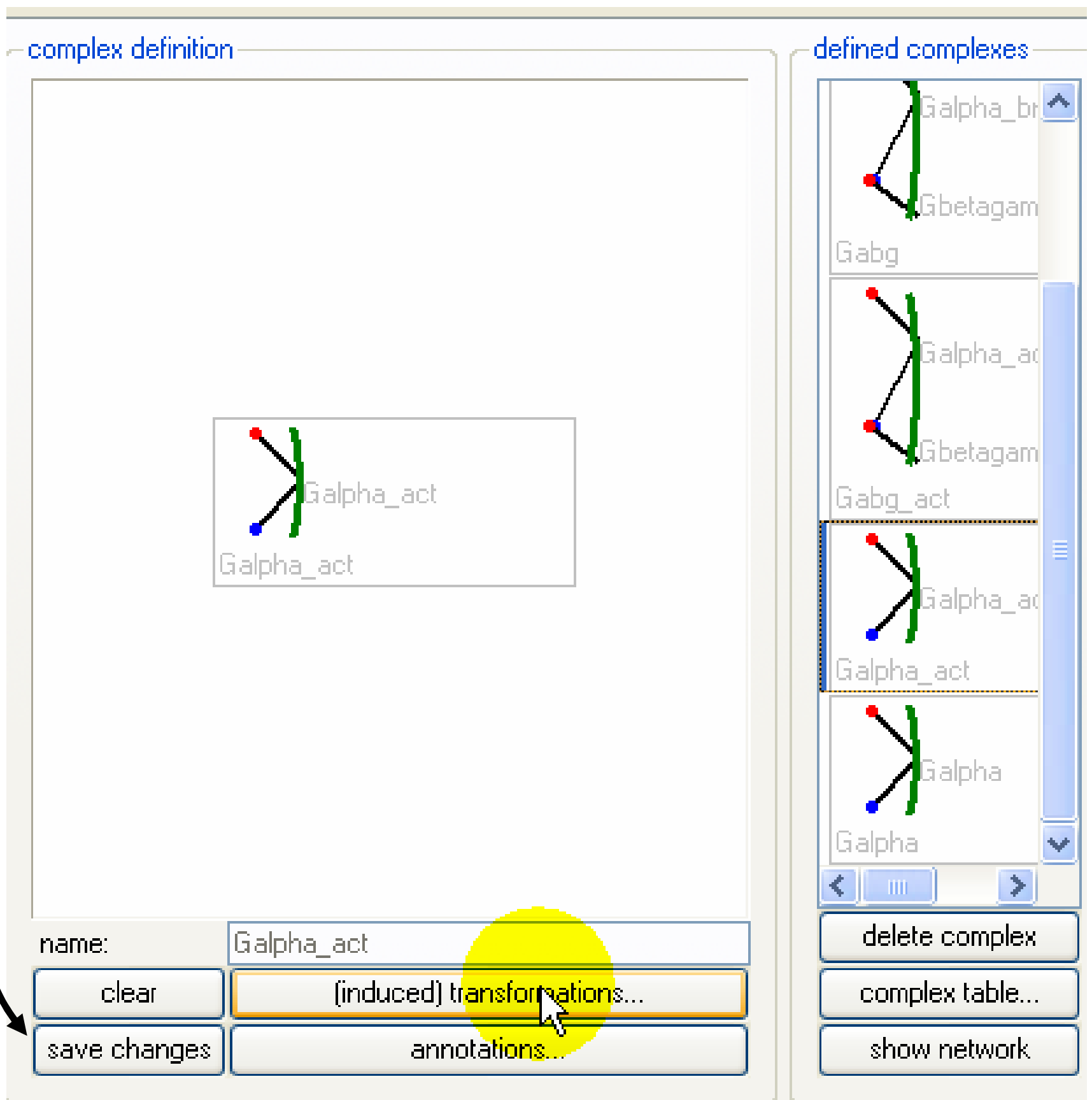

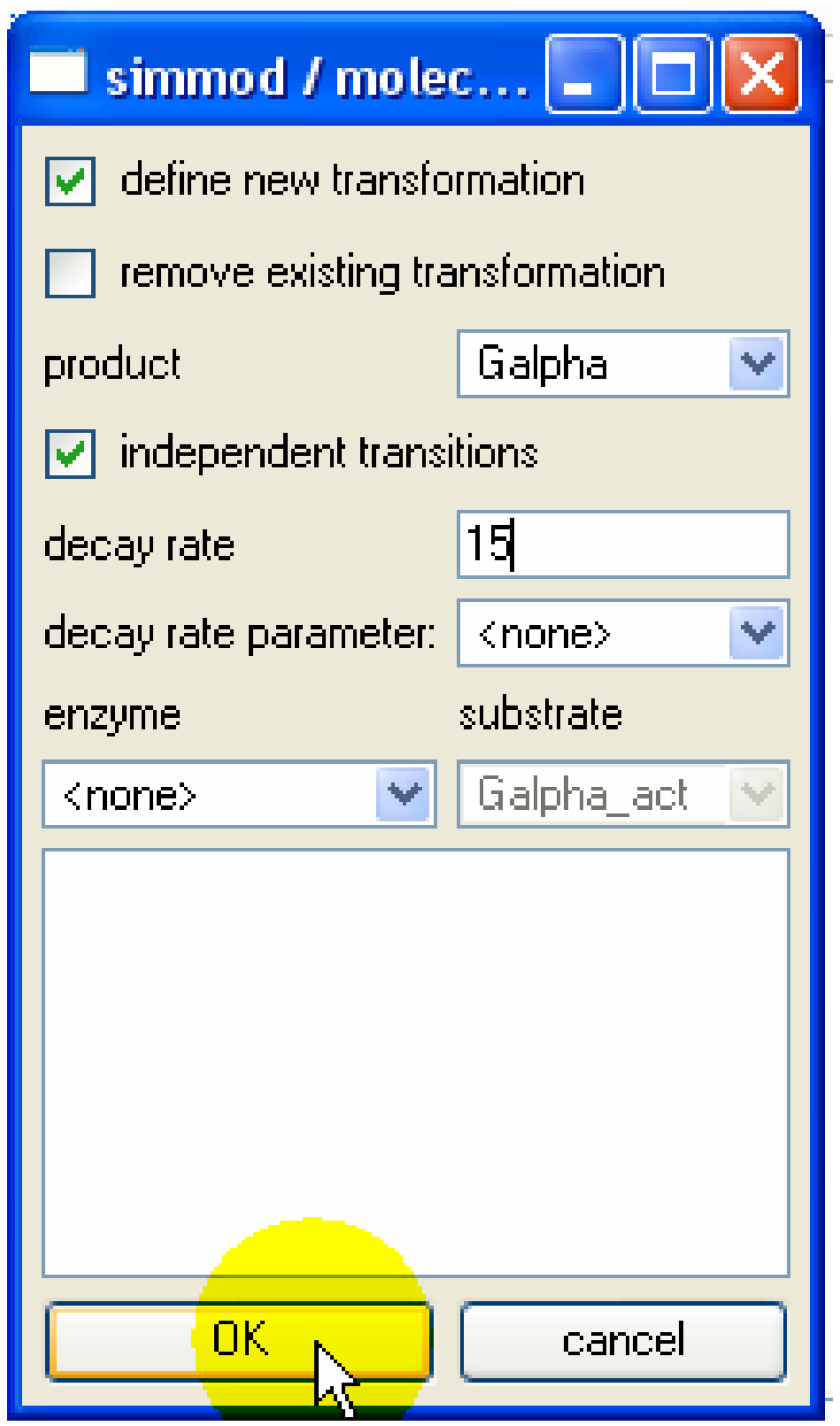

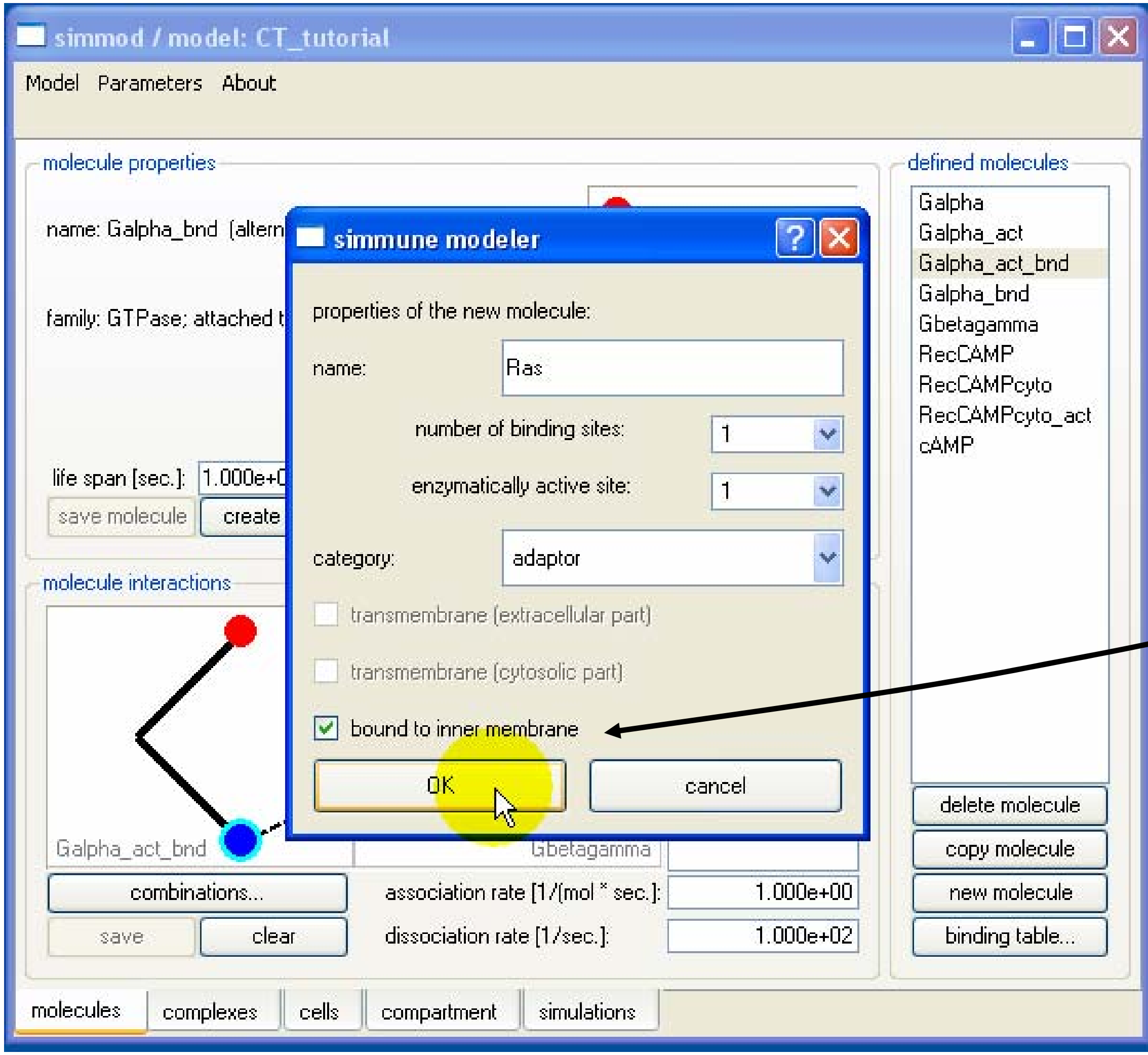

(25) Now, we will include a molecule downstream of the G proteins: Ras. Create it as an adaptor with one binding site which is enzymatically active.

Ras is typically membrane-bound.

(26) Define the appropriate diffusion coefficient for Ras.

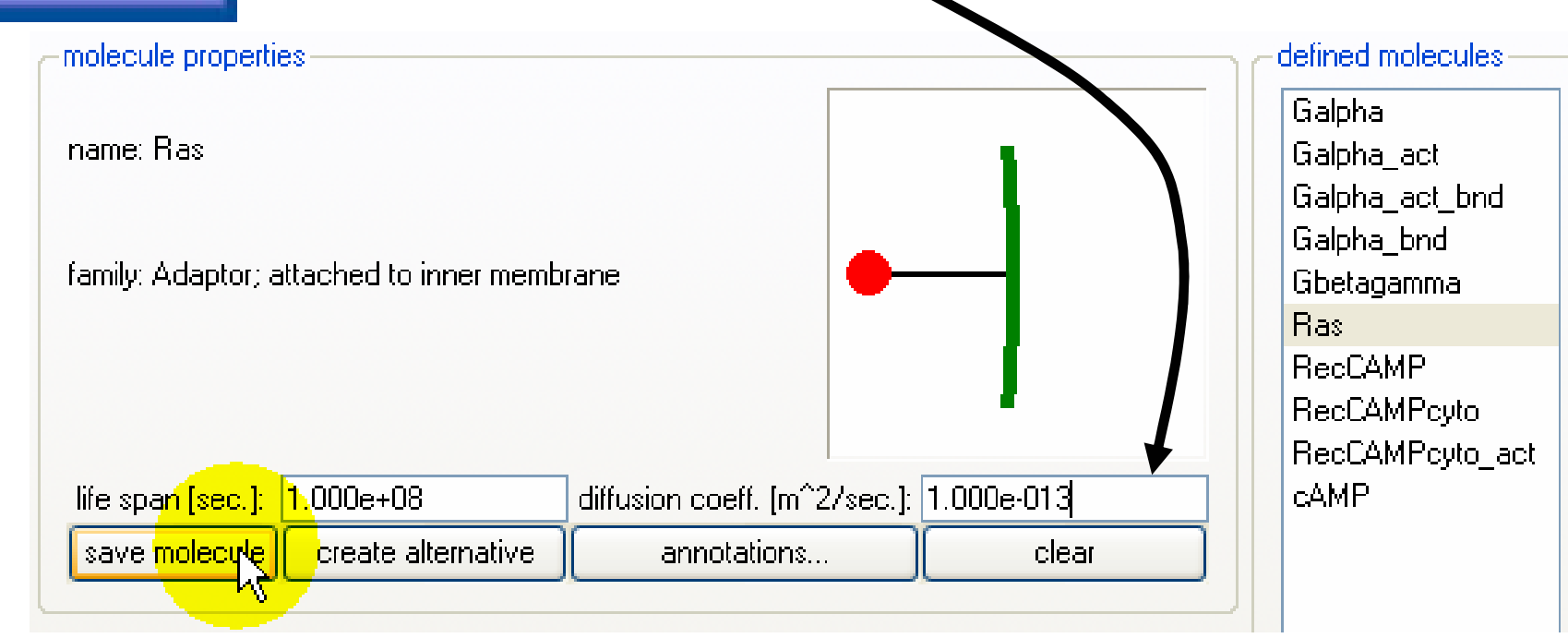

(27) Create an alternative (Ras_act) of Ras.

(28) Define the interaction between Gbetagamma and Ras, as shown below.

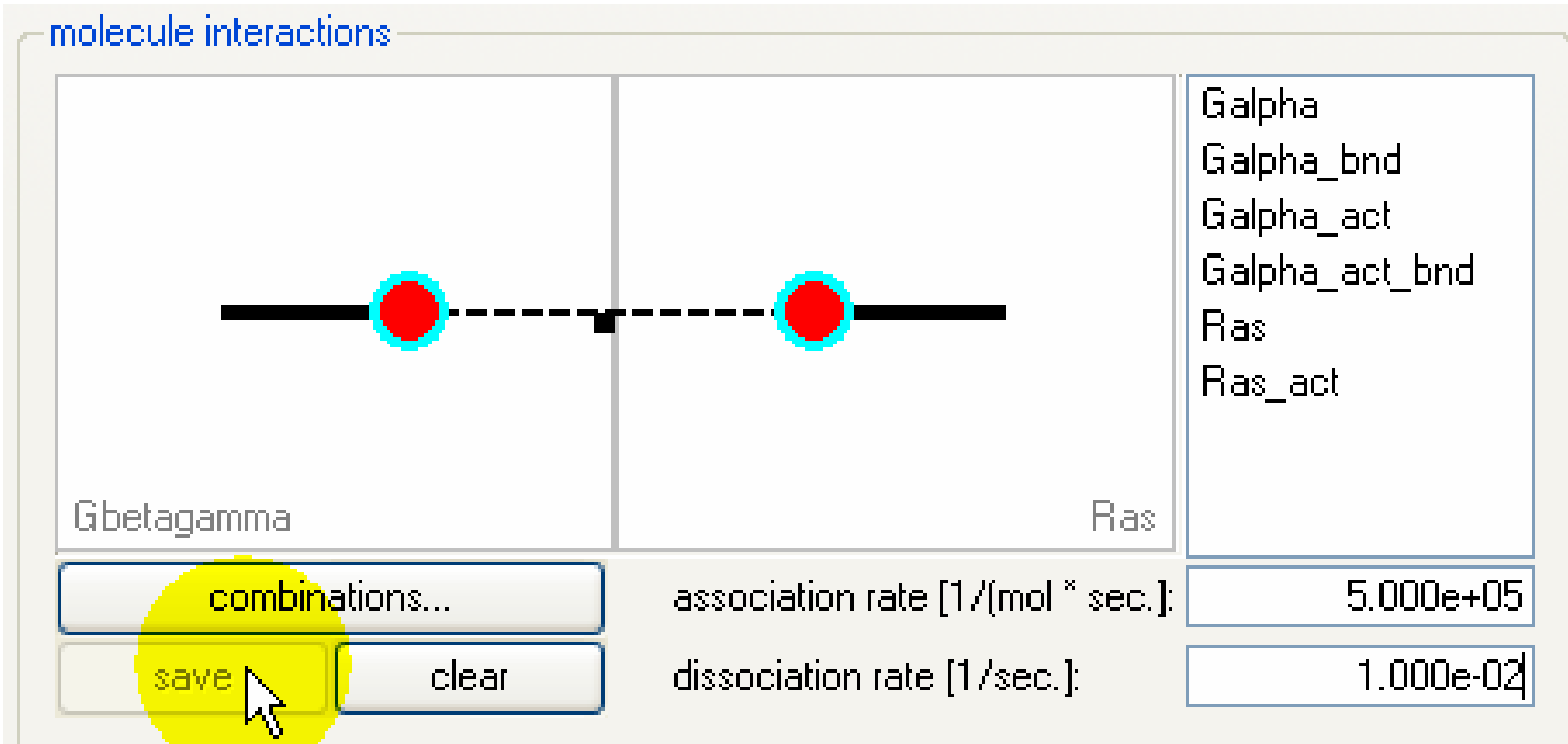

(29) We will define an enzymatic transformation from Ras to Ras_act, mediated by Gbetagamma.
The off-rate for the dissociation between the enzyme (Gbetagamma) and the product (Ras_act) will be the enzymatic transformation rate.

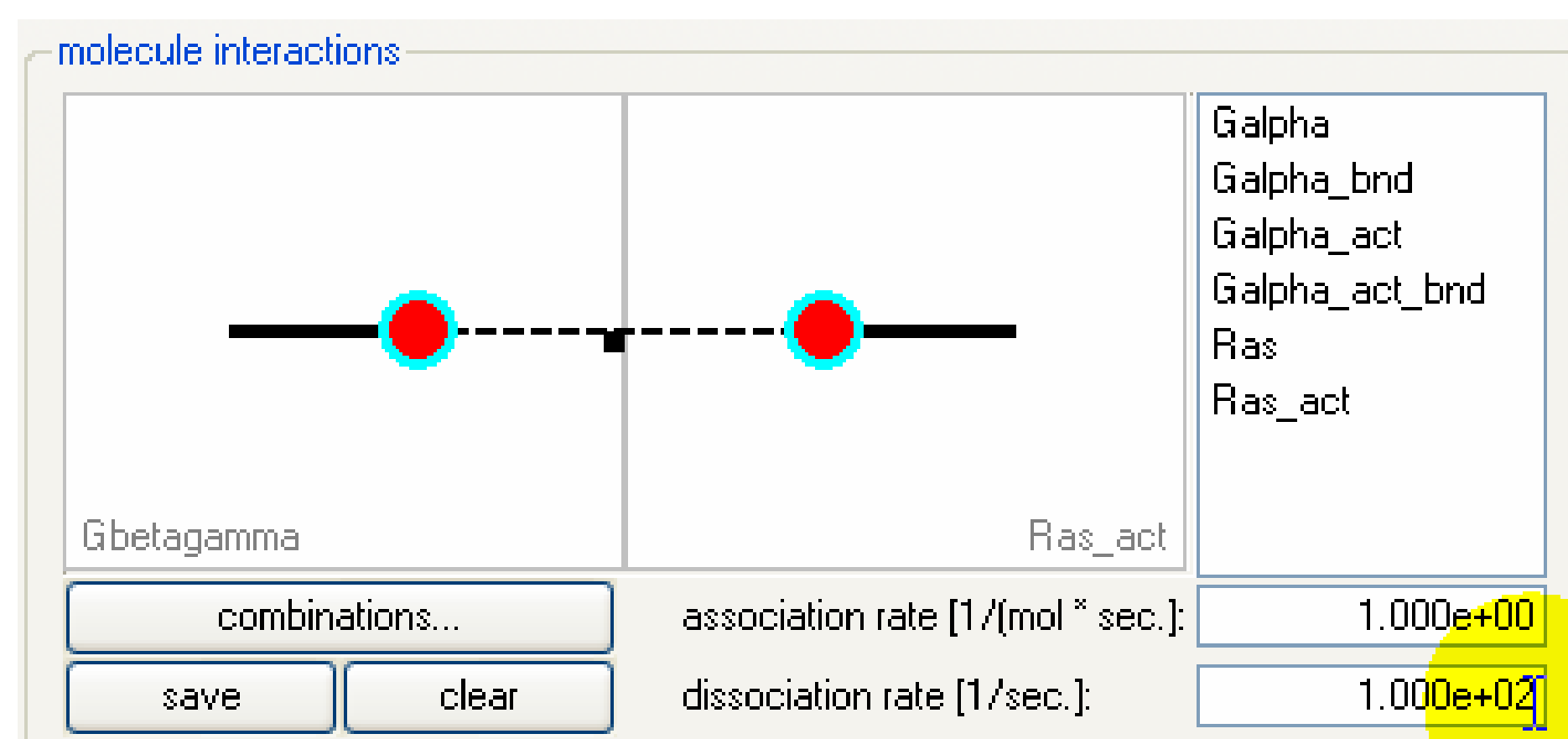

(30) Create the Ras and Ras_act complexes and define the enzymatically induced transformation from Ras to Ras_act as in steps 16-19 for the transformation from Gabg to Gabg_act.

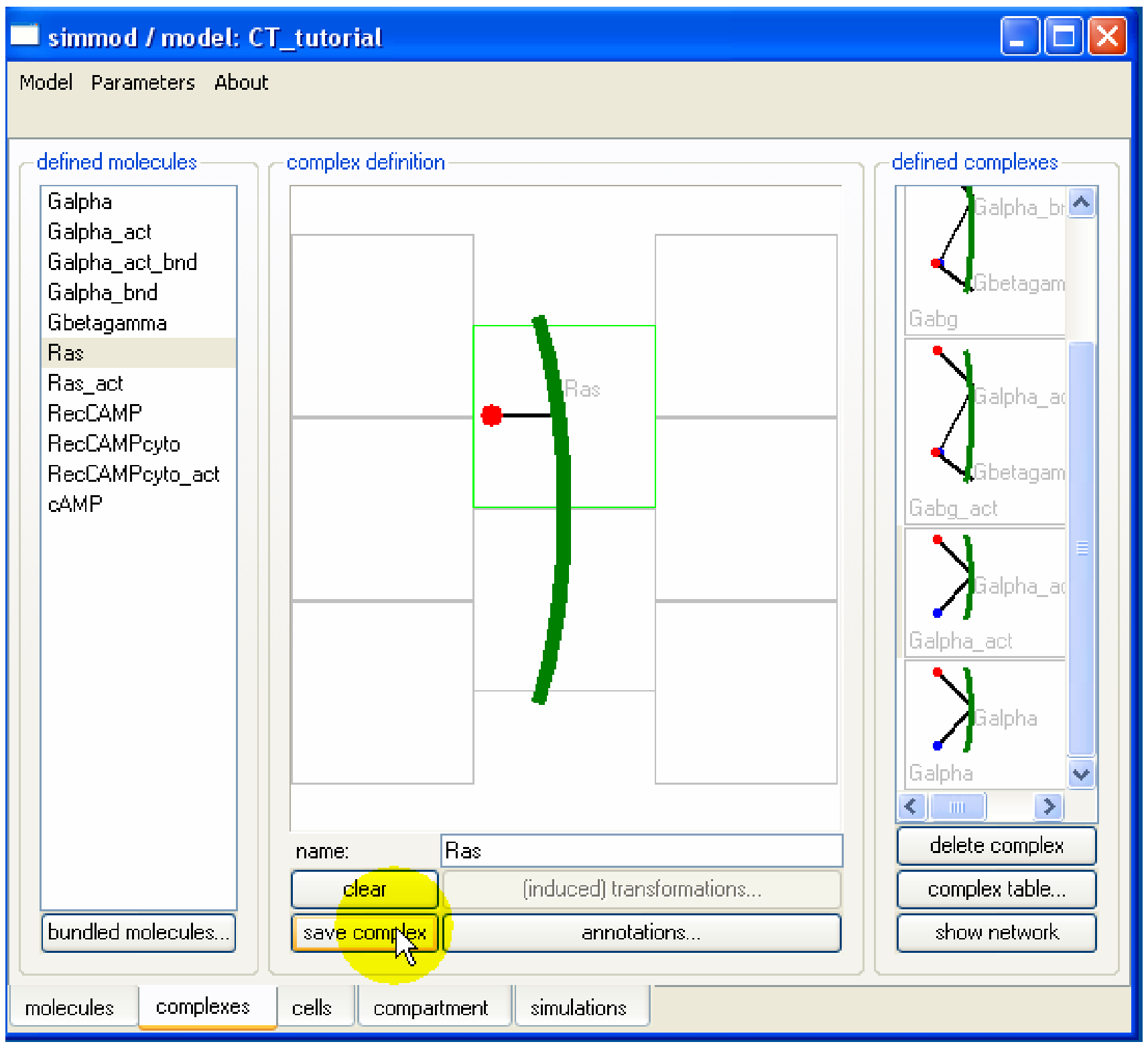

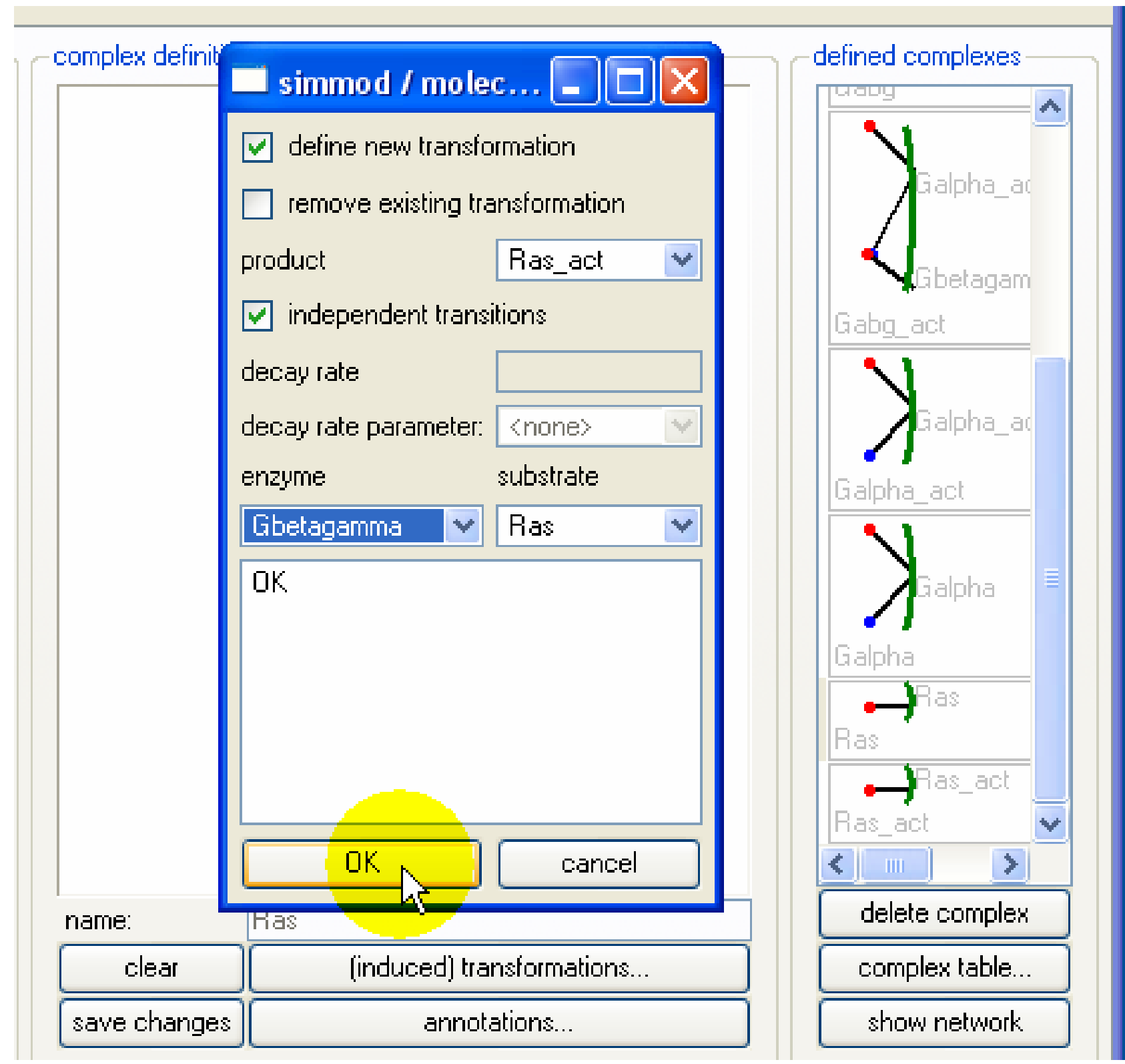

Supplement: Supplementary file 10 [file pcbi.0020082.sd010.pdf]
