# Supplementary material for: Key Role of Local Regulation in Chemosensing Revealed by a New Molecular Interaction-Based Modeling Method
Source: PLoS Comput Biol. 2006 Jul 21;2(7):e82. doi: 10.1371/journal.pcbi.0020082 (PMC1513273; doi:10.1371/journal.pcbi.0020082)

Simmune tutorial 4

Defining the initial biochemistry of cells
Defining the extracellular space
Simulating the model

(1) Load the CT_tutorial model from Tutorial 3.

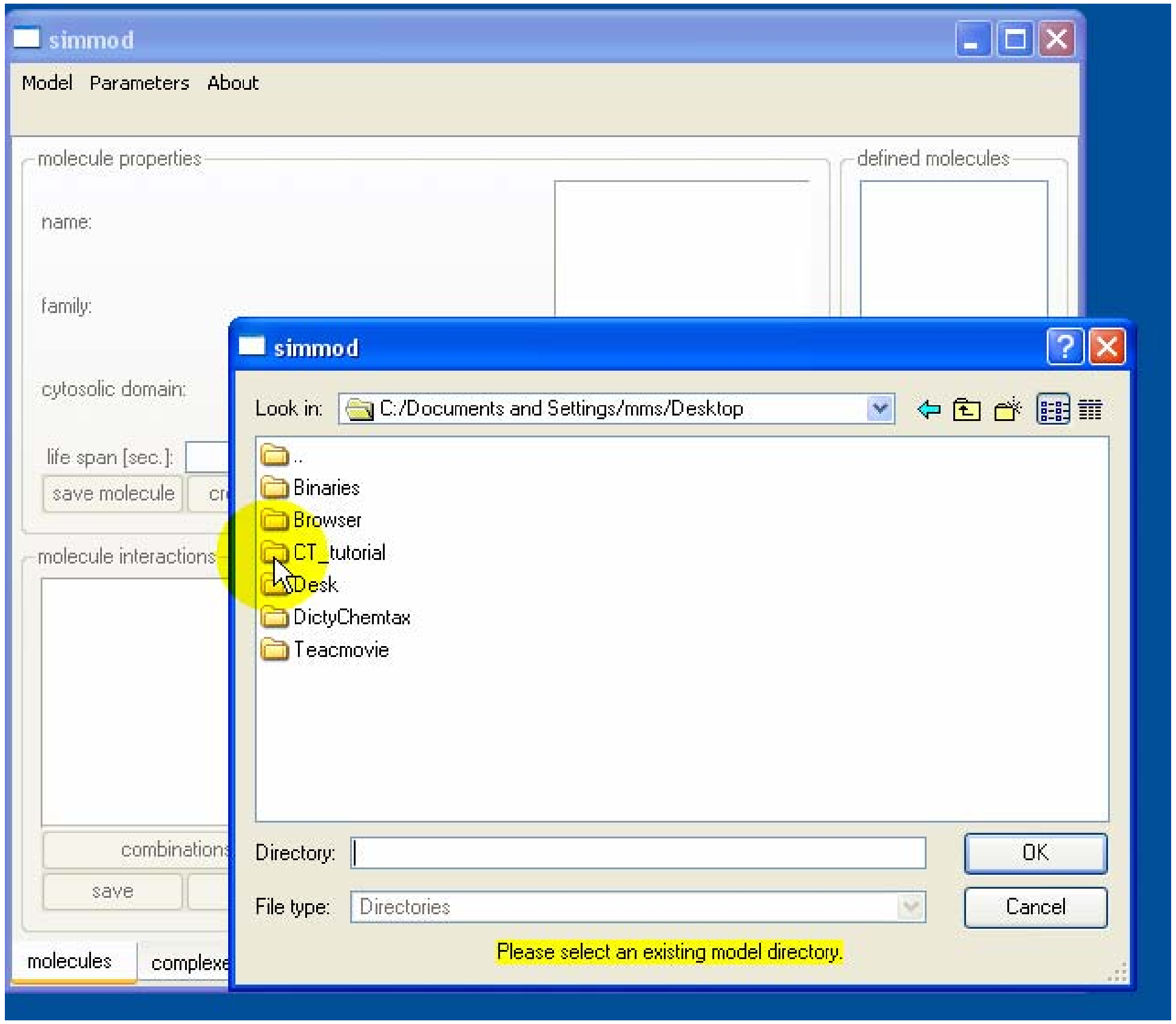

(2) Go to the 'complexes' tab.

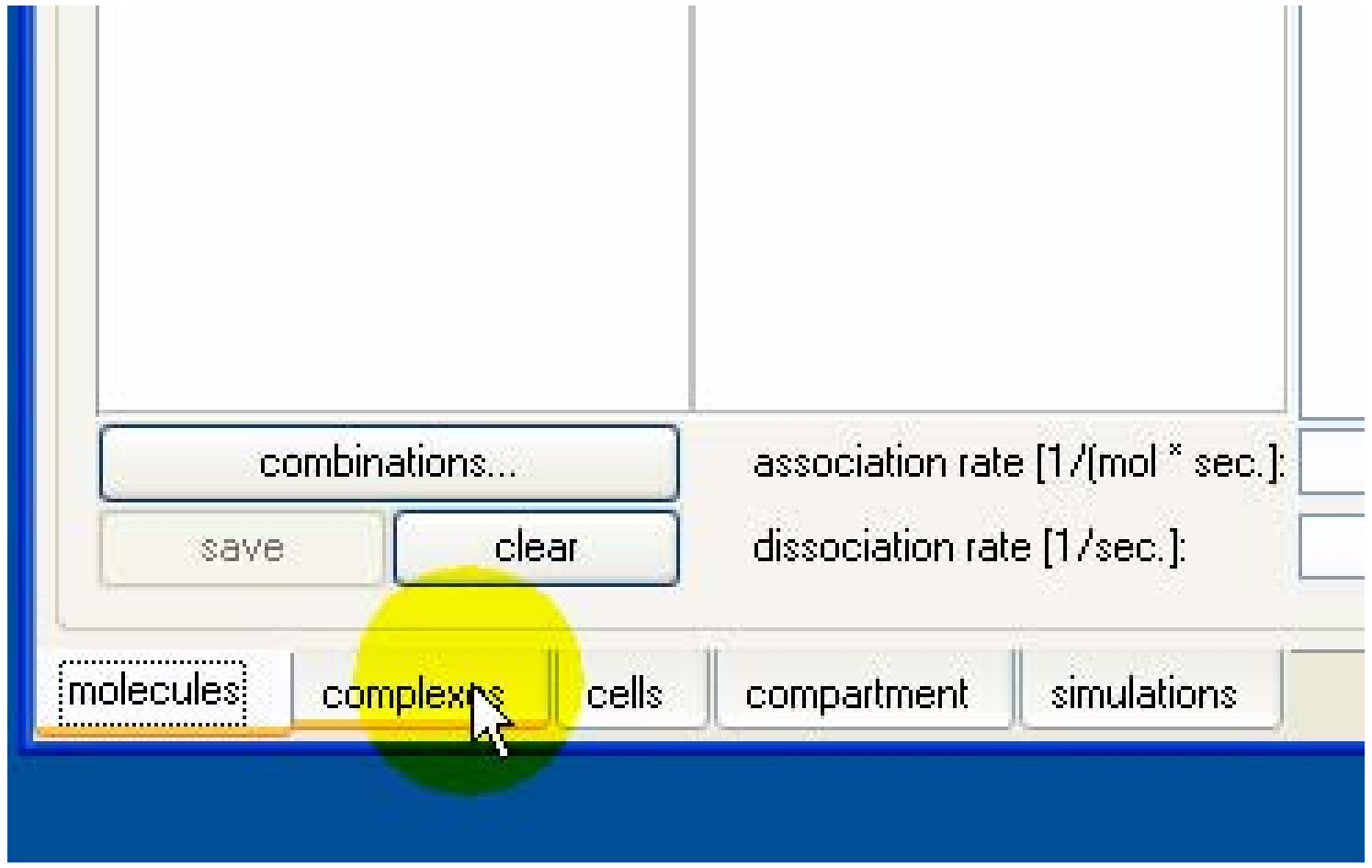

(2) Because only complexes (but not molecules) can be used for the definition of cellular behavior cAMP, the extracellular agent has to be defined as complex:

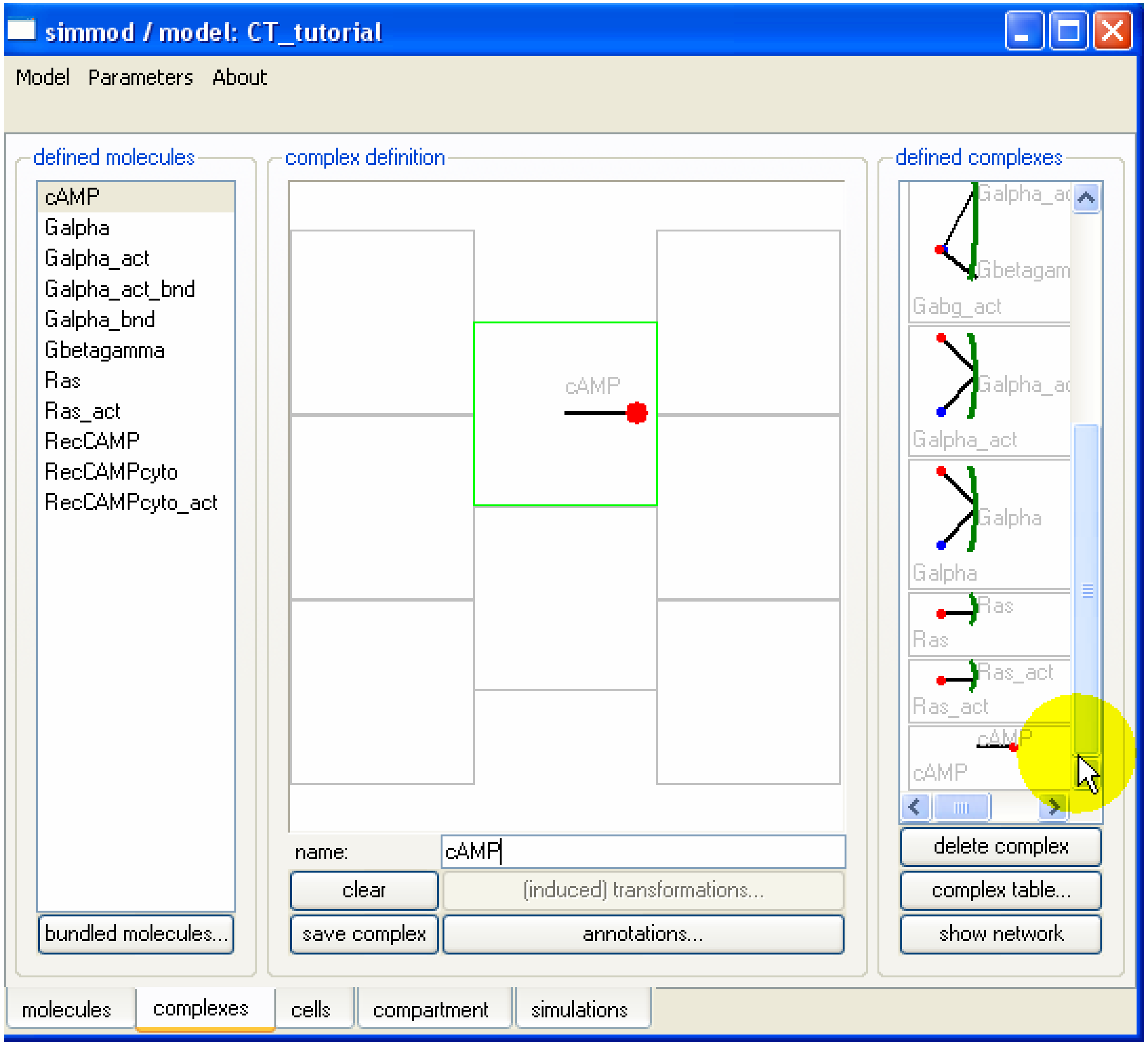

(3) We defined Gbetagamma to activate Ras. Since there will always be free Gbetagamma we have to add a mechanism that deactivates Ras_act again since we would otherwise end up with all Ras being activated – even without extracellular stimulus. Here, we define a spontaneous transformation from Ras_act to Ras. (In a more realistic model, a RasGAP would deactivate Ras_act.)

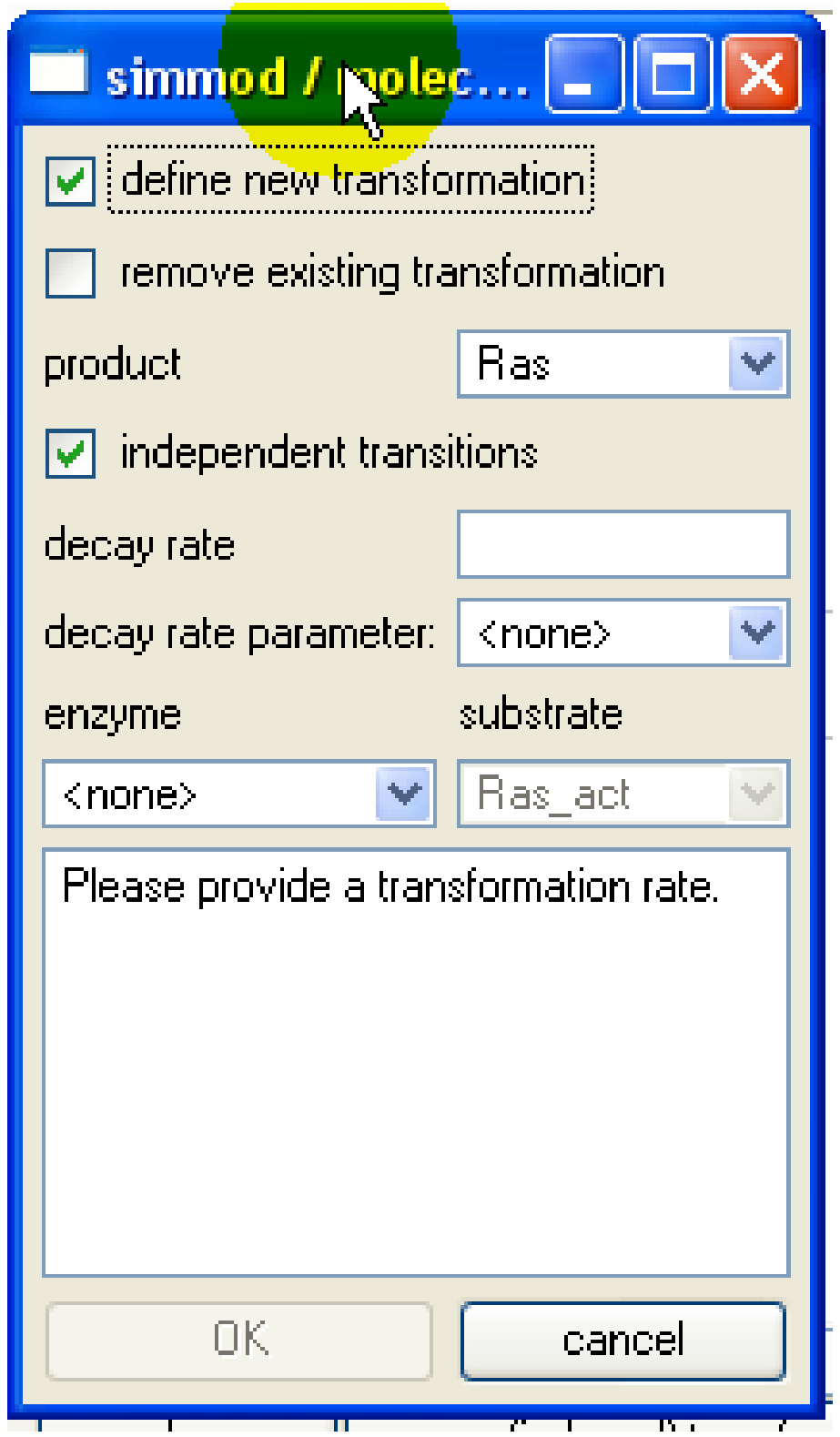

(4) In order to be able to track collections of molecule complexes with certain components one can define ‘bundles’. Click on ‘bundled molecules’ and then in the window that appears on ‘add bundle’.

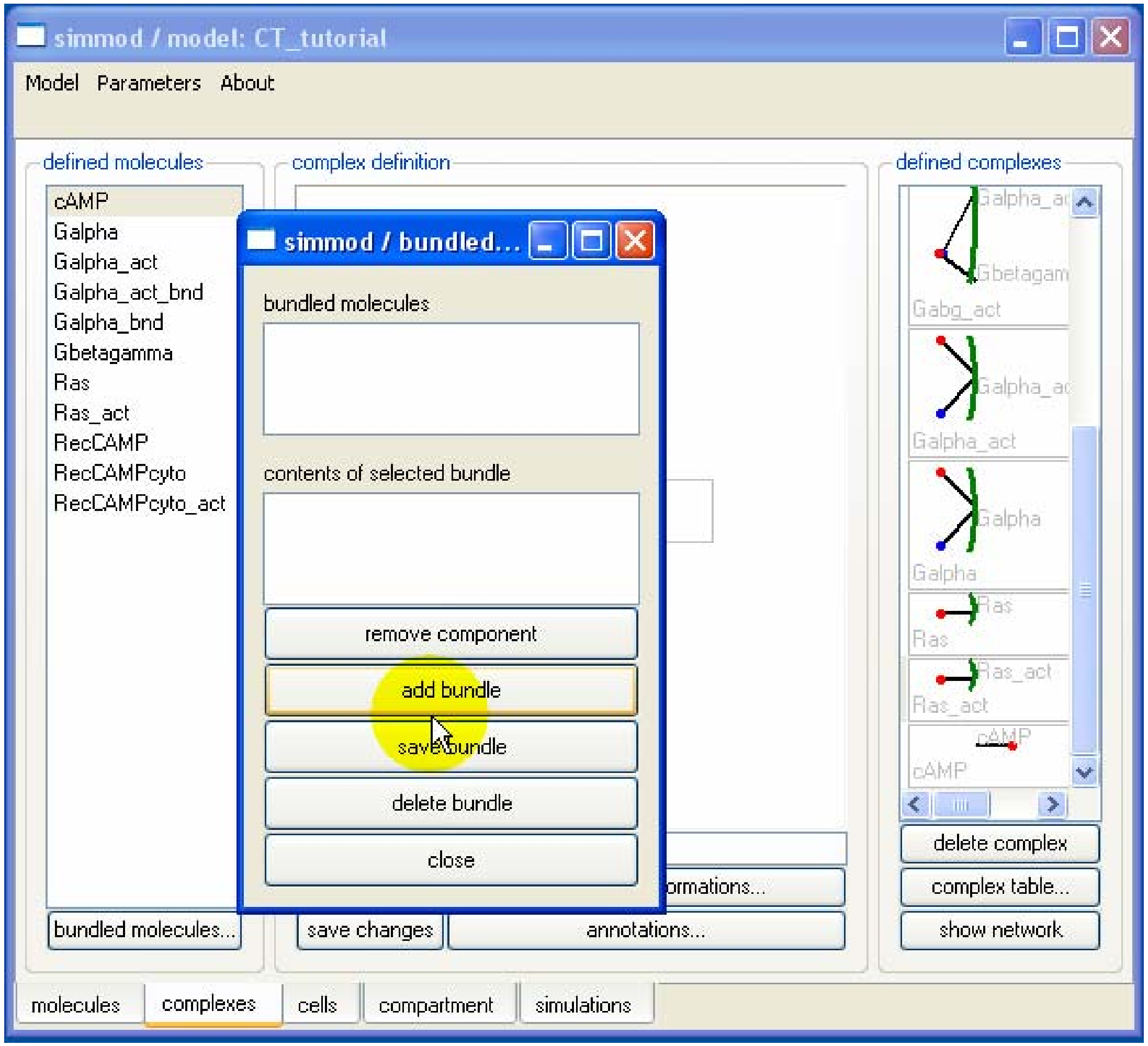

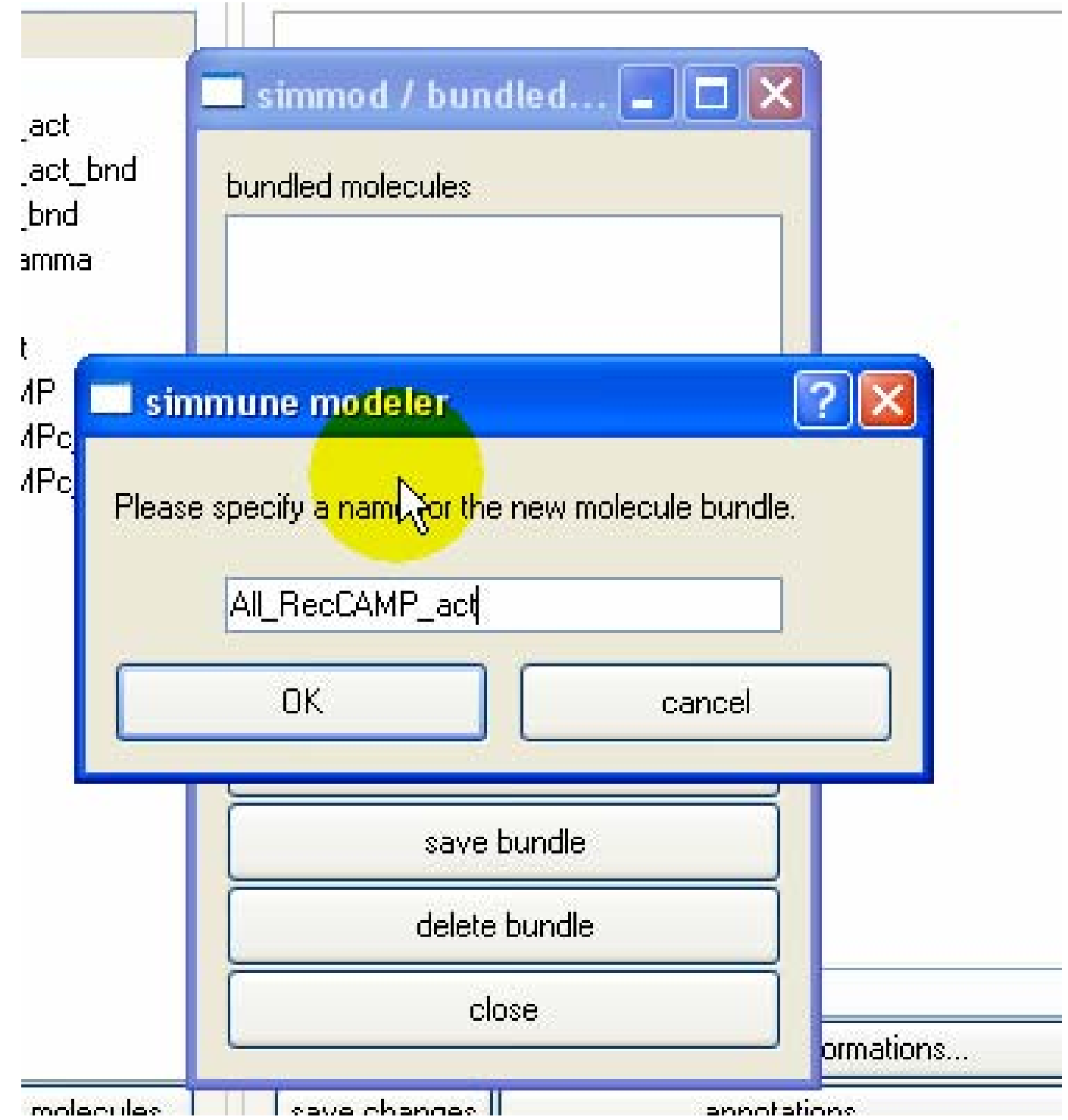

(5) In this bundle, we want to combine all complexes that contain the activated receptor. We therefore call the bundle ‘All_RecCAMP_act’ (the name is, however, arbitrary).

(6) Drag molecules from the ‘defined molecules’ list into the contents list of the selected bundle. Here, we drag only RecCAMPcyto_act. When the contents is complete save the bundle.

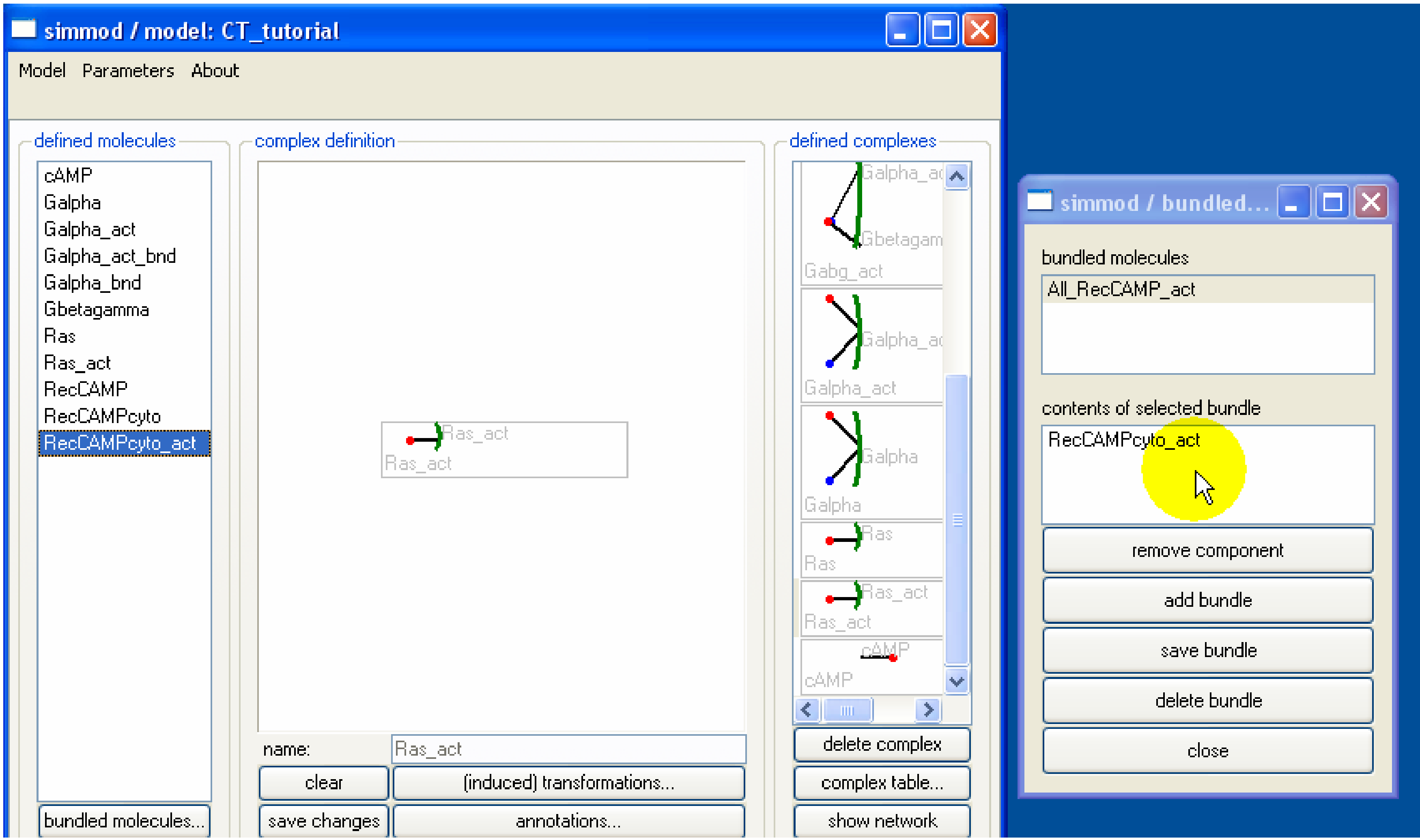

(7) Now, define the cell type of our model. Go to the 'cells' tab and click 'new cell type'. Then, define a name for the cell type.

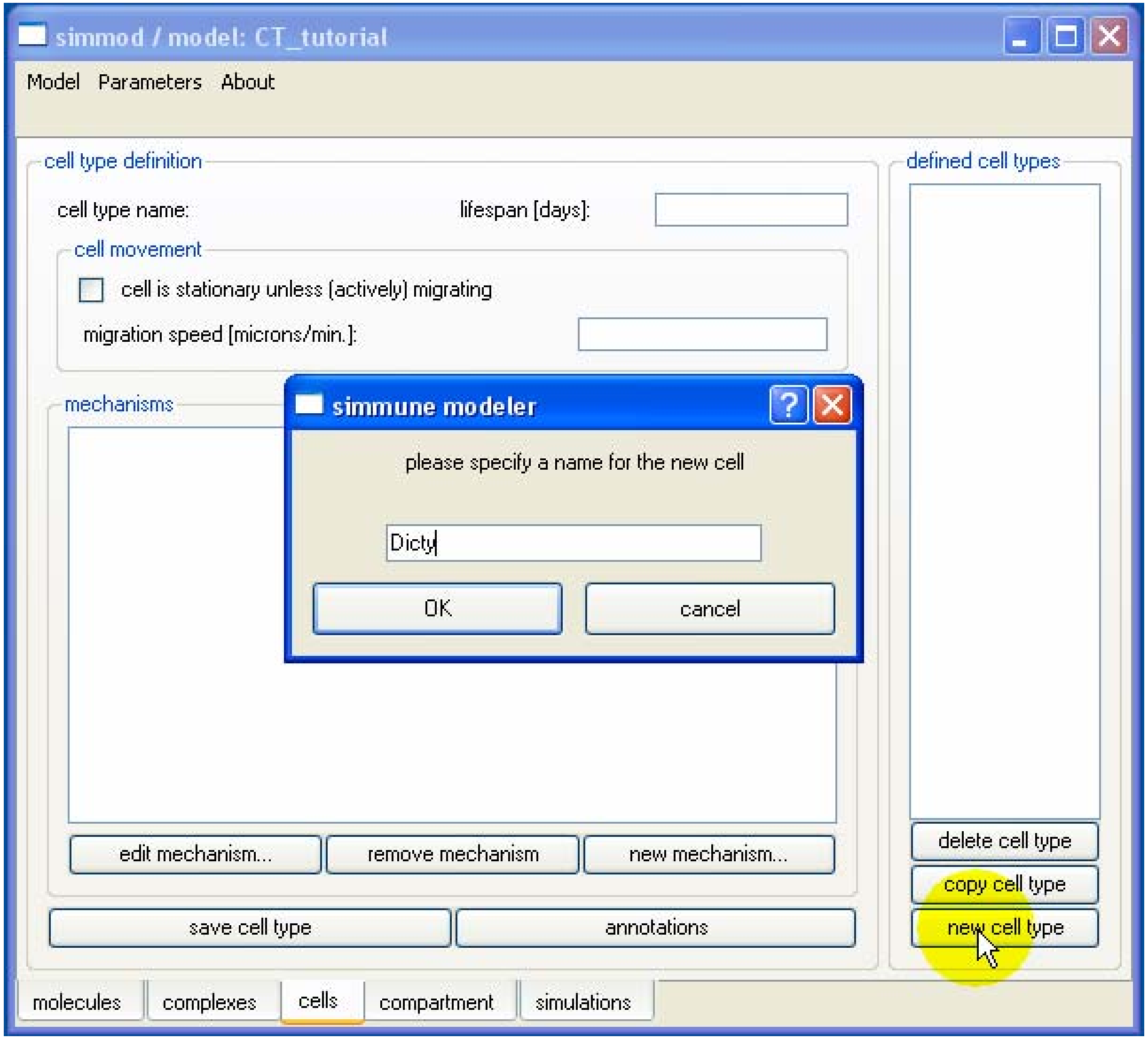

(8) Double-click the cell type name to edit its properties.

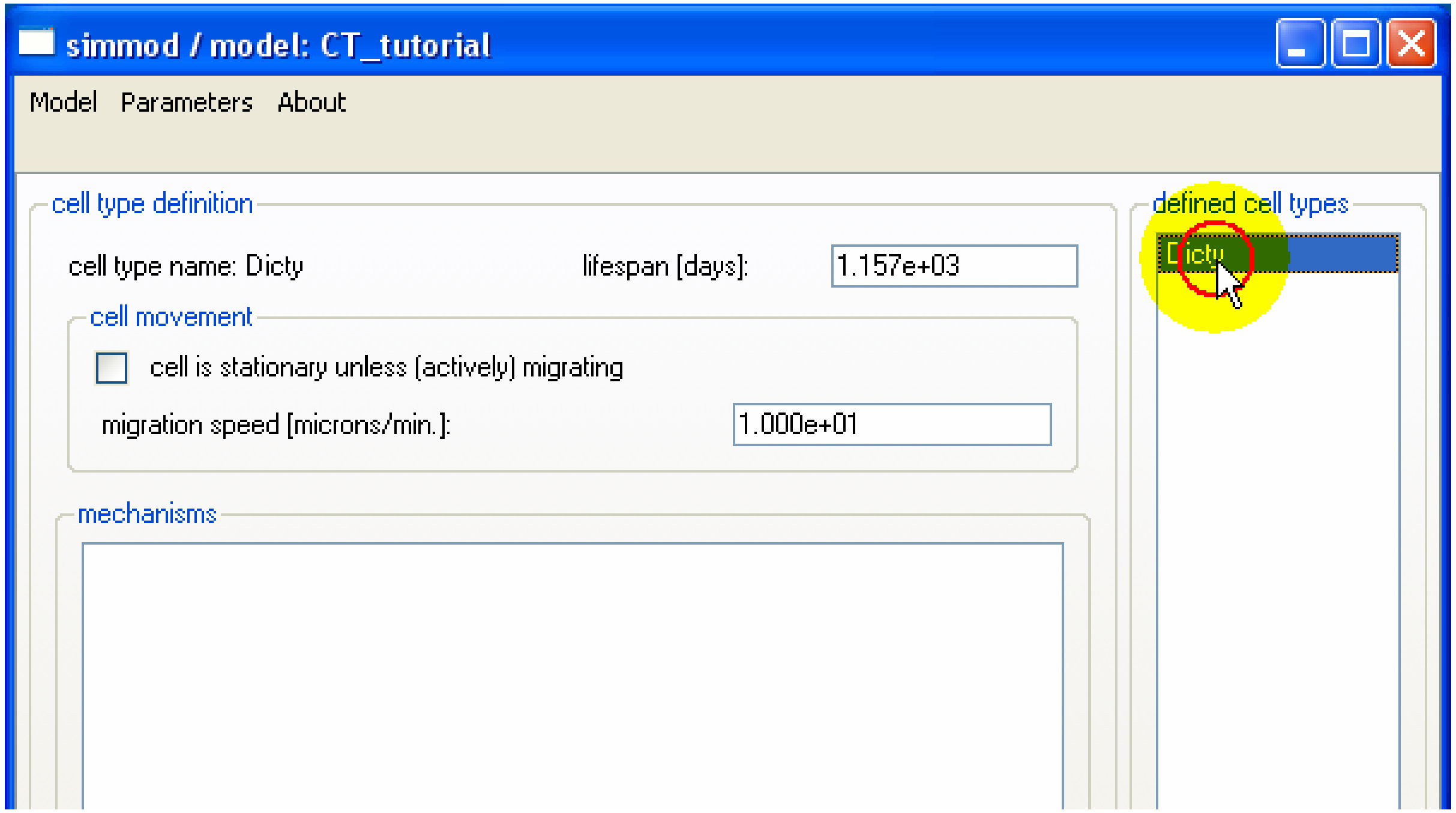

The basic properties are lifespan, migration speed (when actively migrating), and whether the cells should randomly move, when not actively migrating.

(9) Click on 'new mechanism' in the cells tab to open the window for the definition of cellular mechanisms of behavior. (See documentation for details on the concept of cellular mechanisms in Simmune.)

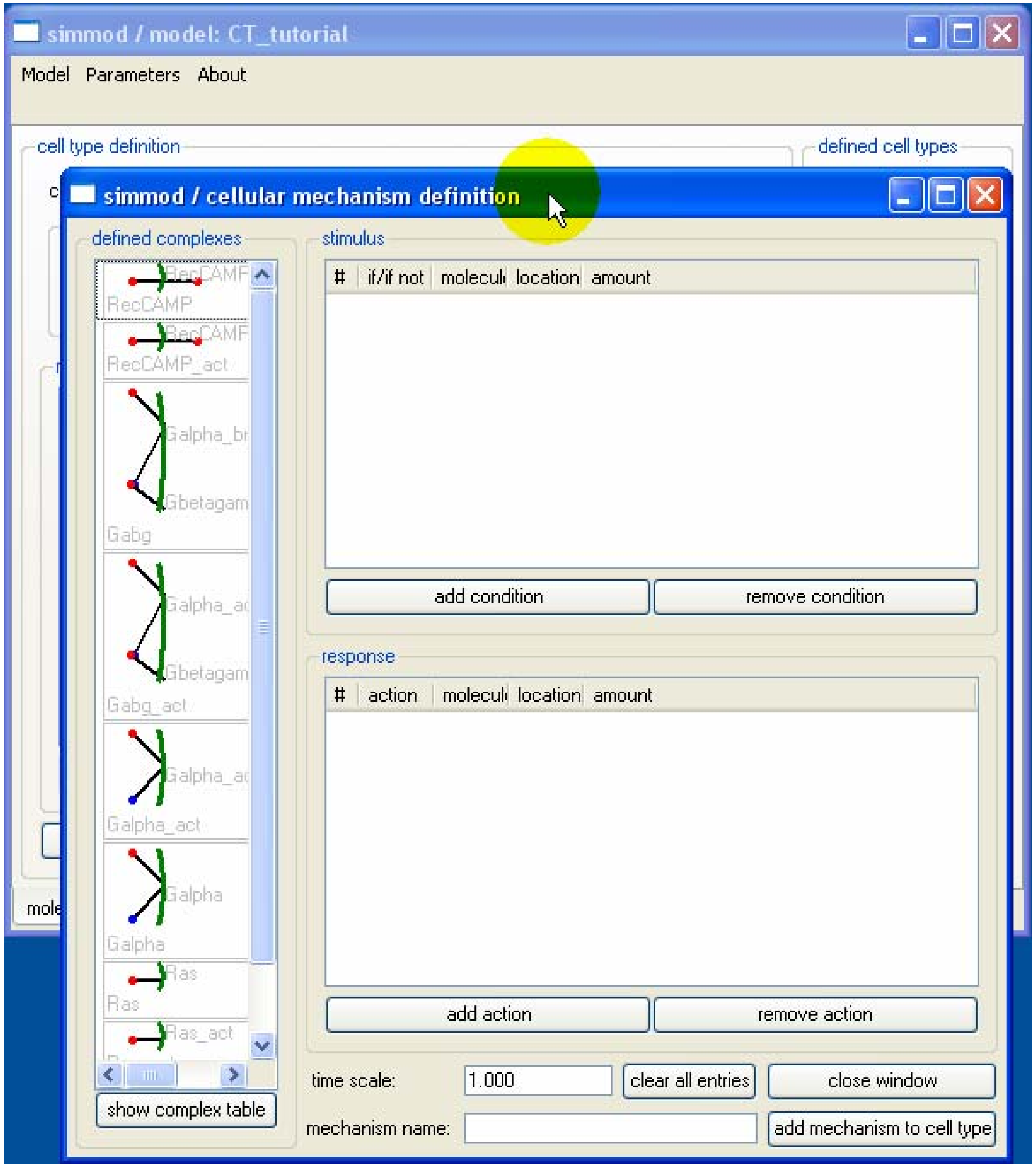

(10) Click on ‘add condition’, then select the condition (here we have only condition ‘0’). Then click on the list header segment ‘if/if not’ to select the type of condition. Choose ‘INITIALLY’.

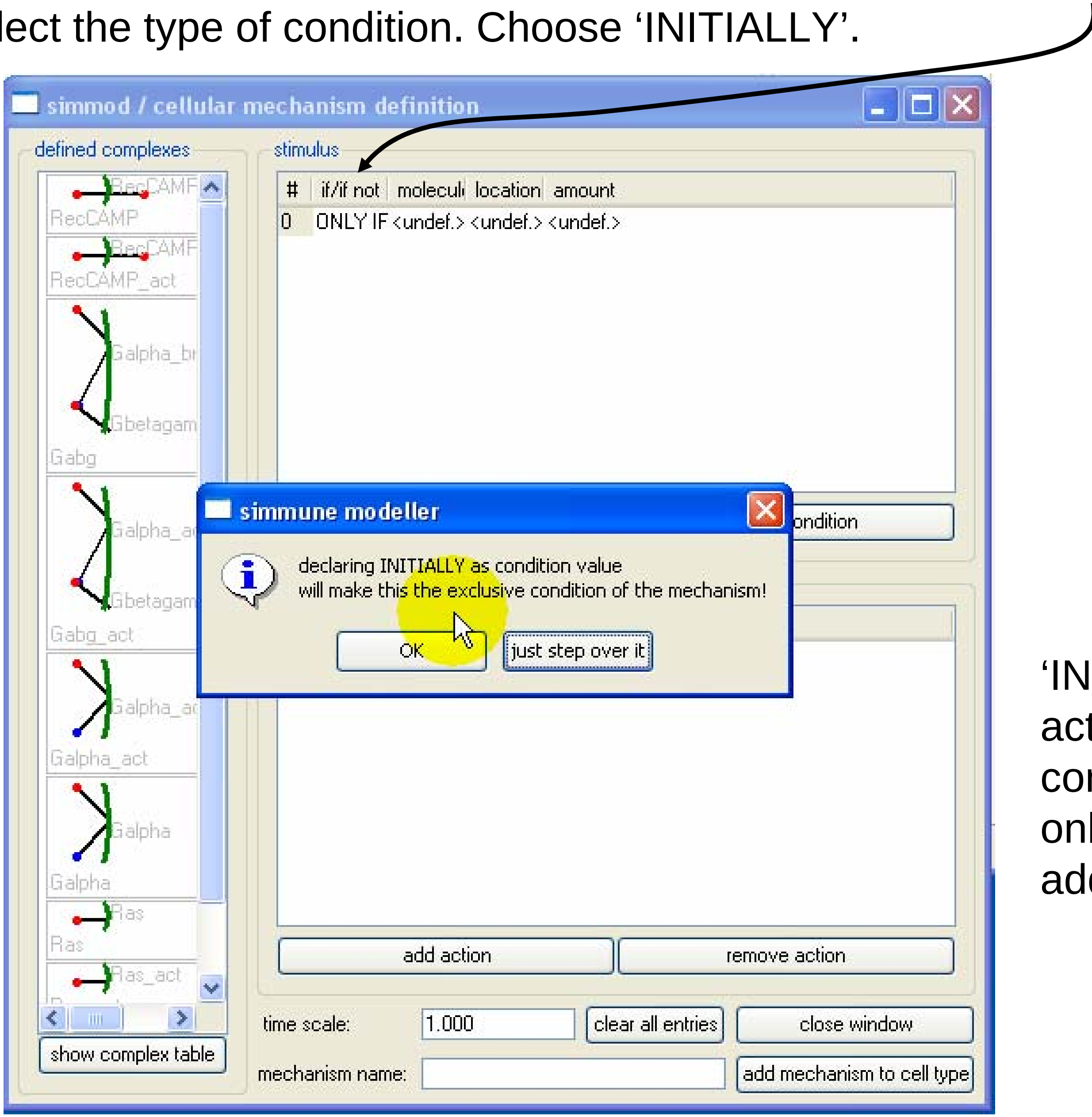

‘INITIALLY’ means that the actions related to this condition will be executed only once, when the cell is added to the simulation.

(11) Then, click ‘add action’ and, by clicking the ‘action’ segment of the list header, open the action selection window.

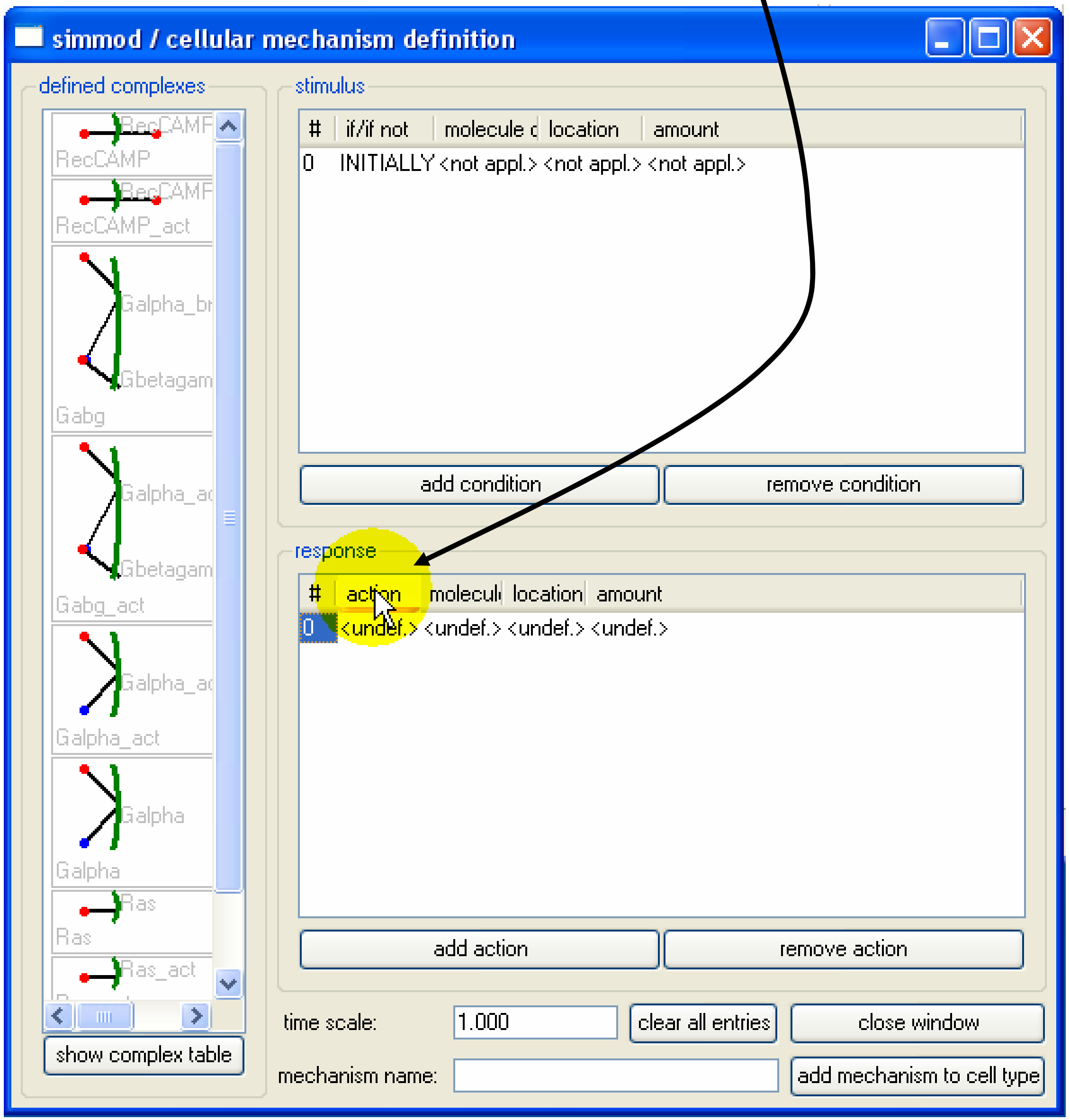

(12) Select the action ‘display membrane molecule complex’.

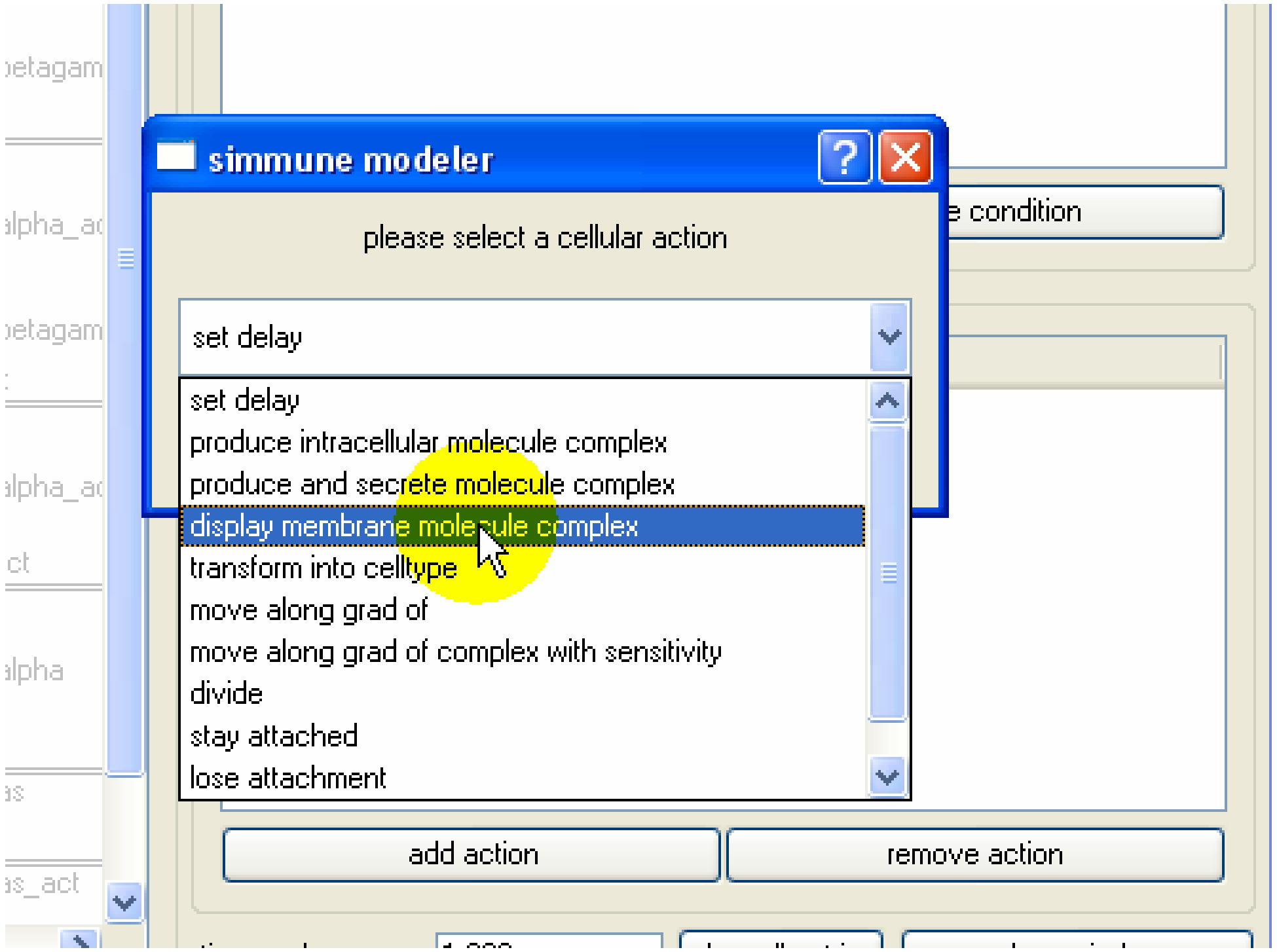

(13) Drag the RecCAMP complex into the selected action. (Attention: editing actions or conditions requires that them to be selected.)

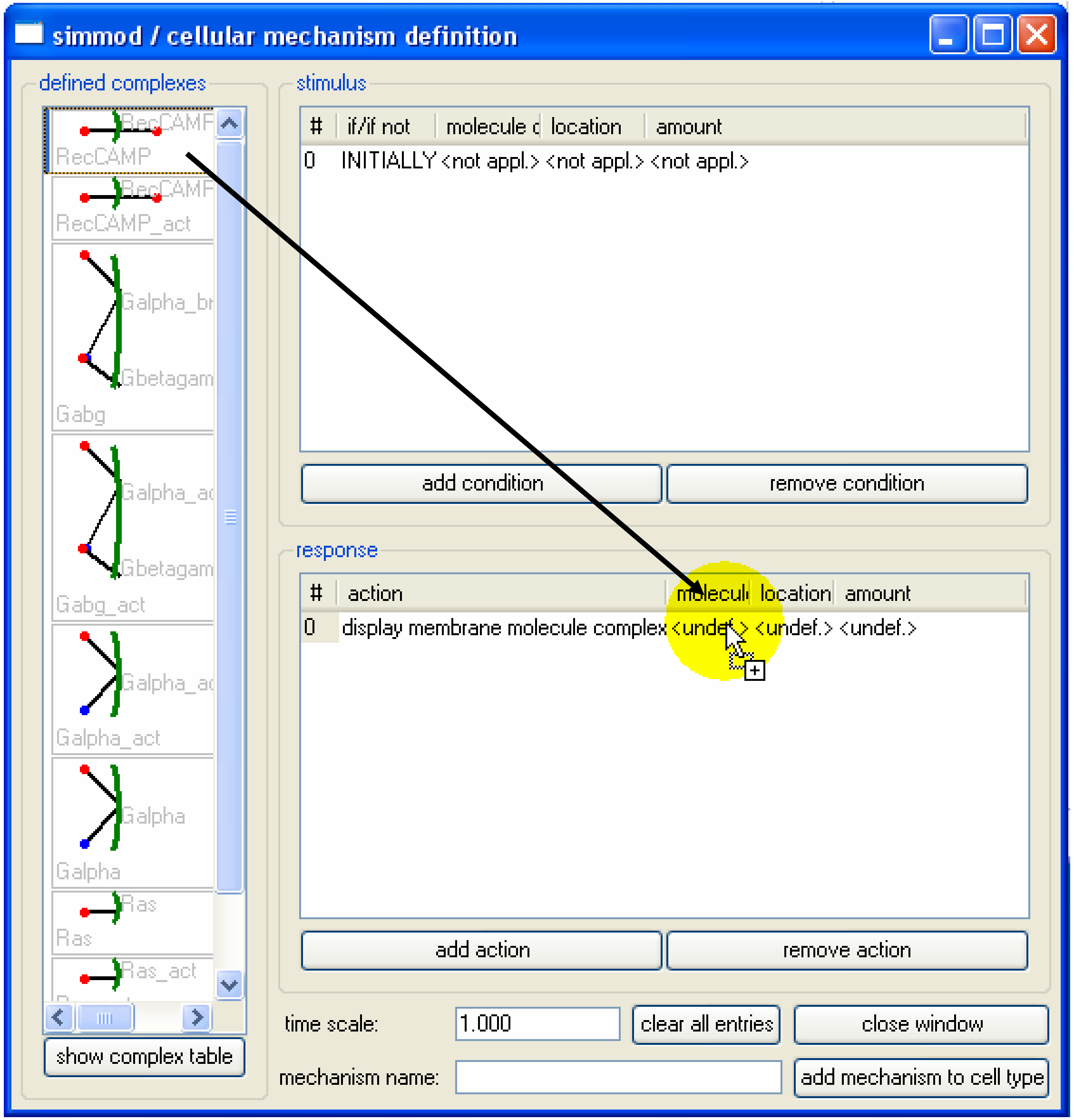

(14) Click the ‘location’ segment of the action list header to cycle through the location options for adding the membrane complex. Select ‘every side’.

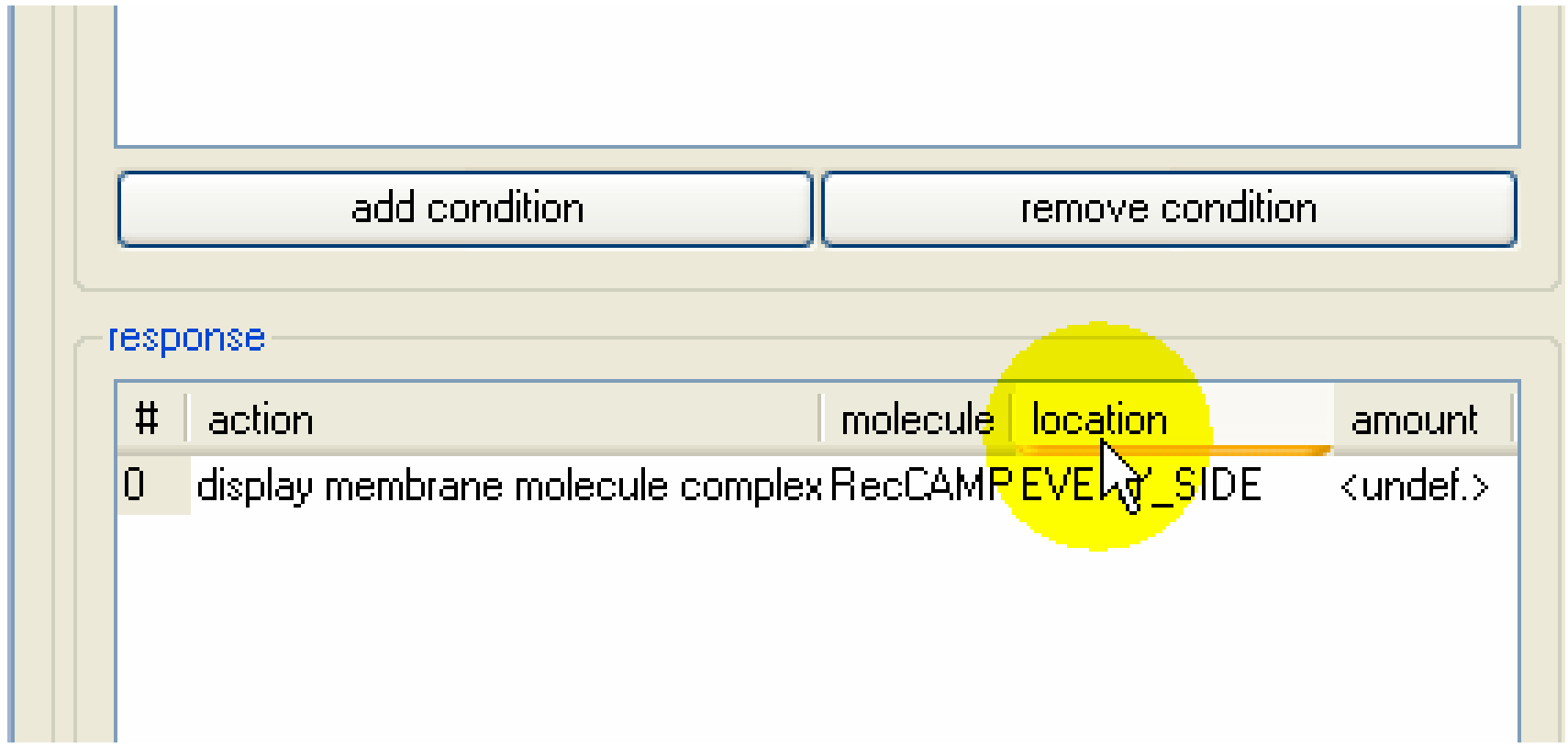

(15) Click the ‘amount’ segment of the action list header to enter the amount of molecule complexes to be added to the cell surface (per cell side – see documentation).

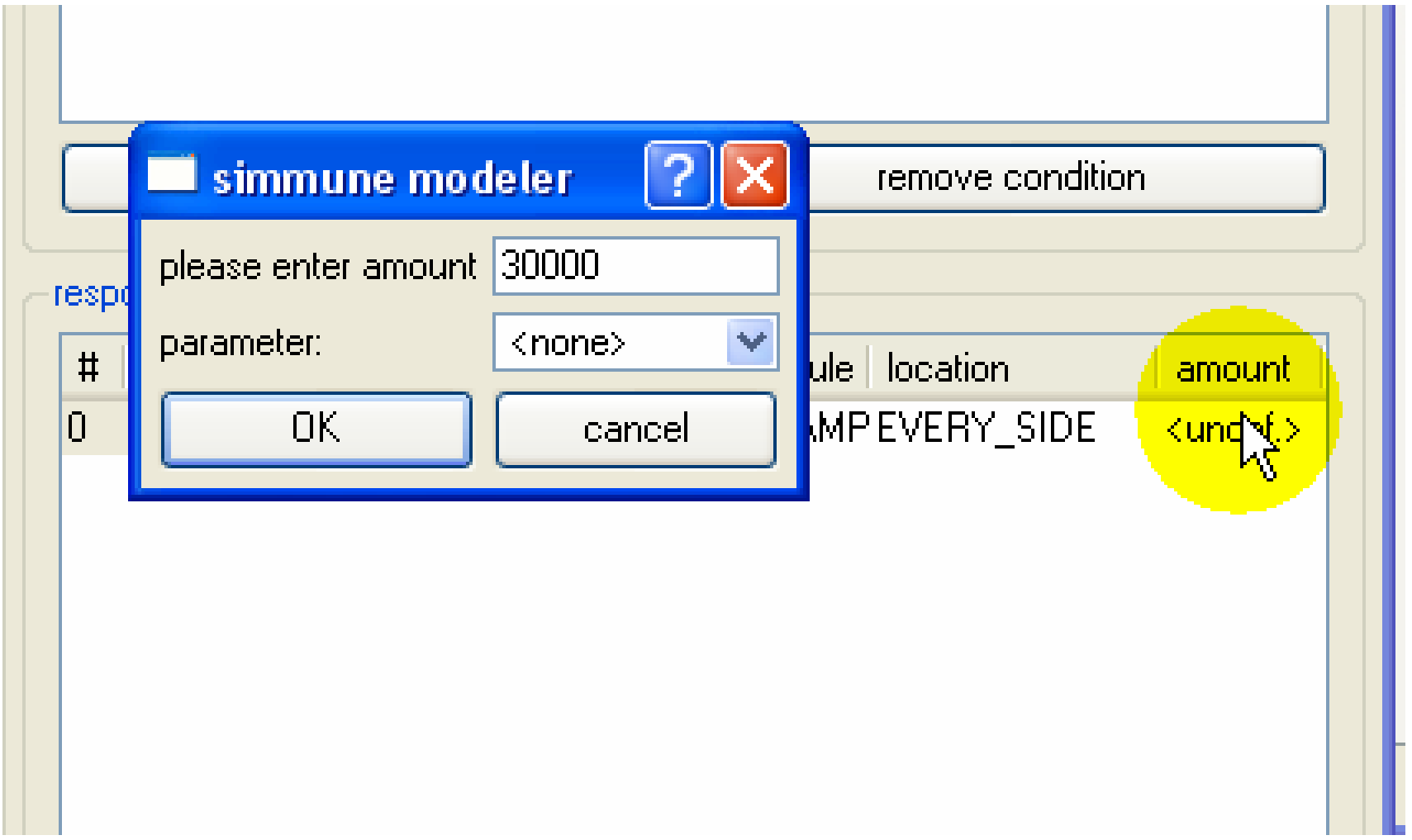

(16) Analogously, define an action for the production of the intracellular complex Gabg.

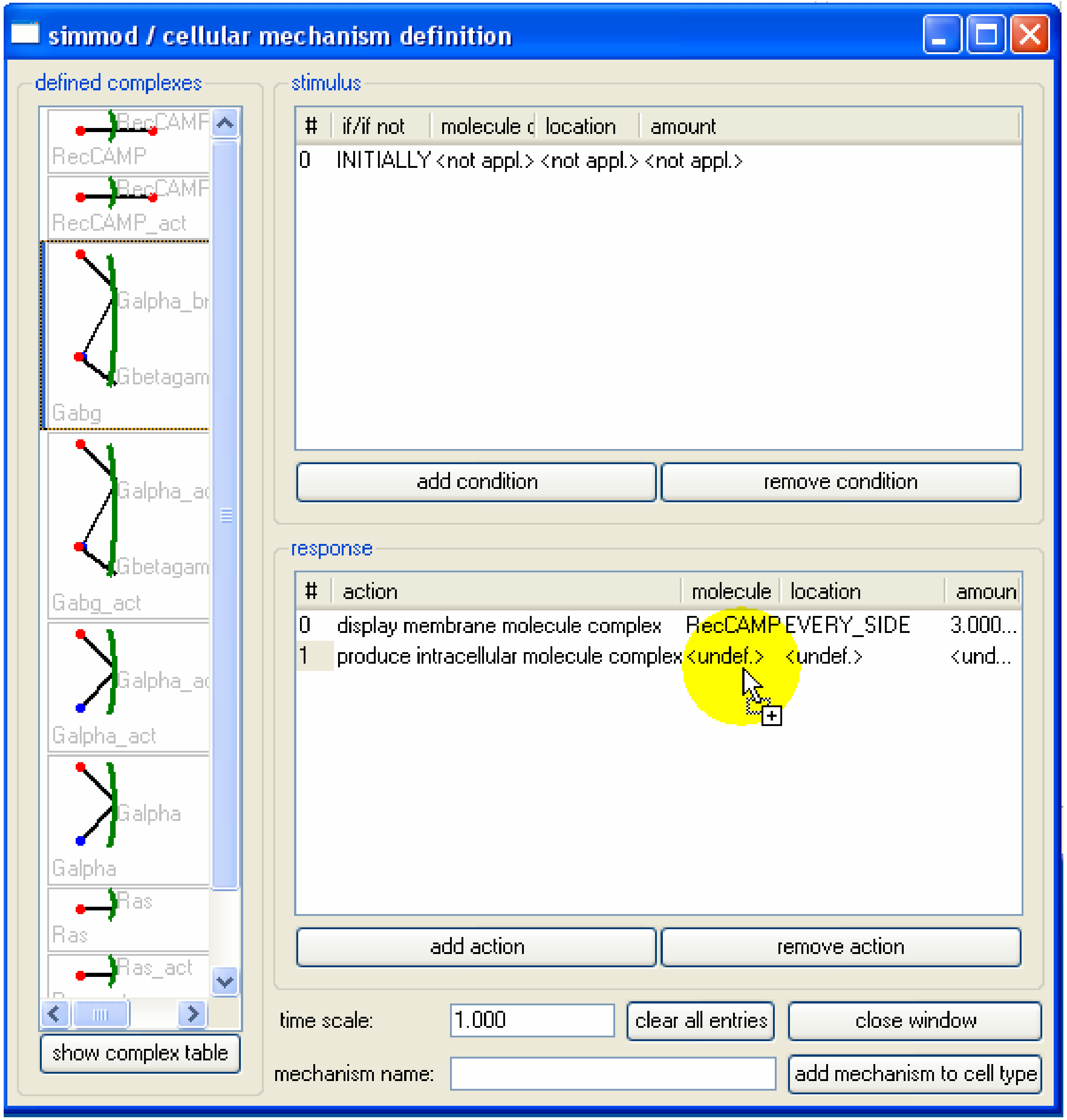

(17) Define the location for the complex Gabg (‘under surface’).
Then, the amount (50,000 per side).

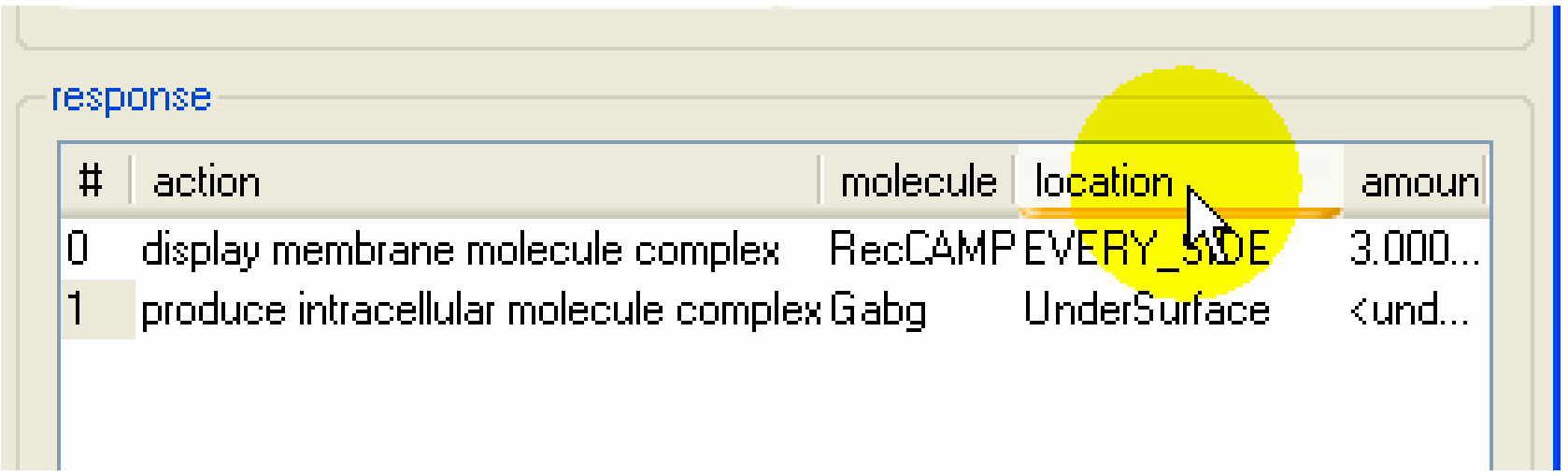

(18) Same for Ras.

This completes the setup of the initial intracellular biochemistry of the Dicty cell type.

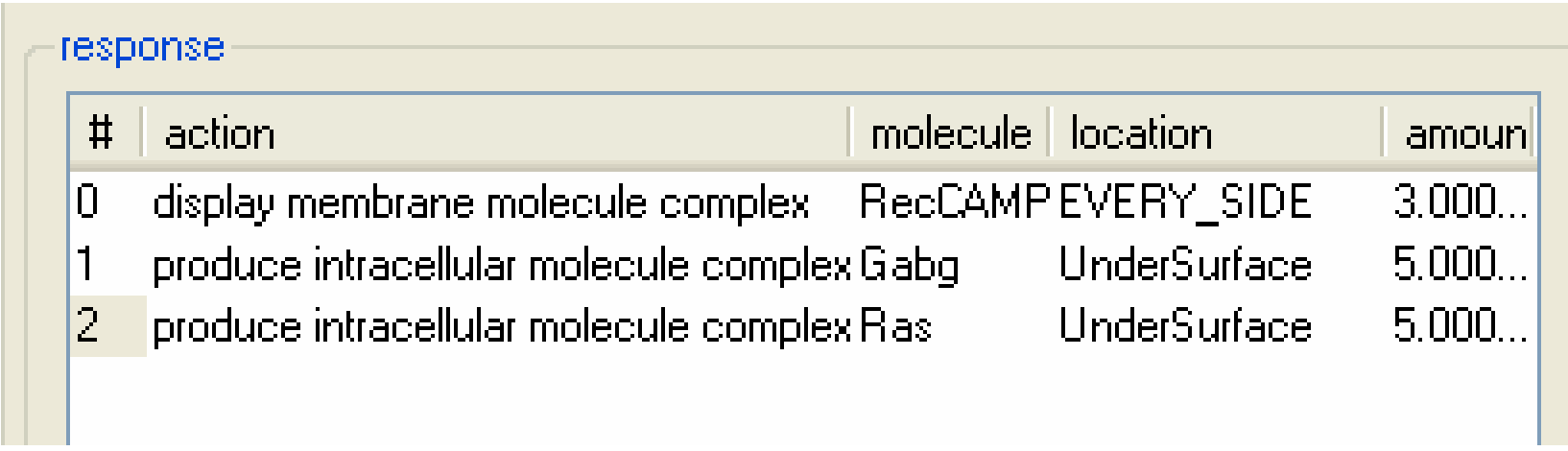

(19) Define a name for the mechanism and add it to the list of mechanisms of the Dicty cell type.

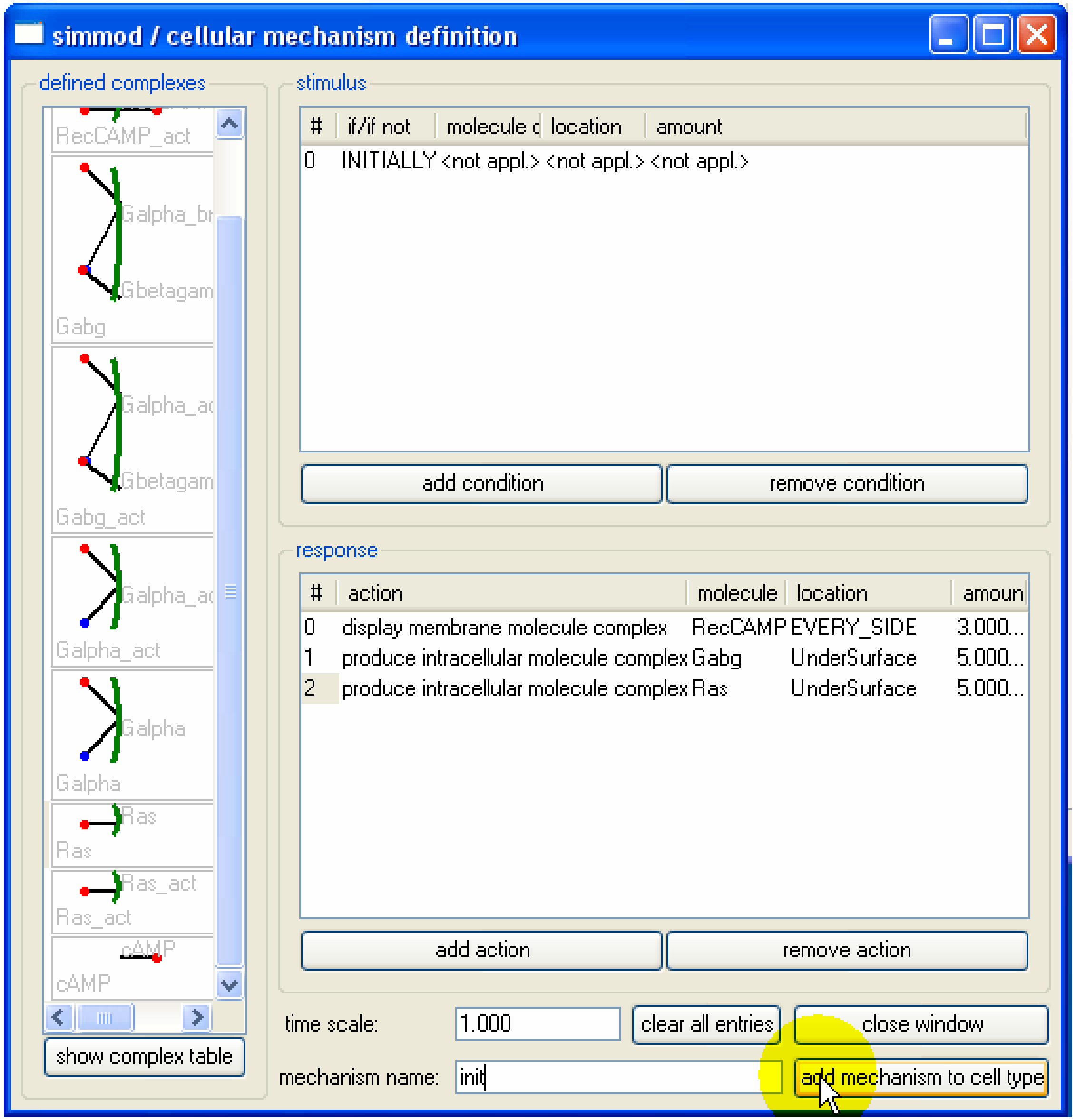

(20) Save the properties of the Dicty cell type.

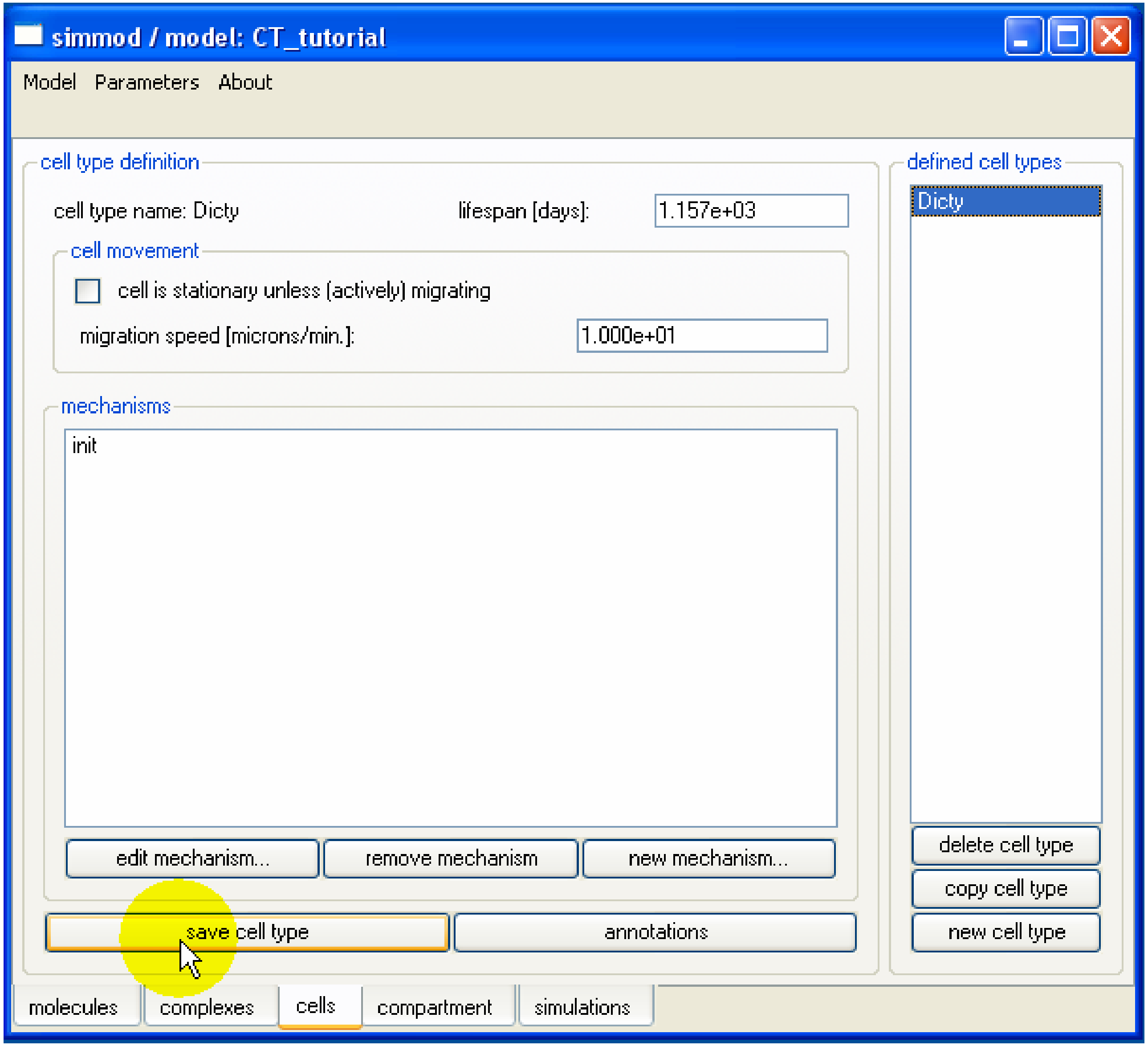

(21) Go to the ‘compartment’ tab, set the desired dimensions of the extracellular compartment and save its properties.

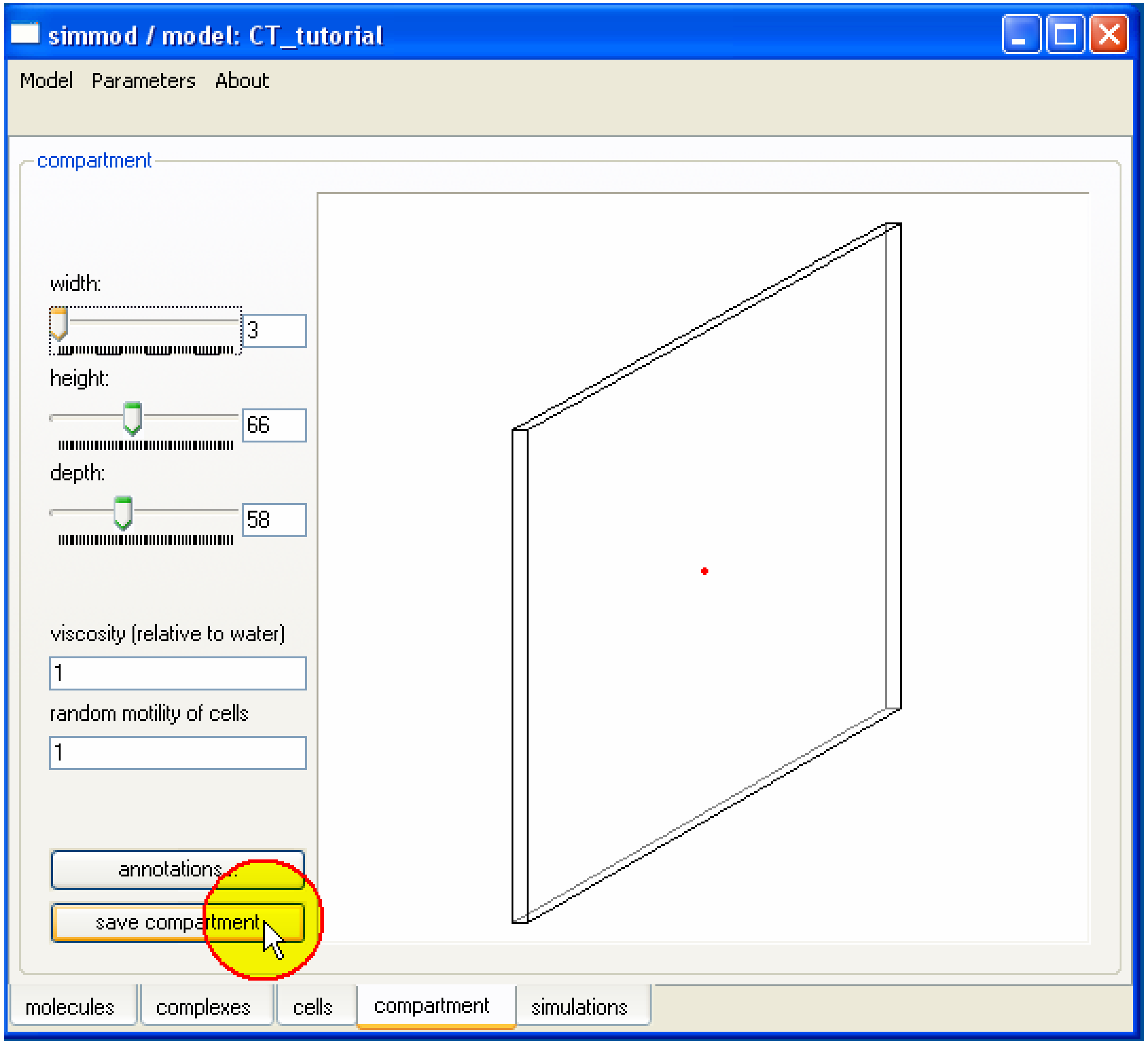

(22) Finally, we go to the ‘simulations’ tab to define a simulation.

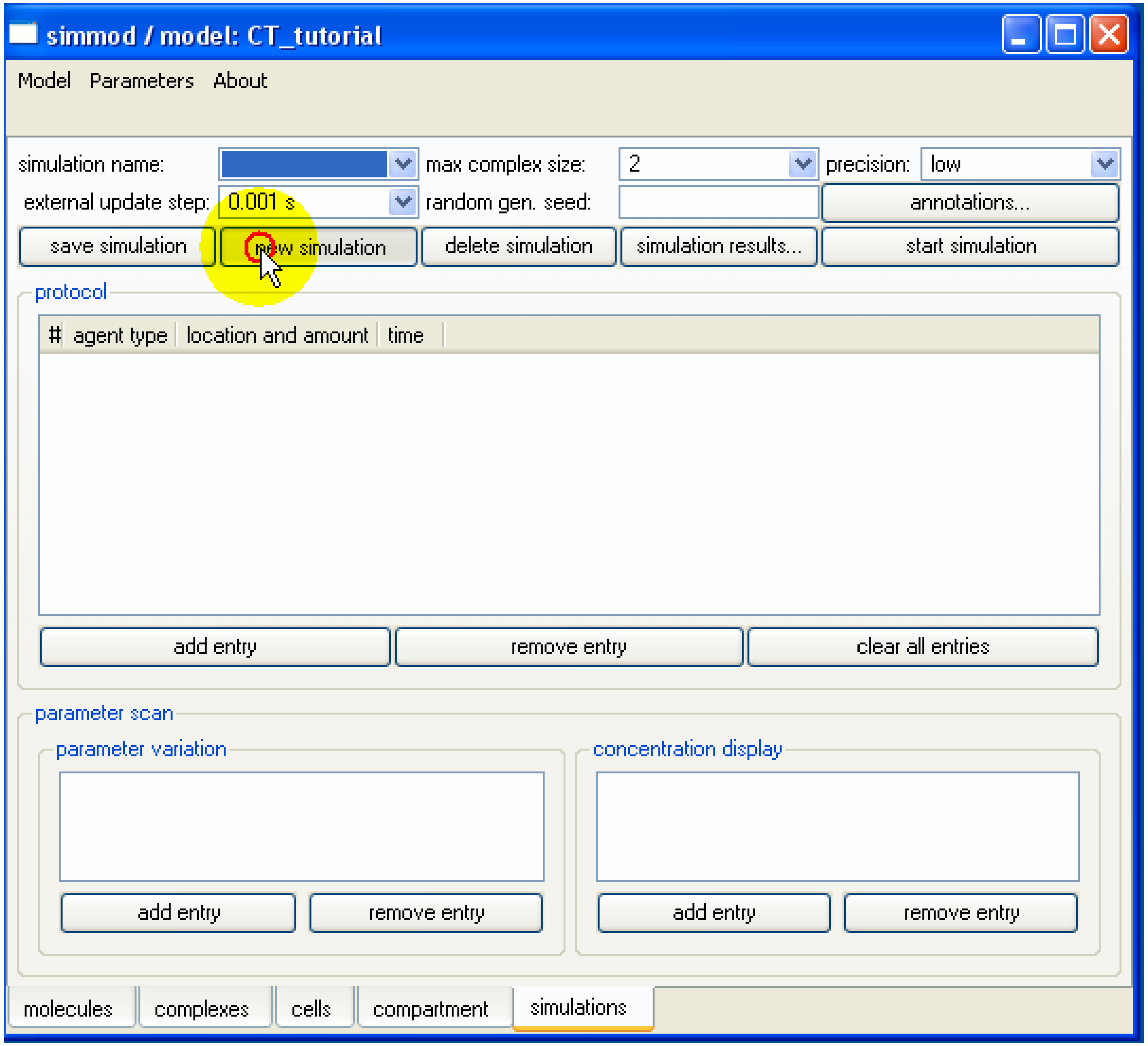

(23) Define a name for the simulation. And save the simulation properties (important !).

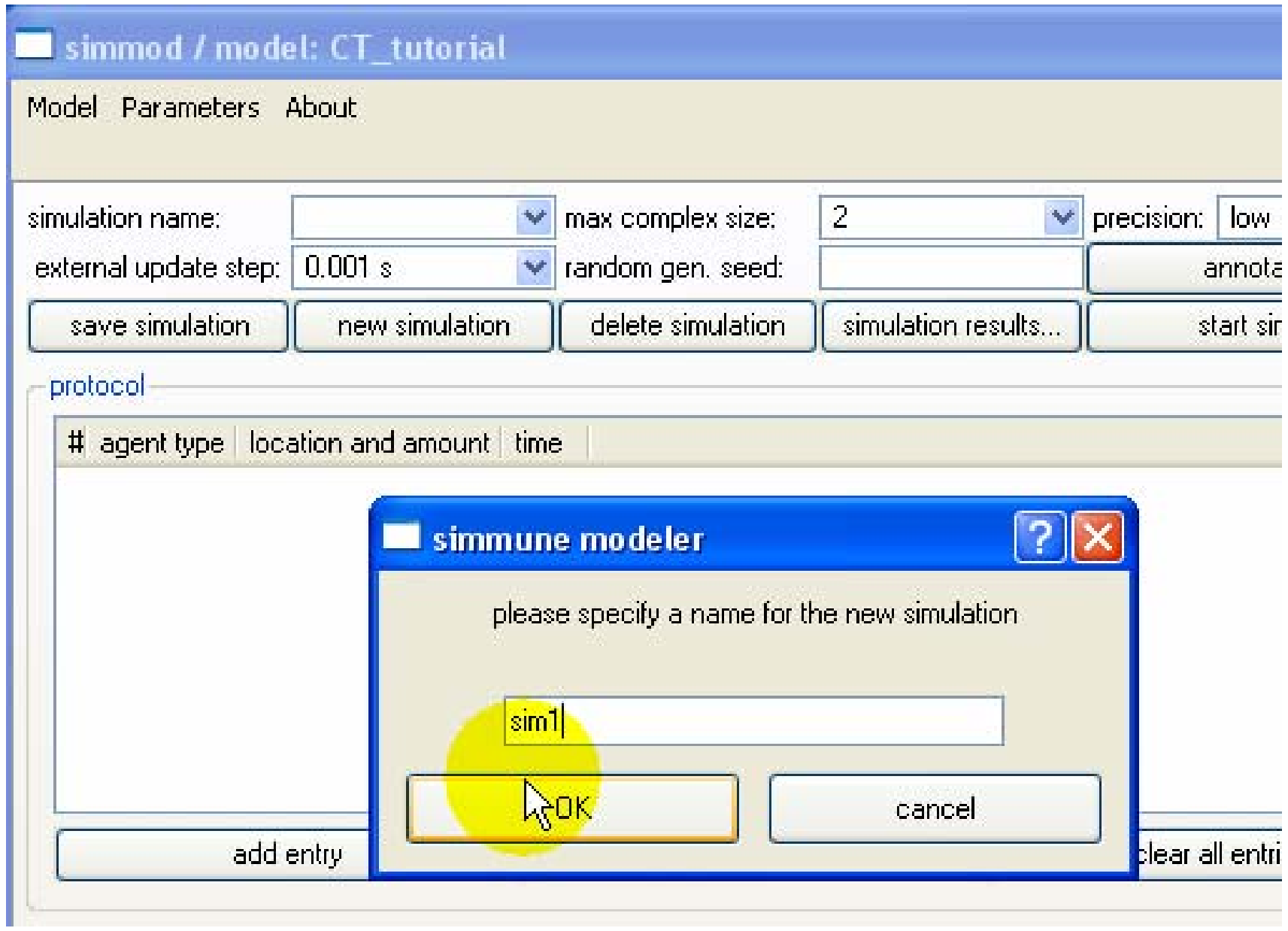

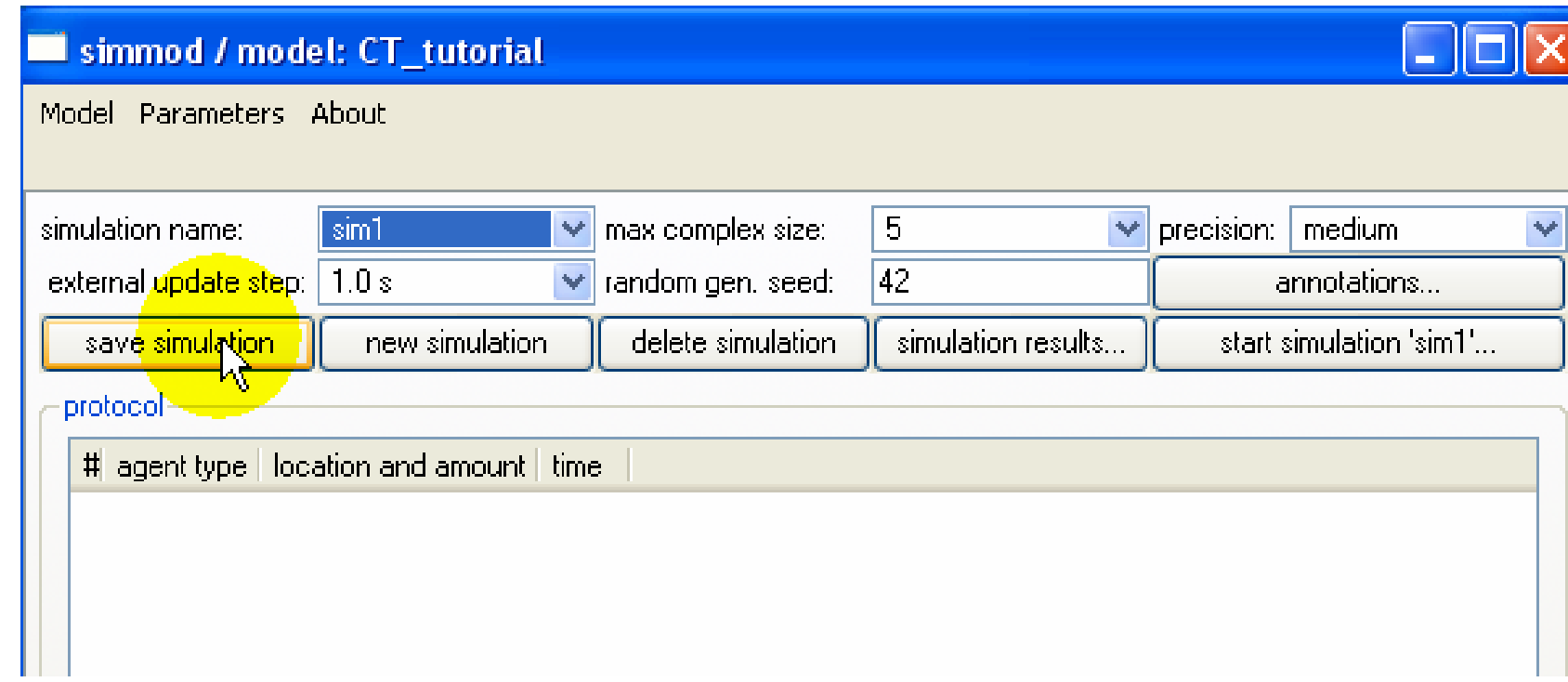

(24) Start the simulation.

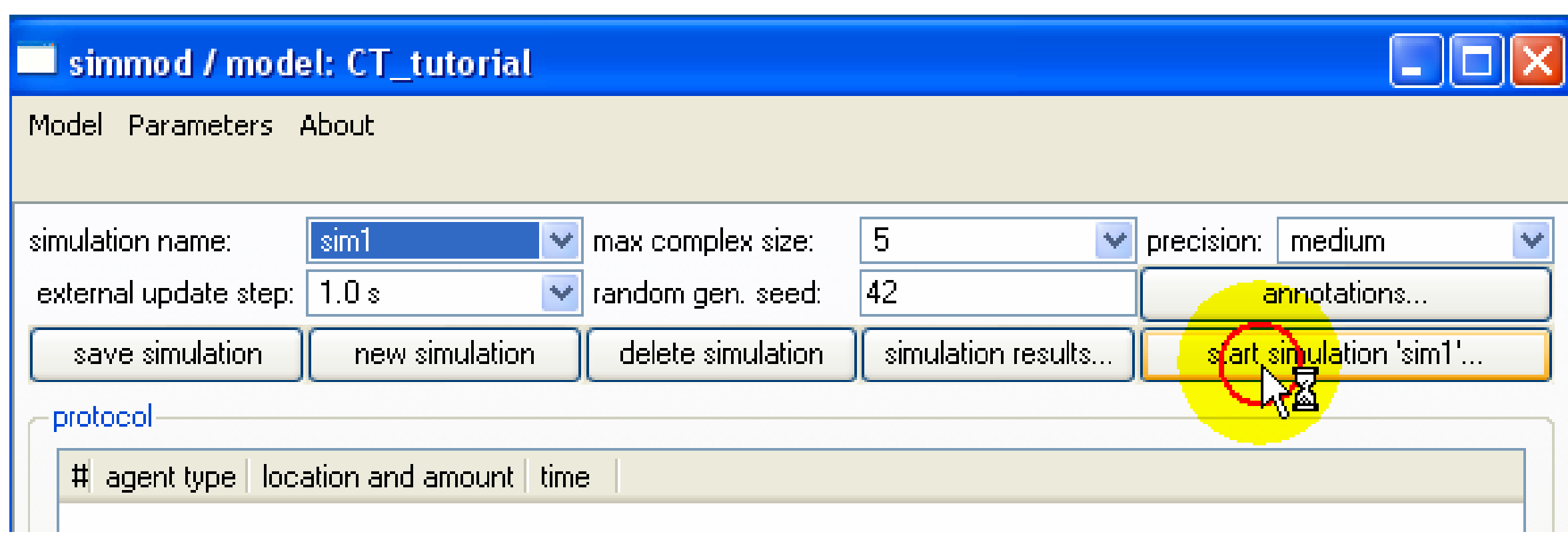

The simulation window has three views of the main simulation:

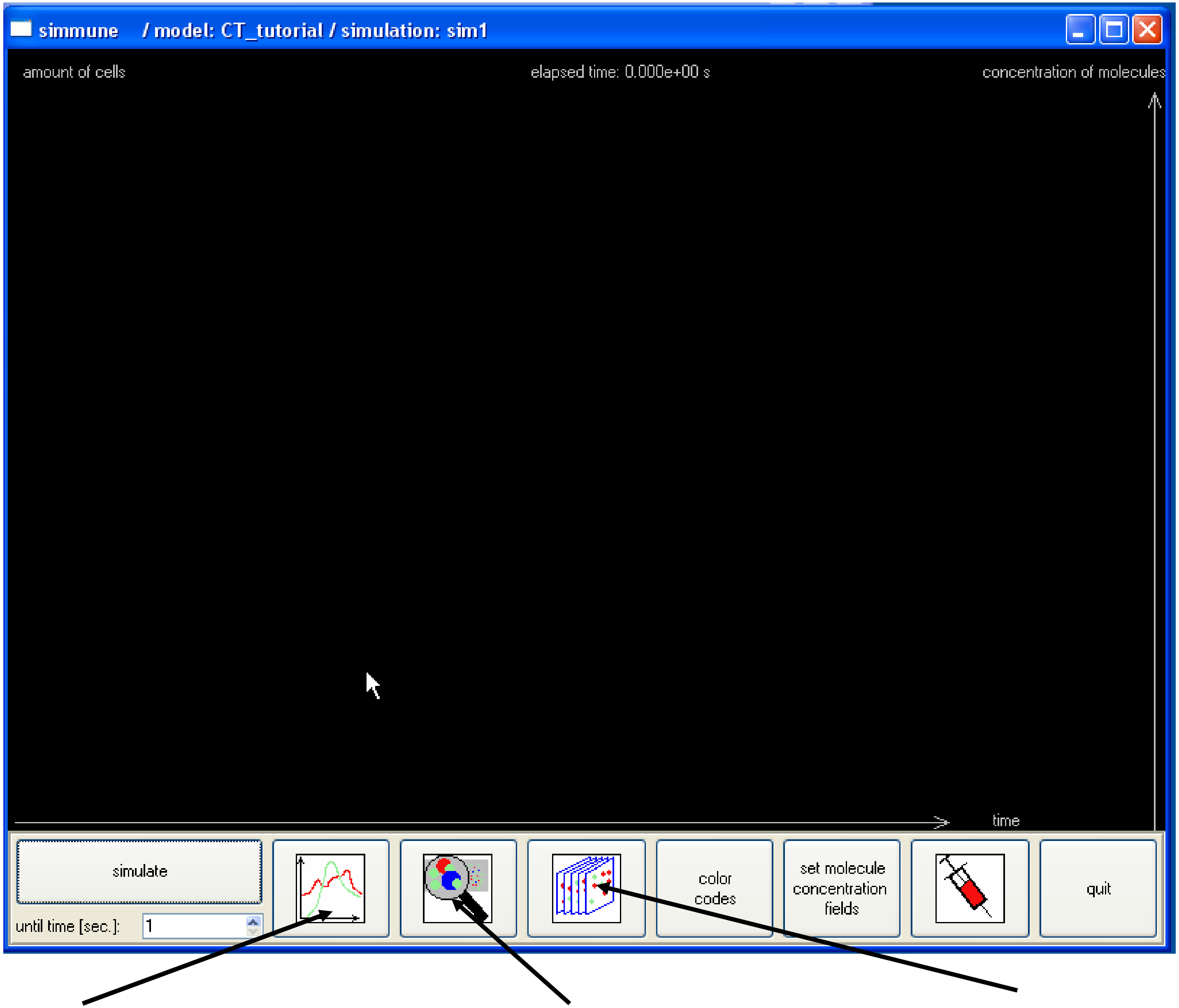

Time course view, the (pseudo-3D) compartment view, the compartment slice view

(25) Go to the slice view to inject a cell:

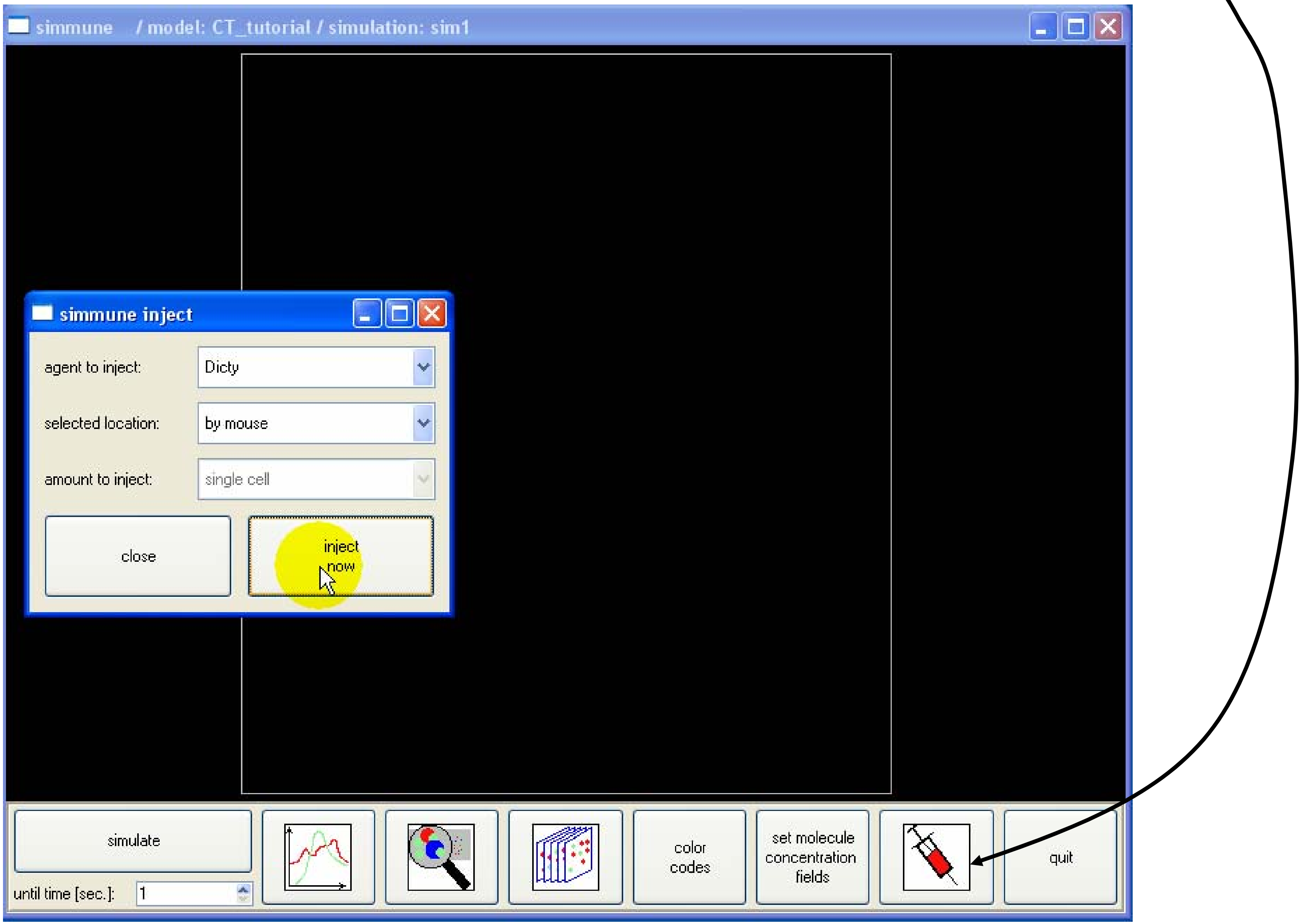

(26) Click on the compartment slice to place the cell into the compartment. The software will automatically assign a color code to the injected type if it is new to the simulation.

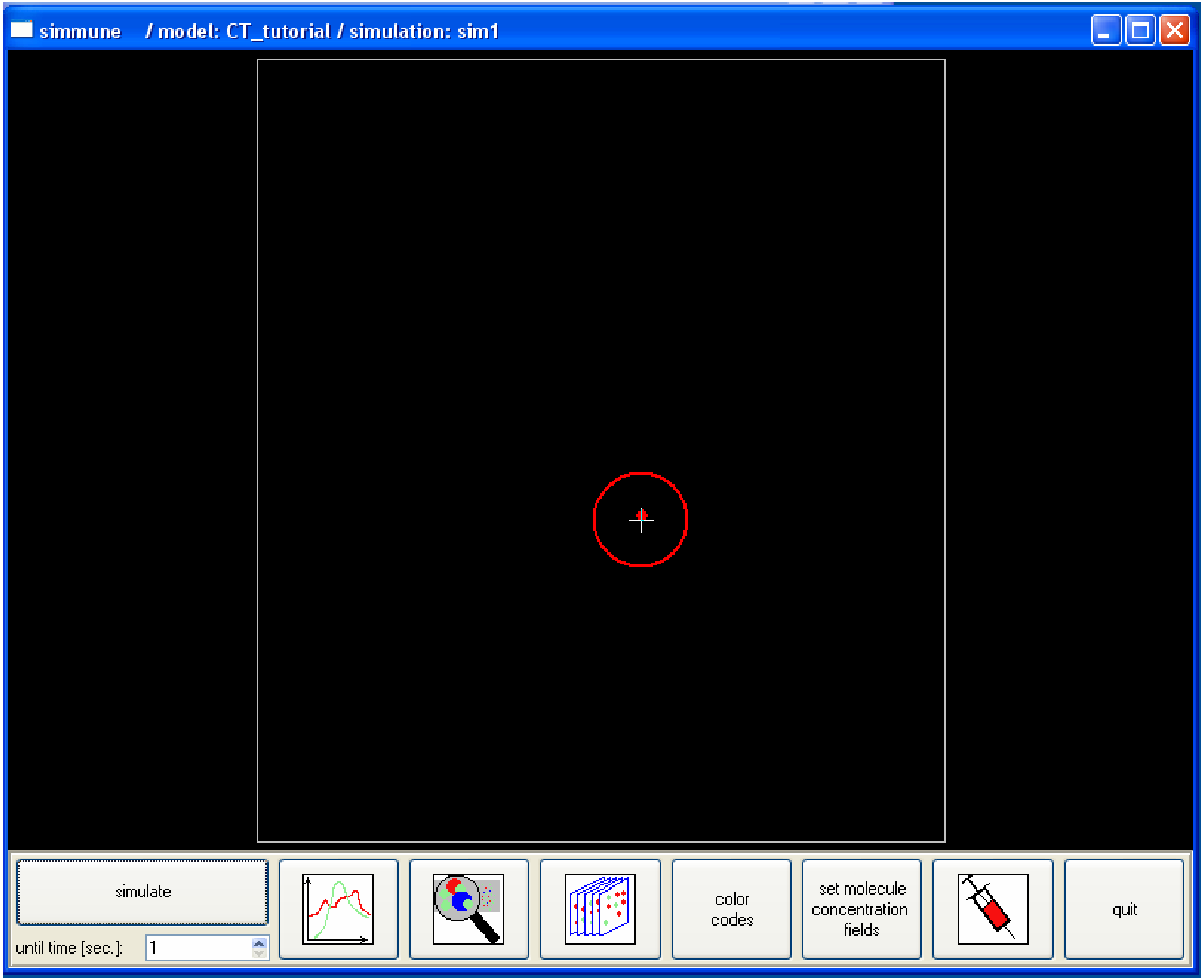

(27) Click on the circle representing the cell in the compartment and open a new cell data display.

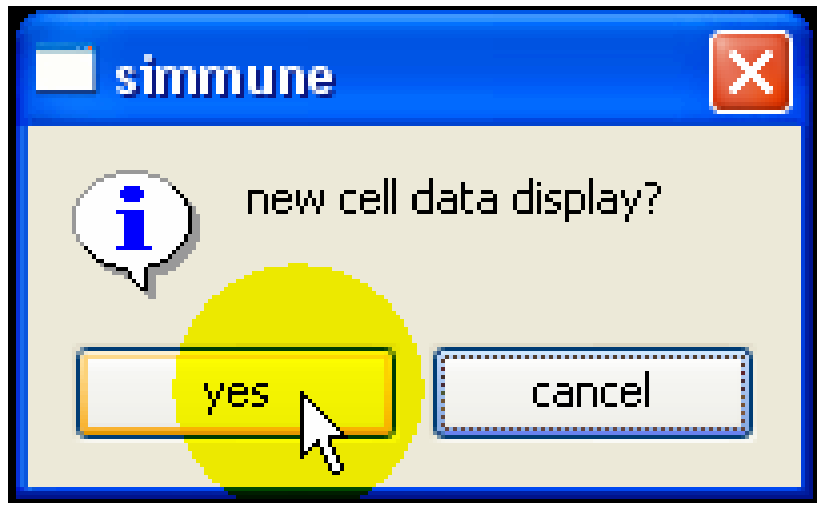

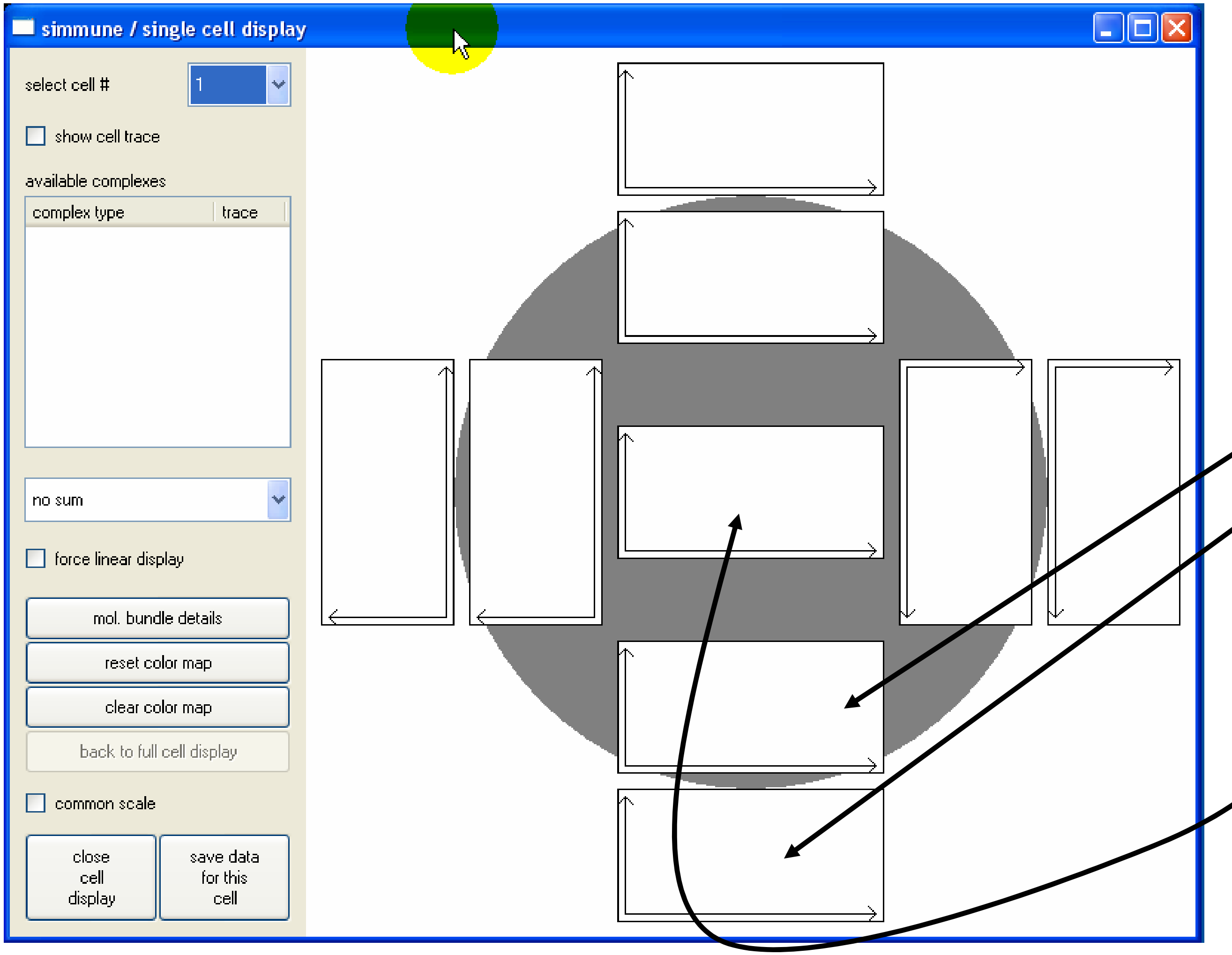

The cell is represented in 2D (a slice through the cell). The display shows four (of the six peripheral regions of the cell, separated into intracellular and membrane domain. In the center is the center cytosolic region.

(28) After some time of simulation, the cell display will show the complexes that exist in the cell and that have been defined as complexes or bundles to be tracked.

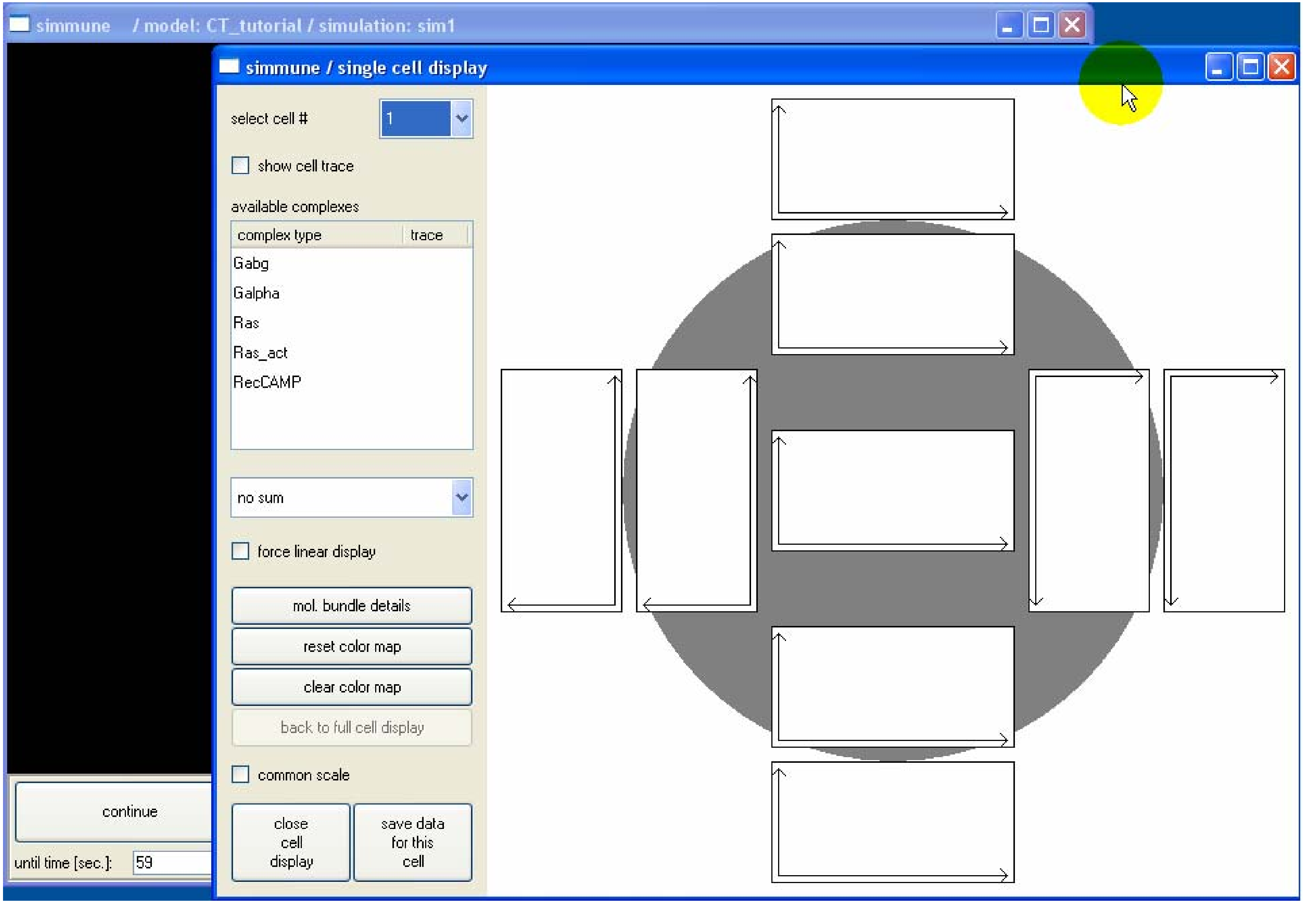

(29) Double-click an entry to associate a color code with the complex or bundle and view the time course of its concentration in the different regions of the cell.

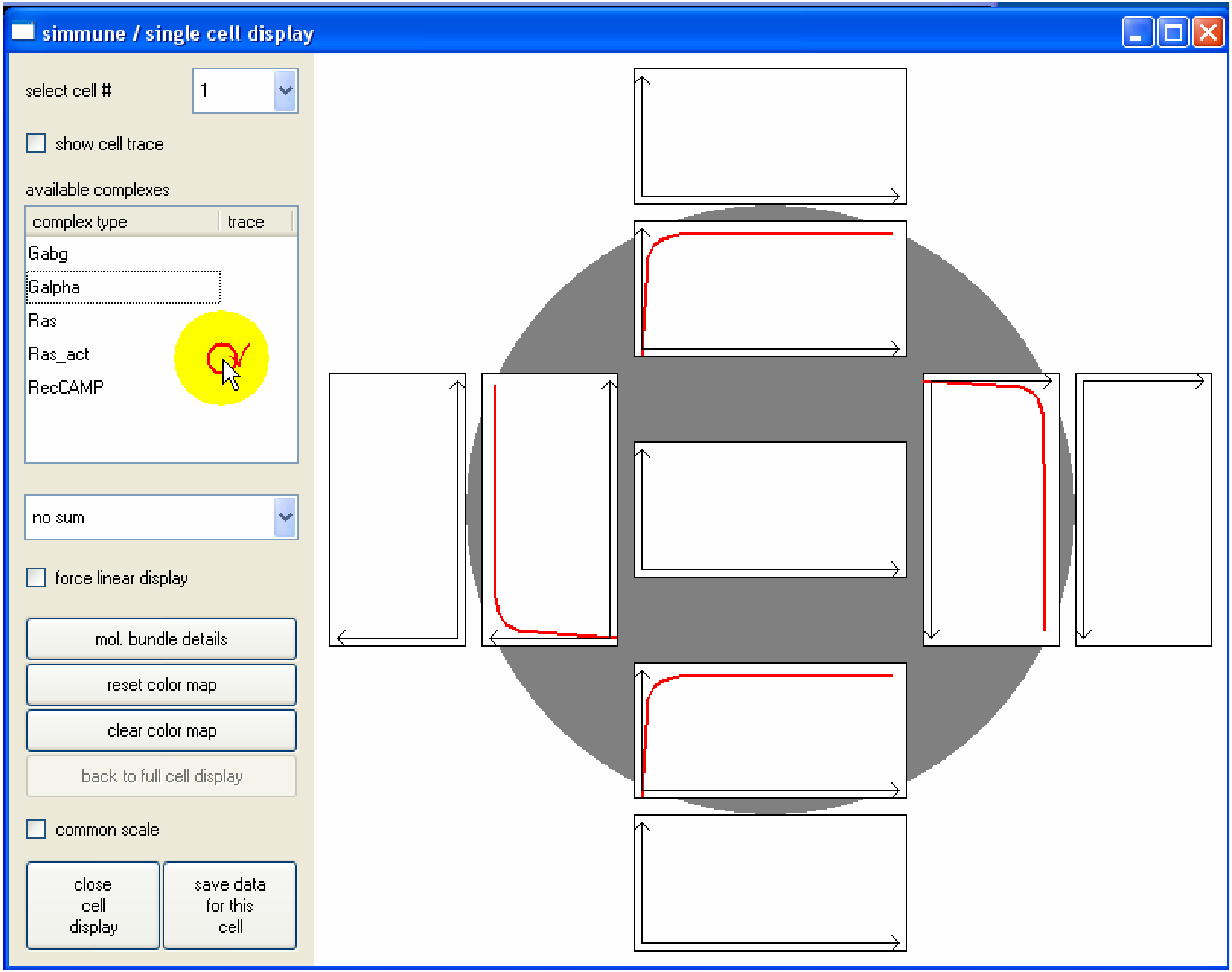

(30) Double-click on a cell region to obtain more quantitative information about the biochemistry in that region.

(31) Clicking the ‘save data for this cell’ button allows you to save the simulated signal flow through the reaction network (for the whole cell, not only the selected region).

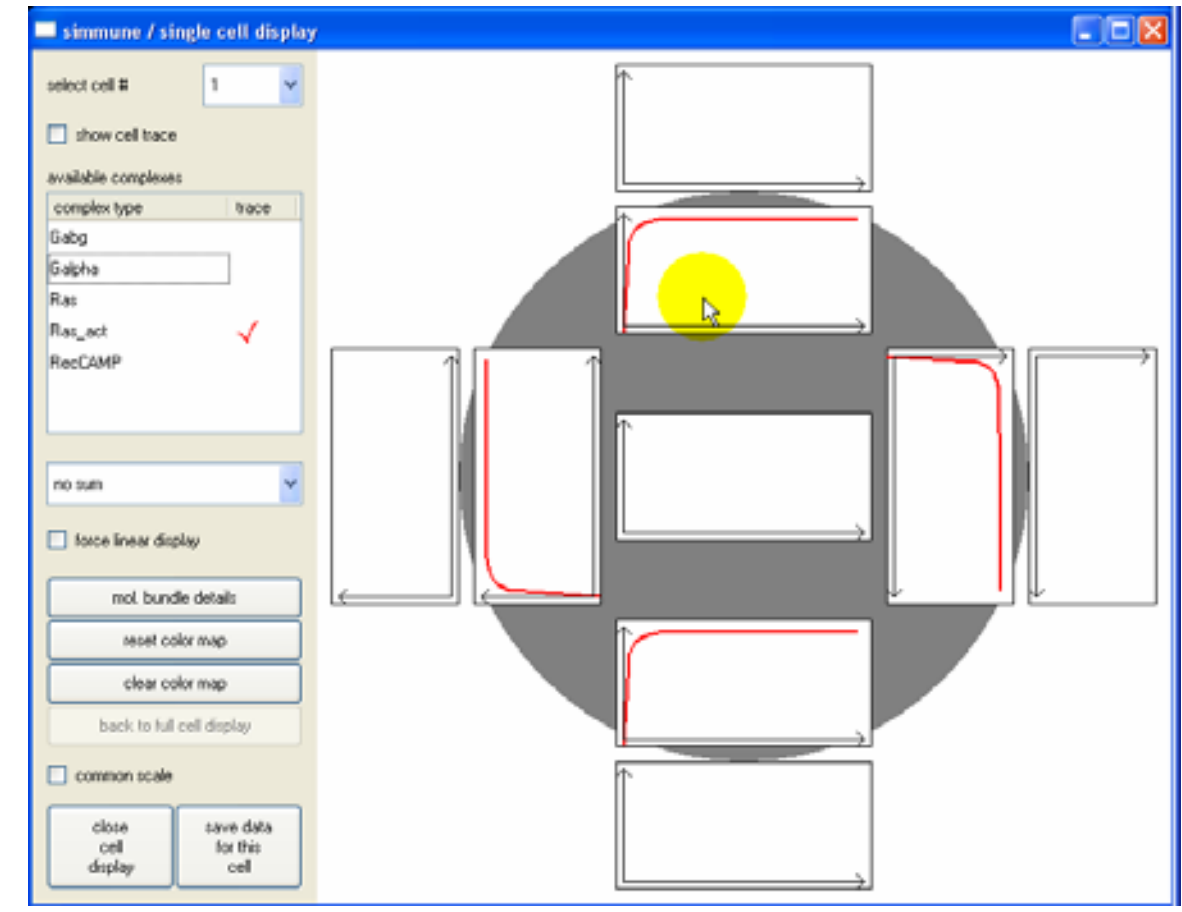

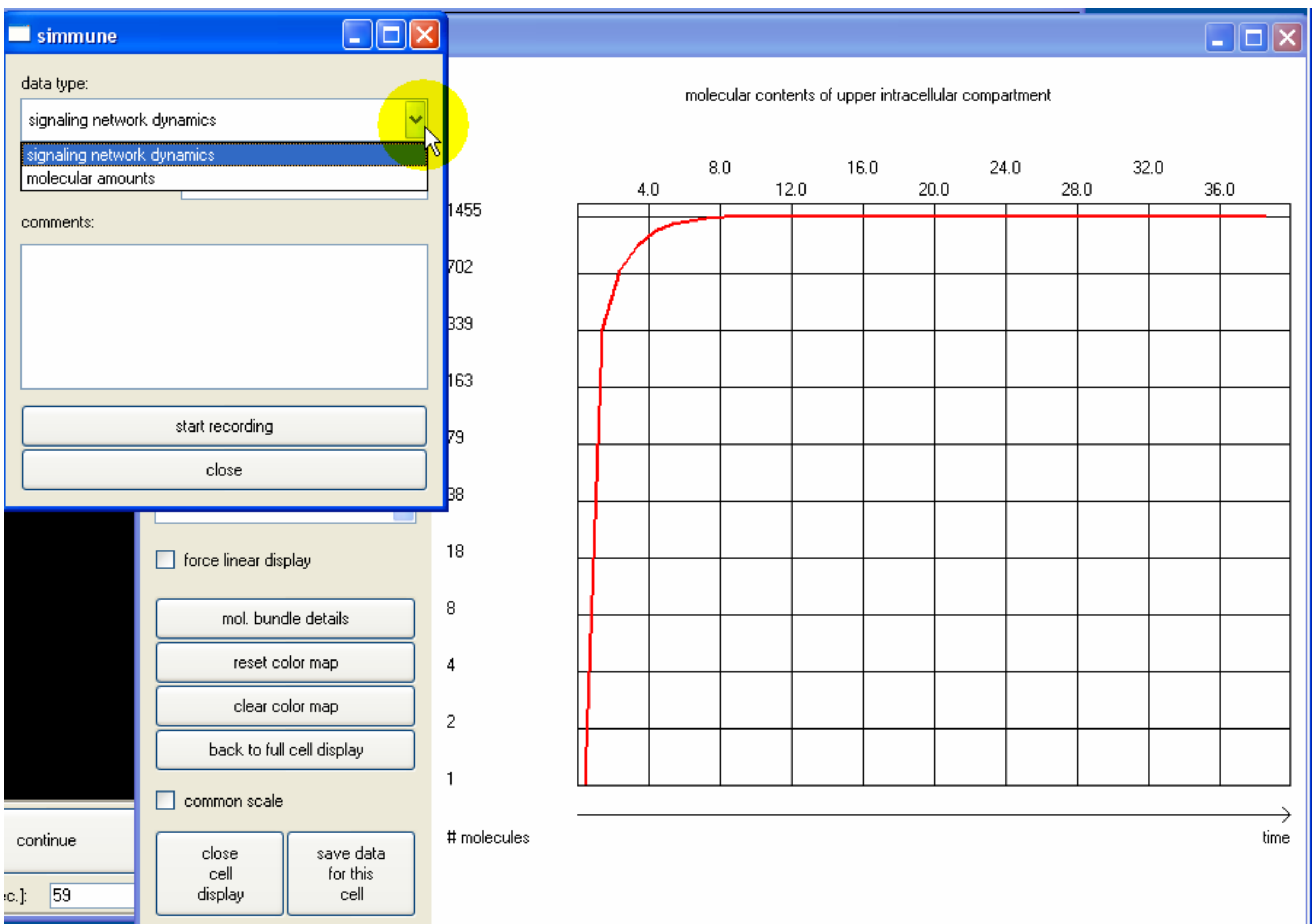

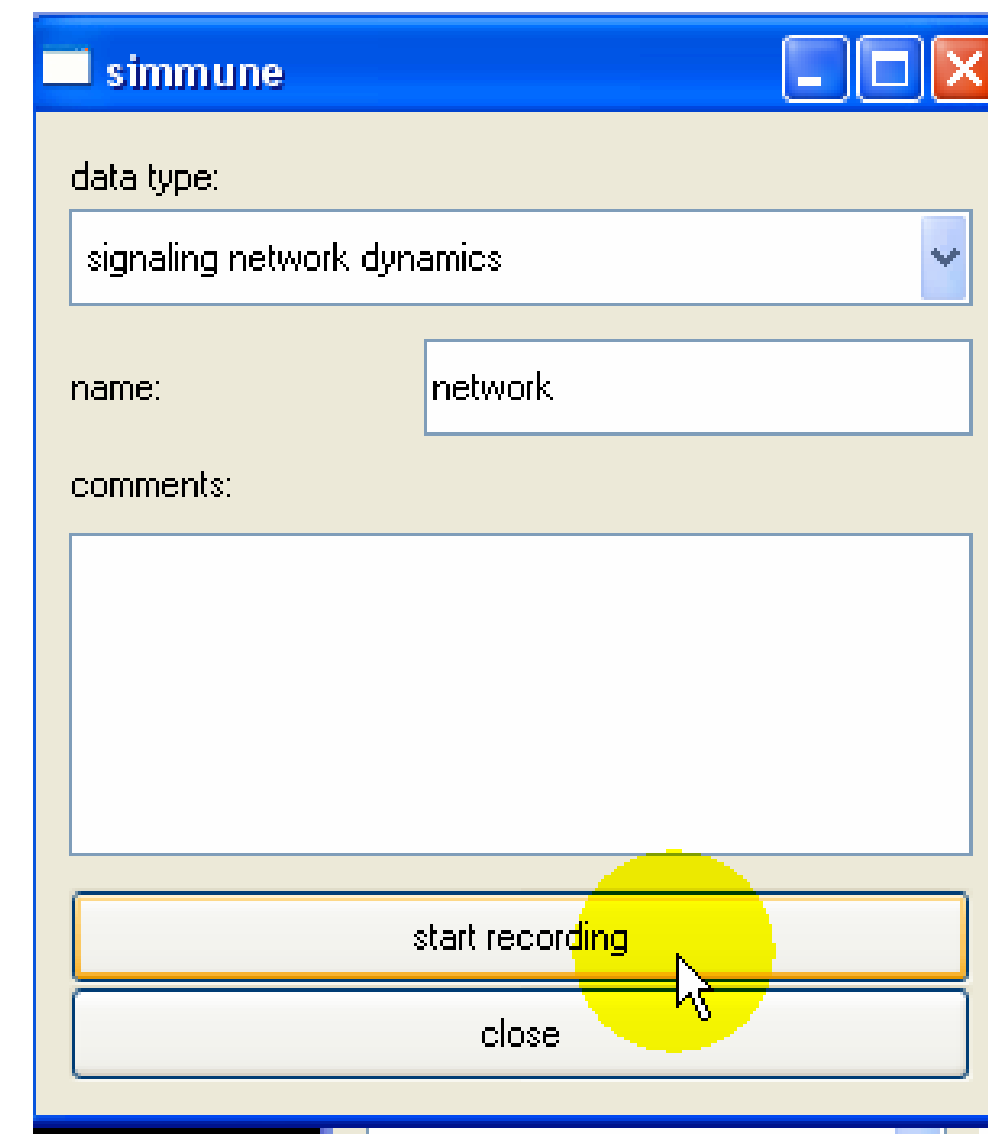

Click ‘start recording’ to start saving the network flow data.

(32) Click ‘set molecule concentration fields’ to apply extracellular molecule concentration fields.

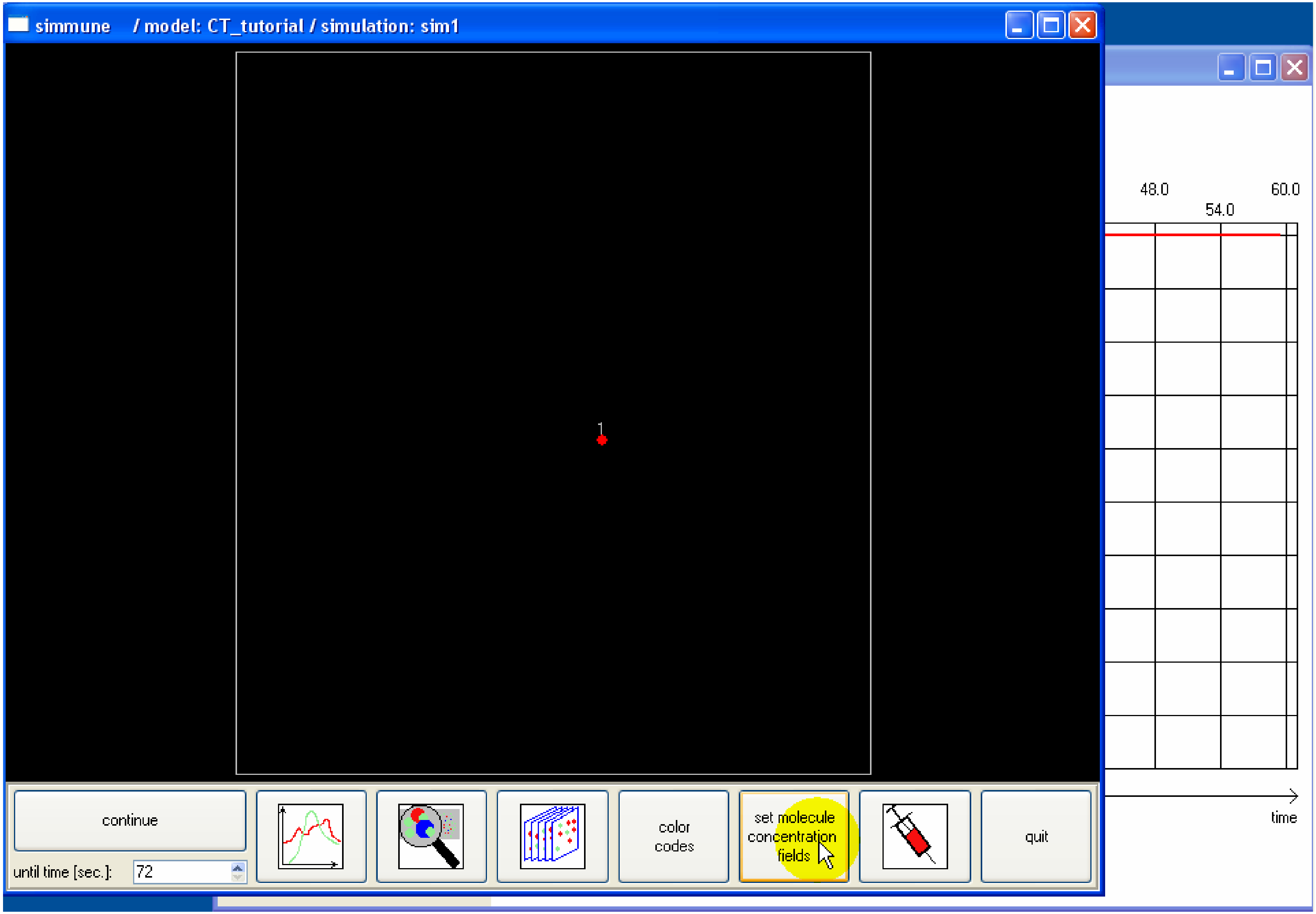

(33) For a point source gradient, set source- and control point concentration.

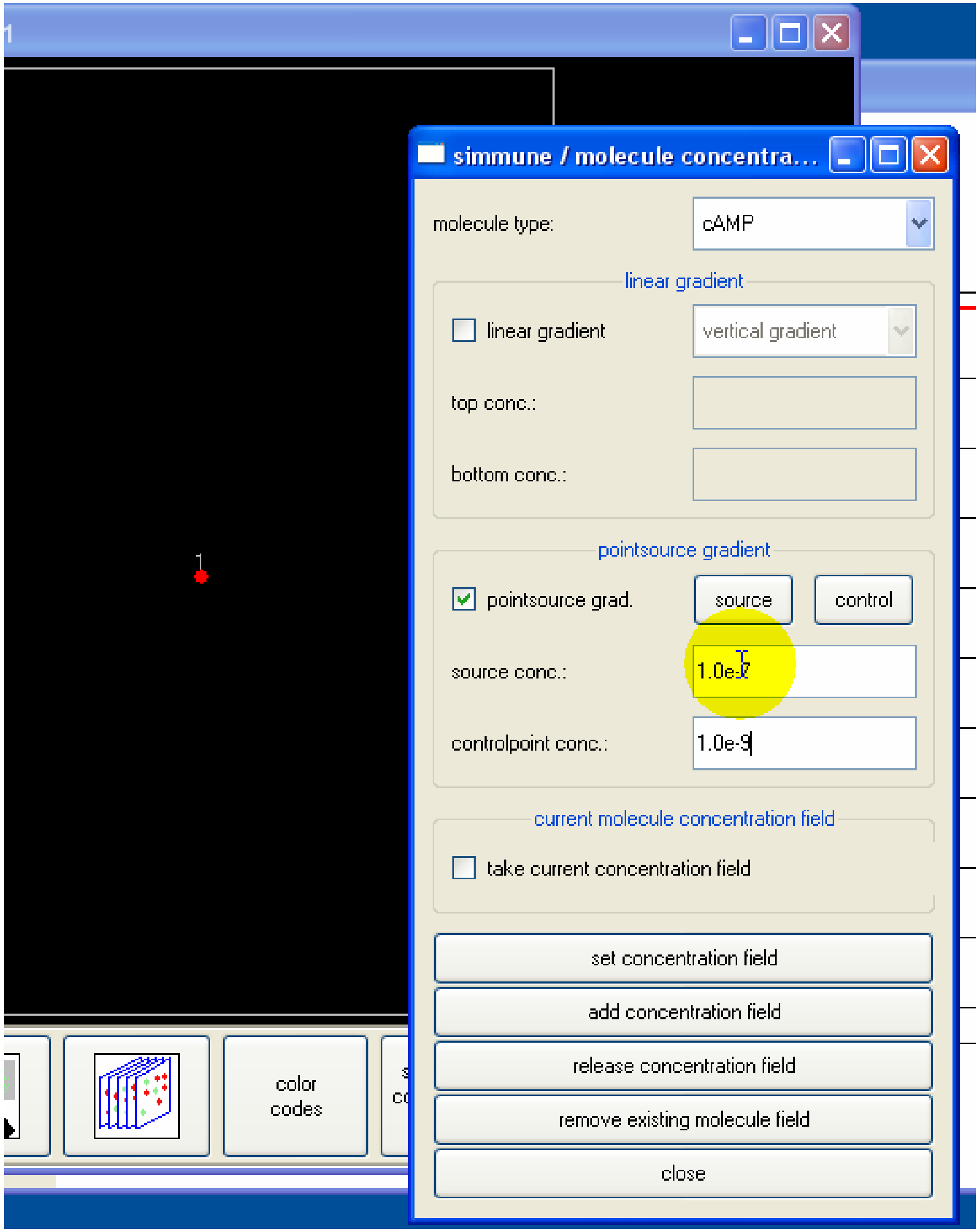

(34) Click on ‘source’ and then on the position within the compartment that should be the origin of the gradient. Analogously for the control point.

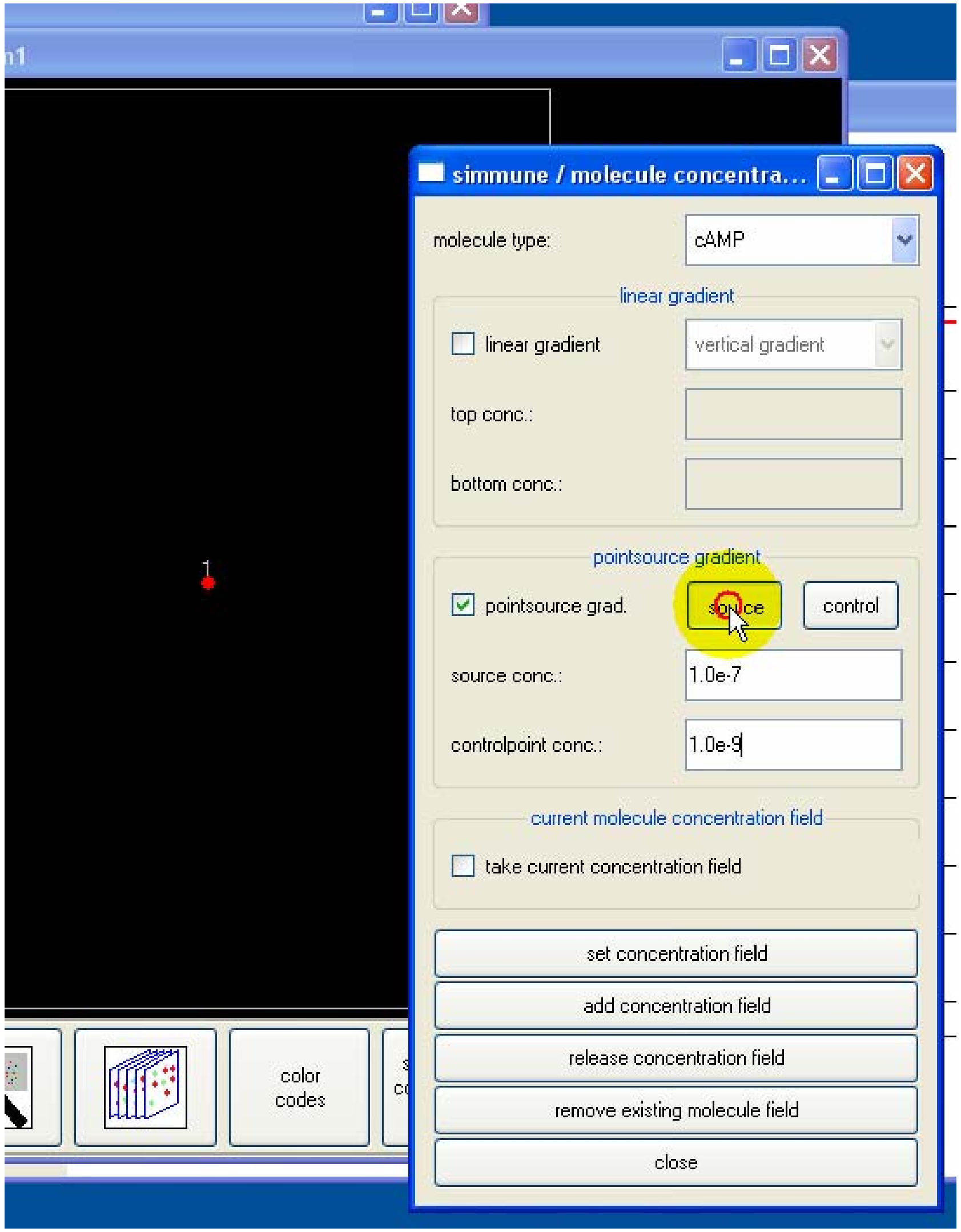

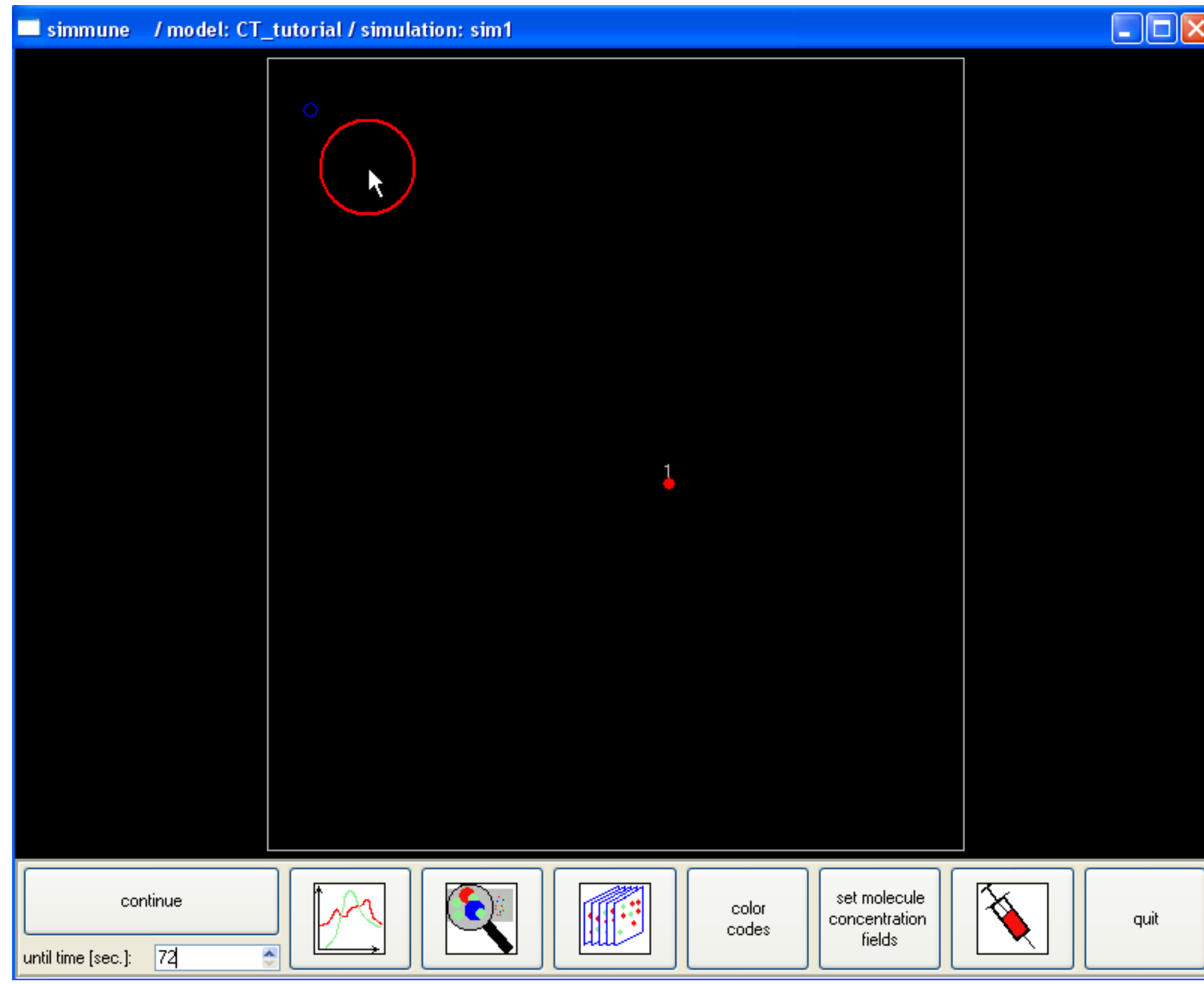

(35) The source point will be marked by a blue circle, the control point by a yellow circle. Click ‘set concentraton field’ to establish the gradient.

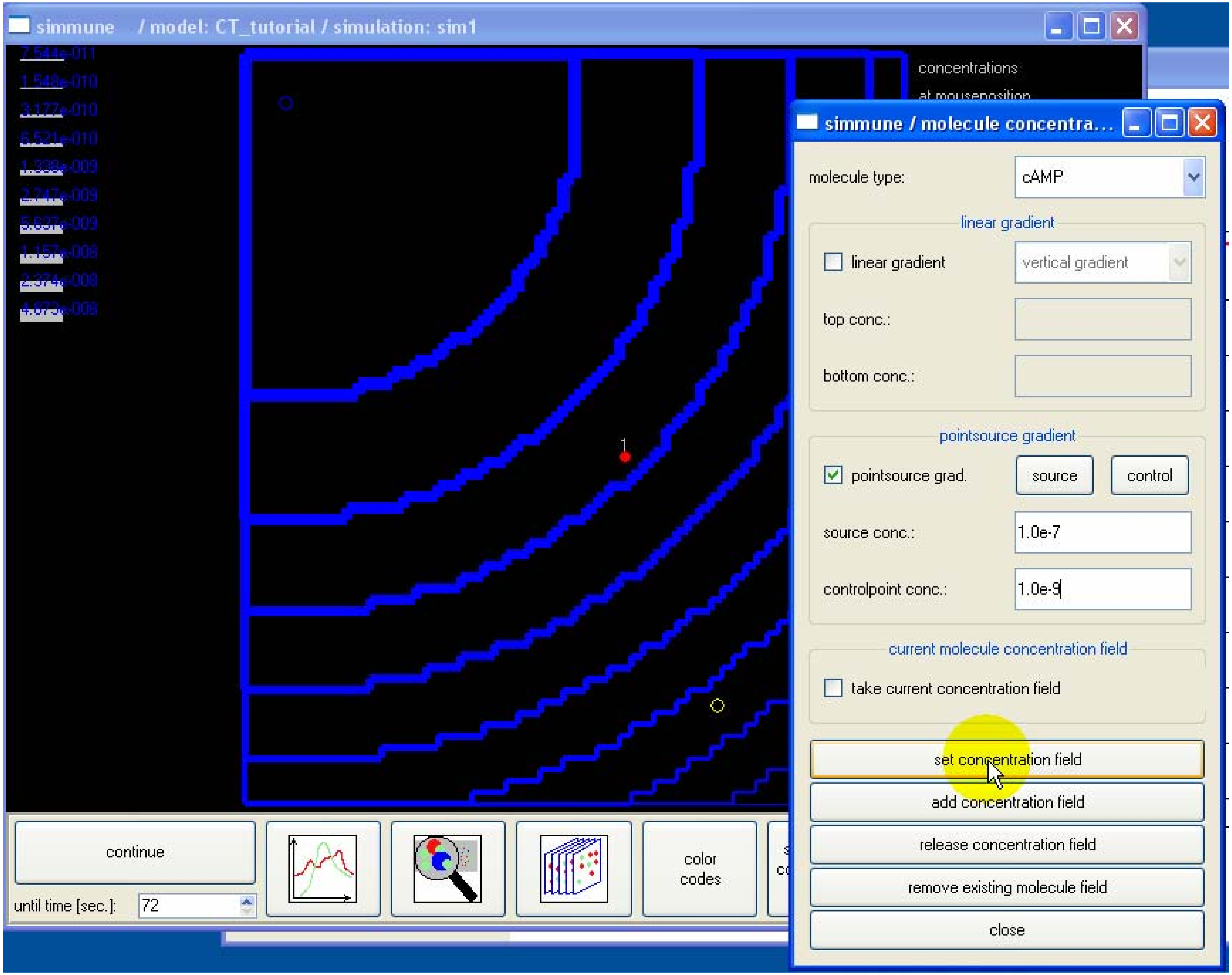

(36) The response of the cell will show some fluctuations because the cell is (randomly) moving within the gradient.

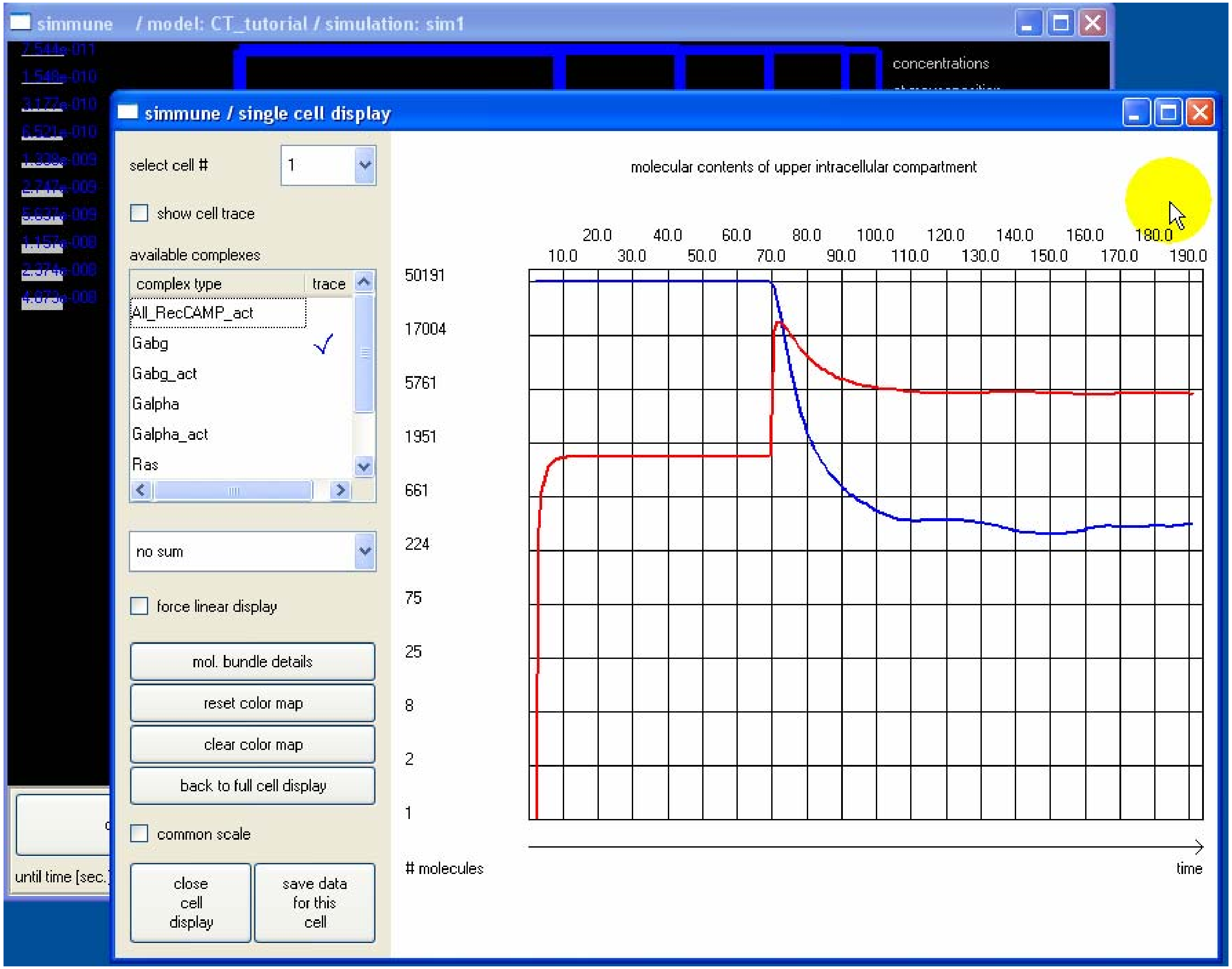

(37) The components of the ‘All_RecCAMP_act’ bundle will all be located in the membrane domain.

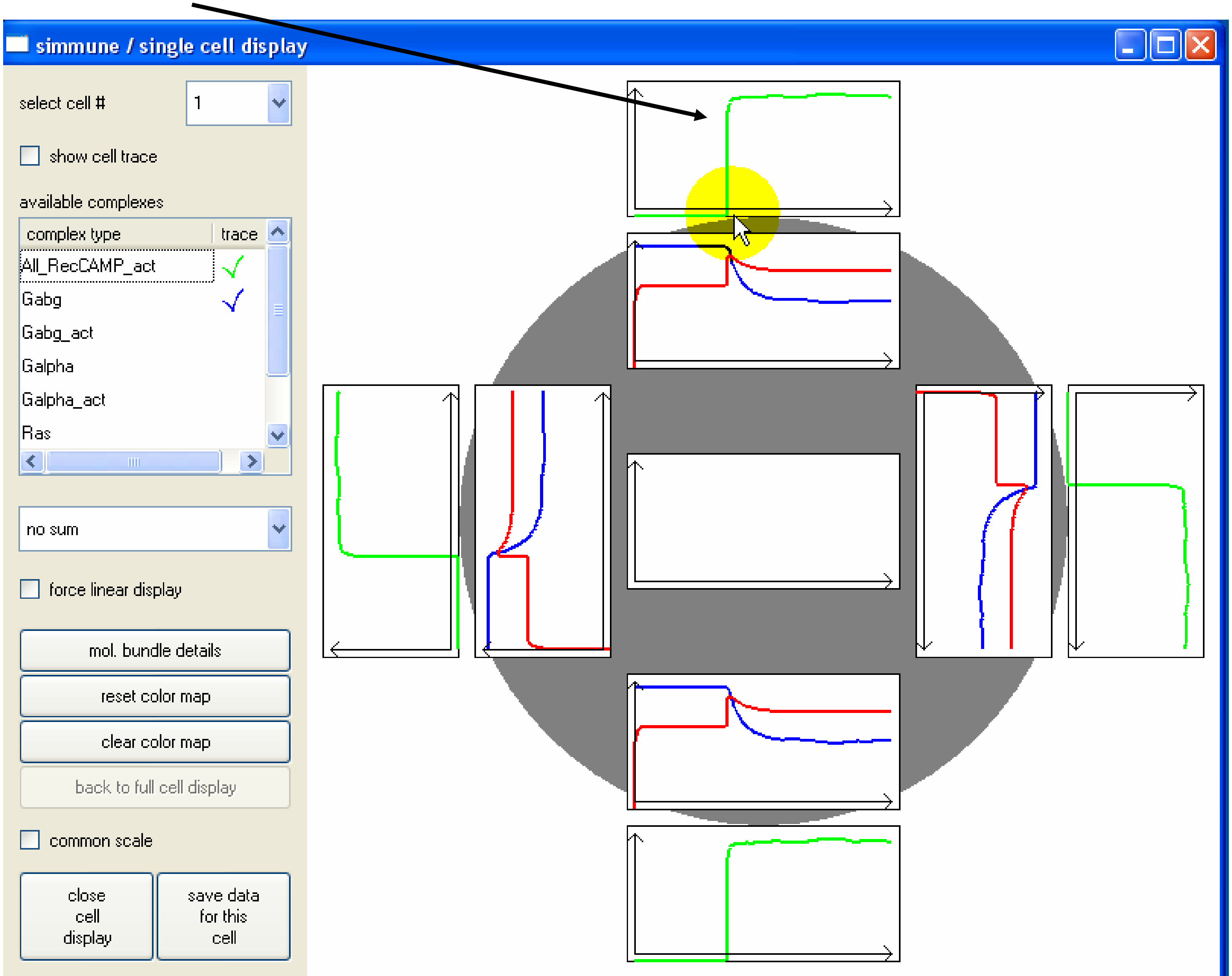

(38) Stop the network recording by clicking on the ‘save data for this cell’ button again and then clicking ‘stop recording’ in the data recording window.

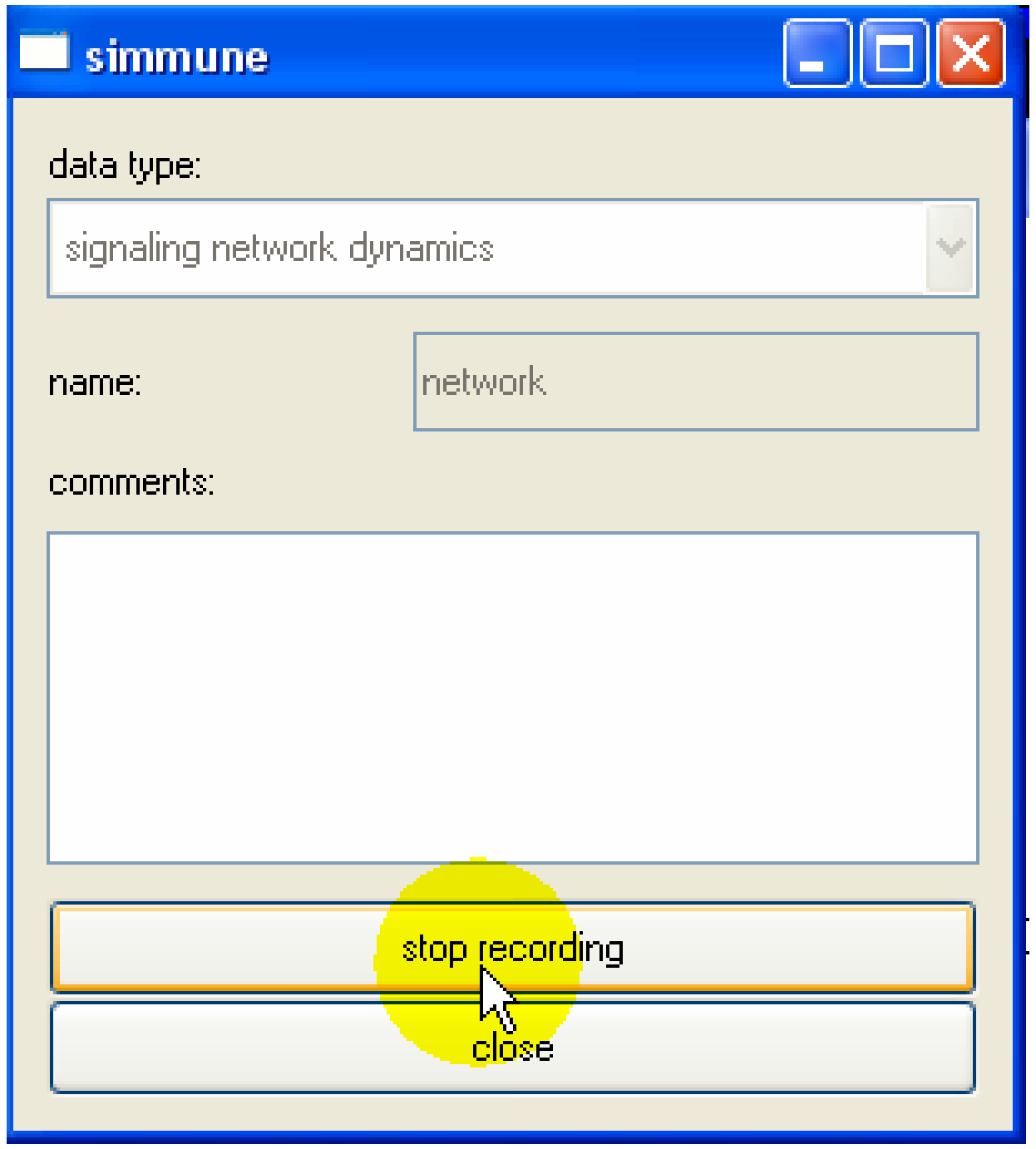

(39) Then, select ‘molecular amounts’ (instead of ‘signaling network dynamics’) define a name for the data and click ‘save amounts’. This will save the localized concentrations for all selected complexes.

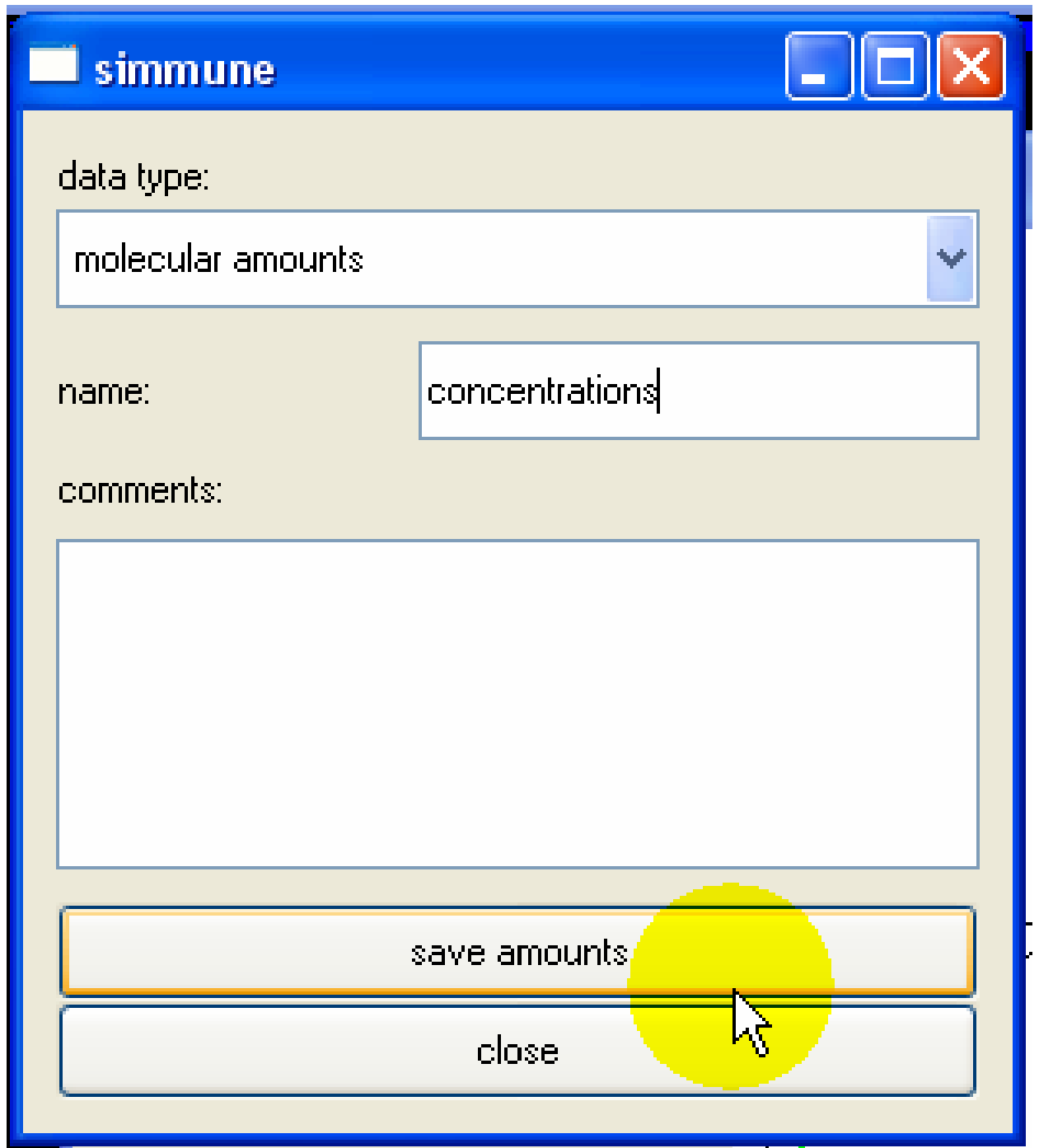

(40) Selecting the ‘show cell trace’ option will show the cell’s trace in the compartment over time.

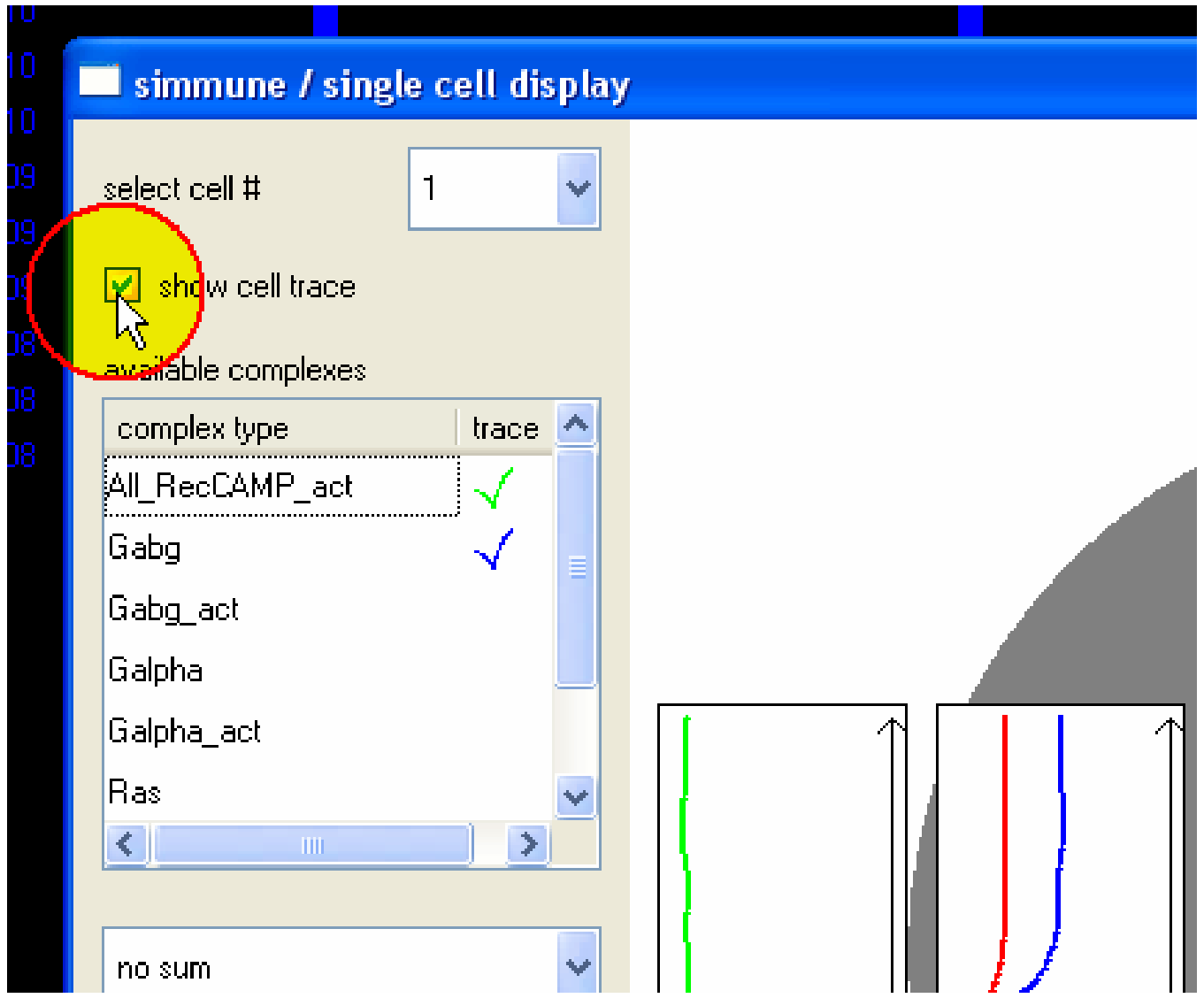

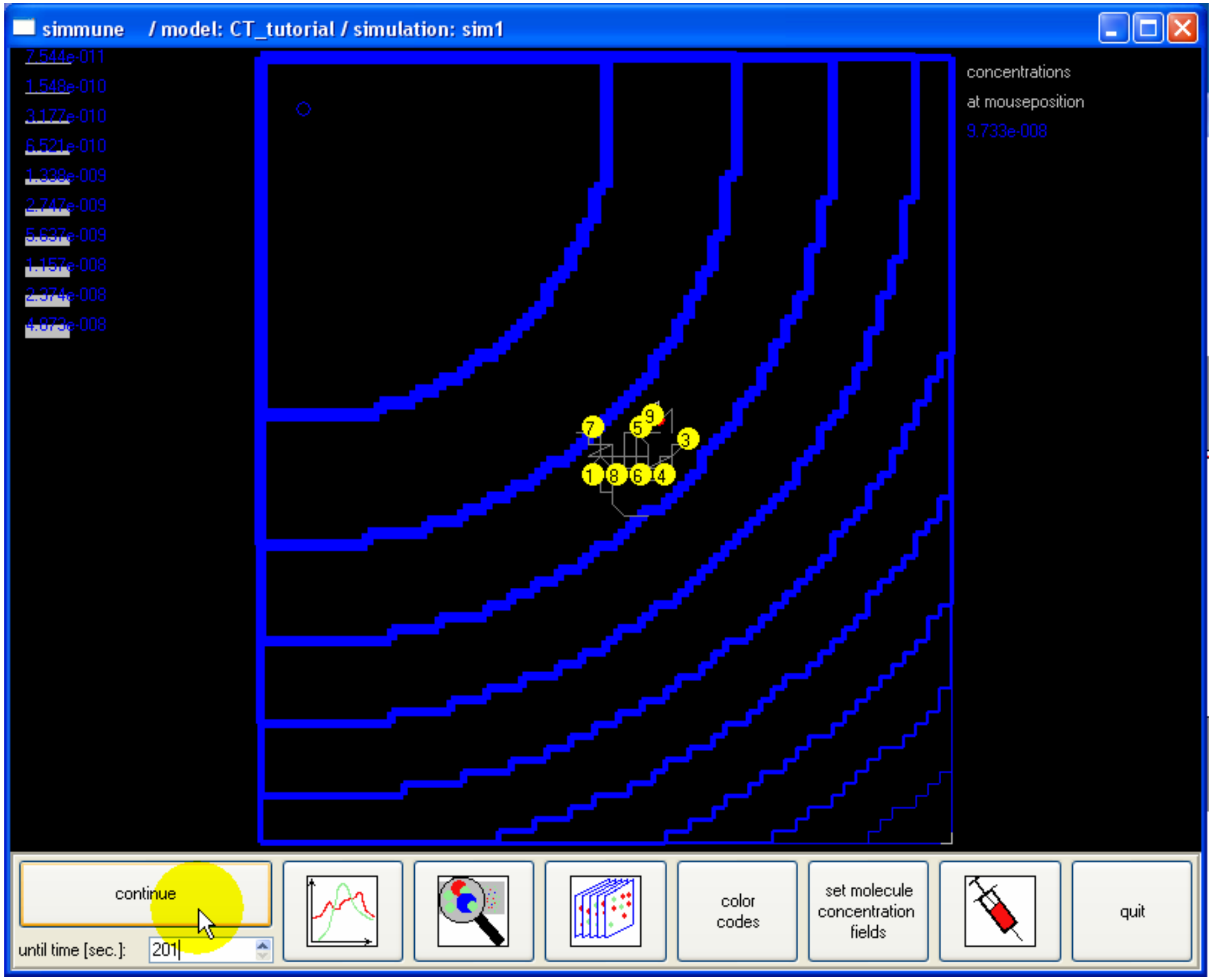

…and relate the positions of the cell to its intracellular biochemistry:

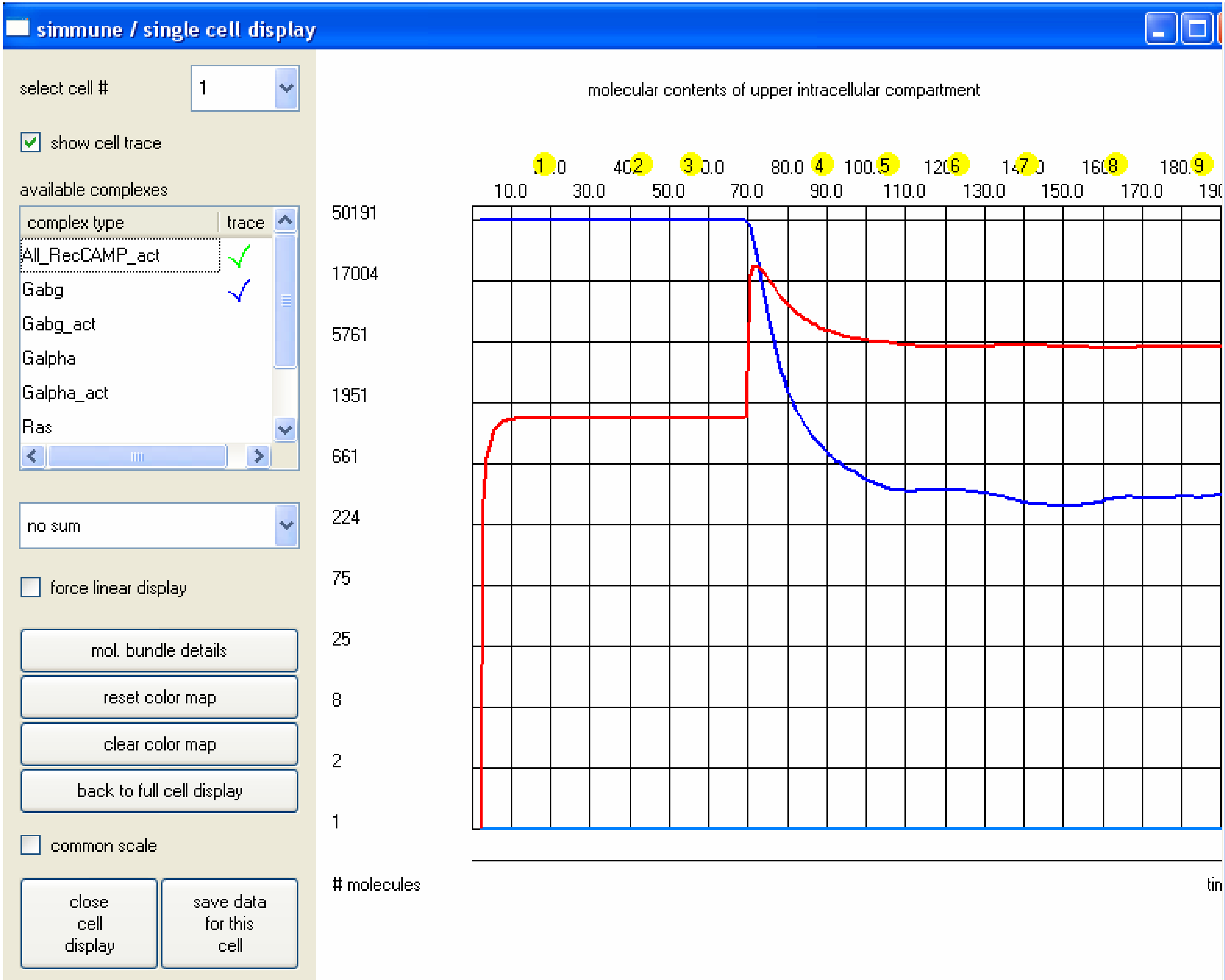

(41) After the simulation is finished we can investigate the simulation data we saved: molecule amount time series or reaction network dynamics.

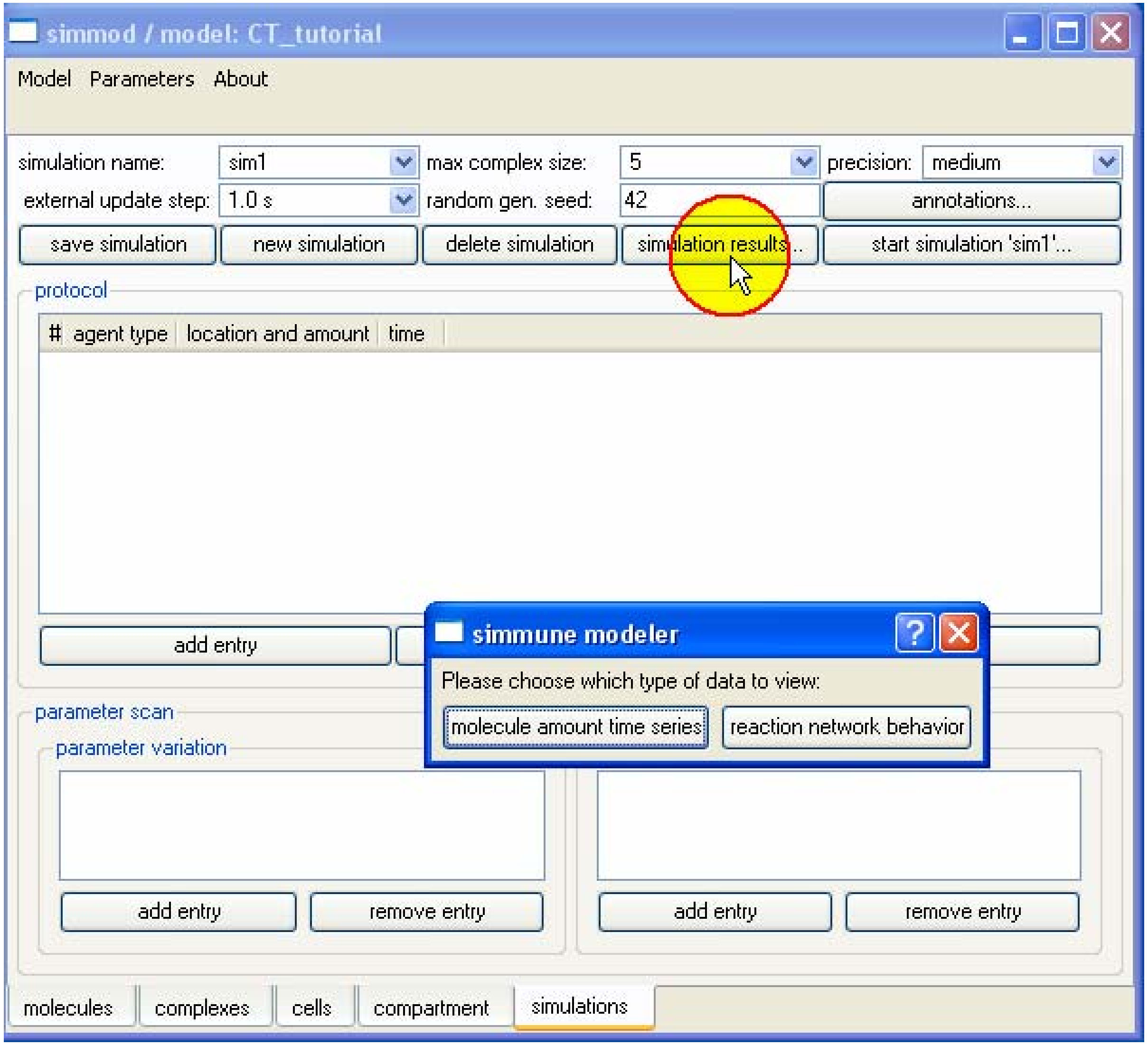

(42) Concentrations in different regions of the cell:

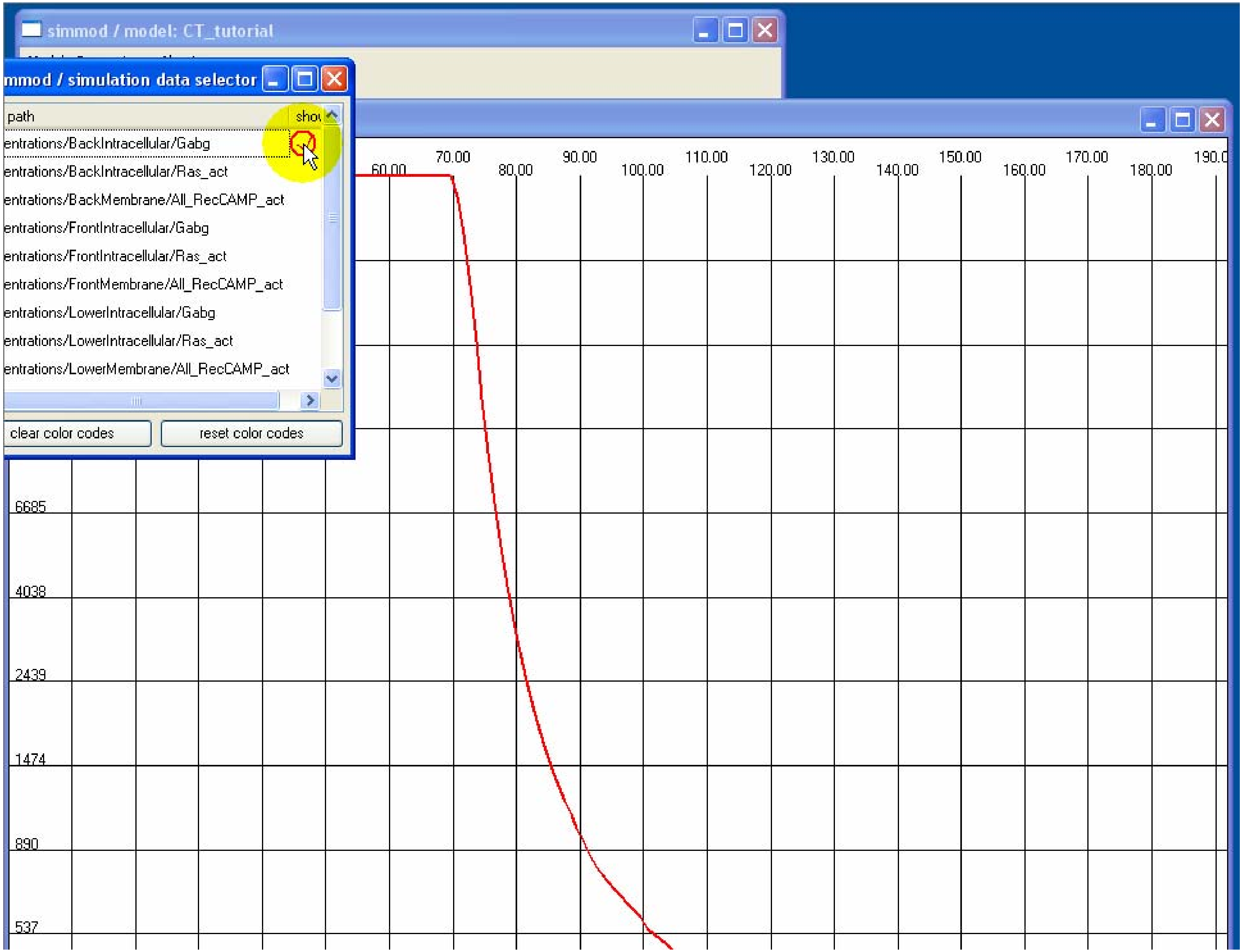

(43) The network browser visualizes the recorded behavior of the reaction network.

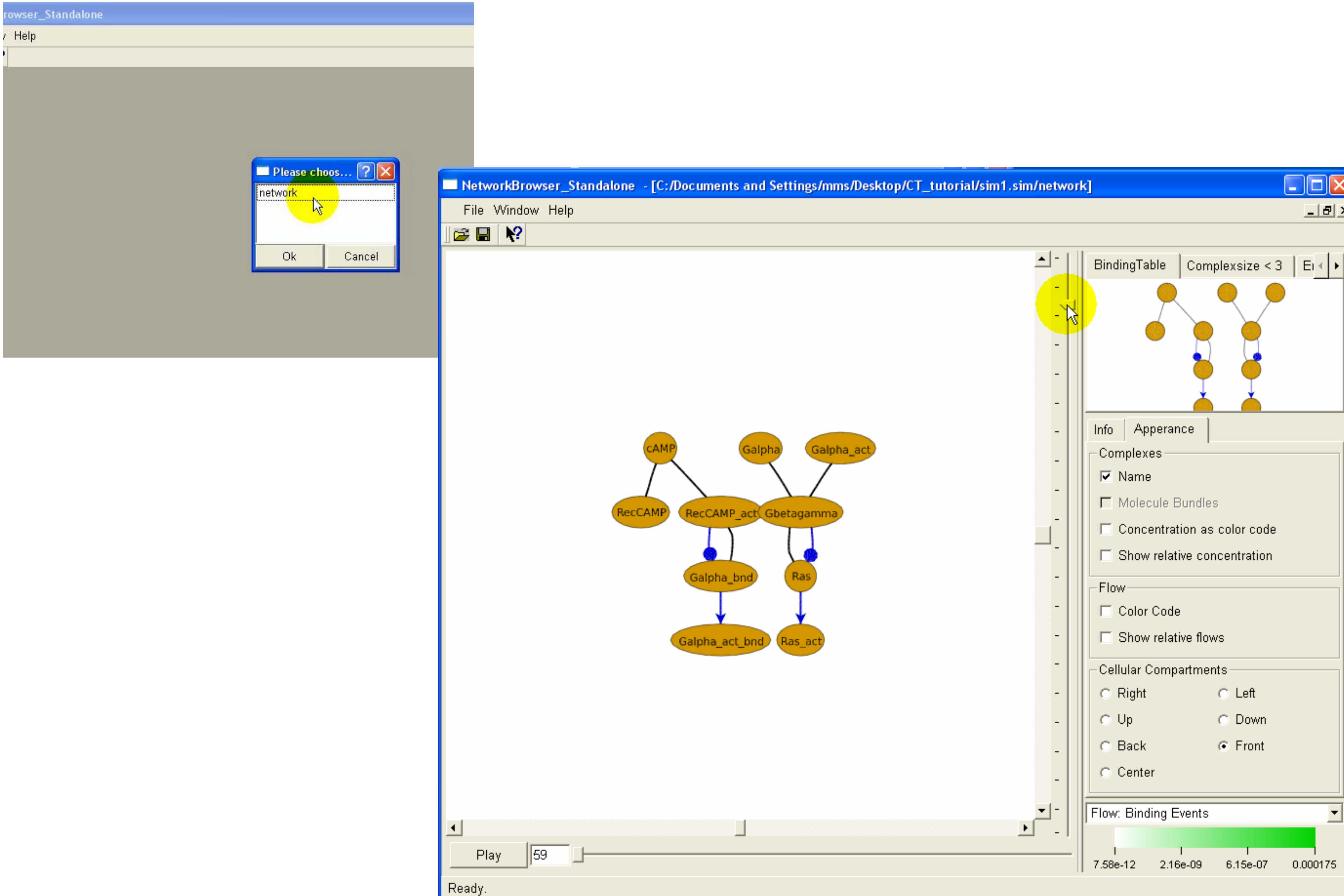

(44) The network browser encodes concentrations and reaction flows as color saturation (see browser documentation for details).

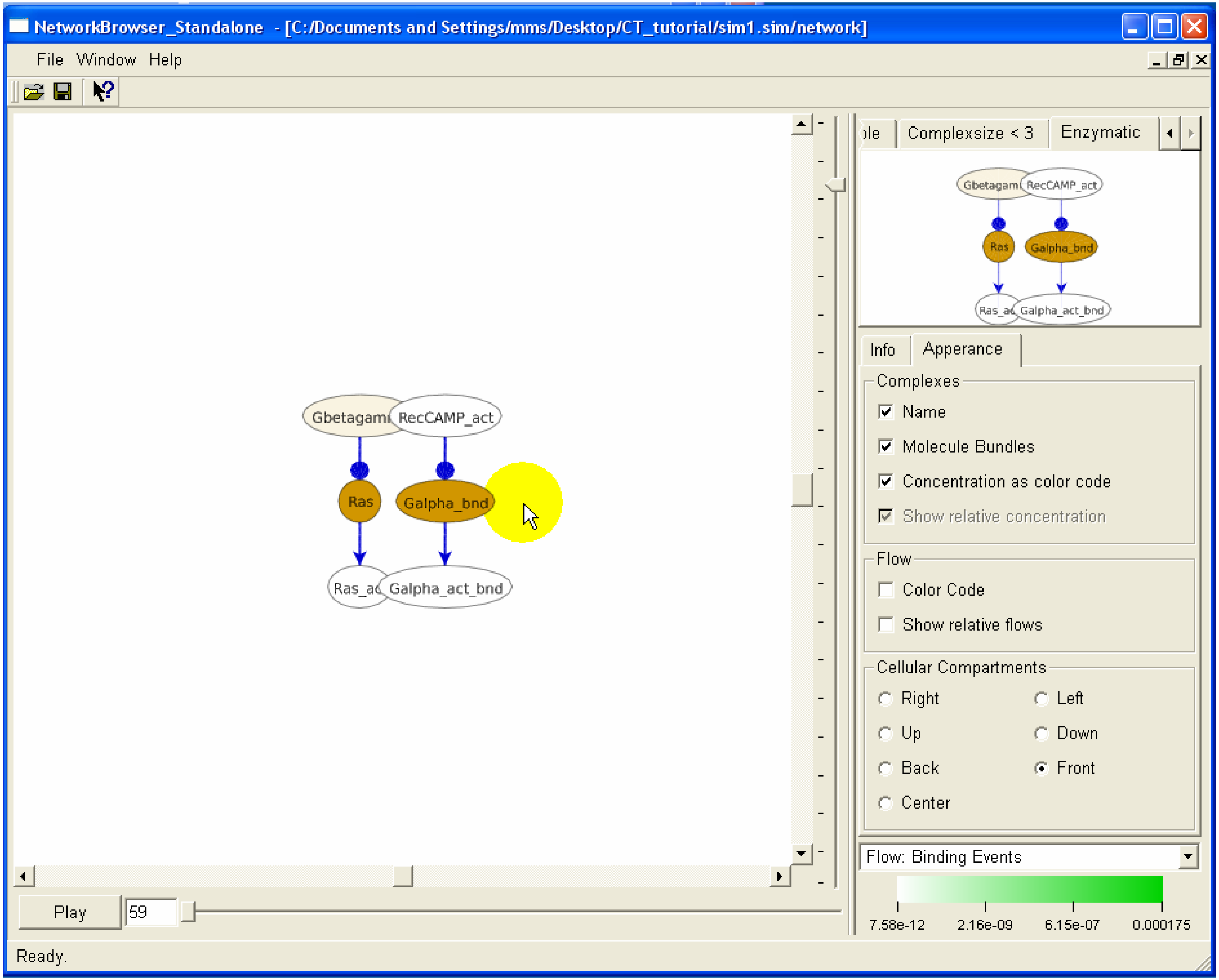

Supplement: Supplementary file 11 [file pcbi.0020082.sd011.pdf]
