# Supplementary material for: Key Role of Local Regulation in Chemosensing Revealed by a New Molecular Interaction-Based Modeling Method
Source: PLoS Comput Biol. 2006 Jul 21;2(7):e82. doi: 10.1371/journal.pcbi.0020082 (PMC1513273; doi:10.1371/journal.pcbi.0020082)

Simmune tutorial 5

Working with the full Dicty Chemtax model
Simulating the cellular response to a cAMP gradient

(1) Open the DictyChemtax model.

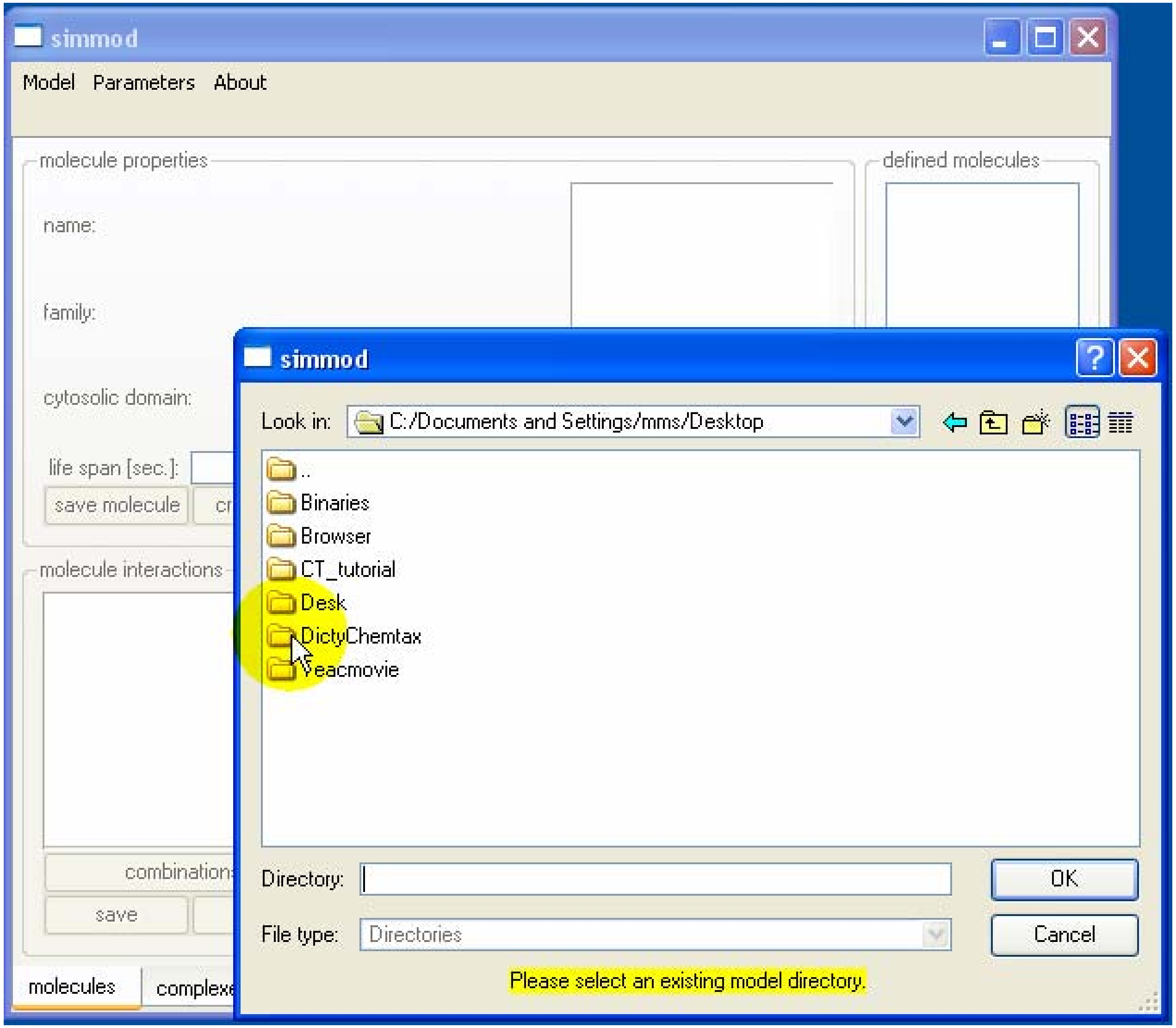

(2) The ‘defined molecules’ lists all molecular ingredients of the model. Clicking on ‘binding table’ opens a window displaying the binding interactions between the molecules.

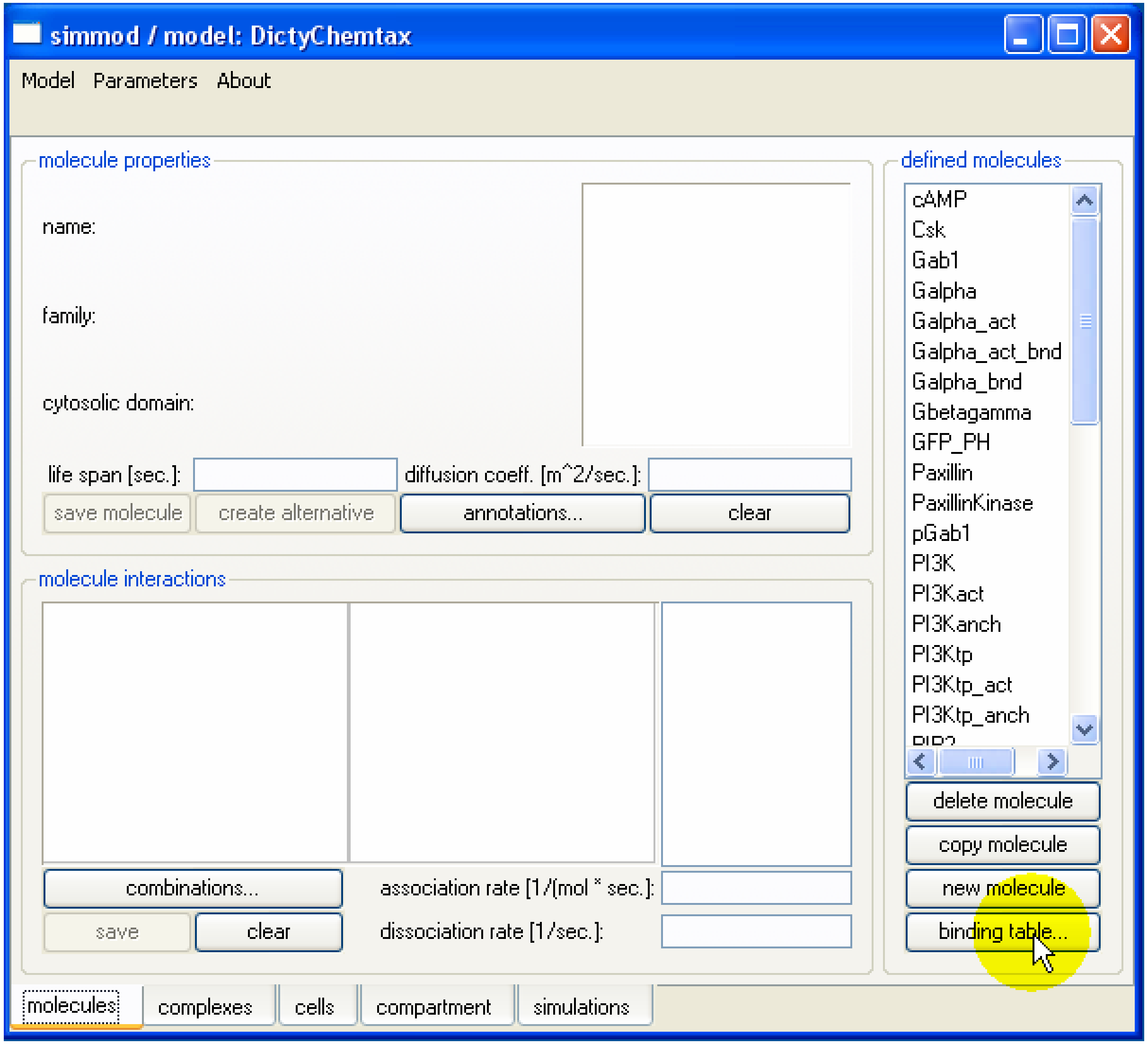

The binding table lists interactions between molecular binding sites

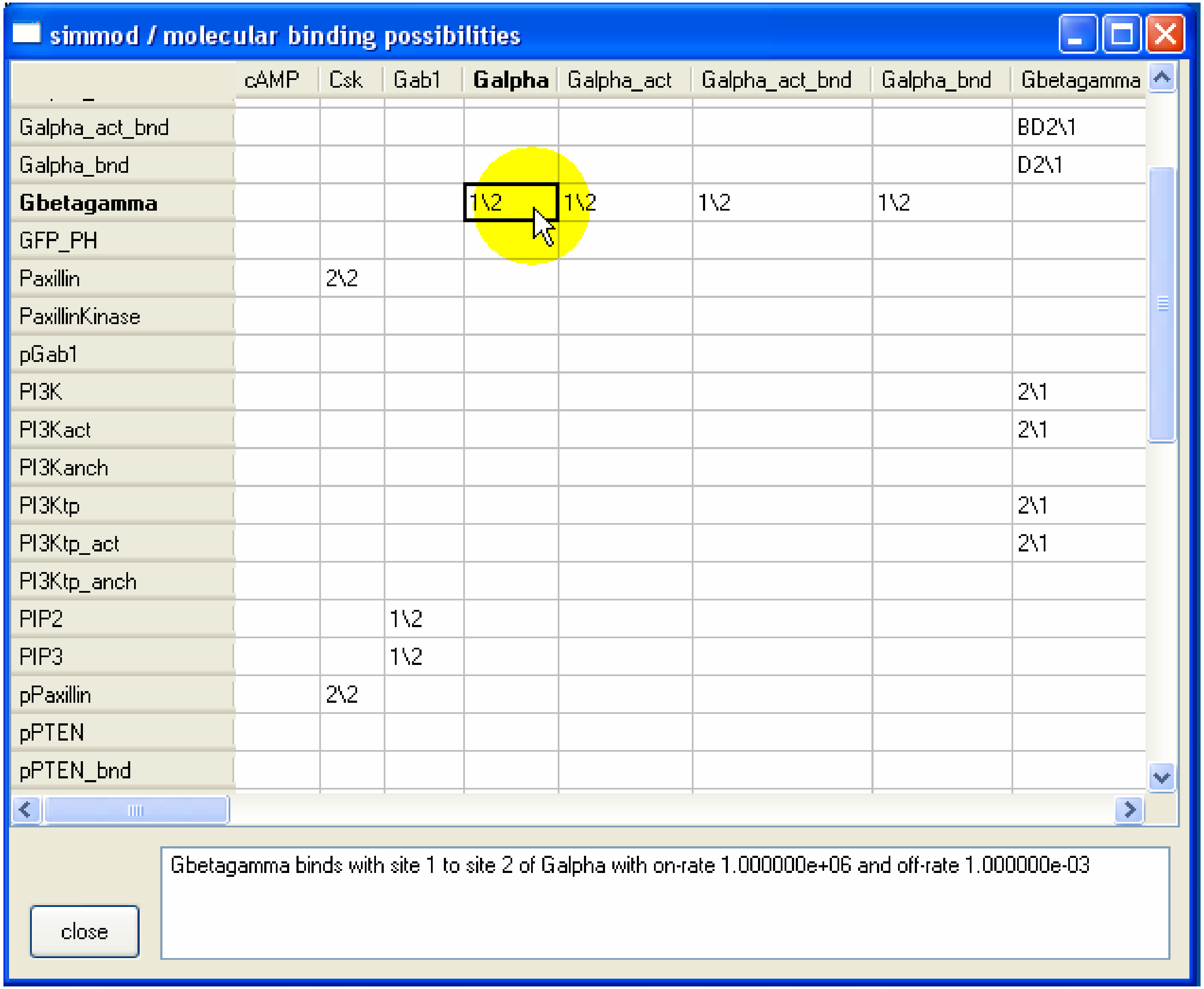

	cAMP	Csk	Gab1	Galpha	Galpha_act	Galpha_act_bnd	Galpha_bnd	Gbetagamma
Galpha_act_bnd								BD2\1
Galpha_bnd								D2\1
**Gbetagamma**				1\2	1\2	1\2	1\2	
GFP_PH								
Paxillin		2\2						
PaxillinKinase								
pGab1								
PI3K								2\1
PI3Kact								2\1
PI3Kanch								
PI3Ktp								2\1
PI3Ktp_act								2\1
PI3Ktp_anch								
PIP2			1\2					
PIP3			1\2					
pPaxillin		2\2						
pPTEN								
pPTEN_bnd								

(3) Go to the 'complexes' tab. Clicking on the 'complex table' button opens a window displaying the user-defined complexes (note that the software creates all possible complexes in the system – see documentation for details).

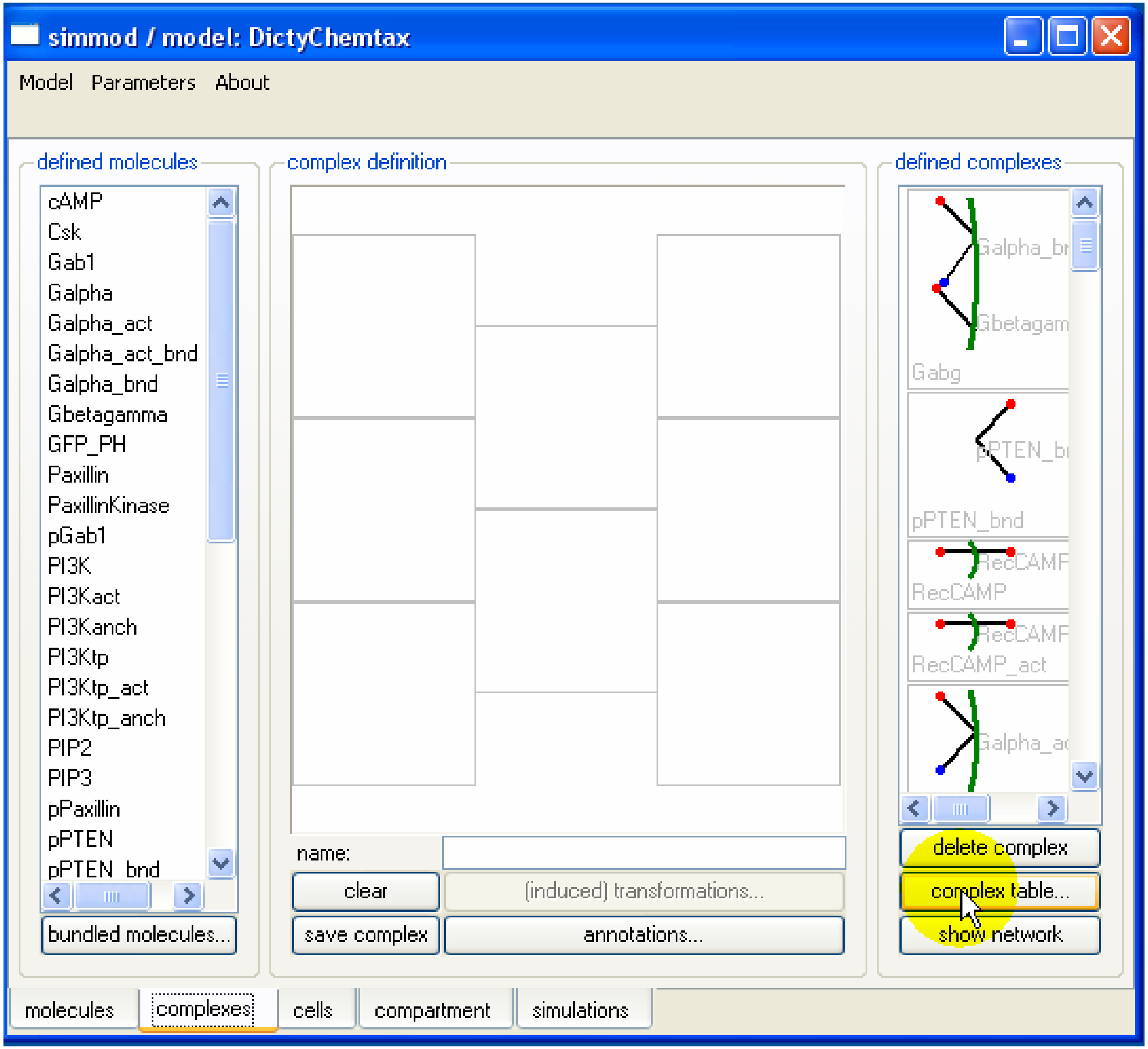

The complex table lists all complexes and their spontaneous and enzymatically induced transformations.

simmod / molecule complexes

	molecular components	(enzymatic) decays
RasGAP	RasGAP	enzyme: Gbetagamma product: (I)RasGAPopen
PI3K	PI3K	enzyme: Ras product: (I)PI3Kact enzyme: Ras_act product: (I)PI
PI3Kact	PI3Kact	enzyme: PI3Ktp product: (I)PI3K enzyme: PI3Ktp_act product: (I)I
PIP2	PIP2	enzyme: PI3Kact product: (I)PIP3 enzyme: PI3K product: (I)PIP3
PIP3	PIP3	enzyme: pPTEN_bnd product: (I)PIP2 enzyme: PTENbnd produc
Ras	Ras	enzyme: Gbetagamma product: (I)Ras_act
Ras_act	Ras_act	(I) decay rate: 1.000e+01 product: Ras enzyme: RasGAP produc
Ras_act_PI3K_Gbg	PI3K, Ras_act, Gbetagamma	
Ras_act_PI3Kact_Gbg	PI3Kact, Ras_act, Gbetagamma	
RecCAMP_deact	RecCAMP(RecCAMPcyto_deact)	(I) decay rate: 1.000e-01 product: RecCAMP
pSHIPanch	pSHIPanch	enzyme: PTENbnd product: (I)SHIPanch
PTEN	PTEN	enzyme: Src_act product: (I)pPTEN enzyme: Src product: (I)pPTI
pPTEN	pPTEN	enzyme: PTENph_act product: (I)PTEN enzyme: PTENph produ
PTENbnd	PTENbnd	enzyme: Src product: (I)pPTEN_bnd enzyme: Src_act product: (I
PTENph	PTENph	enzyme: Gbetagamma product: (I)PTENph_act
SrcAct_act	SrcAct_act	
Gabg_act	Galpha_act_bnd, Gbetagamma	
RecKinase	RecKinase	
PTENph_act	PTENph_act	(I) decay rate: 0.01 product: PTENph
Paxillin	Paxillin	enzyme: PaxillinKinase product: (I)pPaxillin
pPaxillin	pPaxillin	enzyme: SHP2 product: (I)Paxillin
Csk	Csk	
Gab1	Gab1	enzyme: Src_act product: (I)pGab1
SHP2	SHP2	
Src	Src	enzyme: SrcAct_act product: (I)Src_act enzyme: SrcActivator prc

close

The ‘model’ menu provides the option to create a textual representation of the ODEs representing the reaction network. The file is located in the model directory and is called ‘Reactions.txt’.

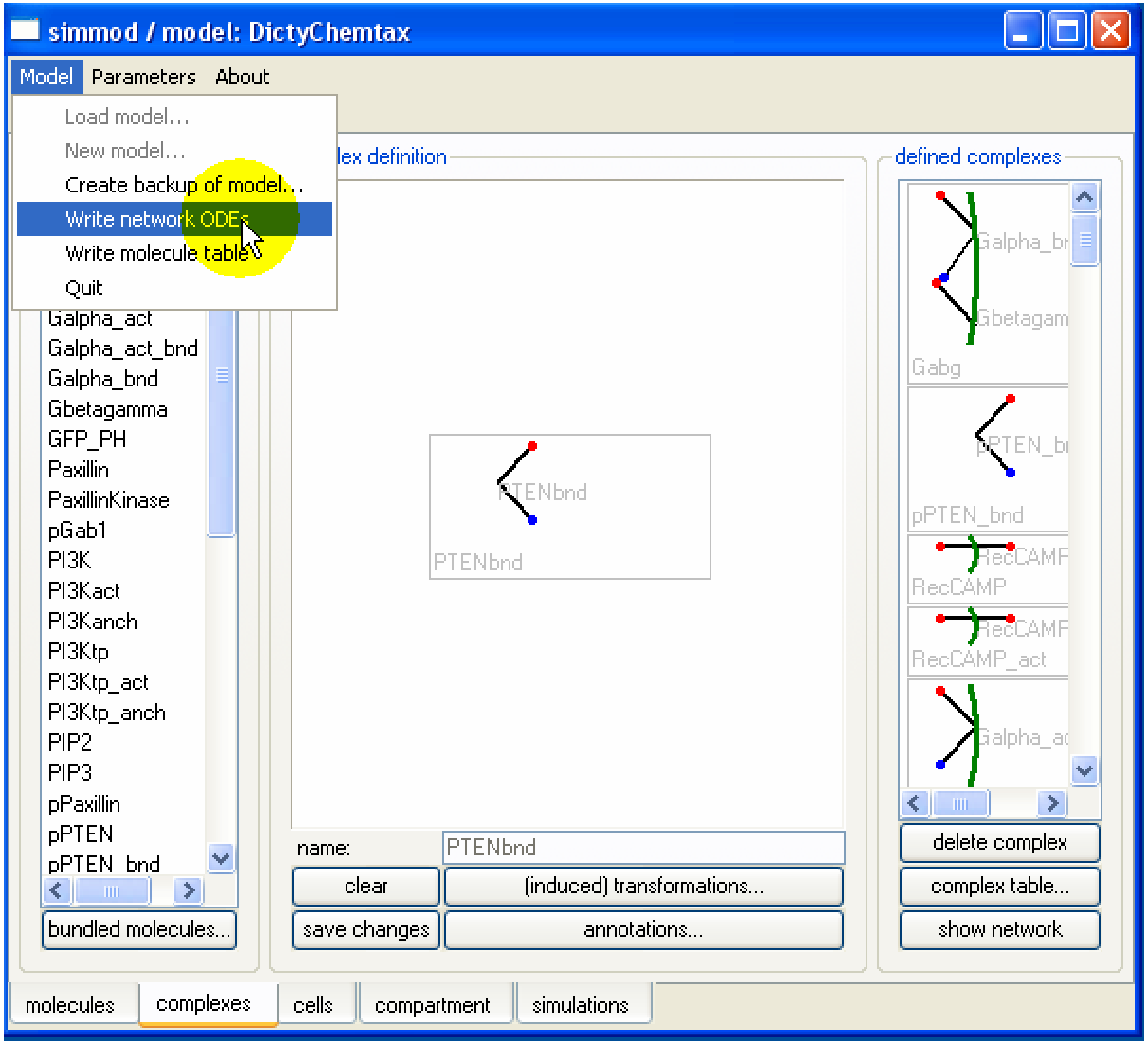

The compartment we use for the simulation of the response of Dicty to cAMP gradients is very small: 3x3x3 (in units of 10 microns). Just large enough for the cell with one layer of extracellular space around it to apply the cAMP gradients.

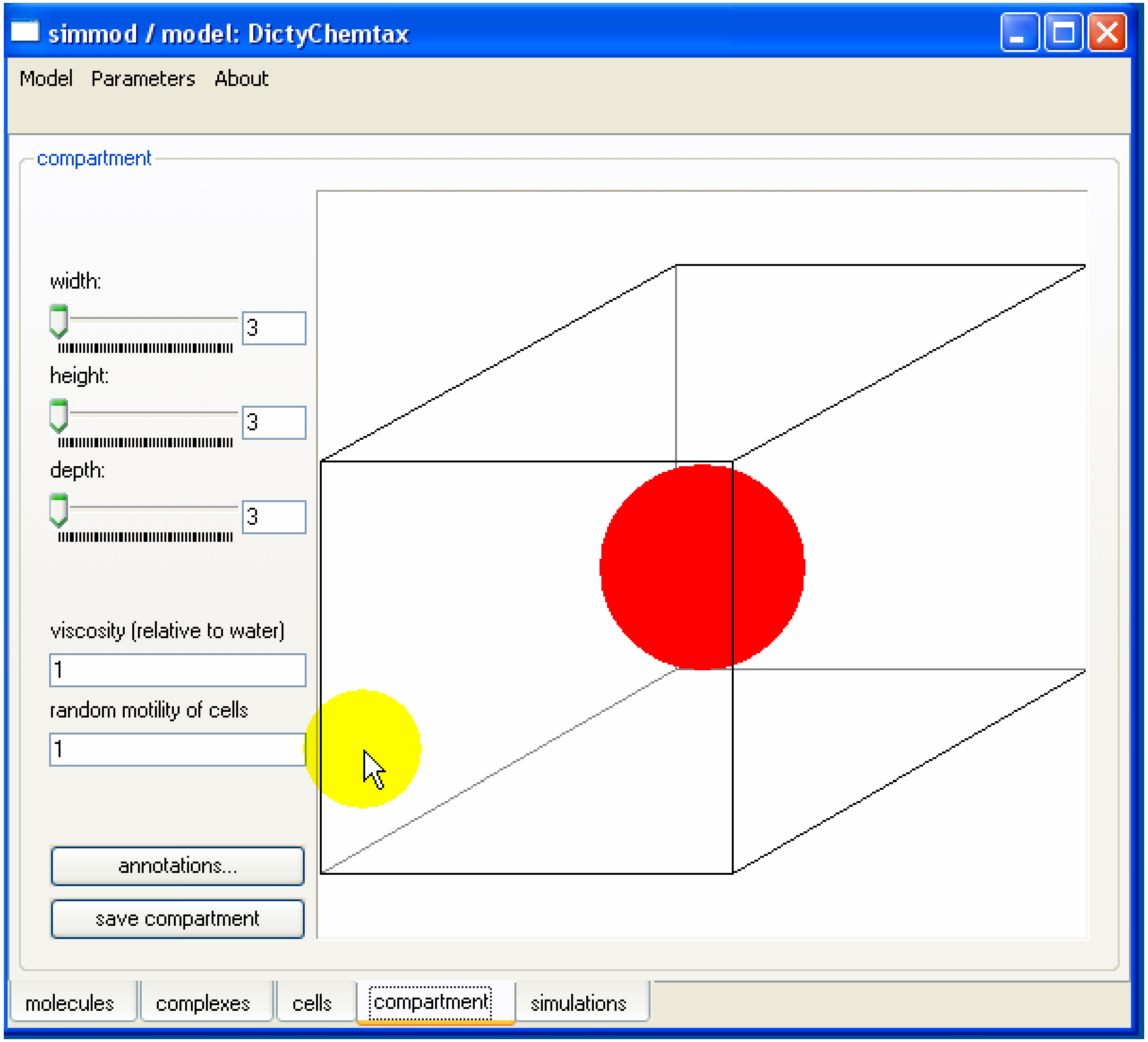

The simulation (‘sim1’) we use puts a ‘Dicty1’ type cell at the center of the compartment. Click ‘start simulation ‘sim1’’ to start a simulation.

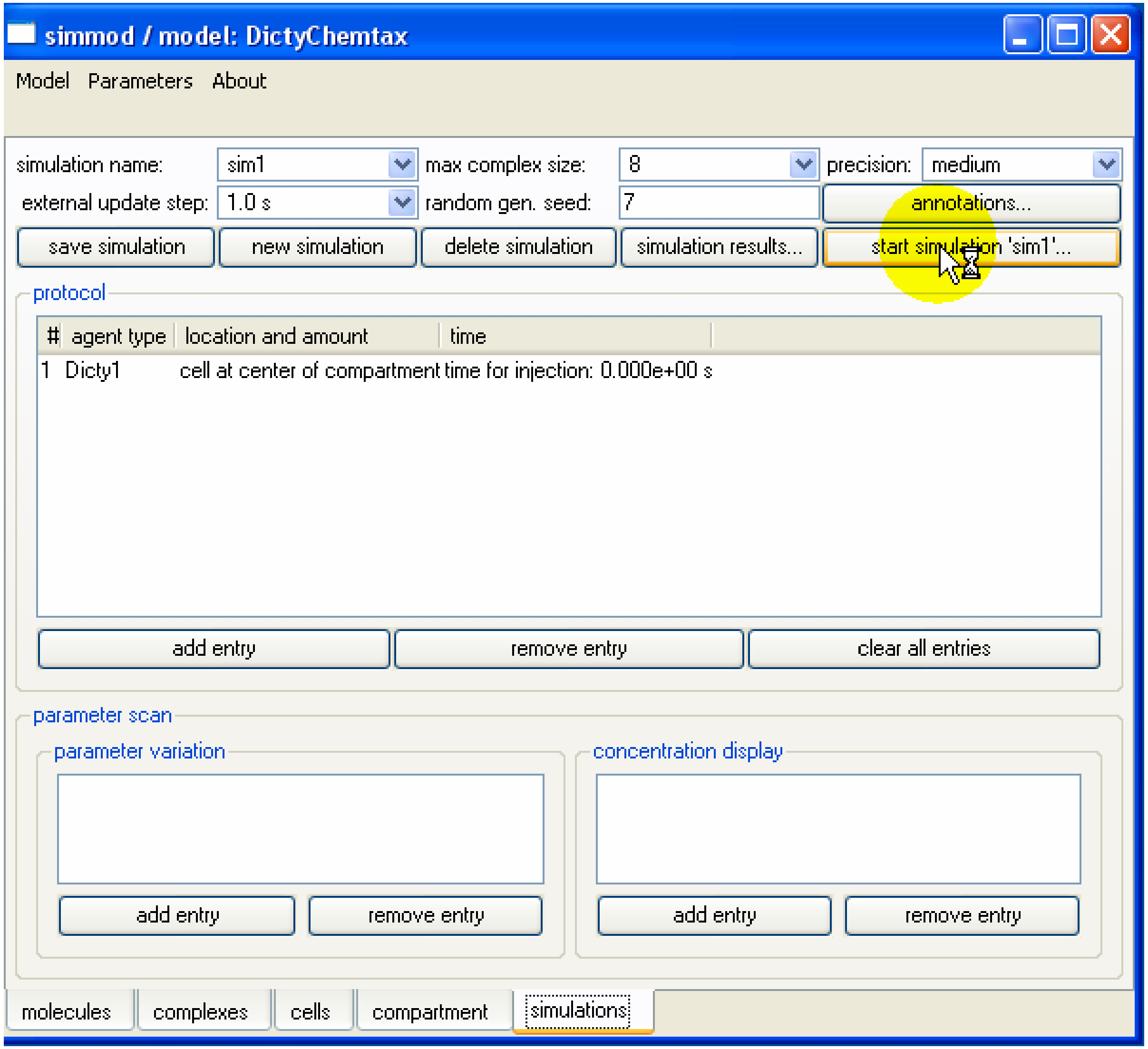

Simulate the system (containing the cell in the compartment's center) for 200 s to allow the intracellular biochemistry to approach an equilibrium.

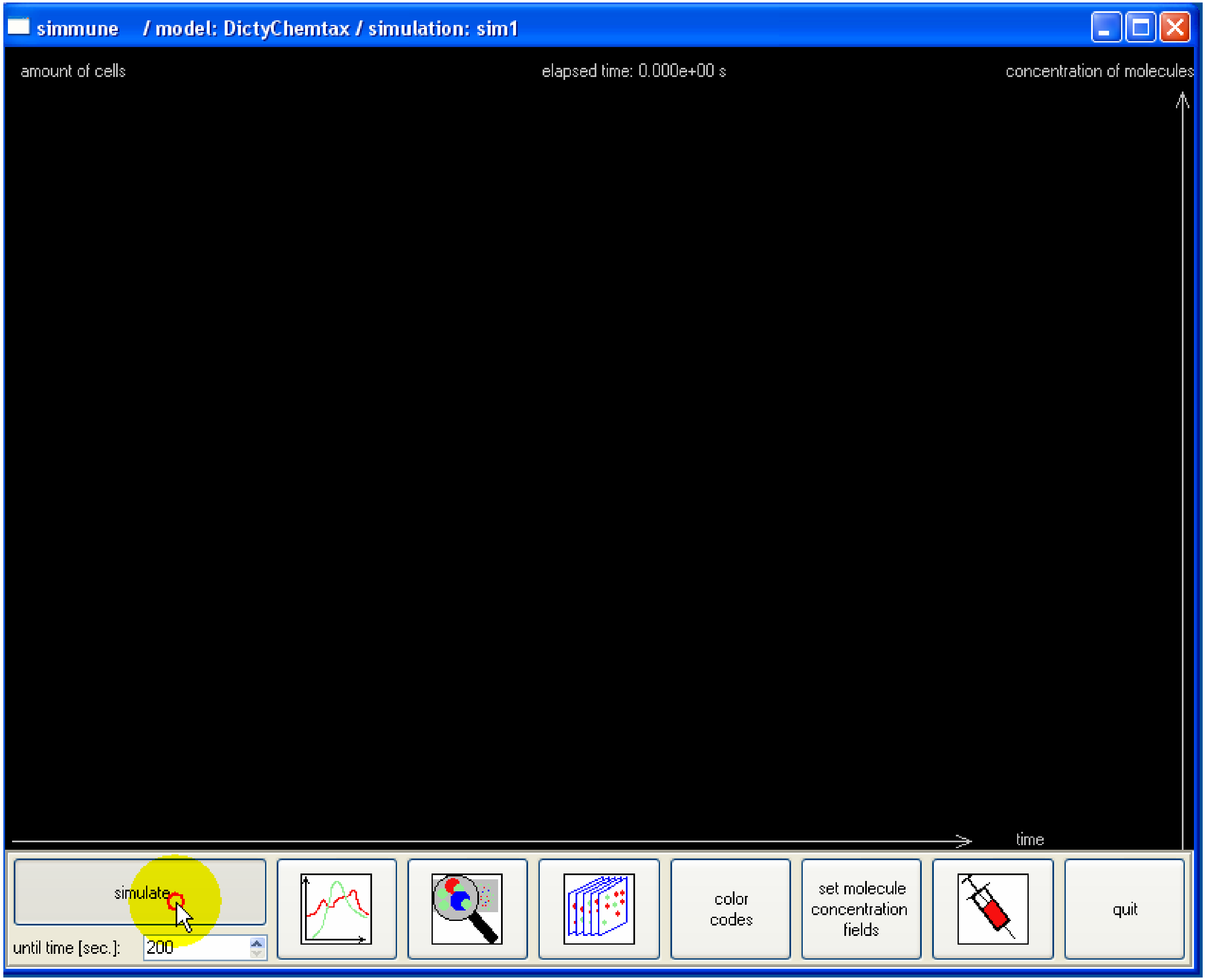

Go to the ‘slice’ view and then click on ‘color codes’.

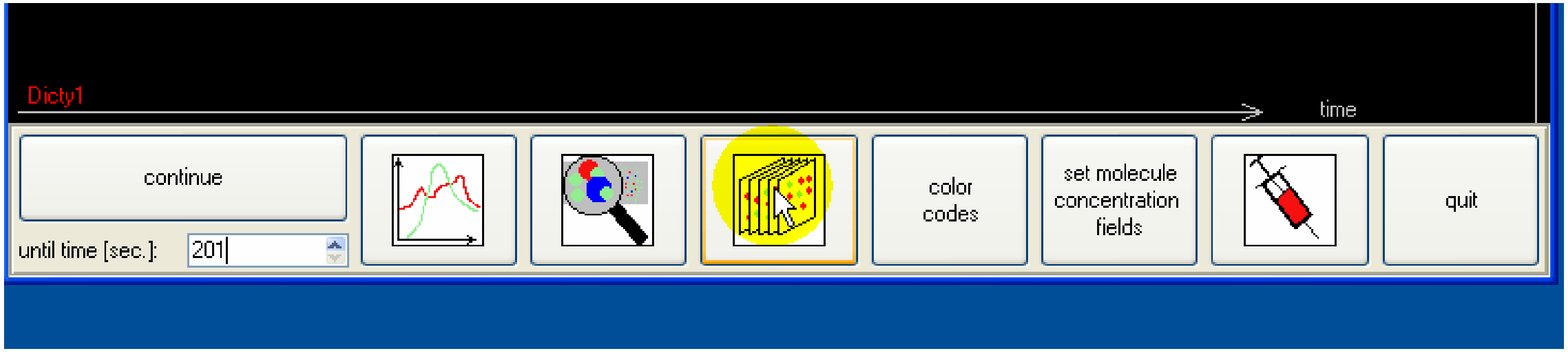

Double-click the ‘Dicty1’ entry to assign a color code to the cell.

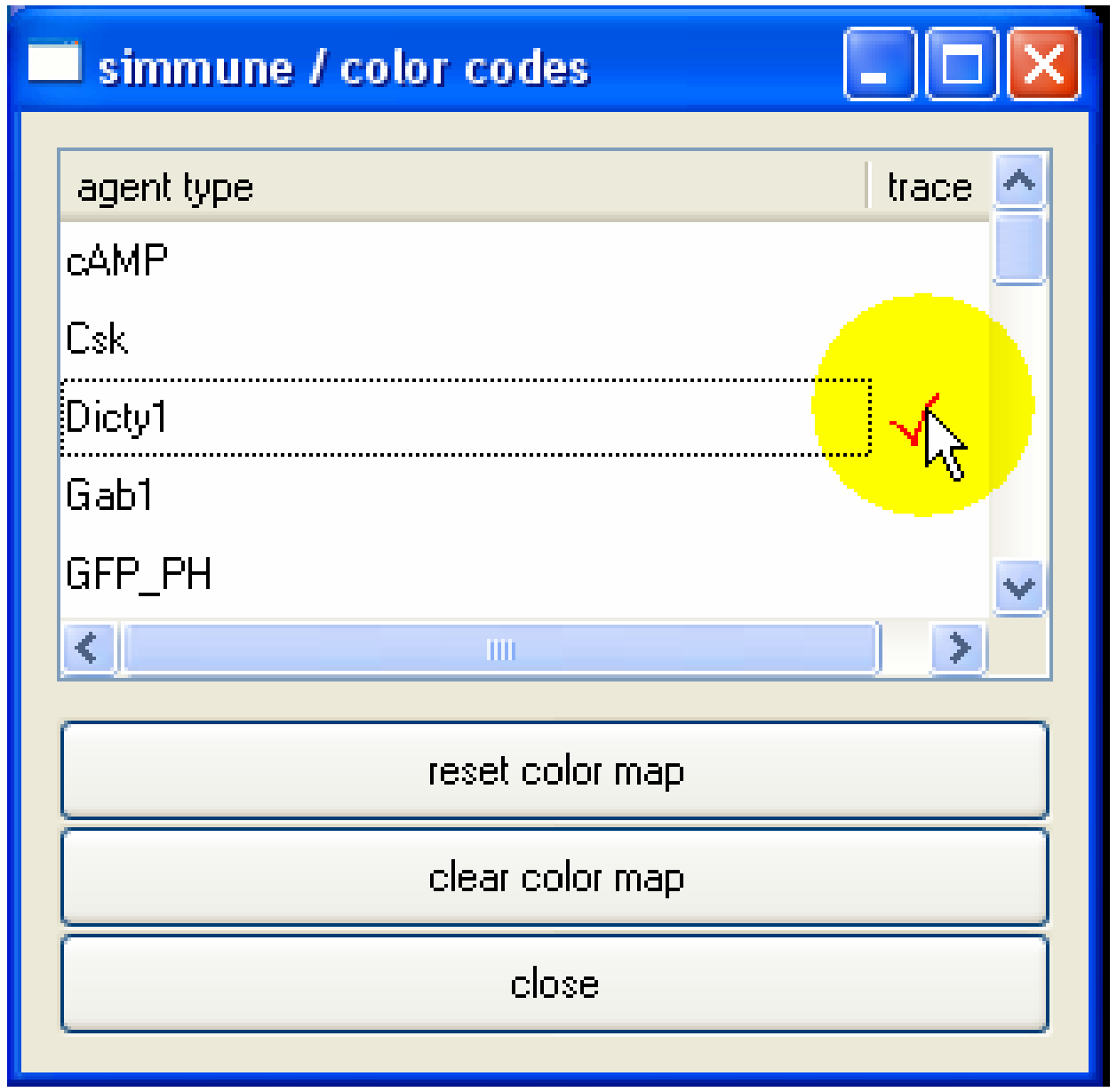

Click on the dot representing the cell to open a display of the cell's intracelular chemistry.

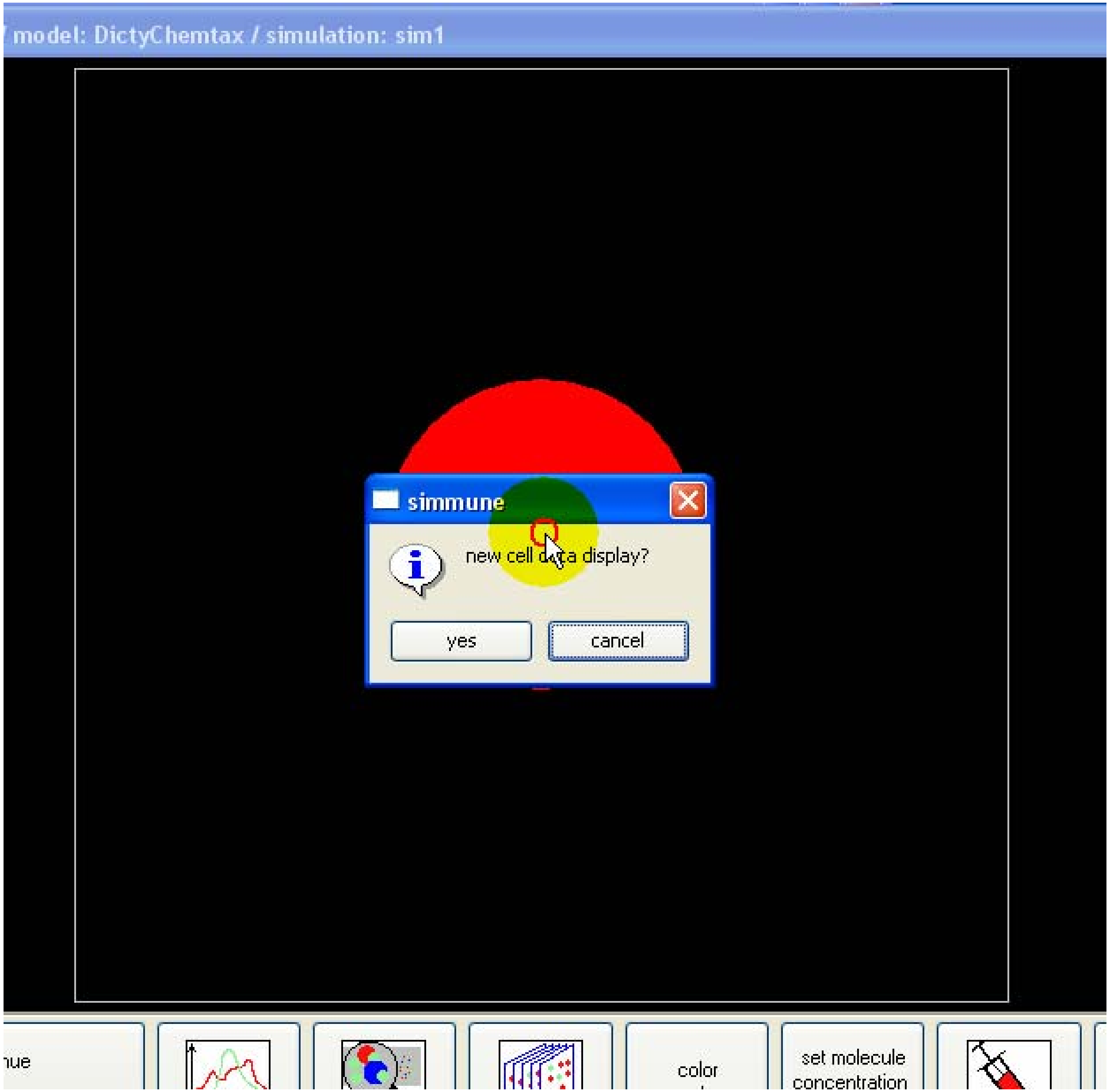

Continue the simulation for some time.

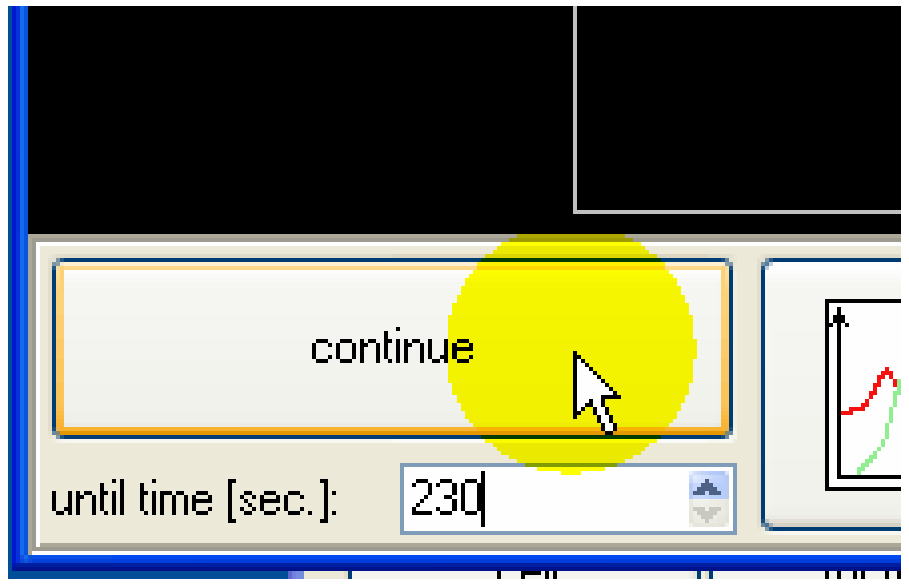

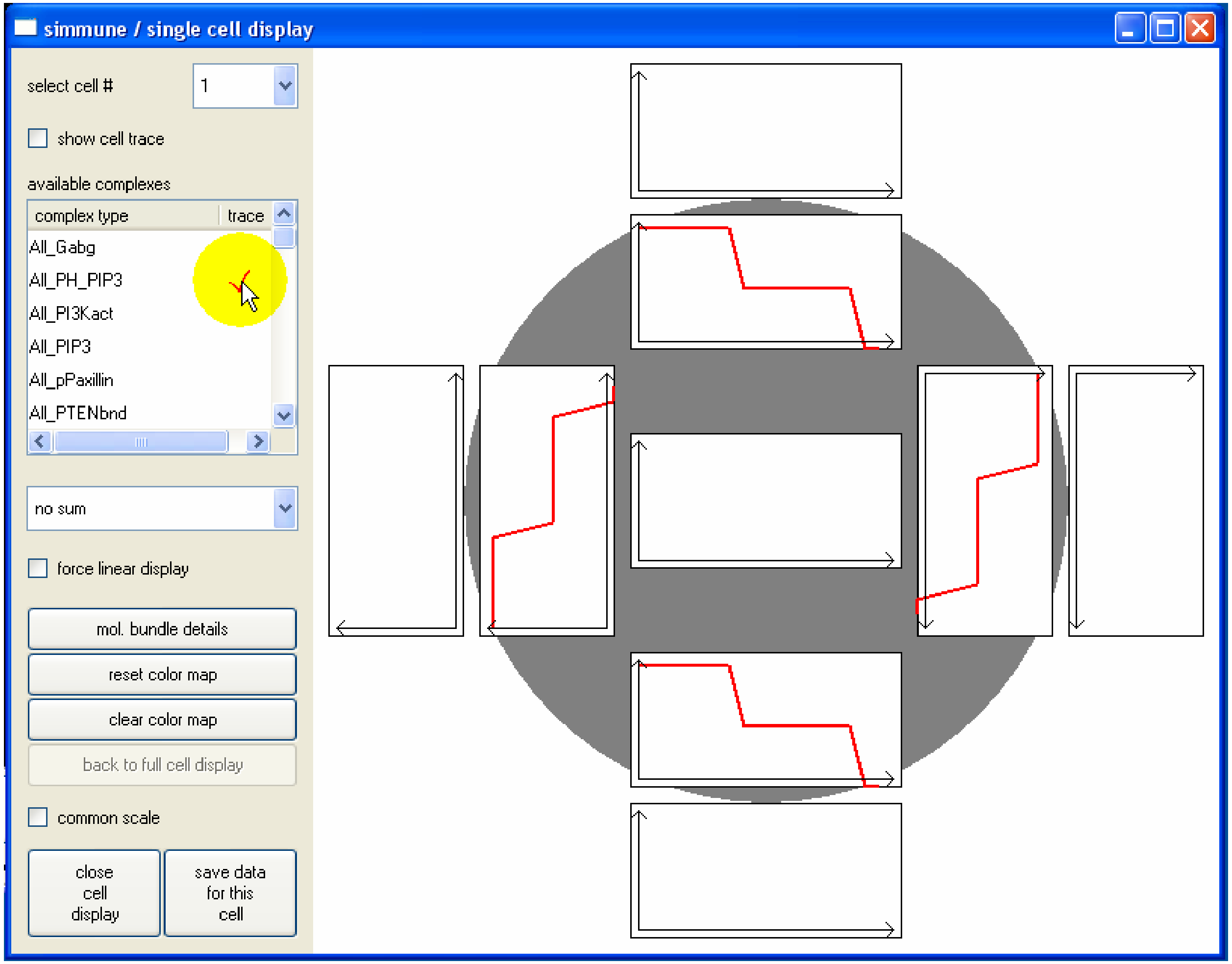

Double-clicking All_PIP3 will assign a color code to the concentration of the bundle consisting of PIP3 in all its binding states (complexes). The concentration changes slowly over time because PIP3 is still equilibrating.

Click on ‘set molecule concentration fields’ in the main simulation window.

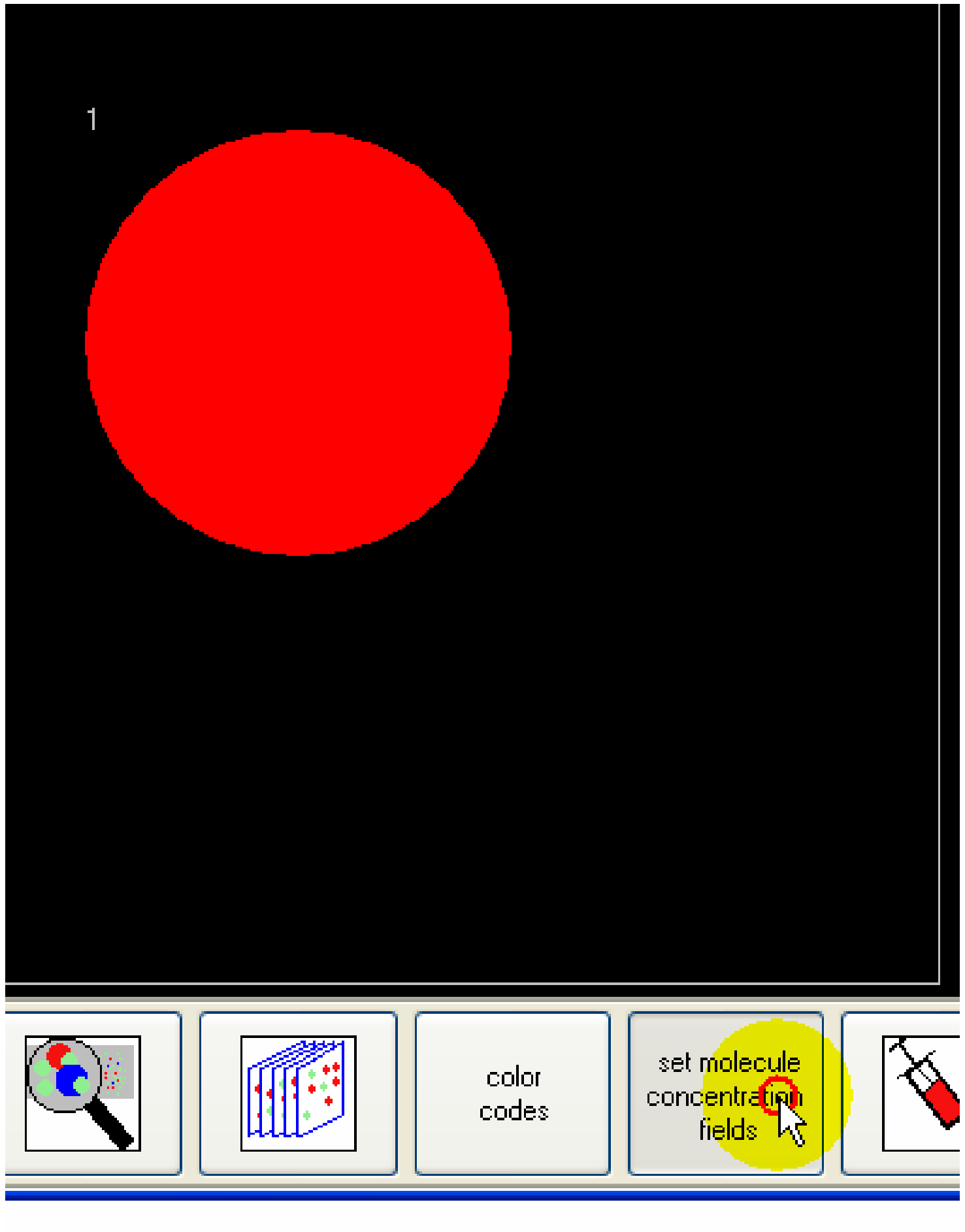

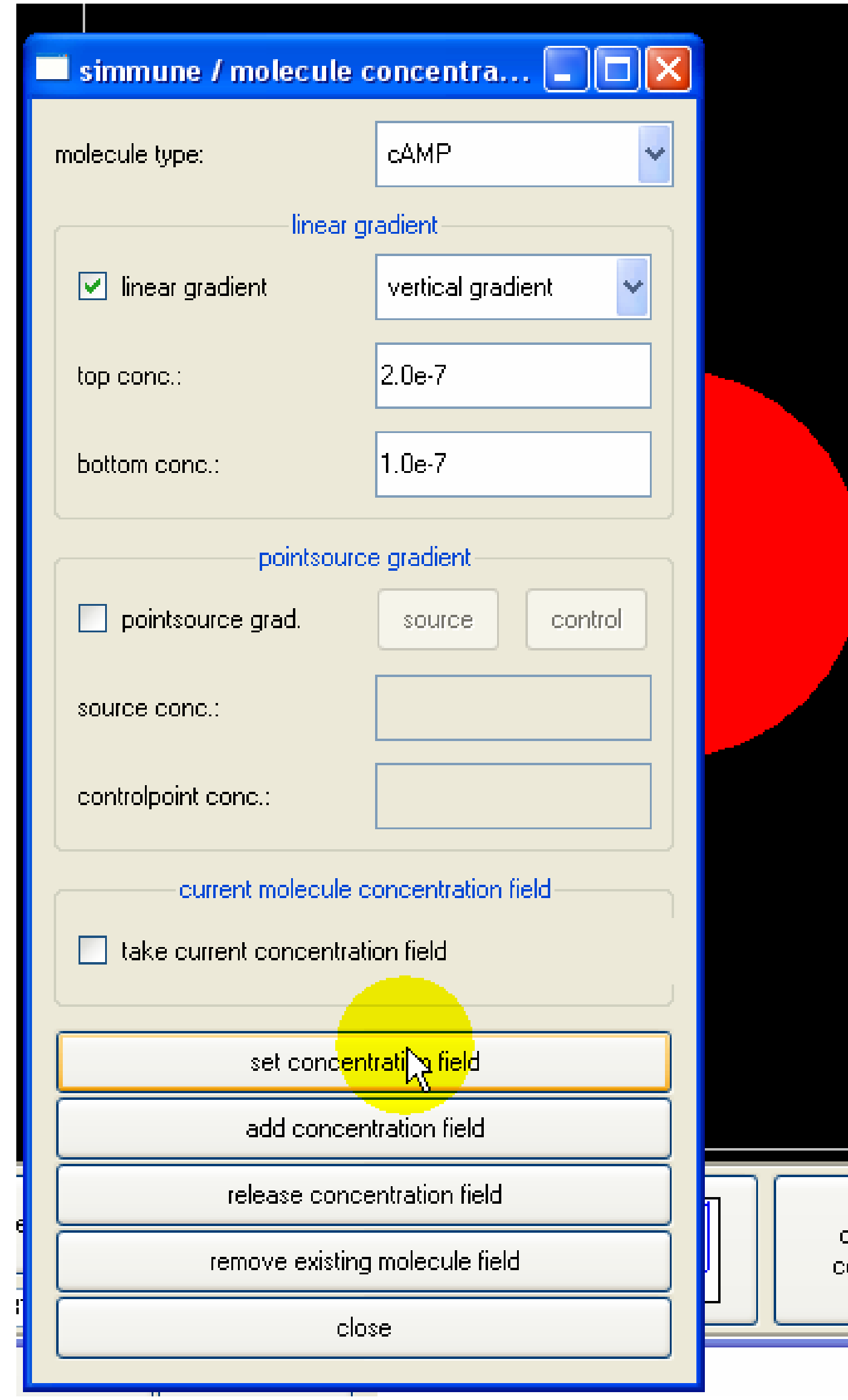

Apply a linear, vertical gradient of cAMP.

The gradient will be visualized by Simmune as iso-concentration lines.

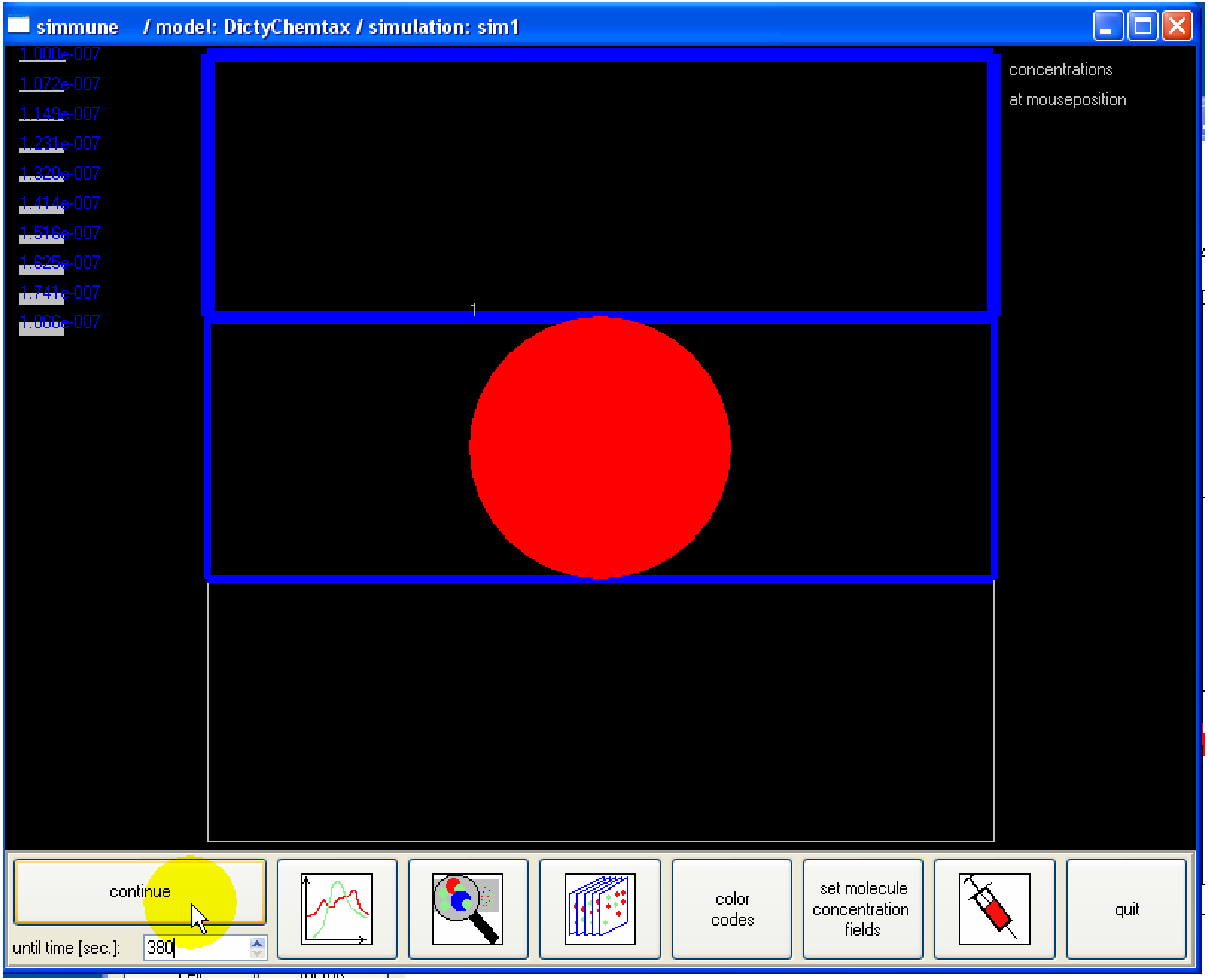

Assign a color to the 'All_PTENbnd' bundle (double-click).
Double-click the upper intracellular region to switch to a more detailed display of that region.

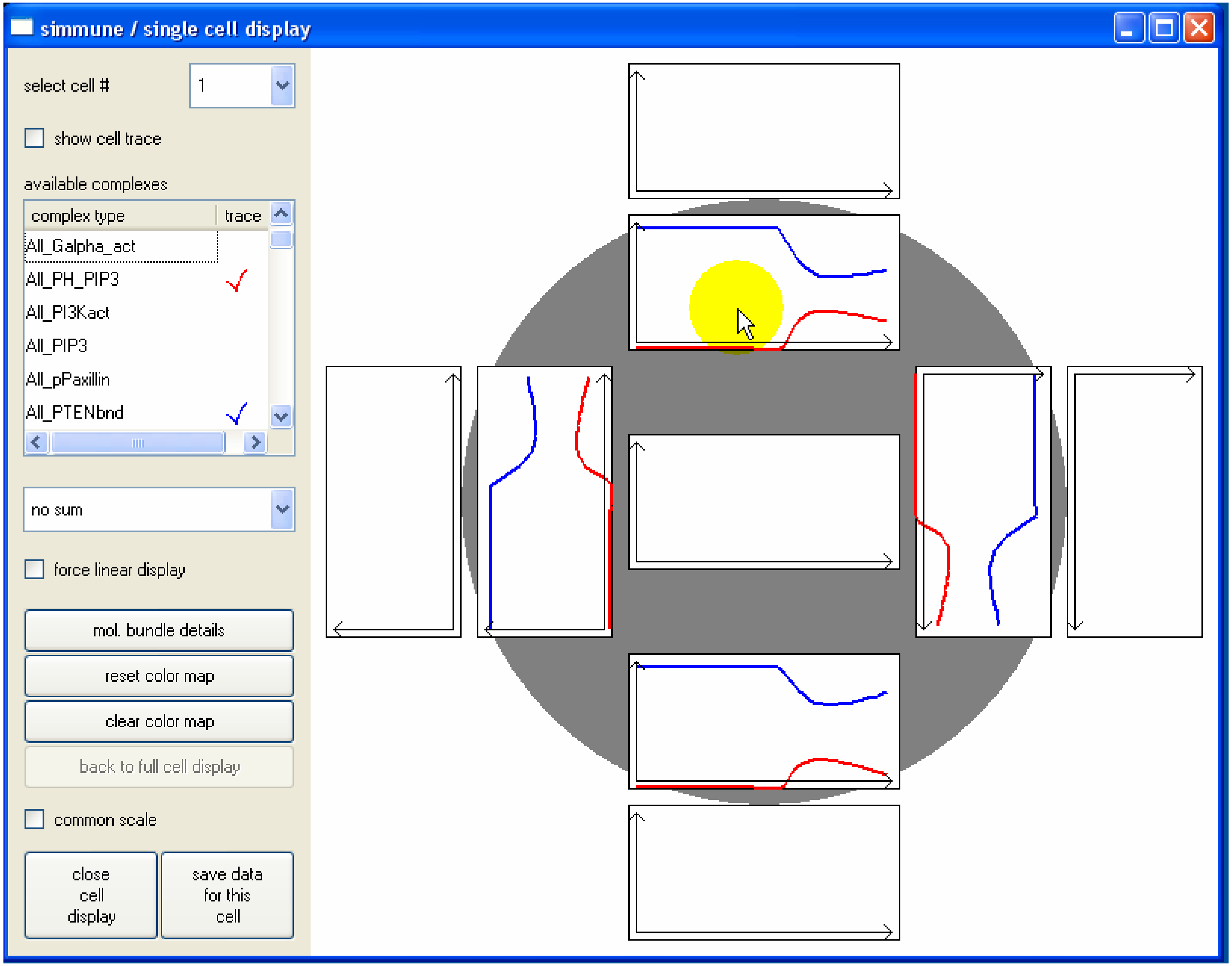

The upper intracellular region:

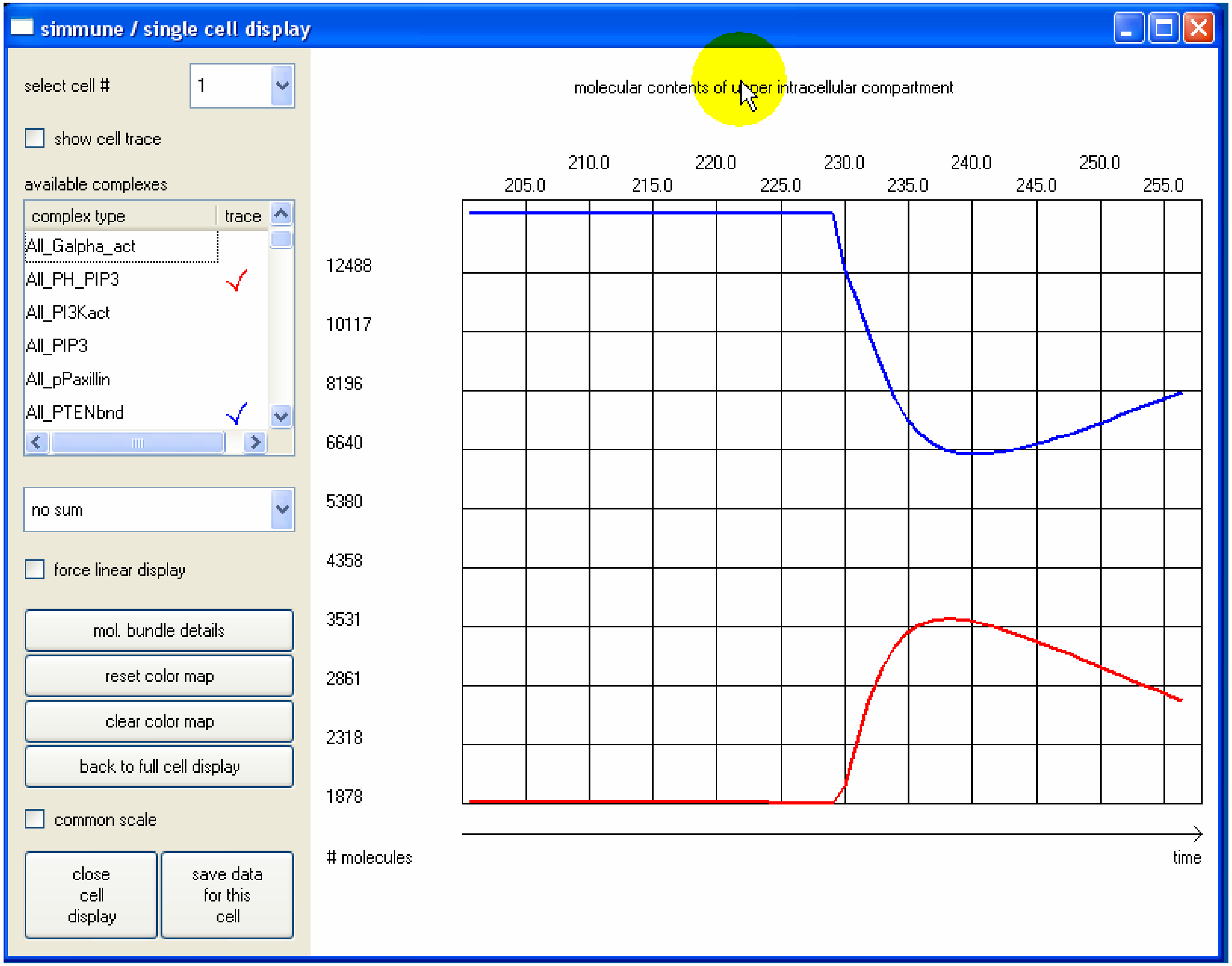

Switching back to the full cell display will show the characteristic multi-phasic responses of PIP3 and membrane bound, active PTEN and how they differ in different regions of the cell.

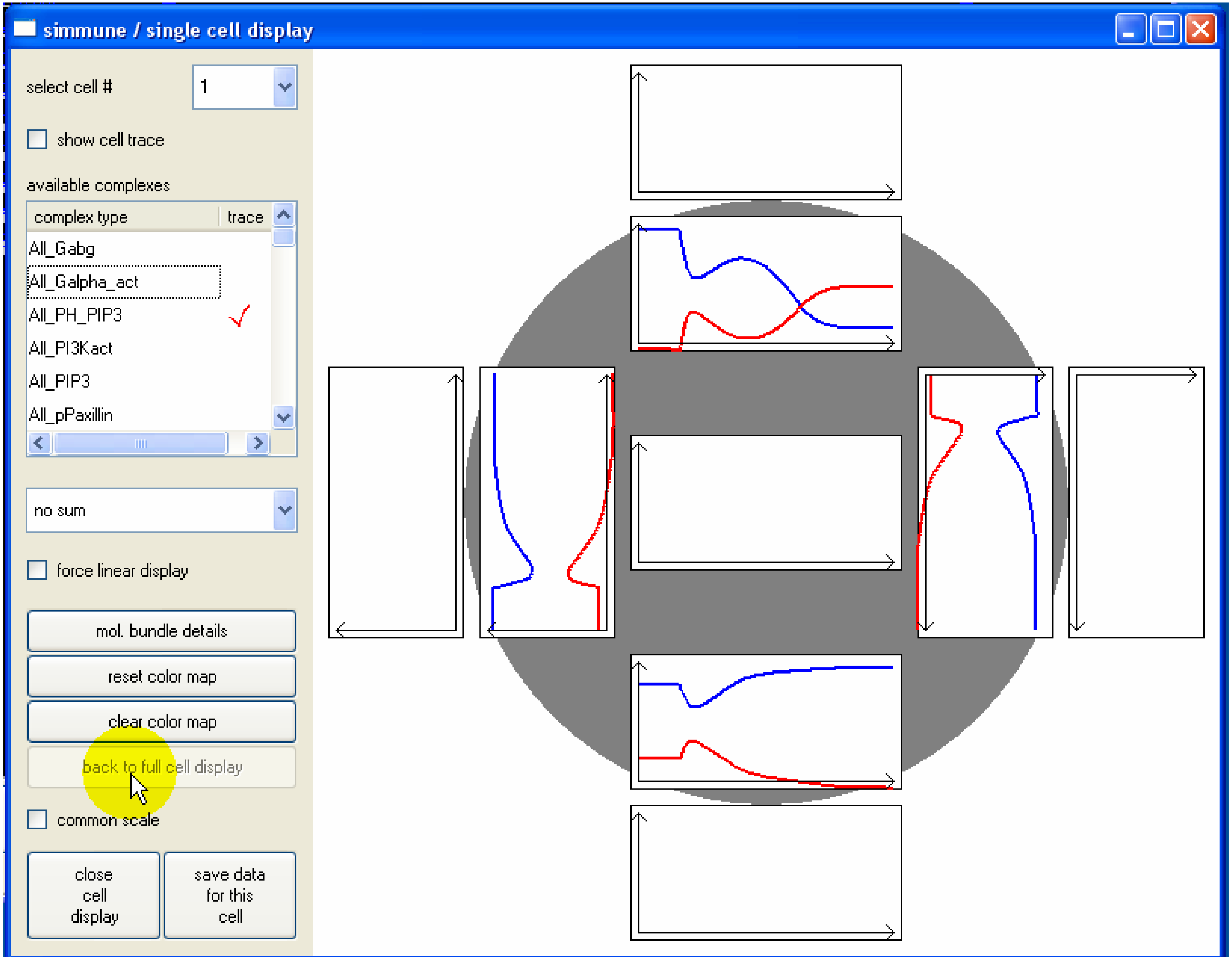

Supplement: Supplementary file 12 [file pcbi.0020082.sd012.pdf]
