# Supplementary material for: Key Role of Local Regulation in Chemosensing Revealed by a New Molecular Interaction-Based Modeling Method
Source: PLoS Comput Biol. 2006 Jul 21;2(7):e82. doi: 10.1371/journal.pcbi.0020082 (PMC1513273; doi:10.1371/journal.pcbi.0020082)

Simmune tutorial 6

Adding parameters to the full Dicty Chemtax model that can varied by the software for parameter scans

(1) Open the DictyChemtax model.

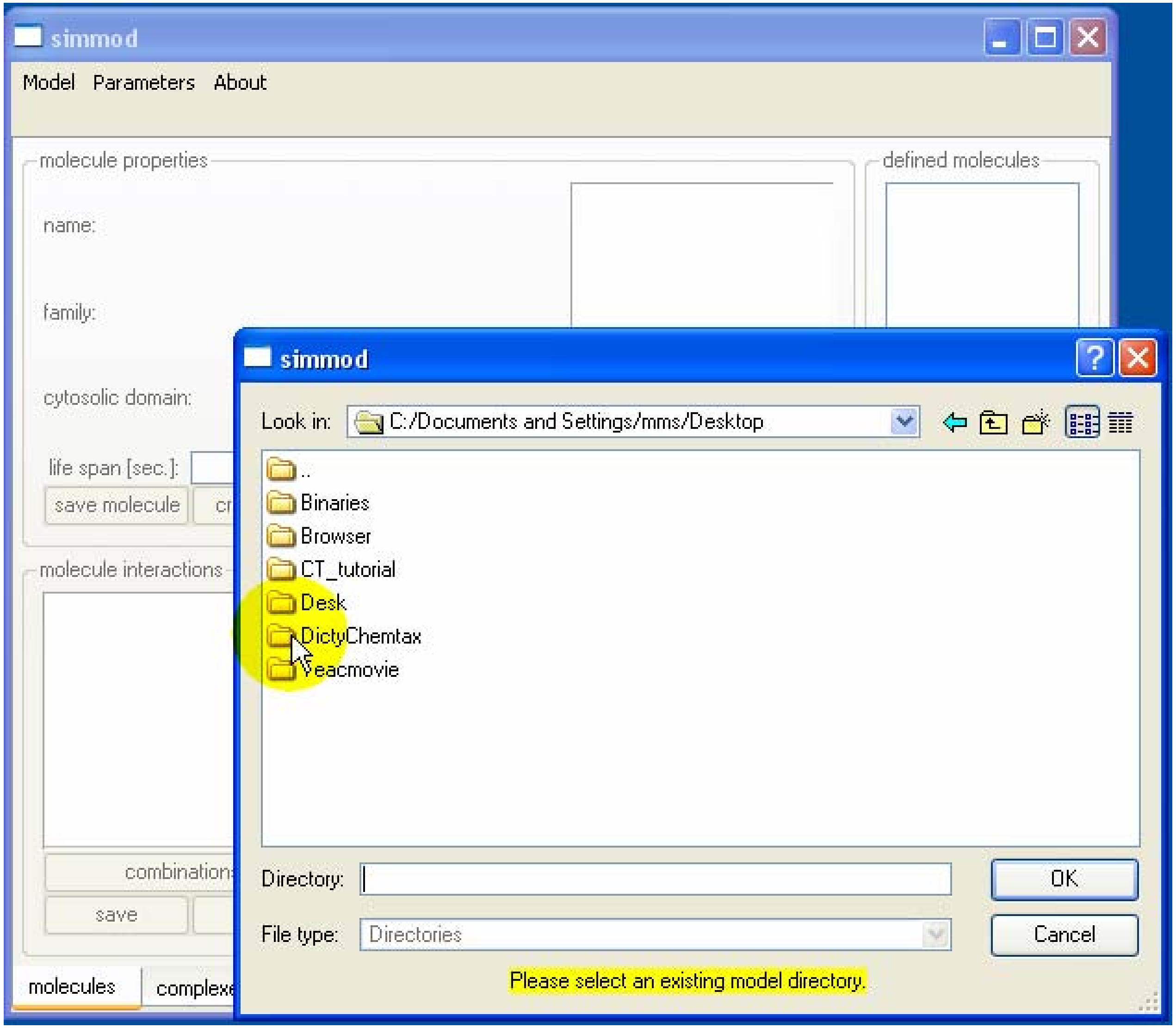

(2) Click on the ‘Parameters’ menu and enter the name for the new parameter. Here we want to vary the association rate between Gbetgamma and PTENph. We call the parameter therefore ‘Gbg_PTENph_assoc’ (but the name is arbitrary).

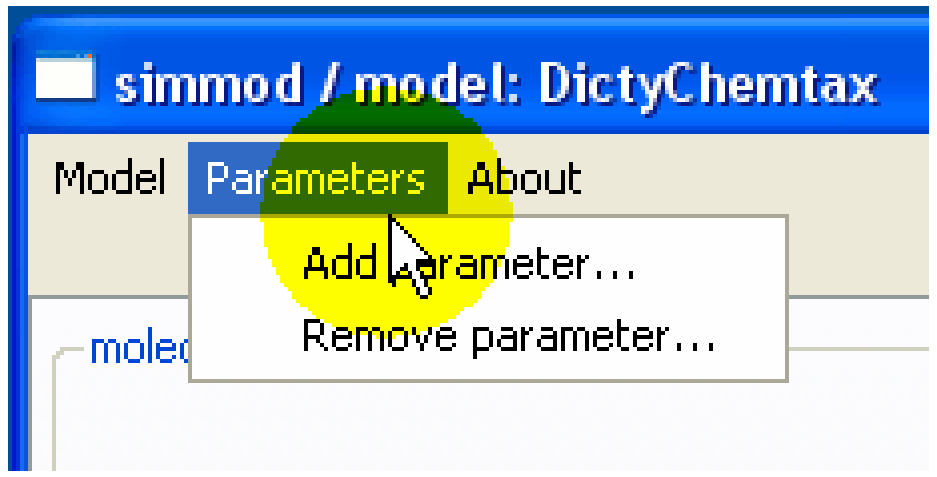

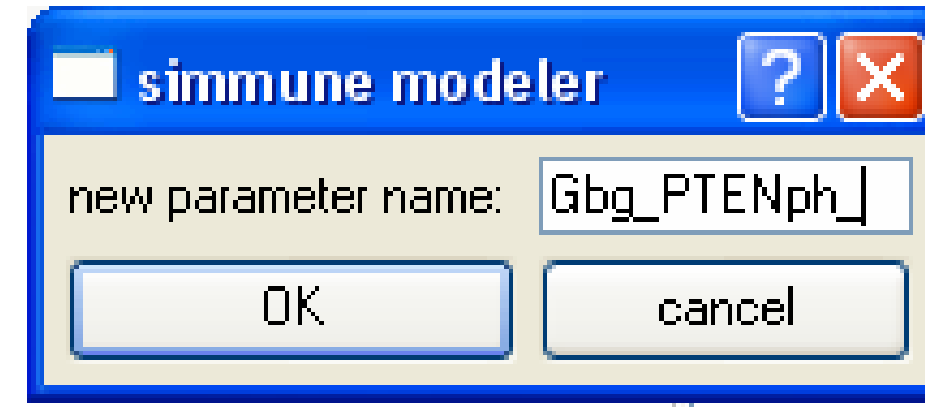

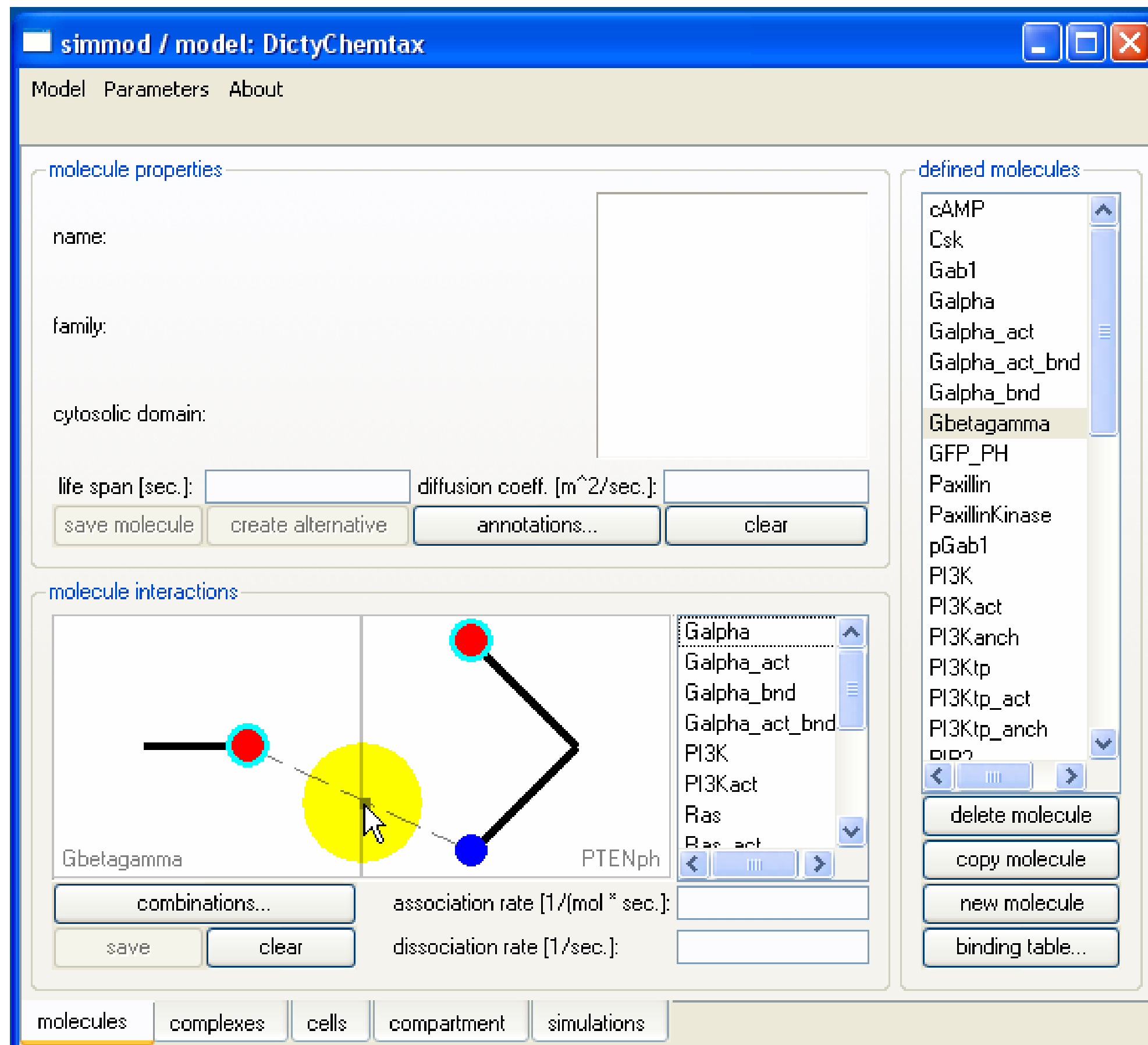

(3) Drag Gbetagamma and PTENph into the ‘molecule interactions’ field and double-click the small rectangle at the line representing their interaction.

(4) Click ‘binding properties’ and then select the parameter we created (Gbg_PTENph_assoc) to be ‘attached’ to the association rate of the molecular interaction.

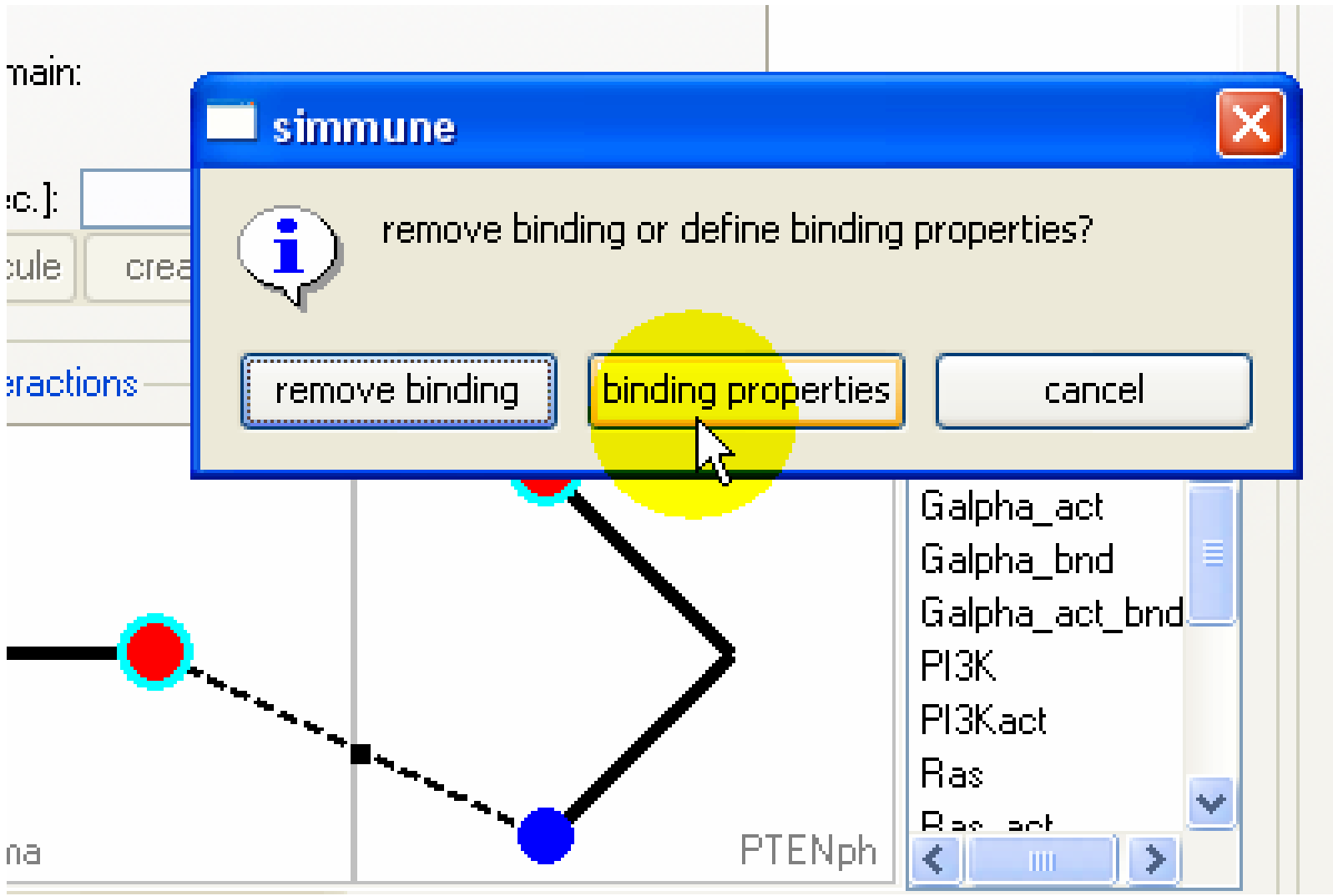

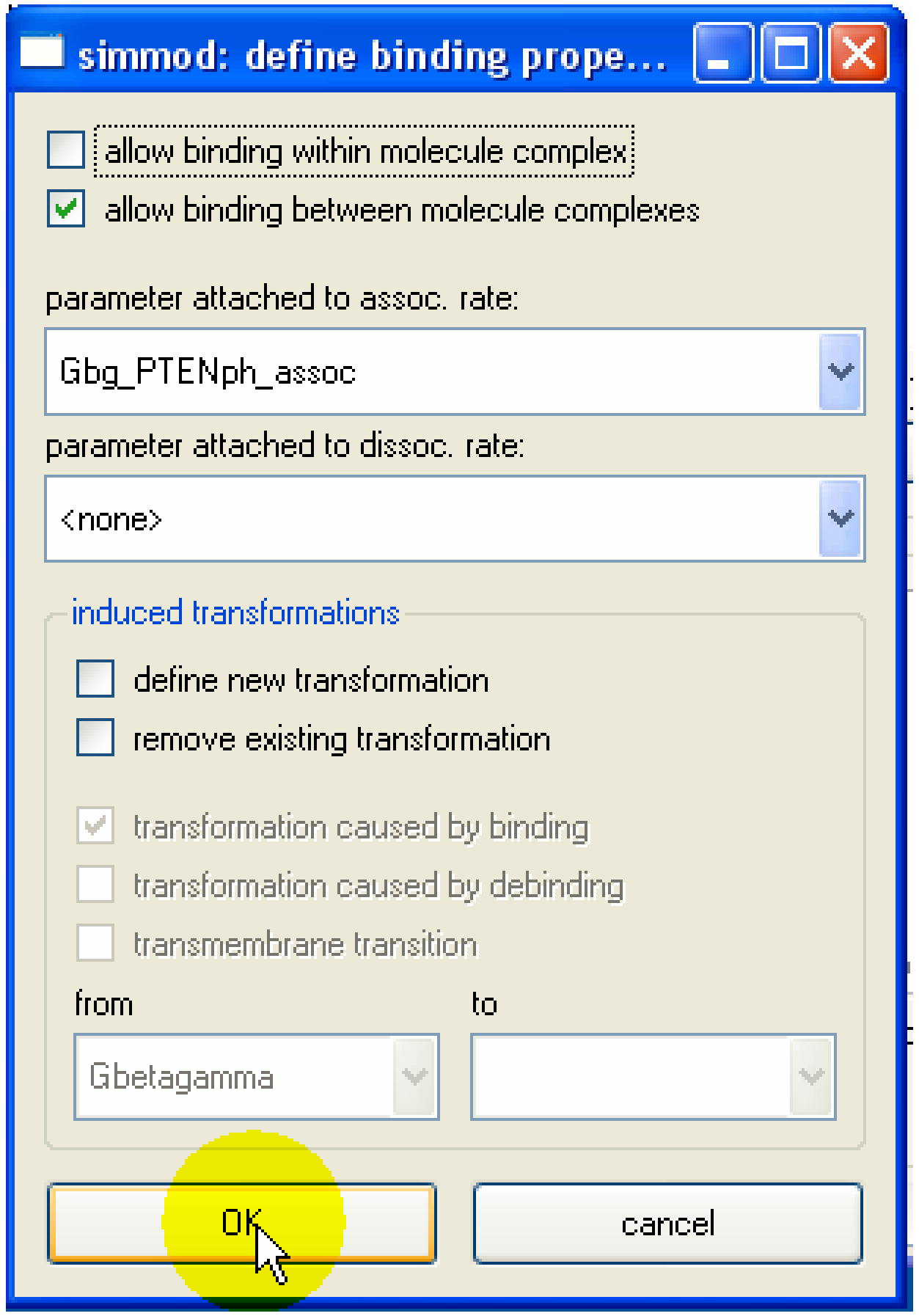

(5) Next, we create a paremeter to vary the number of Csk molecules in the cell.

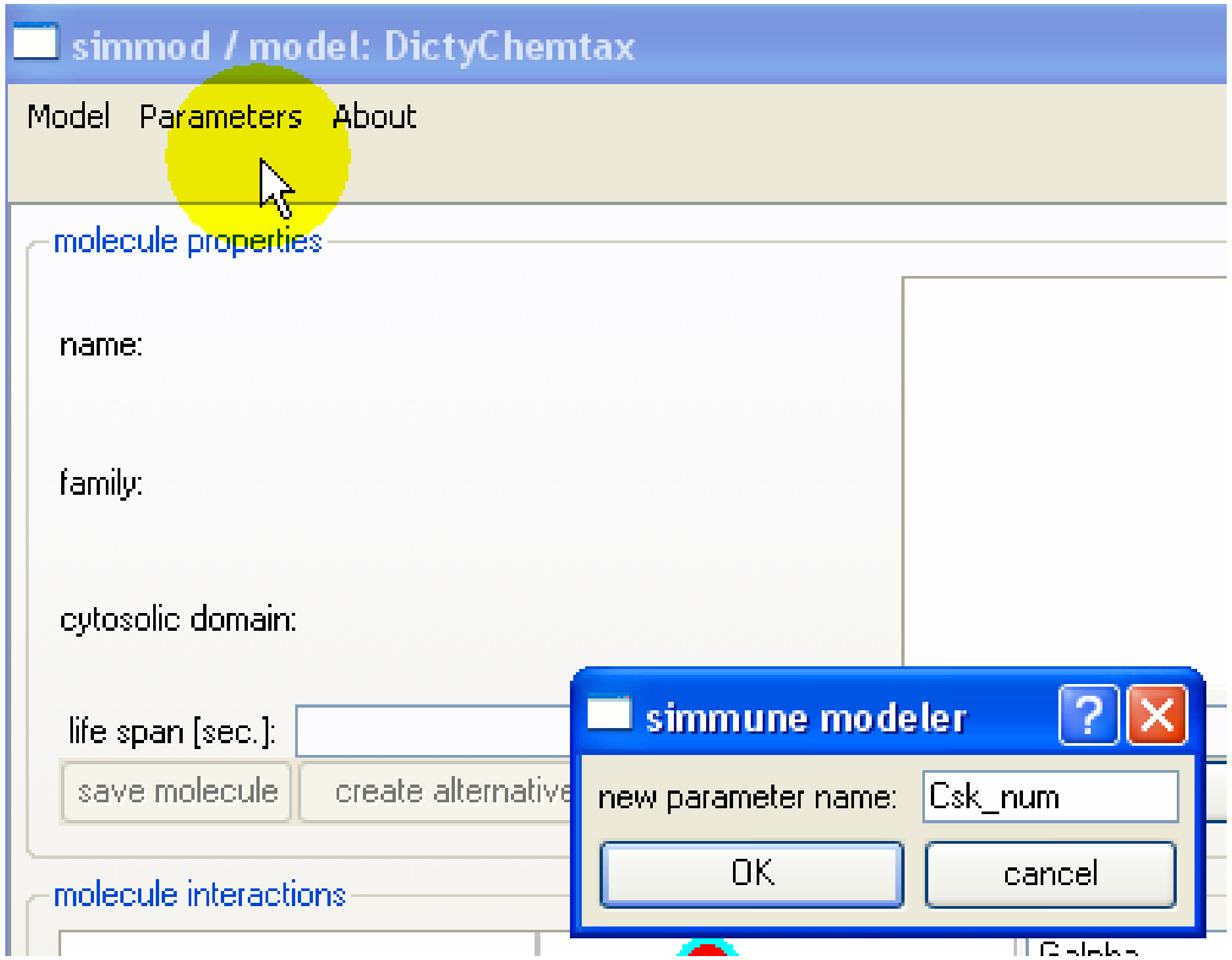

(6) Go to the 'cells' tab, double-click Dicty1 in the defined cell types list and then 'init' in the list of mechanisms of this cell.

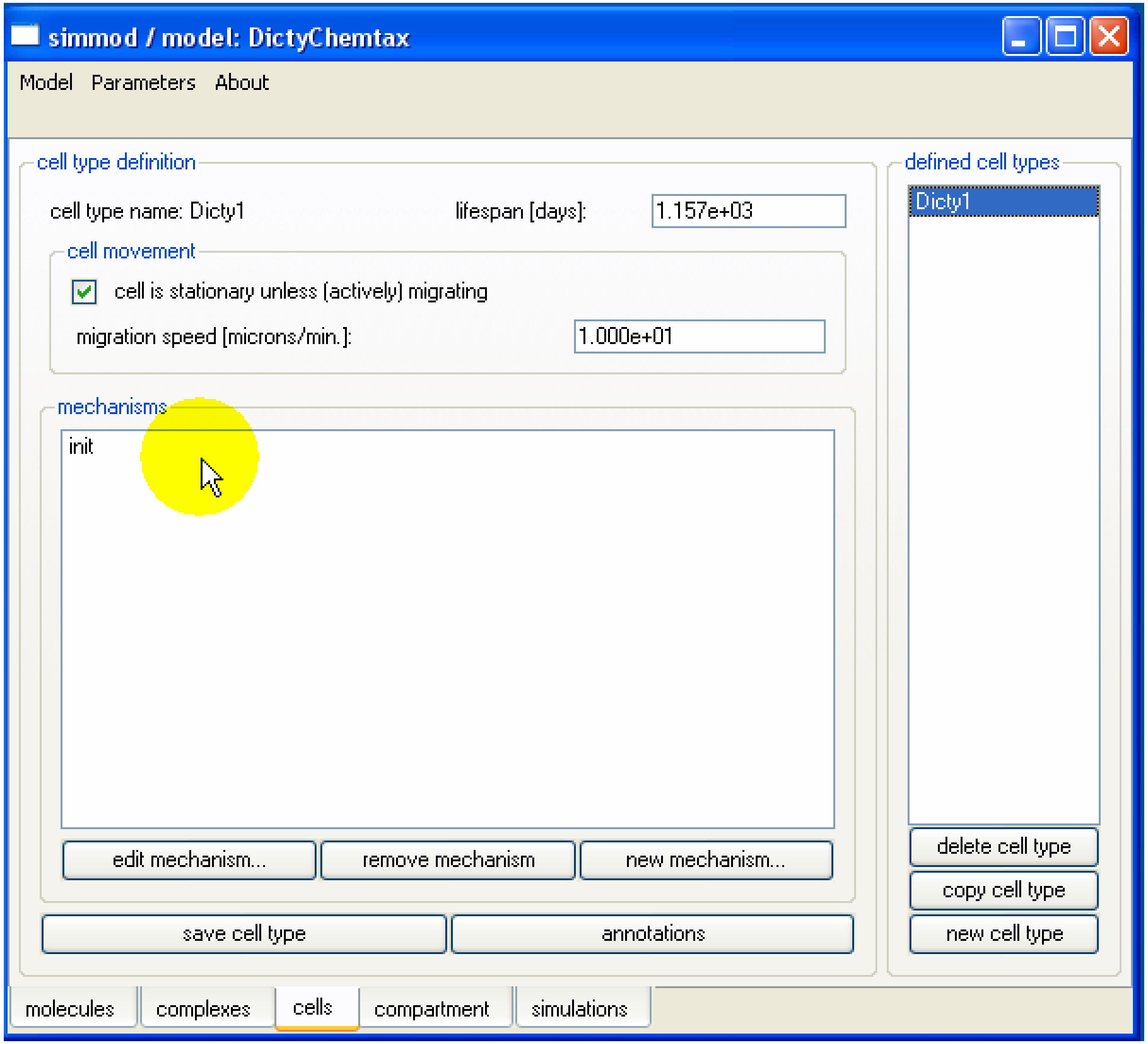

(7) In the ‘response’ list of the mechanism, select the action that makes the cell produce Csk and then click the ‘amount’ section of the header.

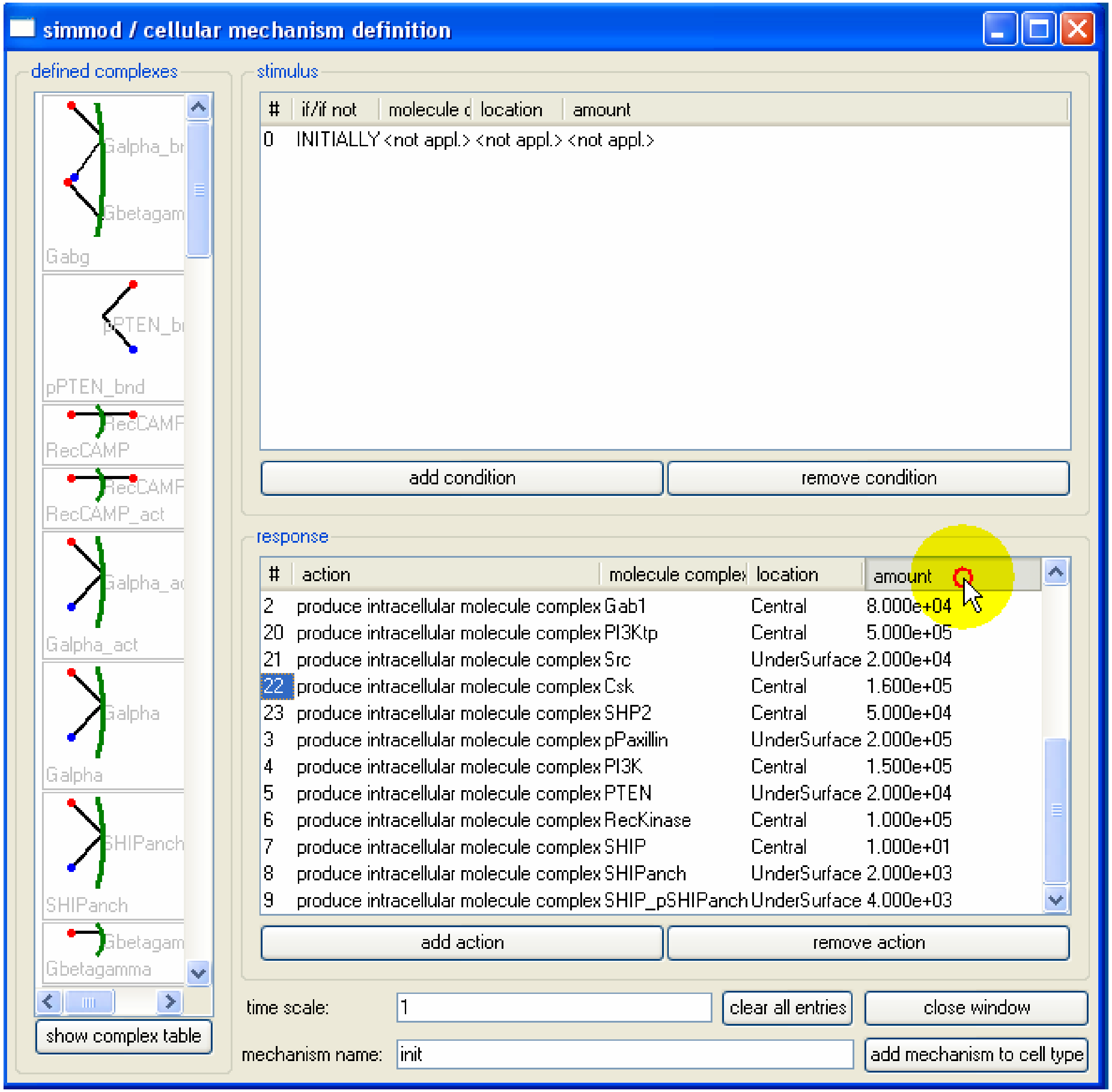

(8) Set the standard amount (here: 100000) and then select the parameter to be attached to the amount of molecules of type Csk.

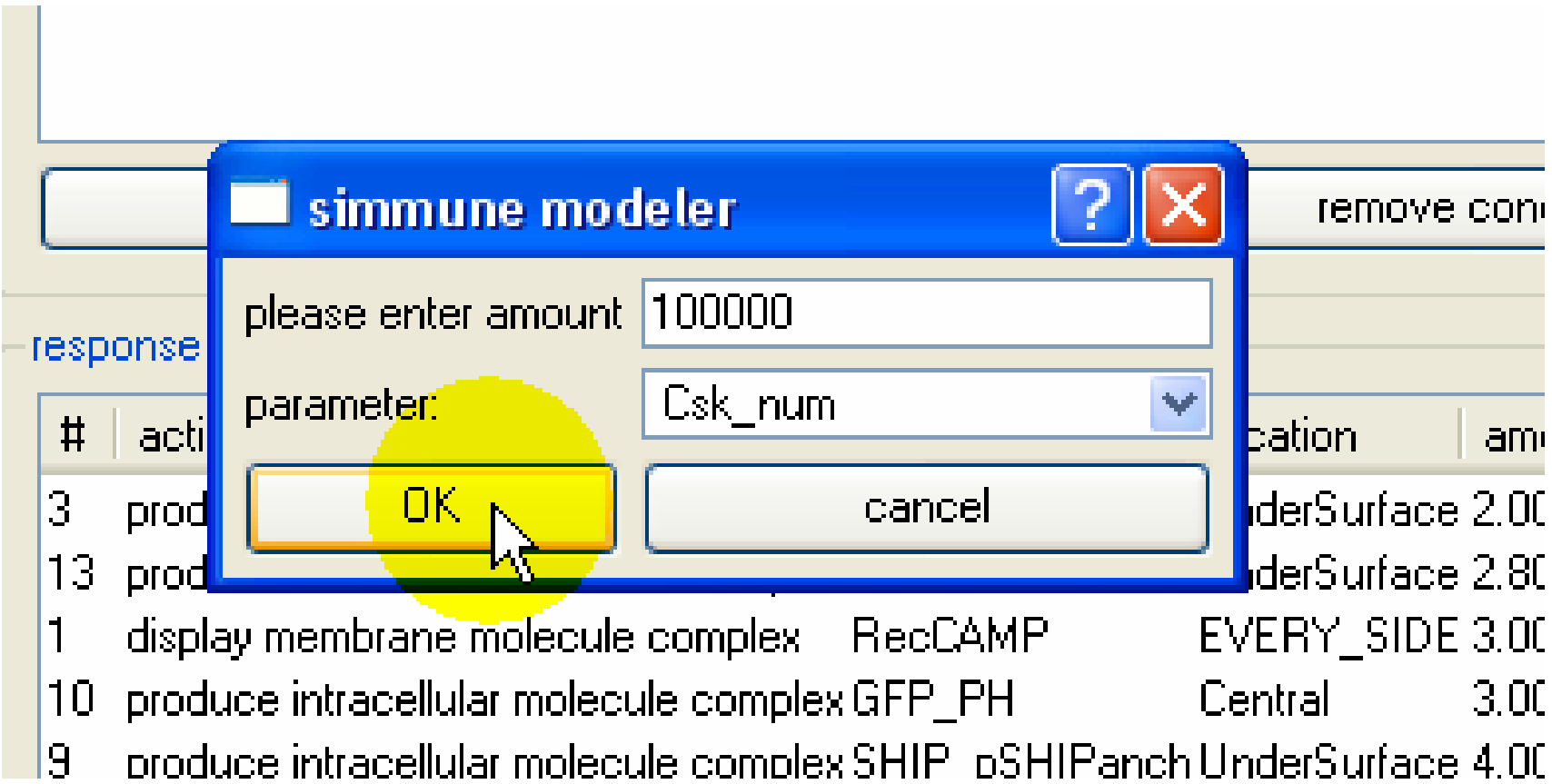

(9) Click 'add mechanism to cell type'.

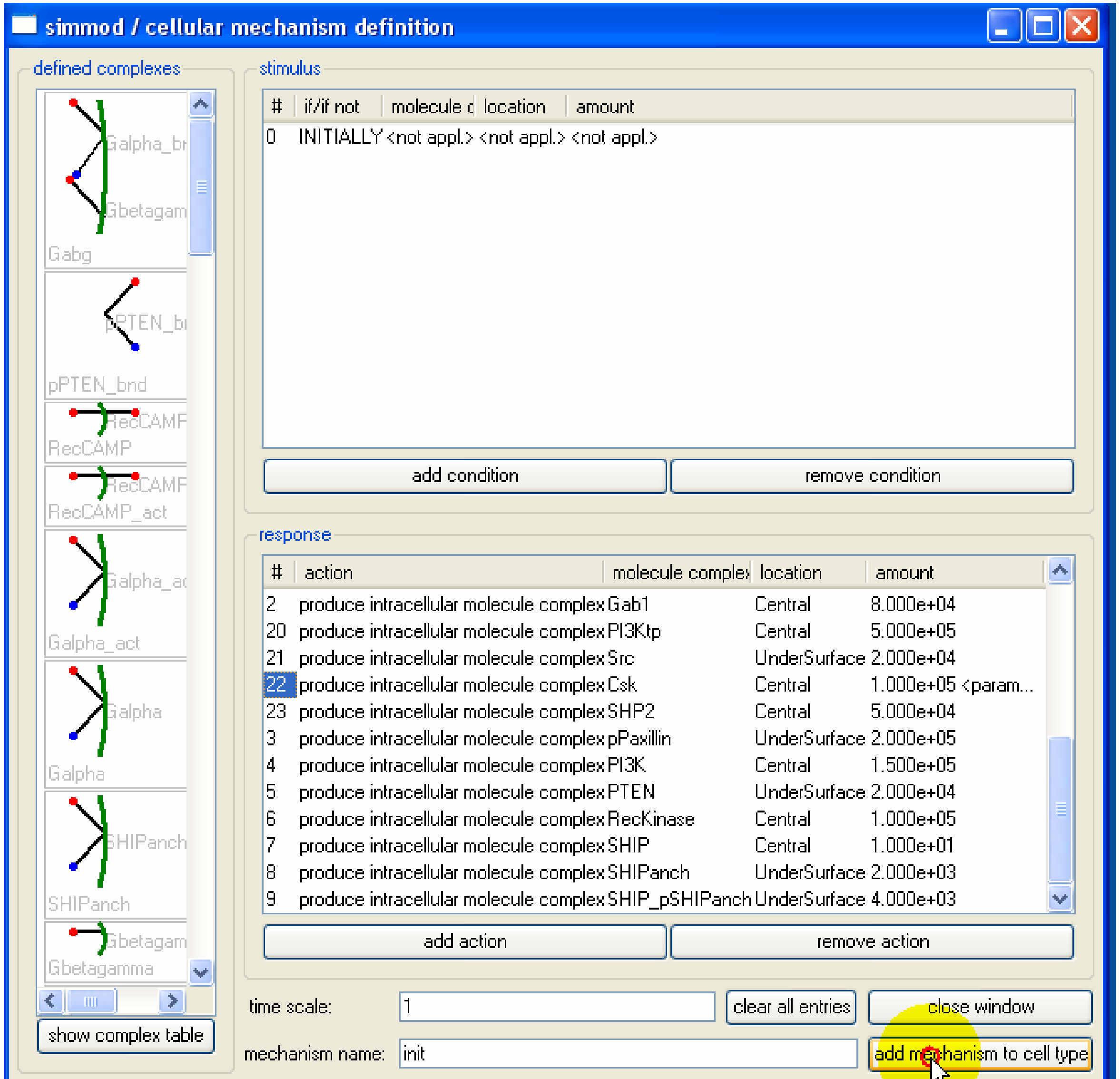

(10) Confirm that you want to Replace the original mechanism

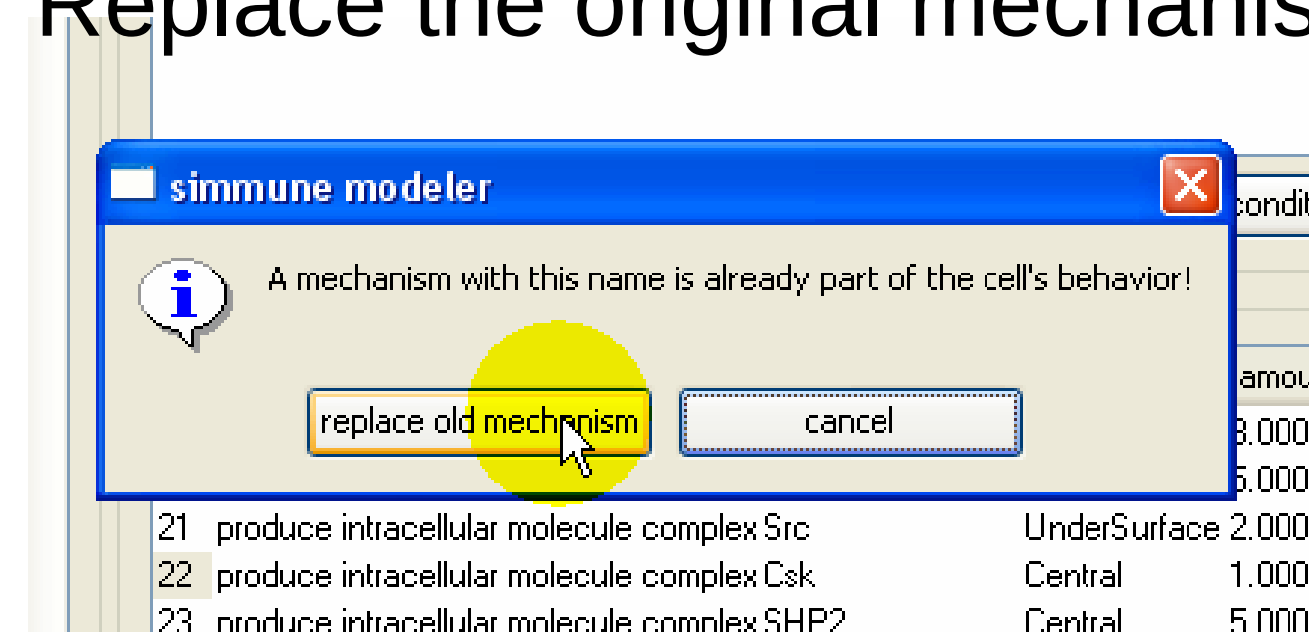

(11) Save the changes.

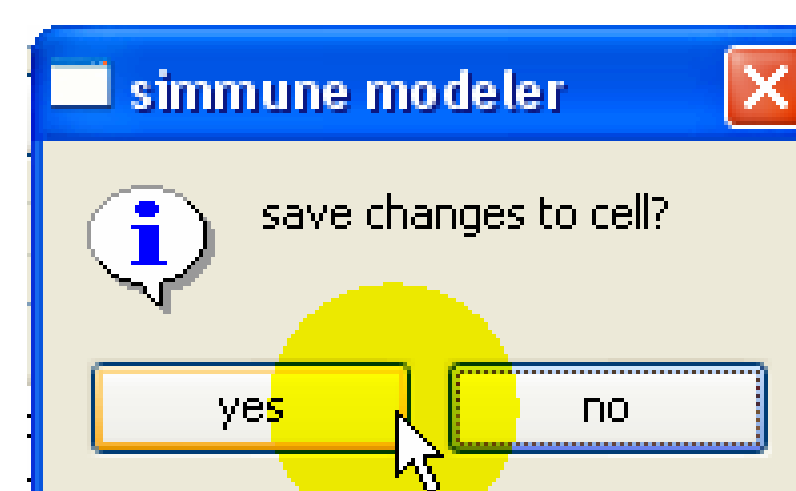

(12) Go to the ‘simulations’ tab and create a new simulation.

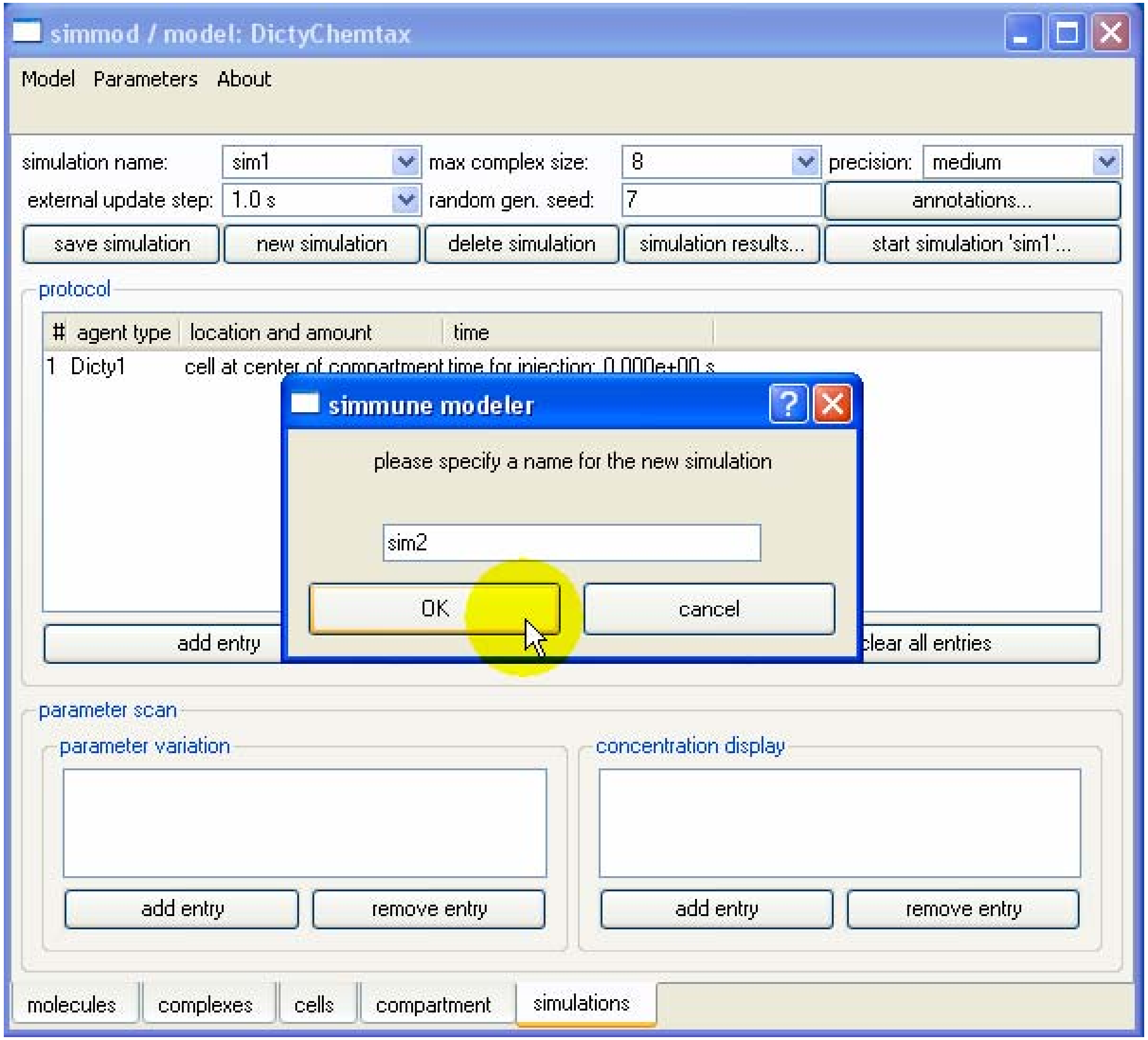

(13) Select the new simulation to edit its properties.

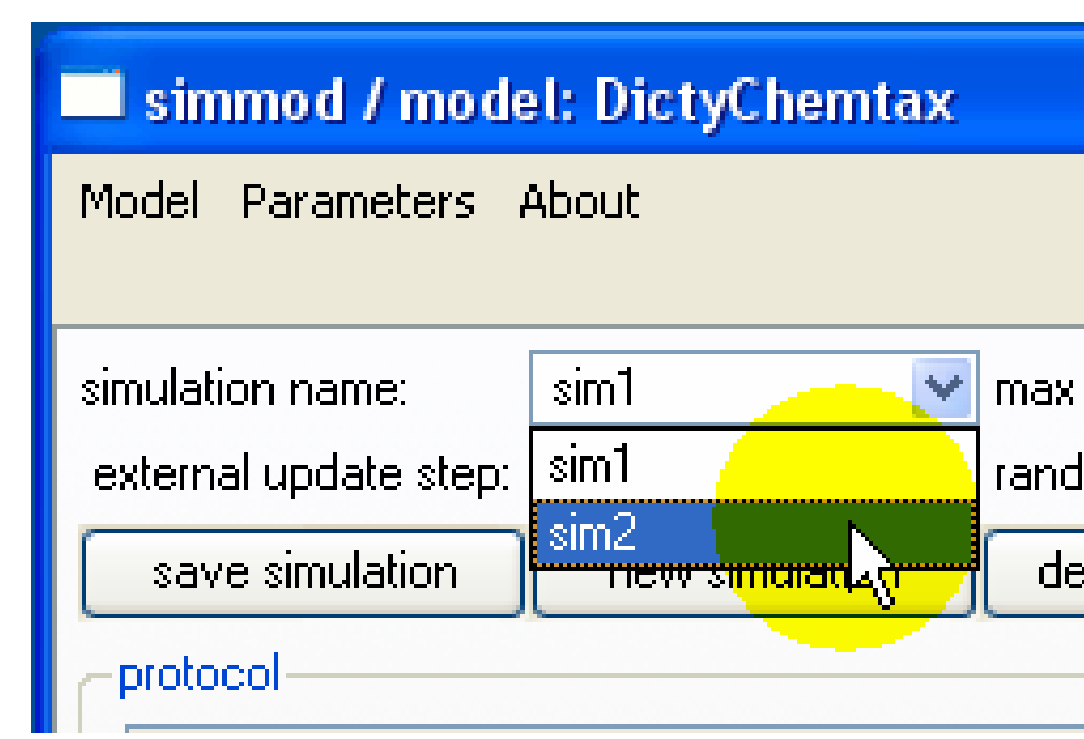

(14) Click ‘add entry’ in the protocol list.

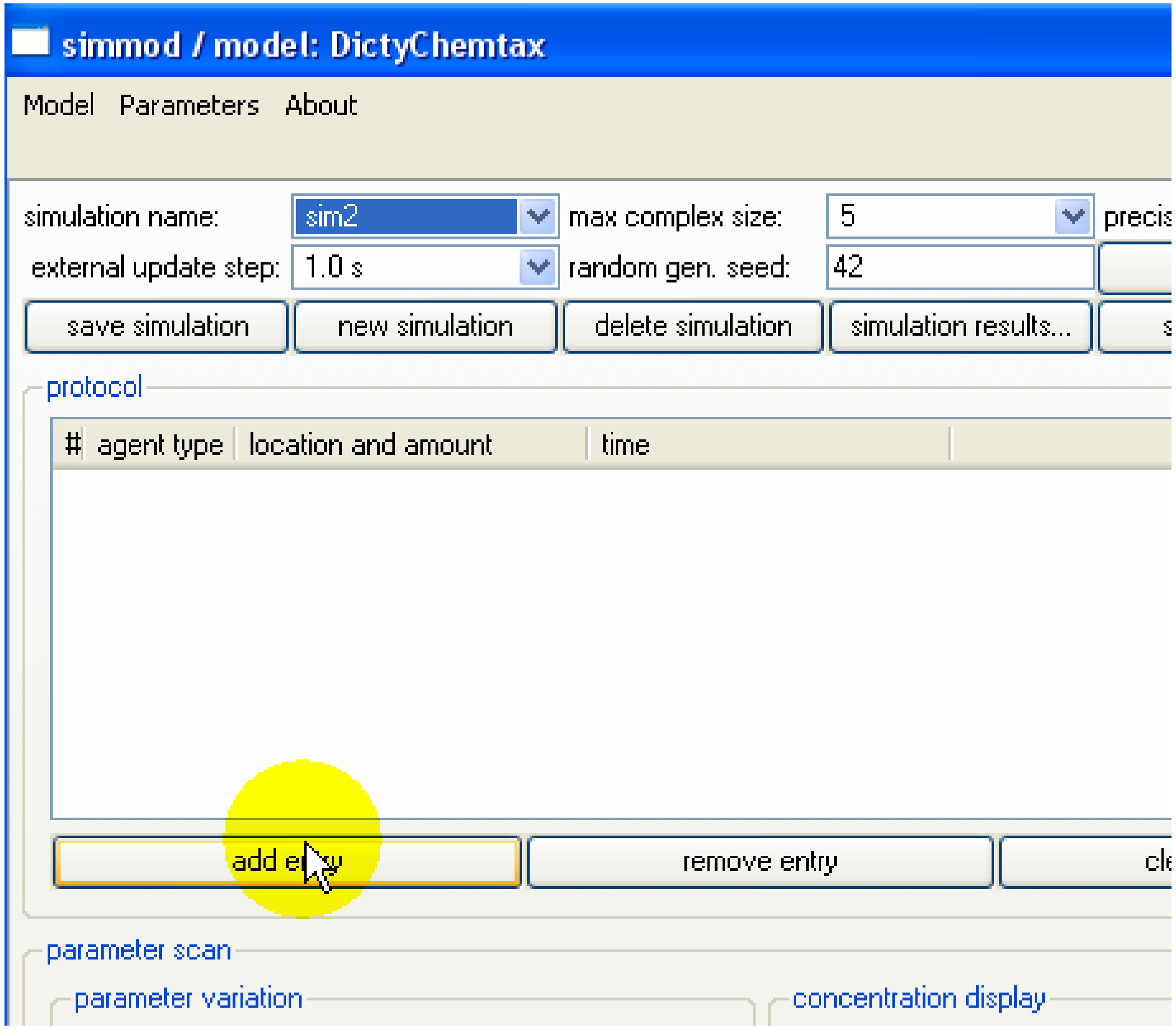

(15) Click the ‘agent type’ section of the protocol header.

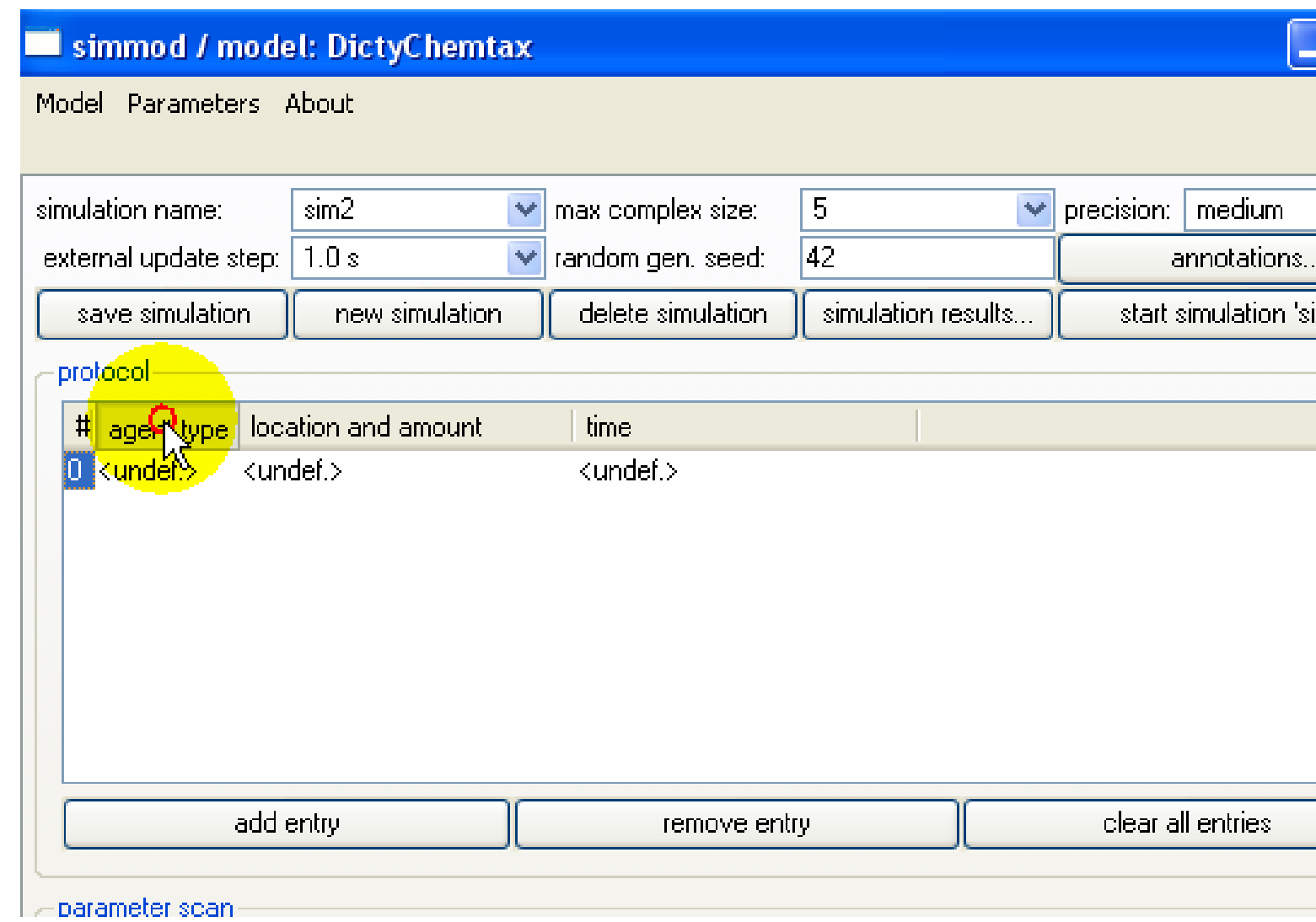

(16) Select agent type Dicty1.

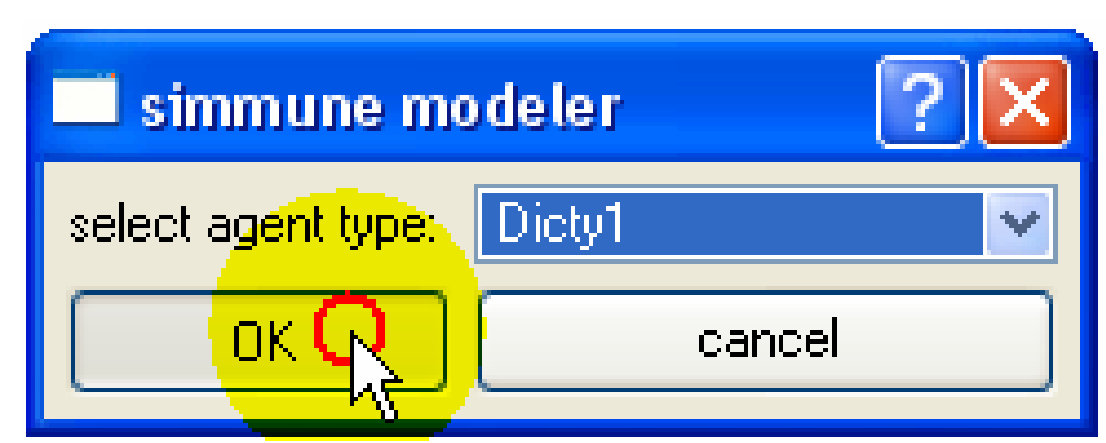

(17) Click ‘location and amount’ and select ‘center’ (the software then automatically chooses 1 as amount as there can be only one cell at the compartment center).

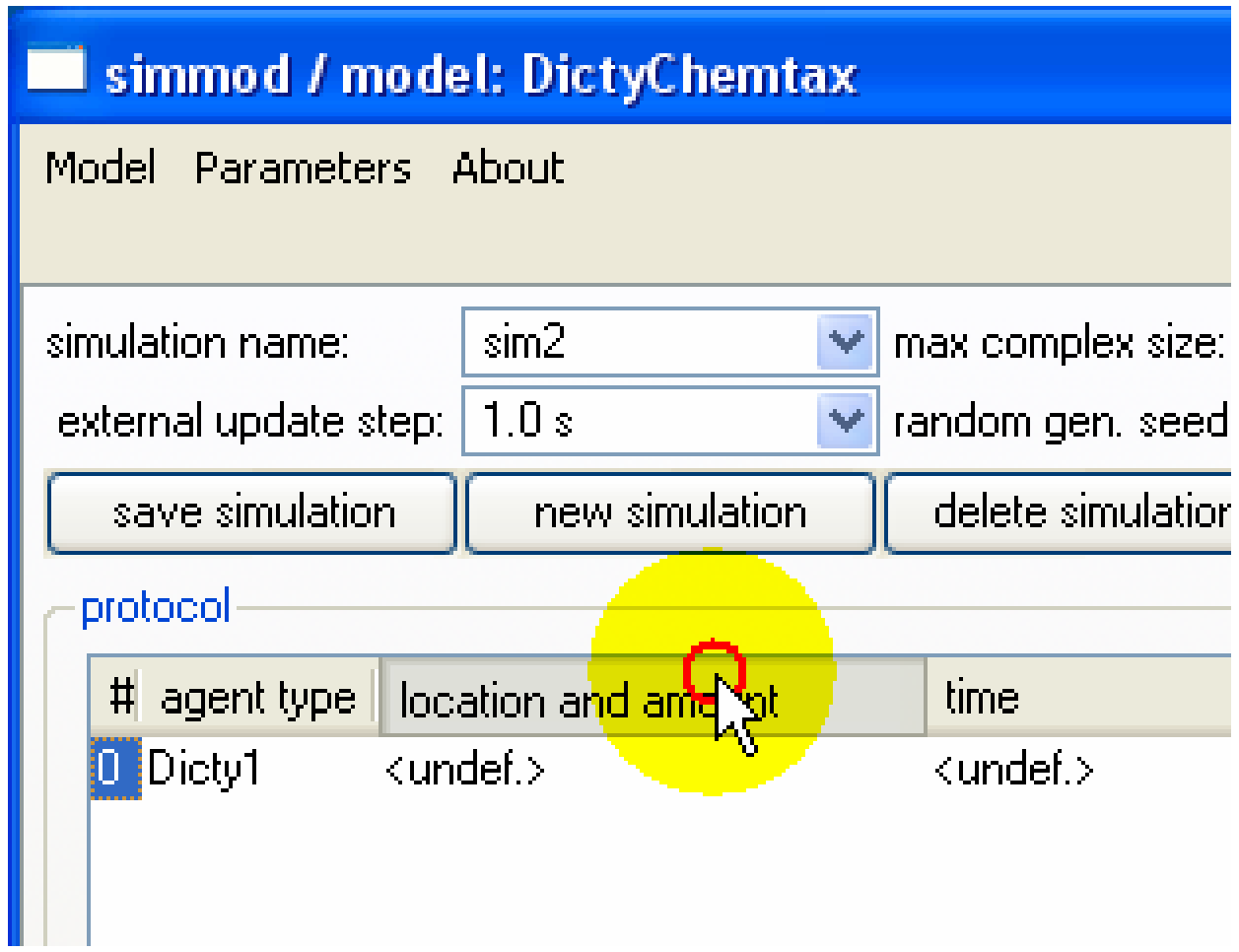

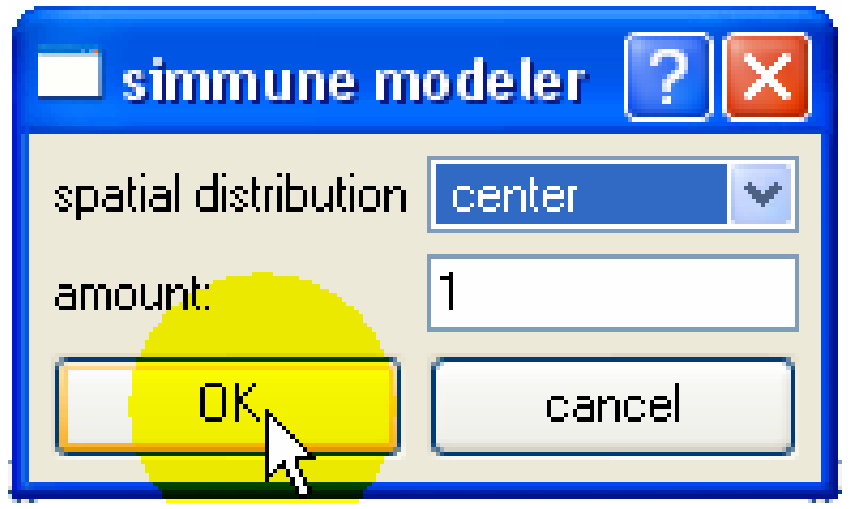

(18) Choose 0 as time (the cell will be placed into the compartment at the beginning of the simulation).

(19) Create a protocol entry for cAMP. Select a vertical gradient with concentration kept constant (check ‘freeze concentration’) and concentrations at the top and bottom of the compartment as shown below.

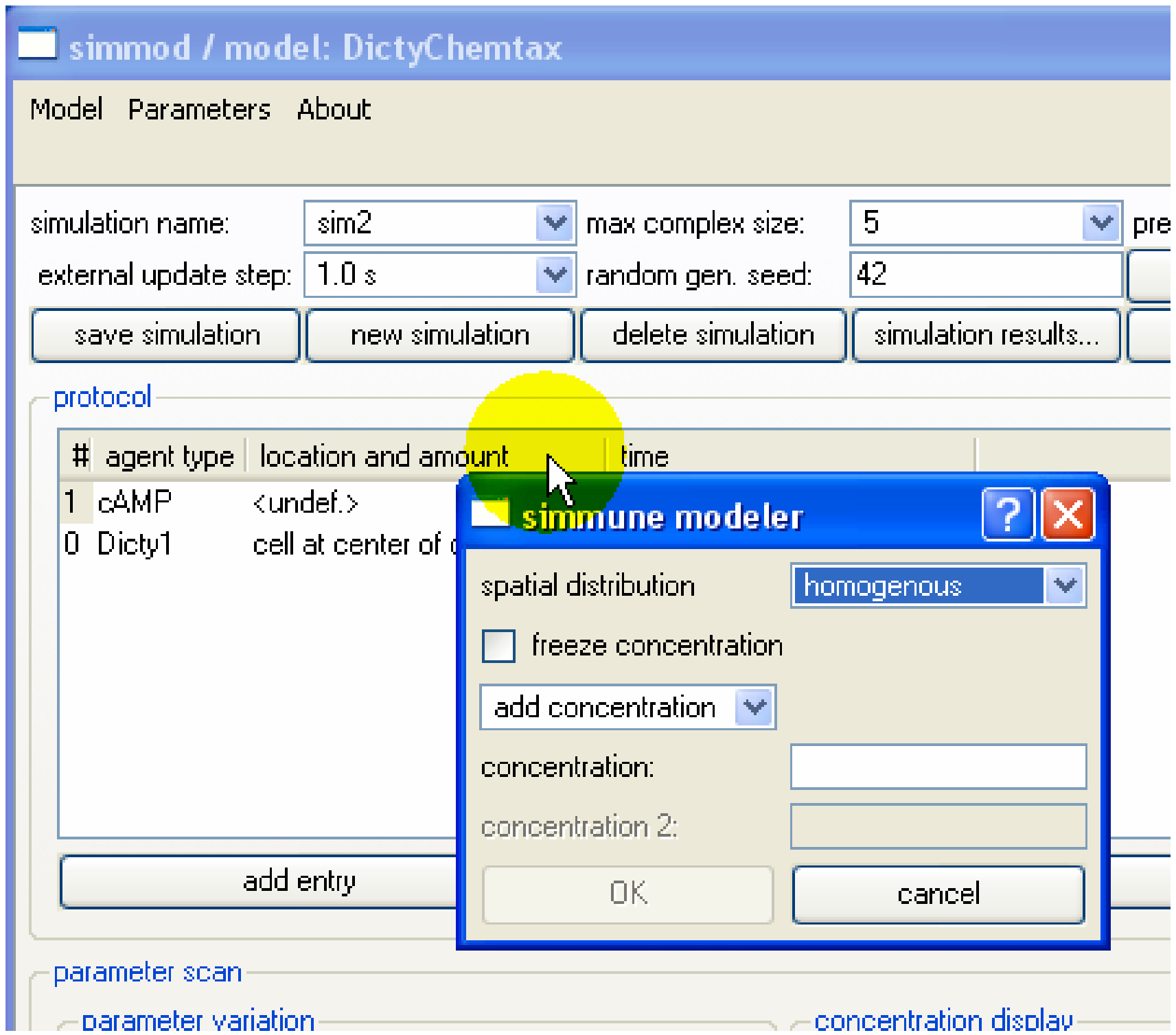

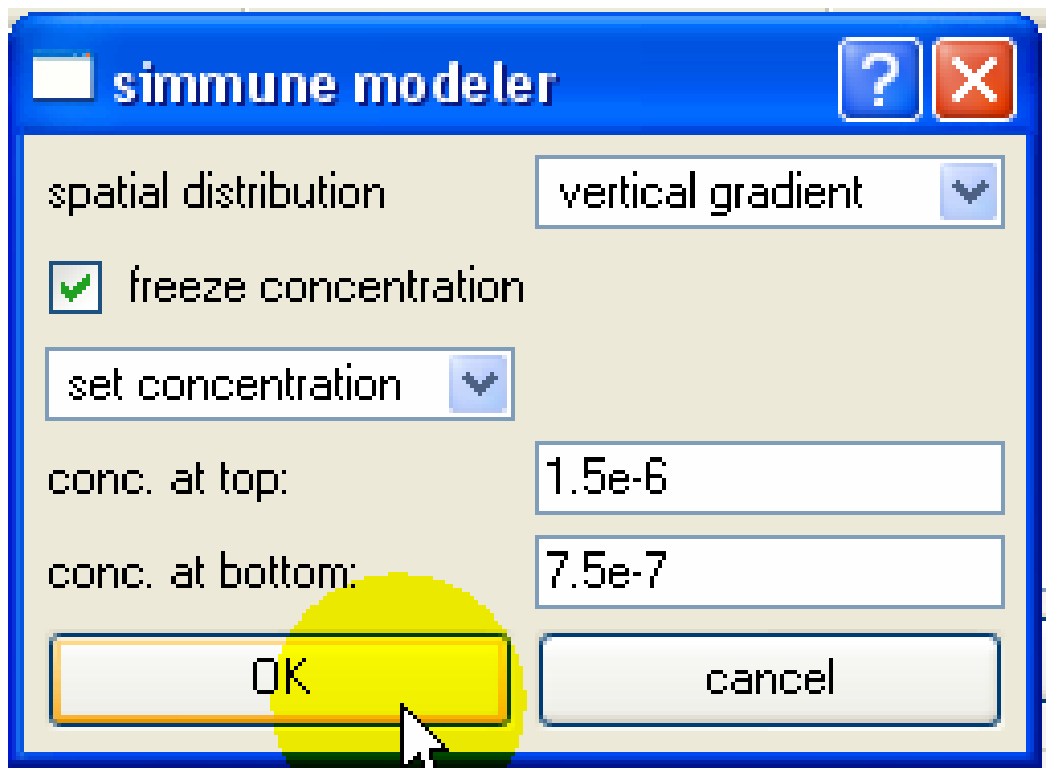

(20) Choose 150 as time (the gradient will be created after the cell has had some time to approach intracellular biochemical equilibrium).

The complete protocol should now look like this

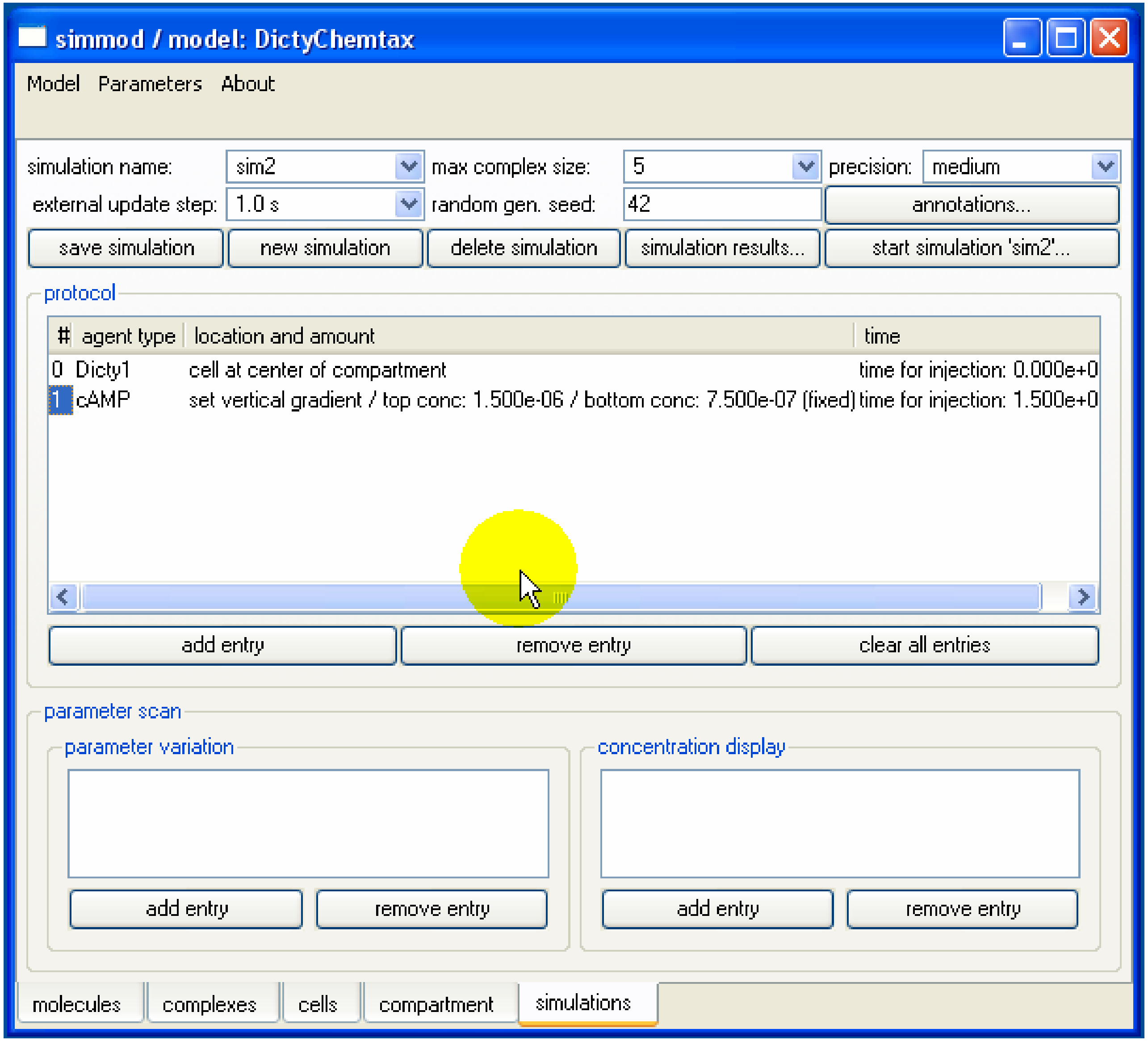

(21) Click on ‘add entry’ in the parameter variation list.

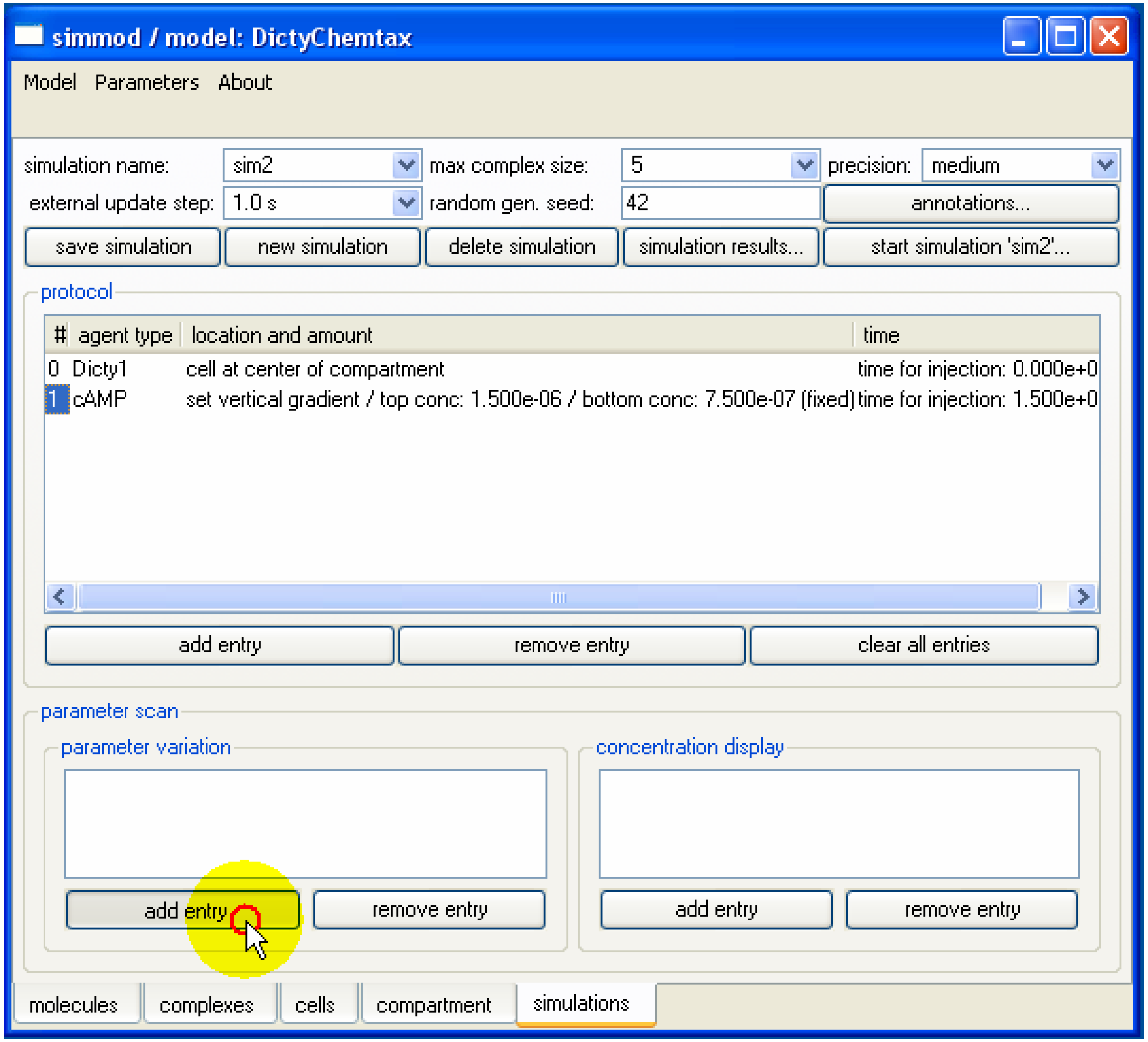

(22) Select the parameter ‘Gbg_PTENph_assoc’ and the lowest and highest value of the variation and the number of steps in between.

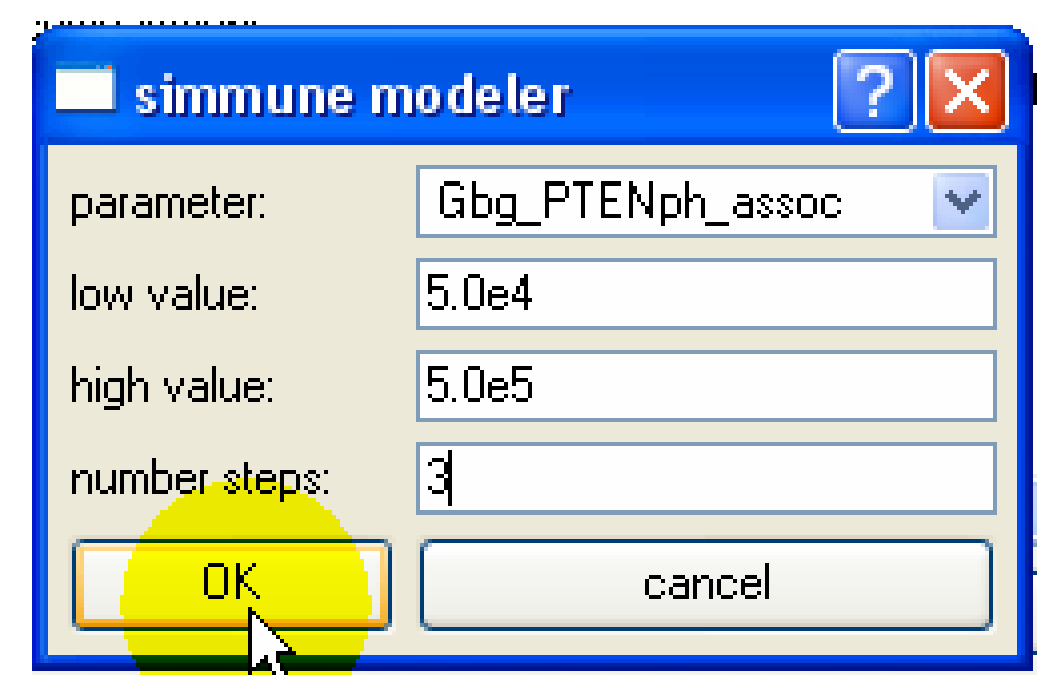

(23) Analogously for the variation of the number of Csk molecules in the cell.

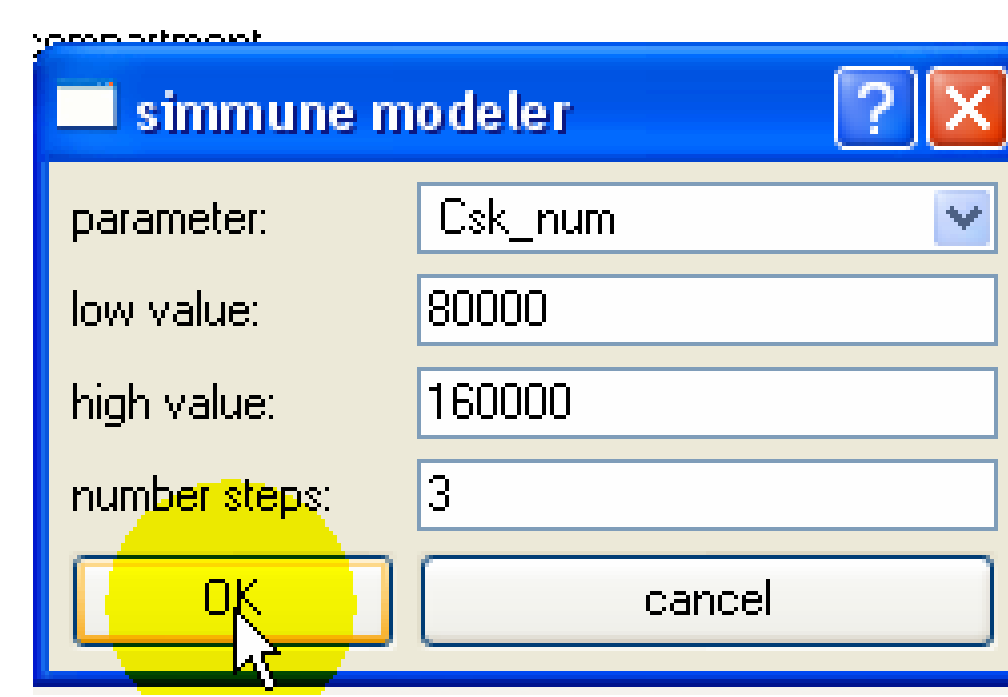

(24) Click on 'add entry' in the concentration display list.

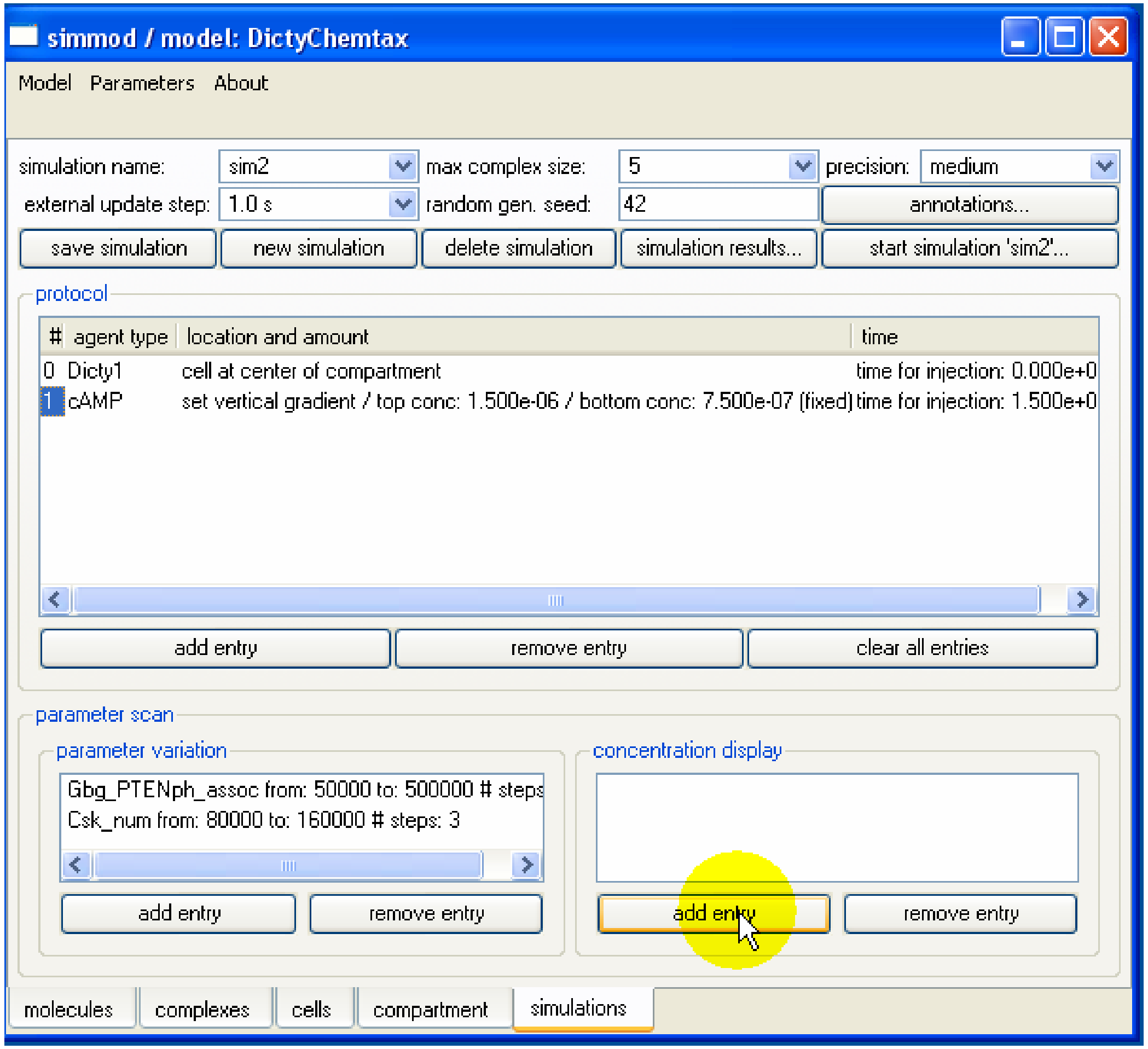

(25) The parameter scan simulation will display the behavior of the selected molecule bundles (see documentation) in the selected locations of the cell.

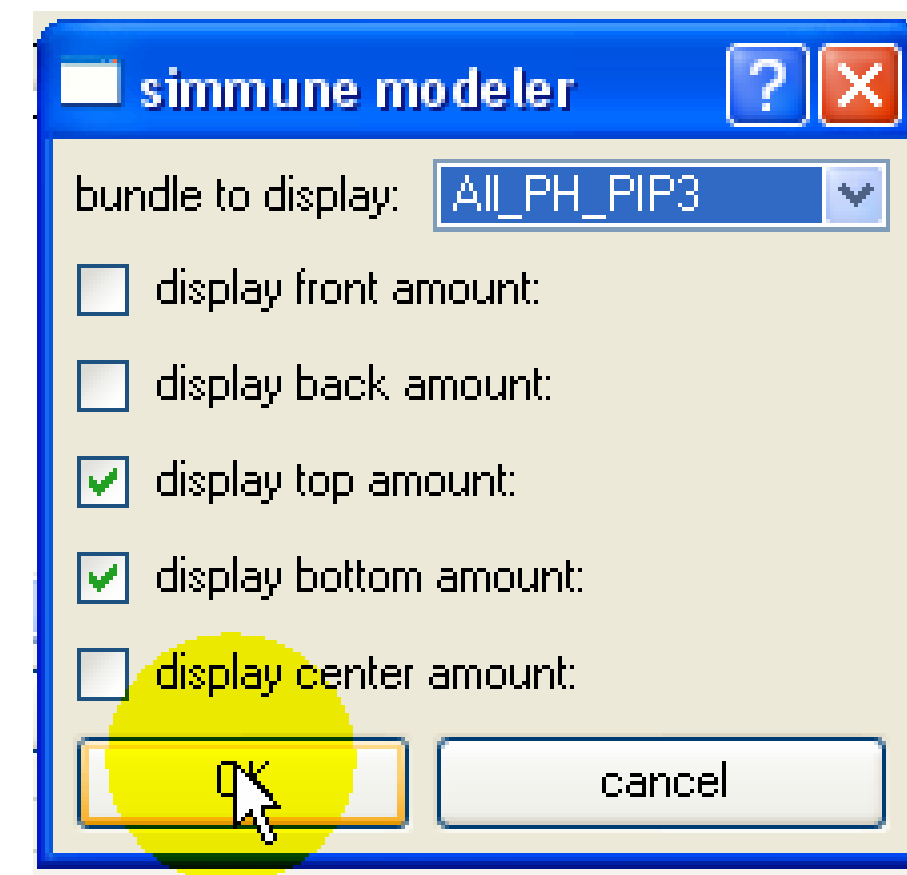

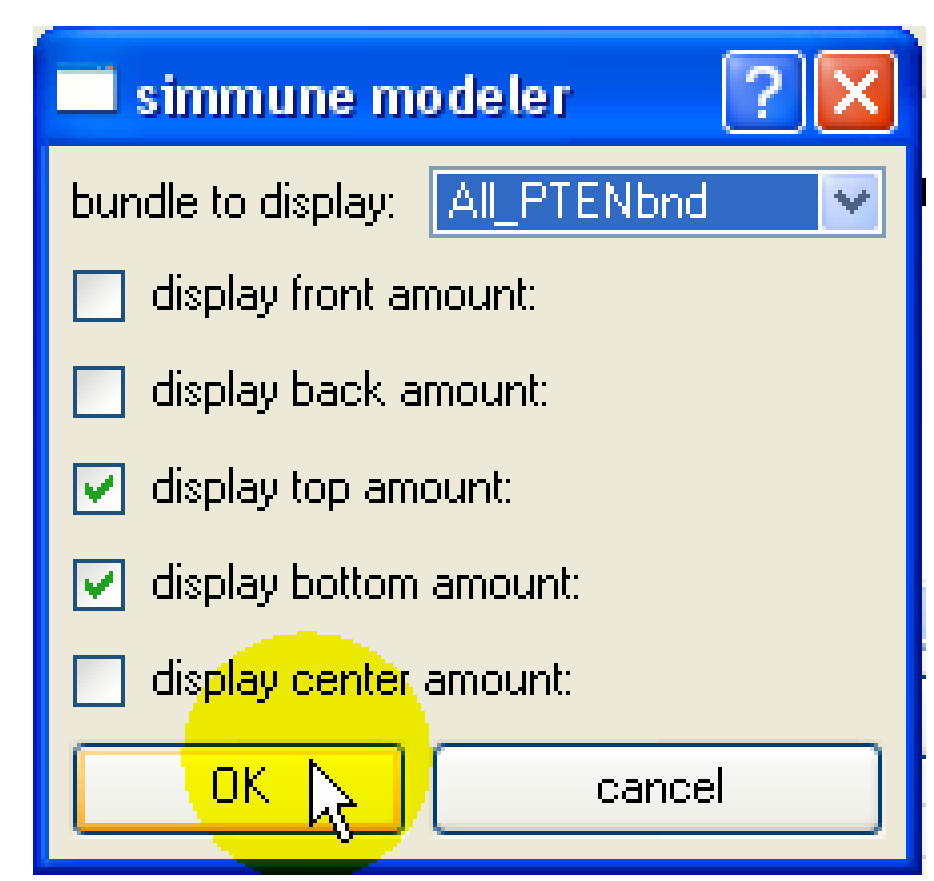

(26) Select 'All_PH_PIP3' and 'All_PTENbnd' as shown here. Important: Only **molecule bundles** can be used for display here!

(27) Select ‘8’ to be the maximum simulated molecule complex size (see documentation for details about the generation of the reaction network)

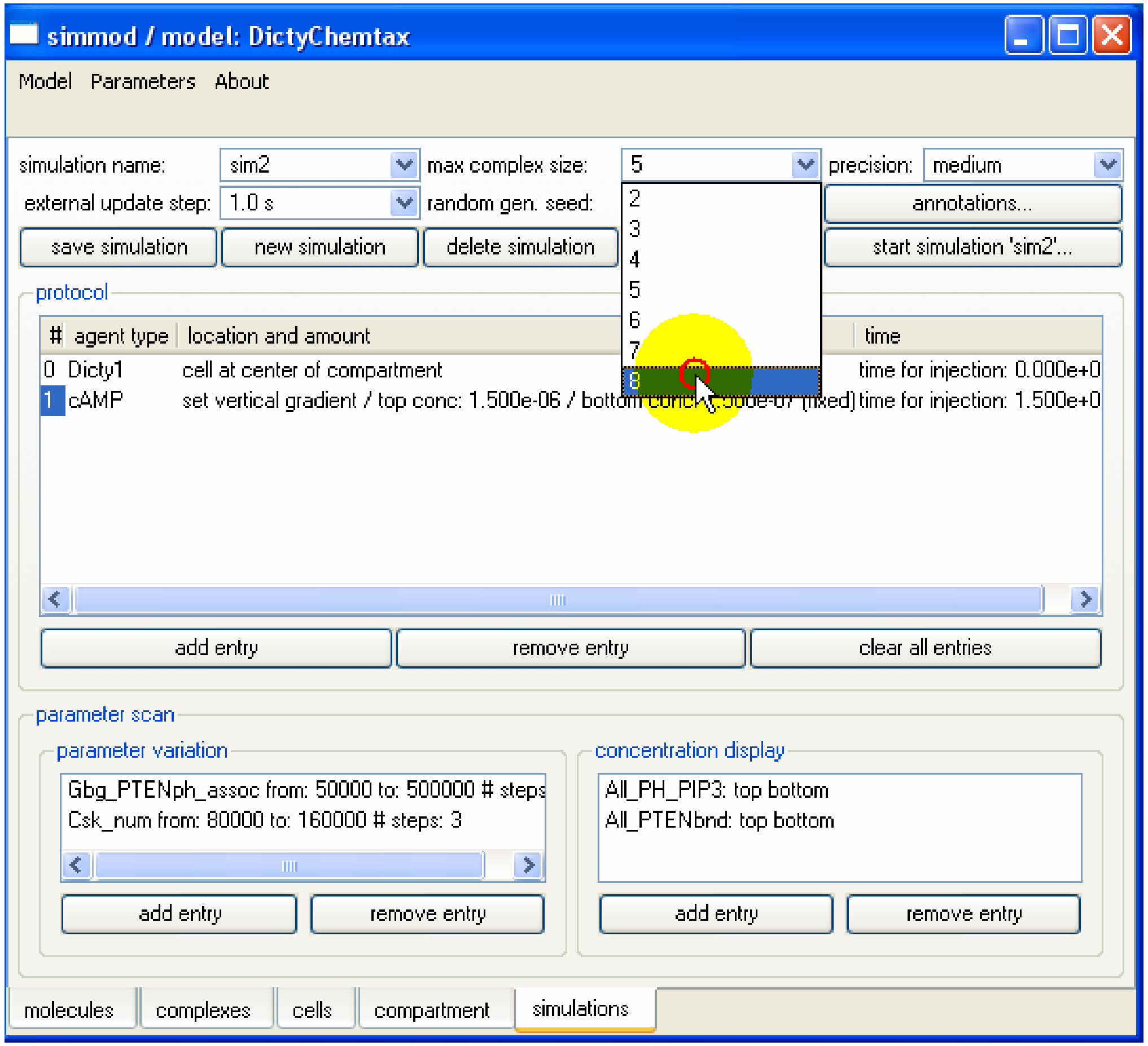

(28) Don’t forget to save the simulation properties before starting the simulation.

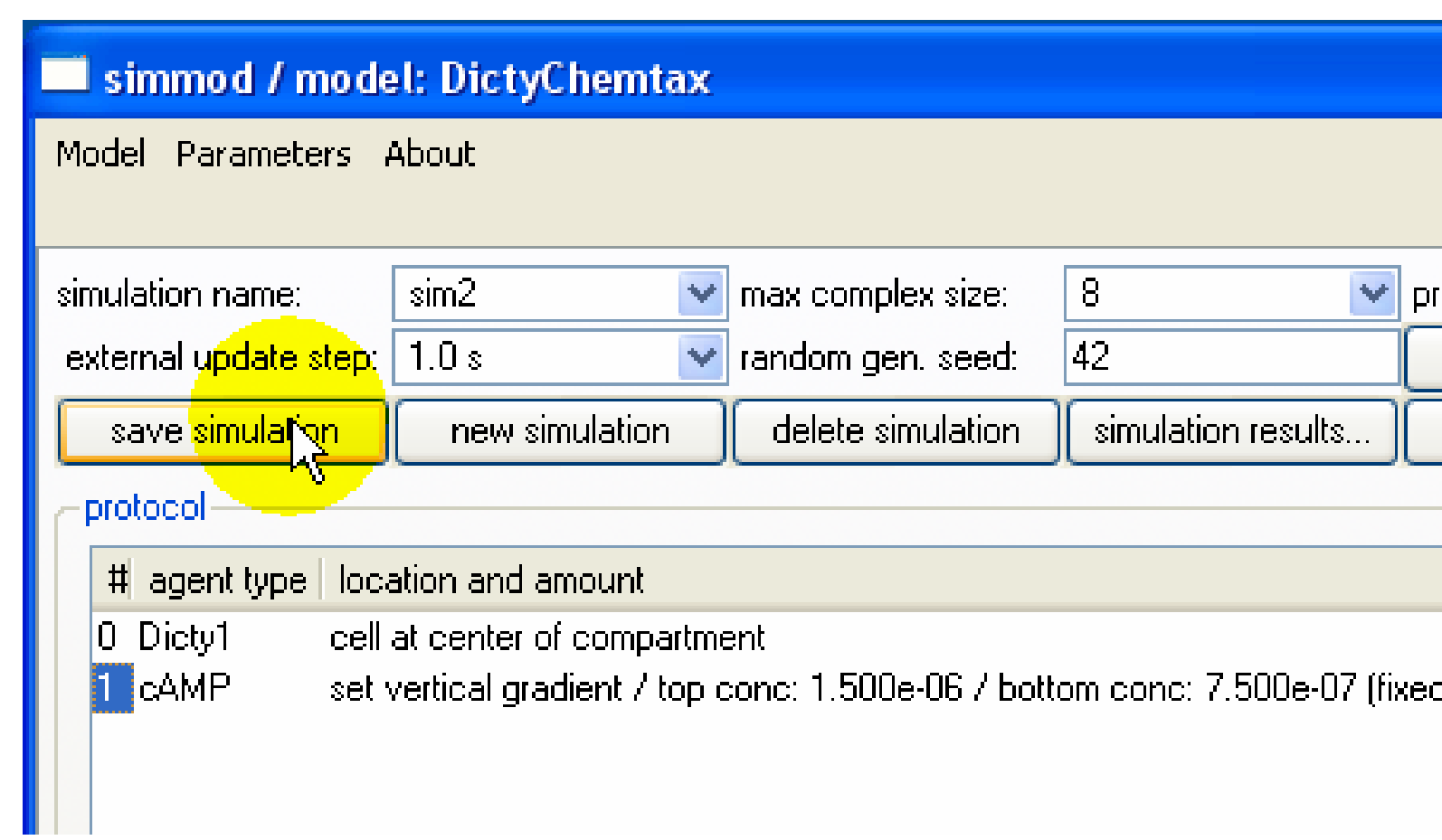

(27) The parameter scan window shows a grid with parameter combinations. Set the duration of each single parameter combination simulation run and start the scanning.

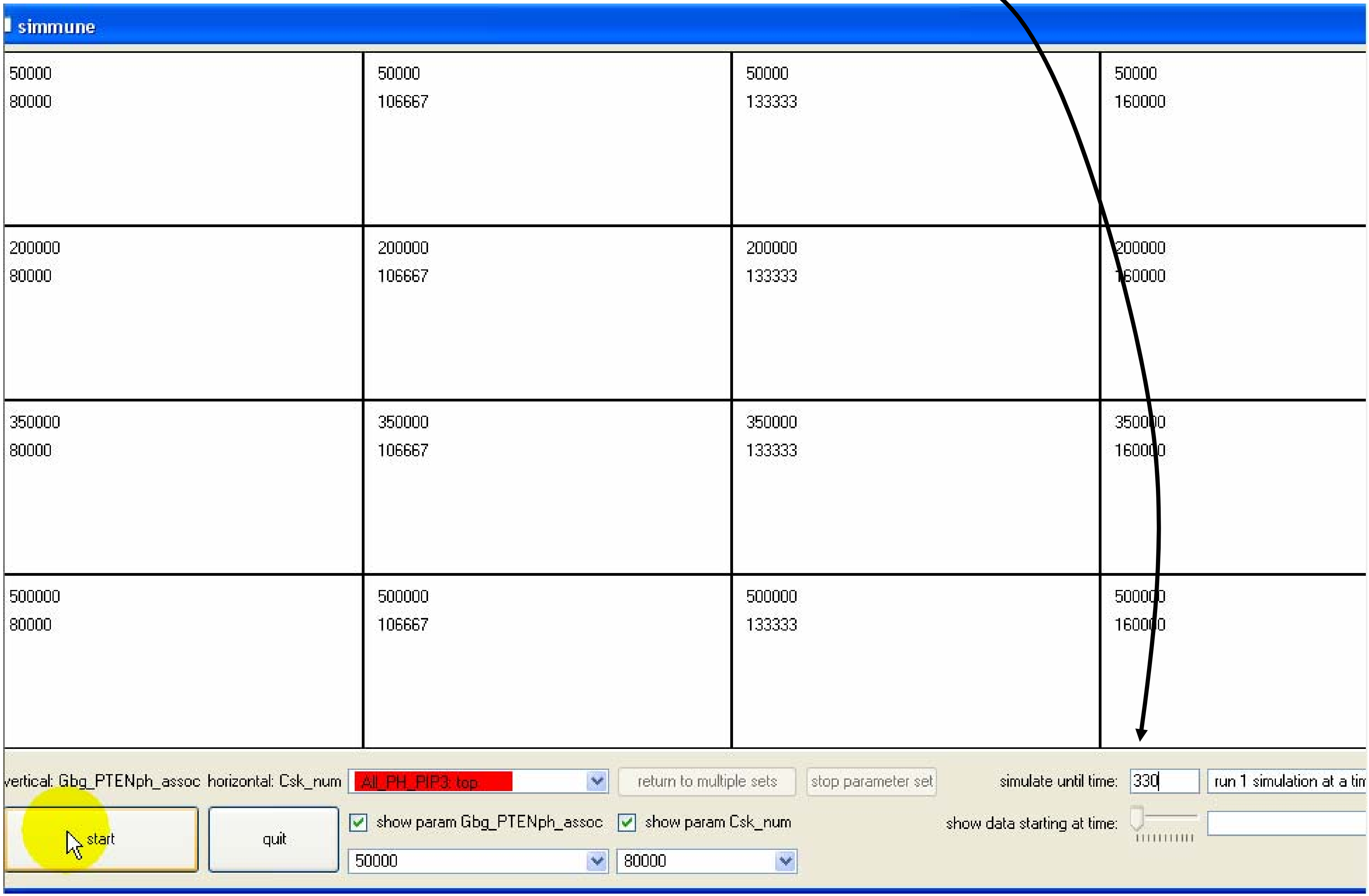

(28) Double-clicking a single-run rectangle switches to a more detailed display of the behavior of that parameter combination.

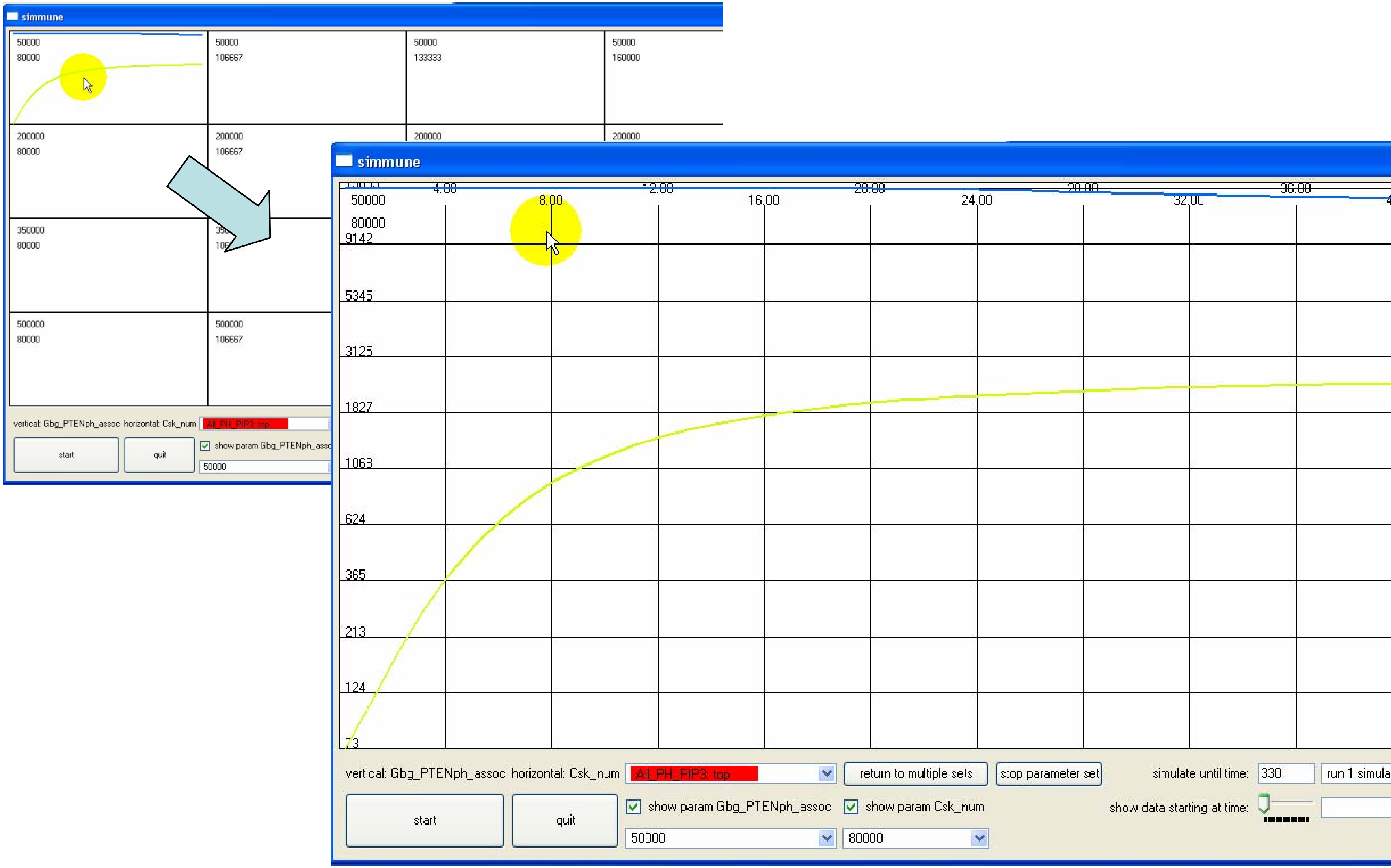

(30) You can change the start time for the display.

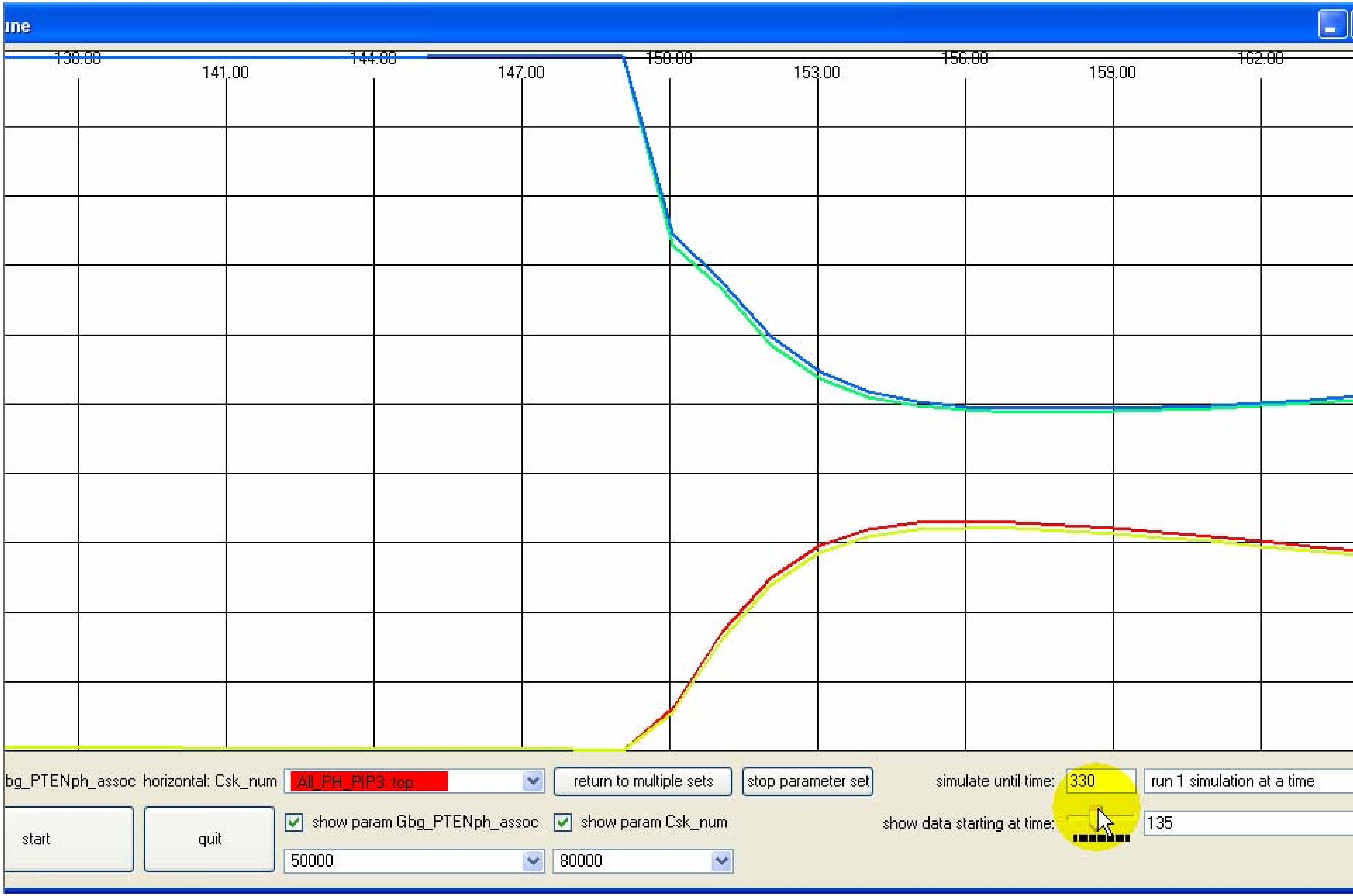

(32) The pop-up menu show the color codes.
Here, the separation between the behaviors of PIP3 and membrane-bound PTEN in the upper and lower regions of the cell can be seen as it unfolds.

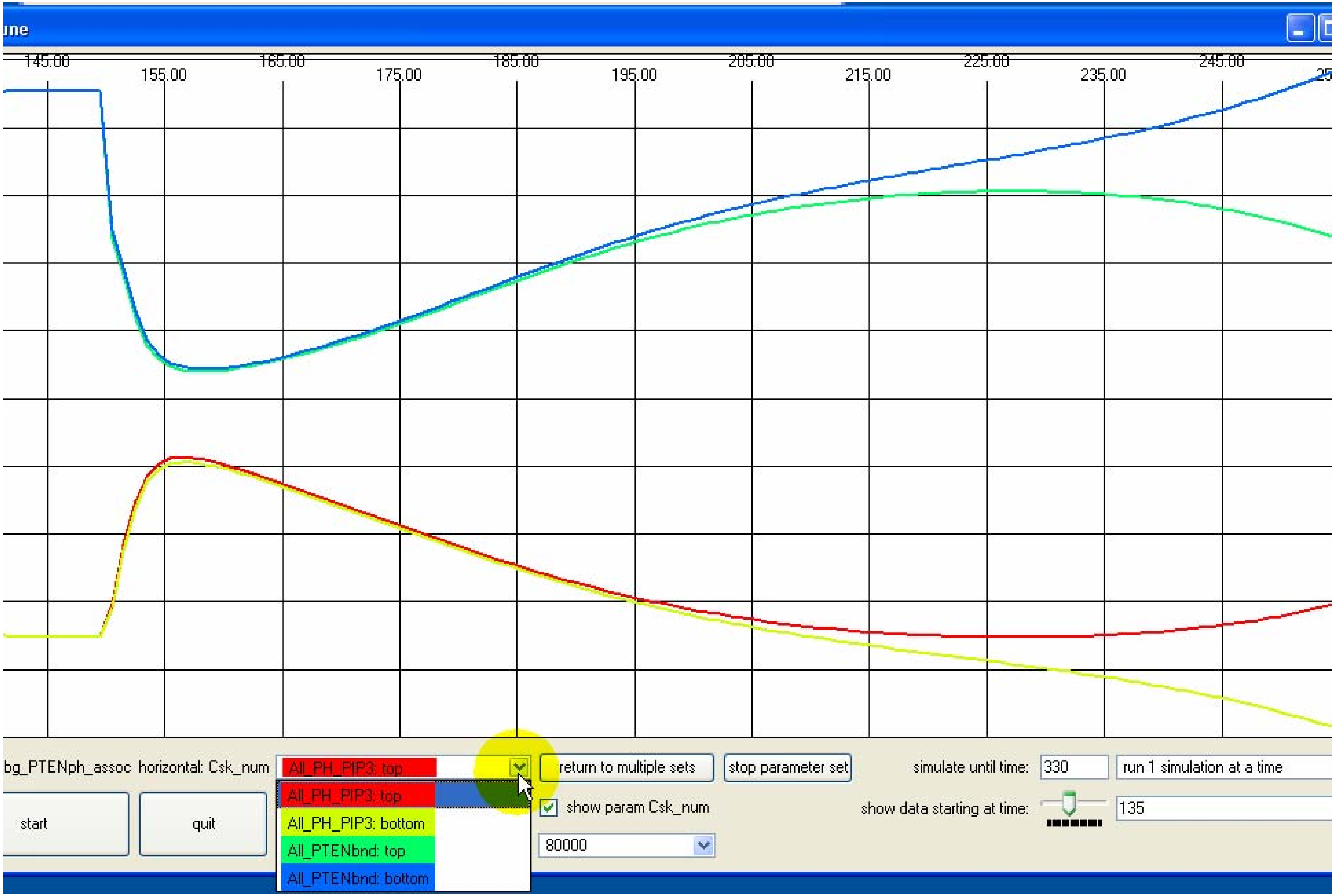

(34) Switch back to the parameter grid view to see simulations of other parameter combinations being run by Simmune.

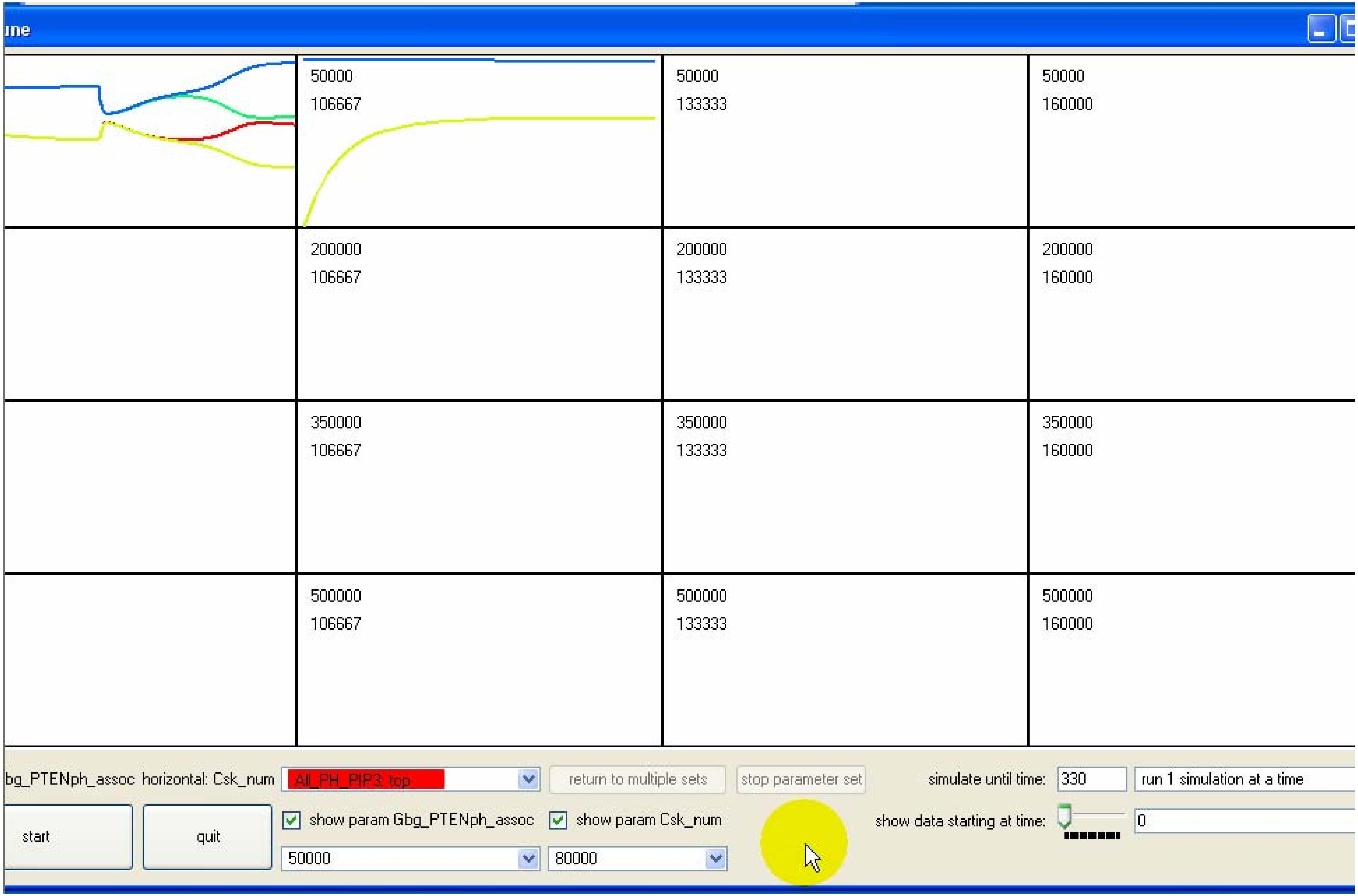

Supplement: Supplementary file 13 [file pcbi.0020082.sd013.pdf]
