# Supplementary material for: Key Role of Local Regulation in Chemosensing Revealed by a New Molecular Interaction-Based Modeling Method
Source: PLoS Comput Biol. 2006 Jul 21;2(7):e82. doi: 10.1371/journal.pcbi.0020082 (PMC1513273; doi:10.1371/journal.pcbi.0020082)
Supplement: Supplementary file 14 [file pcbi.0020082.sd014.pdf]

Simmune tutorial 7

Creating and simulating a simple chemosensing model

In this tutorial we will create a cell type that will translate extracellularly applied concentration gradients of a ligand that binds to cell surface receptors into intracellular gradients of a signaling molecule. The reaction network we will use in this simple model incorporates a feedback loop that will achieve a certain intracellular polarization but falls short of really amplifying the extracellular gradient.

This tutorial is meant to

(1) provide an introduction to simmune using a transmembrane signaling process,

(2) provide some first impression of signaling mechanisms capable of translating extracellular gradients into intracellular biochemical polarization.

(1) Start 'simmod' and create a new model:

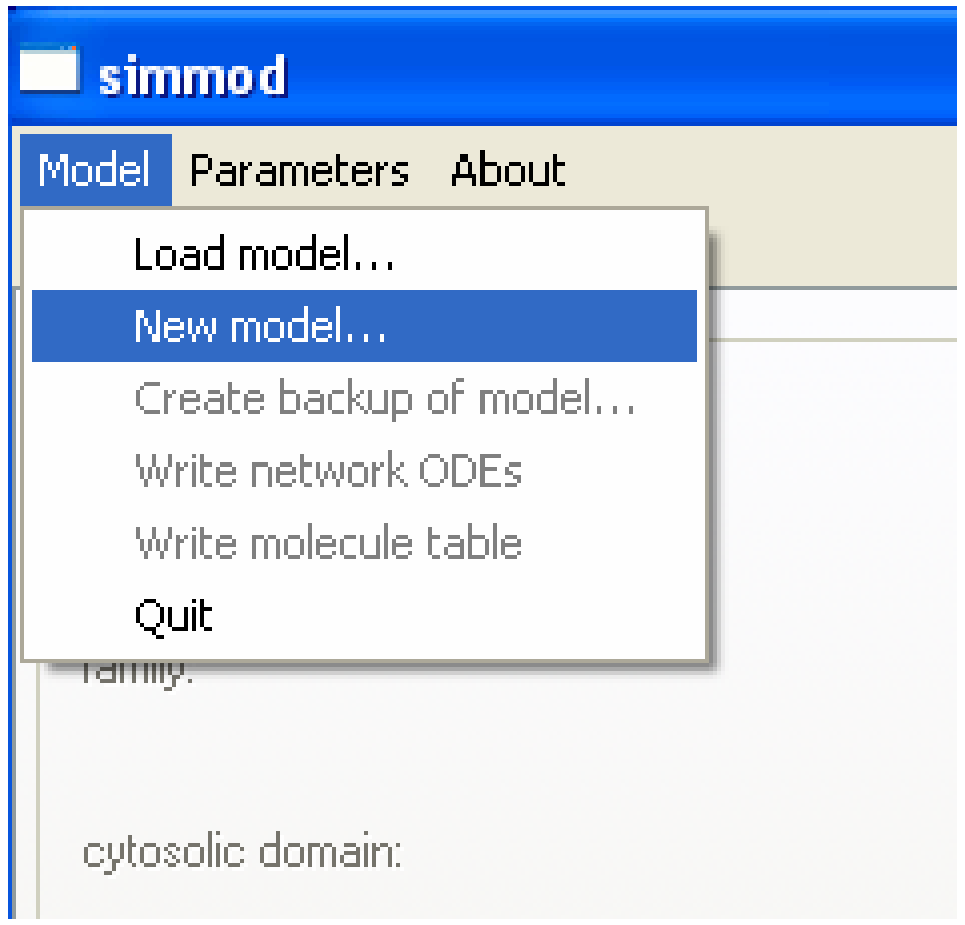

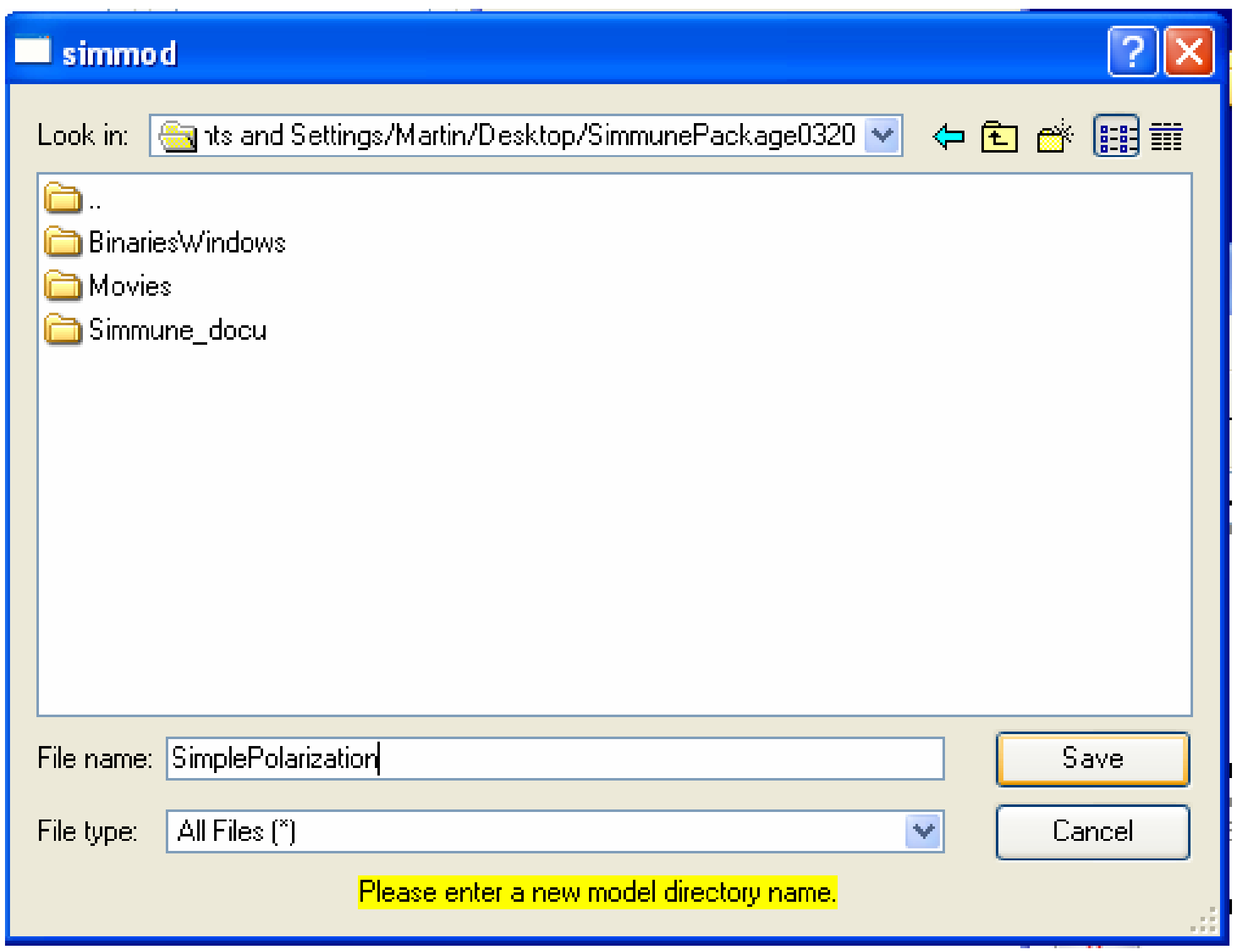

Call it, for example, 'SimplePolarization'.

(2) First, we create a receptor for the extracellular ligand we will use to stimulate simulated cells with. Click on ‘new molecule’.

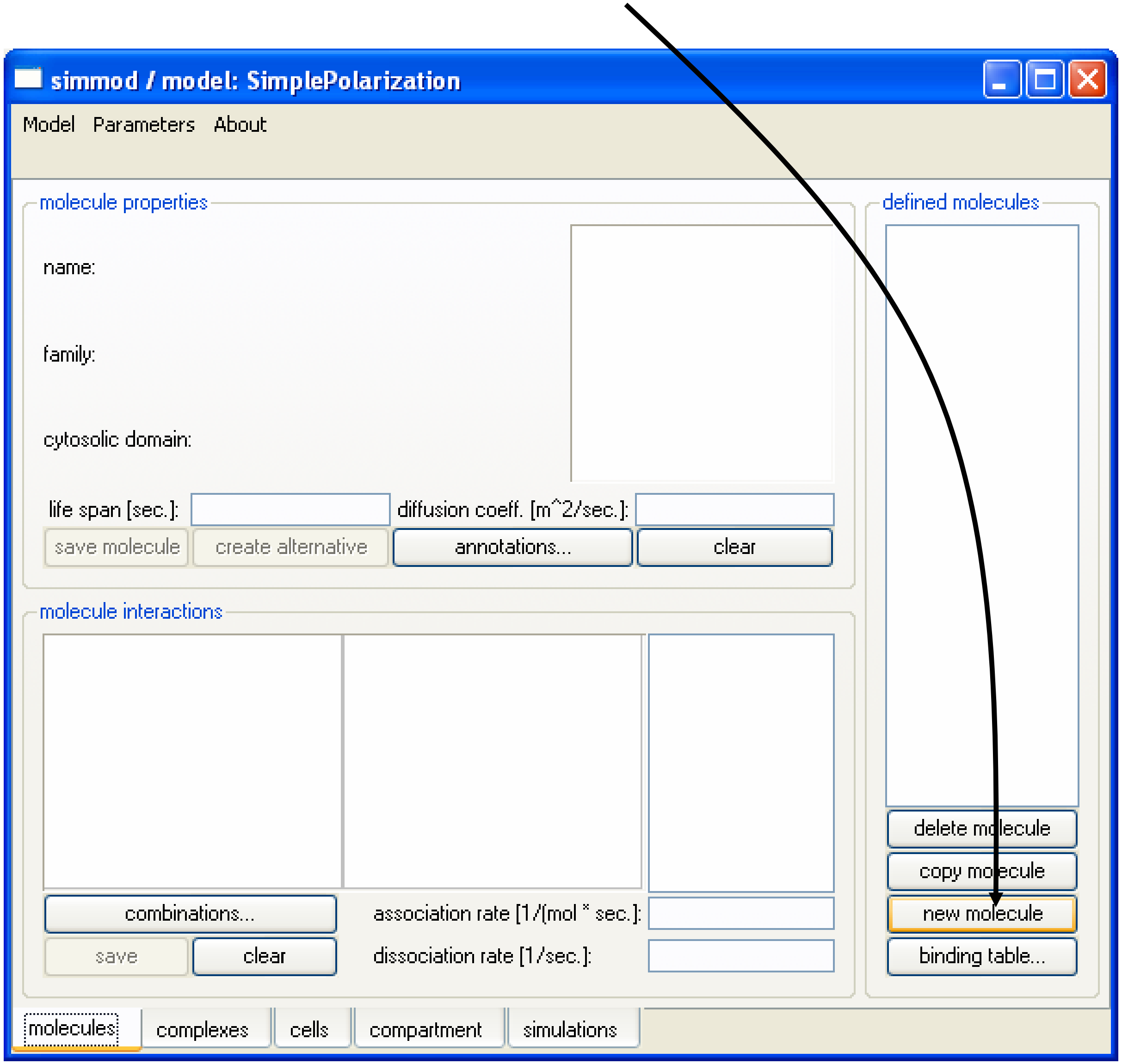

(3) In the window that appears define the molecule to be a receptor, more precisely: to be the extracellular part of a transmembrane receptor.

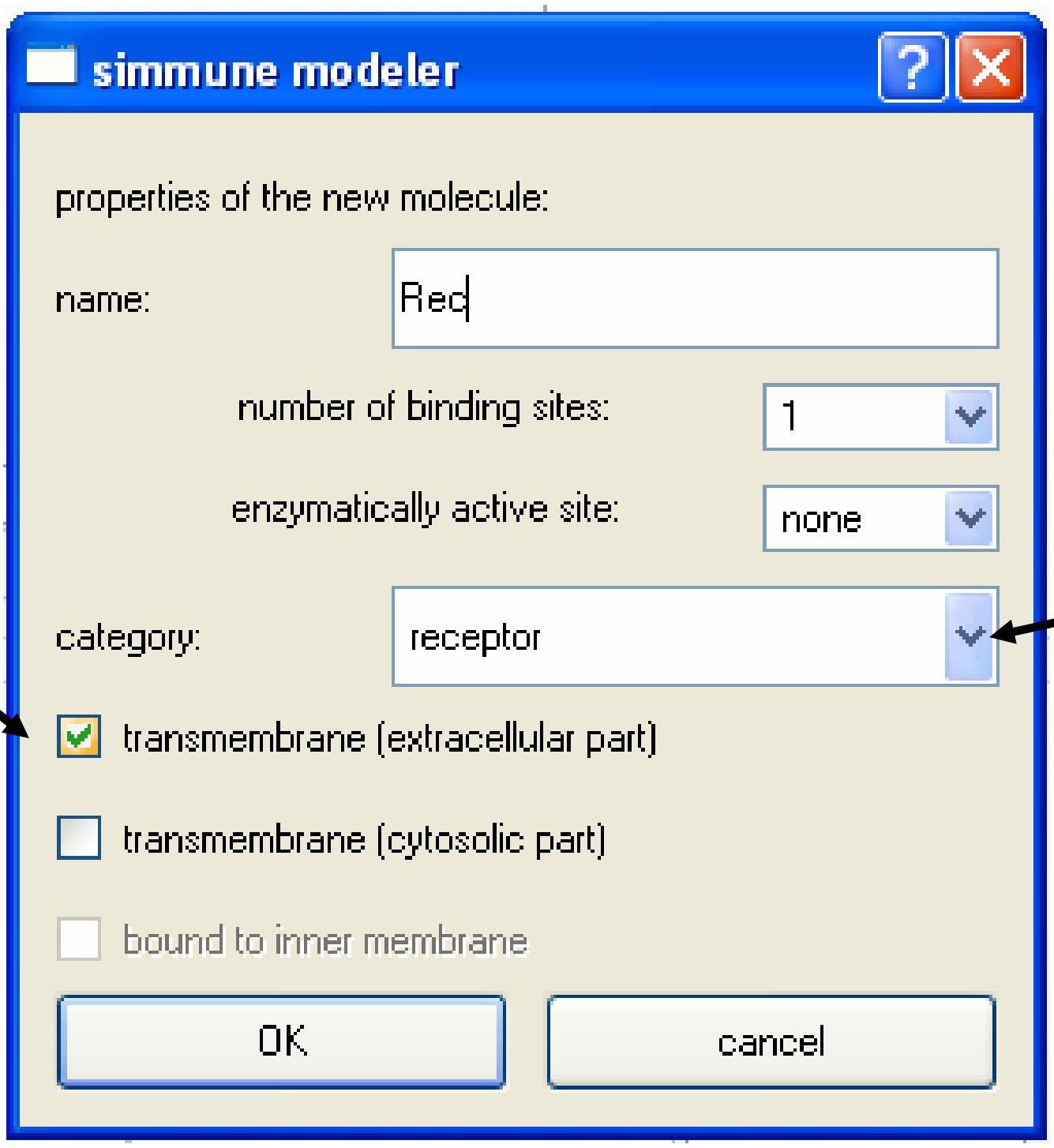

Click OK.

(**4**) Now, create the cytosolic part of a transmembrane receptor.
Make sure you define its (only) binding site to be enzymatically active.

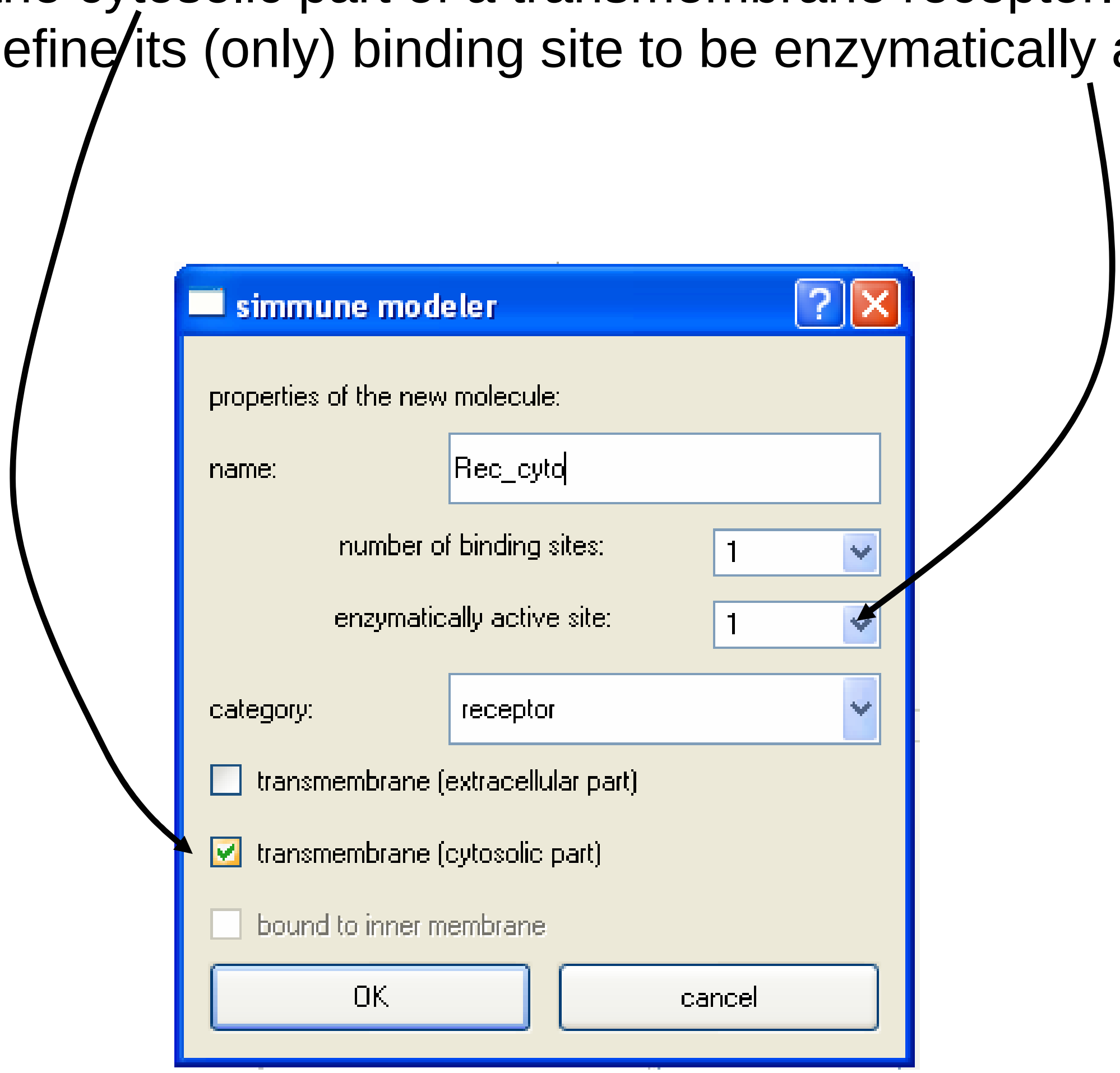

(5) Double-click the ‘Rec’ molecule in the list of defined molecules. It will appear in the ‘molecule properties’ field. (You can also drag the molecule into the white area of the ‘molecule properties’ field.)

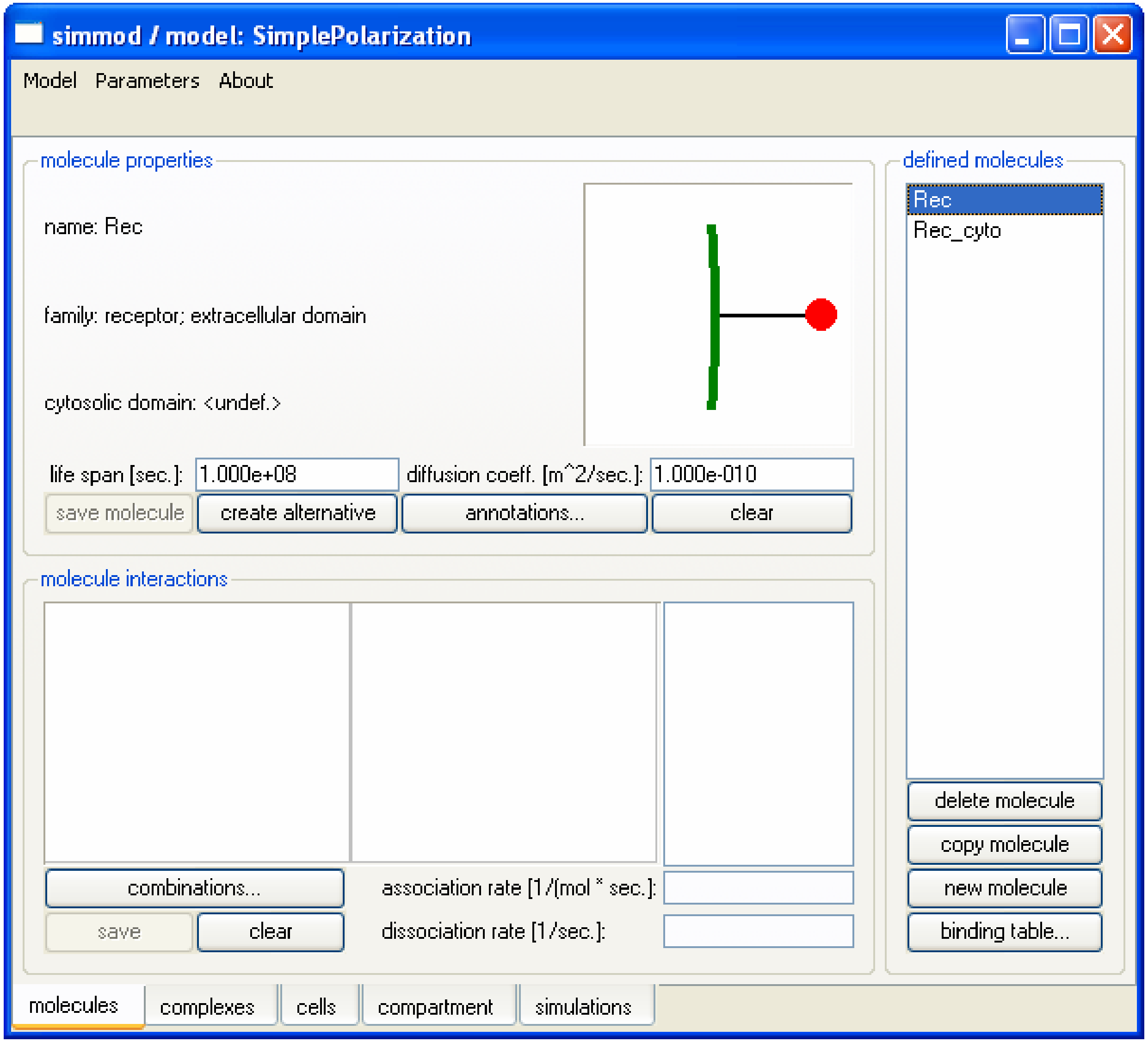

(6) Now drag the cytosolic part ‘Rec_cyto’ onto the extracellular domain Rec.
Click OK in the warning notice simmod below.

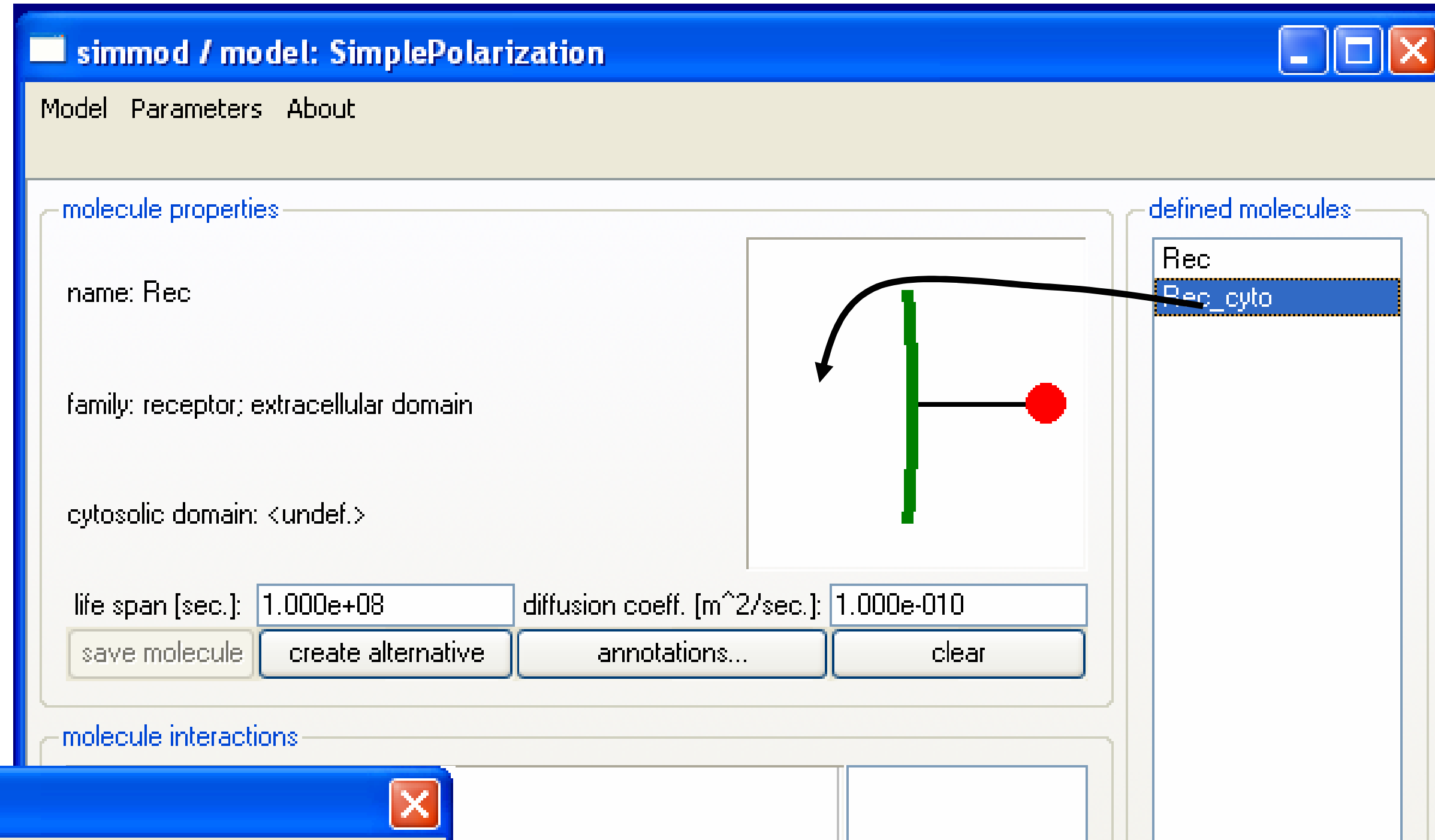

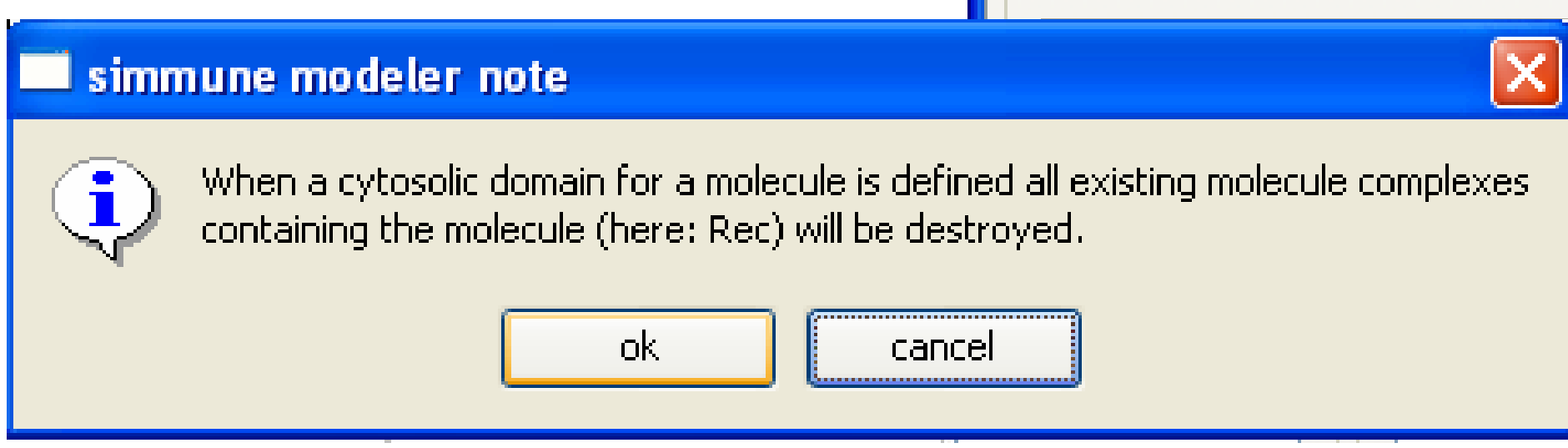

(7) The complete receptor now should look like this. Define its diffusion coefficient to be 1.0e-15 and the save the receptor.

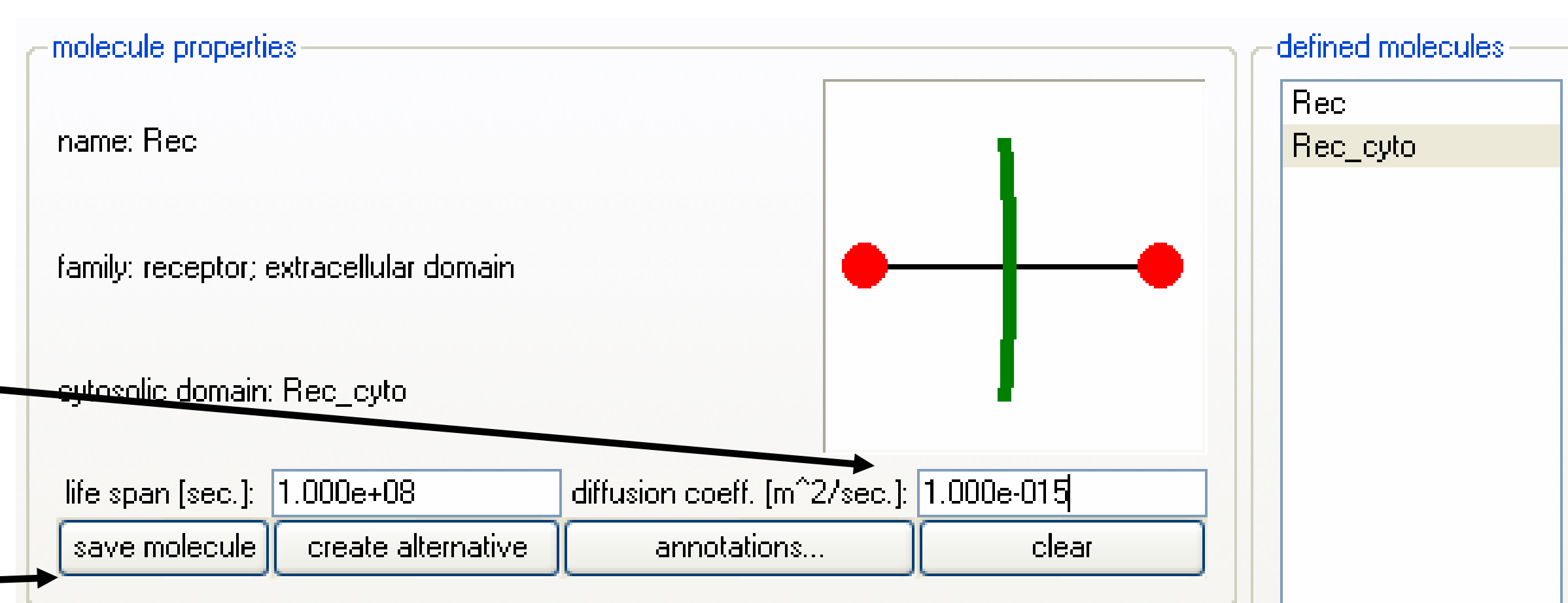

(8) Now, double-click Rec_cyto in the list of defined molecules. It should appear in the ‘molecule properties’ field. Click on ‘create alternative’.

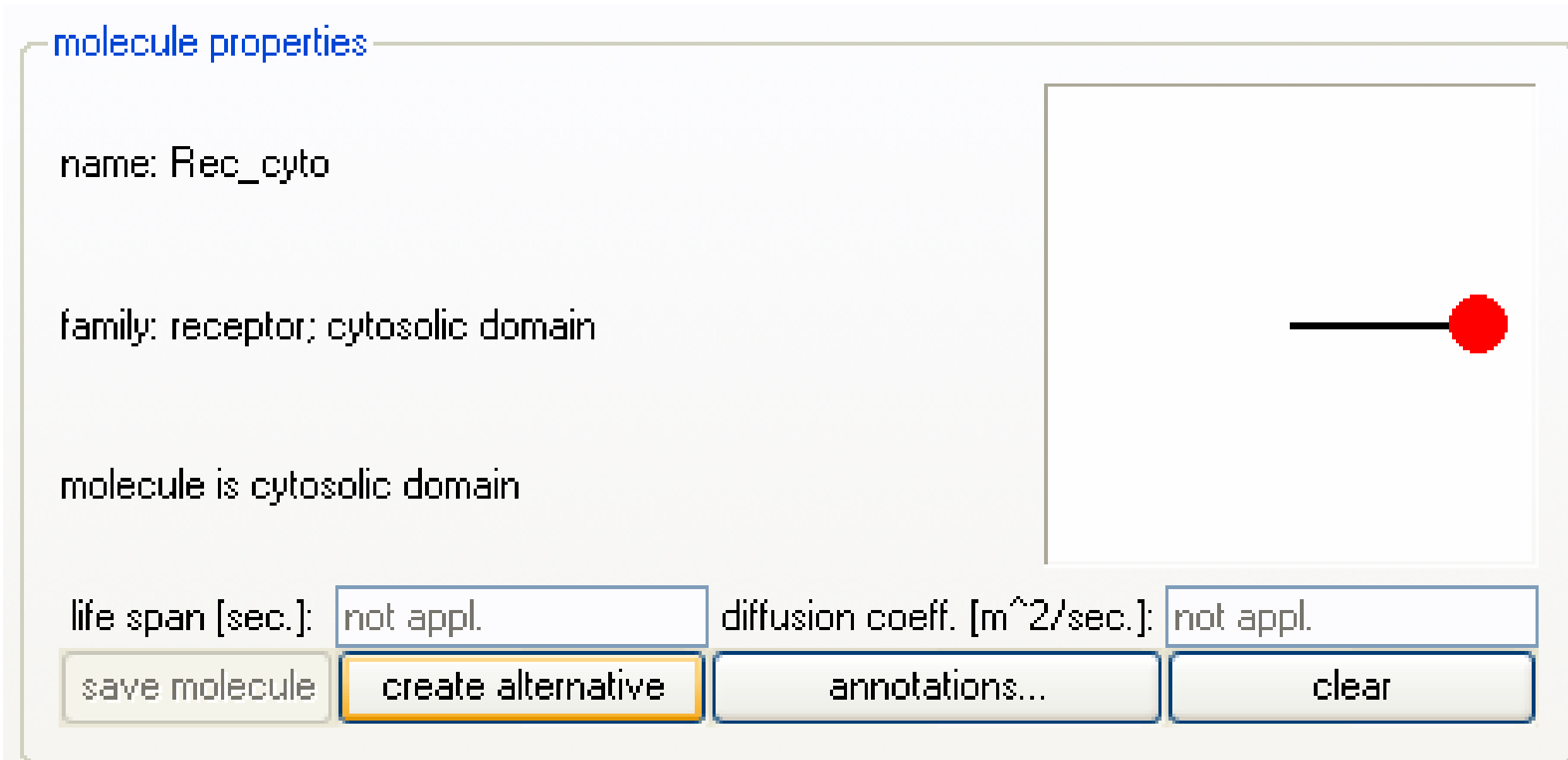

(**9**) Define the name of the alternative state of Rec_cyto to be Rec_cyto_act.

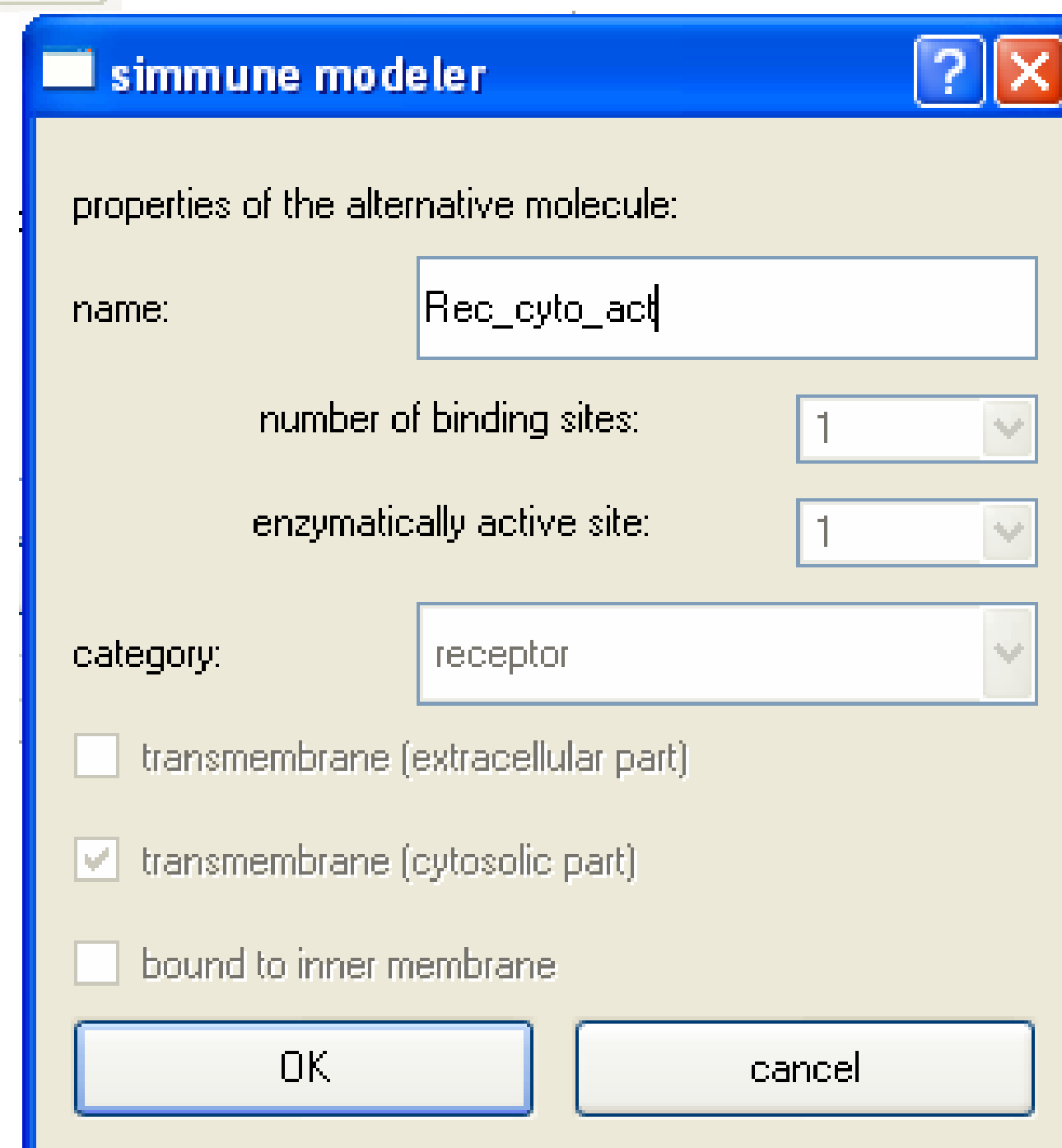

(10) Create the molecule we will use as extracellular ligand for the receptor:

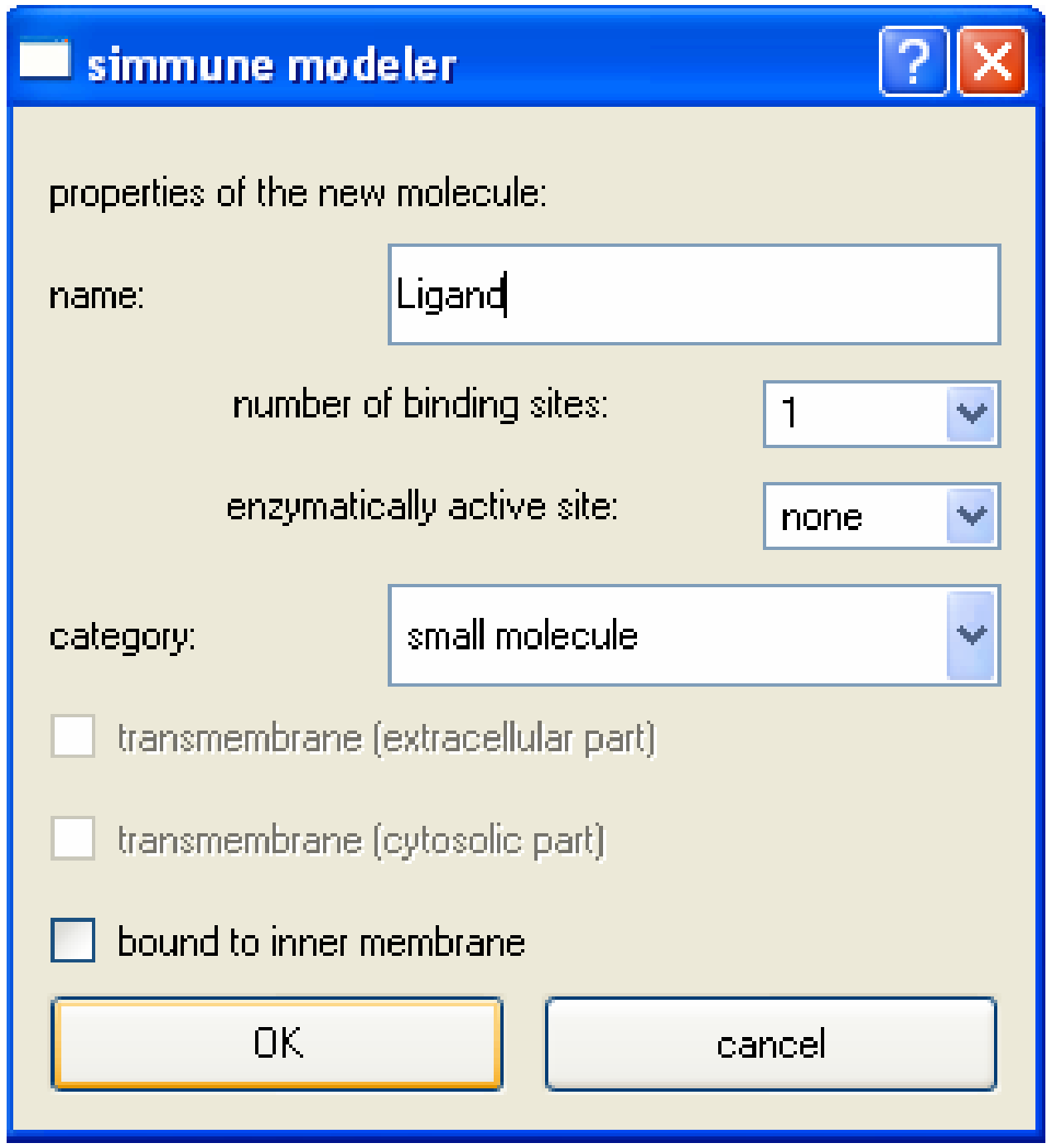

(11) Drag the Rec molecule into the left area of the 'molecule interactions' field and then (!) the Ligand into the right area.

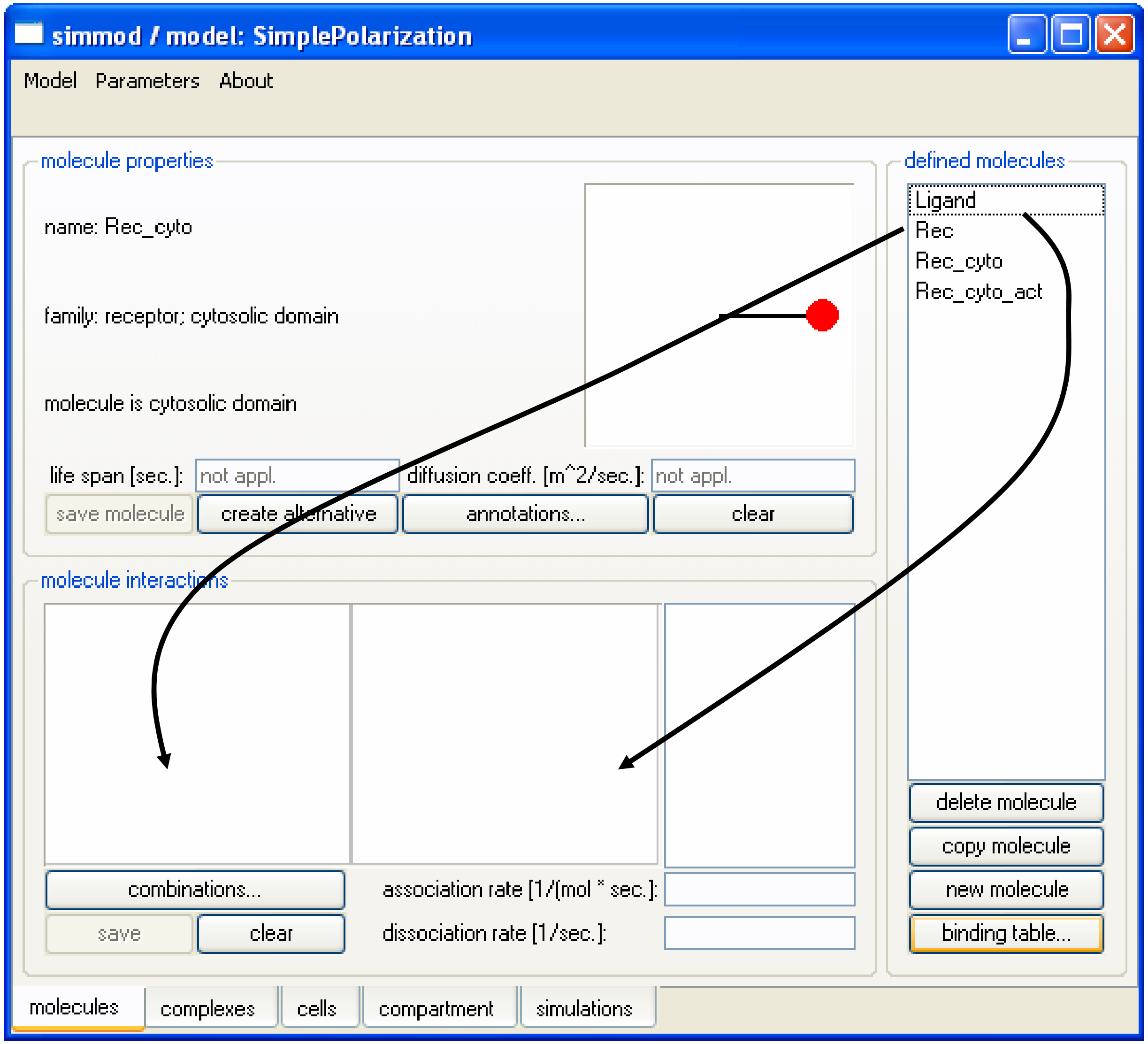

(12) Draw a connection (starting on the left) between the red dots representing the molecular binding sites. Then, click (once) on the small rectangle attached to the dotted line that will appear. The association and dissociation rates of the interaction can now be entered.

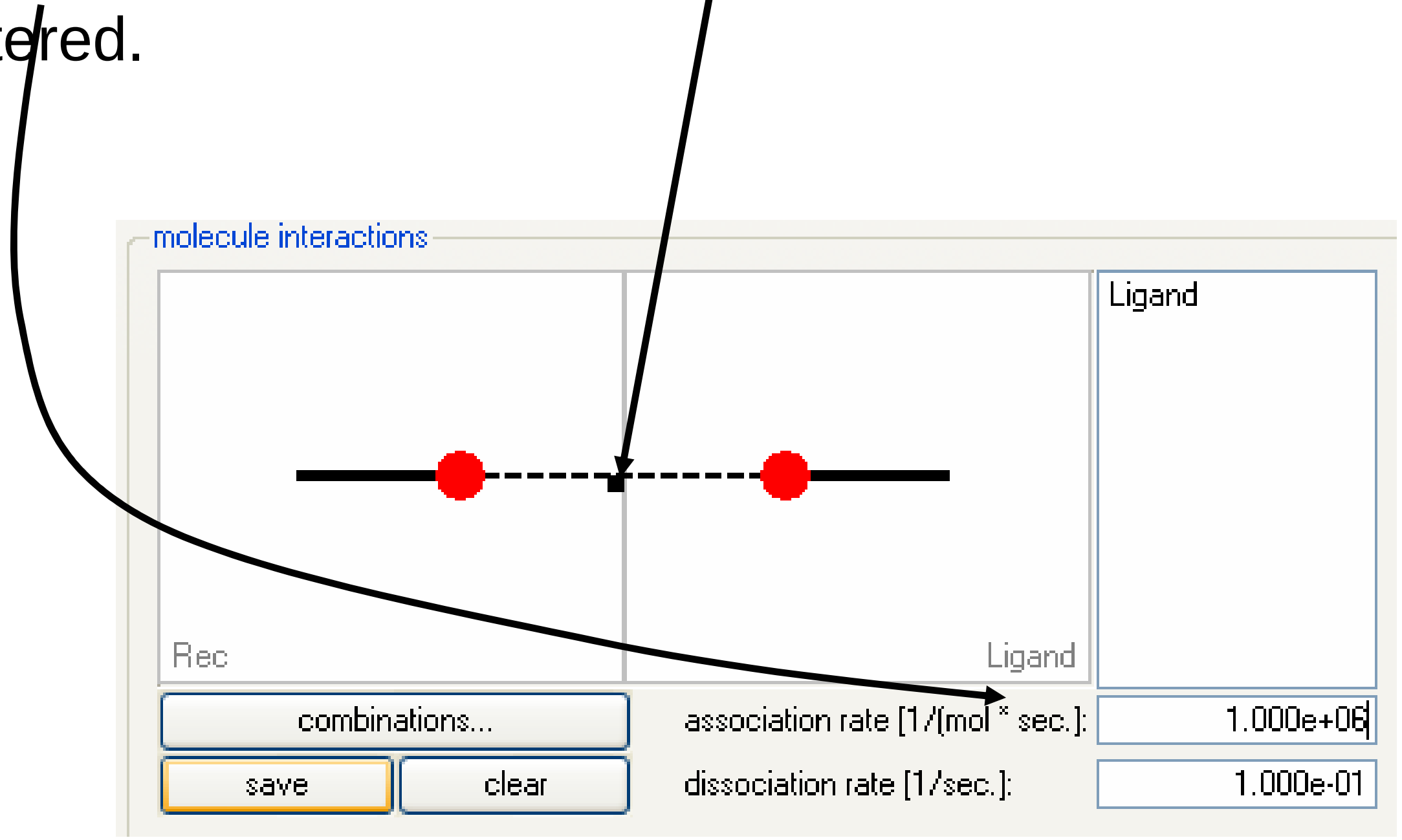

(13) Now, double-click the interaction rectangle.

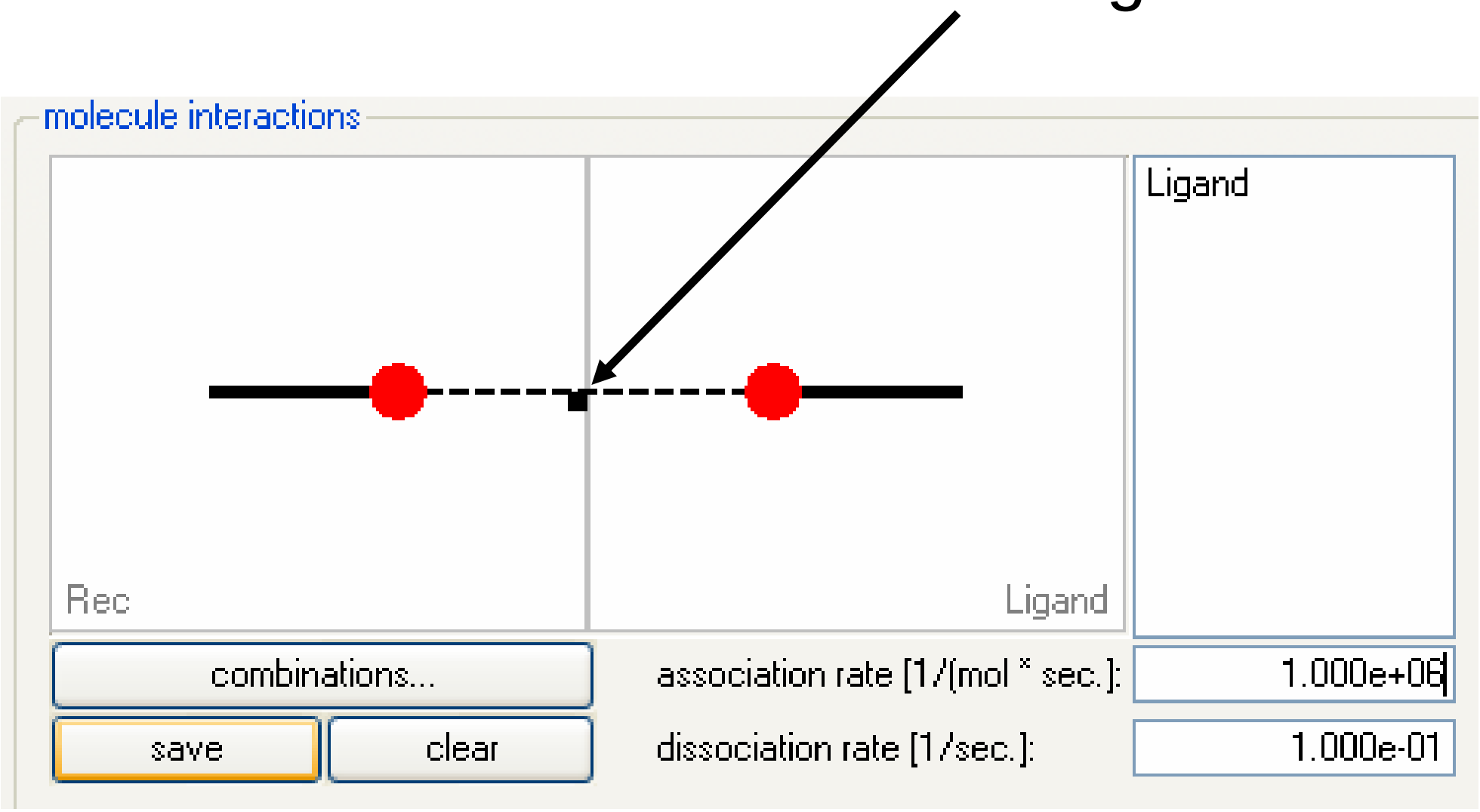

(14) Click on ‘binding properties in the window that appears

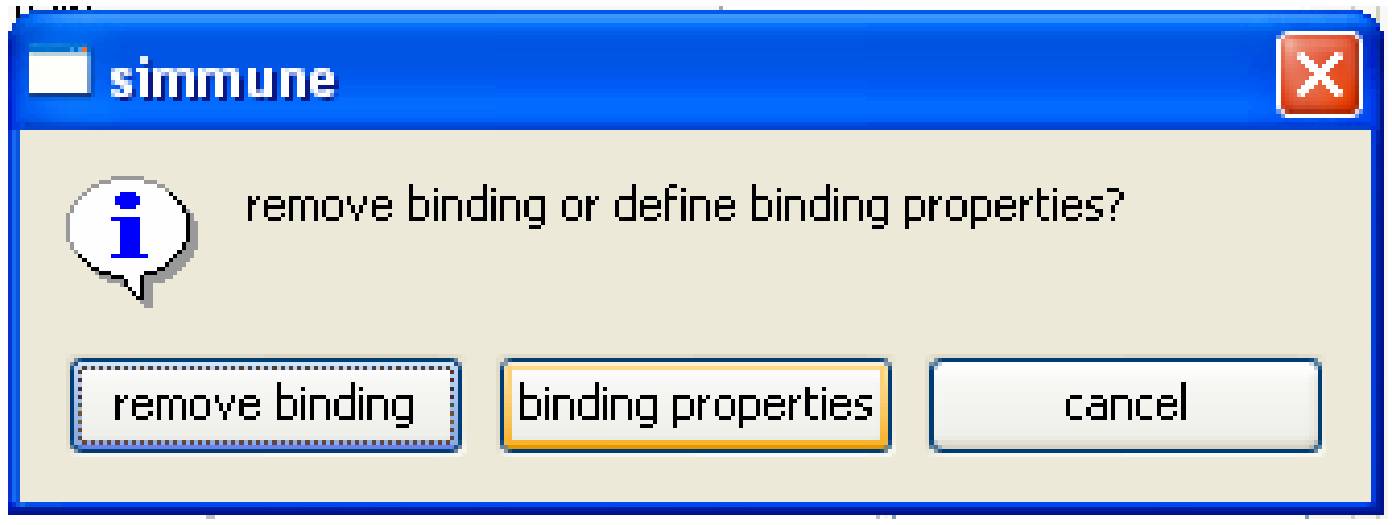

(15) Make the selections as shown below.
The binding of ‘Ligand’ to the receptor extracellular domain (‘Rec’) will now transform the intracellular domain from ‘Rec_cyto’ into ‘Rec_cyto_act’.
By assigning different biochemical properties to Rec_cyto_act than to Rec_cyto we can translate the extracellular ligation signal into an intracellular molecular modification – much in the way real cells do.

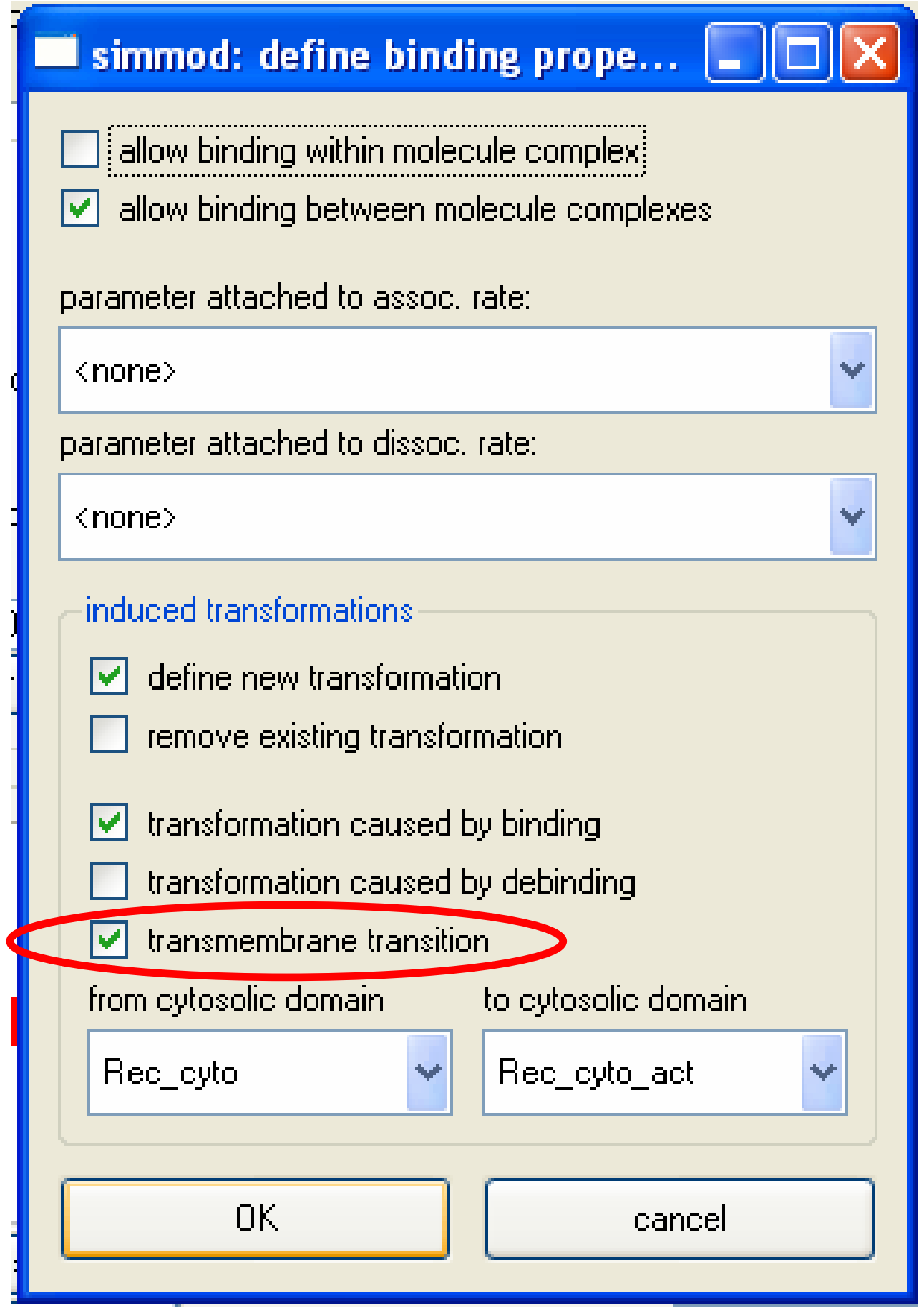

(16) Click on OK and then save the binding properties of Rec.

(**17**) To make the receptor fall back to an (intracellularly) inactive state double-click the interaction rectangle again and set the following transformation. Don't forget to save the changes.

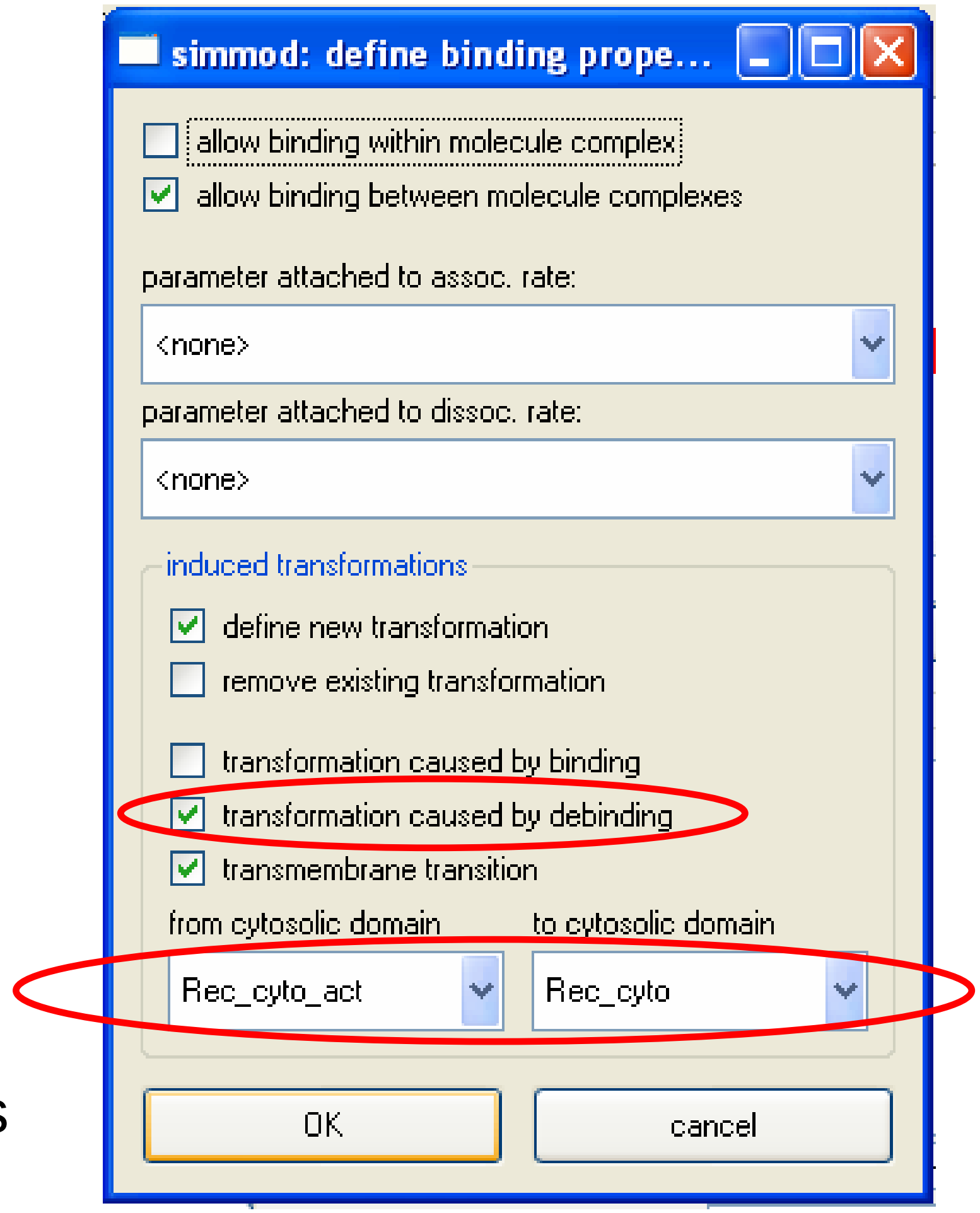

Note: these binding-induced changes will (in this version of simmune) not appear in the molecule tab main window. The binding table will however list them (see (33)).

(18) Now, we create an adaptor molecule that will function as a membrane anchor for another signaling component. We call it ‘MembraneAnchor’ and define it to be bound to the inner cell membrane.

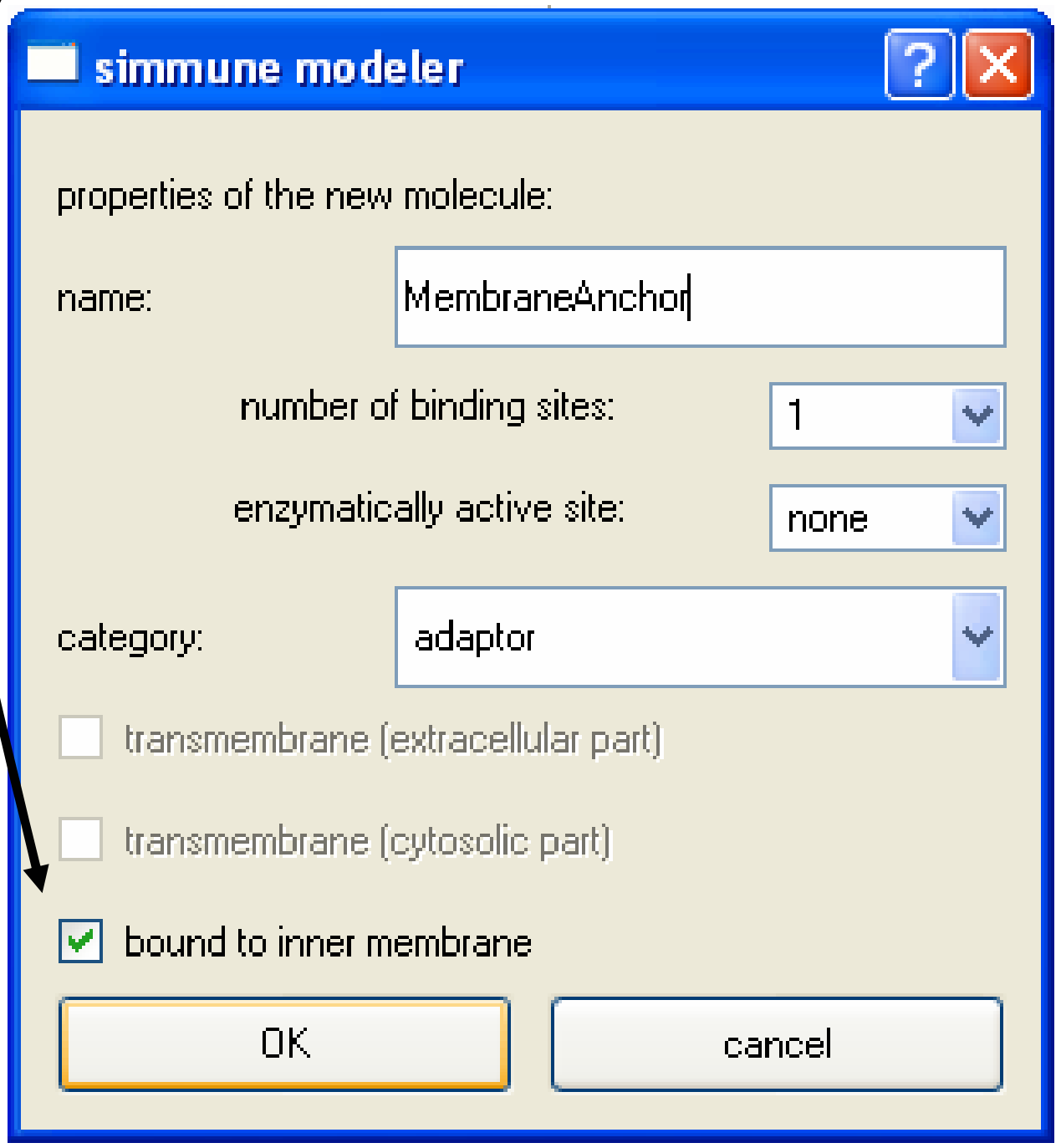

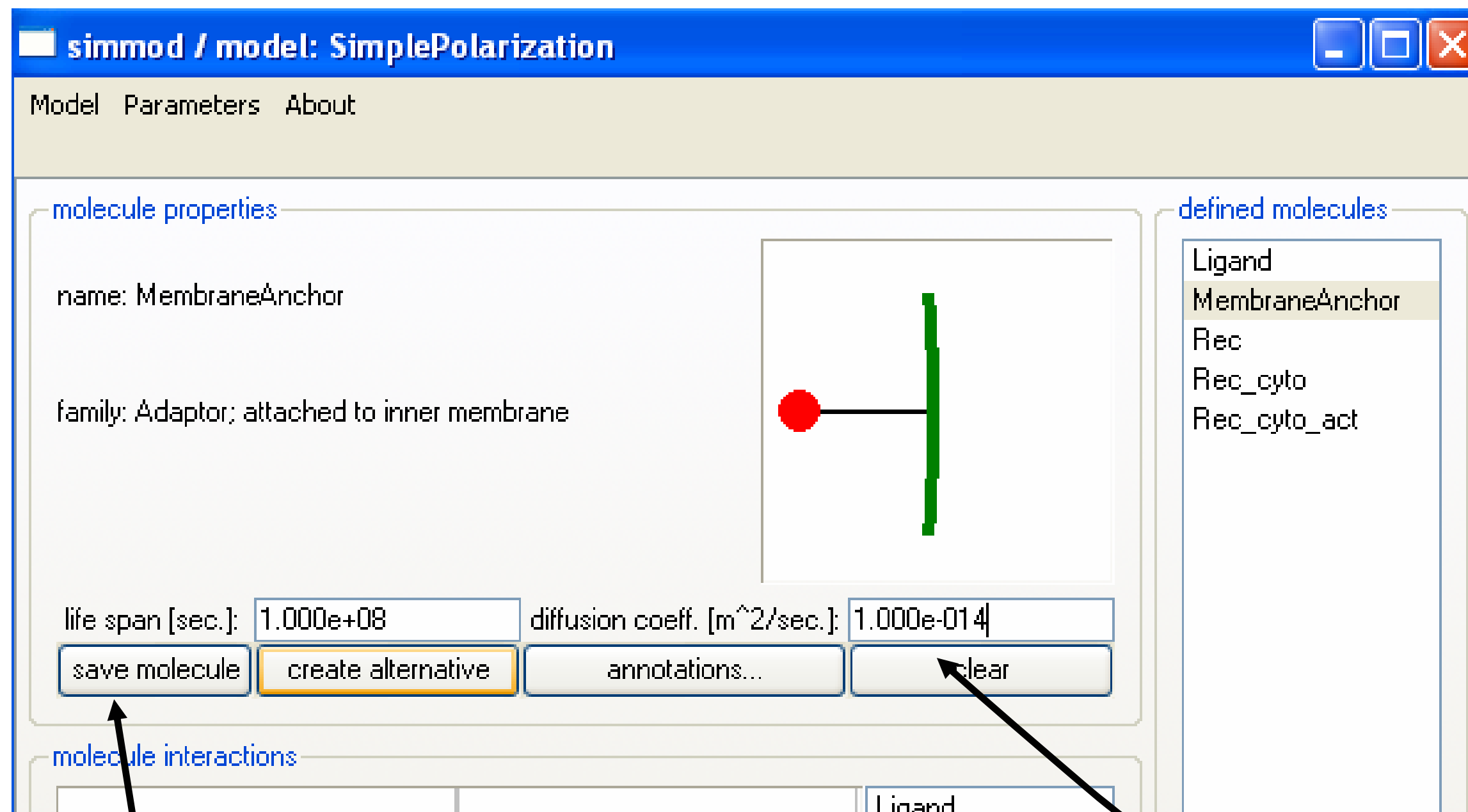

(19) Double-click the new molecule in the list of defined molecules and set its diffusion coefficient to 1.0e-14. Save the changes.

(20) Create an alternative state for the MembraneAnchor. (With the MembraneAnchor being edited click on the 'create alternative' button. Note: this is different from the 'copy' button that creates an independent copy of the behavior of a molecule.) Make sure to set its diffusion coefficient to 1.0e-14 as well.

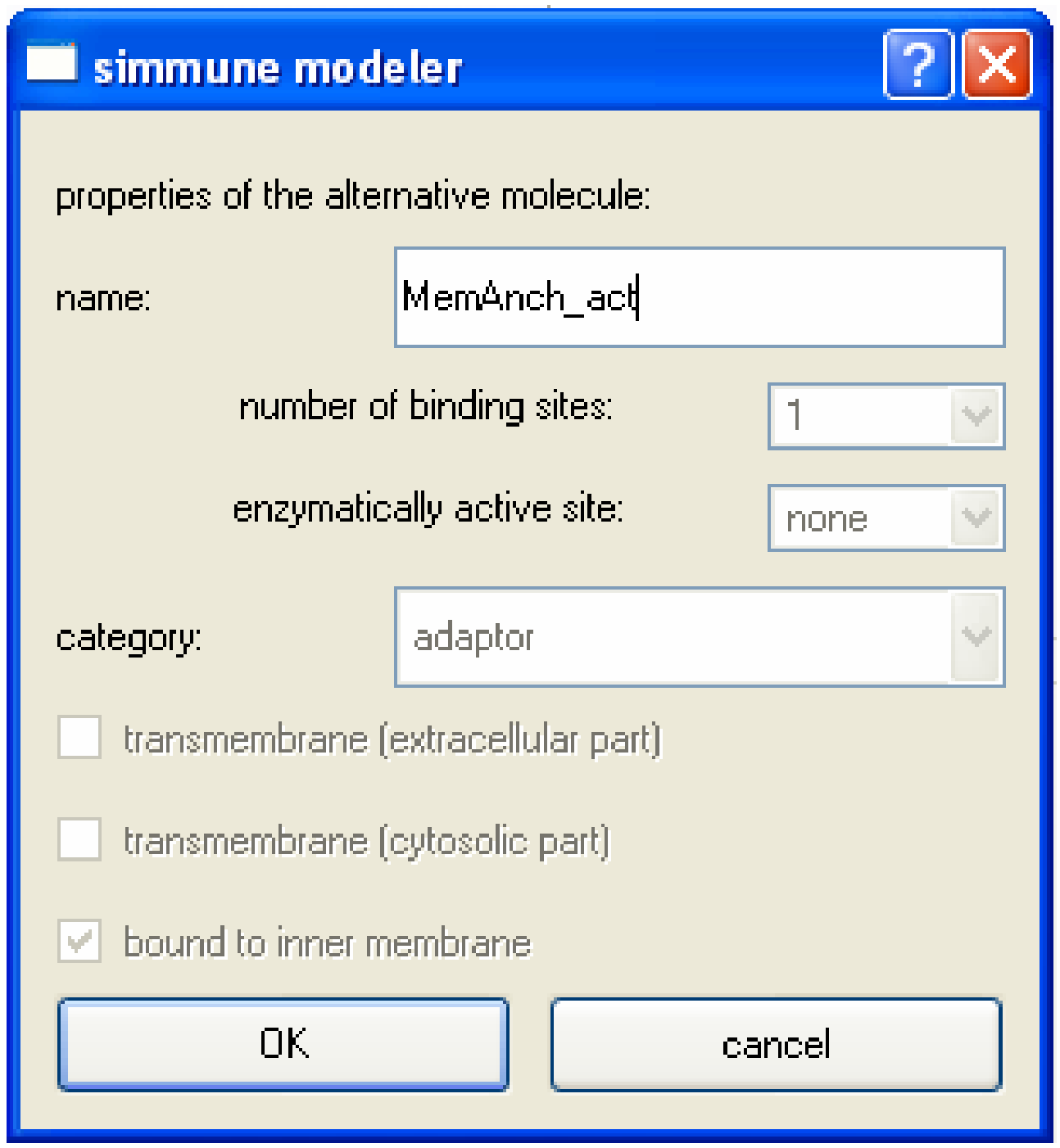

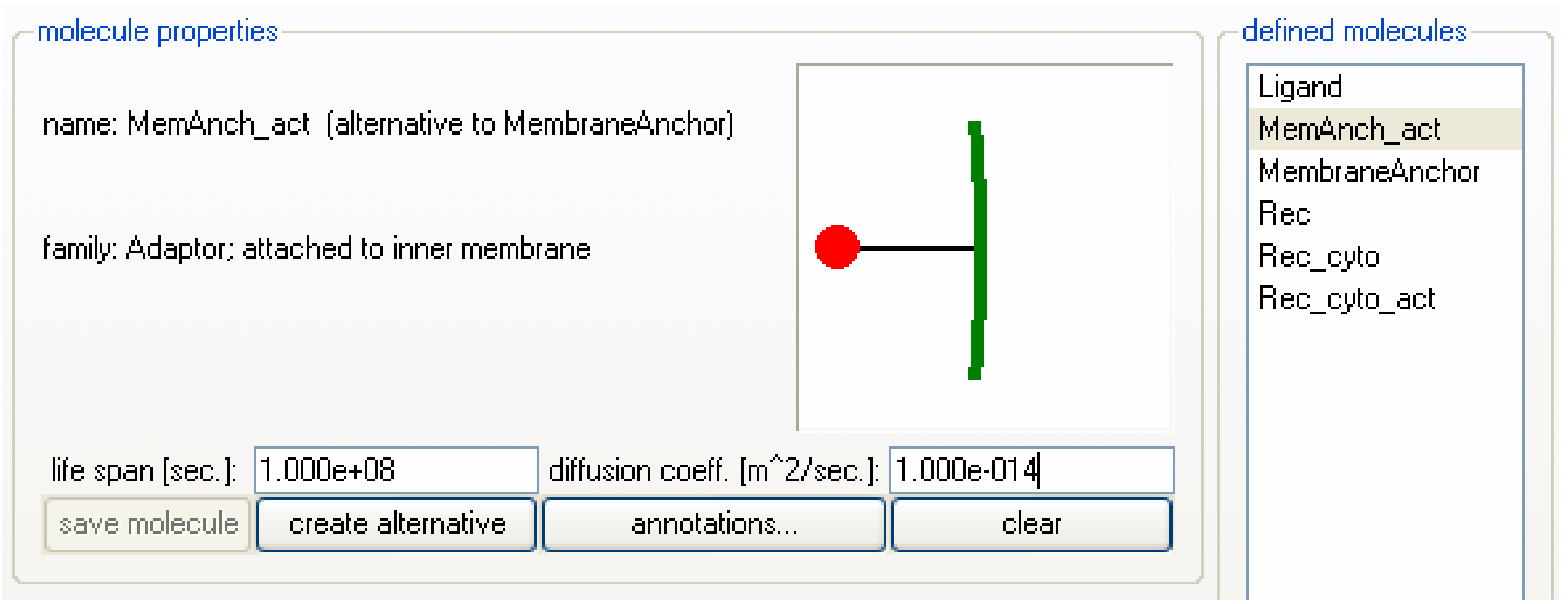

(21) Create a Kinase called ‘Activator’. Define it to have two binding sites, with the first one being enzymatically active.
Set the molecule’s diffusion coefficient to 1.0e-11.

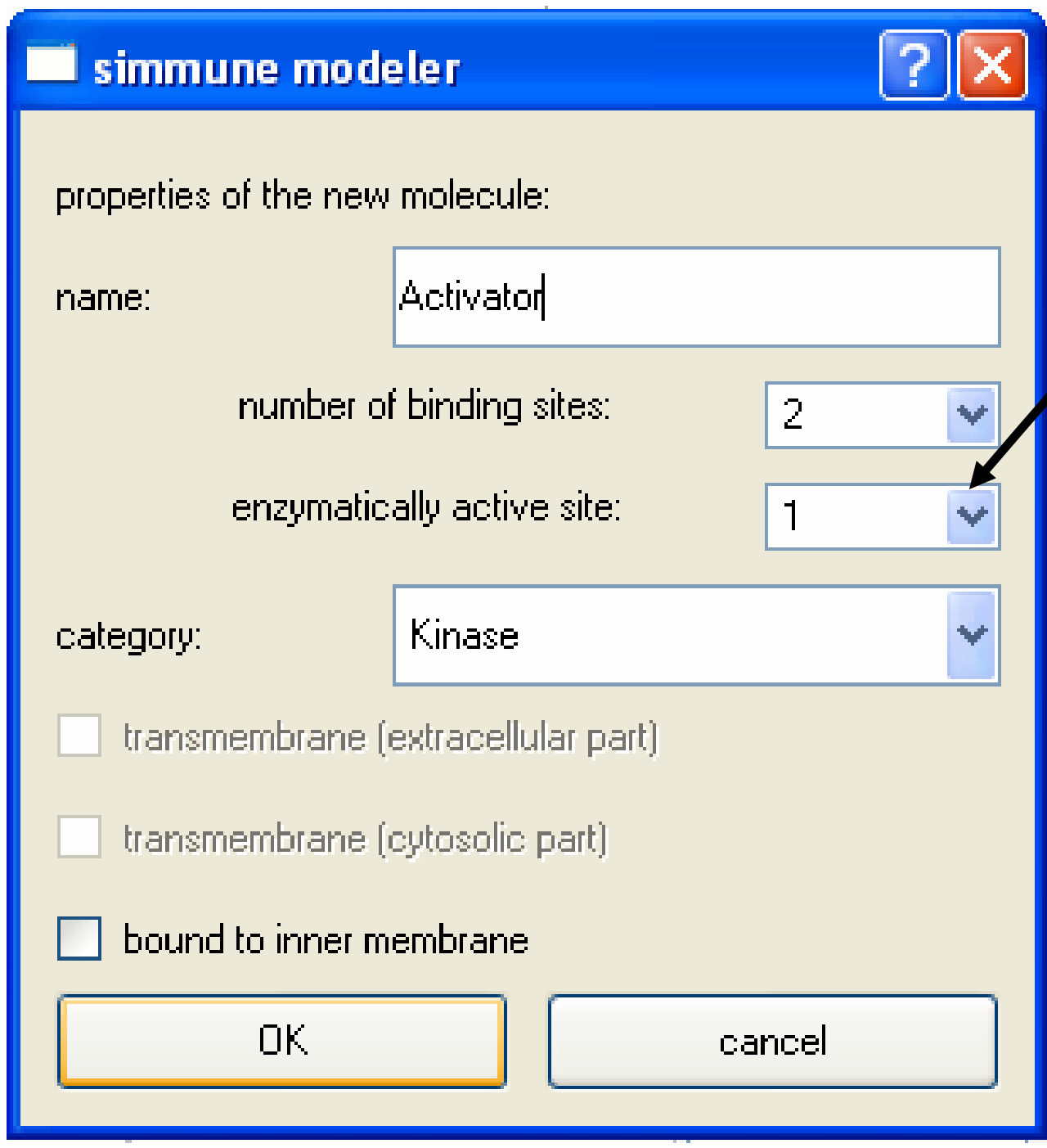

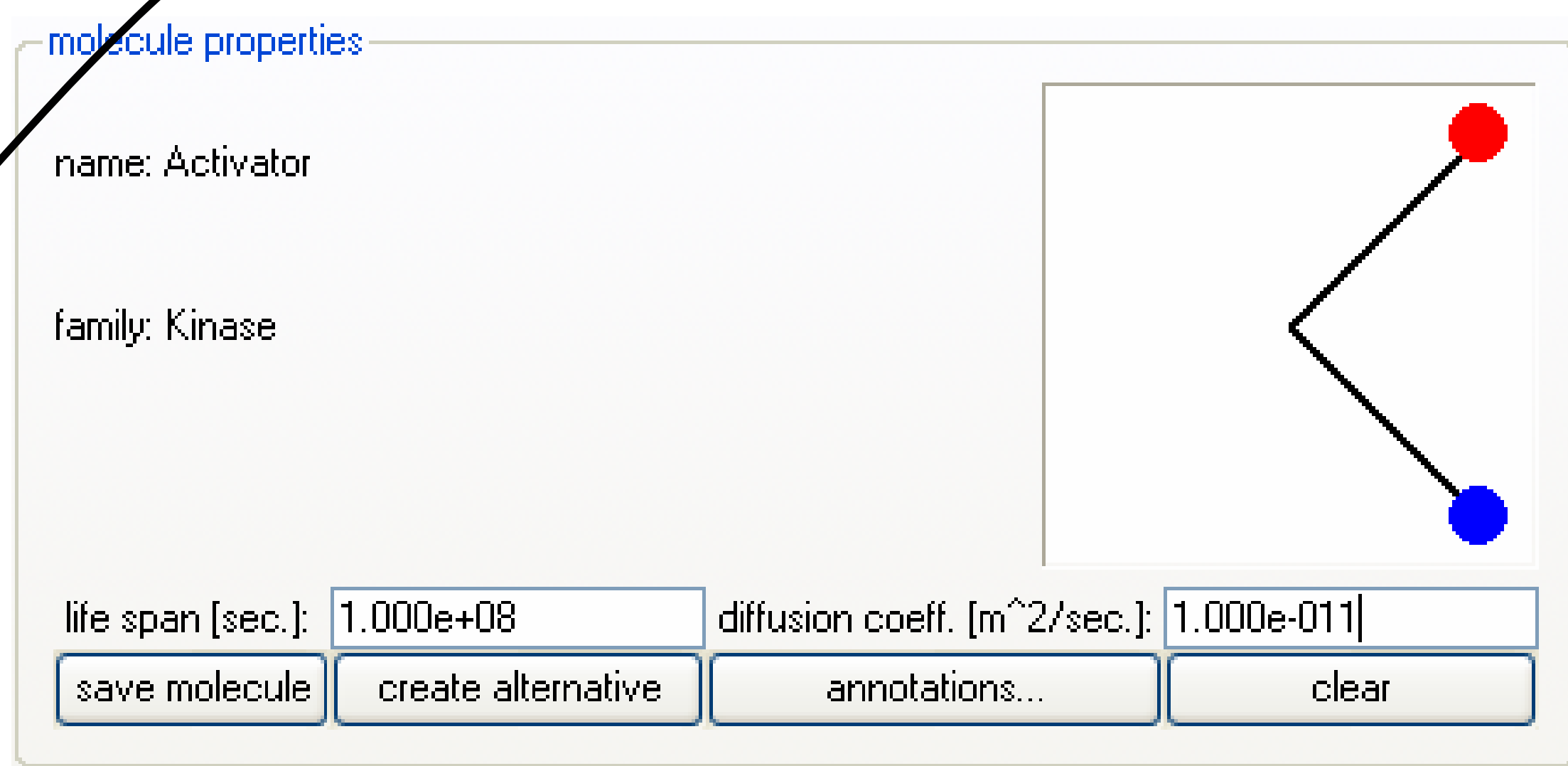

(22) Create an activated state of the ‘Activator’. Call it ‘Activator_act’. Set its diffusion coefficient to 1.0e-11.

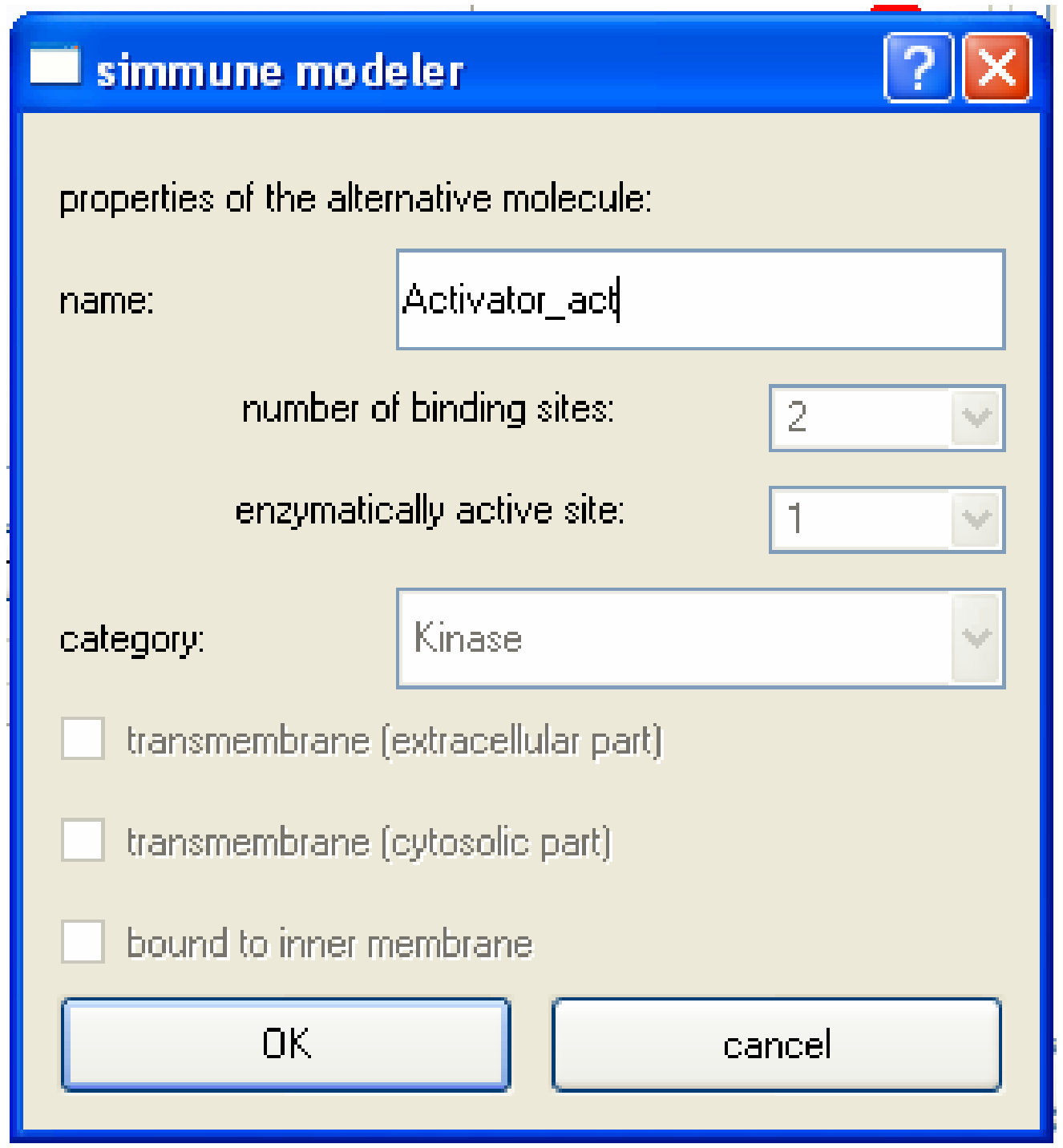

(23) Create a binding possibility between the binding site of Rec_cyto_act and the first (enzymatically active – indicated by the turquoise ring) binding site of the Activator.

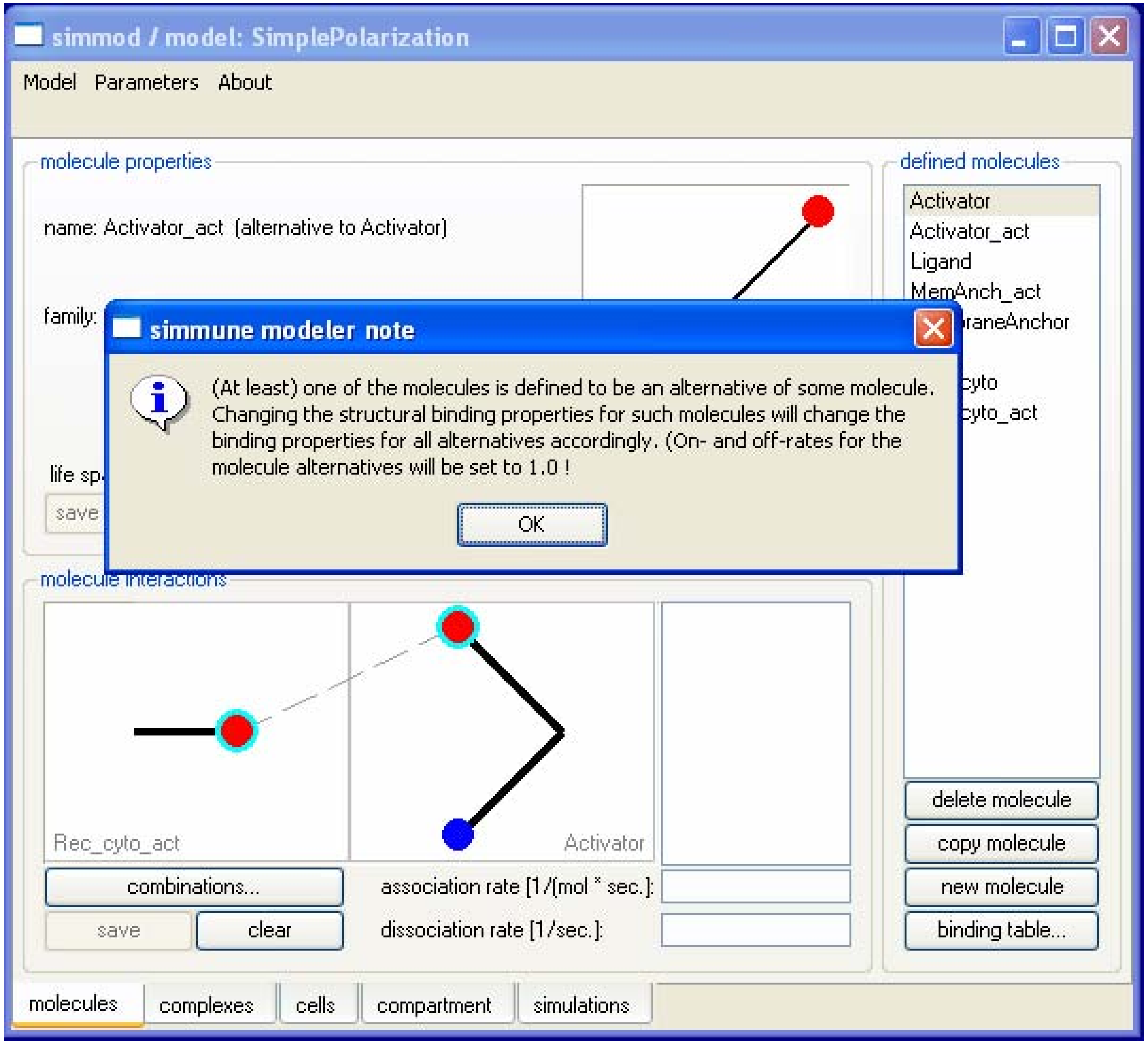

The software will create default binding possibilities between all alternatives of the two molecules. We will subsequently adjust their interaction parameters.

(24) The binding between Rec_cyto_act and the Activator will lead to the activation of the Activator. Set the association and dissociation rates as below.

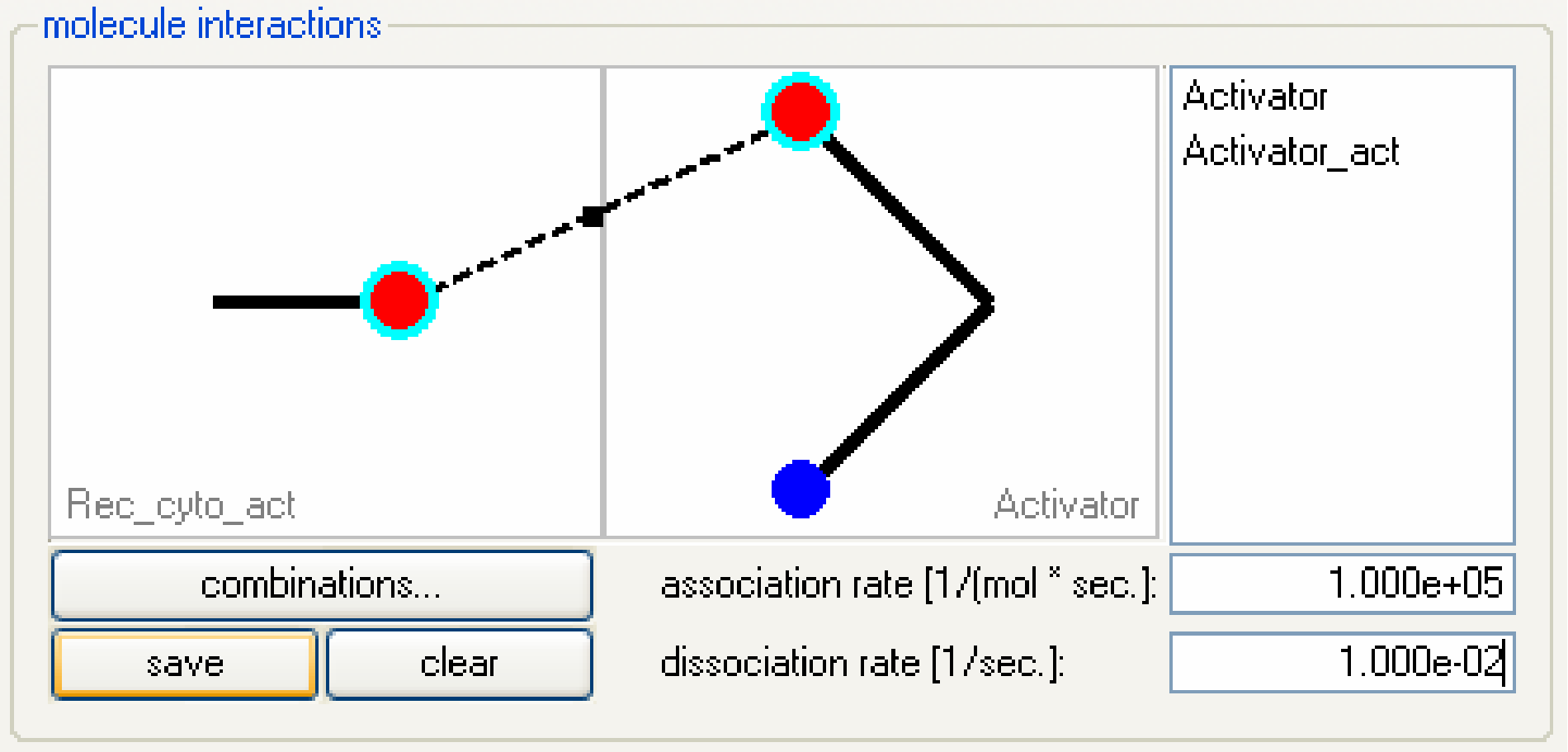

(25) The offrate between Rec_cyto_act and the Activator_act will be the rate at which the activated Activator is released by the intracellular receptor domain. Set it to 1.0e2.

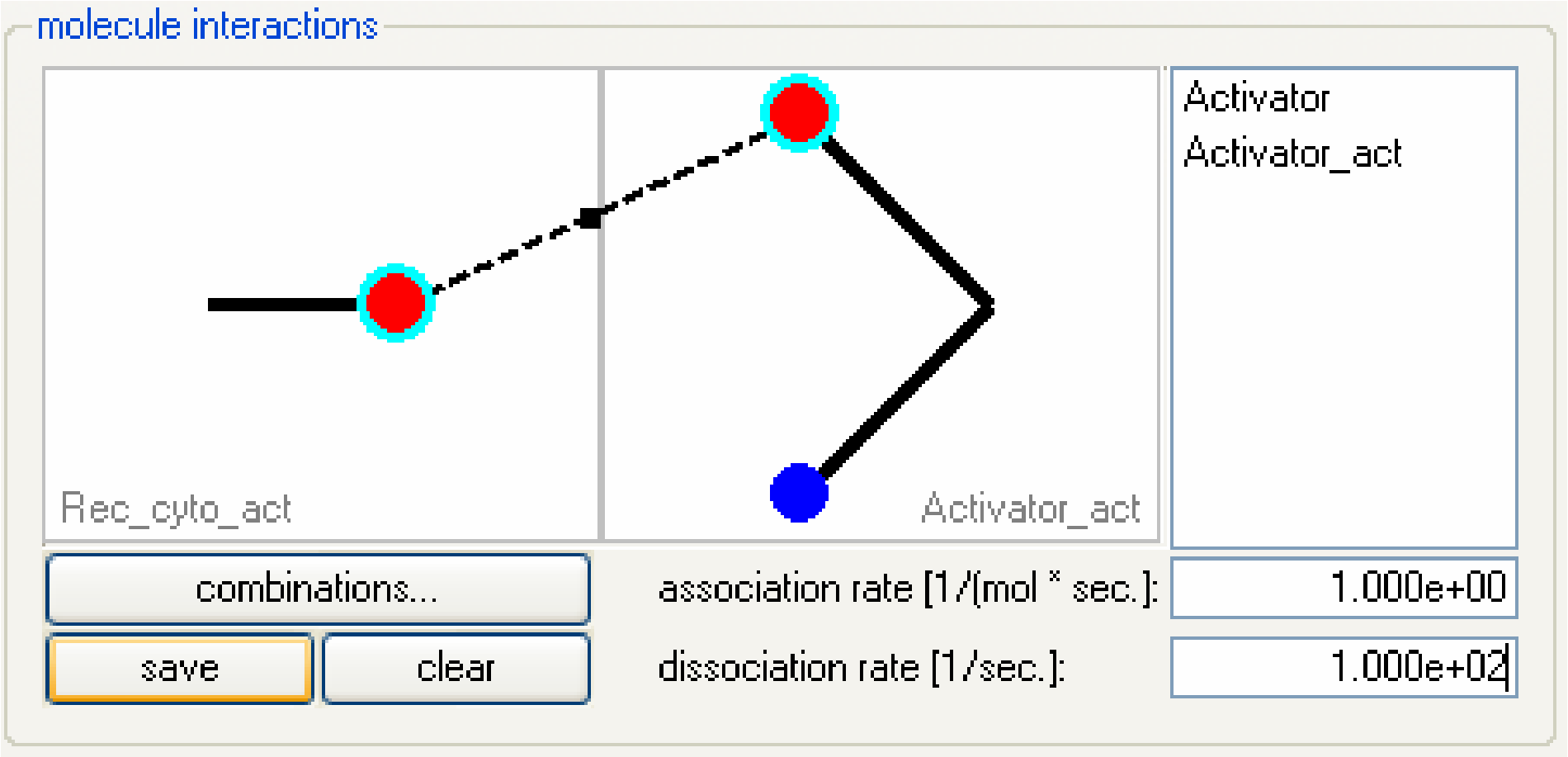

(**26**) Set the dissociation rates between Rec_cyto and the two Activator states to 10. This means that the receptor, should it become deactivated by losing the extracellular Ligand, will let go of the Activator quickly.

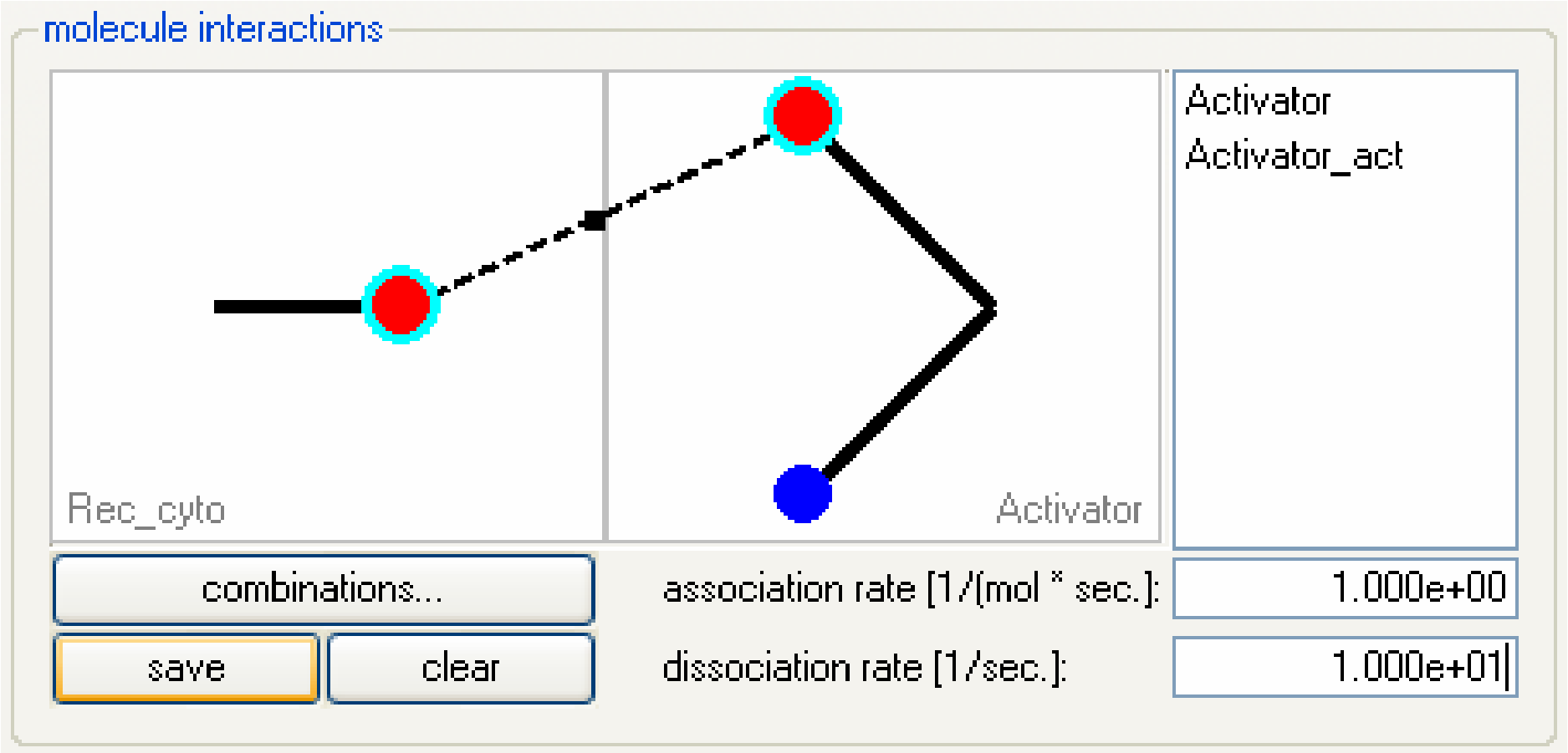

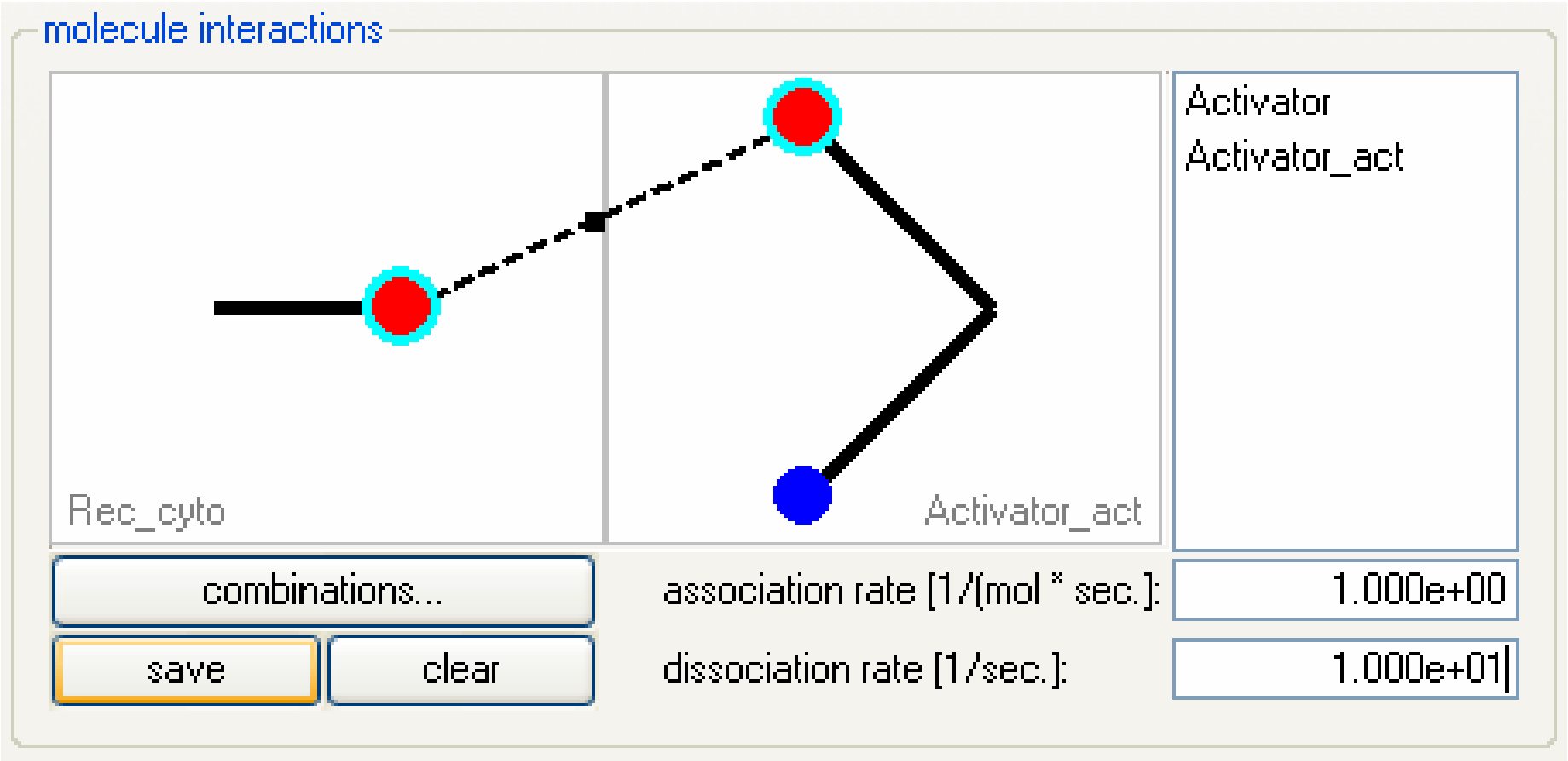

(27) Create a phosphatase called ‘Inhibitor’ with the properties shown below.

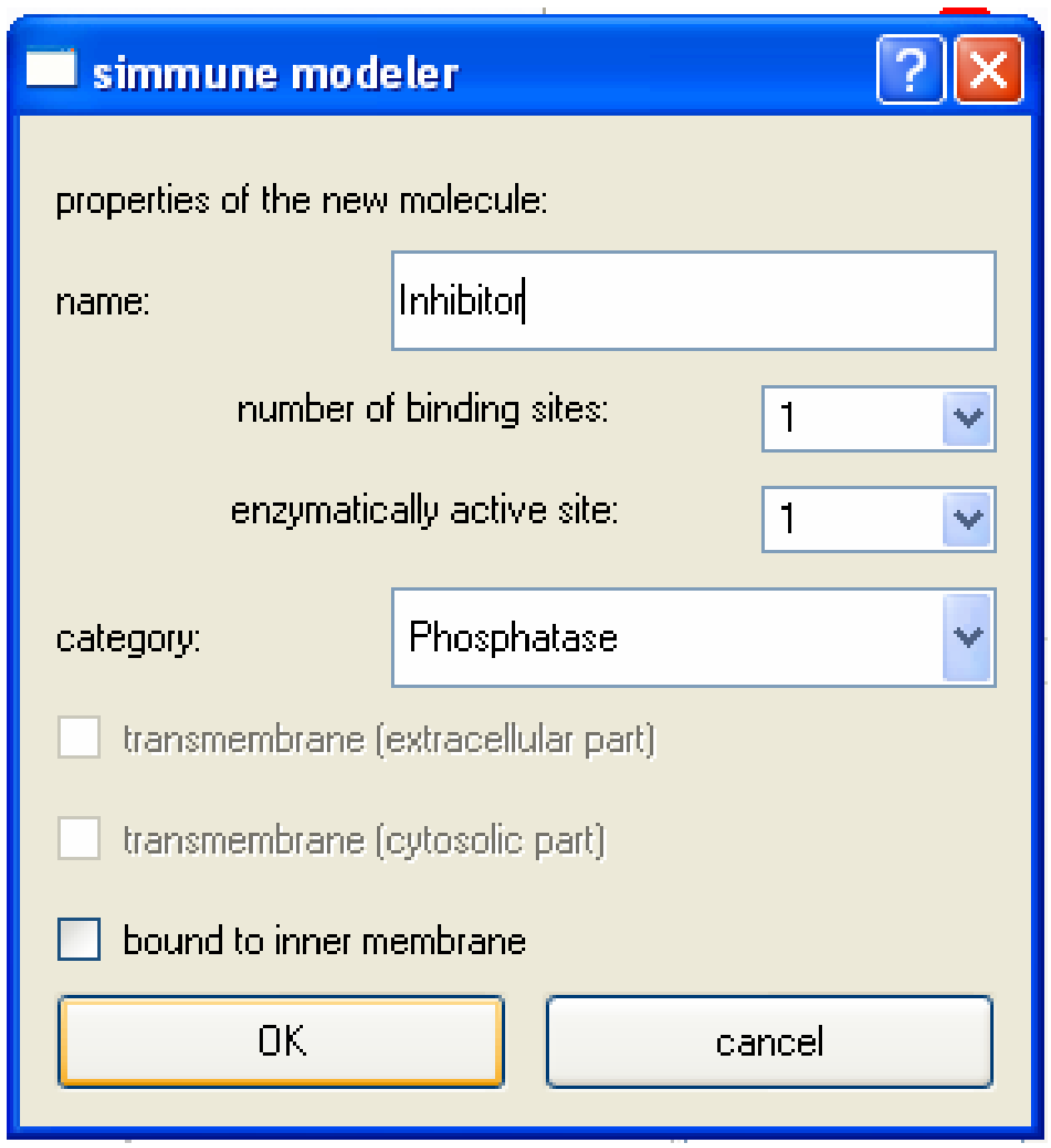

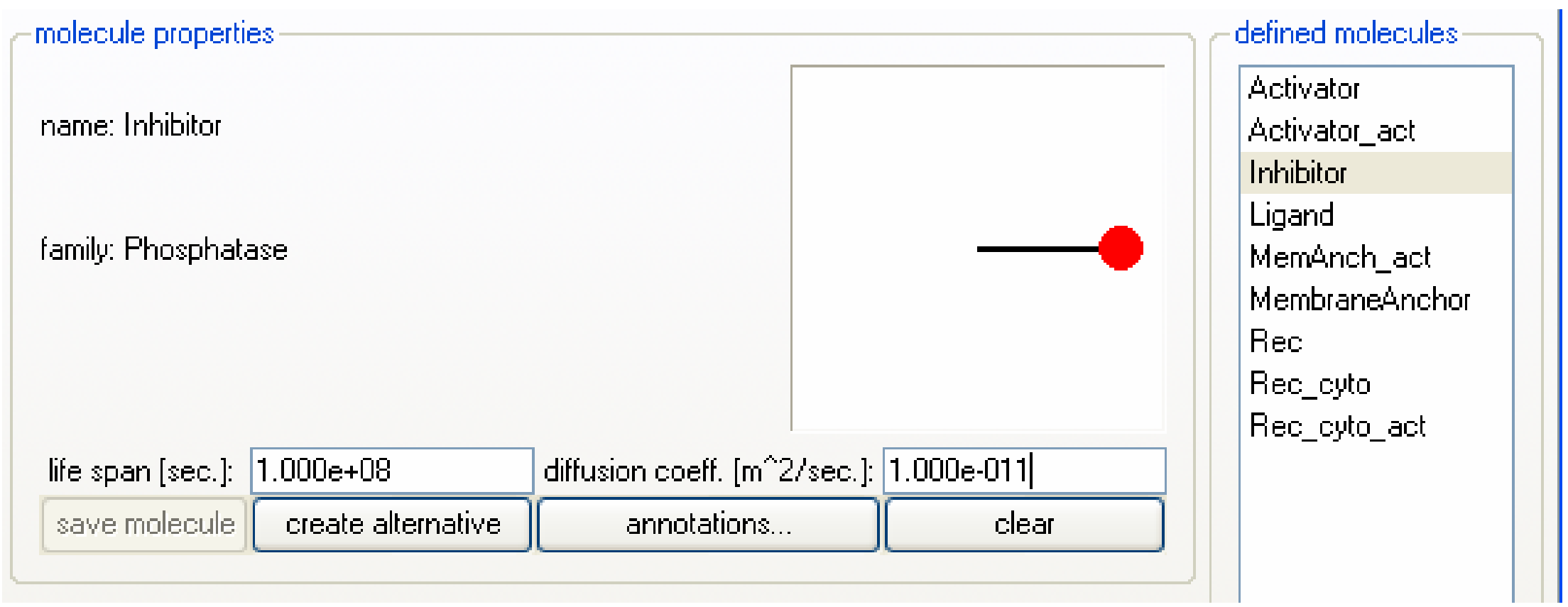

(28) Create an alternative of the Inhibitor with the properties shown below.

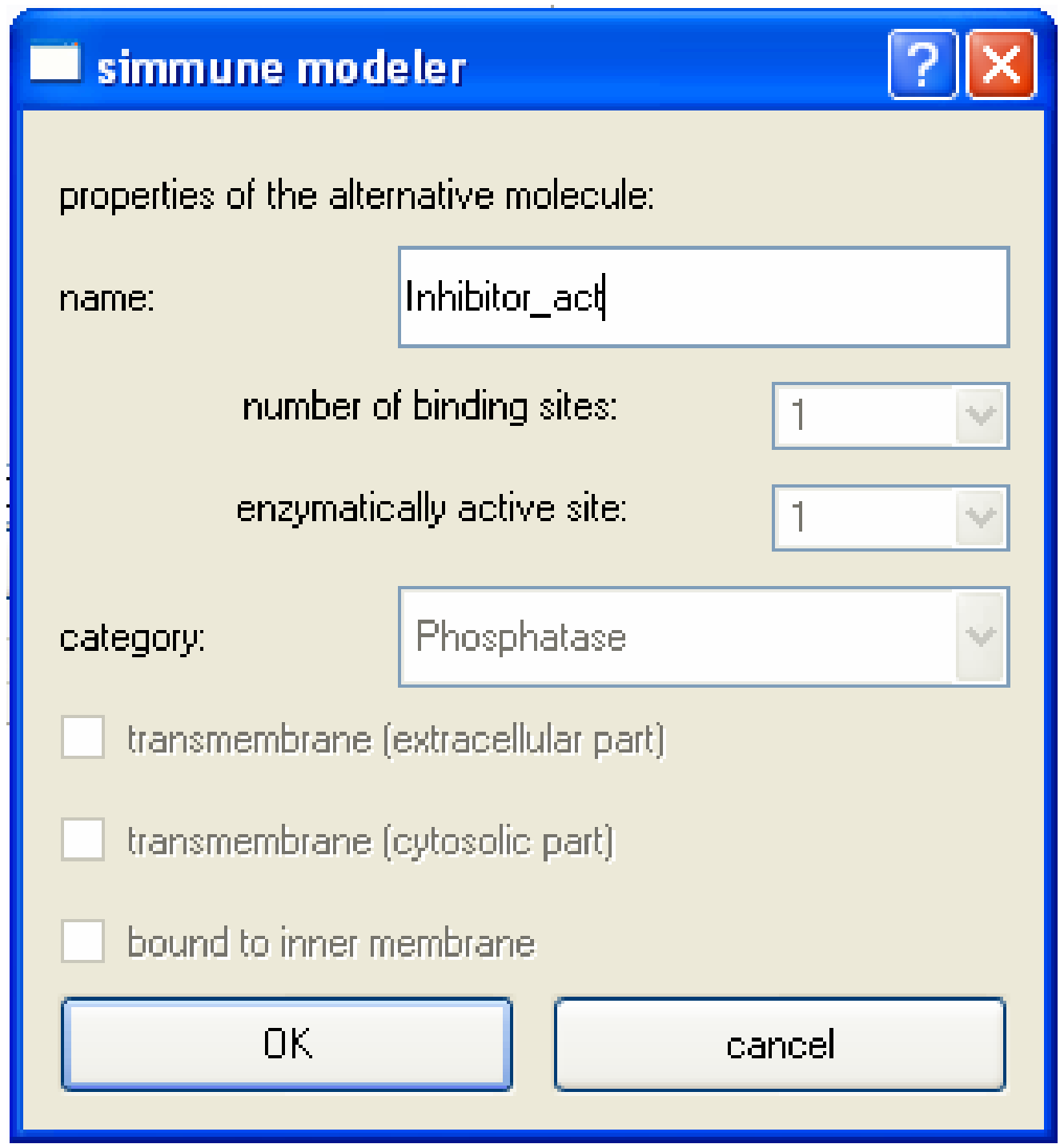

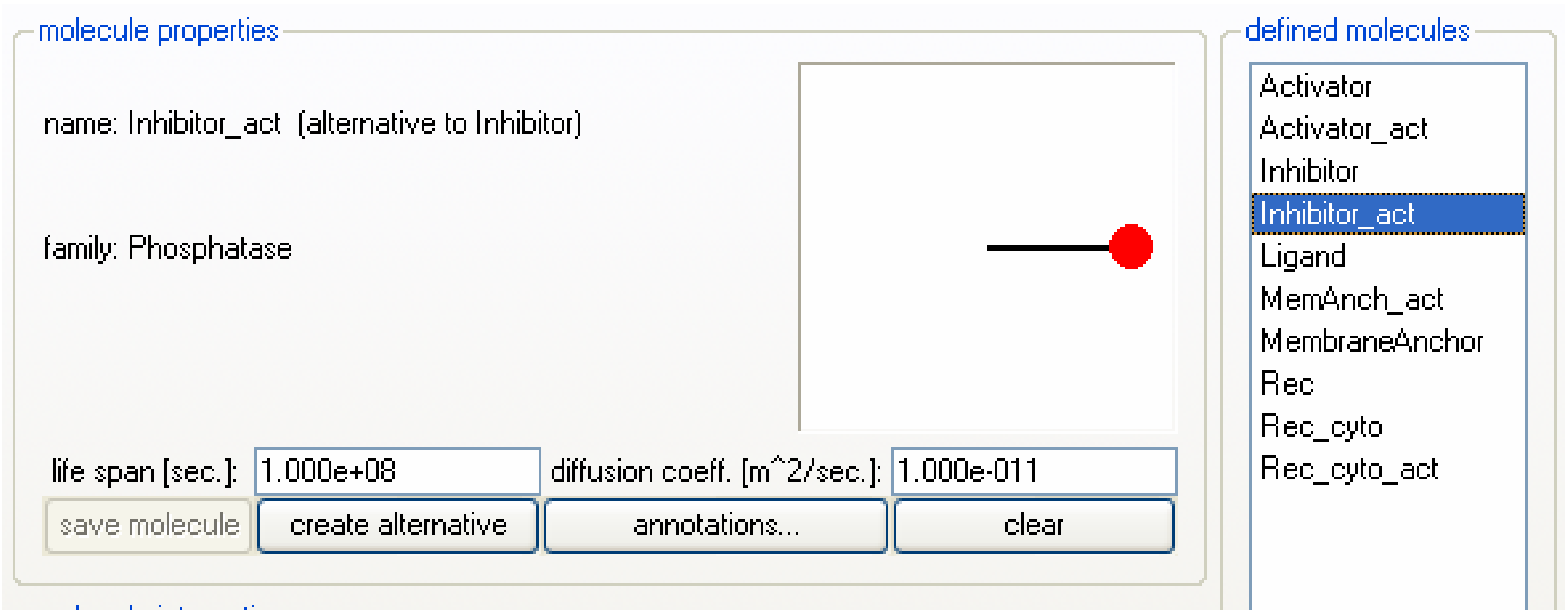

(**29**) Create interactions between the two states of the Inhibitor and the two states of the (cytosolic domain of) the Receptor as shown below.

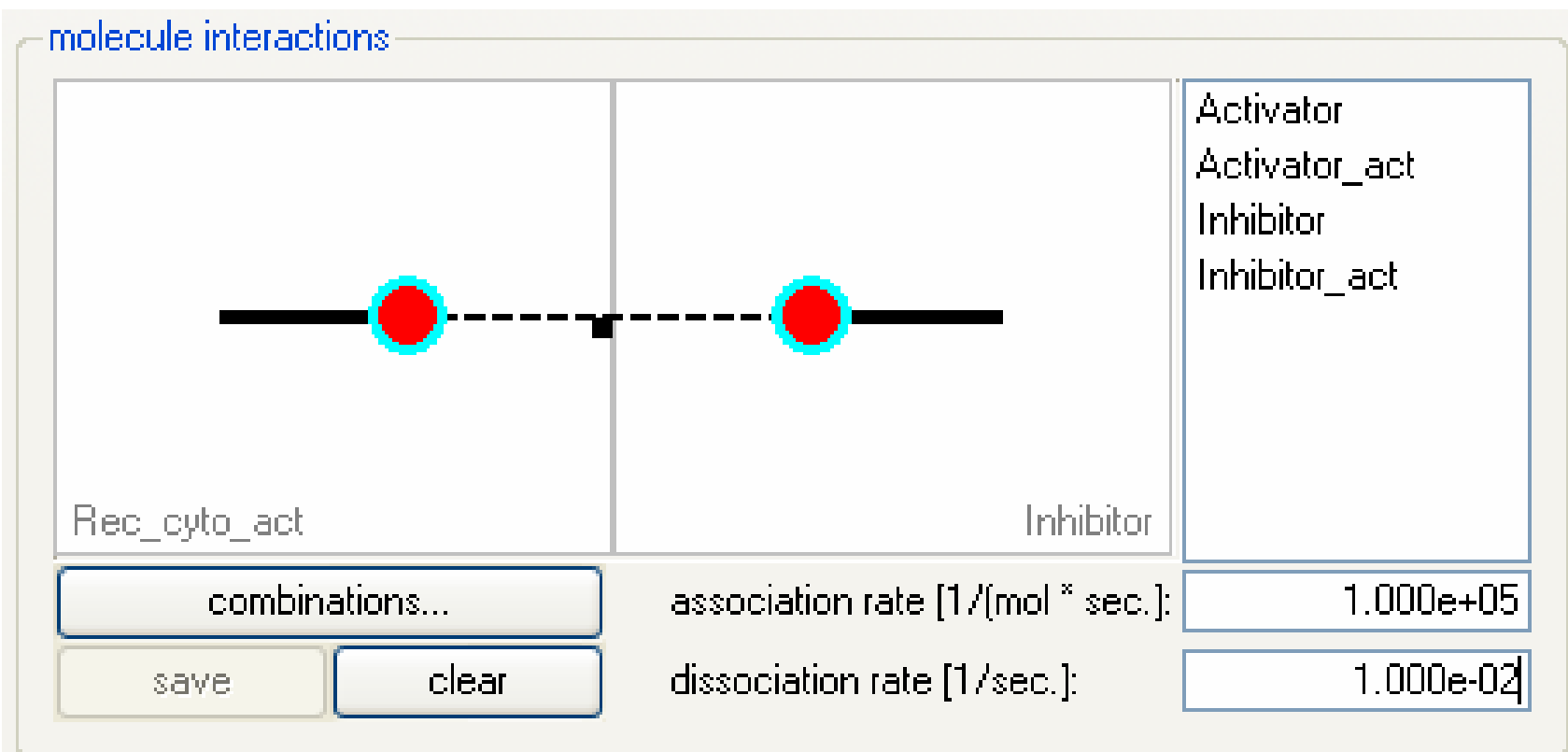

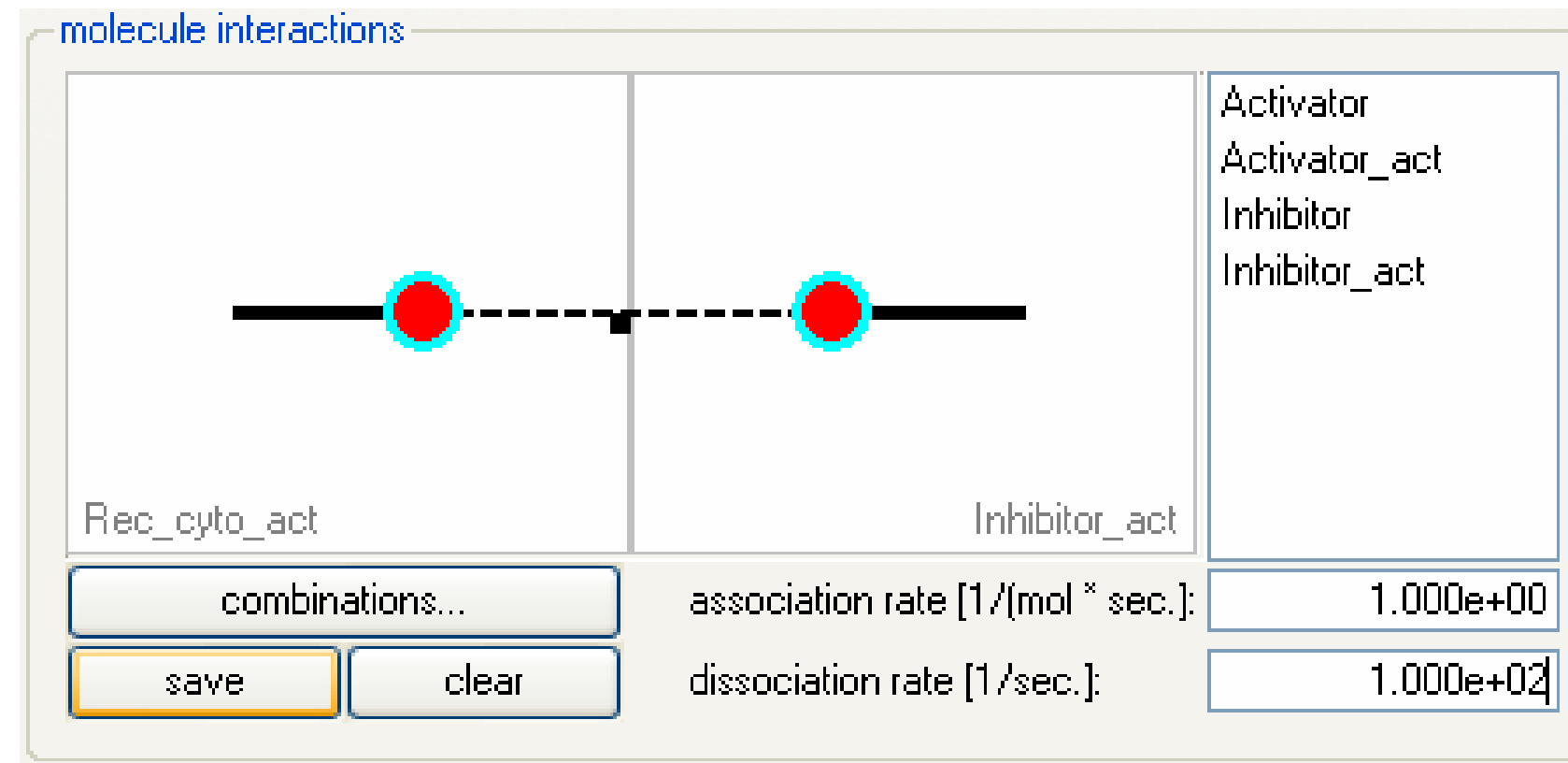

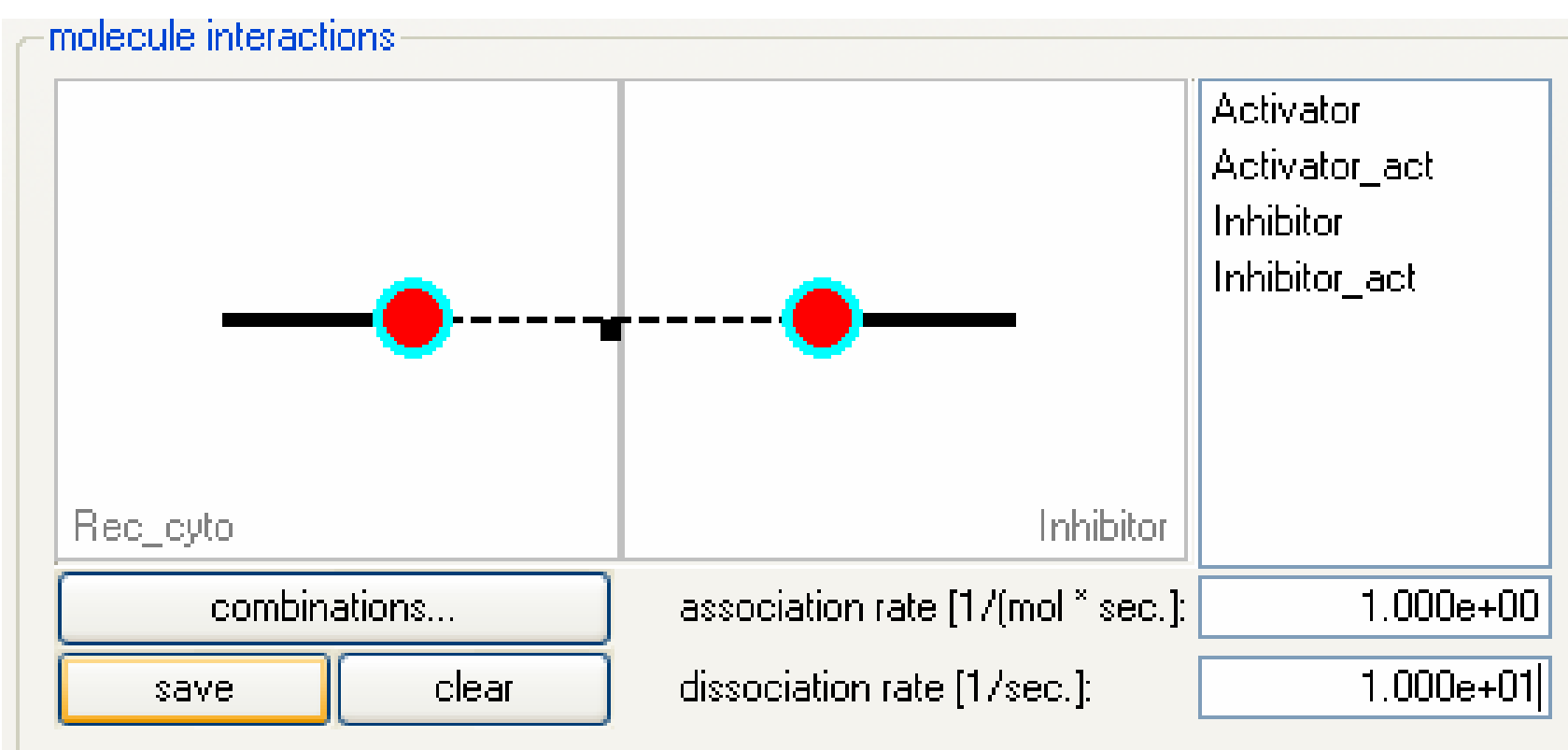

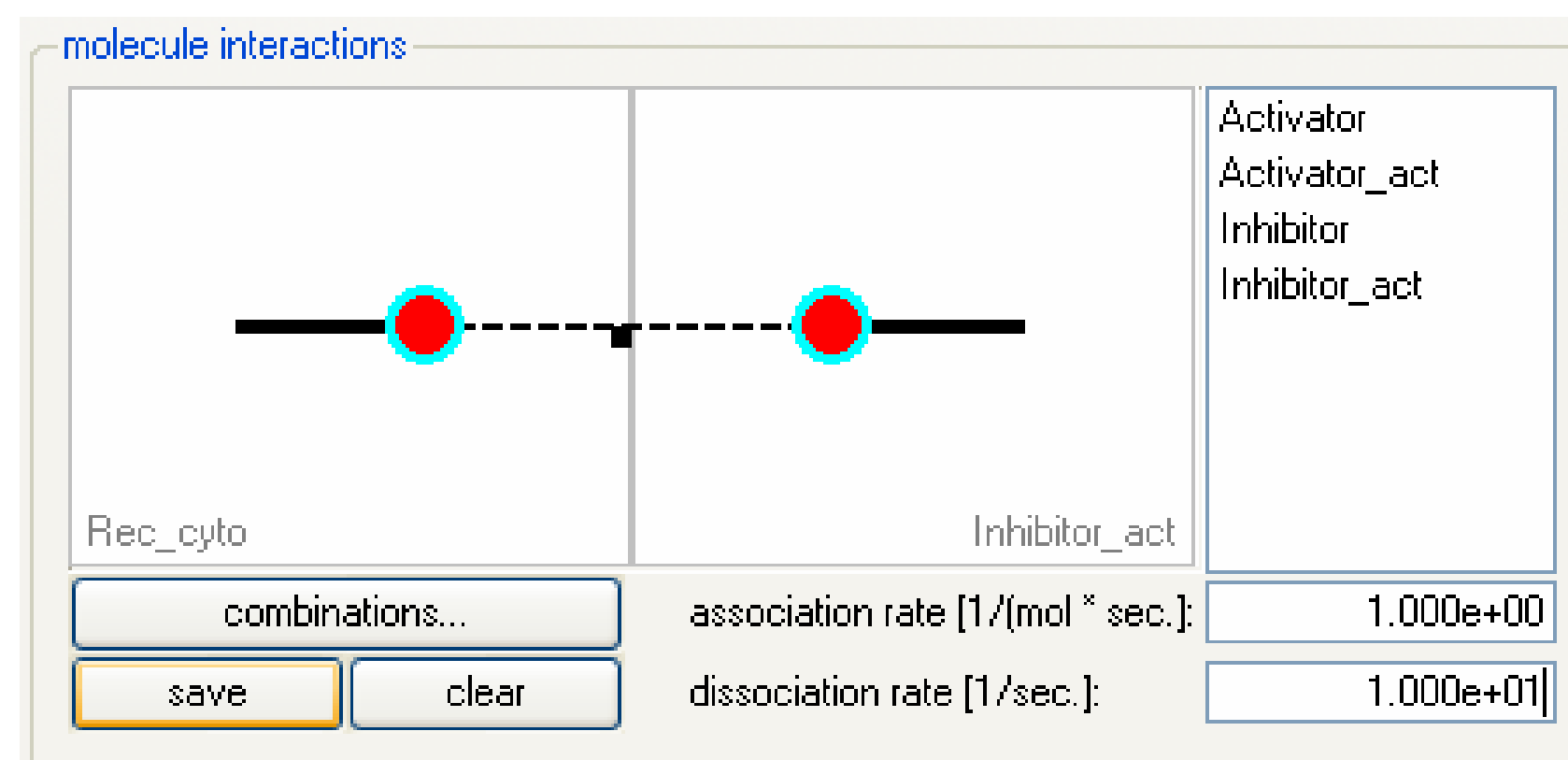

(30) Create interactions between the two states of the Activator and the two states of the MembraneAnchor as shown below.

Note: these interactions will be used to define enzymatic action of the Activator on the MembraneAnchor.

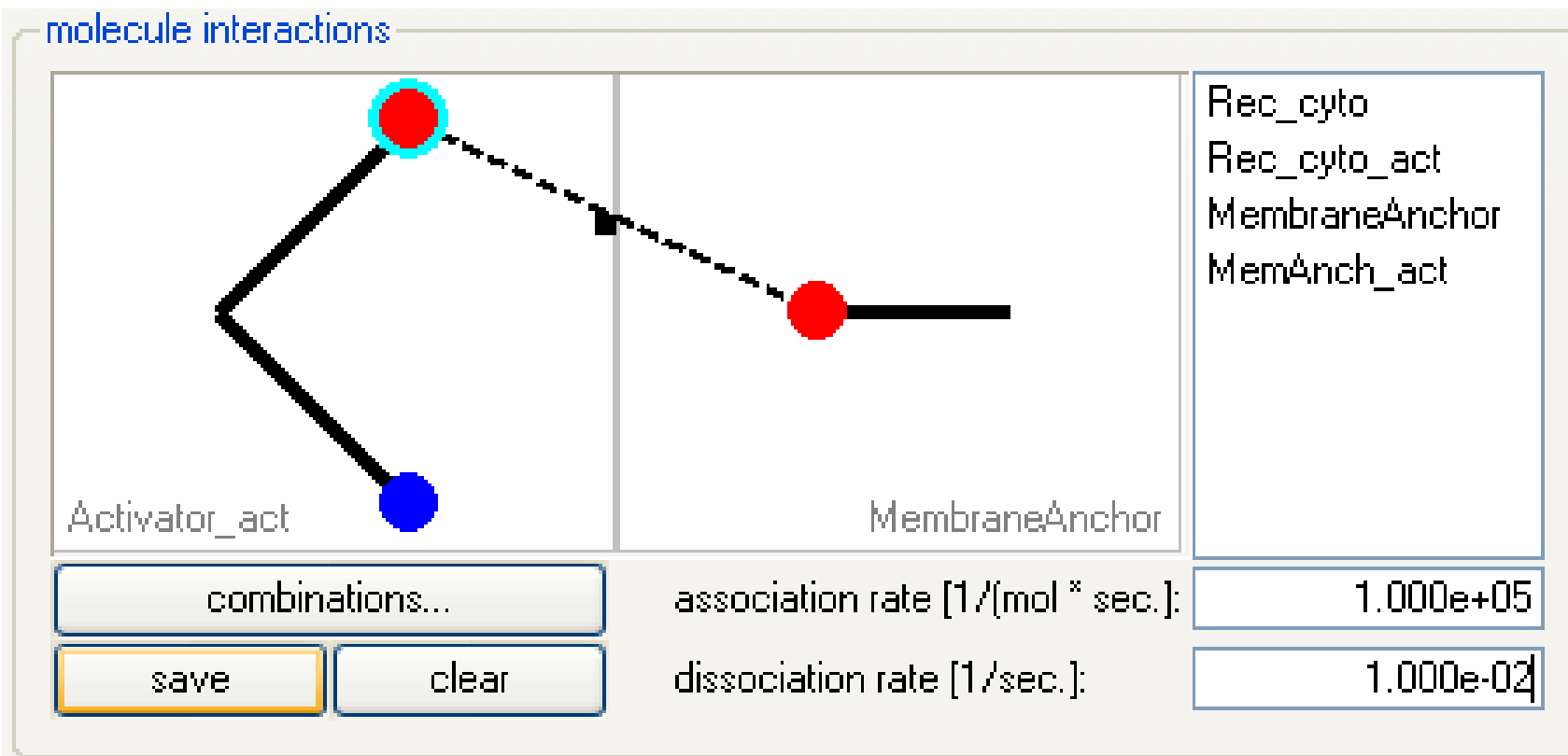

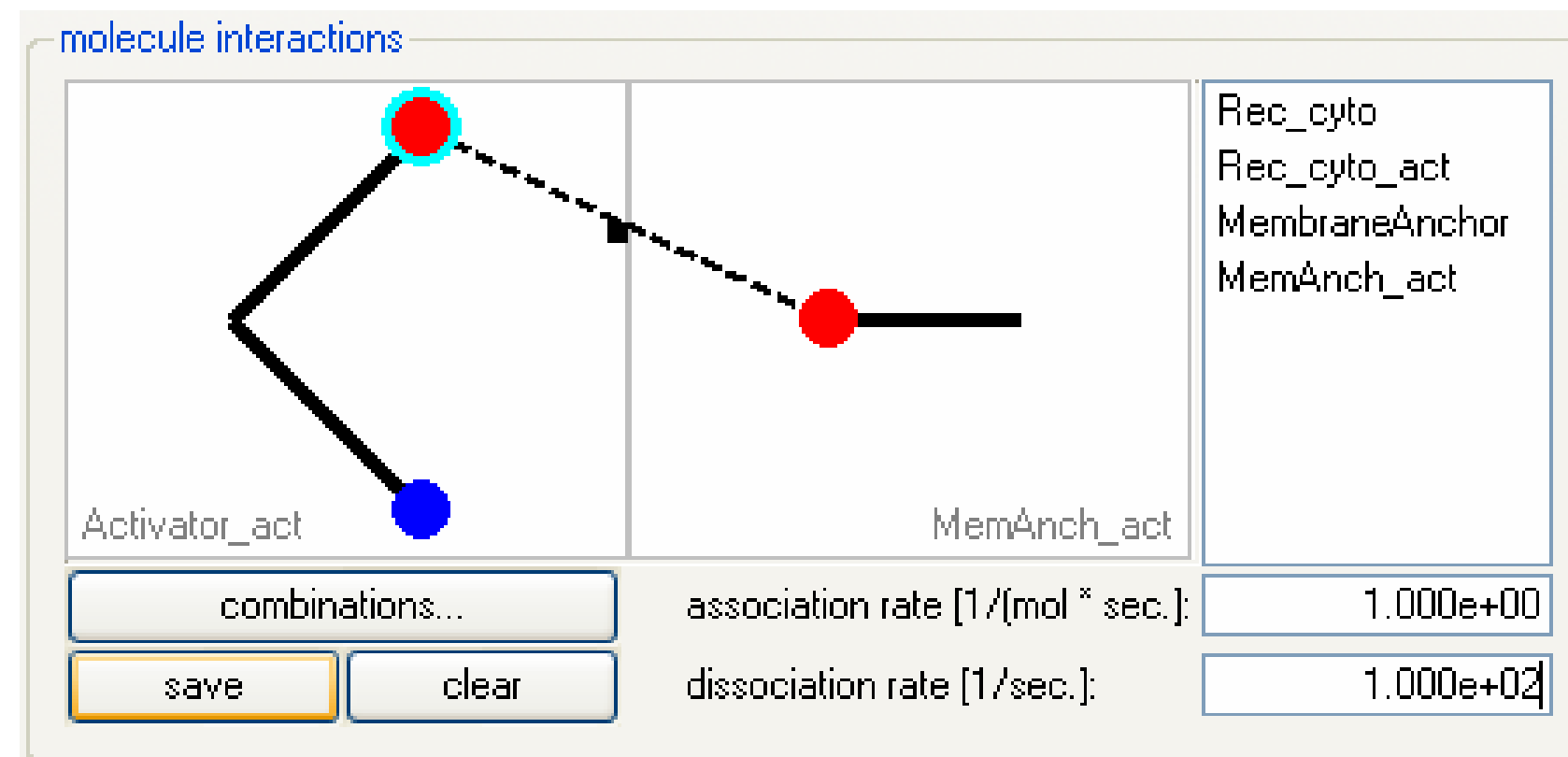

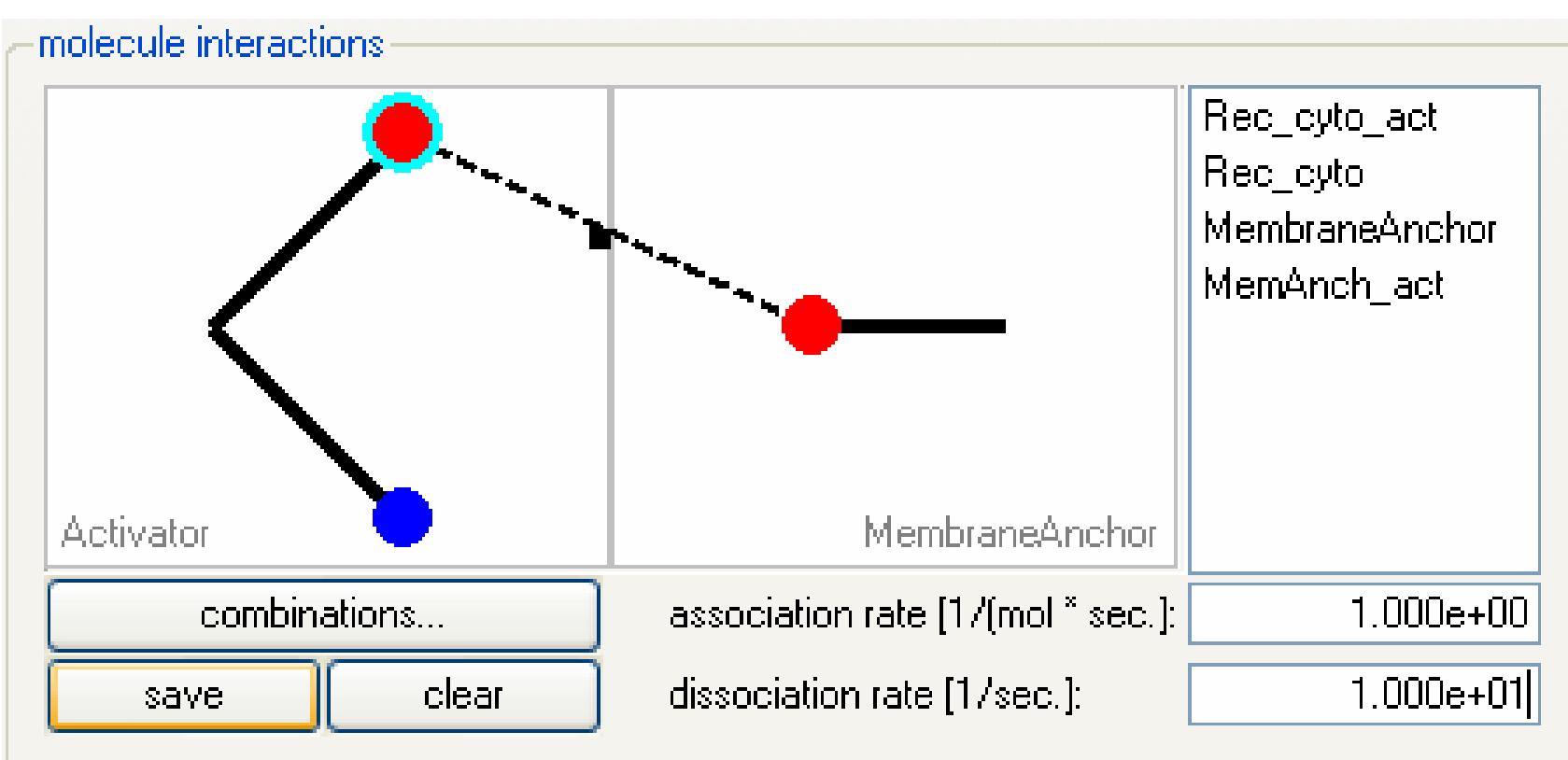

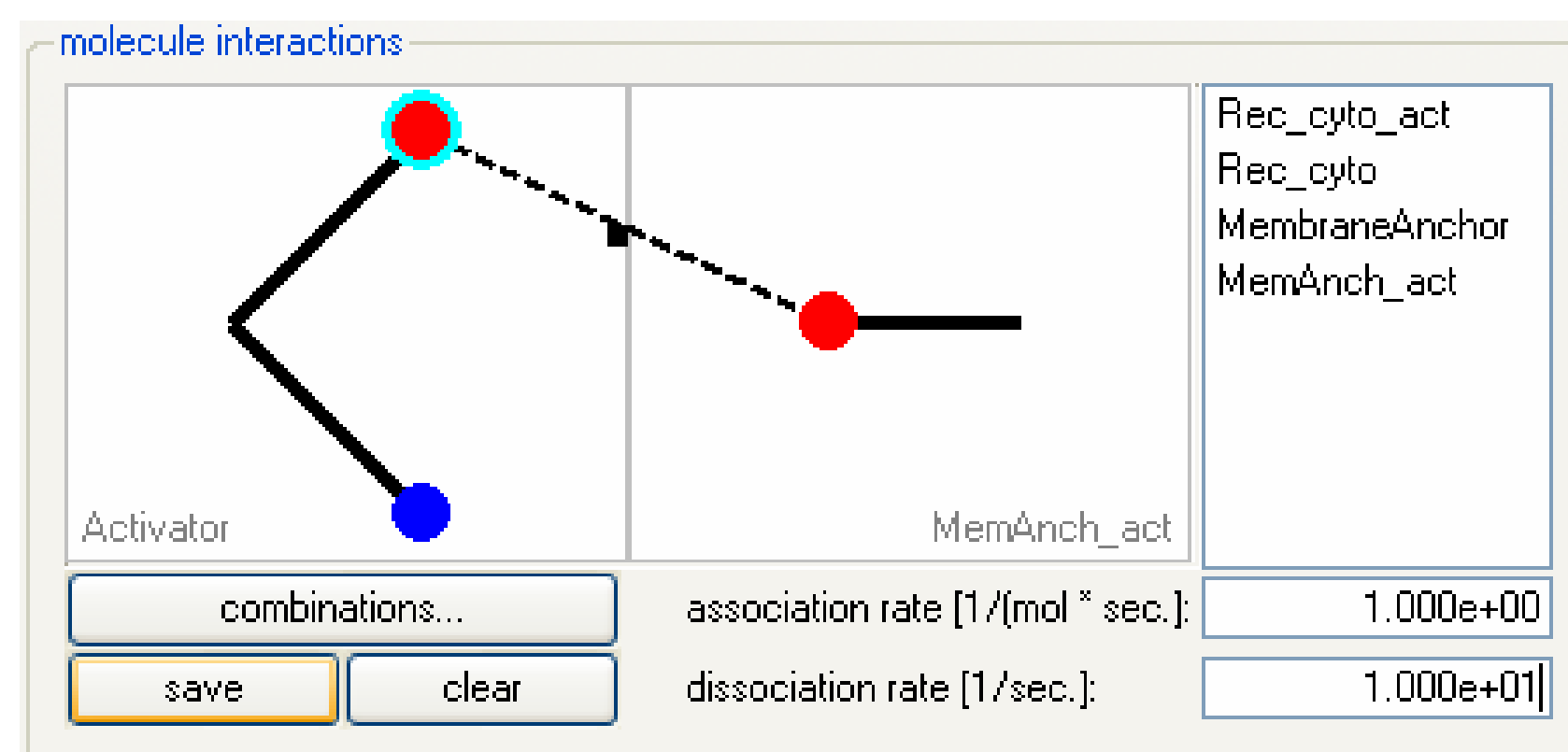

(31) Create interactions between the two states of the Inhibitor and the two states of the MembraneAnchor shown below.

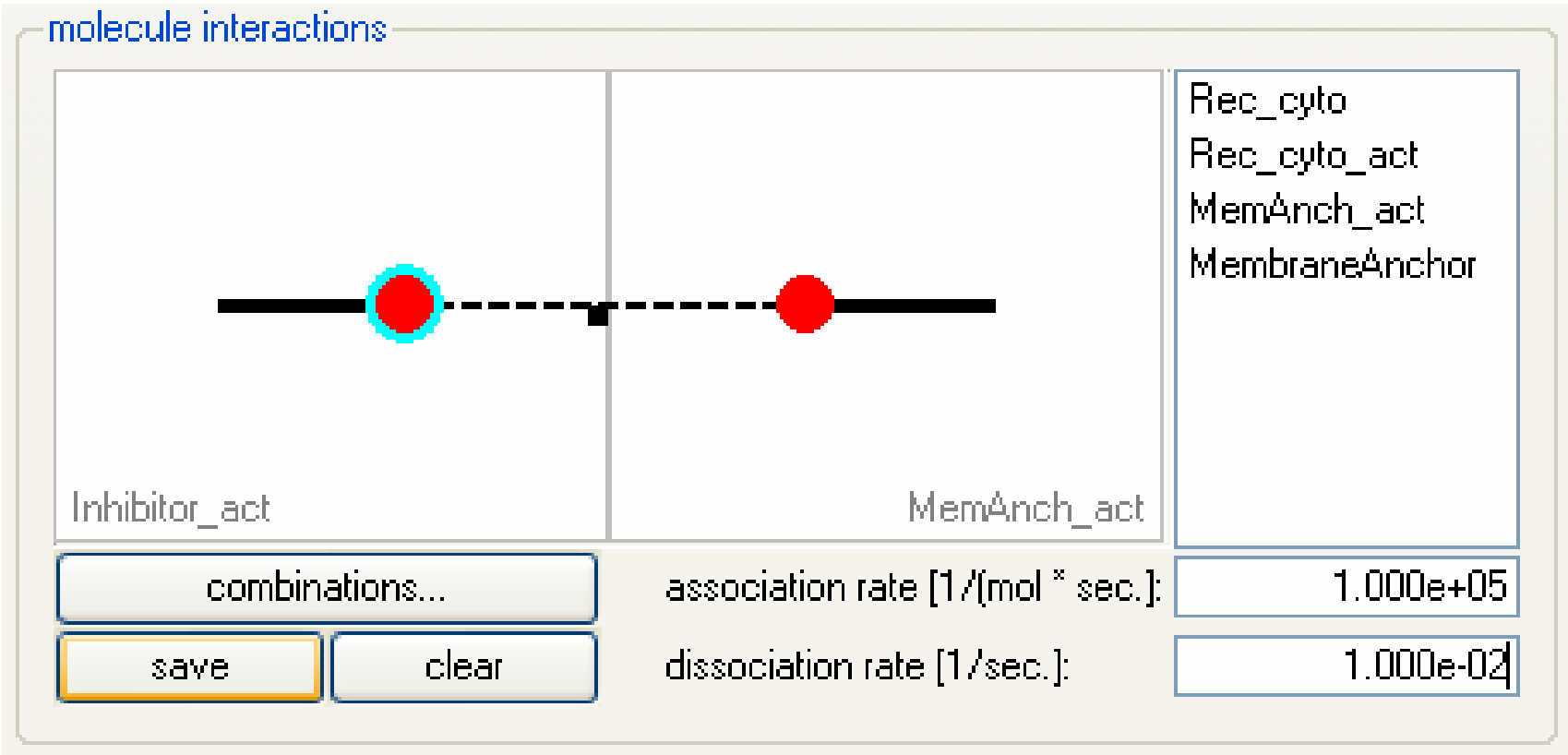

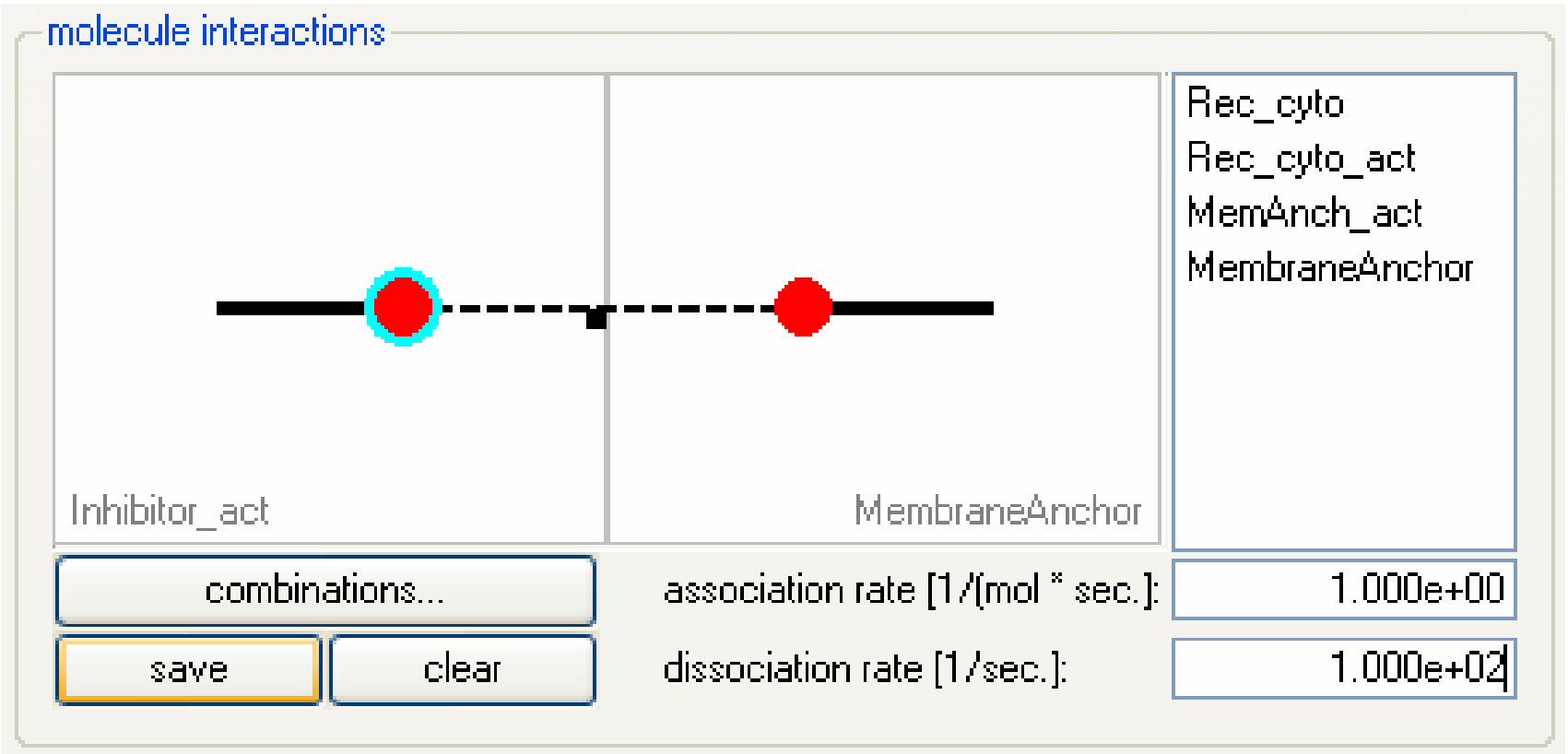

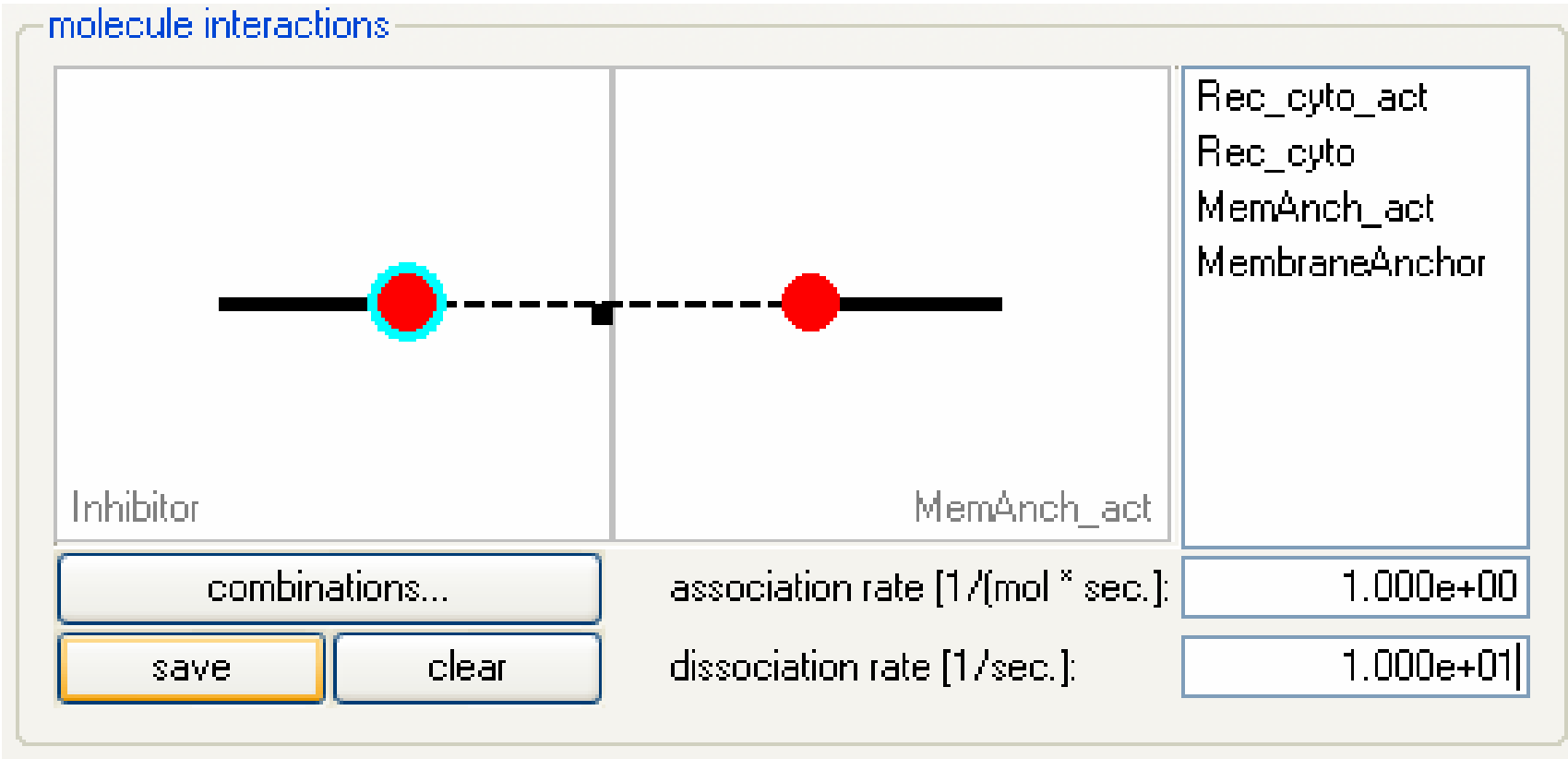

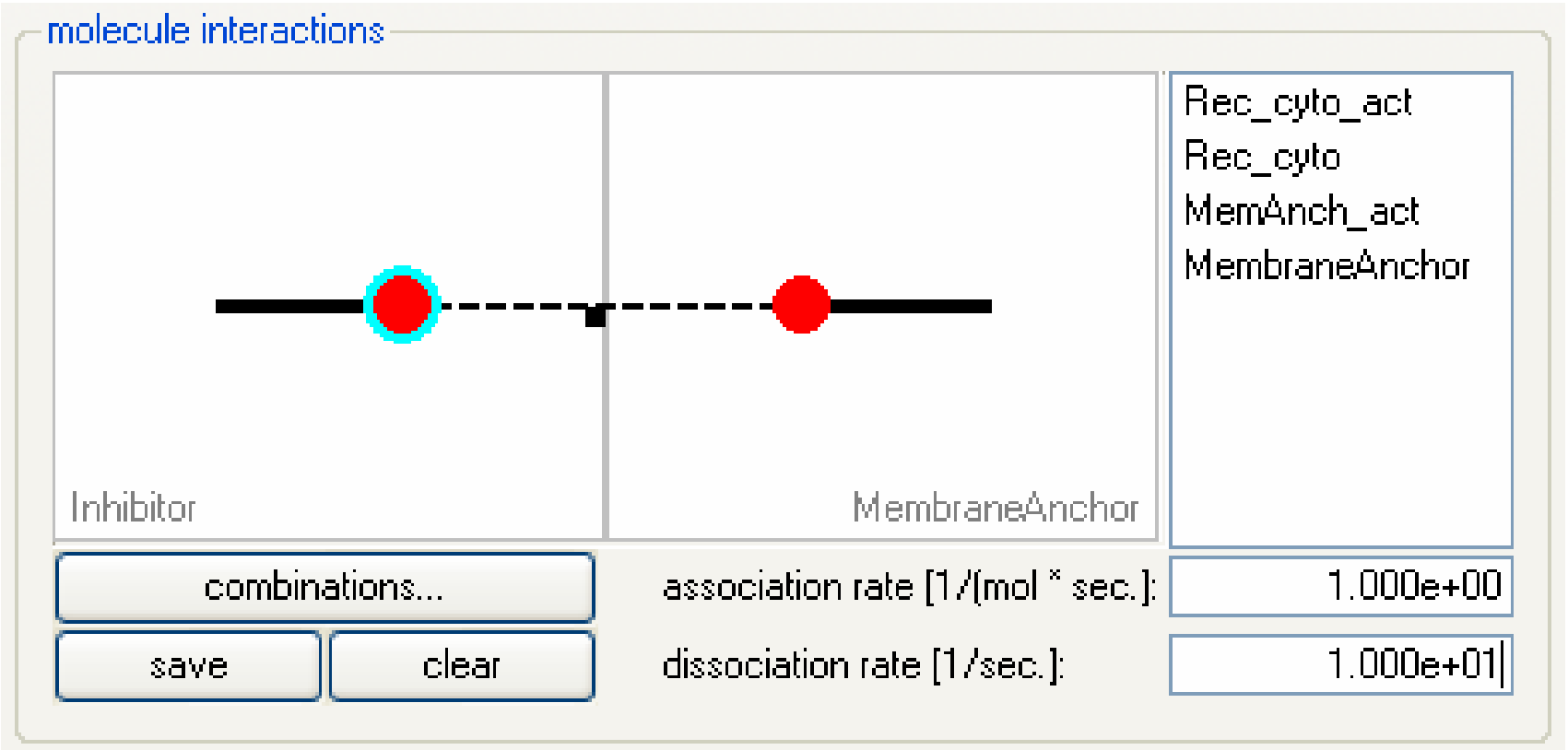

(**32**) Now we create interactions between the two states of the Activator and the two states of the MembraneAnchor in such a way that the activated MembraneAnchor allows attachment of the active and inactive Activator via the second binding site of the Activator (that is not enzymatically active).

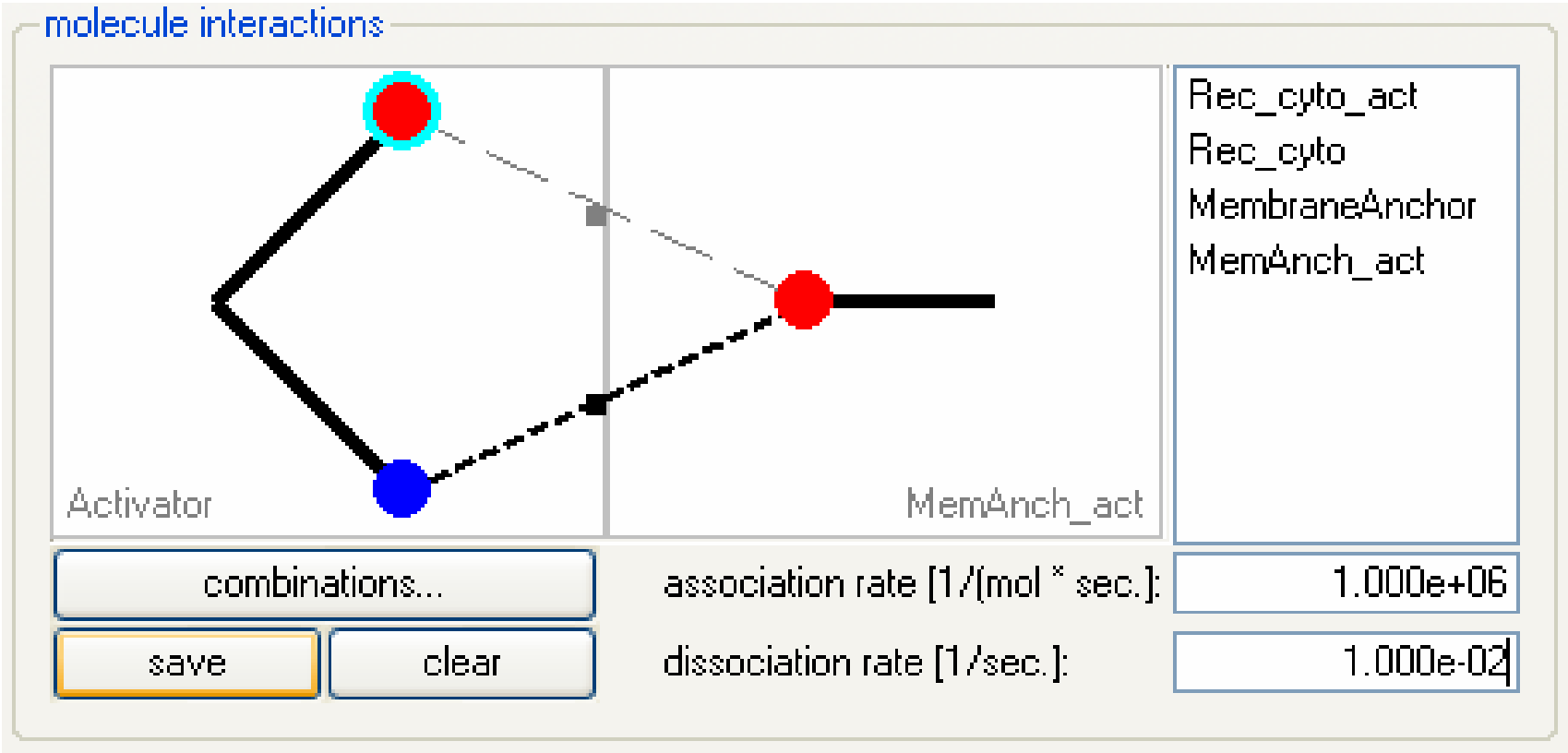

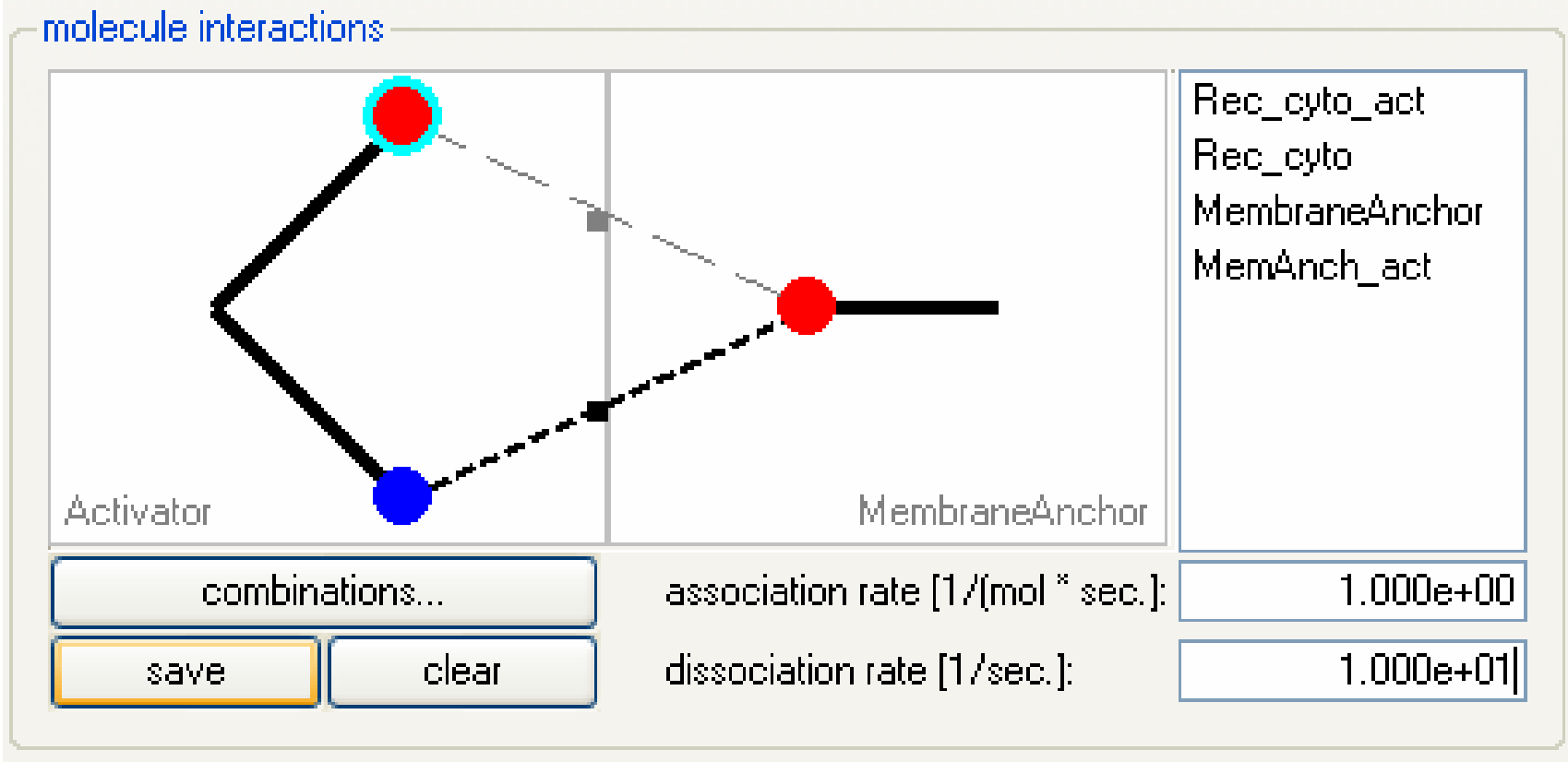

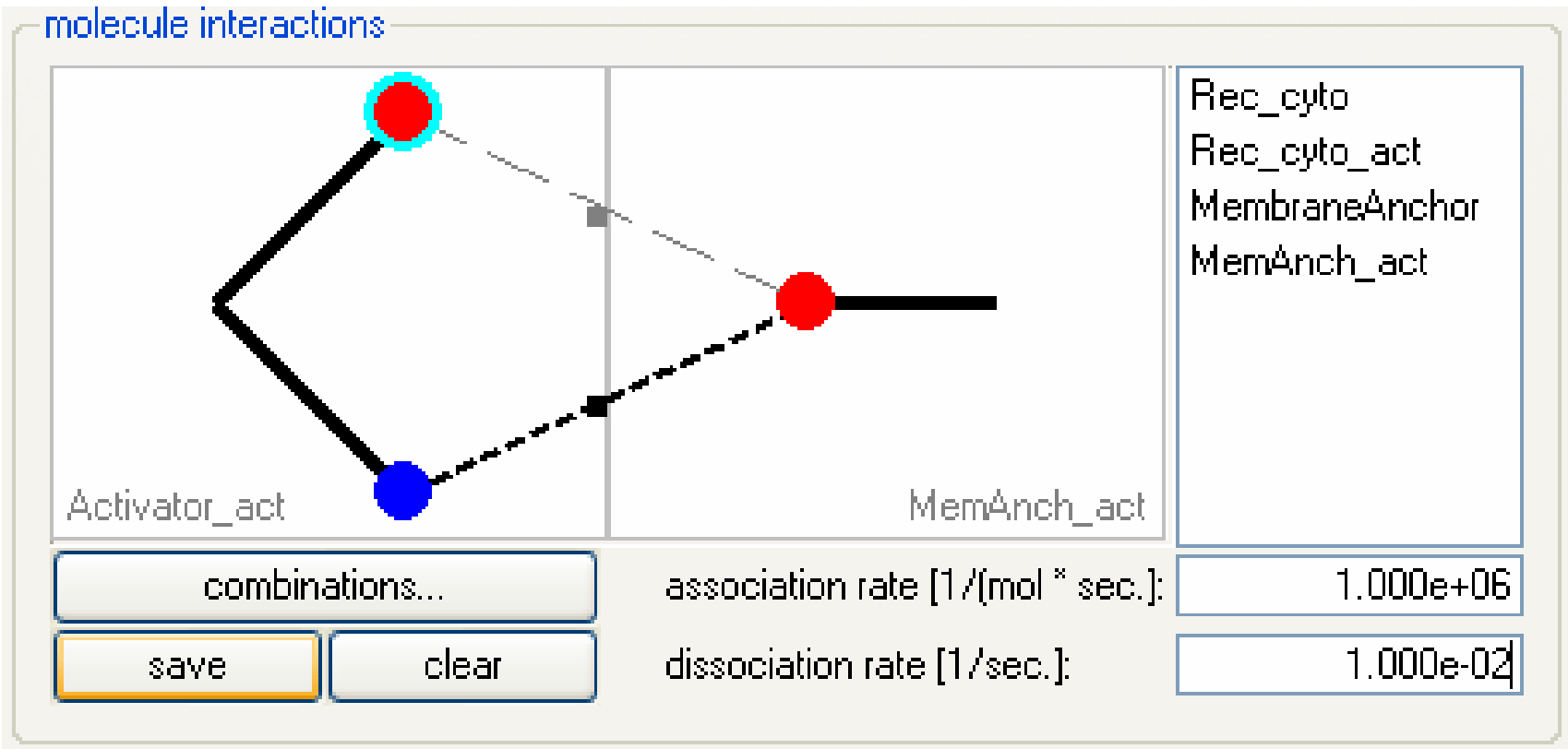

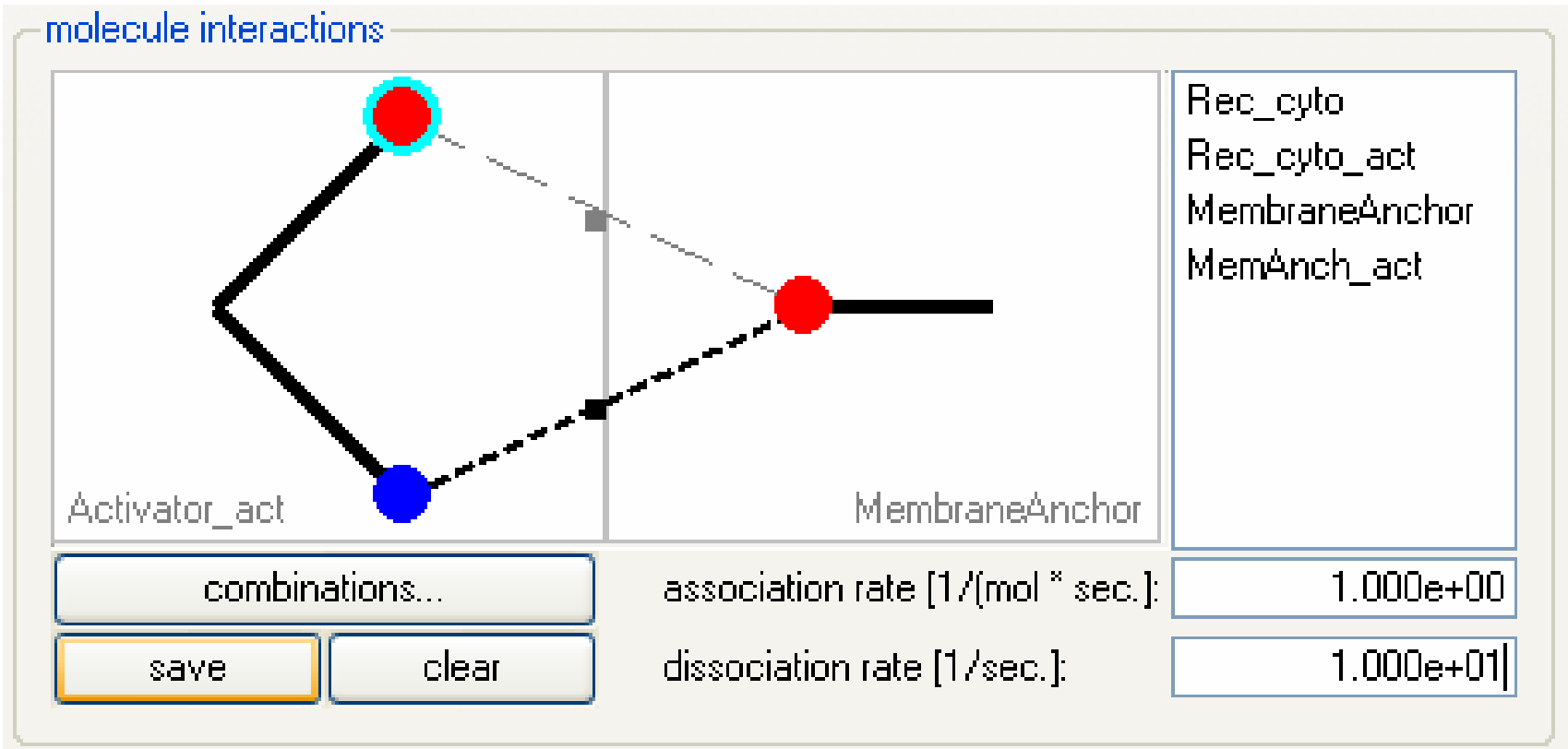

(33) Click on the ‘binding table’ button in the lower right corner to open a tabular display of the binding possibilities in the system so far.
It should look like shown below.
Clicking on an entry will make the software provide information about the interaction.

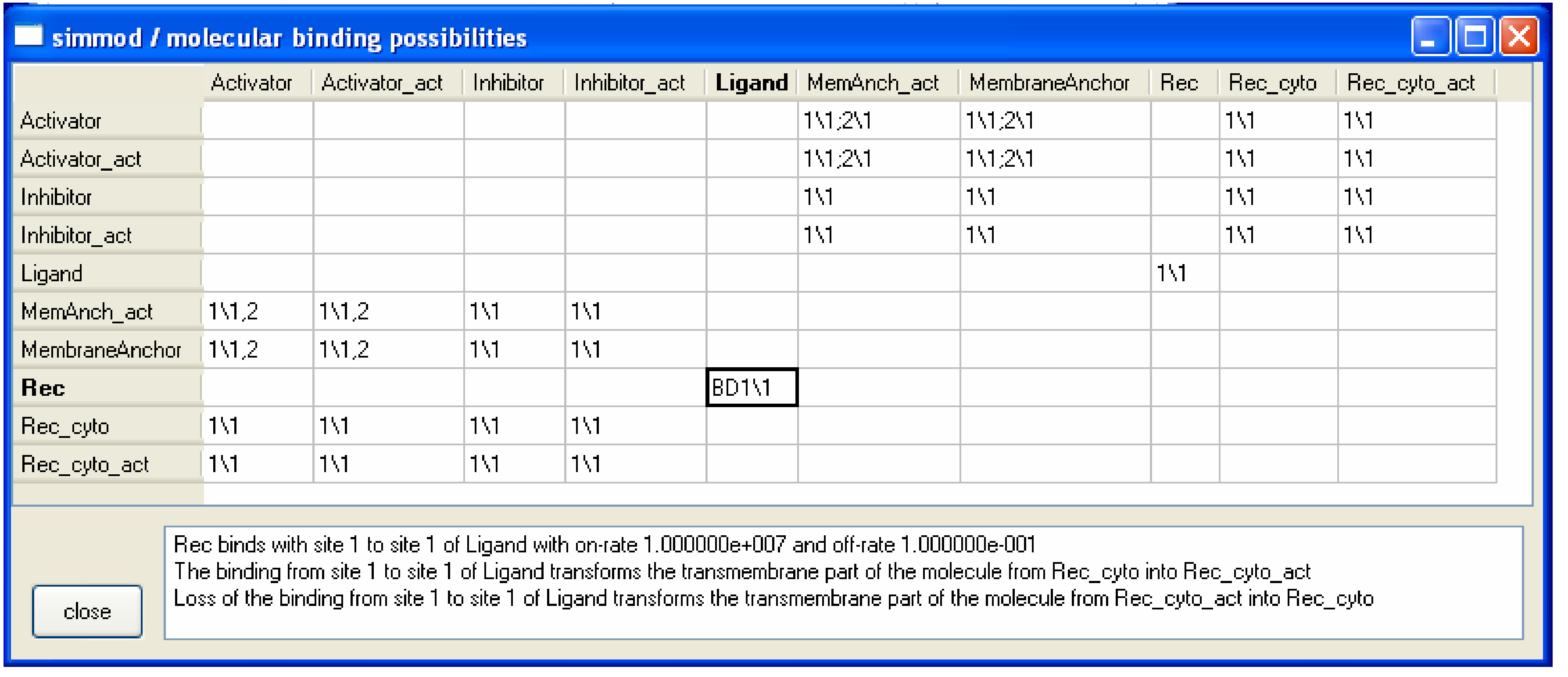

	Activator	Activator_act	Inhibitor	Inhibitor_act	**Ligand**	MemAnch_act	MembraneAnchor	Rec	Rec_cyto	Rec_cyto_act
Activator						1\1;2\1	1\1;2\1		1\1	1\1
Activator_act						1\1;2\1	1\1;2\1		1\1	1\1
Inhibitor						1\1	1\1		1\1	1\1
Inhibitor_act						1\1	1\1		1\1	1\1
Ligand								1\1		
MemAnch_act	1\1,2	1\1,2	1\1	1\1						
MembraneAnchor	1\1,2	1\1,2	1\1	1\1						
**Rec**					BD1\1					
Rec_cyto	1\1	1\1	1\1	1\1						
Rec_cyto_act	1\1	1\1	1\1	1\1						

(34) Now, go to the ‘complexes’ tab.
Drag the ‘Rec’ molecule into the upper middle square of the ‘complex definition’ field.
Then, drag Rec_cyto onto the graphical representation of Rec.

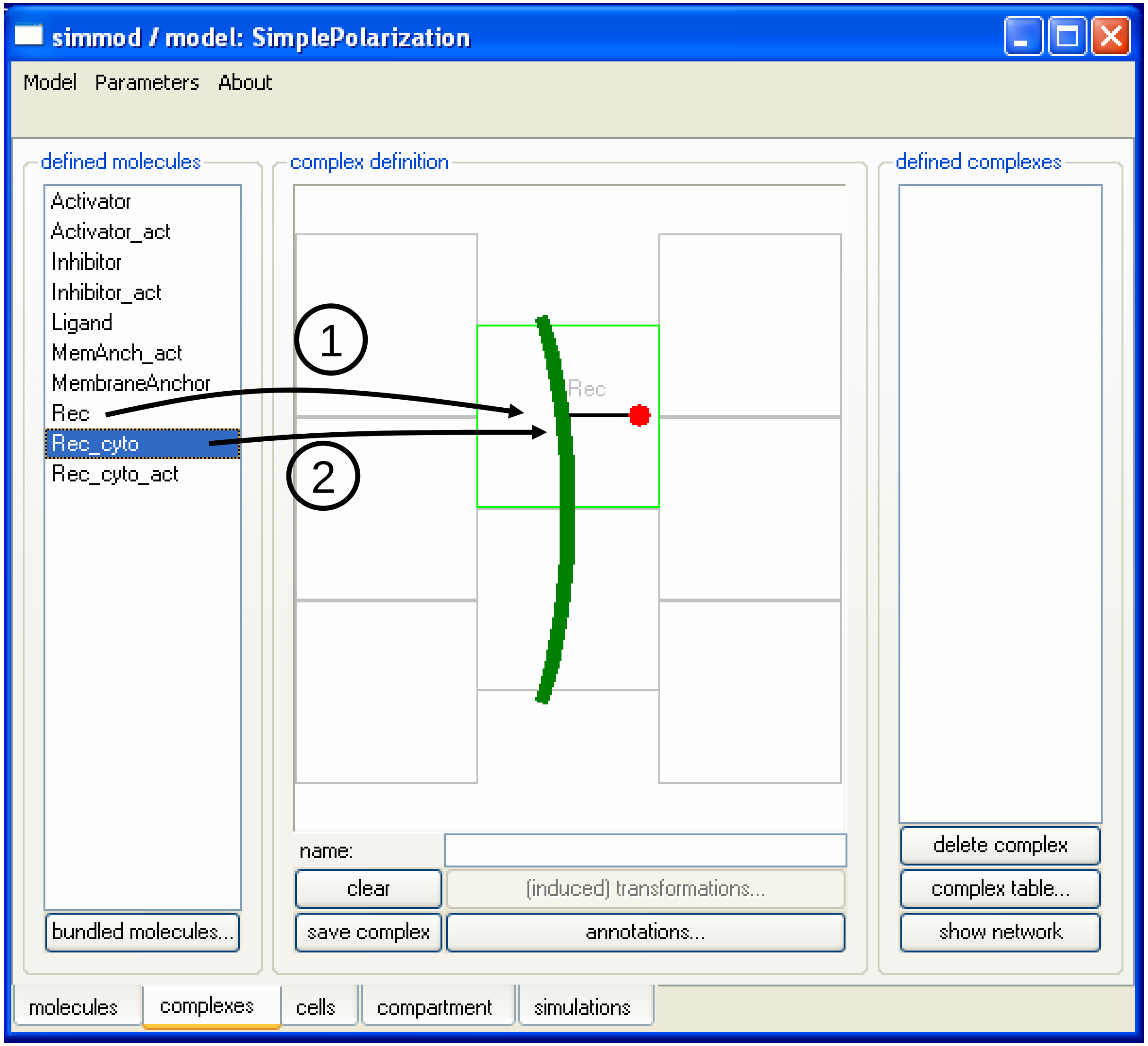

(35) The complete receptor complex should now look like this.
Define ‘Rec’ to be the name of the receptor complex (it is OK for complexes to have the same name as molecules). Save it.

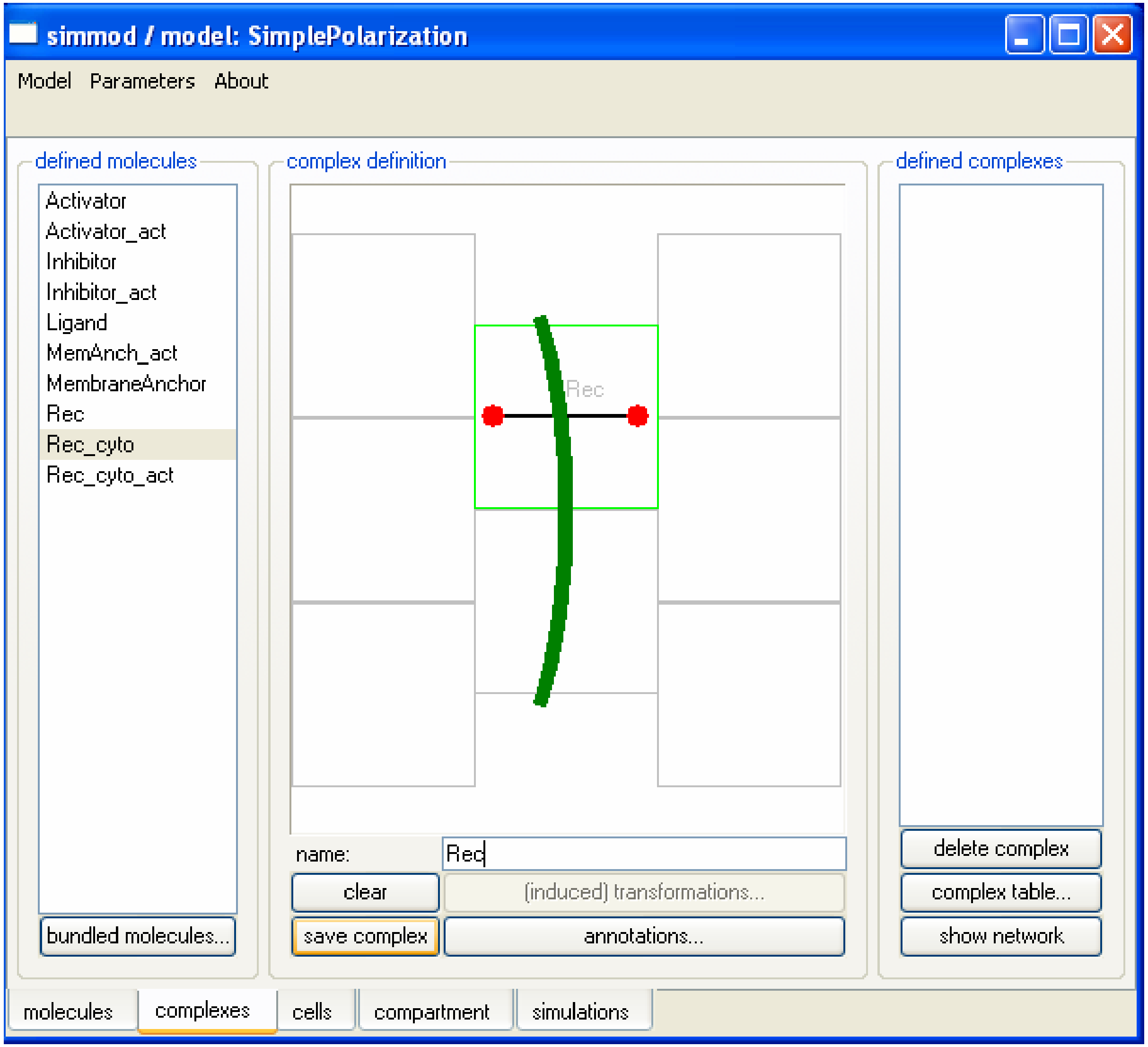

(**36**) Now build a receptor complex with ‘Rec_cyto_act’ as cytosolic domain. Define ‘Rec_act’ to be the name of the receptor complex. Save it.
If you now click on ‘complex table’ you should see the following two complexes:

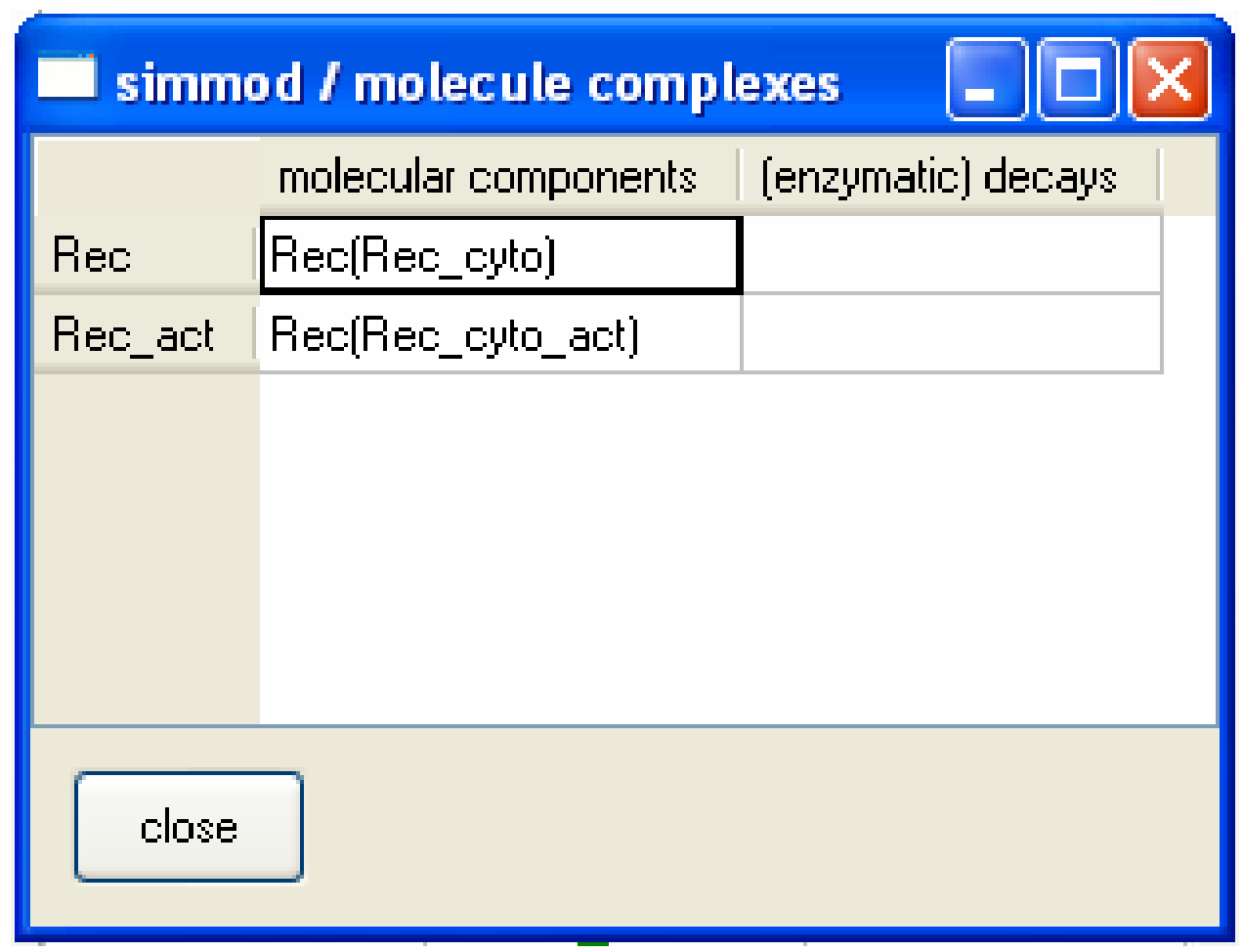

(37) Now, drag and drop the Ligand into the right upper area of the complex definition field already containing the activated receptor complex (with the Rec_cyto_act cytosolic domain). A dotted line will appear, indicating that the Ligand can bind to the receptor. Double-click the small interaction rectangle to close the binding and build the complex of receptor and ligand.

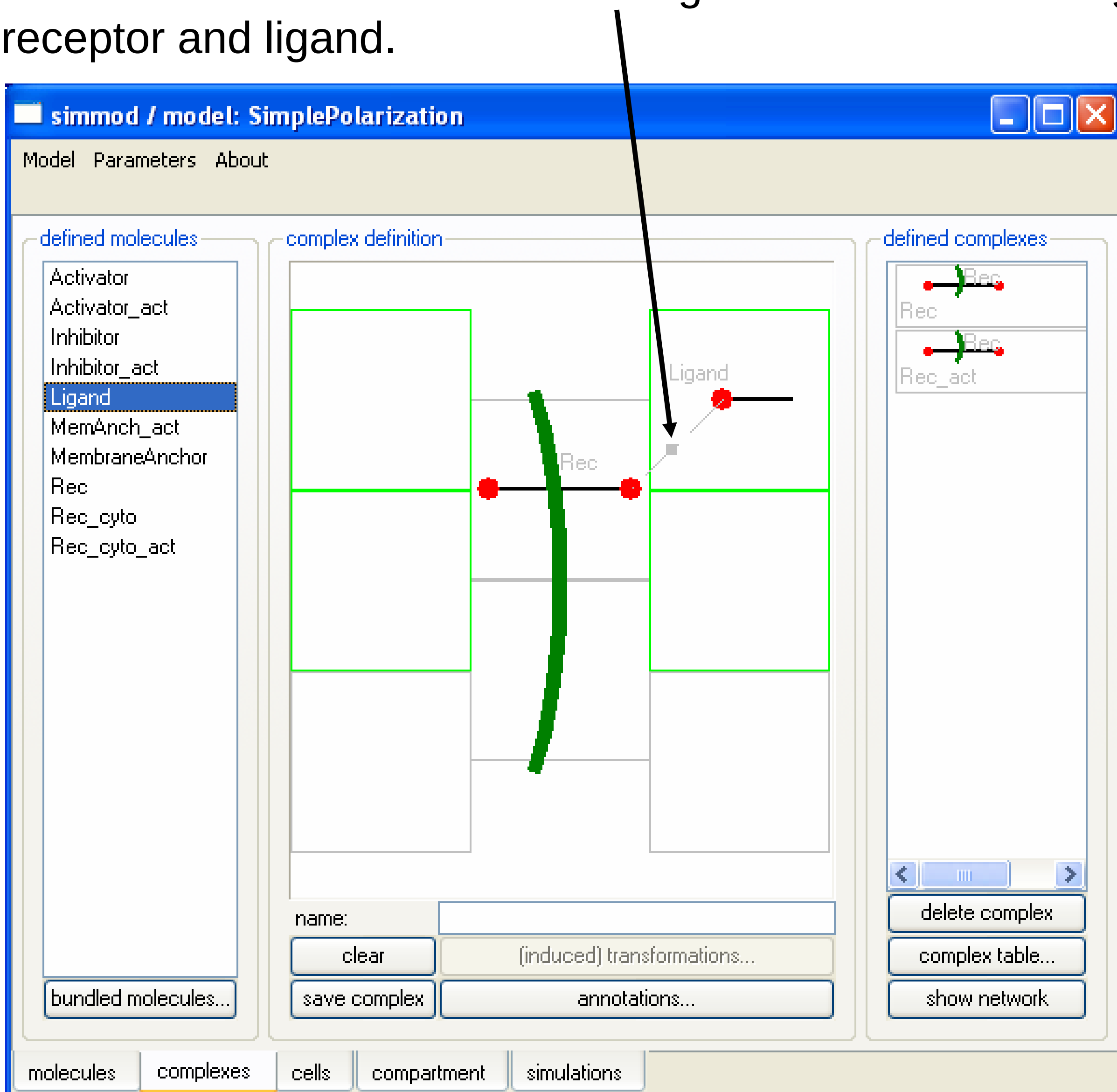

(38) The complex should look like shown below. Assign a name and save the complex. Note, that we could have built a complex with the non-active receptor (Rec_cyto intracellular domain). However: this complex would never be realized during a simulation since binding of the ligand to the receptor activates the receptor and debinding deactivates it.

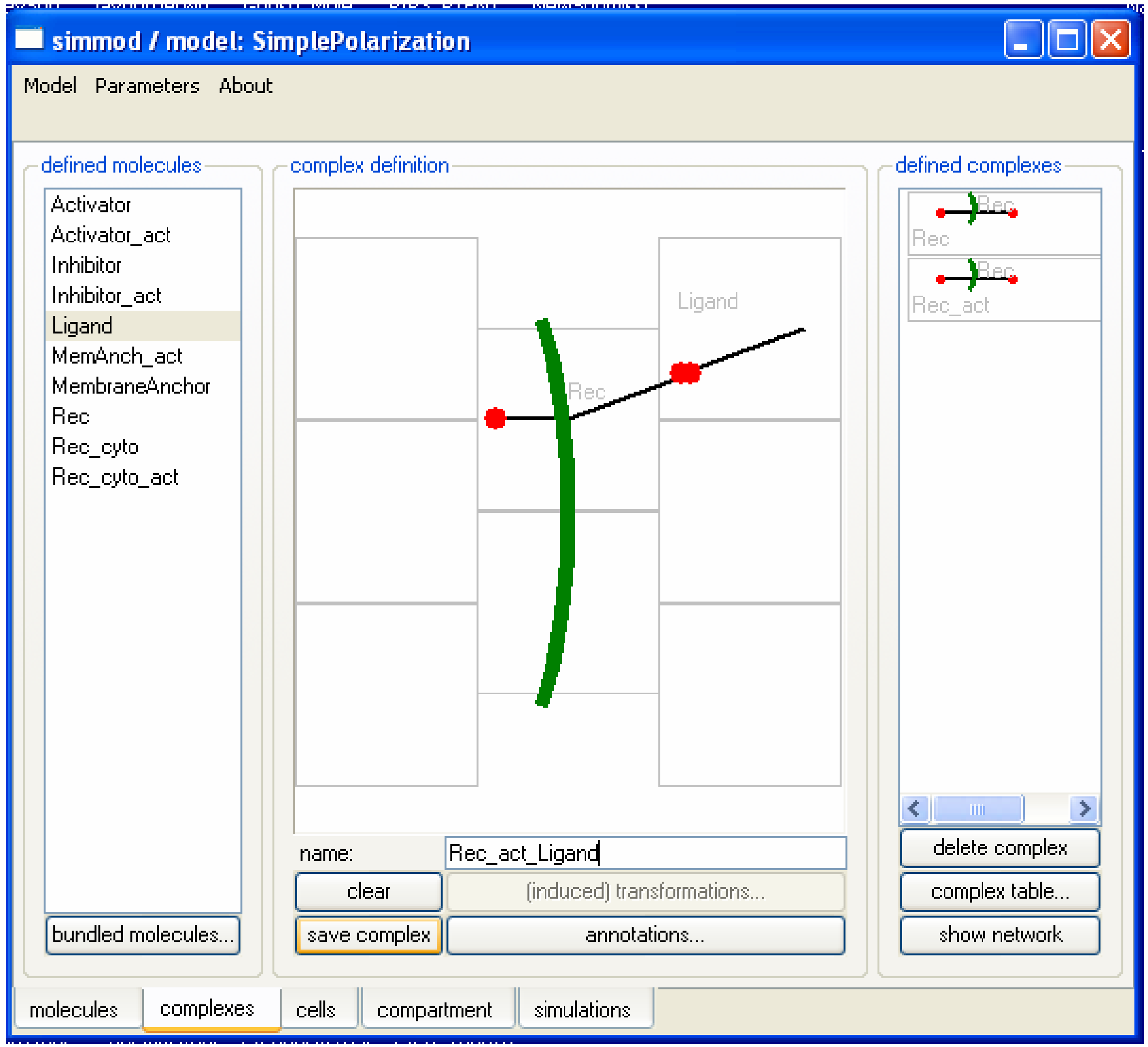

(39) Now build single-molecule complexes as shown below for Activator. Build complexes of Activator_act, Inhibitor, Inhibitor_act, MembraneAnchor, MemAnch_act, Ligand. We only build these complexes because Simmune will during a simulation only provide information about the complexes we define – not the molecules. Note that the software creates all possible complexes automatically. We only define those that we want to track specifically.

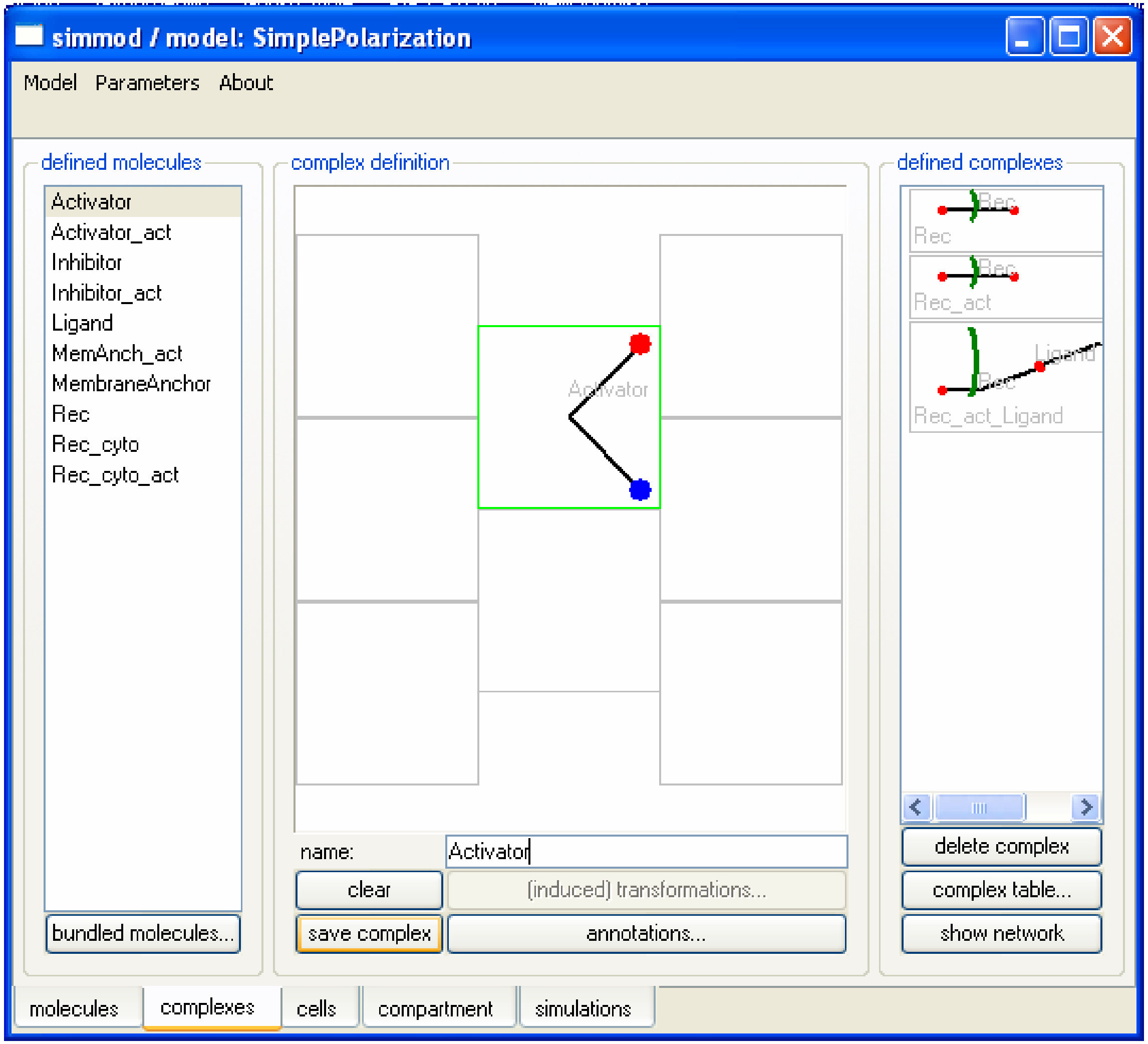

(40) Build a complex of the MemAnch_act and Activator_act:

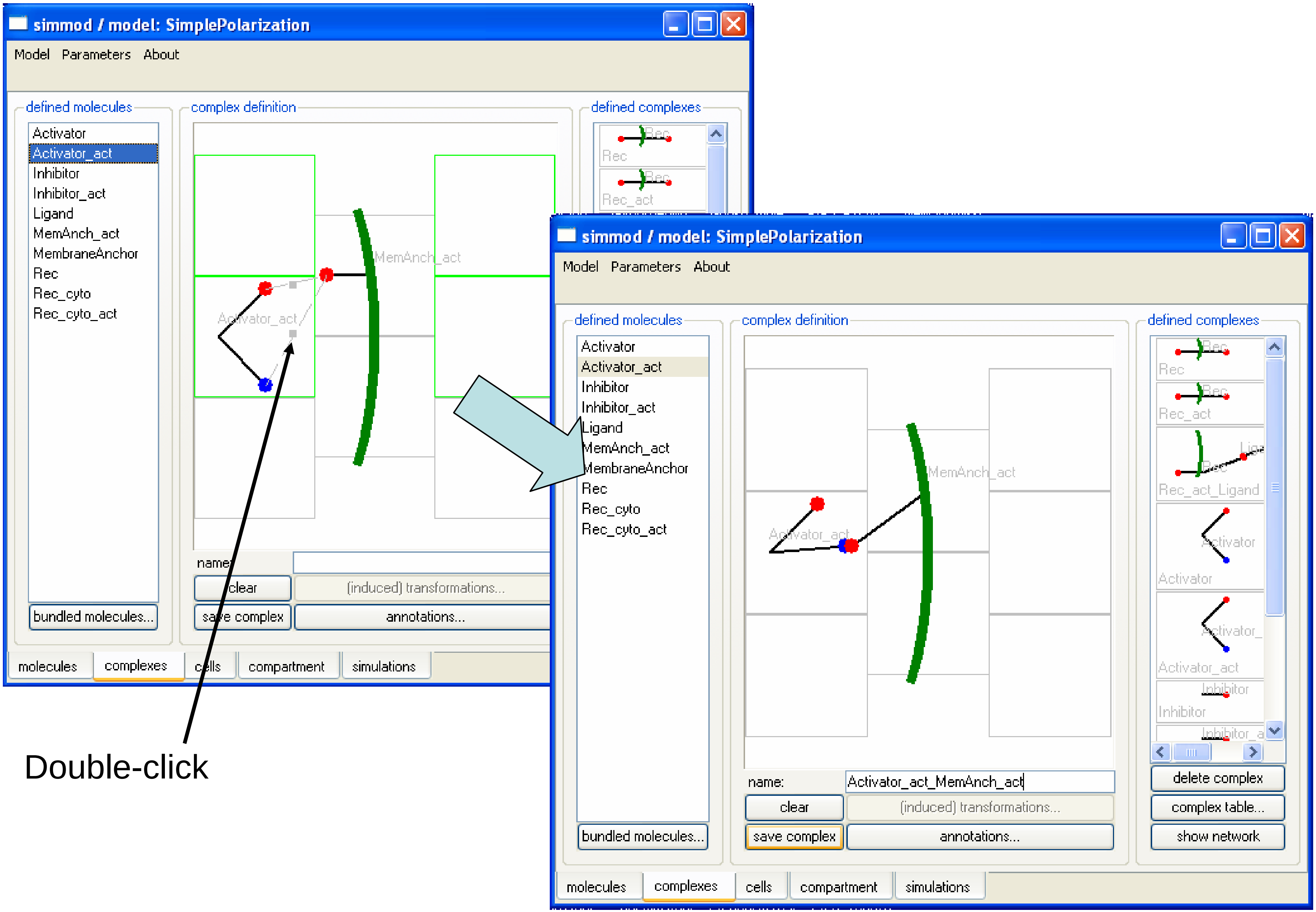

(41) After having built all complexes we want to define a 'molecule bundle' – bundles consist of one or more molecule types. Simmune will be able to inform you about the summed-up concentrations of all complexes each containing all the molecules listed in the definition of the bundle.

Click on 'bundled molecules' in the 'complexes' tab.

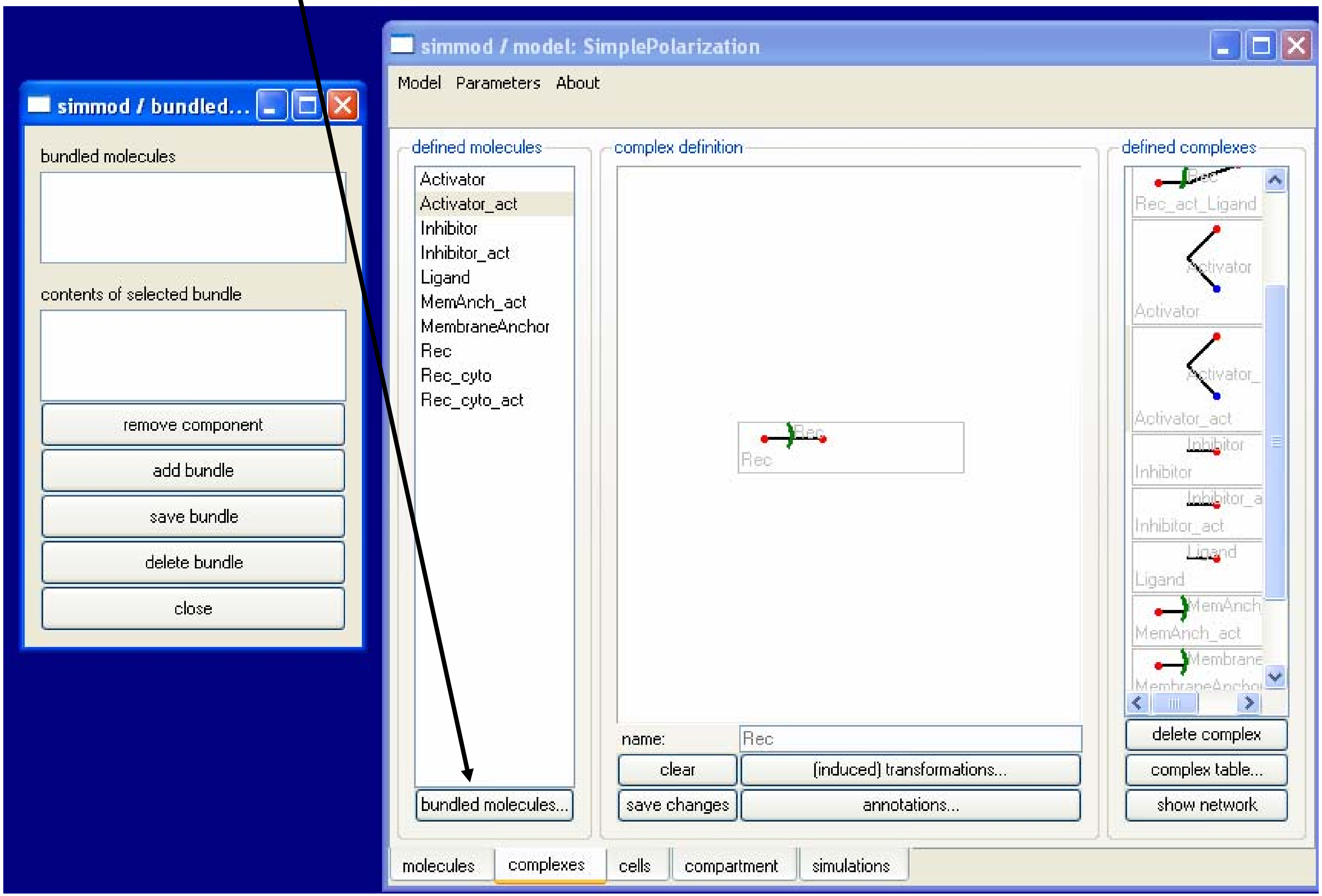

(42) Click on ‘add bundle’ and specify the name ‘All_Activator_act’. We will use this bundle to track all complexes containing the activated Activator.

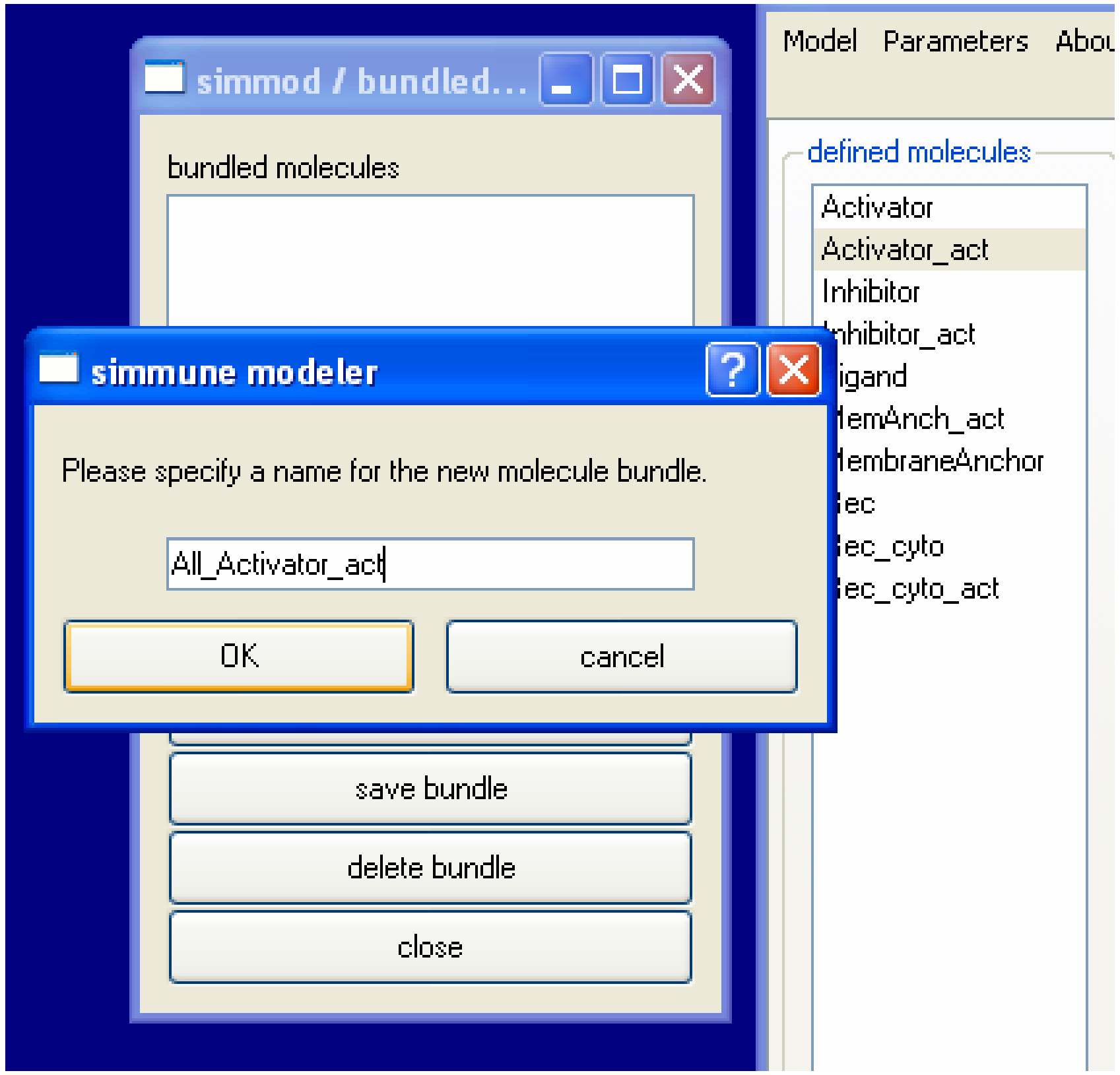

(**43**) Drag and drop the Activator_act molecule into the contents of selected bundle' list. Then, save the bundle

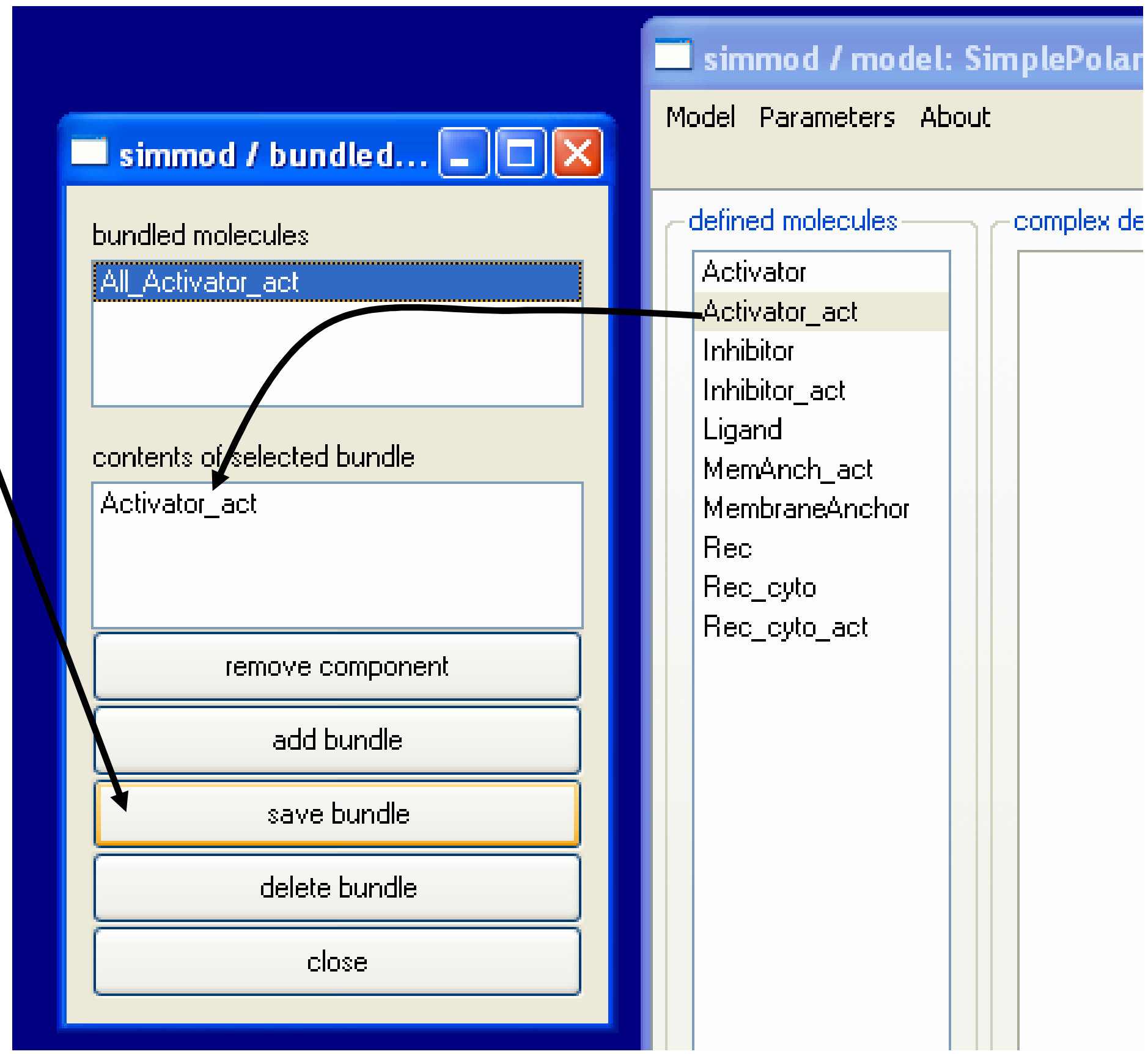

(44) Double-click the Activator complex in the list of 'defined complexes'. Then, click on '(induced) transformations'.

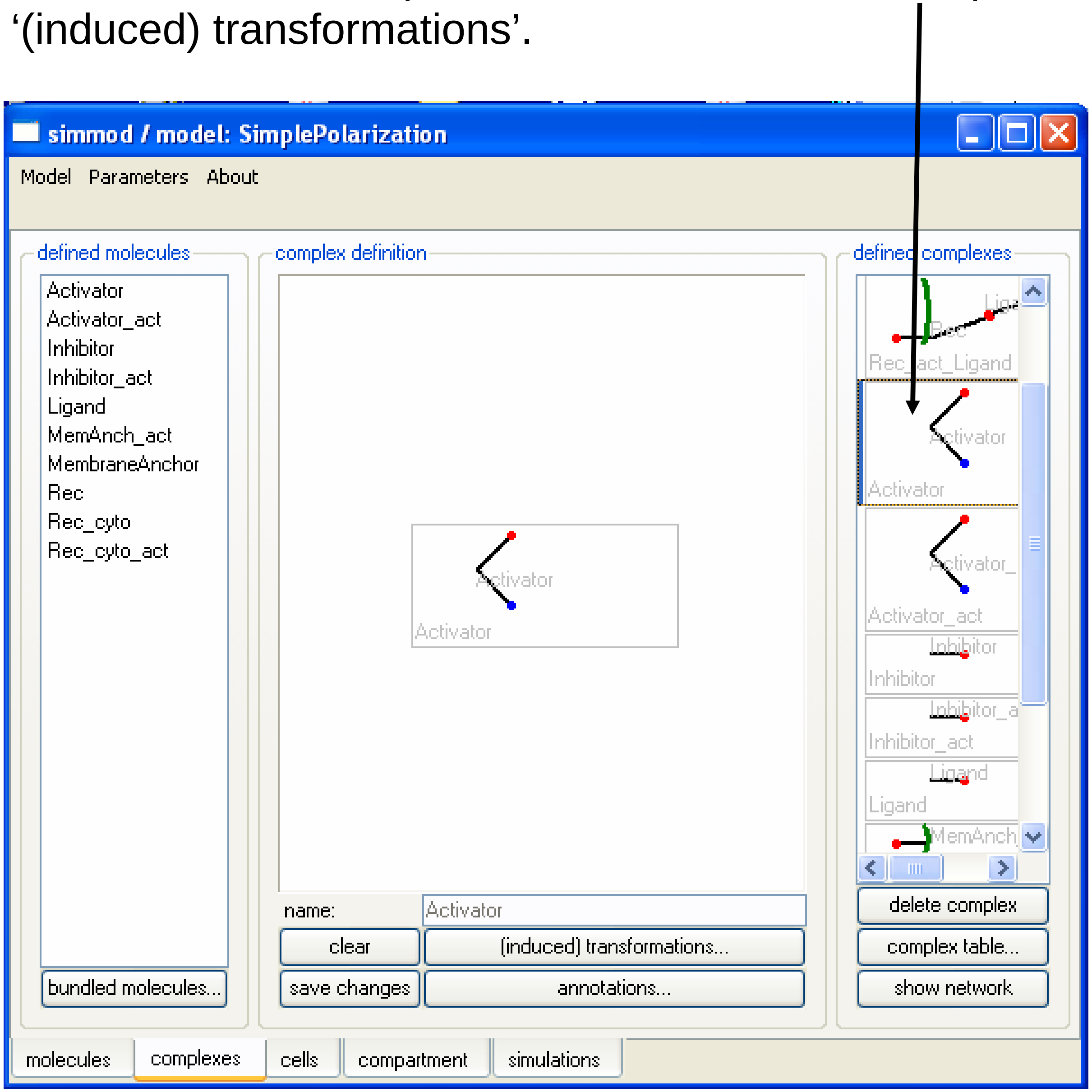

(45) Define a new enzymatic transformation. Let the enzyme ‘Rec_cyto_act’ transform the complex into the product Activator_act. ‘Independent transitions’ means that the enzyme (Rec_cyto_act) will act on the substrate molecule Activator whatever its binding state is – for example also when it is bound to the MemAnch_act.
Click OK and save the changes to Activator.

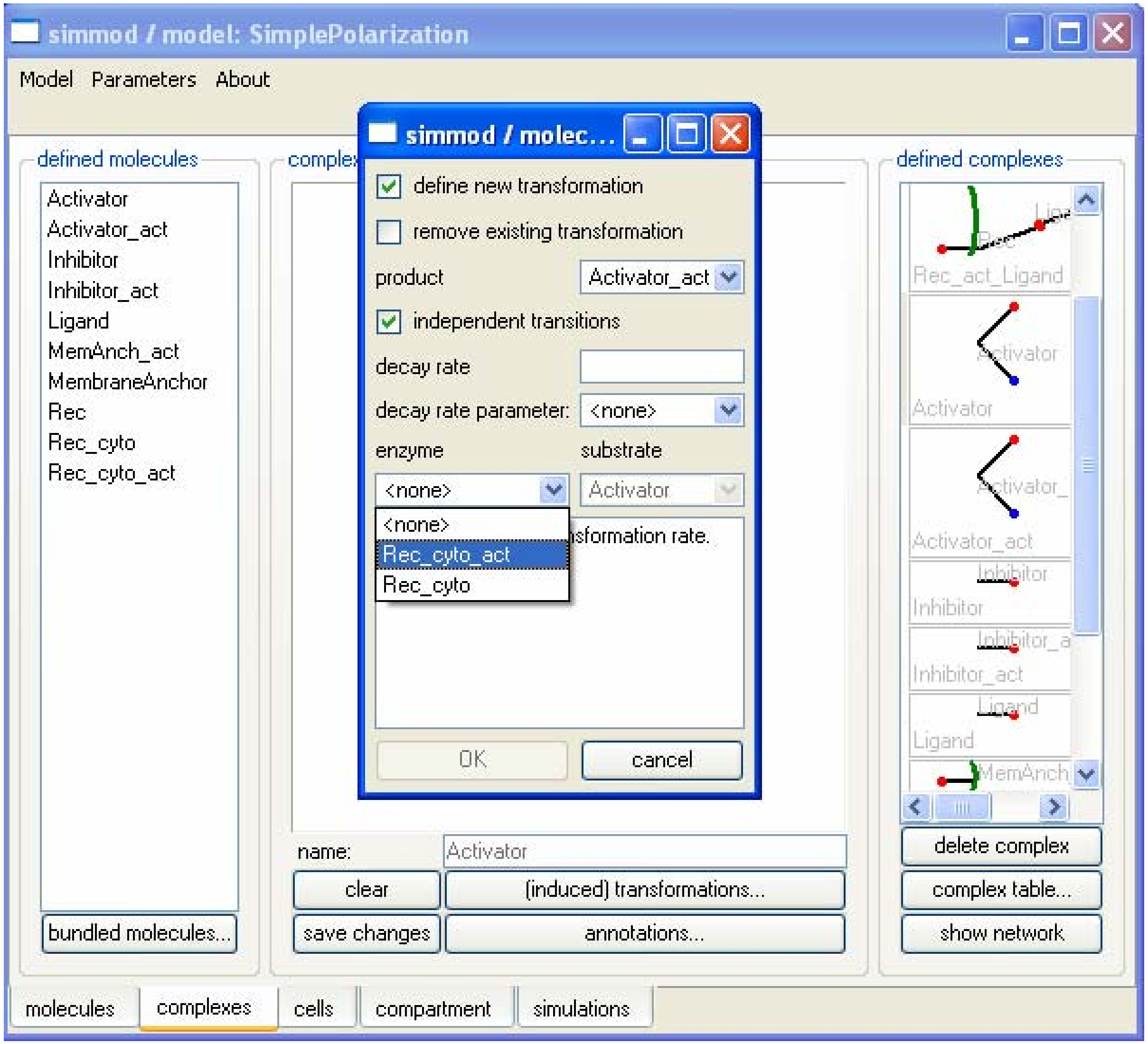

(**46**) If you now click on ‘complex table’ you should see the following:
As mentioned before, the kinetic parameters of the enzymatic transformation are provided by the interaction rates between enzyme, substrate and product.

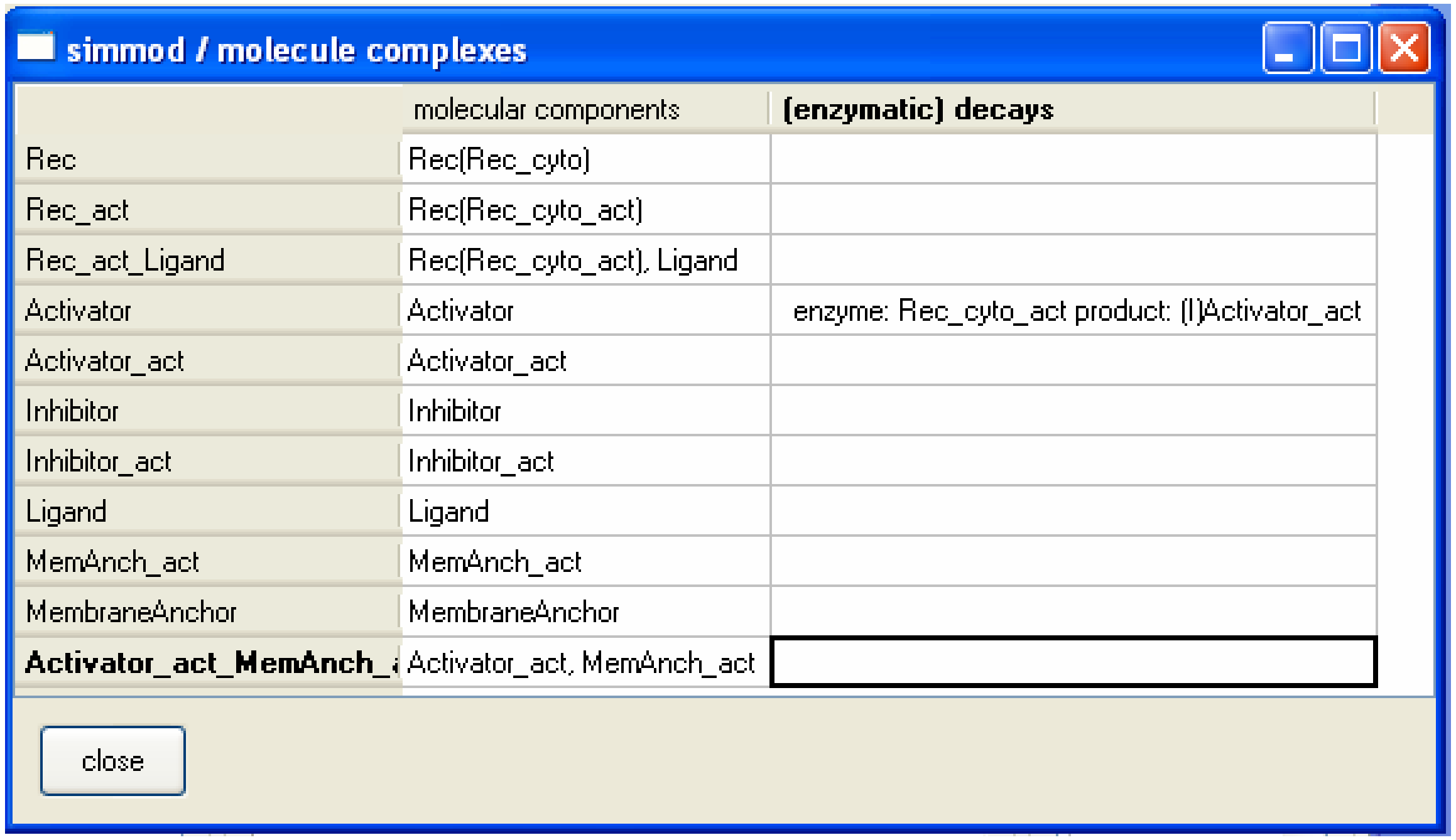

	molecular components	(enzymatic) decays
Rec	Rec(Rec_cyto)	
Rec_act	Rec(Rec_cyto_act)	
Rec_act_Ligand	Rec(Rec_cyto_act), Ligand	
Activator	Activator	enzyme: Rec_cyto_act product: (I)Activator_act
Activator_act	Activator_act	
Inhibitor	Inhibitor	
Inhibitor_act	Inhibitor_act	
Ligand	Ligand	
MemAnch_act	MemAnch_act	
MembraneAnchor	MembraneAnchor	
**Activator_act_MemAnch_**	Activator_act, MemAnch_act	

(47) Now, define the following enzymatic transformations:

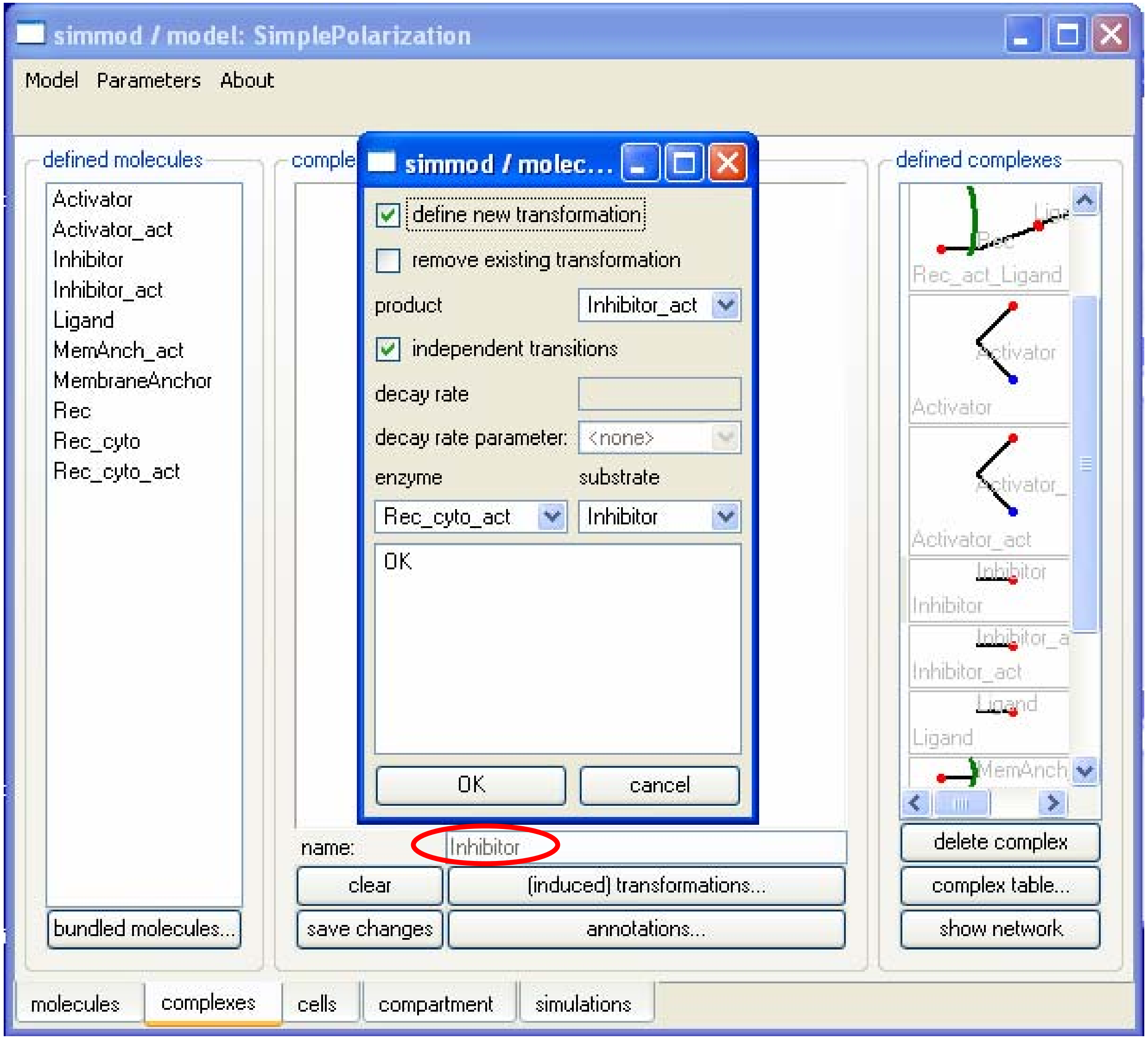

(48)

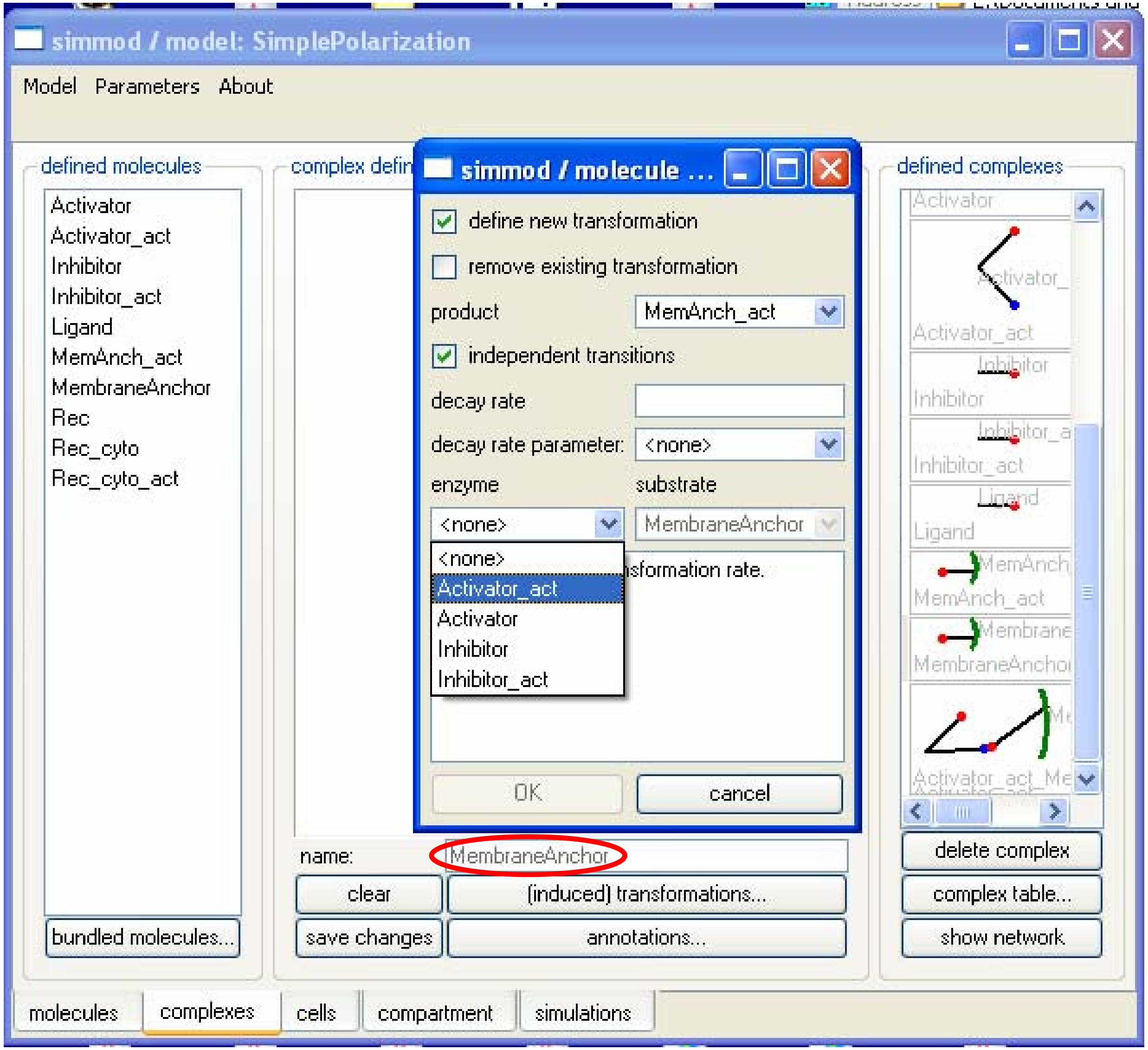

simmod / model: SimplePolarization
Model
Parameters
About
defined molecules
Activator
Activator_act
Inhibitor
Inhibitor_act
Ligand
MemAnch_act
MembraneAnchor
Rec
Rec_cyto
Rec_cyto_act
bundled molecules...
complex defin
simmod / molecule ...
define new transformation
remove existing transformation
product
MemAnch_act
independent transitions
decay rate
decay rate parameter:
<none>
enzyme
substrate
<none>
MembraneAnchor
<none>
Activator_act
Activator
Inhibitor
Inhibitor_act
sformation rate.
OK
cancel
name:
MembraneAnchor
clear
(induced) transformations...
save changes
annotations...
defined complexes
Activator
Activator_act
Inhibitor
Inhibitor_act
Ligand
MemAnch_act
MembraneAnchor
delete complex
complex table...
show network
molecules
complexes
cells
compartment
simulations

(49)

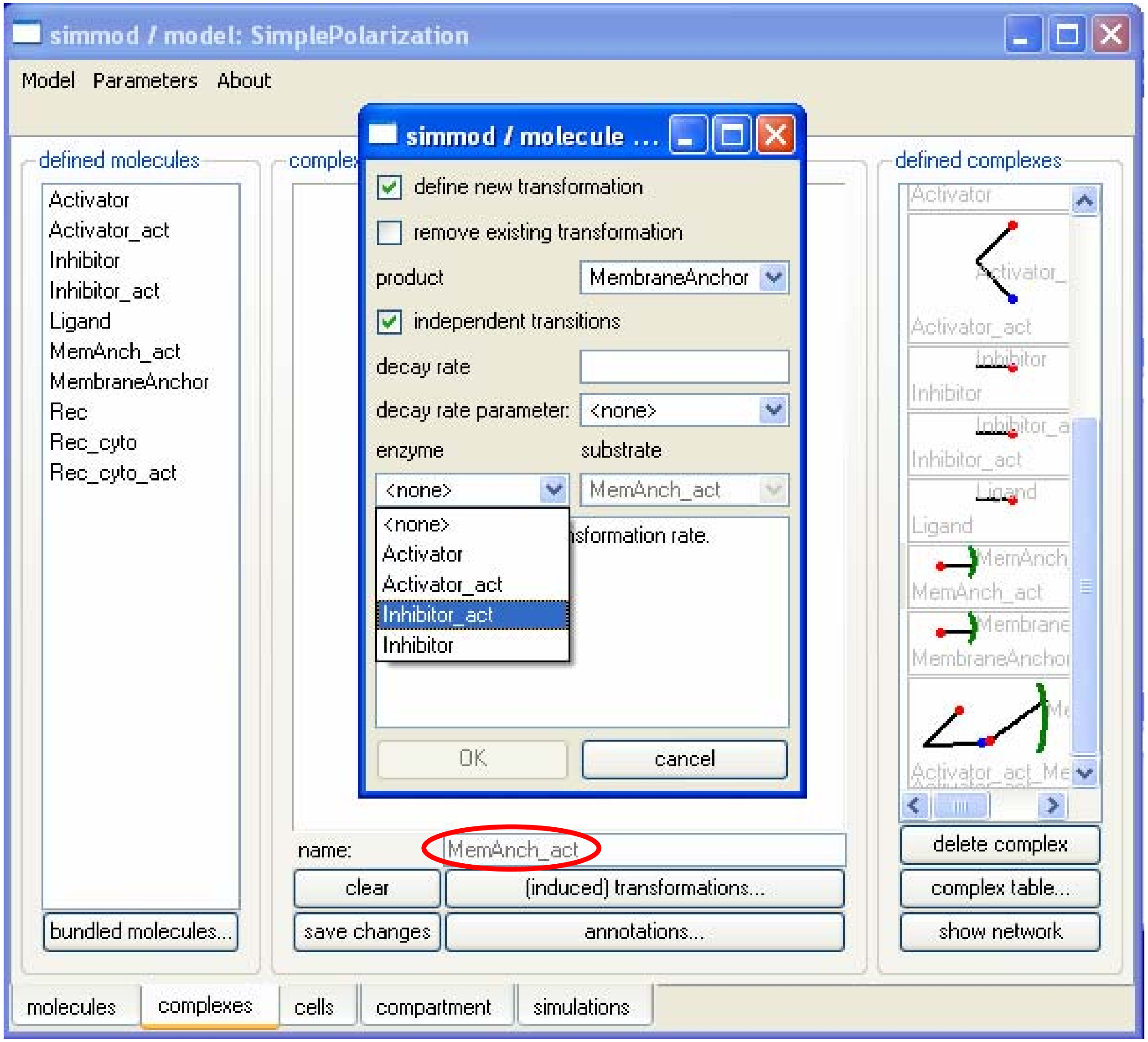
simmod / model: SimplePolarization
Model
Parameters
About
defined molecules
Activator
Activator_act
Inhibitor
Inhibitor_act
Ligand
MemAnch_act
MembraneAnchor
Rec
Rec_cyto
Rec_cyto_act
bundled molecules...
simmod / molecule ...
define new transformation
remove existing transformation
product
MembraneAnchor
independent transitions
decay rate
decay rate parameter:
<none>
enzyme
substrate
<none>
MemAnch_act
<none>
Activator
Activator_act
Inhibitor_act
Inhibitor
sformation rate.
OK
cancel
name:
MemAnch_act
clear
(induced) transformations...
save changes
annotations...
defined complexes
Activator
Activator_act
Inhibitor
Inhibitor_act
Ligand
MemAnch_act
MembraneAnchor
delete complex
complex table...
show network
molecules
complexes
cells
compartment
simulations

(50) Here, we use a spontaneous transformation that deactivates the Inhibitor_act.

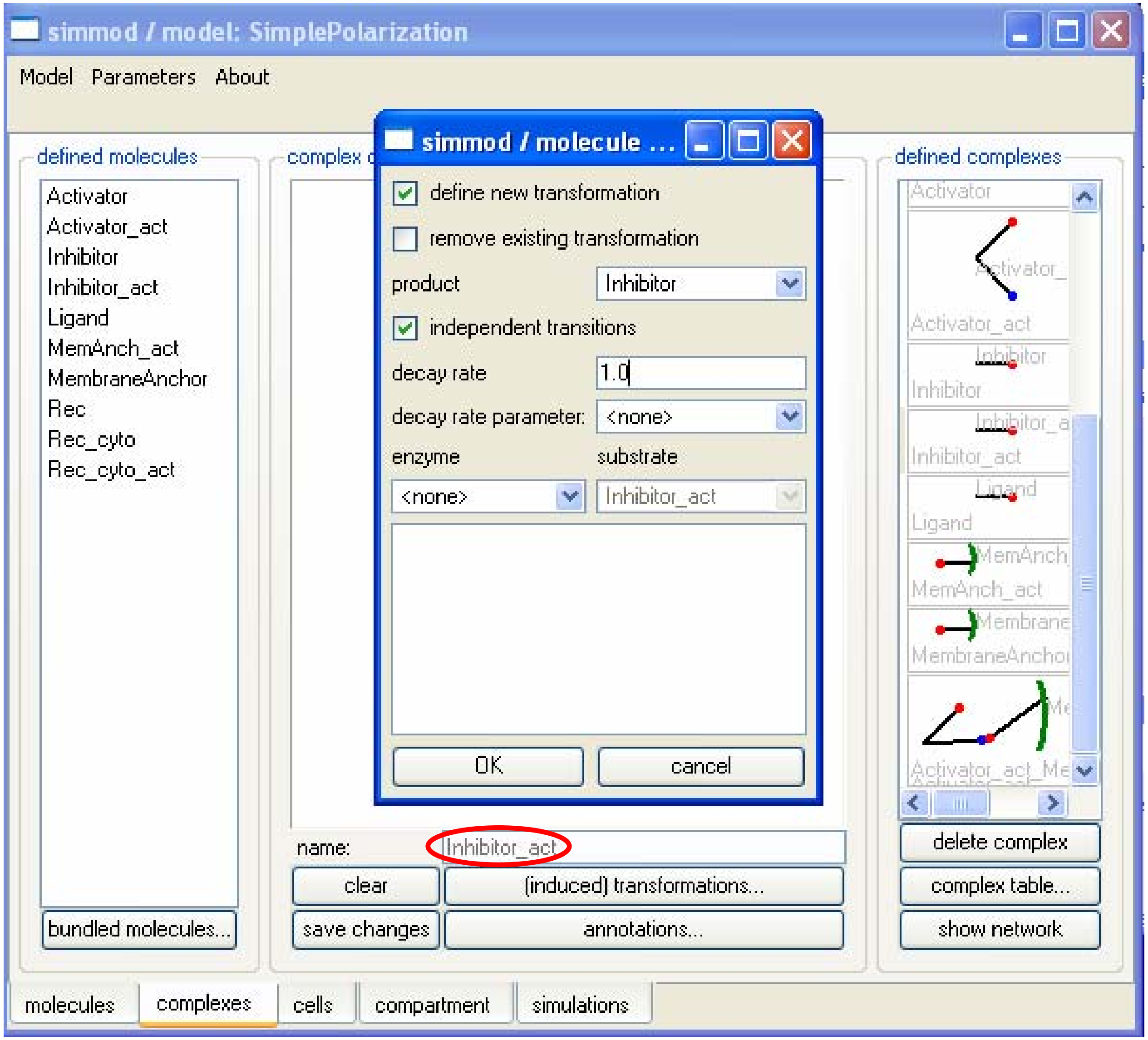

(51) Here, we use a spontaneous transformation that deactivates the Activator_act.

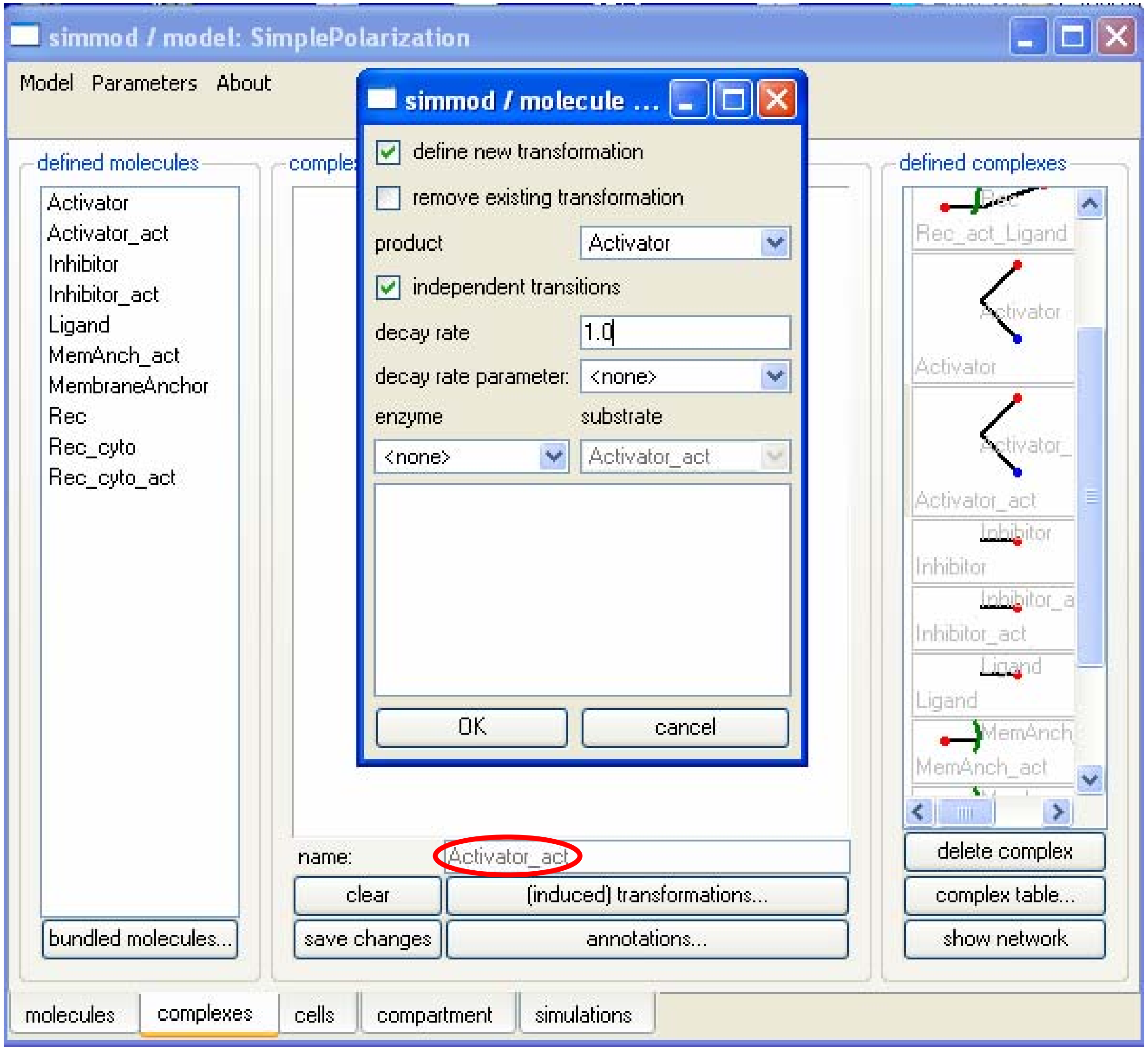

(**52**) Inspect the complex table:

simmod / molecule complexes

	molecular components	(enzymatic) decays
**Rec**	Rec(Rec_cyto)	
Rec_act	Rec(Rec_cyto_act)	
Rec_act_Ligand	Rec(Rec_cyto_act), Ligand	
Activator	Activator	enzyme: Rec_cyto_act product: (I)Activator_act
Activator_act	Activator_act	(I) decay rate: 1.0 product: Activator
Inhibitor	Inhibitor	enzyme: Rec_cyto_act product: (I)Inhibitor_act
Inhibitor_act	Inhibitor_act	(I) decay rate: 1.0 product: Inhibitor
Ligand	Ligand	
MemAnch_act	MemAnch_act	enzyme: Inhibitor_act product: (I)MembraneAnchor
MembraneAnchor	MembraneAnchor	enzyme: Activator_act product: (I)MemAnch_act
Activator_act_MemAnch_act	Activator_act, MemAnch_act	

close

The activated receptor activates Activator and Inhibitor. Activator_act activates the MembraneAnchor. MemAnch_act provides binding possibilities for the Activator and the Activator_act. This works as a positive feedback in two ways: MemAnch_act brings Activator to the membrane and hence close to the receptor for more efficient activation. (2) Activator_act when it is bound to MemAnch_act is close to other MembraneAnchors it can then activate efficiently. Inhibitor simply deactivates MemAnch_act without this localization feedback.

(53) Go to the ‘cells’ tab and click ‘new cell type’.

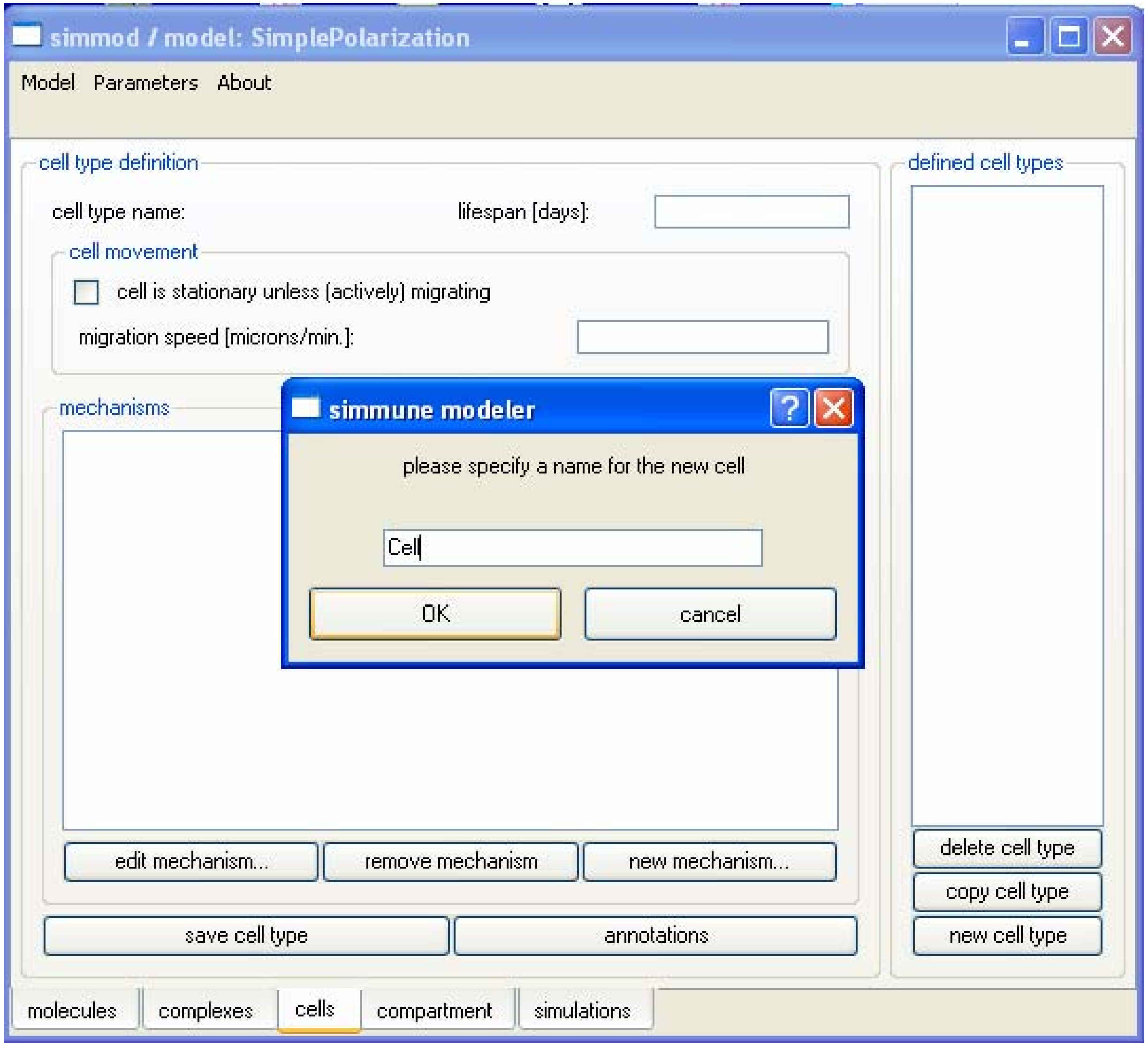

(54) Double-click Cell in the list of defined cell types to edit its properties. Then click on ‘new mechanism’ (see documentation for details on cellular mechanisms).

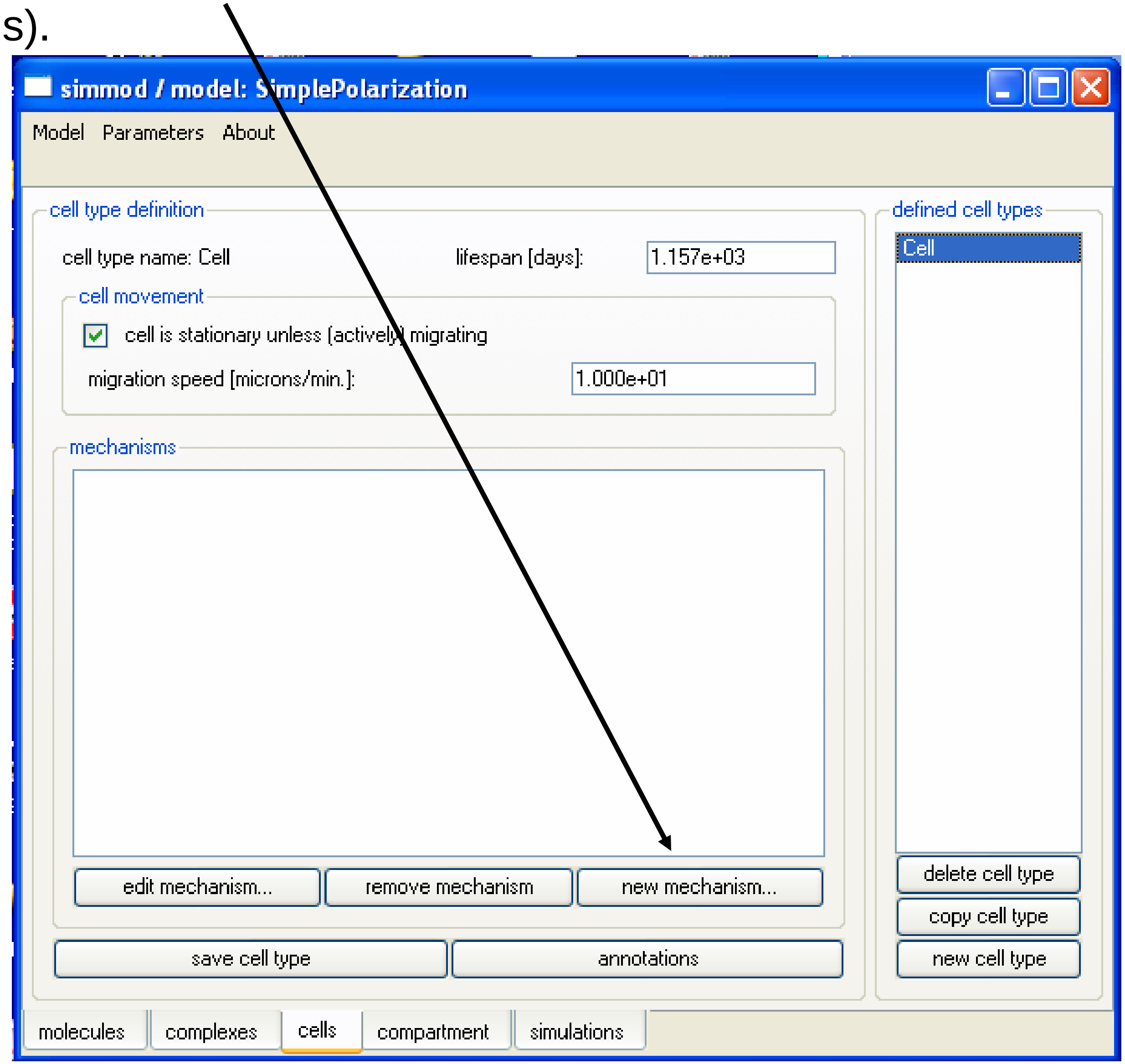

(55) Click ‘add condition’ and then click on the ‘if/if not’ section of the header. Make sure that the line containing the entry is selected (highlighted).

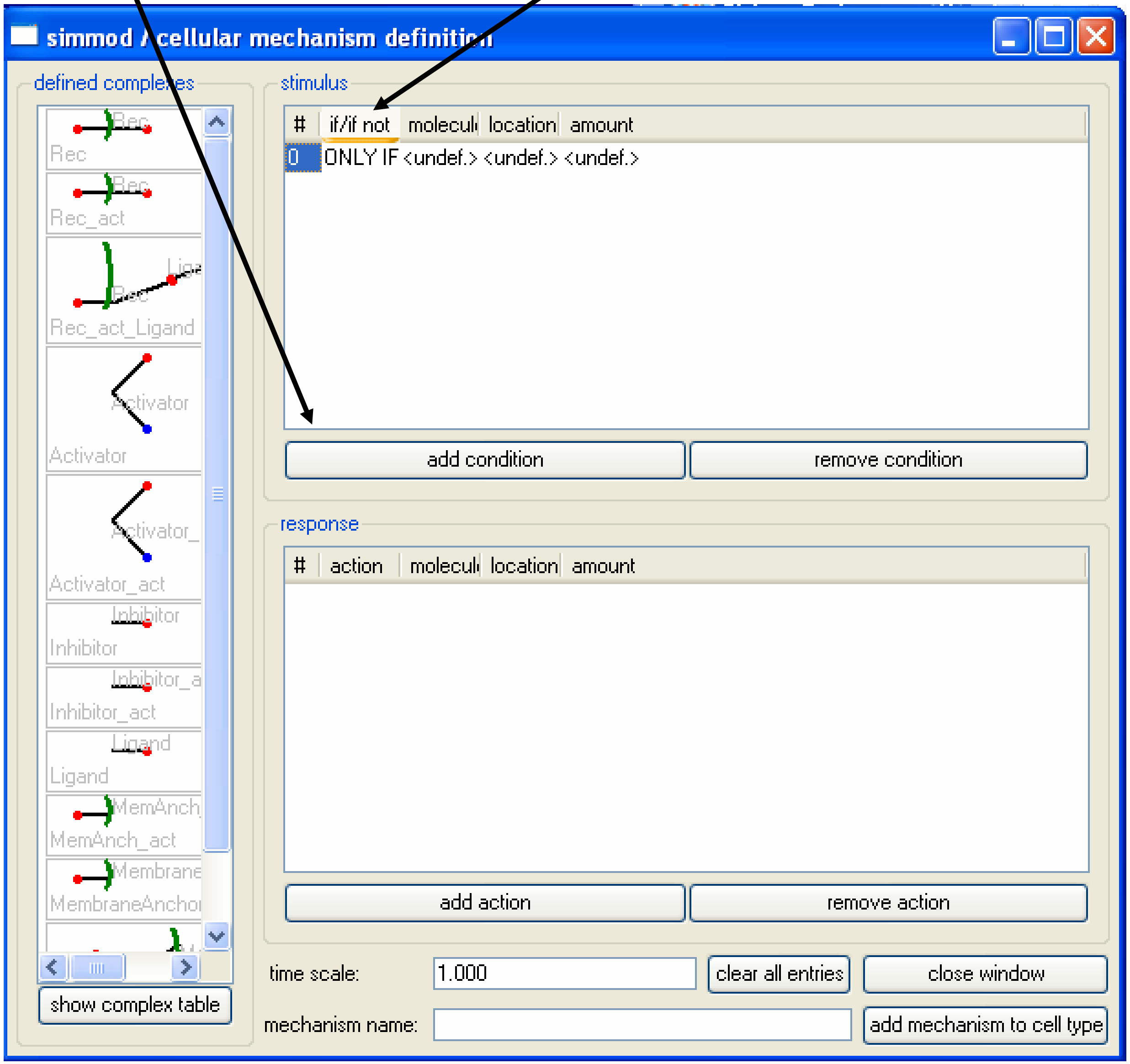

(56) Click OK in the message window below.
The ‘stimulus’ list should now look like this:

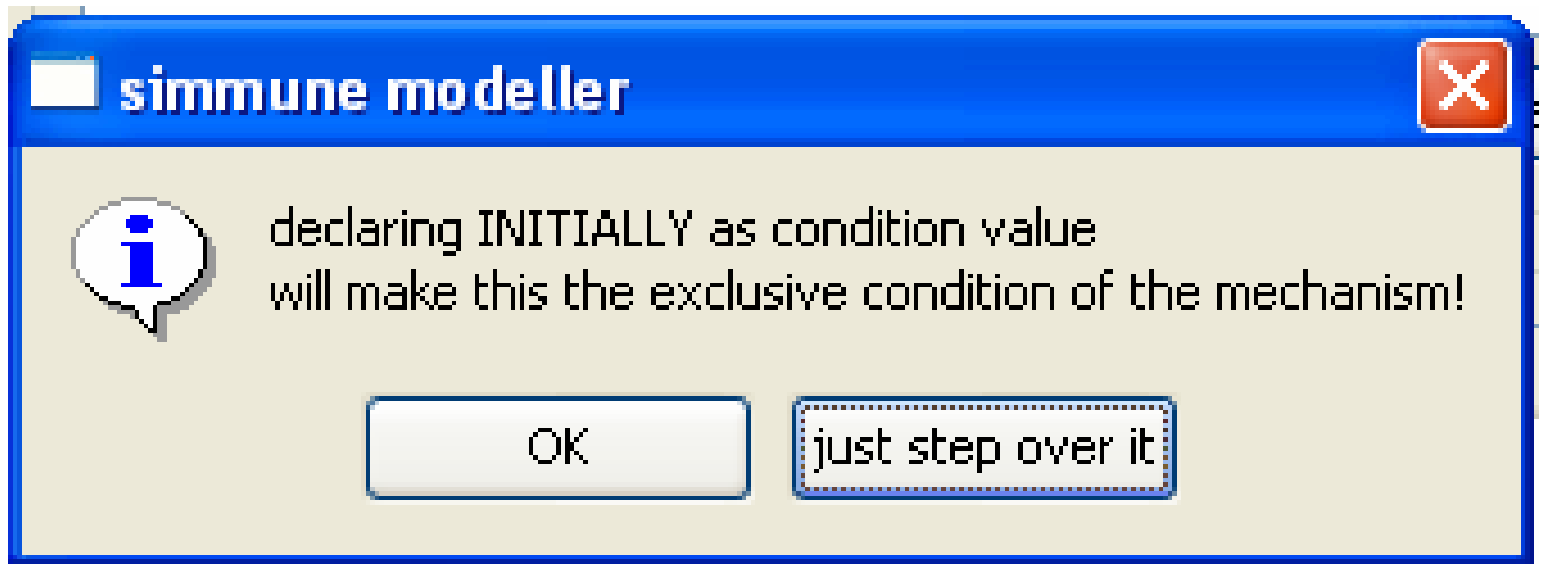

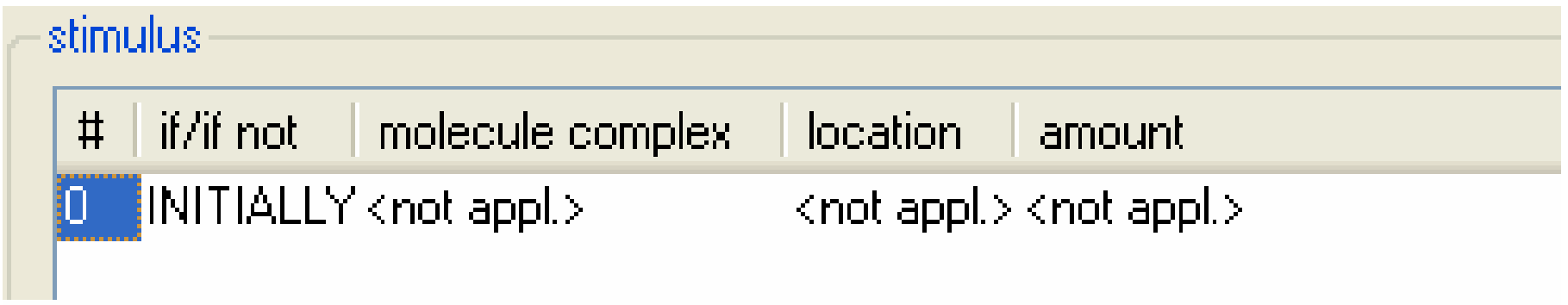

(57) Click ‘add action’ and then the ‘action’ section of the response list header. Make sure that the line containing the entry is selected (highlighted).

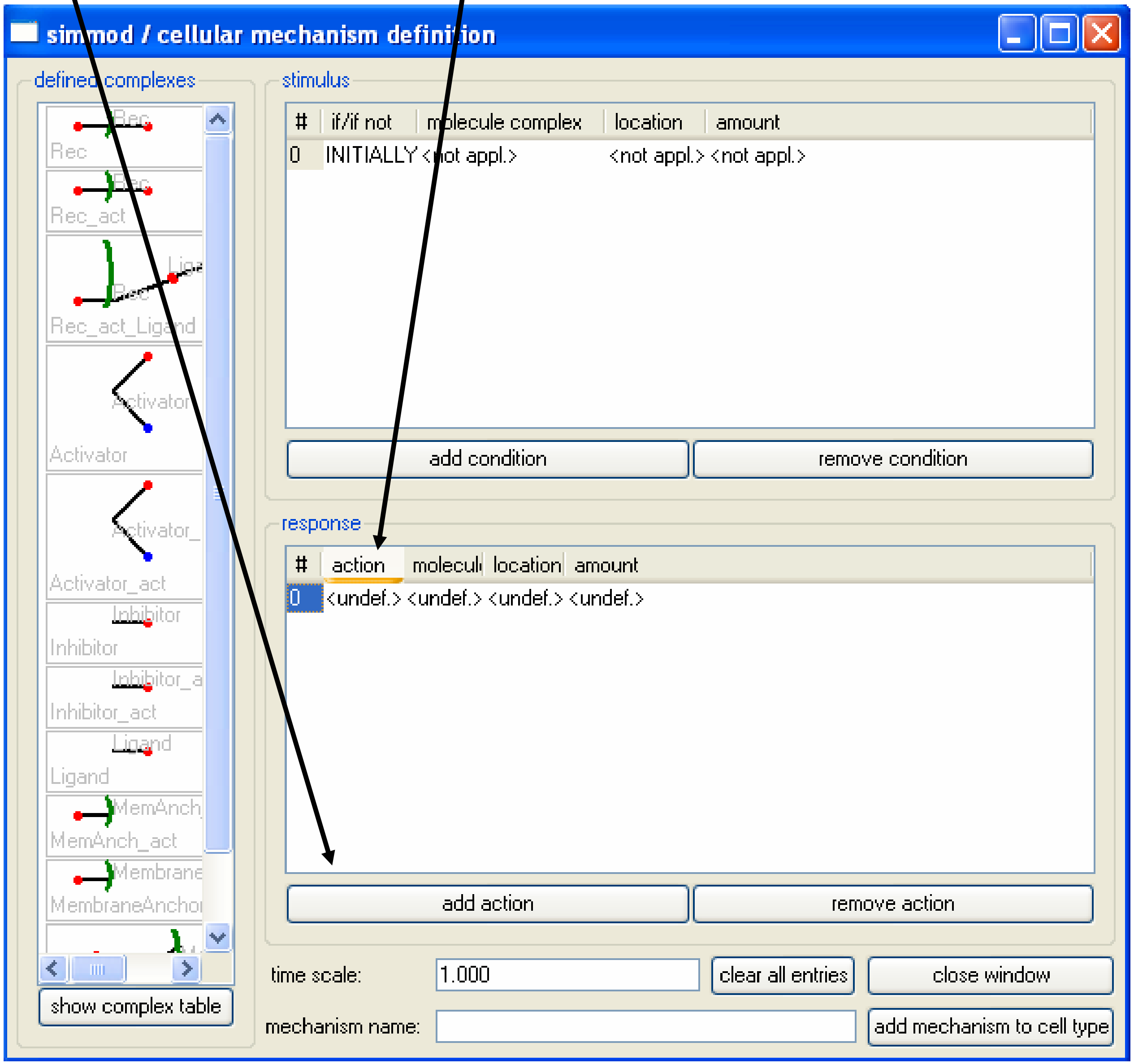

(58) Select as action ‘display membrane molecule complex’.

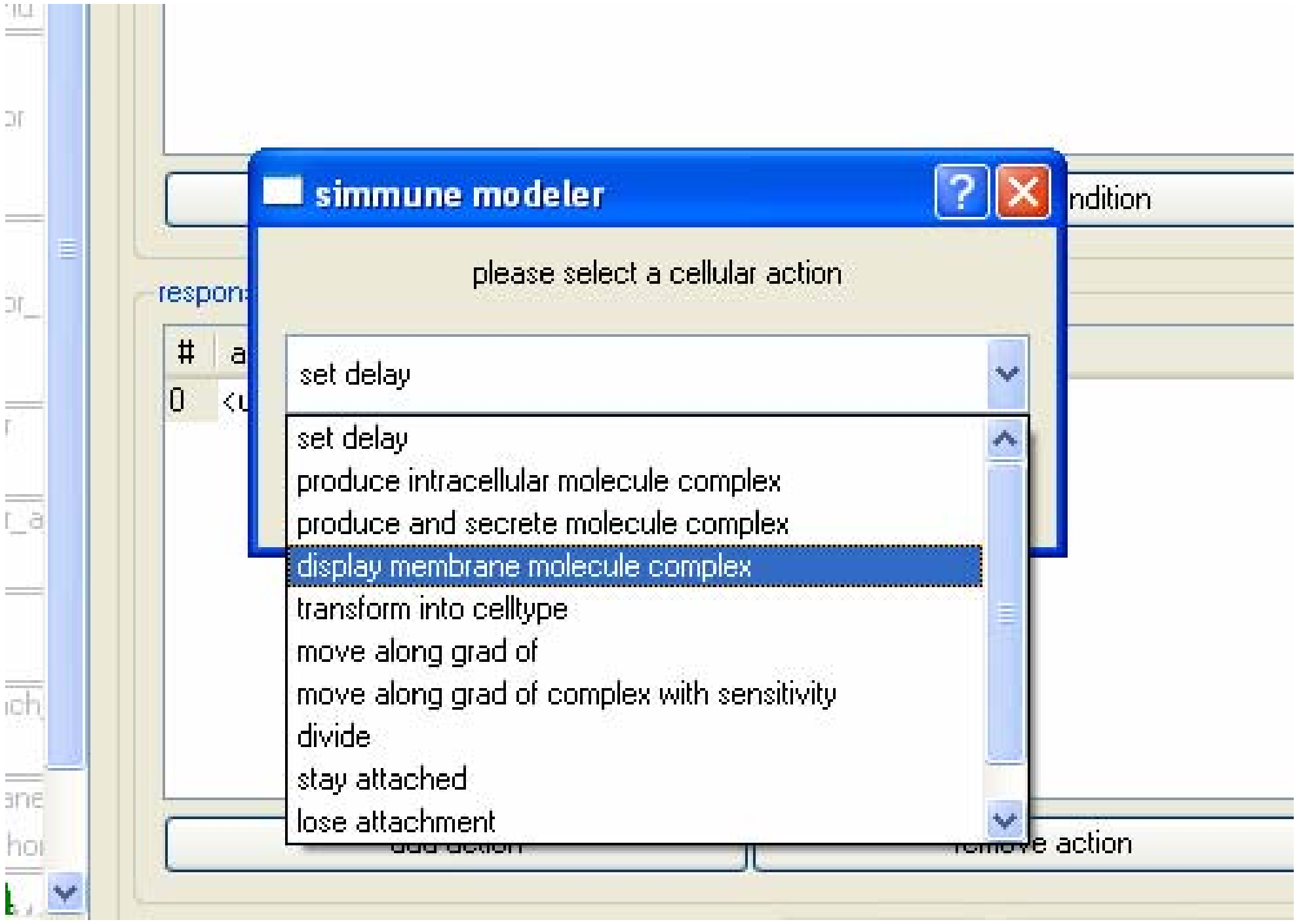

(59) Drag the ‘Rec’ complex into the selected action.

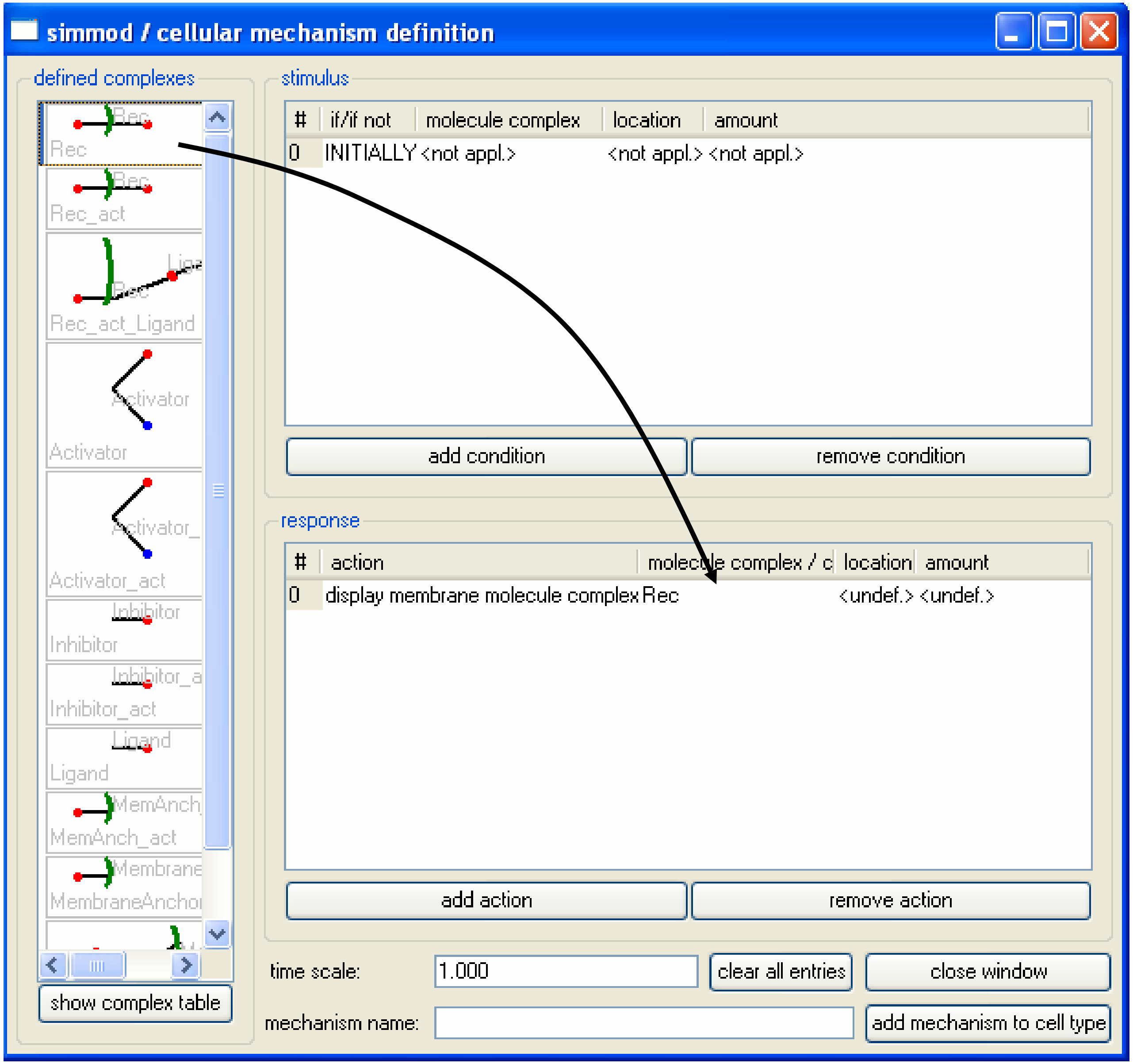

(60) Repeatedly click the 'location' section (with activated list entry) until the location says 'EVERY_SIDE'.

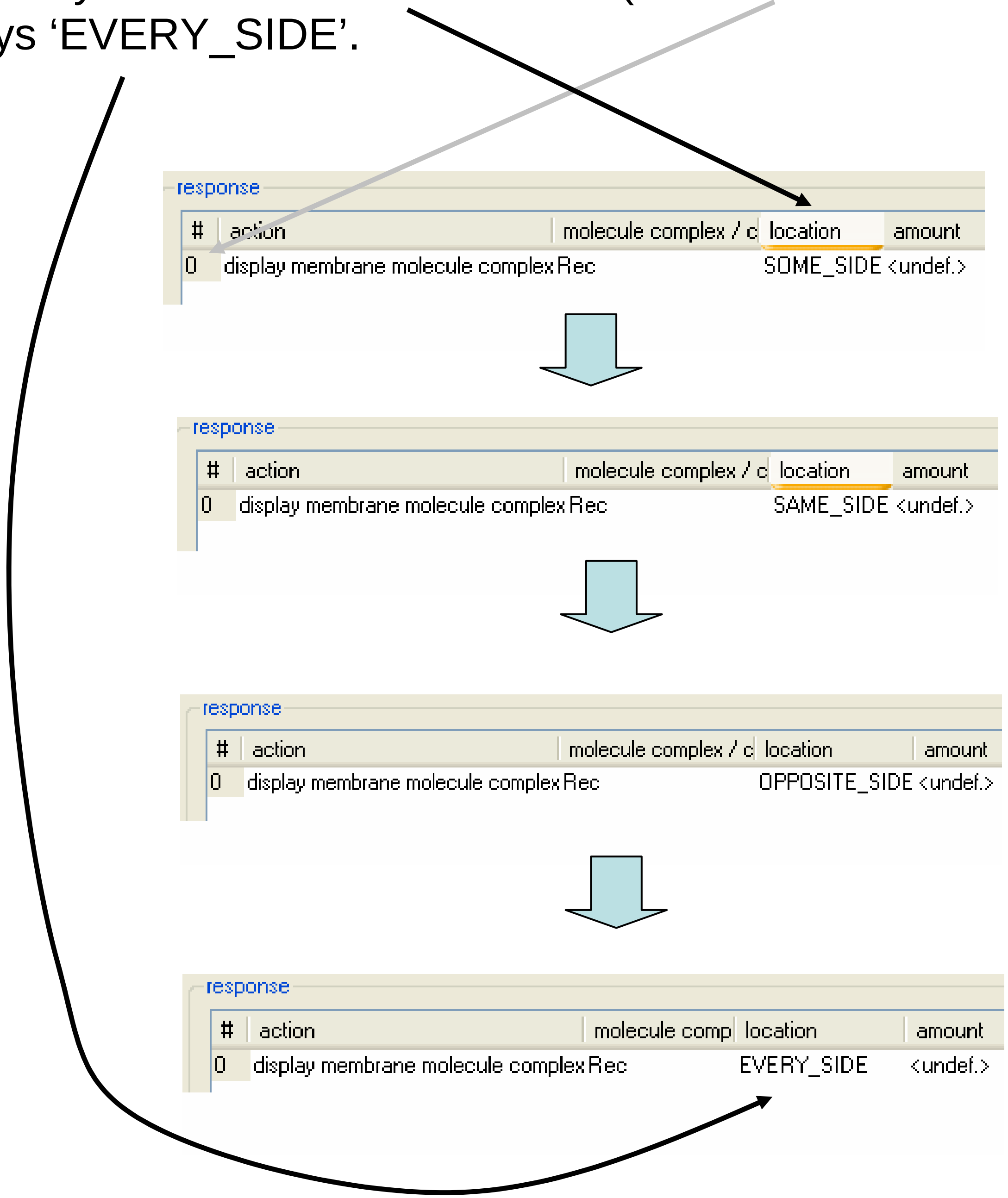

(61) Click the ‘amount’ section (with activated list entry) and enter 30000 as amount. The cell will thus display 30000 receptors on each of its 6 sides (cells in this version of Simmune have six sides, like a cube).

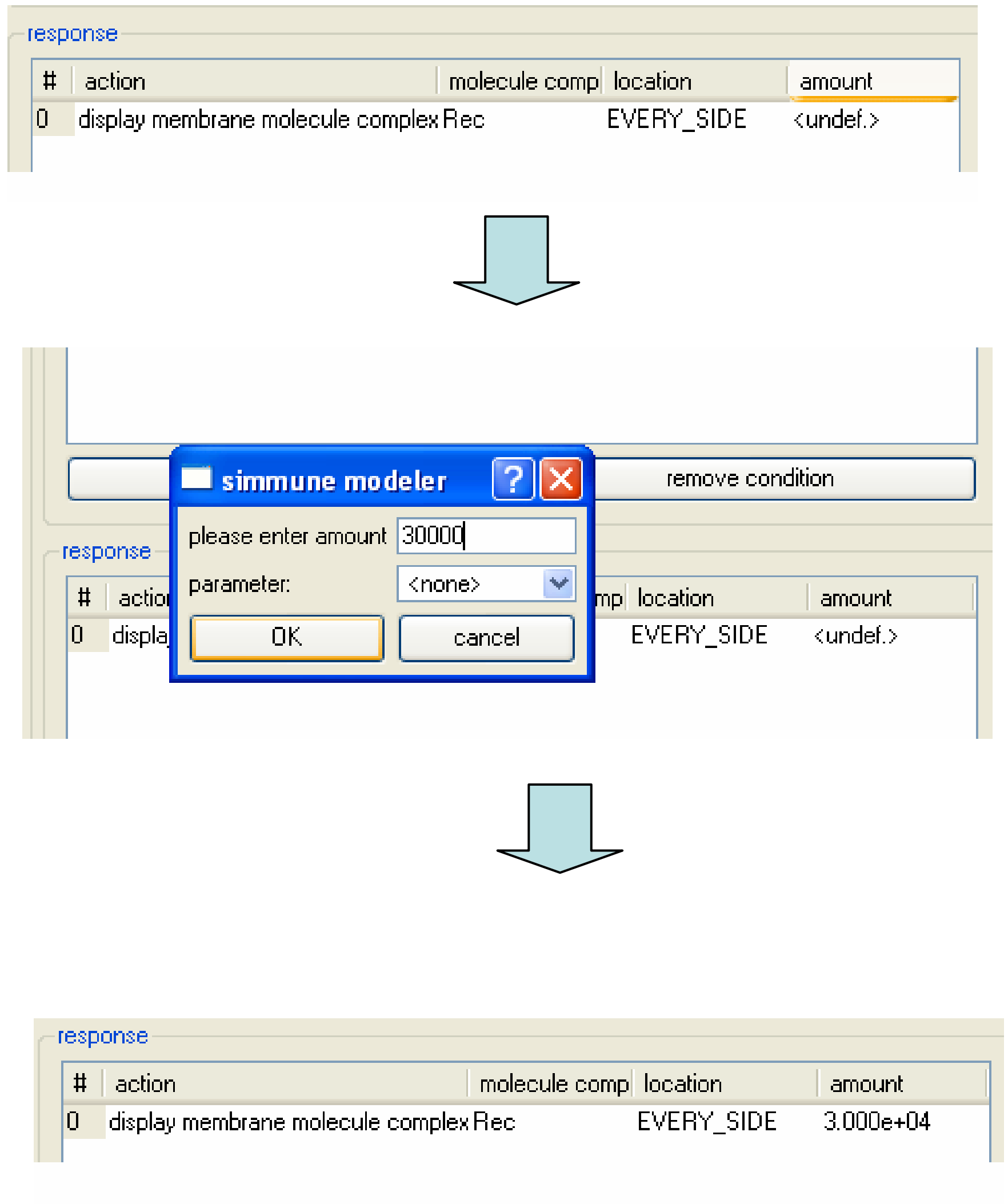

This completes the first entry in the actions list.

(62) Now make the cell produce different amounts of Activator, Inhibitor, and MembraneAnchor.

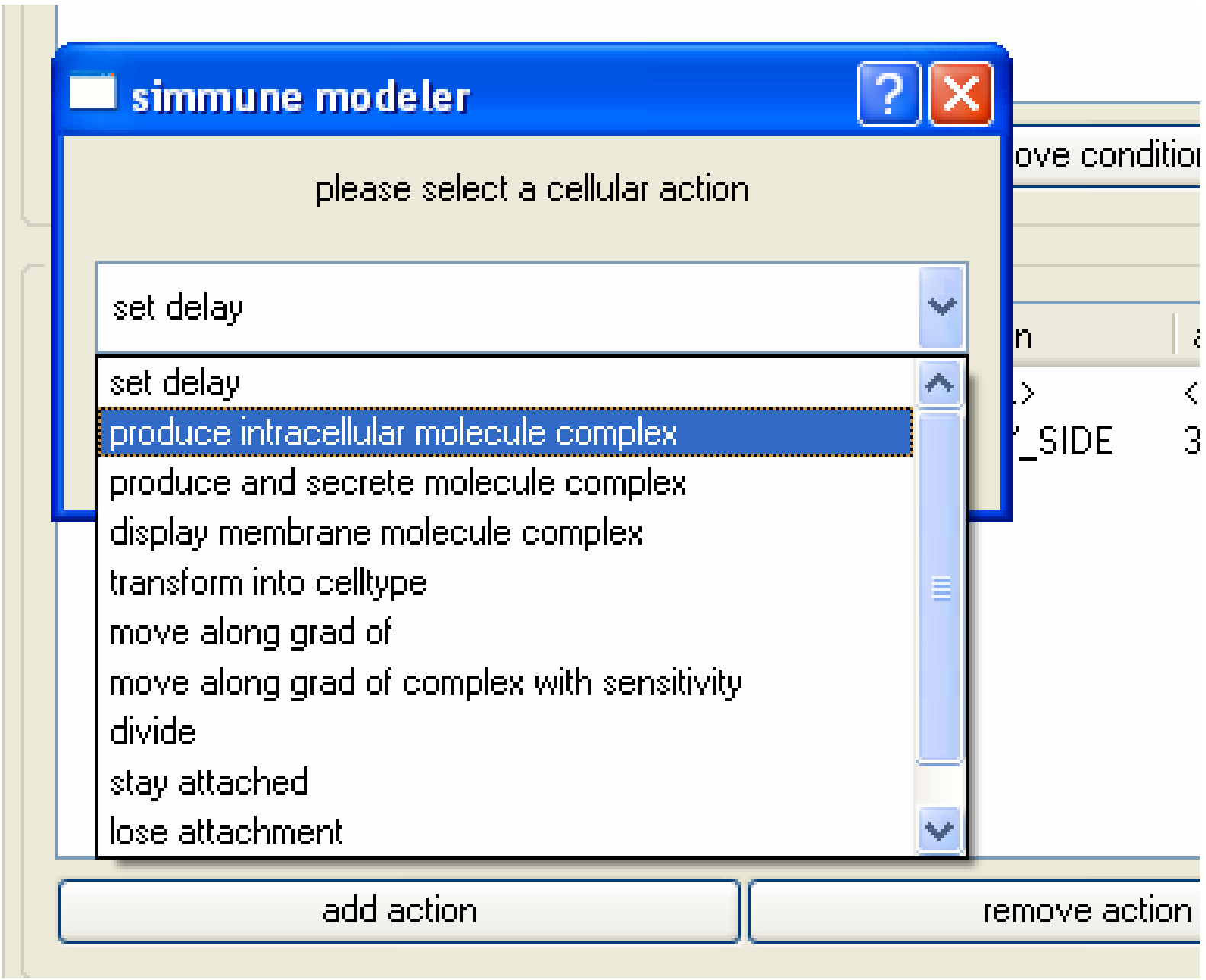

(63) The complete list of actions and the complete mechanism should look like shown below. Assign a name (e.g. 'init') to the mechanism and add it to the cell's behavior.

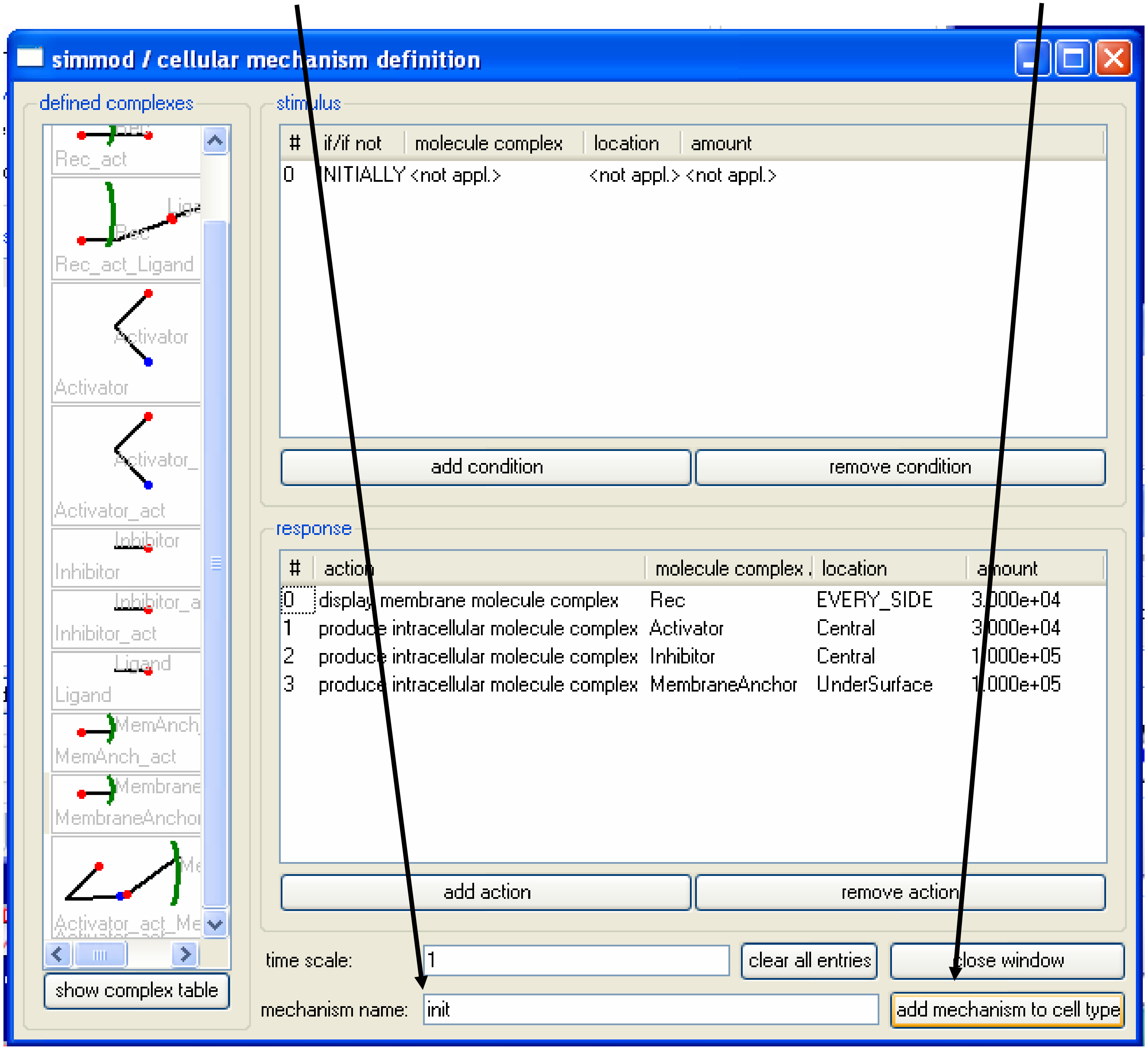

The cell window should now list the ‘init’ mechanism for the cell type ‘Cell’.

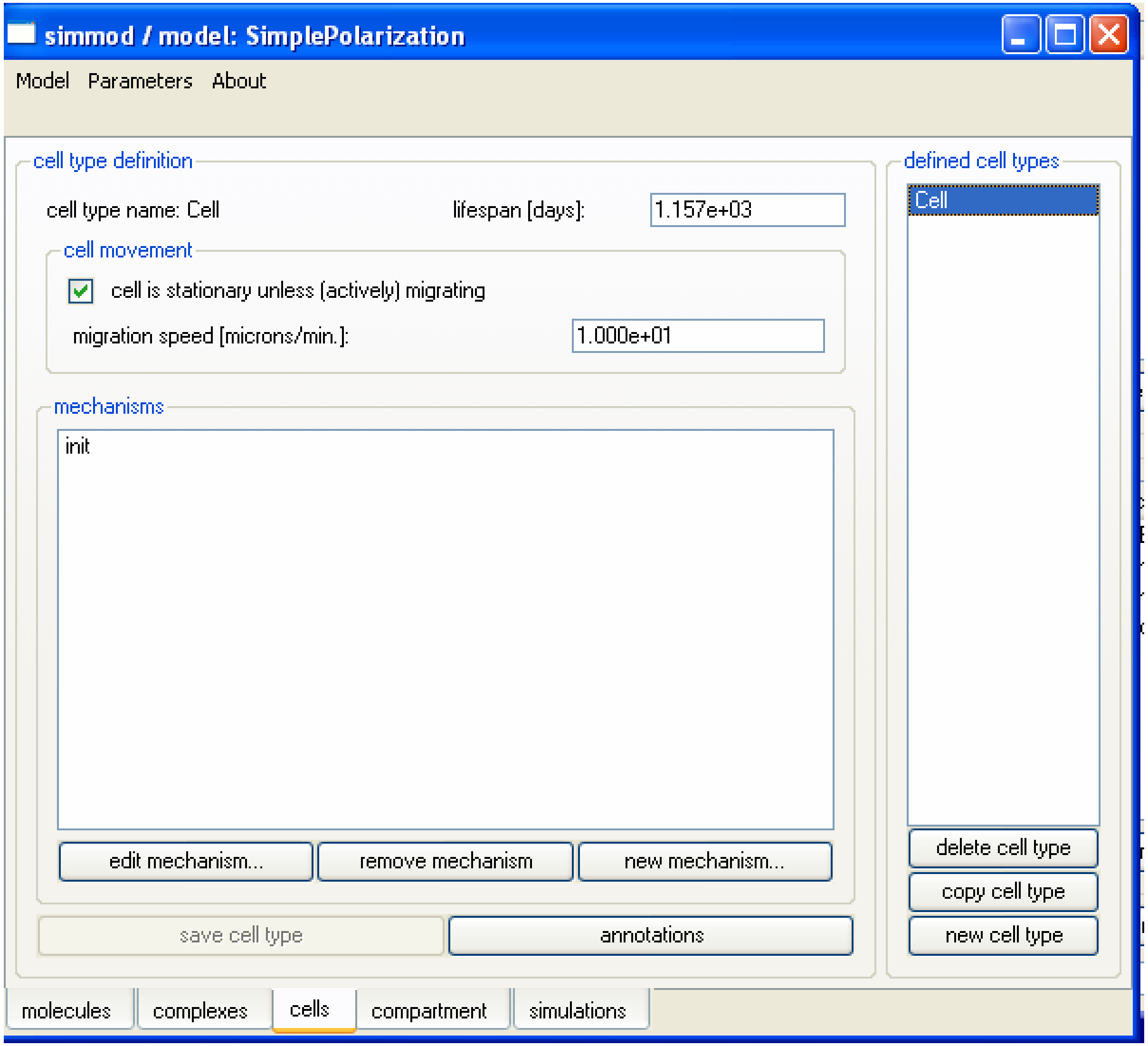

(64) Go to the ‘compartment’ tab and set the properties for the extracellular compartment as shown below. Then, save the compartment.

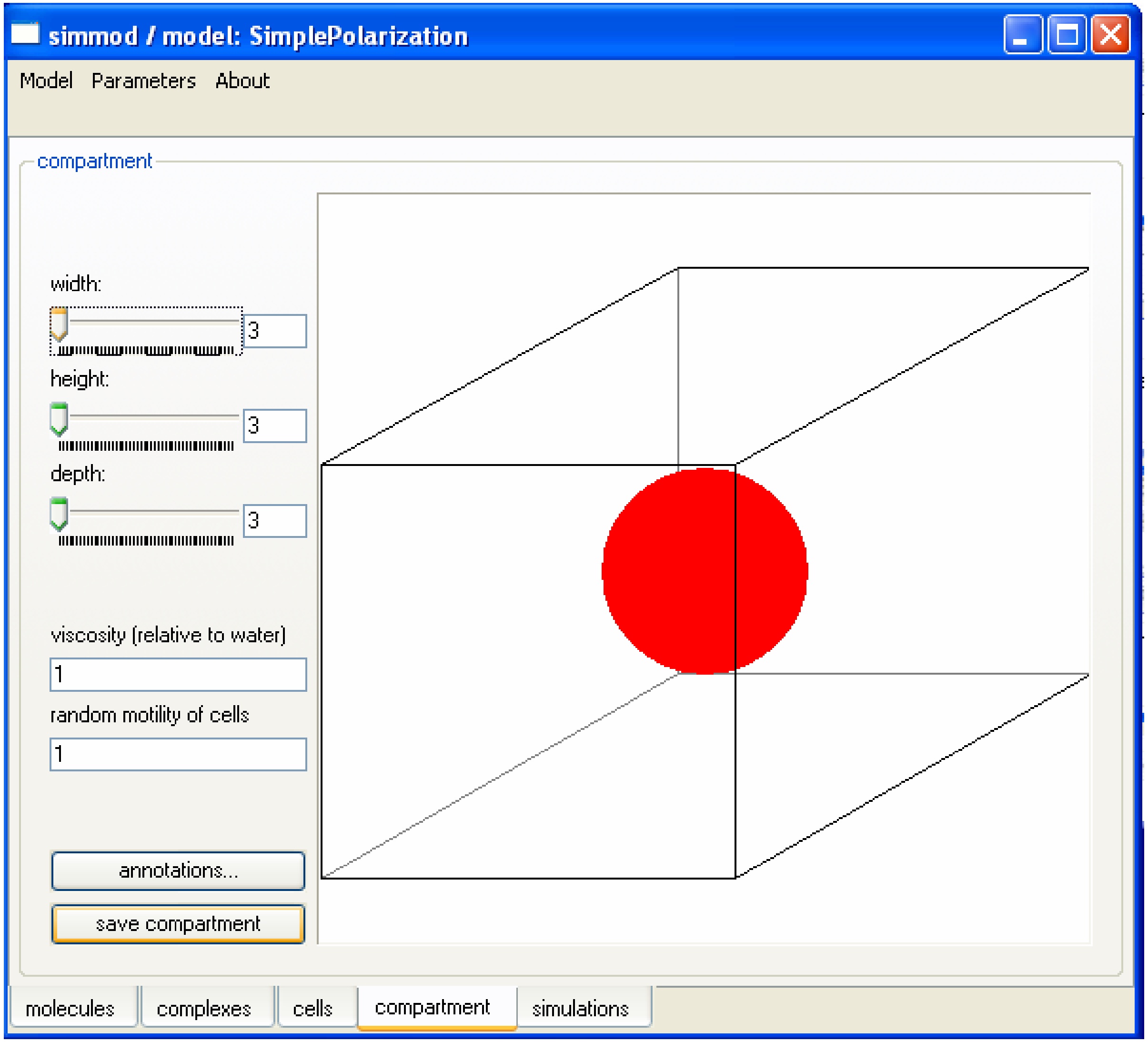

The compartment will be just large enough to have space for a single cell and one layer of extracellular space for the application of extracellular concentration gradients.

(65) Go to the 'simulations' tab and create a new simulation. Save it.

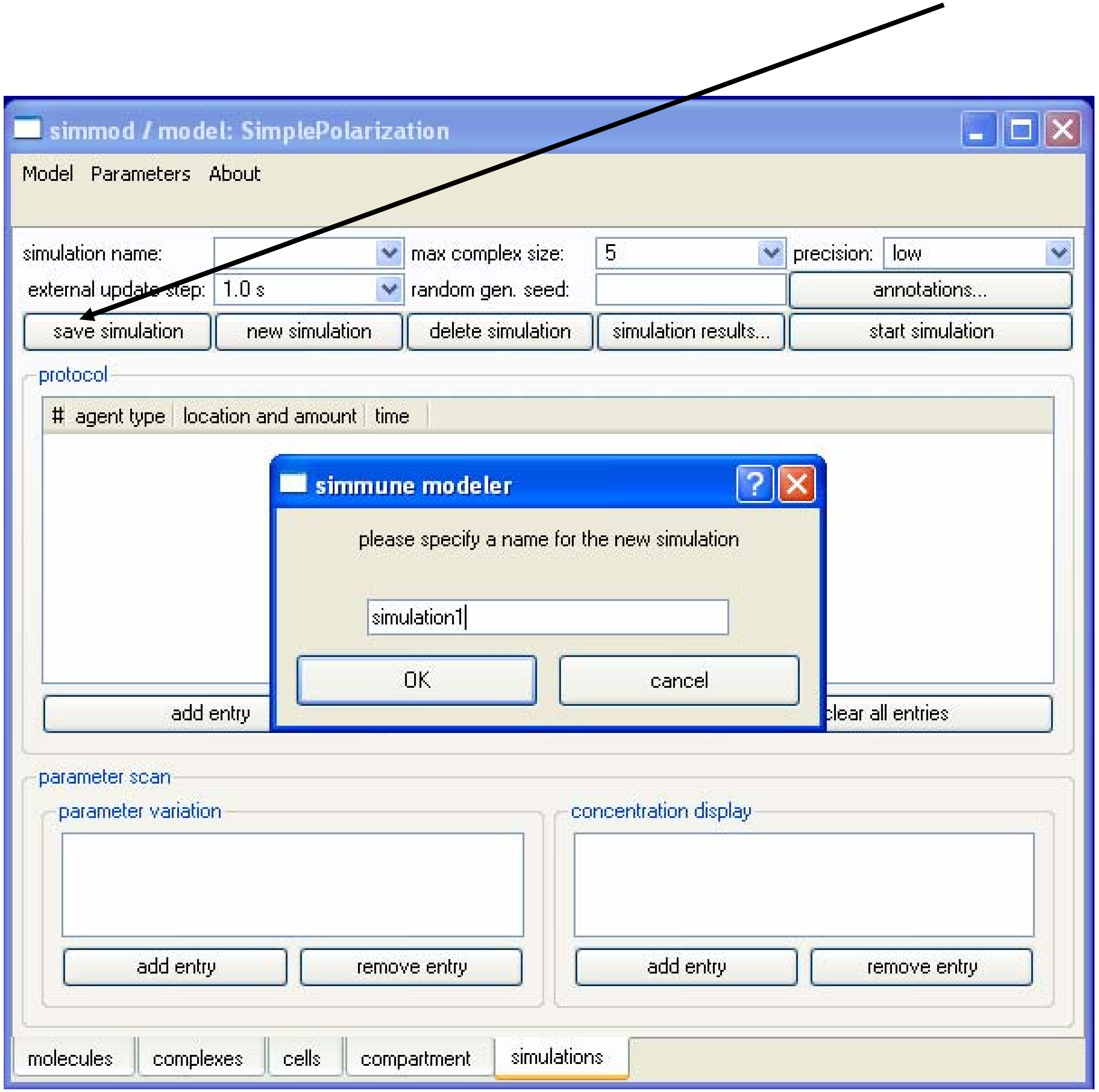

(66) Start the simulation.

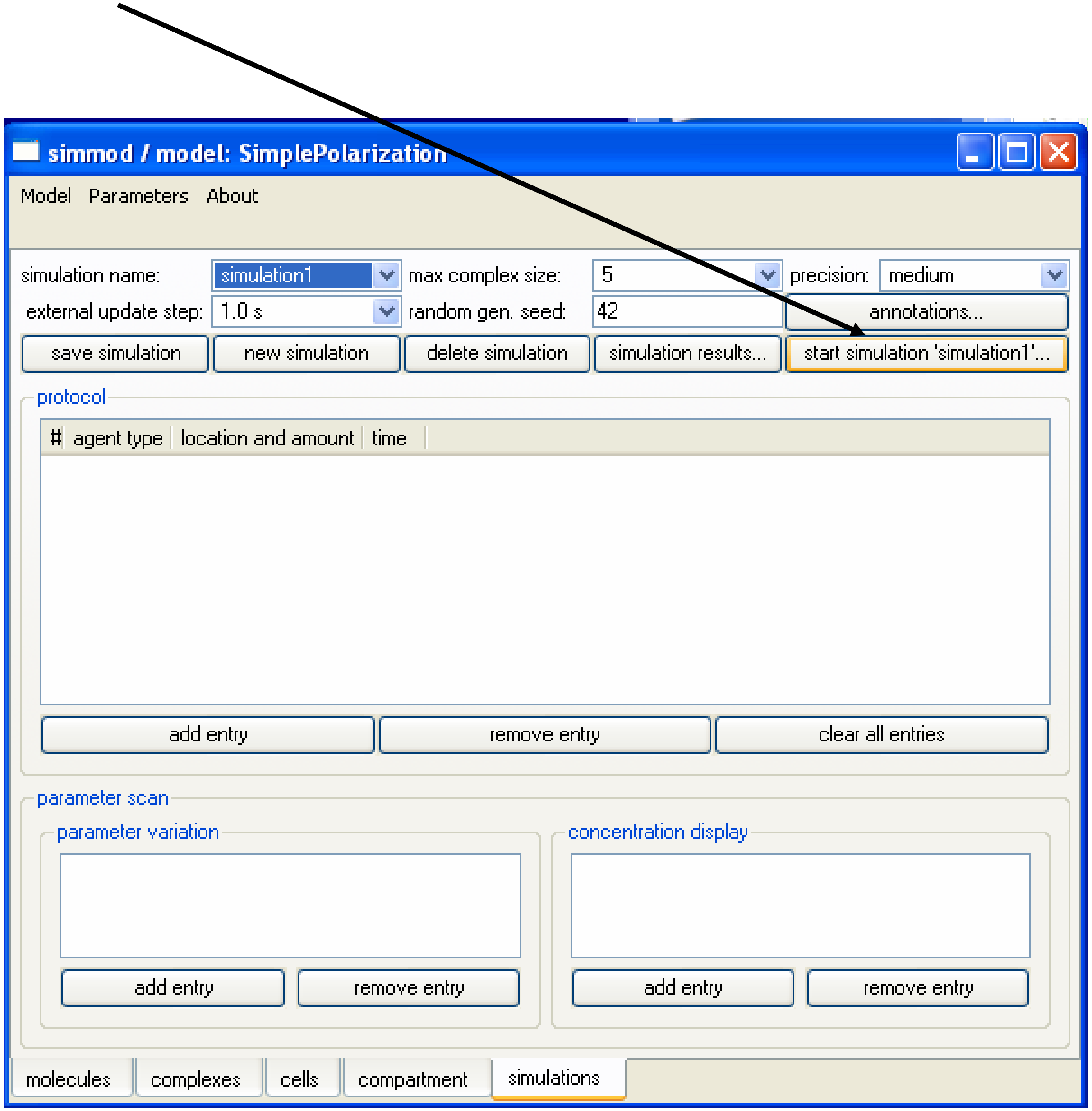

(67) The simulation main window should appear. Click on 'color codes' and double-click Cell and Ligand.

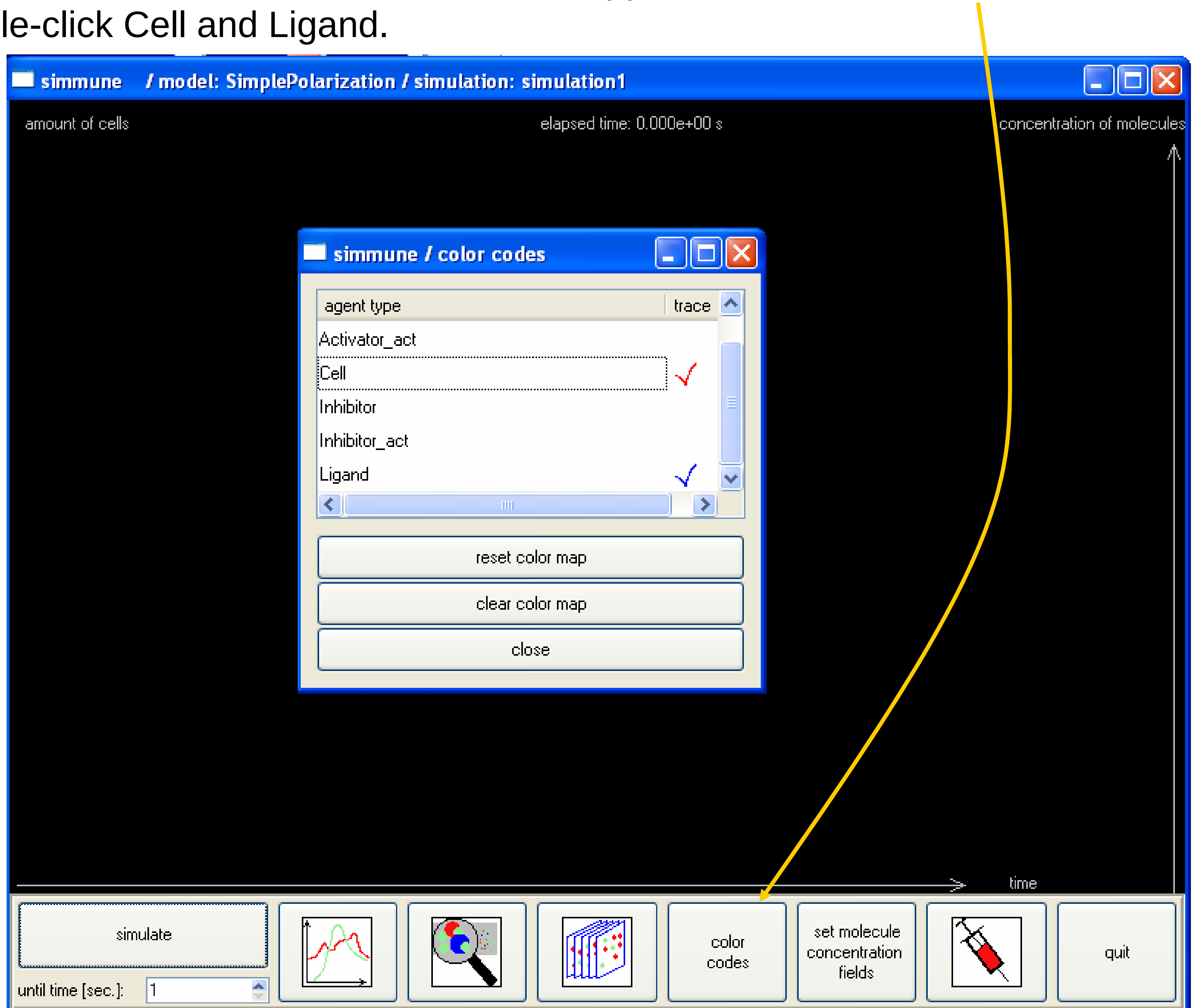

(68) Go to the 'slice-view'.

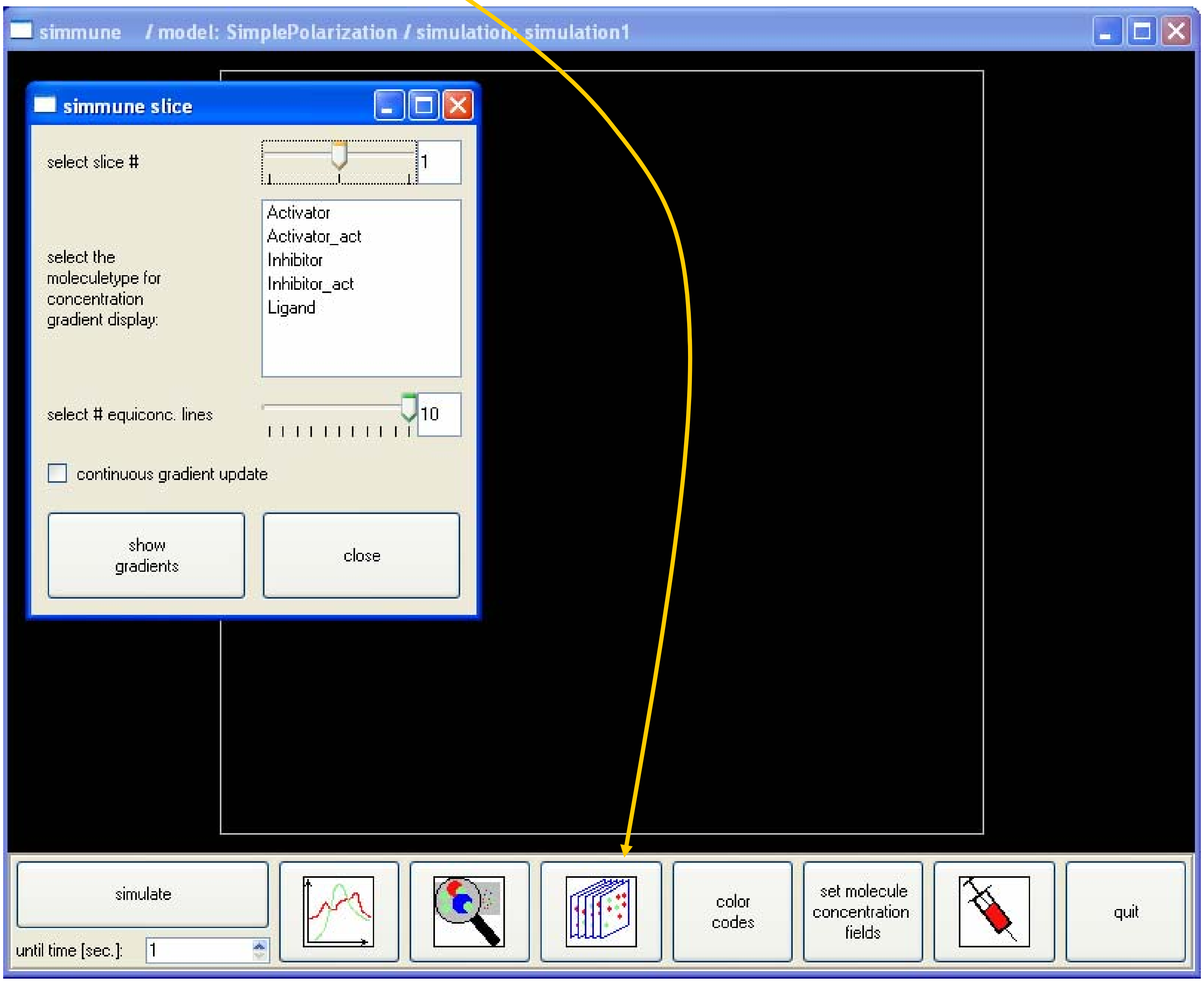

(69) Click on the button with the syringe symbol and select ‘Cell’ as agent to inject and ‘center’ (of the compartment) as location. Then, click ‘inject now’.

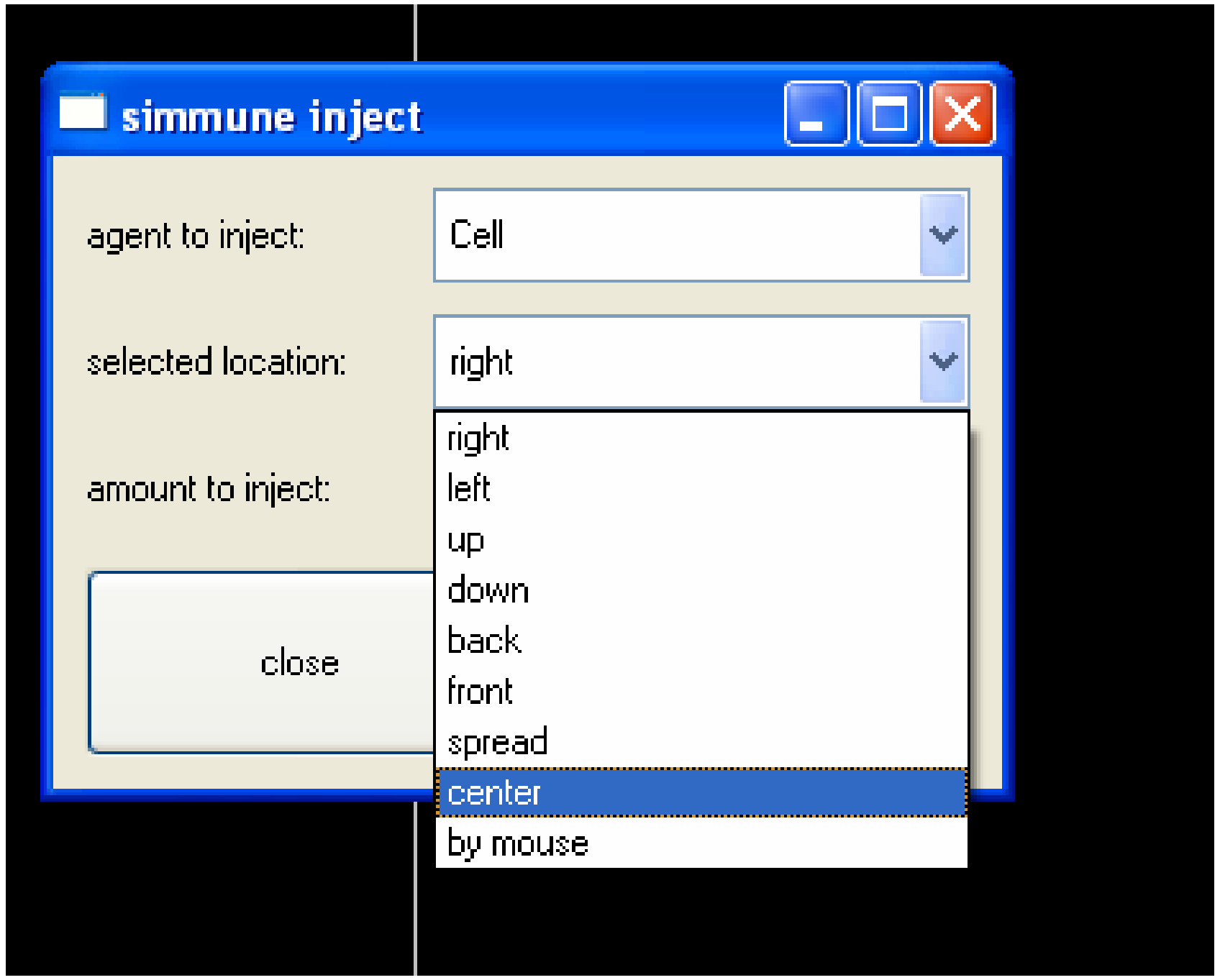

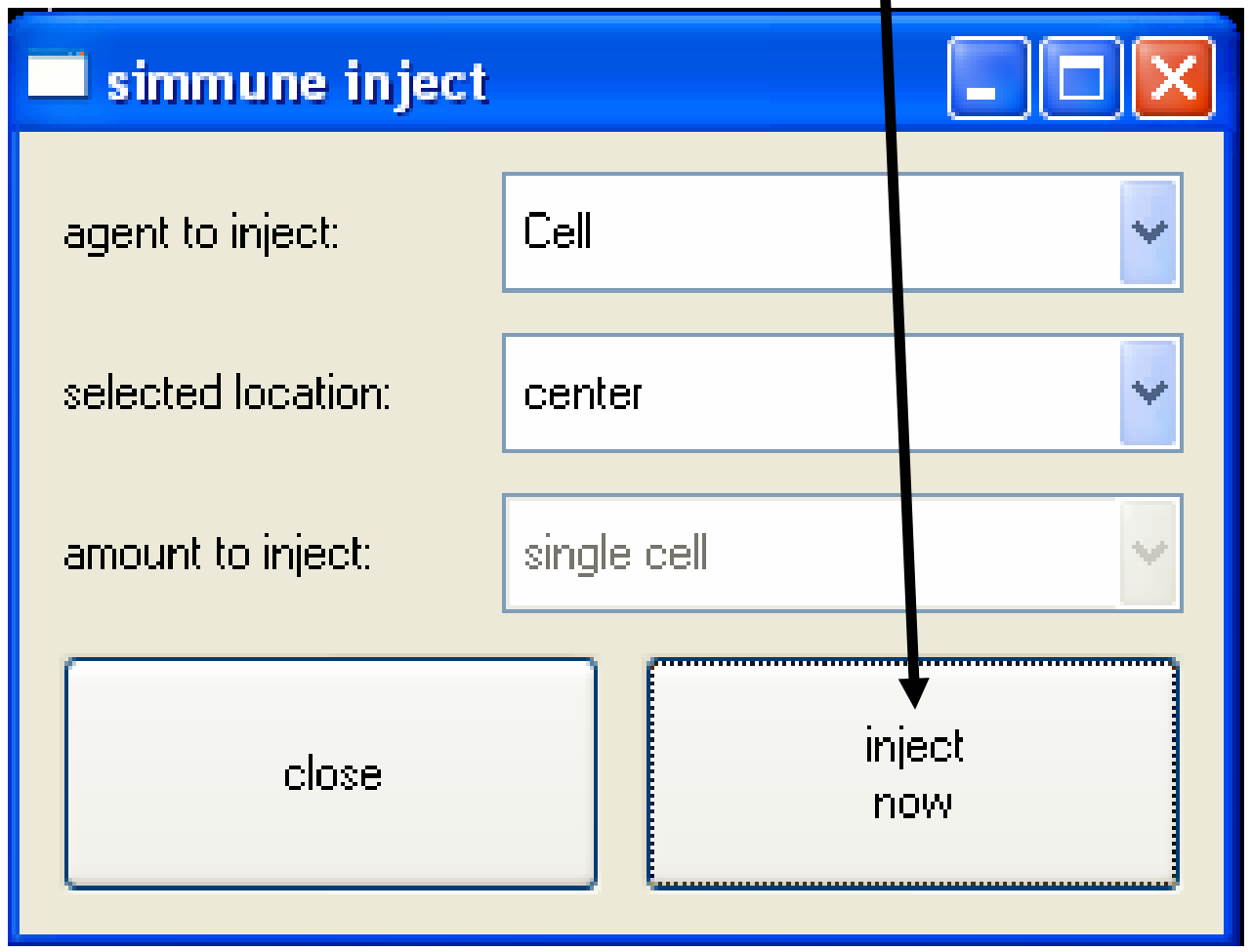

(70) The compartment with the cell should now look like this:

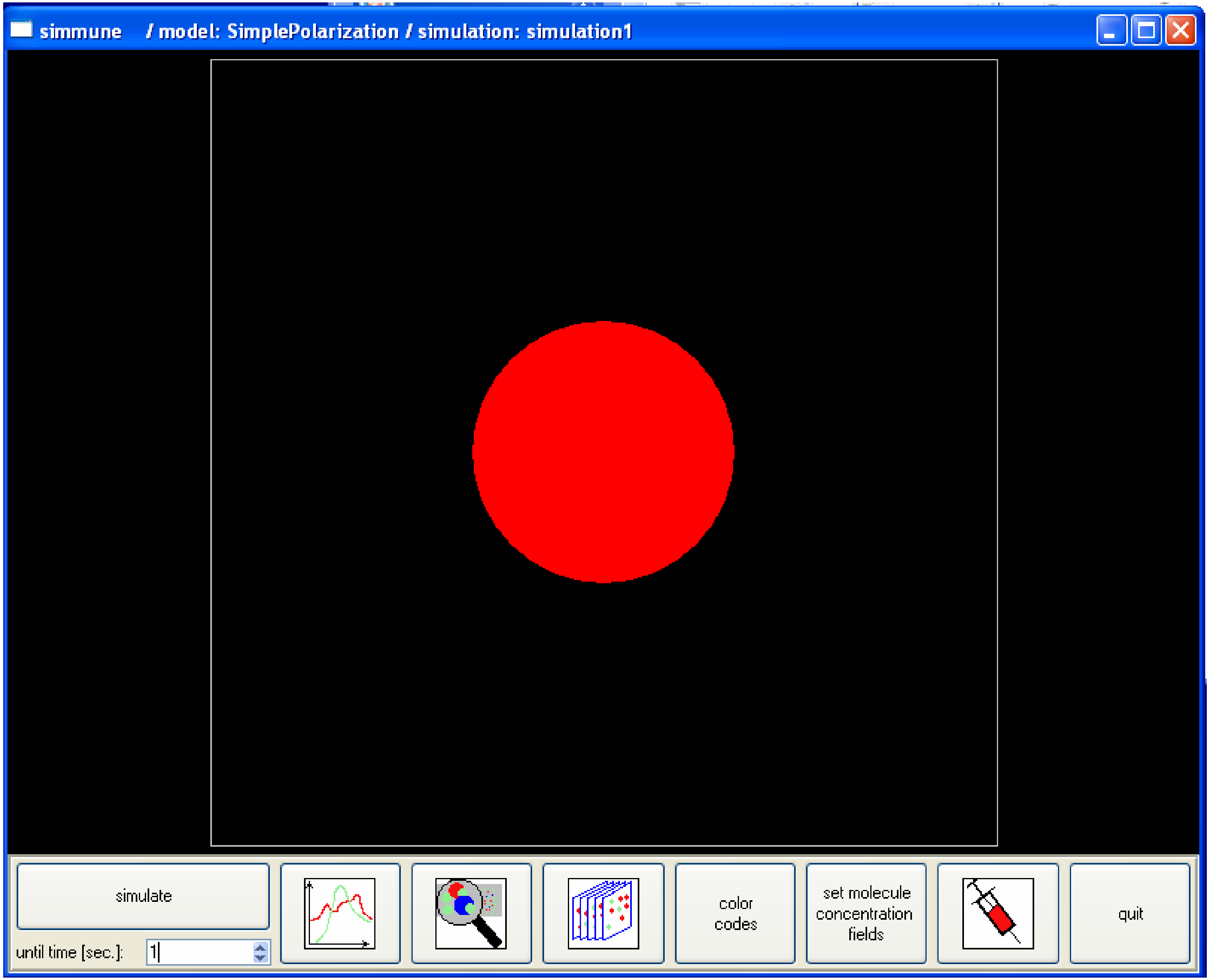

(71) Click on the ‘set molecule concentration fields’ button and select a linear vertical gradient of Ligand with the concentrations at the to and the bottom of the compartment as shown below. Then, click ‘set concentration field’.

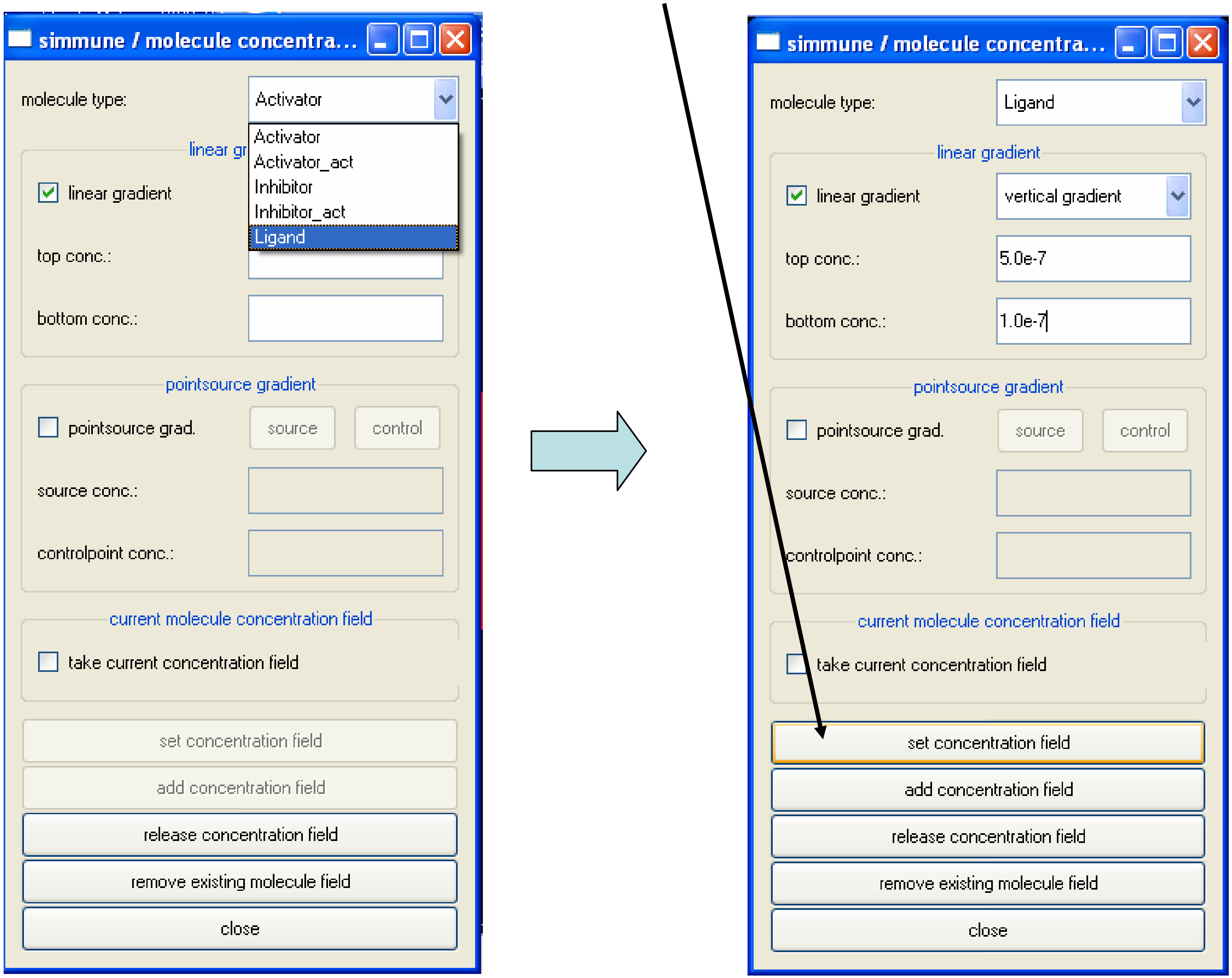

The compartment should now look like this. The blue lines are iso-concentration lines with the concentrations given on the left.

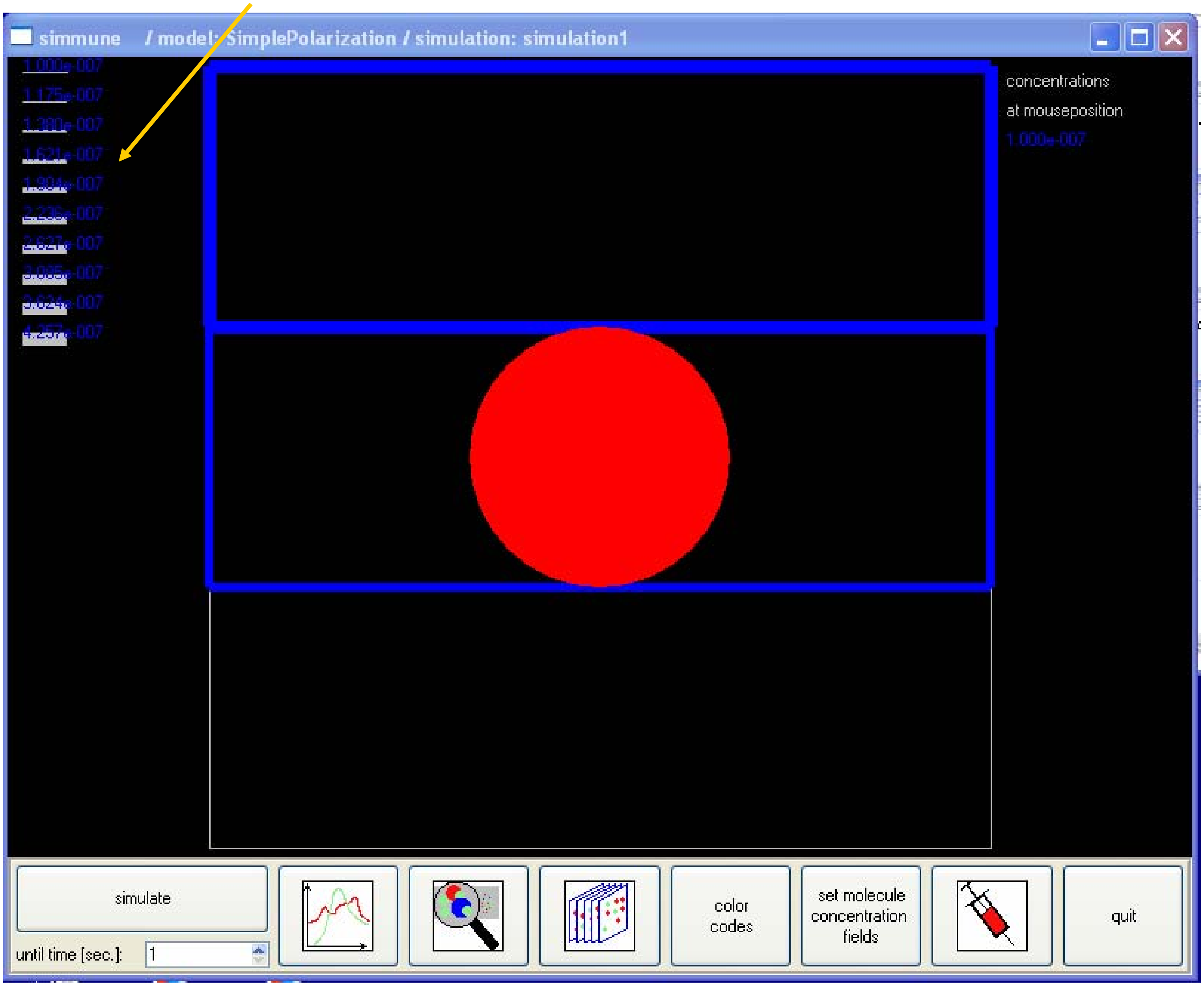

(72) Click on the red dot representing the cell. Click ‘yes’ to activate a new cell data display that will allow us to monitor intracellular concentrations of the complexes and molecule bundles we have defined.

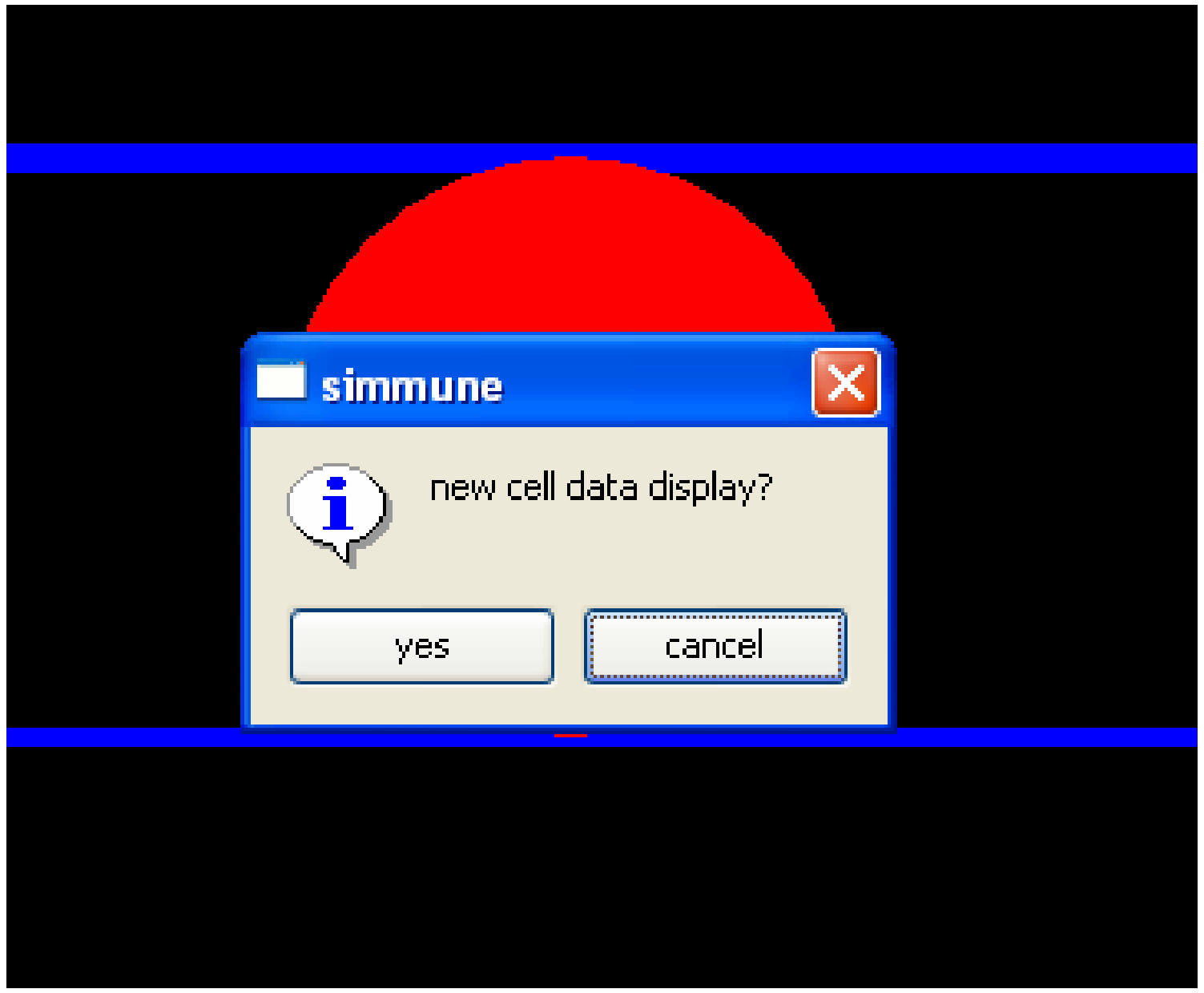

(73) This is the cell data display that should appear. Since nothing has been simulated so far it does not show any complex entries.

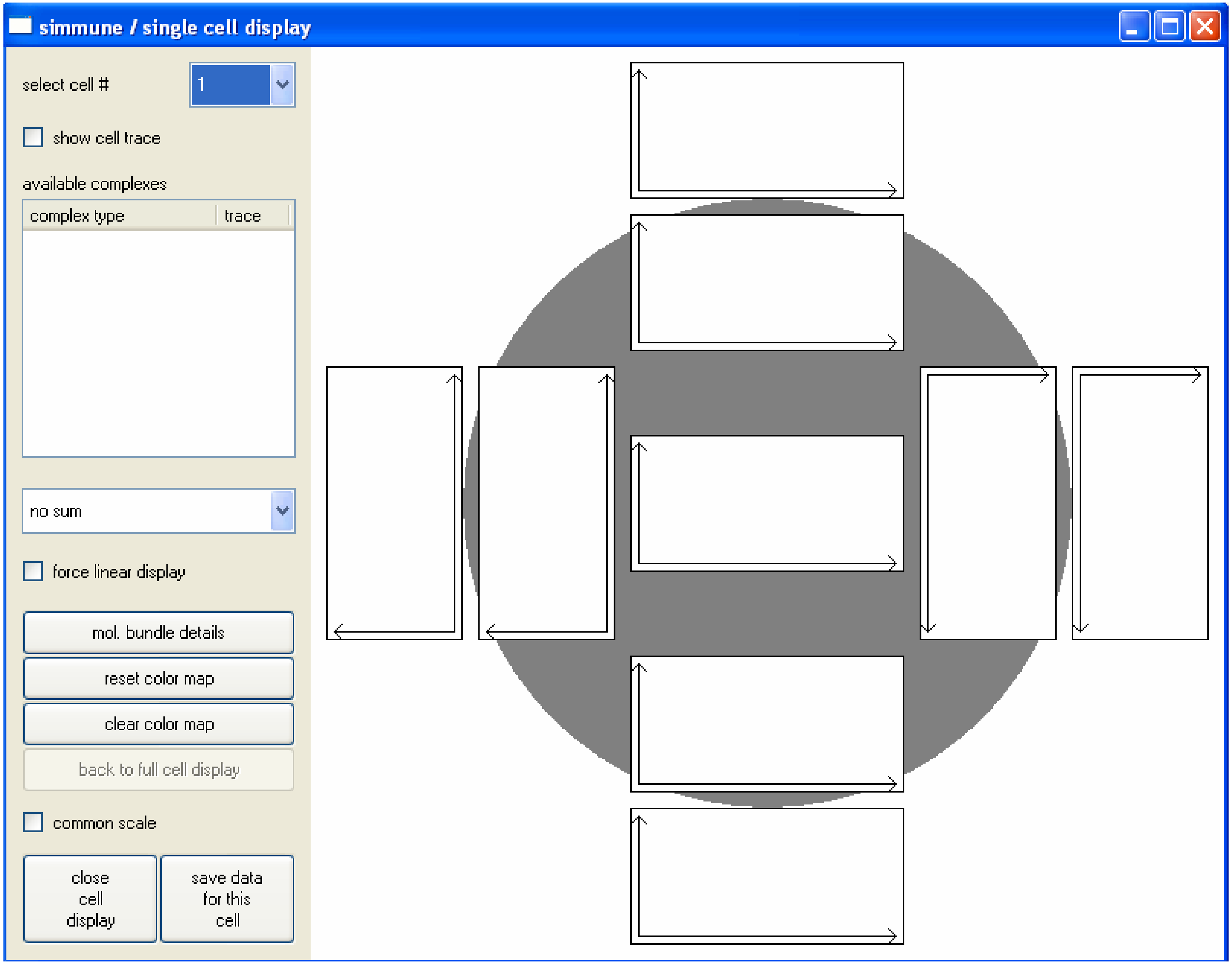

(74) Set a simulation end time of 150 seconds and click on ‘simulate’.

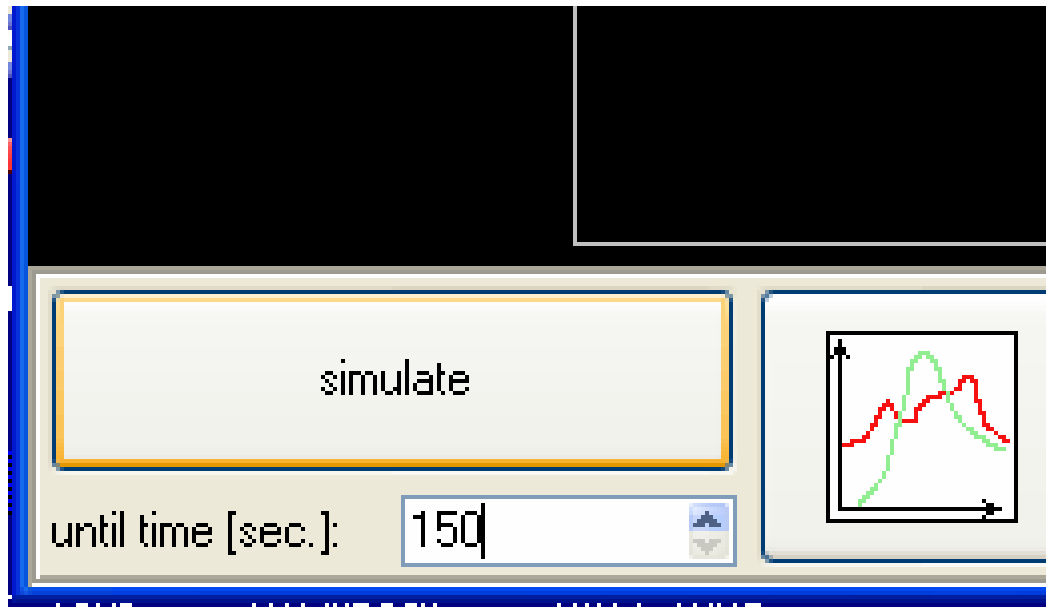

(75) This is how the cell data display should look like after you double-clicked the All_Activator_act' bundle in the list of available complexes to assign a color to the bundle. Double-click the upper intracellular region to inspect it in detail.

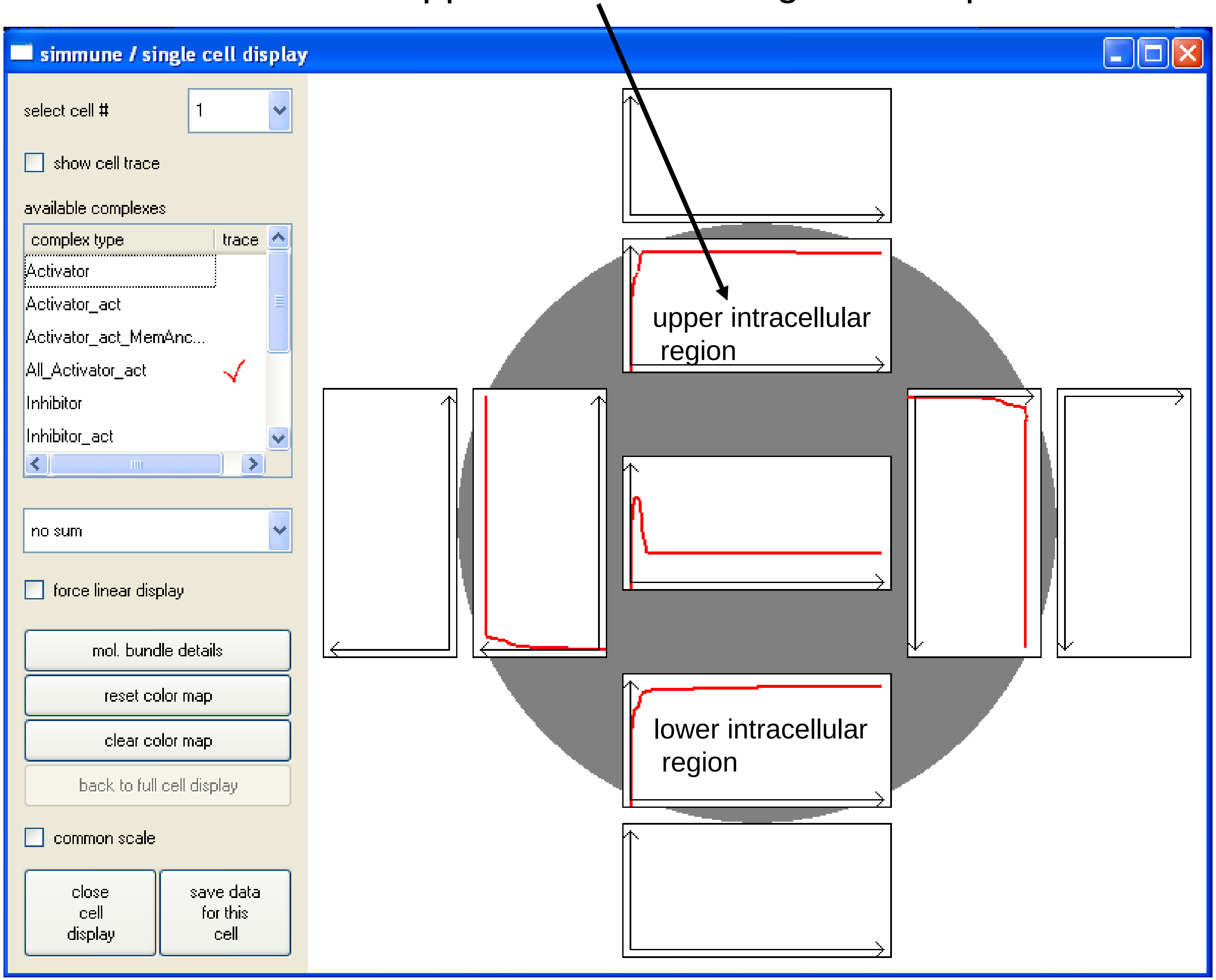

(76) Moving the mouse over the diagram makes the software display the amount of molecules at the chosen position along the time and amount axes. For the screenshot below the mouse was where indicated by the red arrow.This position corresponded thus to the number of 5861 molecule complexes (at t = 13 s).

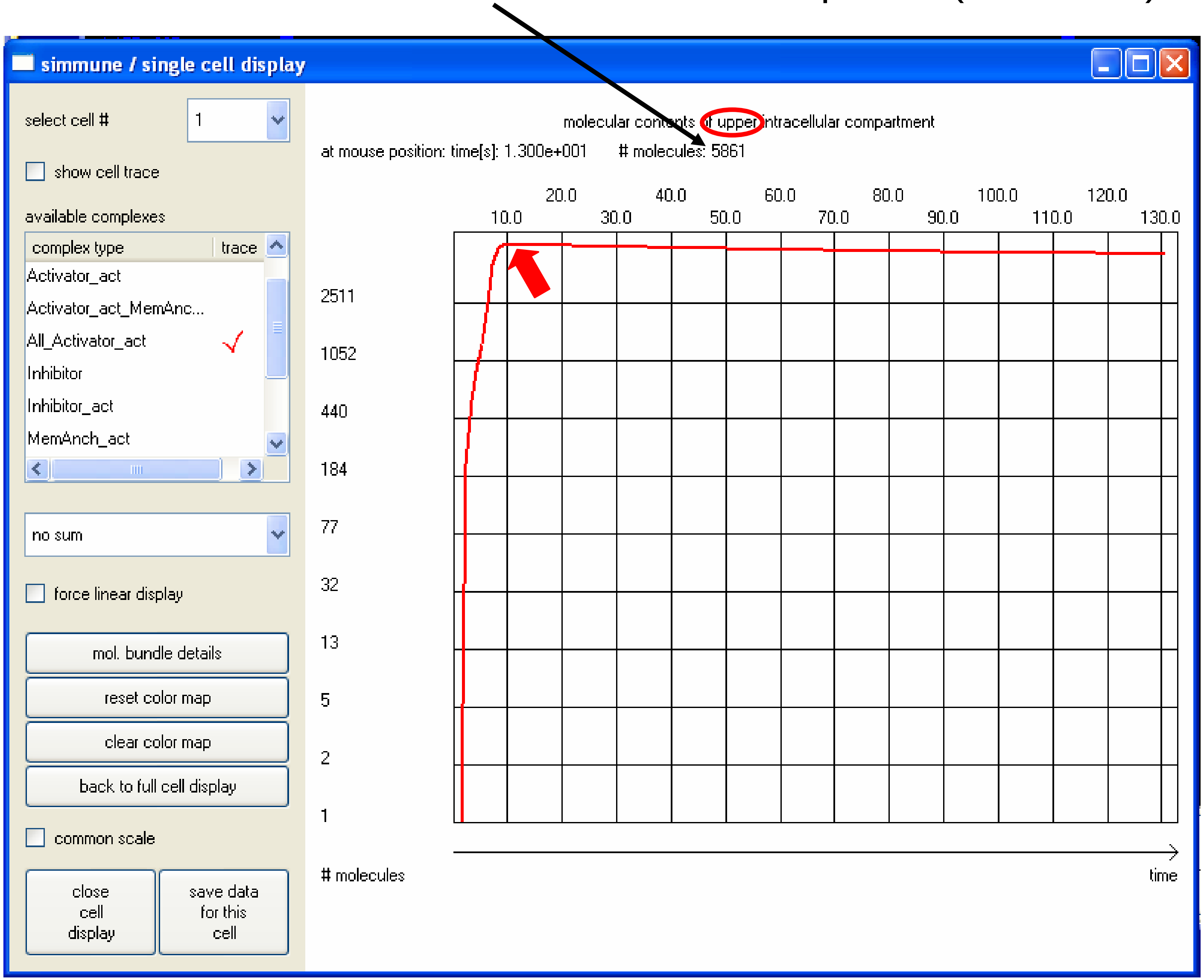

(77) For this screenshot of the lower intracellular region (whose membrane is exposed to the lower dose of Ligand) the mouse was where indicated by the red arrow.This position corresponded thus to the number of 3183 molecule complexes (at t = 12 s).

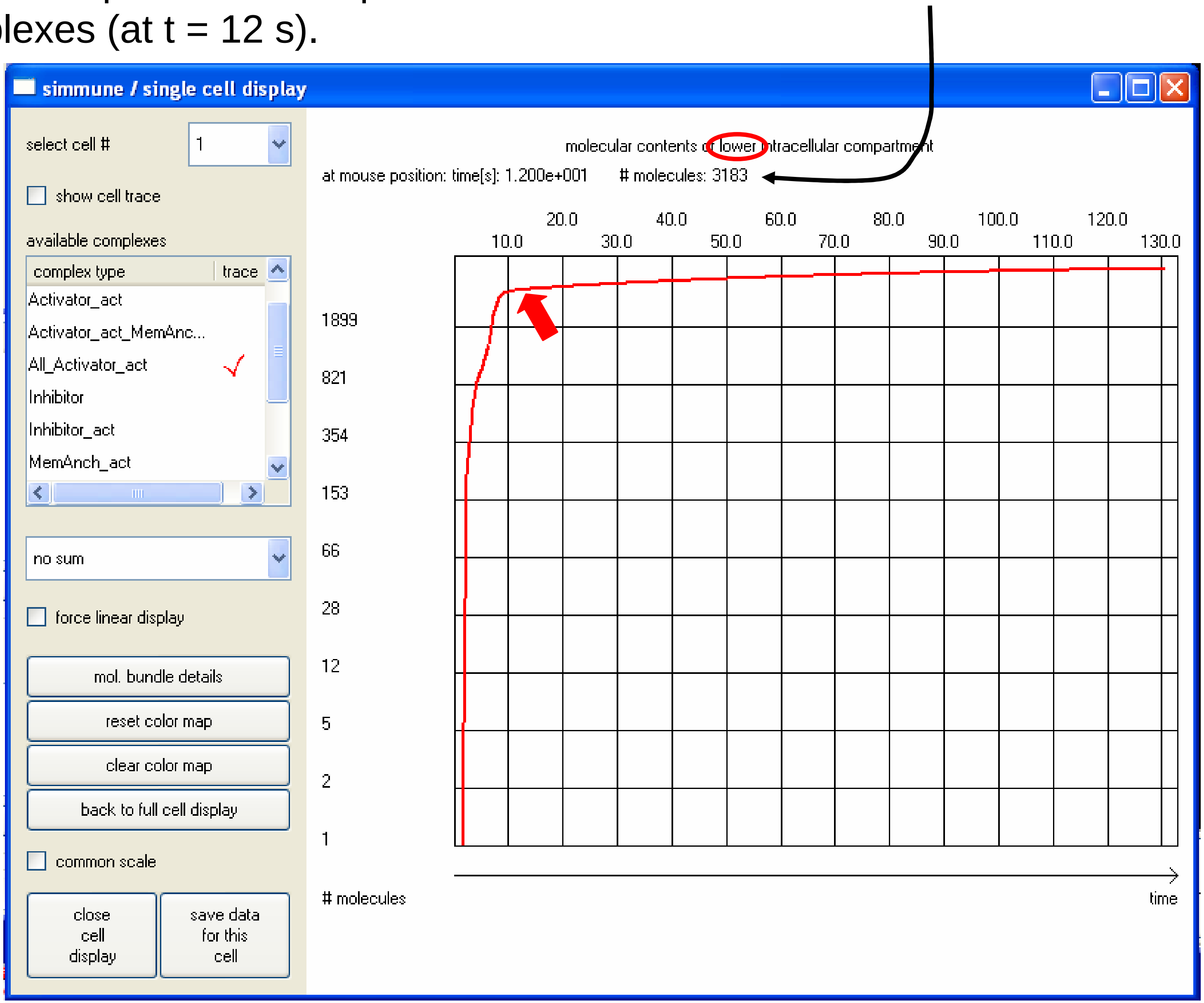

(78) Double-clicking ‘Rec_act_Ligand’ will assign a color for the concentration time course display of the amount of complexes consisting of the receptor and the ligand. Note, however, that this does not correspond to the total number of ligated receptors since some of them might be interacting with the Activator or Inhibitor. To see the total amount of ligated receptors you have to define a bundle consisting of the receptor and the ligand (see points 41-43).

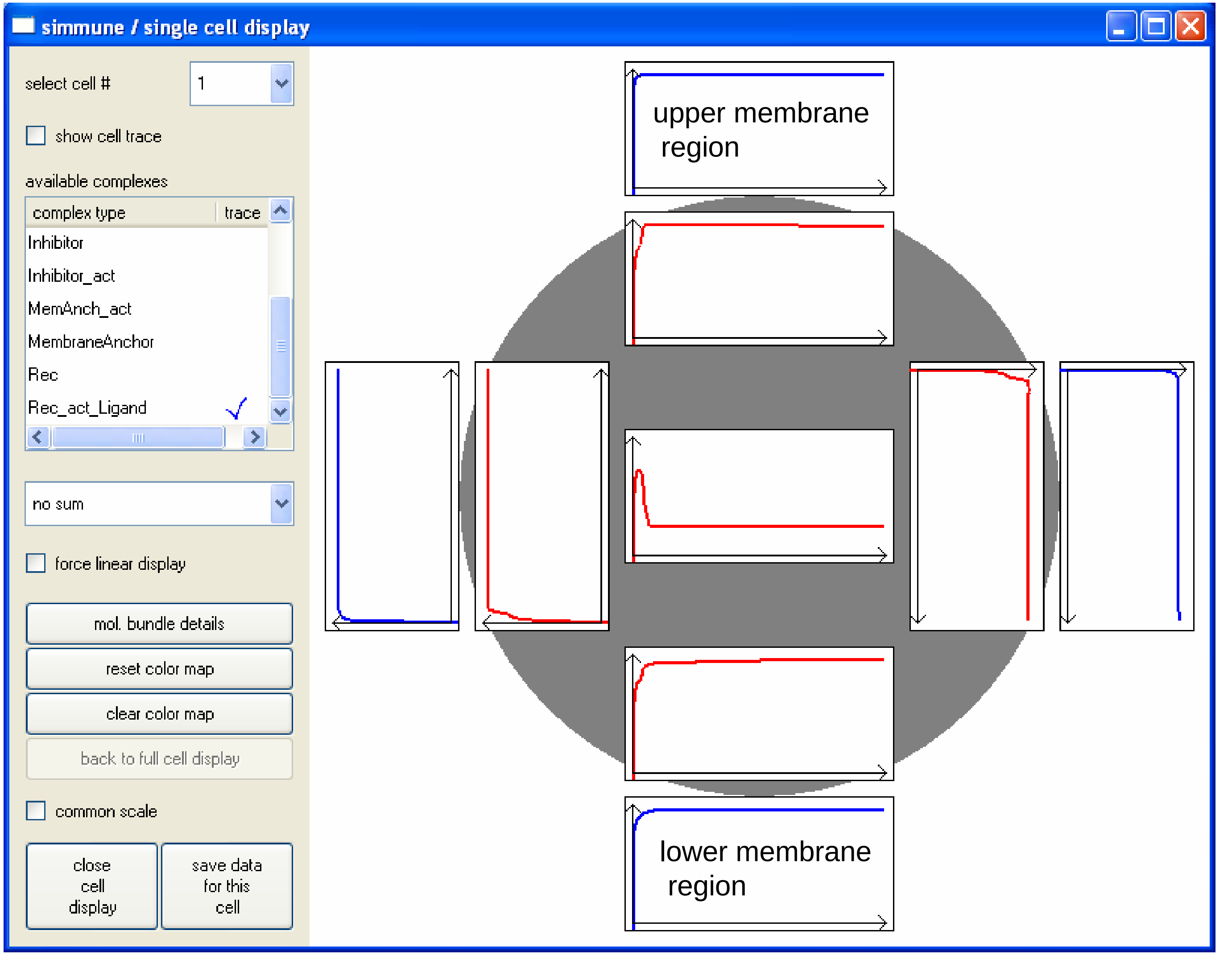

(79) Double-click the upper membrane region to inspect the amount of 'Rec_act_Ligand' and compare it to the corresponding amount of this complex in the lower membrane region.

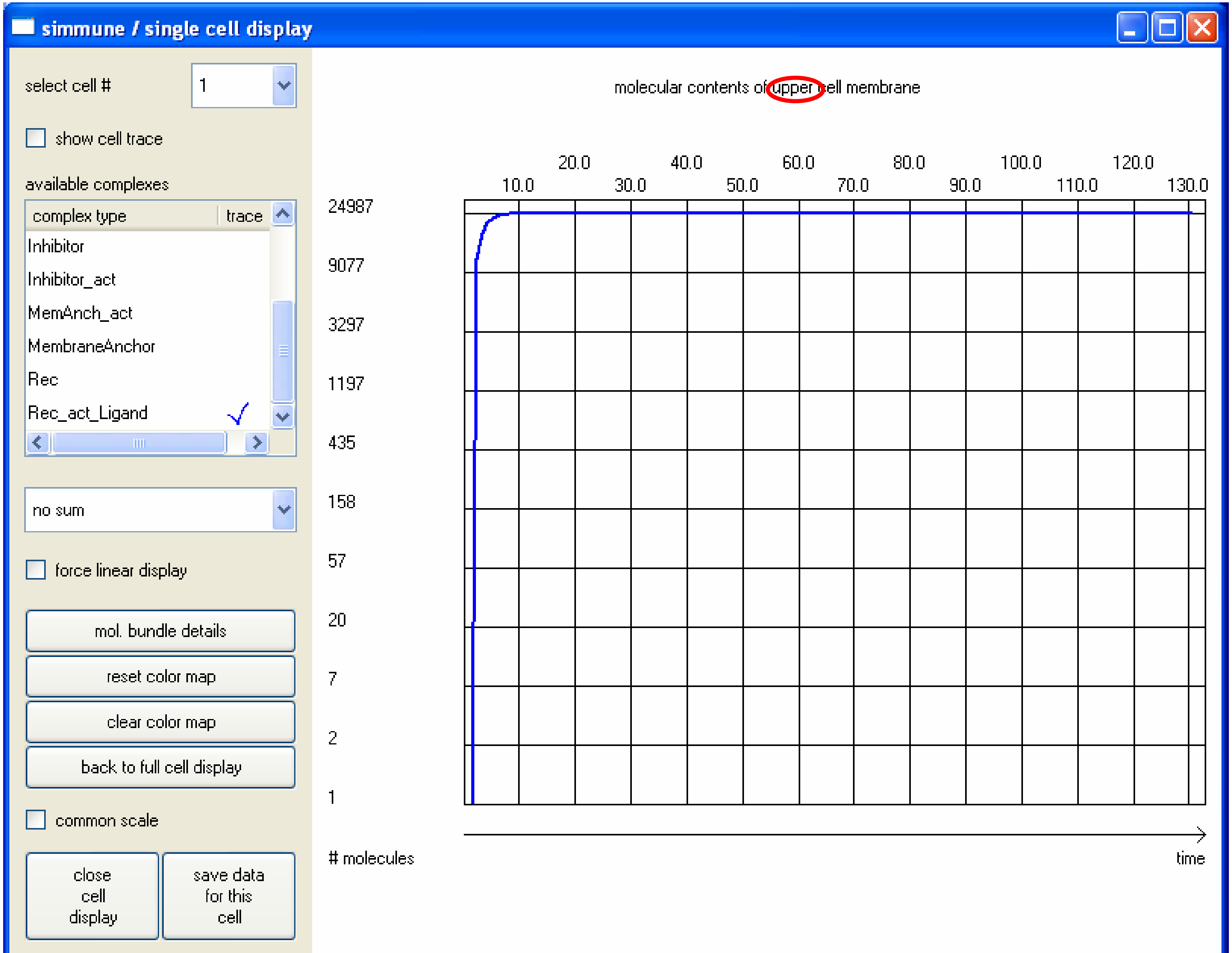

Analysis / suggestions

(1) The simple model created and simulated here does obviously not create a strong (amplified when compared to the extracellular gradient) or stable intracellular polarization of our read-out ‘Activator_act’. The simple feedback mechanism is not efficient enough. The initial polarization decays because the MembraneAnchor diffuses.

(2) You might want to experiment with the diffusion coefficient of the MembraneAnchor (for example setting it to 1.0e-15 m^2/s which would correspond to the diffusion of large transmembrane proteins) to see how it affects the stability of the polarization.

(3) You could create additional feedback elements. For example a mechanism that excludes the action of the inhibitor from the side that accumulates ‘Activator_act’. Such mechanisms are at work in ‘real’ eukaryotic cells that accumulate PIP_3, an ‘excitatory’ component, at the side of the cell facing the high concentration of the extracellular ‘chemoattractant’ and exclude PTEN (which metabolizes PIP_3) from that side.

(4) The receptor binding curve (dose-response of bound receptor vs. concentration of ligand) shows an effect called saturation: the relative differences between the numbers of ligated receptors for a given relative difference in the concentration of ligand become smaller with increasing ligand concentration. Apply a gradient with a 5:1 concentration difference (as in the tutorial) but with a higher absolute concentration (like, e.g ~ 1.0e-6 mol). You will see that the cell can polarize to a much lesser degree because the relative differences in receptor occupancy between top and bottom (or front and back) become smaller.

Potential reasons for your model to fail to polarize (check your implementation of the tutorial model – perhaps you forgot to save some changes along the way…):

-- the diffusion coefficient of the receptor is too high.

-- the diffusion coefficient of the MembraneAnchor is too high.

-- the binding of Activator and Activator_act to MemAnch_act is too weak.

-- the amount of Activator molecules in the cell is too high – part of the polarization mechanisms is that different sides of the cell compete for the Activator.
