# Supplementary material for: Key Role of Local Regulation in Chemosensing Revealed by a New Molecular Interaction-Based Modeling Method
Source: PLoS Comput Biol. 2006 Jul 21;2(7):e82. doi: 10.1371/journal.pcbi.0020082 (PMC1513273; doi:10.1371/journal.pcbi.0020082)
Supplement: Supplementary file 15 [file pcbi.0020082.sg001.pdf]

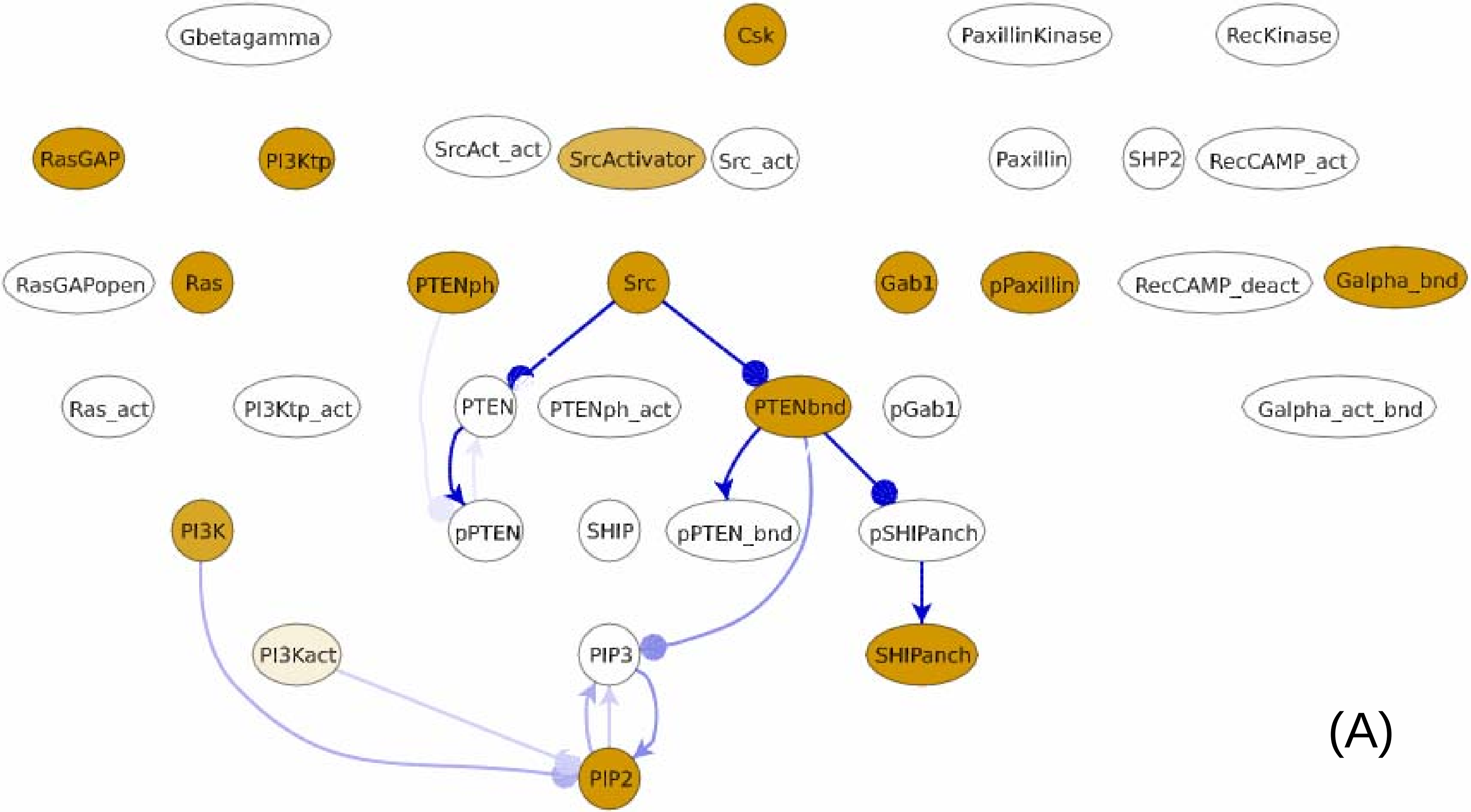

Gbetagamma
Csk
PaxillinKinase
RecKinase
RasGAP
PI3Ktp
SrcAct_act
SrcActivator
Src_act
Paxillin
SHP2
RecCAMP_act
RasGAPopen
Ras
PTENph
Src
Gab1
pPaxillin
RecCAMP_deact
Galpha_bnd
Ras_act
PI3Ktp_act
PTEN
PTENph_act
PTENbnd
pGab1
Galpha_act_bnd
PI3K
pPTEN
SHIP
pPTEN_bnd
pSHIPanch
PI3Kact
PIP3
SHIPanch
PIP2

(A)

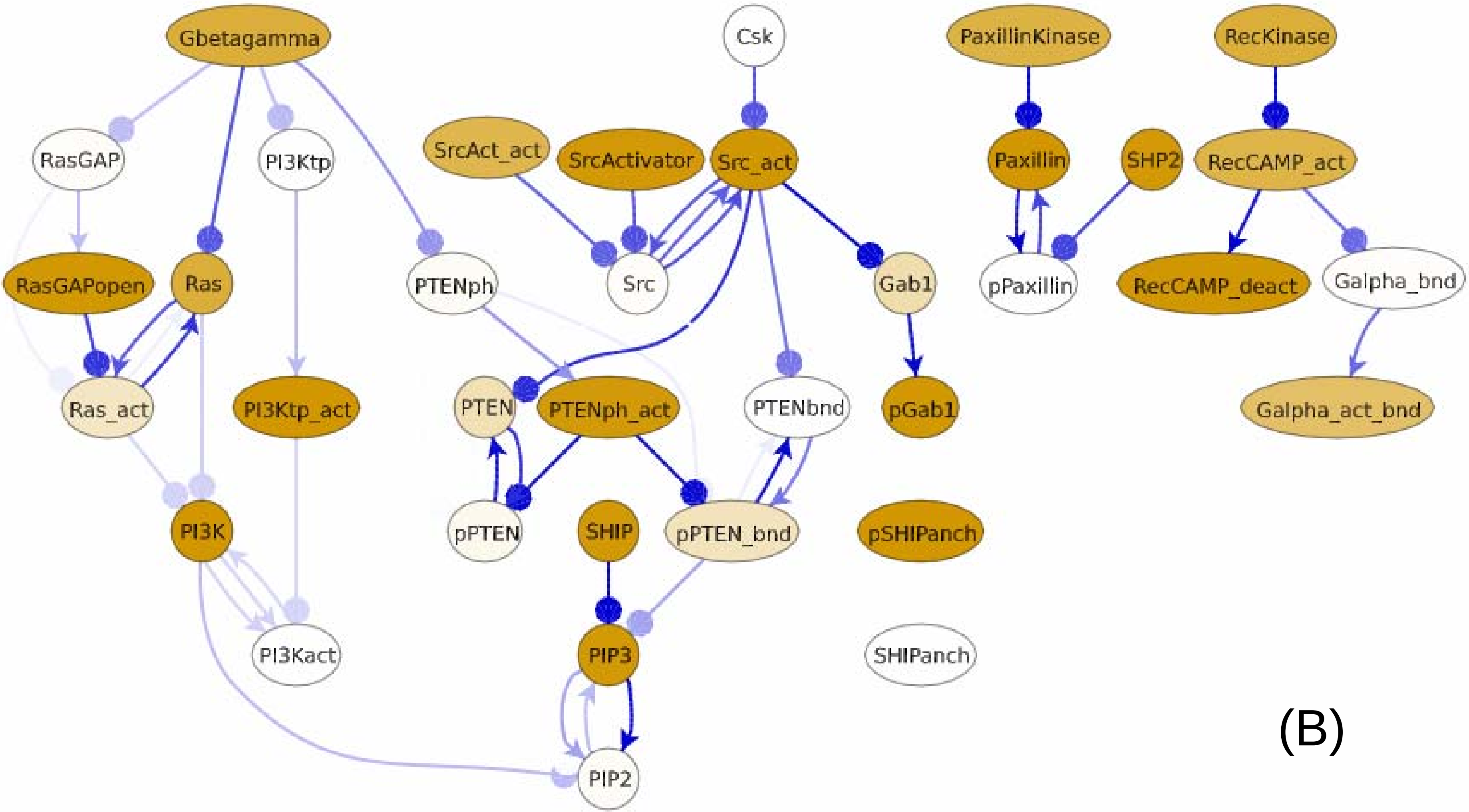

Gbetagamma
Csk
PaxillinKinase
RecKinase
RasGAP
PI3Ktp
SrcAct_act
SrcActivator
Src_act
Paxillin
SHP2
RecCAMP_act
RasGAPopen
Ras
PTENph
Src
Gab1
pPaxillin
RecCAMP_deact
Galpha_bnd
Ras_act
PI3Ktp_act
PTEN
PTENph_act
PTENbnd
pGab1
Galpha_act_bnd
pPTEN
SHIP
pPTEN_bnd
pSHIPanch
PI3K
PI3Kact
PIP3
SHIPanch
PIP2

(B)
