# Supplementary material for: Key Role of Local Regulation in Chemosensing Revealed by a New Molecular Interaction-Based Modeling Method
Source: PLoS Comput Biol. 2006 Jul 21;2(7):e82. doi: 10.1371/journal.pcbi.0020082 (PMC1513273; doi:10.1371/journal.pcbi.0020082)
Supplement: Supplementary file 16 [file pcbi.0020082.sg002.pdf]

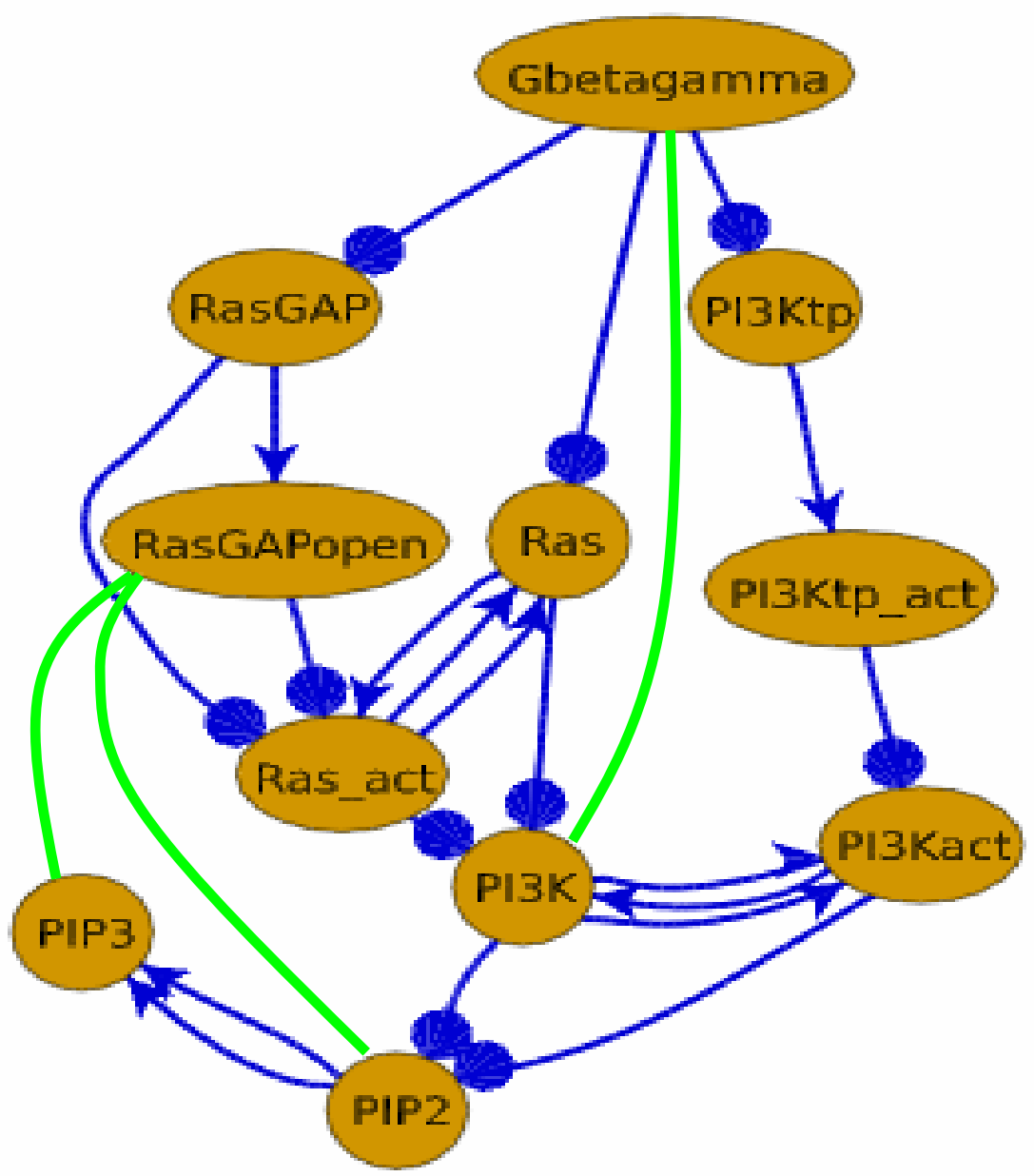
Gbetagamma
RasGAP
PI3Ktp
RasGAPopen
Ras
PI3Ktp_act
Ras_act
PI3Kact
PI3K
PIP3
PIP2
