# Supplementary material for: Key Role of Local Regulation in Chemosensing Revealed by a New Molecular Interaction-Based Modeling Method
Source: PLoS Comput Biol. 2006 Jul 21;2(7):e82. doi: 10.1371/journal.pcbi.0020082 (PMC1513273; doi:10.1371/journal.pcbi.0020082)
Supplement: Supplementary file 17 [file pcbi.0020082.sg003.pdf]

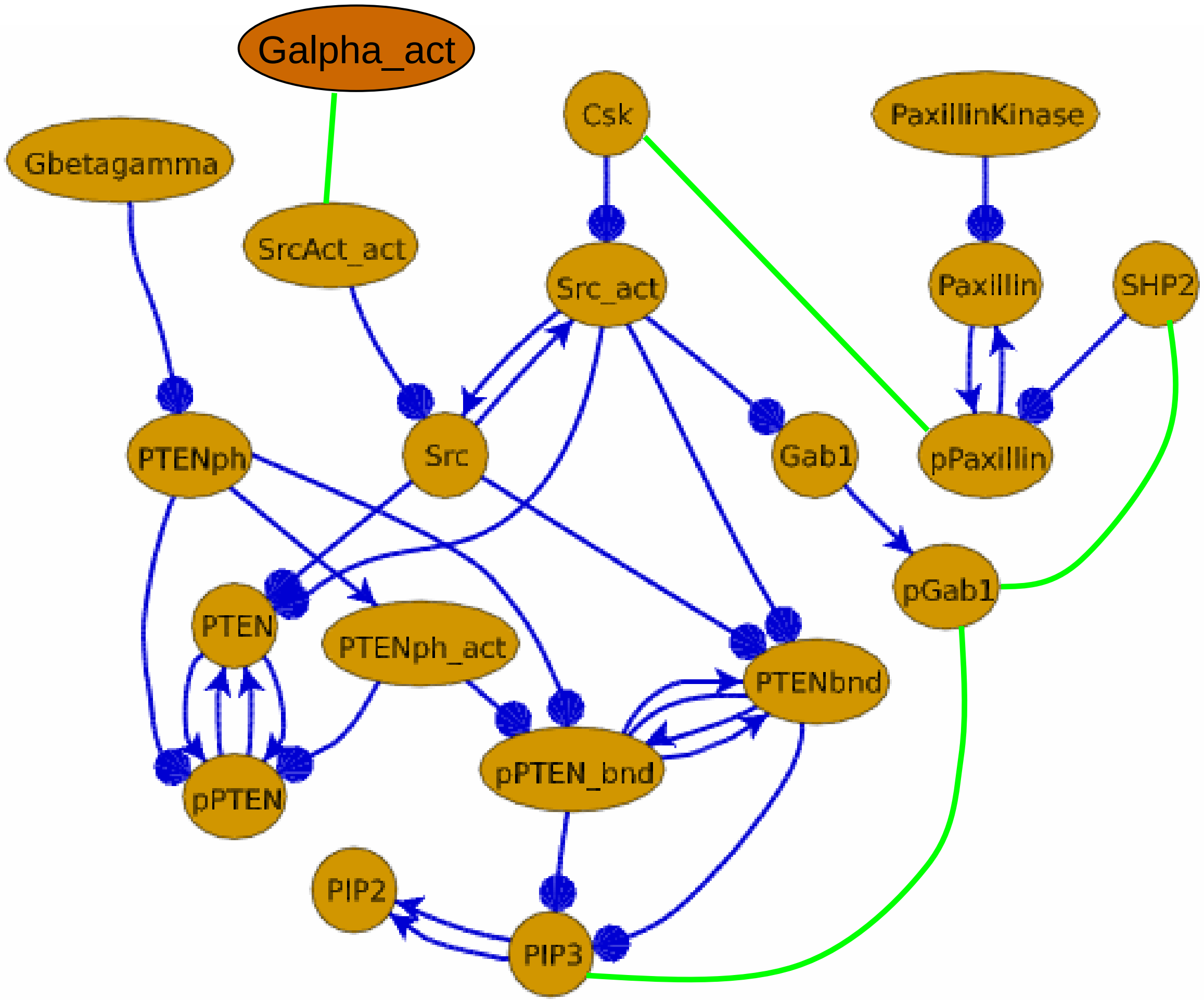
Galpha_act
Csk
PaxillinKinase
Gbetagamma
SrcAct_act
Src_act
Paxillin
SHP2
PTENph
Src
Gab1
pPaxillin
pGab1
PTEN
PTENph_act
PTENbnd
pPTEN_bnd
pPTEN
PIP2
PIP3
