# Supplementary material for: Key Role of Local Regulation in Chemosensing Revealed by a New Molecular Interaction-Based Modeling Method
Source: PLoS Comput Biol. 2006 Jul 21;2(7):e82. doi: 10.1371/journal.pcbi.0020082 (PMC1513273; doi:10.1371/journal.pcbi.0020082)
Supplement: Supplementary file 18 [file pcbi.0020082.sg004.pdf]

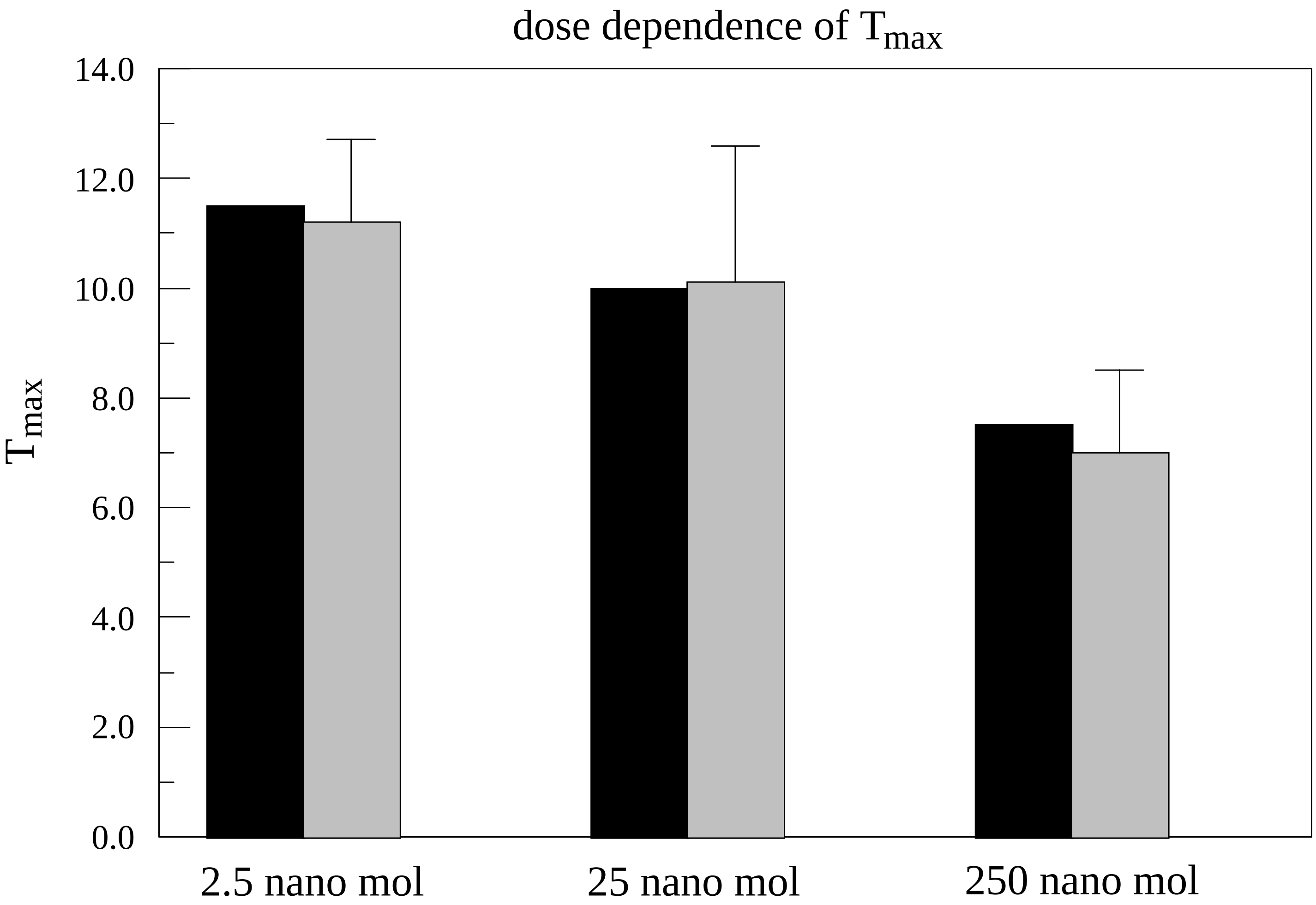
dose dependence of T_{max}
14.0
12.0
10.0
8.0
6.0
4.0
2.0
0.0
T_{max}
2.5 nano mol
25 nano mol
250 nano mol
