# Supplementary material for: Key Role of Local Regulation in Chemosensing Revealed by a New Molecular Interaction-Based Modeling Method
Source: PLoS Comput Biol. 2006 Jul 21;2(7):e82. doi: 10.1371/journal.pcbi.0020082 (PMC1513273; doi:10.1371/journal.pcbi.0020082)
Supplement: Supplementary file 19 [file pcbi.0020082.sg005.pdf]

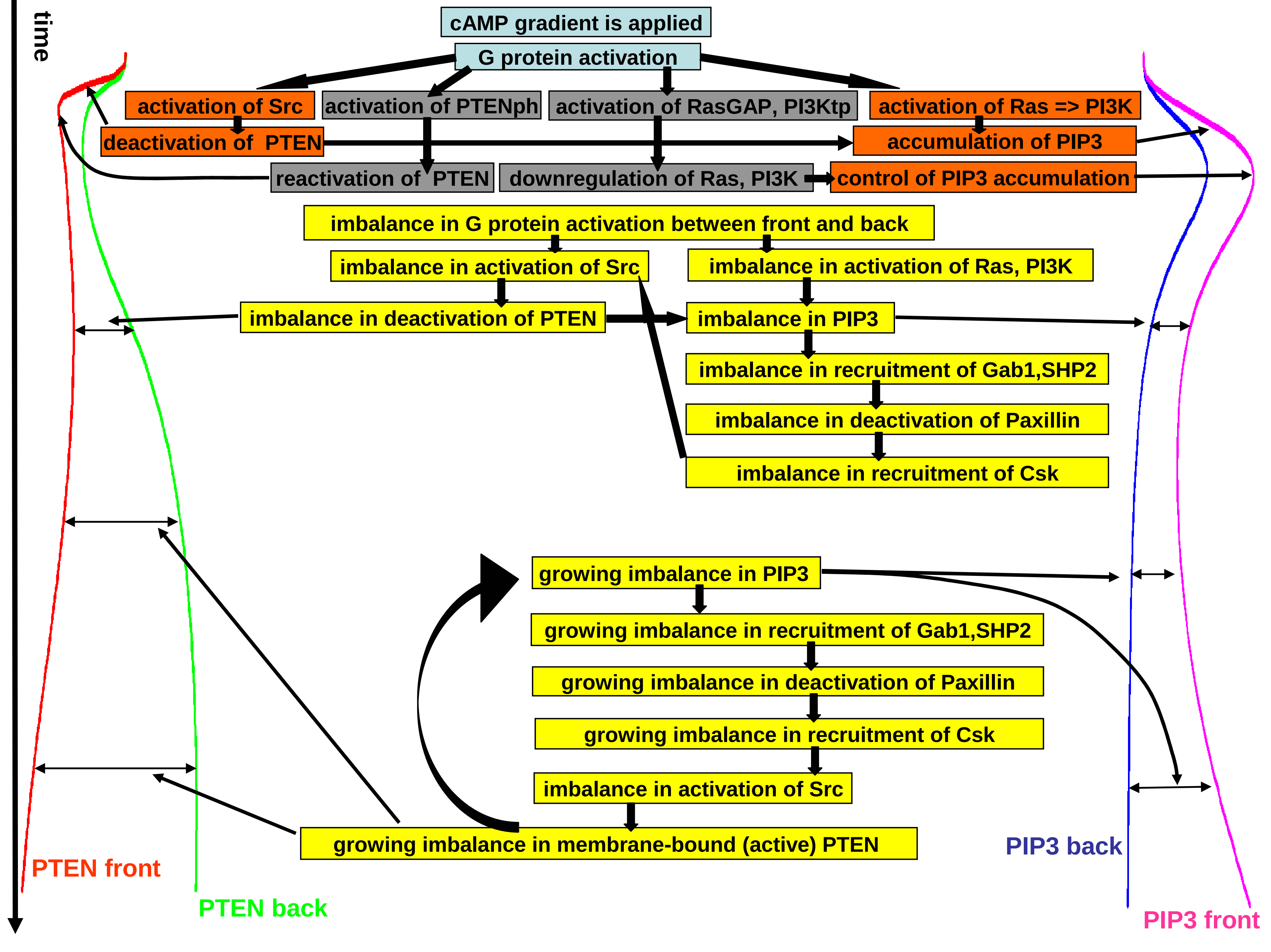
time
cAMP gradient is applied
G protein activation
activation of Src
activation of PTENph
activation of RasGAP, PI3Ktp
activation of Ras => PI3K
deactivation of PTEN
accumulation of PIP3
reactivation of PTEN
downregulation of Ras, PI3K
control of PIP3 accumulation
imbalance in G protein activation between front and back
imbalance in activation of Src
imbalance in activation of Ras, PI3K
imbalance in deactivation of PTEN
imbalance in PIP3
imbalance in recruitment of Gab1,SHP2
imbalance in deactivation of Paxillin
imbalance in recruitment of Csk
growing imbalance in PIP3
growing imbalance in recruitment of Gab1,SHP2
growing imbalance in deactivation of Paxillin
growing imbalance in recruitment of Csk
imbalance in activation of Src
growing imbalance in membrane-bound (active) PTEN
PTEN front
PTEN back
PIP3 back
PIP3 front
