# Supplementary material for: Key Role of Local Regulation in Chemosensing Revealed by a New Molecular Interaction-Based Modeling Method
Source: PLoS Comput Biol. 2006 Jul 21;2(7):e82. doi: 10.1371/journal.pcbi.0020082 (PMC1513273; doi:10.1371/journal.pcbi.0020082)
Supplement: Supplementary file 20 [file pcbi.0020082.sg006.pdf]

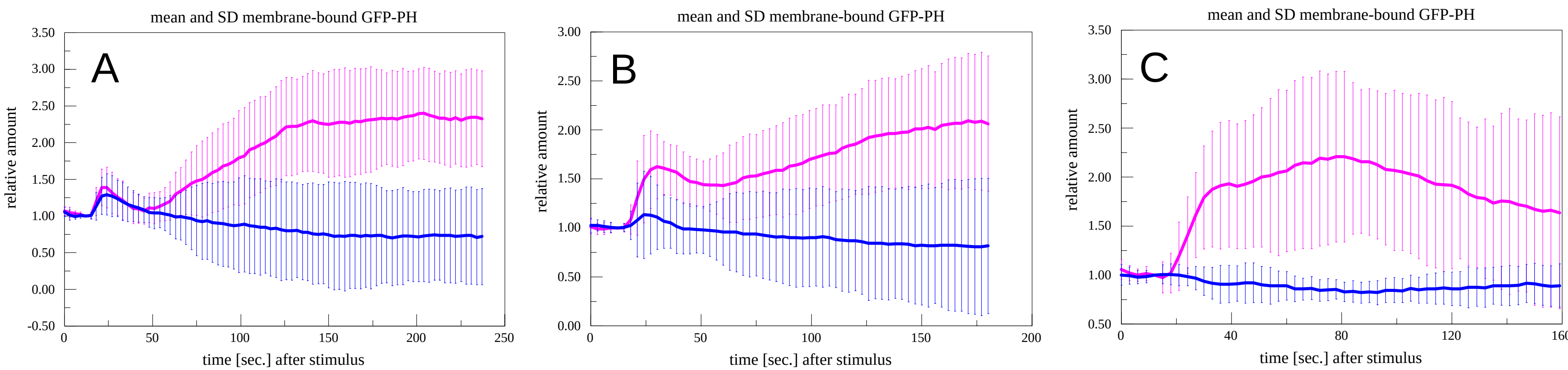
mean and SD membrane-bound GFP-PH
A
relative amount
3.50
3.00
2.50
2.00
1.50
1.00
0.50
0.00
-0.50
0
50
100
150
200
250
time [sec.] after stimulus
mean and SD membrane-bound GFP-PH
B
relative amount
3.00
2.50
2.00
1.50
1.00
0.50
0.00
0
50
100
150
200
time [sec.] after stimulus
mean and SD membrane-bound GFP-PH
C
relative amount
3.50
3.00
2.50
2.00
1.50
1.00
0.50
0
40
80
120
160
time [sec.] after stimulus
