# Supplementary material for: Key Role of Local Regulation in Chemosensing Revealed by a New Molecular Interaction-Based Modeling Method
Source: PLoS Comput Biol. 2006 Jul 21;2(7):e82. doi: 10.1371/journal.pcbi.0020082 (PMC1513273; doi:10.1371/journal.pcbi.0020082)
Supplement: Supplementary file 21 [file pcbi.0020082.sg007.pdf]

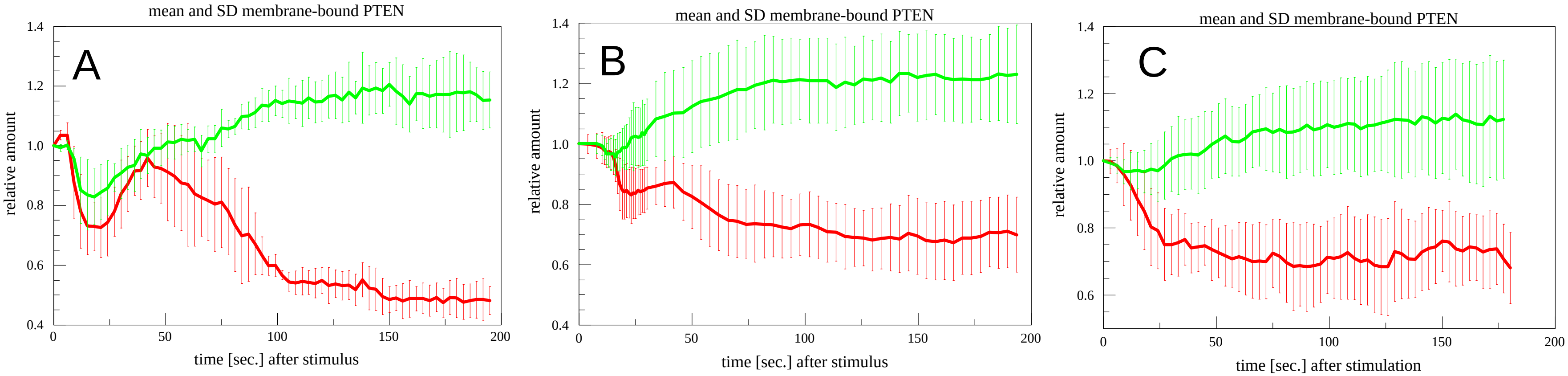

mean and SD membrane-bound PTEN
A
relative amount
time [sec.] after stimulus
0.4
0.6
0.8
1.0
1.2
1.4
0
50
100
150
200
mean and SD membrane-bound PTEN
B
relative amount
time [sec.] after stimulus
mean and SD membrane-bound PTEN
C
relative amount
time [sec.] after stimulation
