# Supplementary material for: Key Role of Local Regulation in Chemosensing Revealed by a New Molecular Interaction-Based Modeling Method
Source: PLoS Comput Biol. 2006 Jul 21;2(7):e82. doi: 10.1371/journal.pcbi.0020082 (PMC1513273; doi:10.1371/journal.pcbi.0020082)
Supplement: Supplementary file 22 [file pcbi.0020082.sg008.pdf]

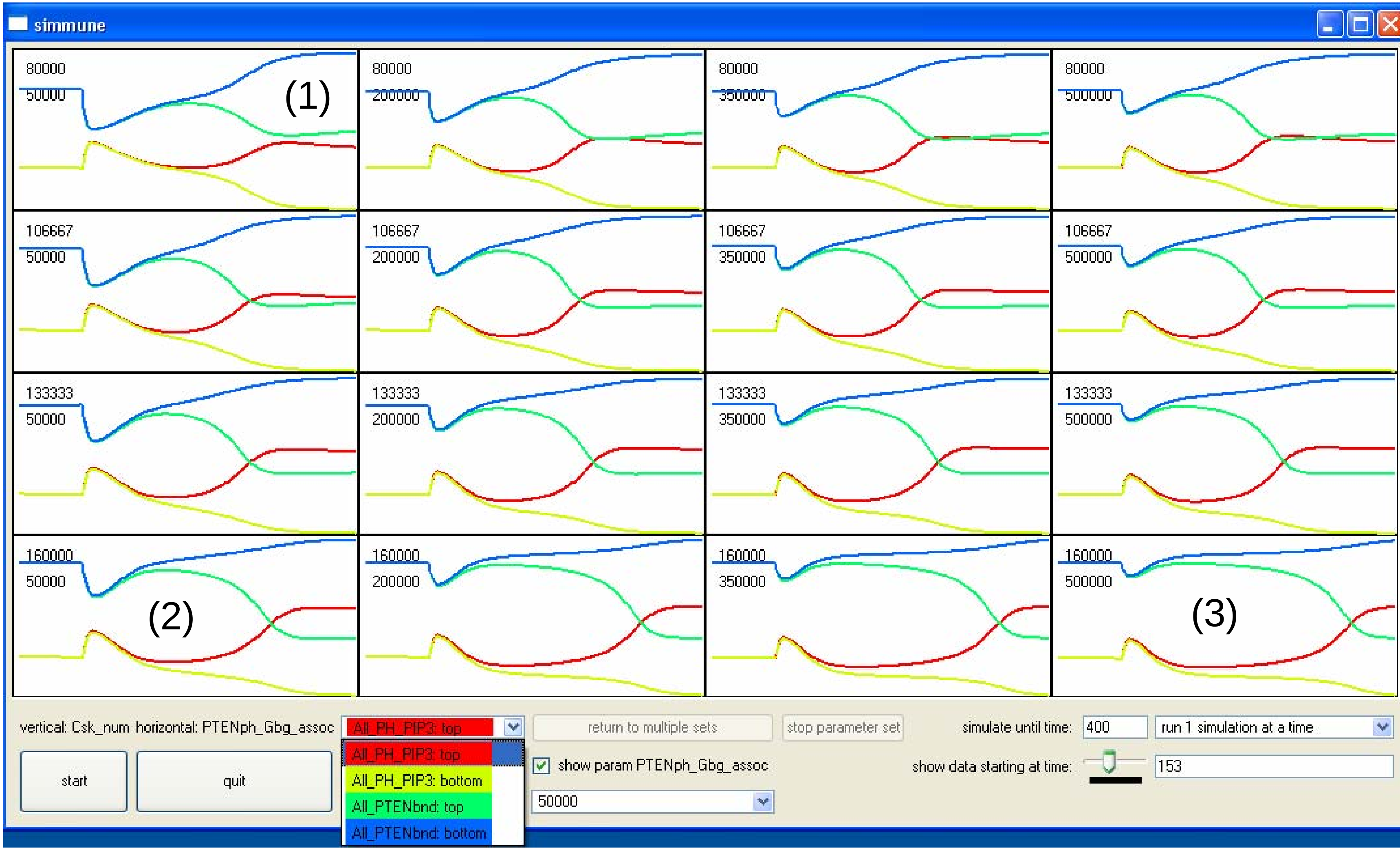

simmune
80000
50000
(1)
80000
200000
80000
350000
80000
500000
106667
50000
106667
200000
106667
350000
106667
500000
133333
50000
133333
200000
133333
350000
133333
500000
160000
50000
(2)
160000
200000
160000
350000
160000
500000
(3)
vertical: Csk_num horizontal: PTENph_Gbg_assoc
All_PH_PIP3: top
All_PH_PIP3: top
All_PH_PIP3: bottom
All_PTENbnd: top
All_PTENbnd: bottom
return to multiple sets
stop parameter set
simulate until time:
400
run 1 simulation at a time
start
quit
show param PTENph_Gbg_assoc
50000
show data starting at time:
153

(A)

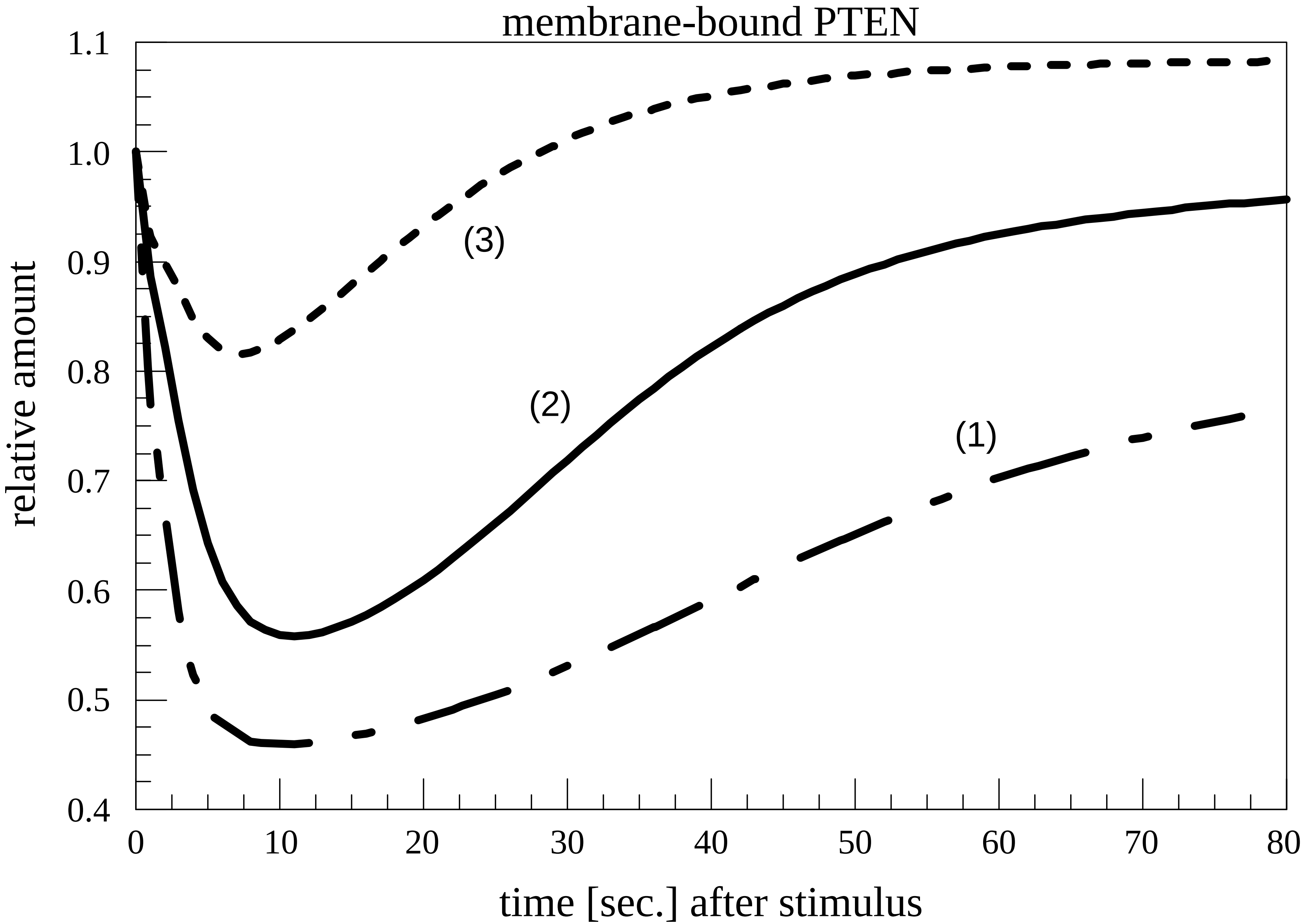
membrane-bound PTEN
relative amount
1.1
1.0
0.9
0.8
0.7
0.6
0.5
0.4
(3)
(2)
(1)
0
10
20
30
40
50
60
70
80
time [sec.] after stimulus

(B)
