# Supplementary material for: Key Role of Local Regulation in Chemosensing Revealed by a New Molecular Interaction-Based Modeling Method
Source: PLoS Comput Biol. 2006 Jul 21;2(7):e82. doi: 10.1371/journal.pcbi.0020082 (PMC1513273; doi:10.1371/journal.pcbi.0020082)
Supplement: Supplementary file 23 [file pcbi.0020082.sg009.pdf]

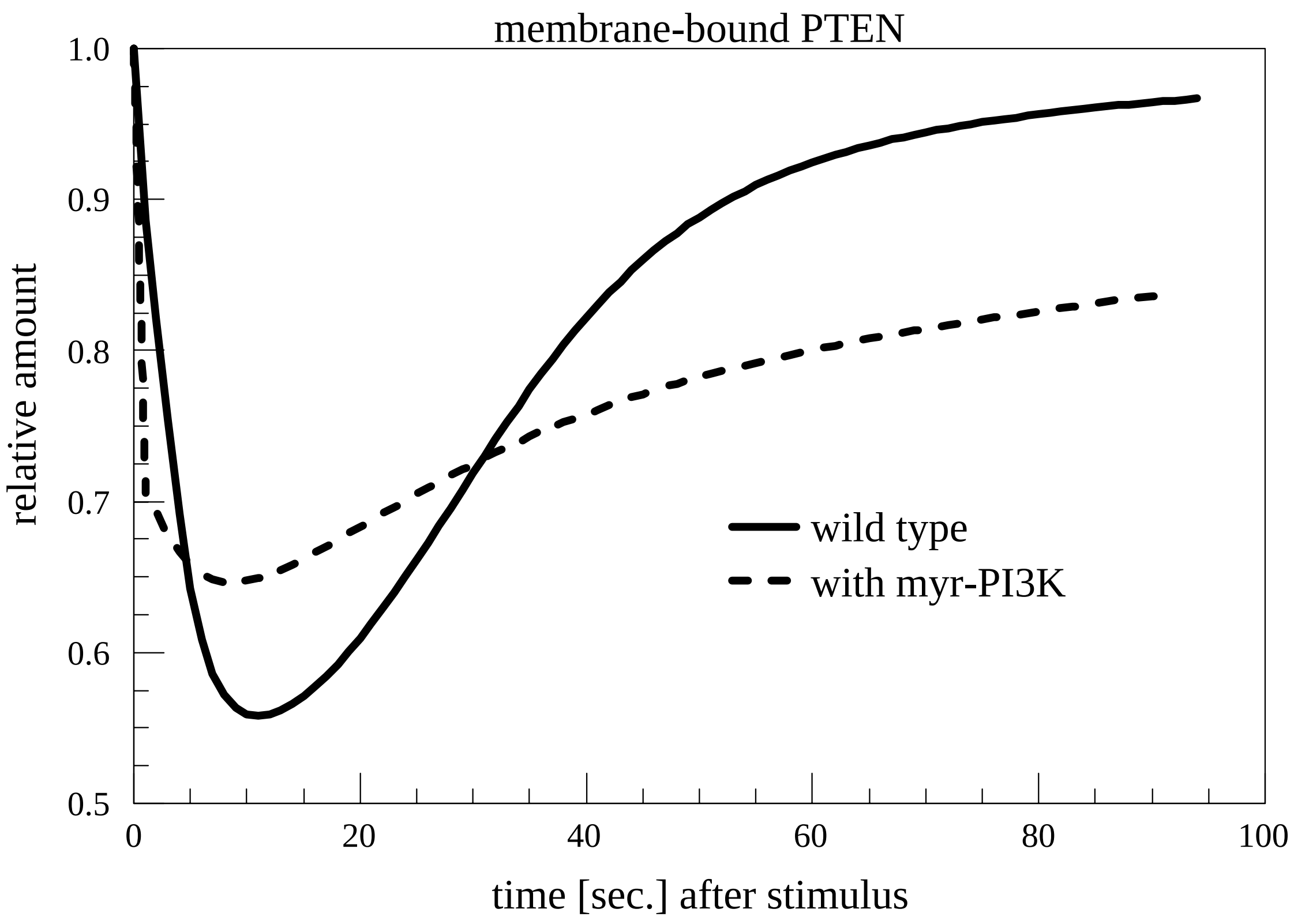
membrane-bound PTEN
1.0
0.9
0.8
0.7
0.6
0.5
relative amount
0
20
40
60
80
100
time [sec.] after stimulus
wild type
with myr-PI3K
