# Supplementary material for: Key Role of Local Regulation in Chemosensing Revealed by a New Molecular Interaction-Based Modeling Method
Source: PLoS Comput Biol. 2006 Jul 21;2(7):e82. doi: 10.1371/journal.pcbi.0020082 (PMC1513273; doi:10.1371/journal.pcbi.0020082)
Supplement: Supplementary file 24 [file pcbi.0020082.sg010.pdf]

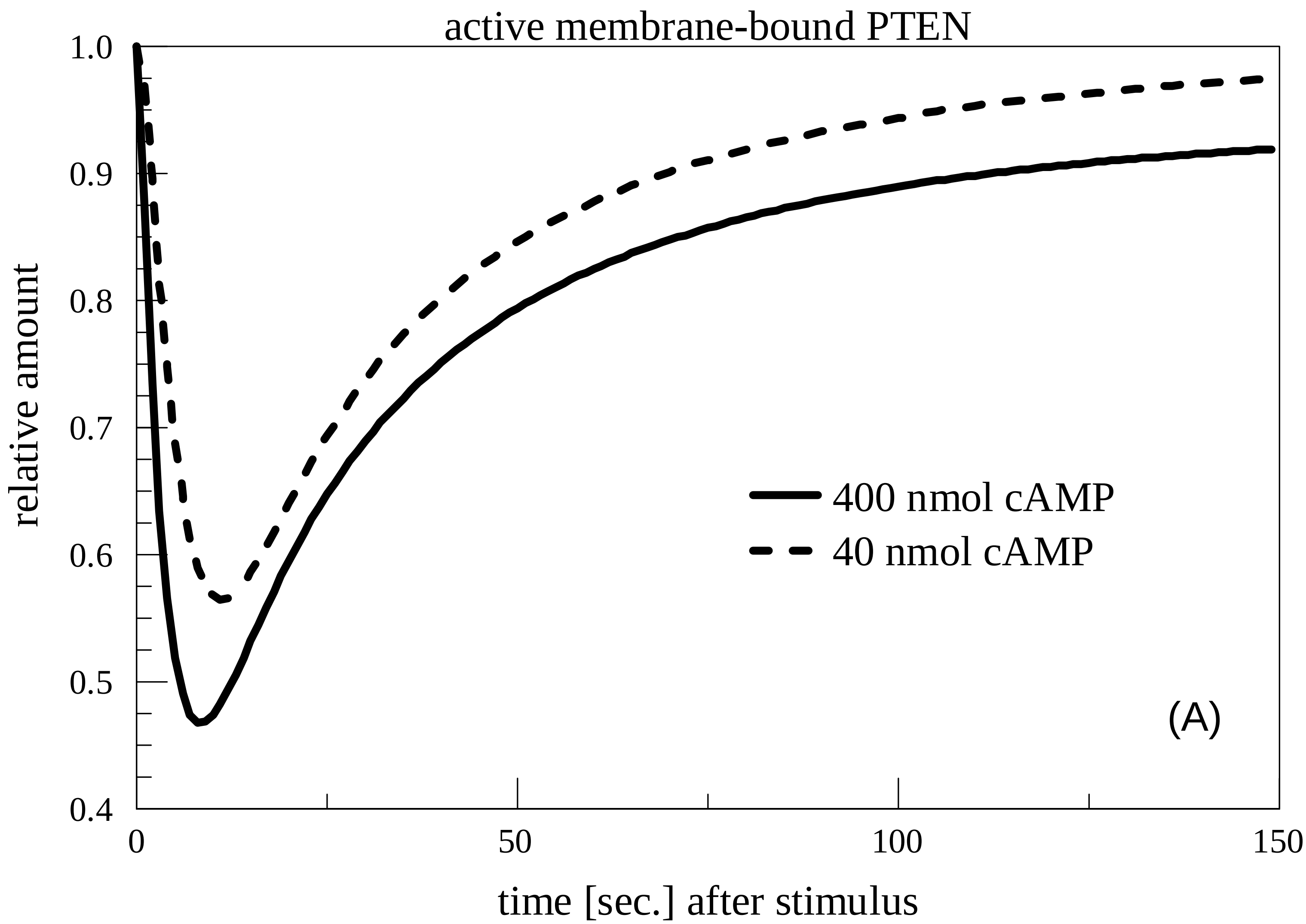
active membrane-bound PTEN
relative amount
1.0
0.9
0.8
0.7
0.6
0.5
0.4
0
50
100
150
time [sec.] after stimulus
400 nmol cAMP
40 nmol cAMP
(A)

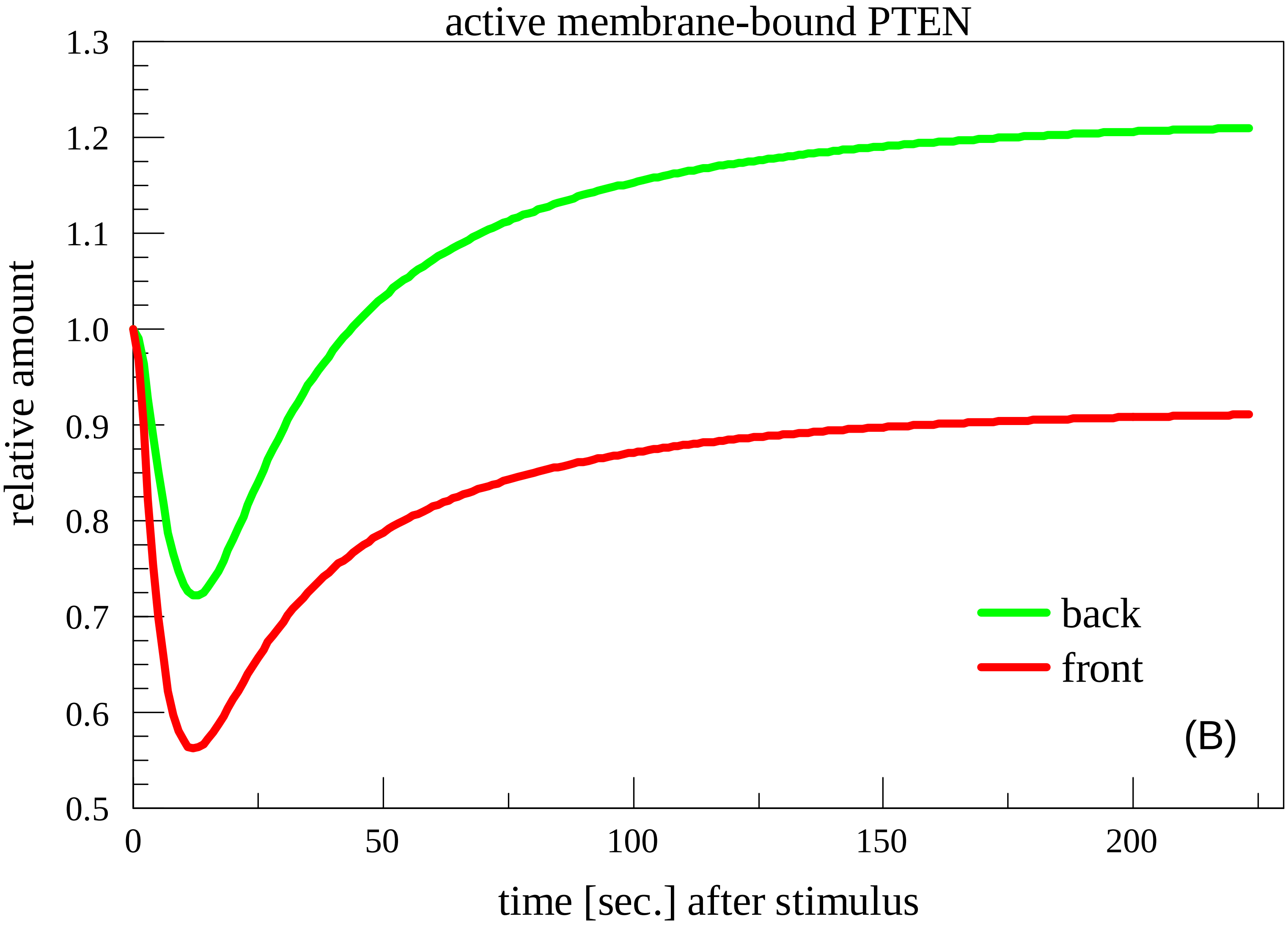
active membrane-bound PTEN
relative amount
1.3
1.2
1.1
1.0
0.9
0.8
0.7
0.6
0.5
0
50
100
150
200
time [sec.] after stimulus
back
front
(B)

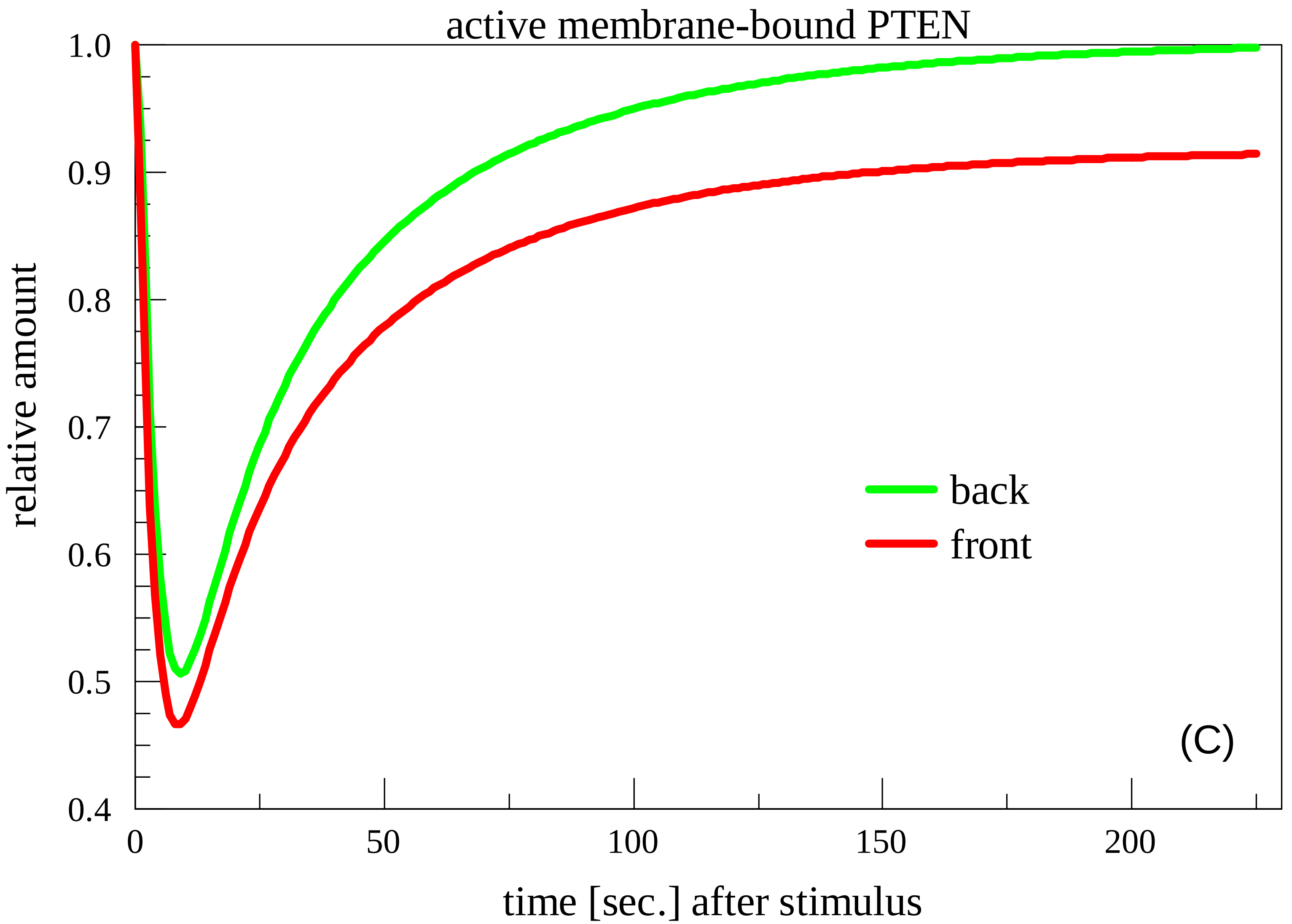

active membrane-bound PTEN
1.0
0.9
0.8
0.7
0.6
0.5
0.4
relative amount
0
50
100
150
200
time [sec.] after stimulus
back
front
(C)
