# Supplementary material for: Key Role of Local Regulation in Chemosensing Revealed by a New Molecular Interaction-Based Modeling Method
Source: PLoS Comput Biol. 2006 Jul 21;2(7):e82. doi: 10.1371/journal.pcbi.0020082 (PMC1513273; doi:10.1371/journal.pcbi.0020082)
Supplement: Supplementary file 25 [file pcbi.0020082.sg011.pdf]

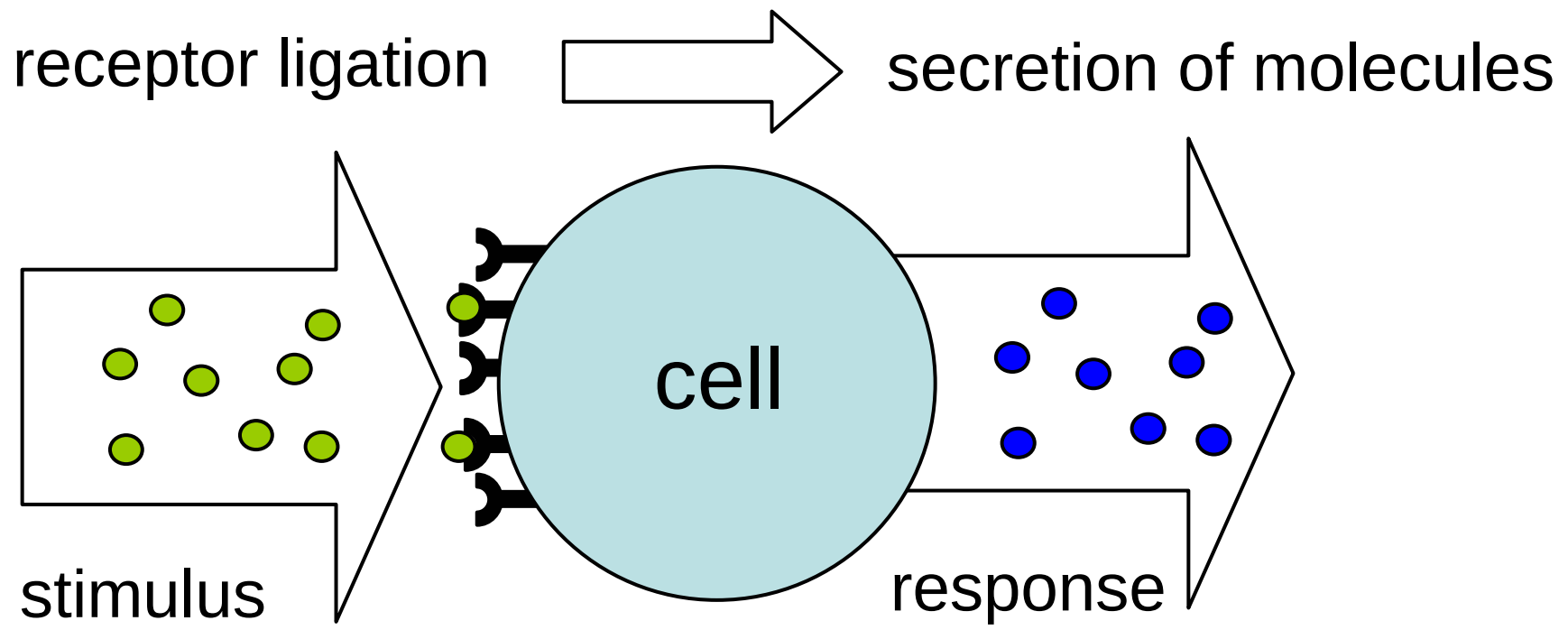
receptor ligation
secretion of molecules
cell
stimulus
response

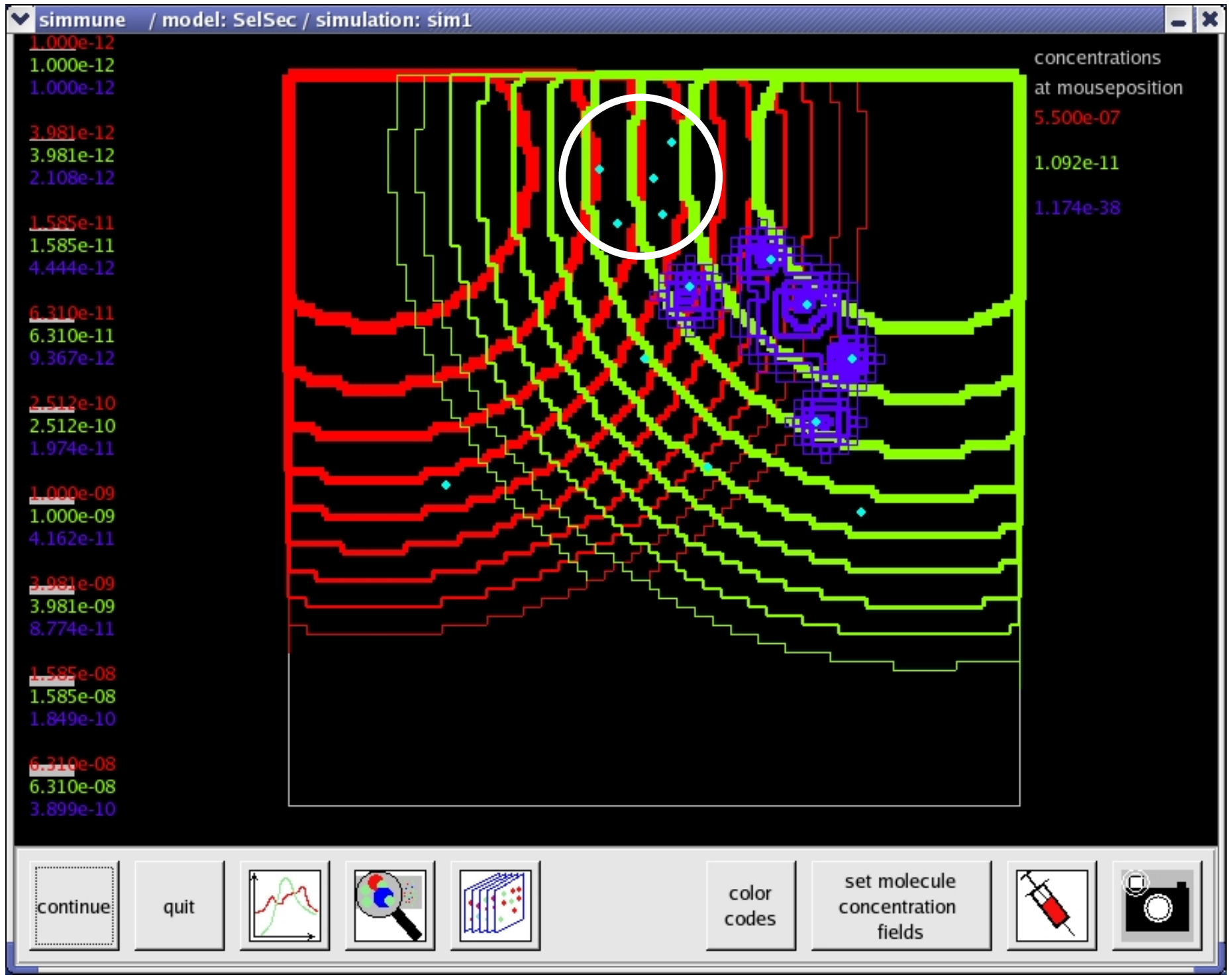
simmune / model: SelSec / simulation: sim1
1.000e-12
1.000e-12
1.000e-12
3.981e-12
3.981e-12
2.108e-12
1.585e-11
1.585e-11
4.444e-12
6.310e-11
6.310e-11
9.367e-12
2.512e-10
2.512e-10
1.974e-11
1.000e-09
1.000e-09
4.162e-11
3.981e-09
3.981e-09
8.774e-11
1.585e-08
1.585e-08
1.849e-10
6.310e-08
6.310e-08
3.899e-10
concentrations
at mouseposition
5.500e-07
1.092e-11
1.174e-38
continue
quit
color codes
set molecule concentration fields
