# Supplementary material for: Key Role of Local Regulation in Chemosensing Revealed by a New Molecular Interaction-Based Modeling Method
Source: PLoS Comput Biol. 2006 Jul 21;2(7):e82. doi: 10.1371/journal.pcbi.0020082 (PMC1513273; doi:10.1371/journal.pcbi.0020082)
Supplement: Supplementary file 26 [file pcbi.0020082.sg012.pdf]

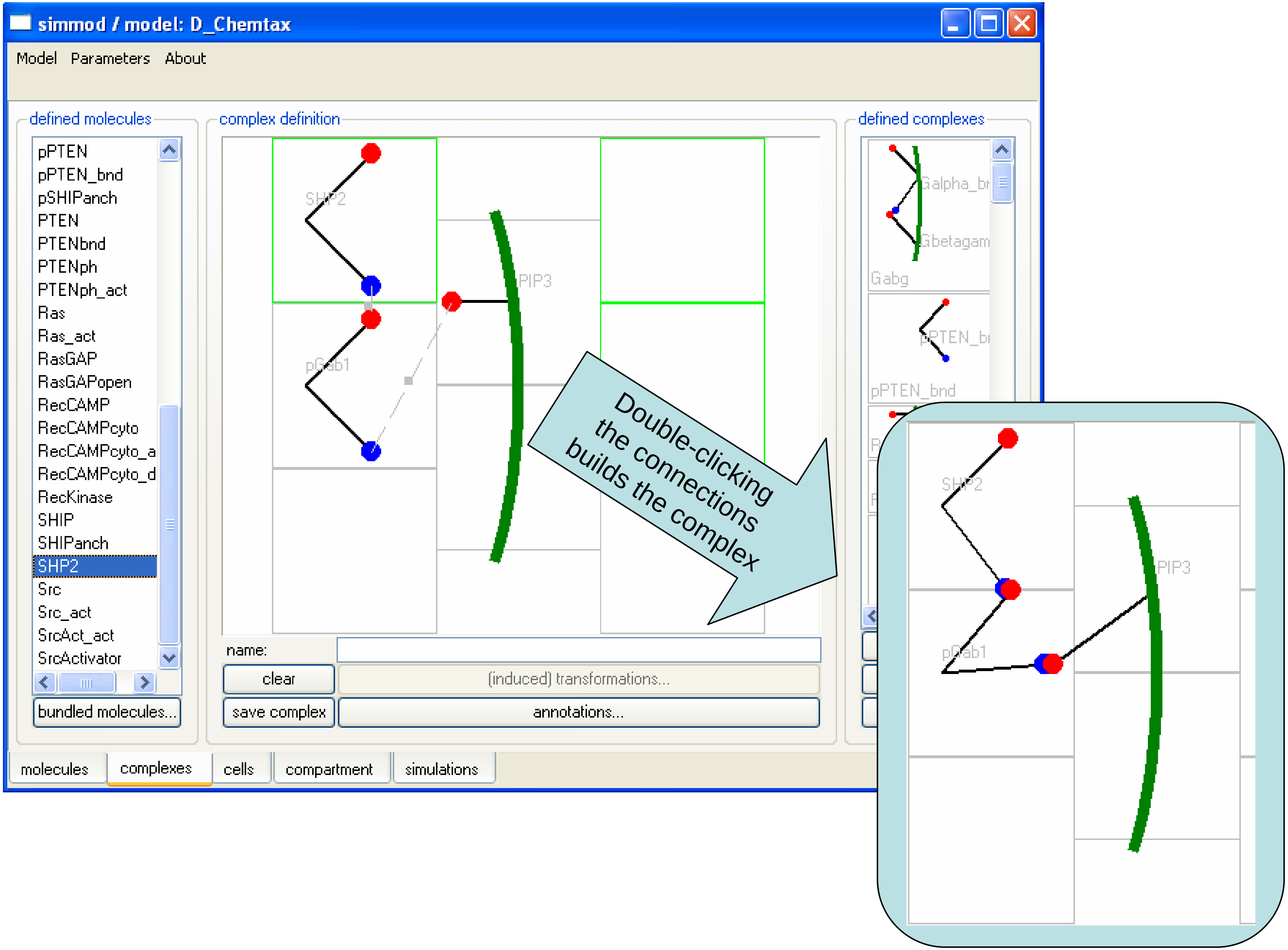
simmod / model: D_Chemtax
Model Parameters About
defined molecules
pPTEN
pPTEN_bnd
pSHIPanch
PTEN
PTENbnd
PTENph
PTENph_act
Ras
Ras_act
RasGAP
RasGAPopen
RecCAMP
RecCAMPcyto
RecCAMPcyto_a
RecCAMPcyto_d
RecKinase
SHIP
SHIPanch
SHP2
Src
Src_act
SrcAct_act
SrcActivator
bundled molecules...
complex definition
SHP2
pGab1
PIP3
name:
clear
(induced) transformations...
save complex
annotations...
defined complexes
Galpha_br
Gbetagam
Gabg
pPTEN_bi
pPTEN_bnd
molecules
complexes
cells
compartment
simulations
Double-clicking the connections builds the complex
