# Supplementary material for: Key Role of Local Regulation in Chemosensing Revealed by a New Molecular Interaction-Based Modeling Method
Source: PLoS Comput Biol. 2006 Jul 21;2(7):e82. doi: 10.1371/journal.pcbi.0020082 (PMC1513273; doi:10.1371/journal.pcbi.0020082)
Supplement: Supplementary file 27 [file pcbi.0020082.sg013.pdf]

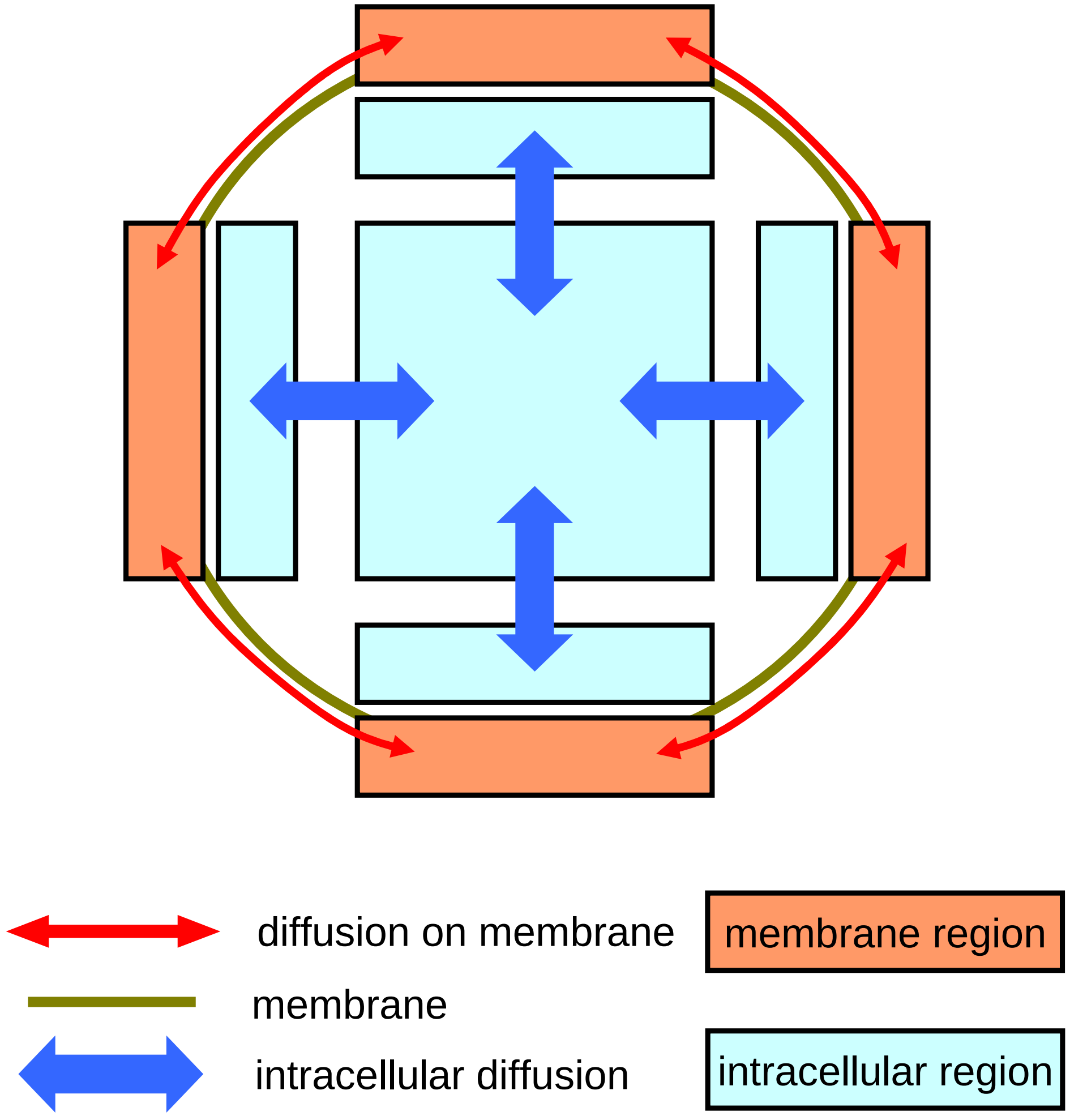
diffusion on membrane
membrane
intracellular diffusion
membrane region
intracellular region

(A)

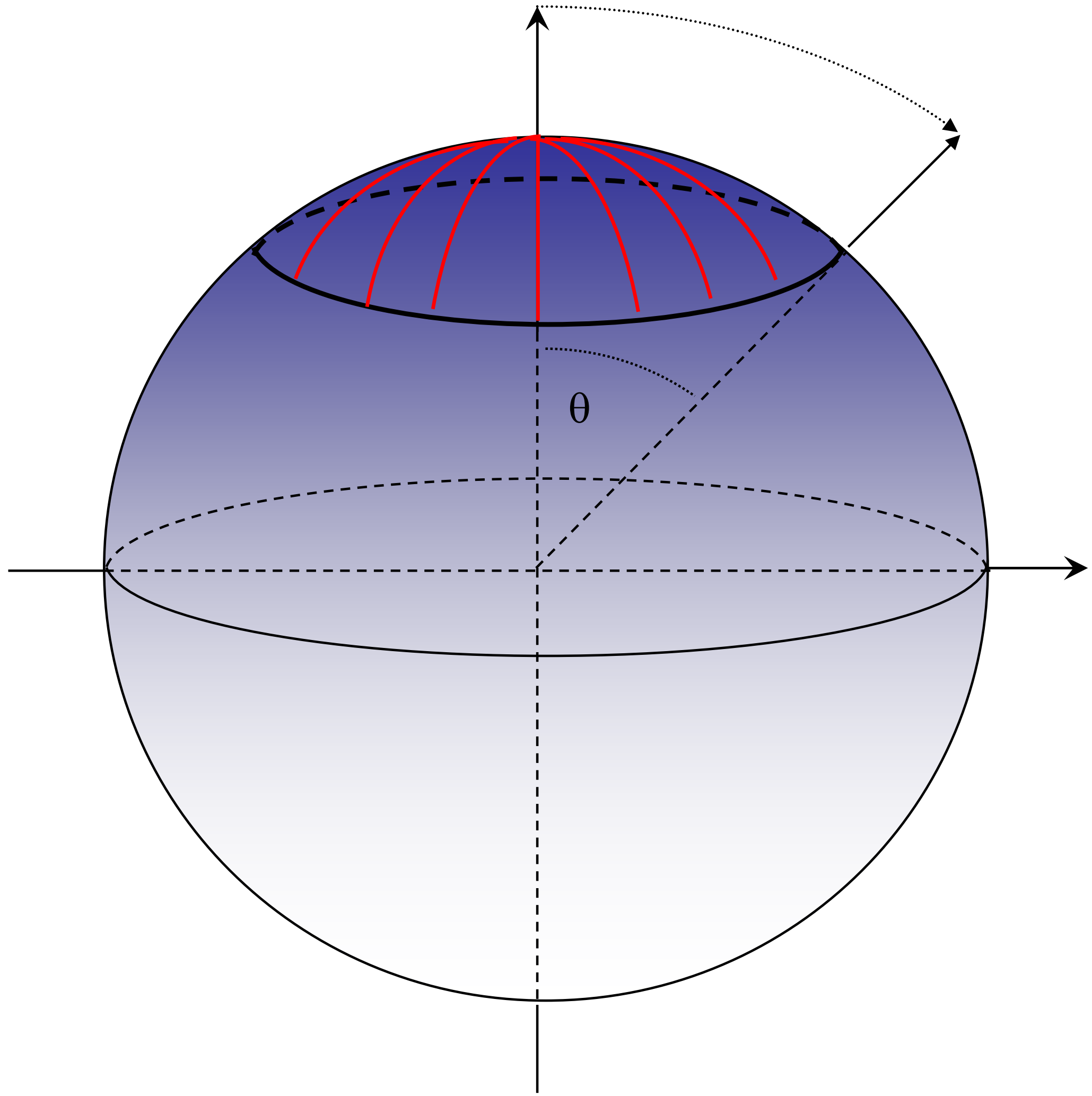

(B)

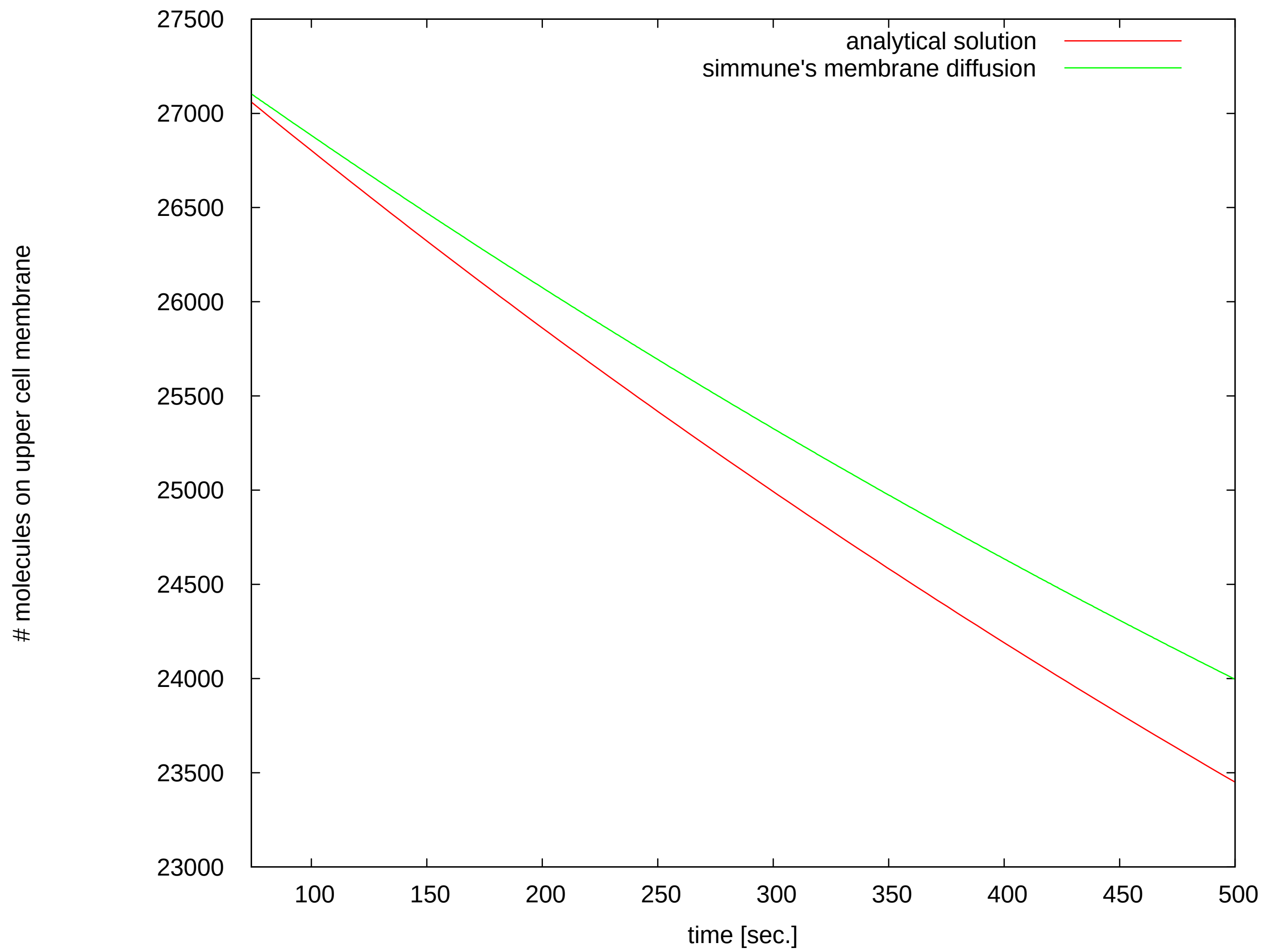
analytical solution
simmune's membrane diffusion
molecules on upper cell membrane
time [sec.]
27500
27000
26500
26000
25500
25000
24500
24000
23500
23000
100
150
200
250
300
350
400
450
500

(C)
